# Nickel‐Catalyzed Reductive Cross‐Coupling of Xanthate Esters With Aryl and Alkenyl Iodides

**DOI:** 10.1002/chem.71207

**Published:** 2026-06-06

**Authors:** Felix Vöhringer, Kathrin Zwettler, Dmitry I. Sharapa, Felix Studt, Ivana Fleischer

**Affiliations:** ^1^ Institute of Organic Chemistry Faculty of Science Eberhard Karls Universität Tübingen Tübingen Germany; ^2^ Institute of Catalysis Research and Technology Karlsruhe Institute of Technology Eggenstein‐Leopoldshafen Germany

**Keywords:** cross‐electrophile coupling, diarylmethanes, nickel catalysis, radicals, xanthates

## Abstract

We report a nickel‐catalyzed reductive cross‐coupling of xanthate esters and aryl and alkenyl iodides. Utilizing xanthate esters with an *S*‐allyl substituent leads to higher yields compared to the more commonly used *S*‐Me xanthate esters. The reaction is tolerant toward a wide array of functional groups and allows for the conversion of xanthate esters derived from pharmaceuticals to diarylmethanes. Electronic properties of the reaction partners have a strong influence on the reaction. Mechanistic investigations revealed that the benzylic radical is generated from the xanthate ester, presumably by single electron transfer by Ni(I).

## Graphical abstract

A nickel‐catalyzed reductive cross‐coupling of xanthate esters and aryl/alkenyl iodides is reported without the need for separate activators for the xanthate esters. The reaction utilizes a relatively low catalyst loading. Diarylmethanes with a good functional group variety can thus be synthesized indirectly from the corresponding benzylic alcohols. The reaction is proposed to proceed via single electron transfer by Ni(I) or Zn and the generation of a benzylic radical.

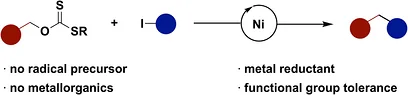

## 1 Introduction

Transition metal‐catalyzed cross‐coupling reactions are among the most common methods to form C─C bonds and can be used to efficiently and selectively generate highly functionalized molecules [1, 2, 3, 4]. These reactions are usually performed between an electrophilic organo(pseudo)halide and a nucleophilic metalorganic coupling partner. The limited commercial availability and air sensitivity of some of these nucleophiles can be addressed through their in situ synthesis from an electrophile or a cross‐electrophile coupling (XEC), in which two electrophiles can be coupled under reductive conditions [5].

Since alcohols represent one of the most prevalent functional groups in natural products [6], the development of methods for their functionalization, especially in C─C cross couplings, is desirable [7, 8]. But due to the high bond dissociation energy (BDE) of the C─O bond (∼95 kcal/mol), direct cross‐coupling reactions are challenging [9]. This can be mitigated by either prefunctionalization of the alcohol or by adding activating agents [10, 11, 12, 13, 14]. Common derivatives of alcohols for cross‐coupling reactions are mesylates, tosylates, triflates, pivalates, and methyl oxalates [15, 16, 17, 18, 19]. This prefunctionalization can occasionally be performed in situ [20].

**SCHEME 1:**
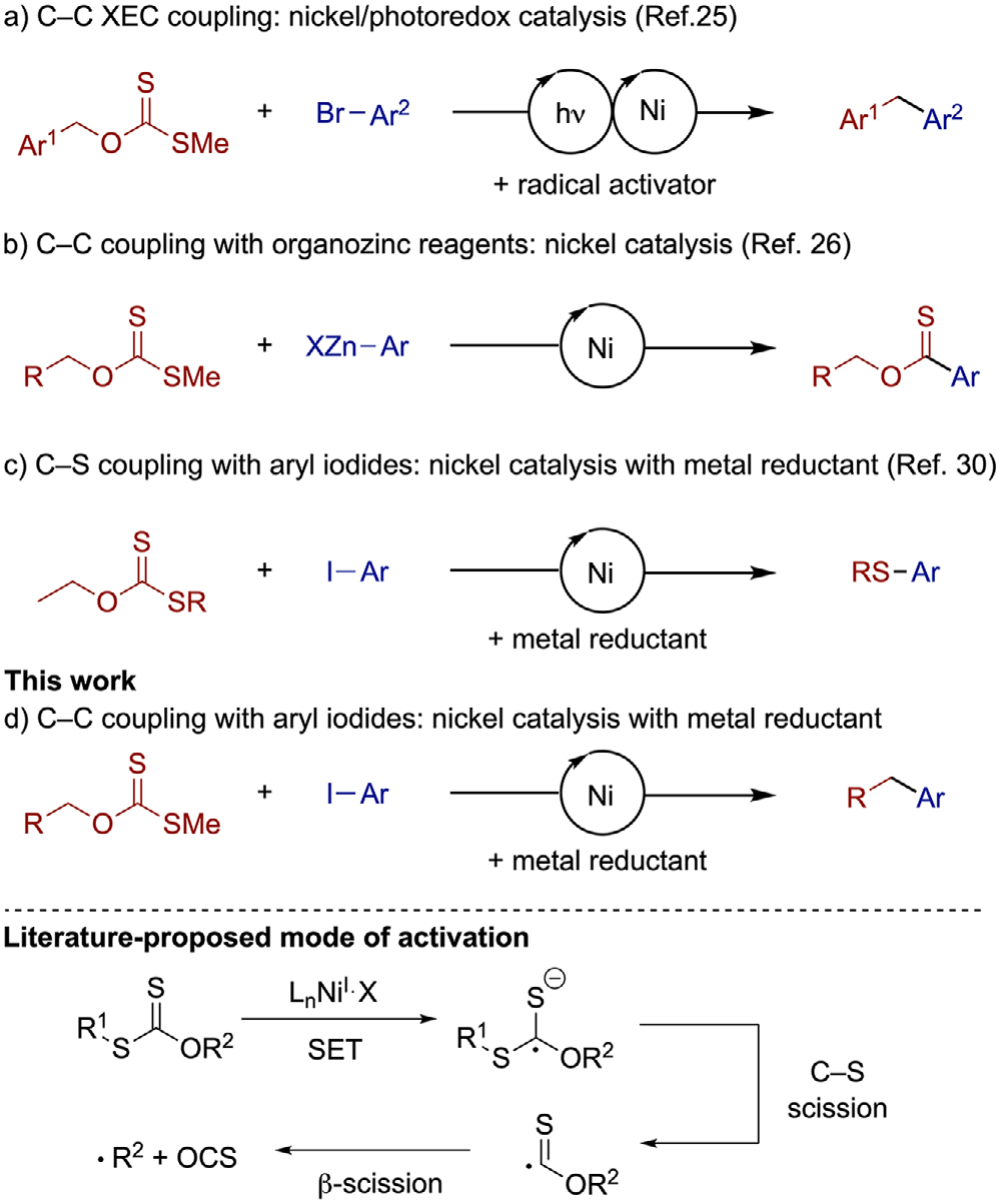
(a–c) Literature examples of cross‐coupling reactions with xanthate esters; (d) the developed method and the proposed activation by SET.

Other useful alcohol derivatives are xanthate esters, which have been recently explored as redox‐active agents in cross‐coupling reactions. They can be easily synthesized from alcohols, carbon disulfide, and an alkyl halide in the presence of a base. They can undergo a multitude of radical reactions to form a diverse array of products [21, 22]. The best known is the Barton–McCombie deoxygenation, in which the xanthate is attacked by a tributyltin radical, leading to the release of the corresponding alkyl radical [23]. The activation by radicals has been employed in C─C cross couplings by either copper catalysis with aryl diazonium salts as radical sources [24] and under nickel/photoredox catalysis with potassium trifluoroborate as a radical precursor (Scheme 1a) [24, 25]. These methods come with the disadvantage of needing an additional reaction partner to form the radical for xanthate activation.

Another mode of activation is by single electron transfer (SET), which was suggested to occur from a Ni(I) species generated in the C─C cross coupling of xanthate esters with aryl zinc salts to form thionoesters (Scheme 1b) [26]. Similar reaction conditions were employed in the arylation of thiocarbamates derived from cyclopropanols and *α*‐hydroxy esters following a similar mechanism [27, 28]. Propargylic xanthate ester also underwent cross‐coupling with arylzinc salts in low yield [29]. The SET‐enabled activation also occurred in the C─S coupling of the thiol moiety with acyl chlorides and aryl iodides to afford thioesters or thioethers (Scheme 1c) [30]. The Ni(I) species here was generated by the reduction of the precursor with zinc metal, which enabled the use of functional groups prone to reaction with metalorganic compounds.

The activation by SET is assumed to proceed by C─S scission of the reduced xanthate species, followed by *β*‐scission that liberates the corresponding alkyl radical and carbonyl sulfide [30]. We envisioned harnessing this alkyl radical in C─C cross‐coupling with aryl halides to form diarylmethanes (Scheme 1d).

## 2 Results and Discussion

The model reaction between *S*‐methyl *O*‐benzyl xanthate ester **1a^Me^** and 1.6 equiv. aryl iodide **2a** to form diarylmethane **3a** was chosen for the optimization of the reaction conditions (Table 1, entry 1). Initially, reproducibility issues were encountered when using under 10 mol% NiCl_2_(dme) and phenanthroline separately. This could be alleviated by using the preligated NiCl_2_(phen) catalyst that allowed for the reduction of the catalyst loading to 1.5 mol% without encountering reliability issues. This relatively low amount of catalyst is especially remarkable considering the possible catalyst poisoning effects exhibited by sulfur‐containing compounds that are present in this reaction. Interestingly, good yields are only achieved if the reaction is performed in a 1:1 mixture of dimethylacetamide (DMA) and EtOAc. Using these solvents separately leads either to a significantly lower yield (Table 1, entry 2) or no yield at all (Table 1, entry 3). Similar yields were achieved by using *^i^* PrOH or *^t^* BuOH as cosolvents instead of EtOAc, but catalyst loadings under 5 mol% led to irreproducible reaction outcomes.

**TABLE 1:** Screening of reaction parameters.^a^

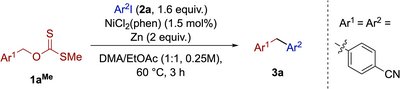		
Entry	Variations from standard conditions	Yield (%)^b^
1	none	49
2	DMA as solvent	27
3	EtOAc as solvent	n. d.
4	2 equiv. Ar^2^I	58
5	3 equiv. Ar^2^I	70
6	4 equiv. Ar^2^I	69
7	1.6 equiv. Ar^2^Cl	n. d.
8	1.6 equiv. Ar^2^Br	n. d.
9	1.6 equiv. Ar^2^OTf	n. d.
10	*S*–Allyl instead of S–Me	65
11	*S*–Bn instead of *S*–Me	67
12	Mn instead of Zn	27
13	Me_4_TMS_2_DHP instead of Zn	29
14	*S*–Allyl, 3 equiv. Ar^2^I	quant.

^a^ Reaction conditions: **1a** (0.25 mmol), Ar^2^I (3 equiv.), NiCl_2_(phen) (1.5 mol%), Zn (2 equiv.) in 1 mL DMA/EtOAc (1:1) at 60°C for 3 h.

^b^ Determined by GC‐FID with *n*‐pentadecane as an internal standard.

More equivalents of aryl iodide lead to an increase in yield that reaches a maximum at 3 equiv. (Table 1, entries 4–6) Other aryl halides and aryl triflates did not lead to product formation, possibly due to insufficient activation, as low conversions of these (pseudo)halides were observed (Table 1, entries 7–9). This selectivity toward aryl iodides could possibly be explained by the tendency of aryl iodides to undergo stepwise SET (single electron transfer) oxidative addition, while other aryl halides preferably undergo concerted oxidative addition [31]. The variation of the methyl group on the xanthate sulfur atom to an allyl or benzyl group increased the yield (Table 1, entries 10 and 11). Further experiments were performed with the allyl xanthate due to atom‐economic reasons. Manganese, as another heterogeneous reductant, led to a lower yield, and 2,3,5,6‐tetramethyl‐1,4‐bis(trimethylsilyl)‐1,4‐dihydropyrazine (Me_4_TMS_2_DHP), as a homogenous organic reductant, also performed worse than zinc (Table 1, entries 12 and 13). Combination of 3 equiv. of aryl iodide and allyl xanthate resulted in a quantitative yield.

**SCHEME 2:**
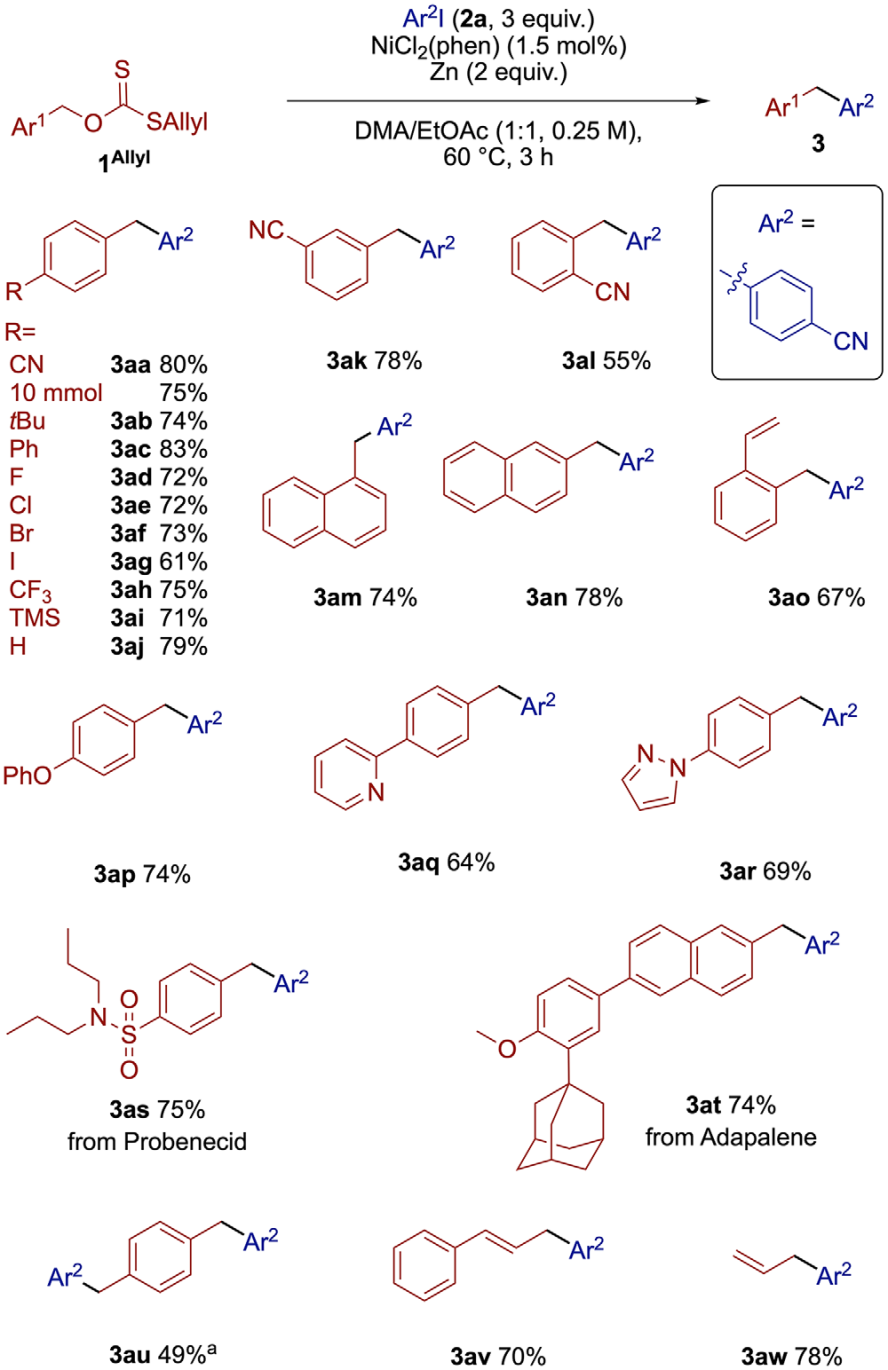
Substrate scope of *O*‐benzyl xanthates. Standard conditions: **1^Allyl^** (1 mmol), **2a** (3 equiv.), NiCl_2_(phen) (1.5 mol%), Zn (2 equiv.), 0.5 mL DMA/0.5 mL EtOAc, 60°C, 3 h. ^a^ **2a** (6 equiv.), NiCl_2_(phen) (3 mol%), Zn (4 equiv.), 0.5 mL DMA/0.5 mL EtOAc, 60°C, 3 h.

With the optimized reaction conditions in hand, the scope of xanthate esters was investigated (Scheme 2). The reaction in general only proceeds with primary benzylic xanthate esters. Decomposition of the xanthate is also observed with non‐benzylic substrates, but no coupling with the aryl iodide occurs. The standard product **3aa** could be isolated in 80% yield and in 75% when upscaled to 10 mmol. Substrates with no substituents, simple alkyl and aryl groups in the para position were well tolerated to form diarylmethanes **3ab** and **3ac** in similar yields. Halide substituents were also well tolerated, with **3ad**, **3ae**, and **3af** being synthesized in comparable yields. Even the iodoarene **3ag** was furnished in high yield despite the coupling partner being an aryl iodide as well. This selectivity stems from the electron‐rich properties of the iodo‐substituted xanthate compared to the aryl iodide coupling partner. Trifluoromethyl (**3ah**) and trimethylsilyl (**3ai**) groups also had no negative impact on the reaction. Next, the influence of the position of the electron‐withdrawing cyanide group was evaluated. While the *meta*‐substituted nitrile **3ak** was obtained in a similar yield to the corresponding *para*‐ compound, the *ortho*‐substitution resulted in a lower yield of **3al,** possibly due to steric reasons. Both naphthylmethanes, **3am**, **3an,** were obtained in similar yields, with the 1‐naphthyl furnishing a slightly lower yield, possibly again due to steric hindrance. The *ortho*‐vinyl‐substituted diarylmethane **3ao** was synthesized in a lower yield than the standard product. Moreover, the reaction could also be performed in the presence of an electron‐donating phenoxy‐group (**3ap**), even though electron‐donating substituents were generally not suitable for the reaction. This could be due to the instability of the electron‐rich xanthate esters [25].

Heterocyclic products **3aq** and **3ar** could also be obtained in good to acceptable yields. Xanthates derived from pharmaceutical agents also underwent the coupling to produce the diarylmethanes **3as** and **3at** in good yields. Double arylation of a bisxanthate afforded product **3au** in an expectedly lower yield. Besides O‐benzyl xanthates, O‐allyl xanthates can also be arylated under these conditions. O‐Allyl xanthates have the disadvantage of relatively fast decomposition under ambient conditions. They can also not be purified by column chromatography and have to be used crude. Nevertheless, allylated arenes **3av** and **3aw** were obtained in good yields.

**SCHEME 3:**
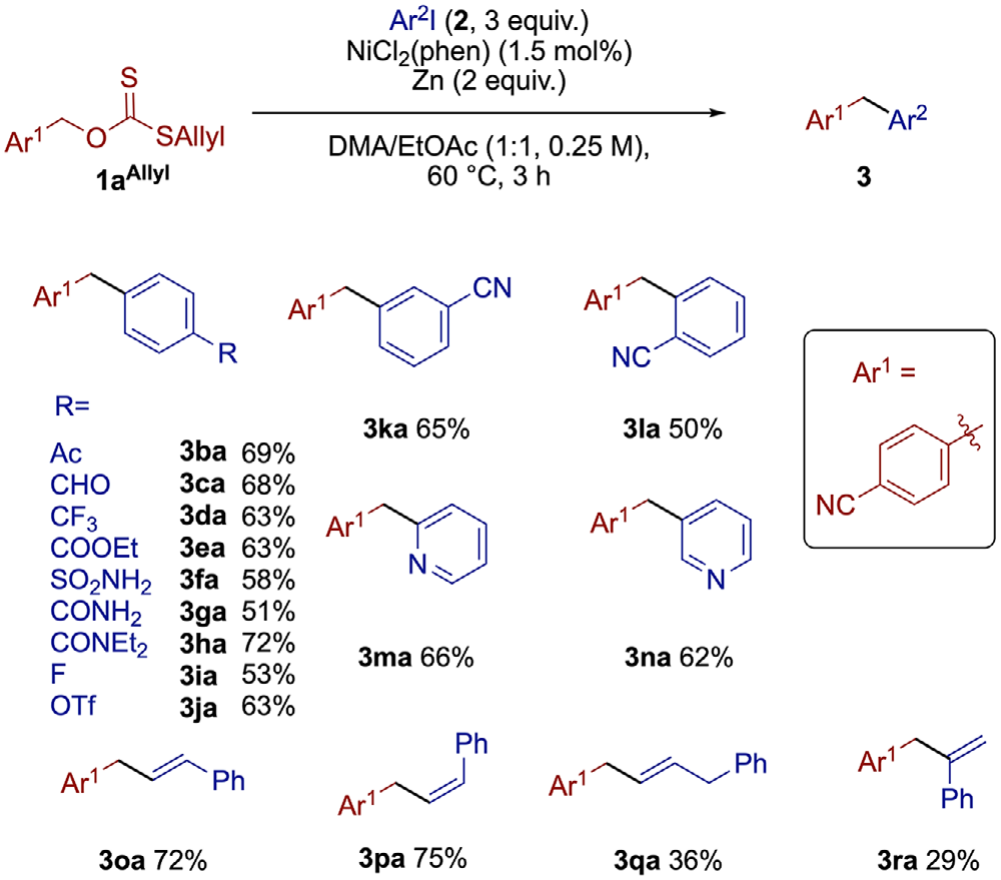
Substrate scope of aryl iodides. Standard conditions: **1a^Allyl^** (1 mmol), **2** (3 equiv.), NiCl_2_(phen) (1.5 mol%), Zn (2 equiv.), 0.5 mL DMA/0.5 mL EtOAc, 60°C, 3 h.

Subsequently, the scope of aryl iodides was investigated (Scheme 3). Generally, only electron‐poor aryl iodides were able to form diarylmethane products. Electron‐neutral aryl iodides led to low yields, and electron‐rich aryl iodides resulted in no product formation. For both electron‐rich and neutral aryl iodides, the decomposition of the xanthate was observed, but no homocoupling product was formed. The diarylmethanes with an acetyl **3ba** and formyl group **3ca** in the para positions could be isolated in a slightly lower yield than the nitrile‐substituted one. Similarly, lower yields of coupling products **3da**–**3ha** were also achieved in the reactions of the corresponding trifluoromethyl‐, ethyl ester‐, sulfonamide‐, and amide‐substituted aryl iodides. A fluorine substituent in **3ia** was also tolerated with a slightly lower yield due to the weaker electron‐withdrawing character. Interestingly, the selective conversion of iodo triflate **2j** was also possible, even though the triflate group can also be active in XECs [17]. If the nitrile substituent was placed in the meta position, the yield of **3ka** was lower, possibly because of the weakened electronic influence of the substituent, while the ortho position resulted in even lower yield of **3la**, probably due to steric constraint.

Next, we found that also alkenyl iodides can be employed in the reaction, as both (*E*)‐ and (*Z*)‐*β*‐iodo styrenes gave the corresponding products **3oa** and **3pa** in good yields under stereo‐retention. The non‐conjugated benzyl vinyl iodide (3qa) and α‐iodo styrene (**3ra**) were synthesized in significantly lower yields, possibly due to the missing conjugation to the benzene moiety.

**SCHEME 4:**
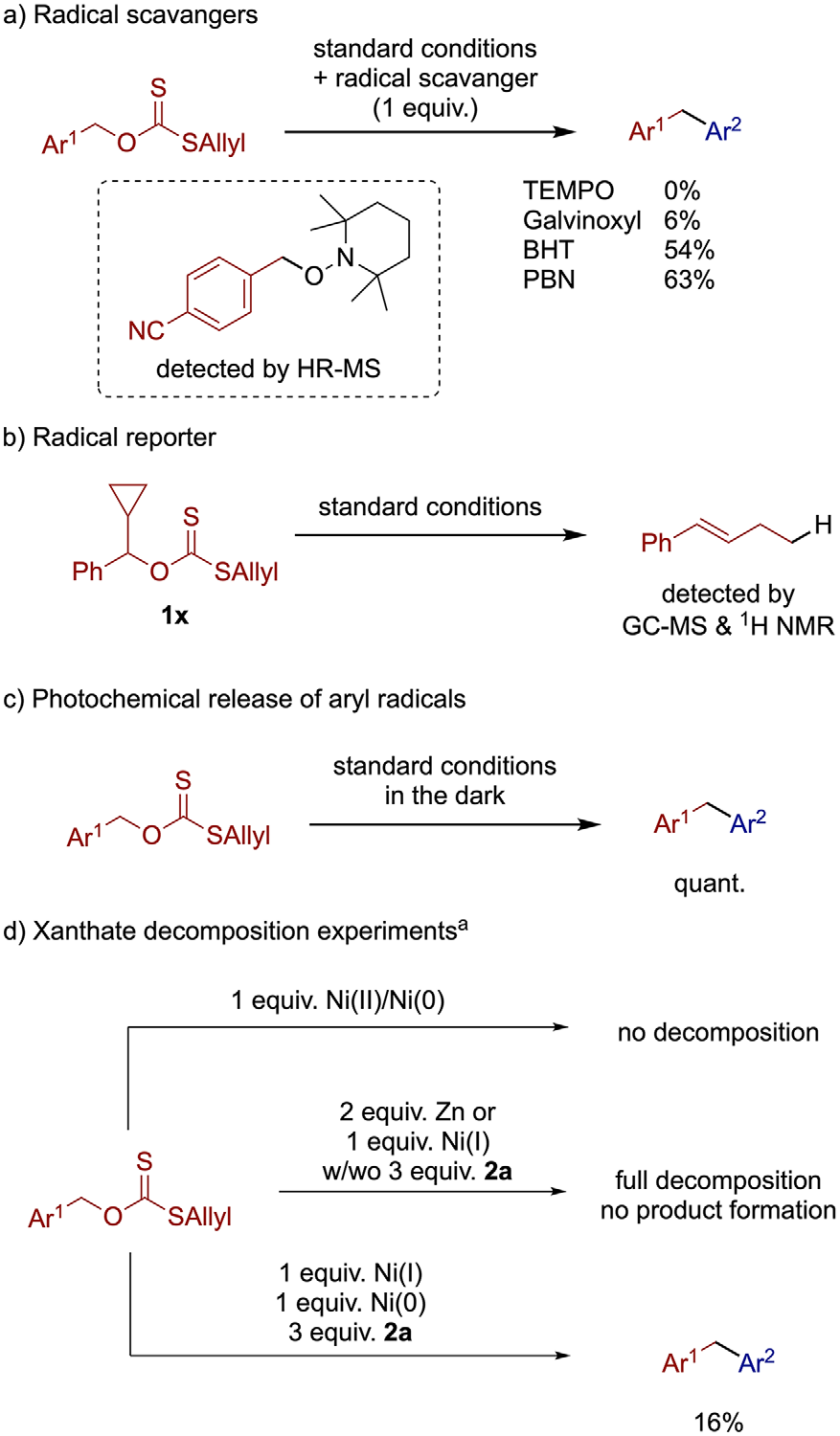
Radical trapping, radical reporter, photochemical decomposition, and xanthate decomposition experiments. Standard conditions: **1a^Allyl^** (0.25 mmol), **2a** (3 equiv.), NiCl_2_(phen) (1.5 mol%), Zn (2 equiv.), 0.5 mL DMA/0.5 mL EtOAc, 60°C, 3 h. Yields determined by GC‐FID with *n*‐pentadecane as an internal standard. ^a^ Ni(II) ≙ NiCl_2_(phen), Ni(0) ≙ 1 equiv. Ni(COD)_2_ + 1 equiv. phen, Ni(I) ≙ 0.5 equiv NiCl_2_(phen) + 0.5 equiv. Ni(COD)_2_ + 0.5 equiv. phen.

To further elucidate the mechanism of the reaction, a series of mechanistic experiments was performed. It was observed that when the reaction was conducted in the presence of several radical scavengers, the coupling was inhibited (Scheme 4a). The reaction was only partially suppressed when 2,6‐di‐*tert*‐butyl‐4‐methylphenol (BHT) and *N*‐*tert*‐butyl‐α‐phenylnitrone (PBN) were used. The inhibition was especially pronounced in the presence of (2,2,6,6‐tetramethylpiperidin‐1‐yl)oxyl (TEMPO) or Galvinoxyl. Moreover, the TEMPO adduct of the benzyl radical released from the xanthate ester was observed via HR‐MS. This provides evidence that the mechanism involves a radical species.

Further experiments were performed with a secondary, α‐cyclopropyl substituted xanthate ester 1x to determine if the typical radical‐induced ring opening of this moiety takes place (Scheme 4b). As expected, no coupling products were observed, but a linear decomposition product of the xanthate ester was detected by GC‐MS and ^1^H‐NMR, further confirming the involvement of radical species. This finding also indicates that the activation of the xanthate ester occurs by SET and not by oxidative addition of the xanthate C─O bond followed by Ni─C bond homolysis [32].

To ensure that the fragmentation of the xanthate ester is not initiated by an aryl radical, generated from the nickel oxidative addition complex by photoexcitation, the reaction was performed in the dark (Scheme 4c) [33]. This experiment displayed the same yield as the reaction performed during daylight; therefore, the activation of the xanthate ester in this fashion can be excluded.

The decomposition of the xanthate ester was investigated in the presence of different nickel species to ascertain if the radical species is generated by Ni(I). Neither 1 equiv. of Ni(0) nor Ni(II) lead to any decomposition of the xanthate ester. In contrast, 10 mol% of Ni(I), either generated by reduction of Ni(II) by zinc or by comproportionation of Ni(II) and Ni(0) led to partial decomposition. A complete decomposition was only observed with 1 equiv. of Ni(I) or by stirring with Zn. Performing the decomposition with 1 equiv. of Ni(I) in the presence of aryl iodide **2a** did still lead to complete decomposition of the xanthate ester, but no product was observed. Only when this reaction was carried out with another 1 equiv. of Ni(0), the product was detected in 16% yield. This indicates that the benzyl radical is generated by either Ni(I) or Zn, and the cross‐coupling of this radical and aryl iodide occurs on Ni(0).

**SCHEME 5:**
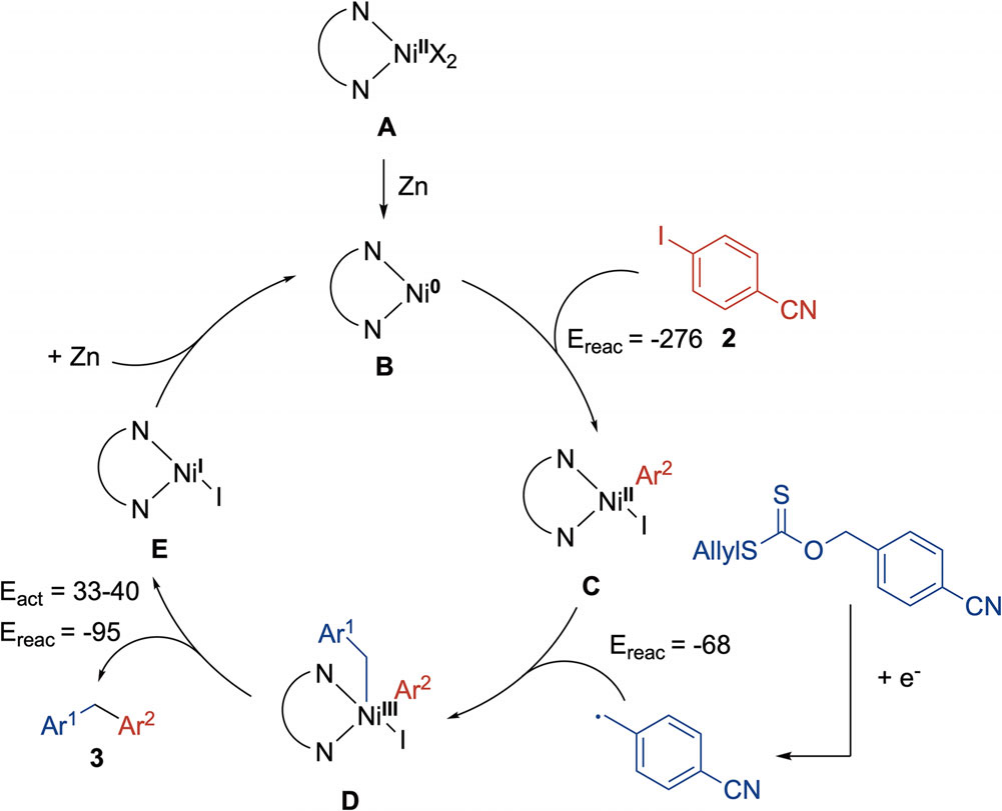
Reaction mechanism of the cross‐coupling reaction between xanthate and para‐iodobenzonitrile over Ni(phen) catalysts as obtained from DFT calculations using the PBE0‐D3 functional. All values are given in kJ/mol.

Based on the experimental results, we performed density functional theory (DFT) calculations of the reaction between the xanthate and para‐iodobenzonitrile in order to shed light on the mechanistic details of the reaction (Scheme 5, for computational details, see Supporting Information). The reaction proceeds via the generation of a Ni(0) species B by reduction of a Ni(II) precatalyst A through metallic Zn. B reacts with para‐iodobenzonitrile via oxidative addition to produce the Ni(II) intermediate C, with a reaction enthalpy of −276 kJ/mol. The xanthate ester 1 can be reduced by one electron (most likely from Zn), which results in the dissociation via splitting of the C─O bond, producing allyl‐carbonodithioic acid and the benzyl radical 2. Note that this process is calculated to be spontaneous without a barrier with a vertical electron affinity of 71 kJ/mol and relaxation energy of −134 kJ/mol (total energy gain of −205 kJ/mol). C reacts with the benzyl radical 1‐rad to produce the Ni(III) intermediate D, which undergoes a reductive elimination through a C─C coupling mechanism to produce the diarylmethane 3 under generation of the Ni(I) species E, which can be reduced by Zn to produce the catalytically active species B. The C─C coupling proceeds via a square‐pyramidal structure where in the apical position is either the benzyl ligand (D) or iodine D’, with the first isomer being slightly more stable (∆*H* = 6 kJ/mol), consistent with similar results reported previously [34, 35, 36]. The barrier for C─C coupling is calculated to be 33 and 40 kJ/mol with respect to D when using D′ and D as the starting point, respectively, with the reaction being downhill by −95 kJ/mol.

At the same time, a mechanism involving the in situ generation of arylzinc salt from aryl iodide cannot be completely excluded. Stirring just zinc and the aryl iodide together under the standard reaction conditions, followed by an acidic quench, resulted in the formation of about 15% of the hydrodehalogenation product. Formation of arylzinc salts in DMF at a slightly elevated temperature is known in the literature [37]. However, the reaction also proceeded with an organic reductant wherein no organozinc species can be formed (Table 1, entry 13). Additionally, the reaction can be conducted using protic cosolvents (*^i^* PrOH, *^t^* BuOH) that should quench the aryl zinc species right after formation.

## 3 Conclusion

In summary, we developed a nickel‐catalyzed reductive cross‐coupling of O‐benzylic and allylic xanthate esters with aryl and alkenyl iodides. The catalytic system operates with a comparatively low amount of catalyst to convert a multitude of differently functionalized substrates and also pharmaceutical compounds. Furthermore, chemoselective couplings in the presence of chlorides, bromides, more electron‐rich iodides, and triflates were achieved. Strong influence on the yield of the reaction has the electronic properties of the aryl iodides. Mechanistic investigations provided evidence that radicals are involved in the process and that the proposed SET potentially occurs from metallic Zn, while the coupling of the radical with the aryl iodide takes place on a Ni(0) species.

## Conflicts of Interest

The author declare no conflicts of interest.

## Supporting information




**Supporting File**: The authors have cited additional references within the Supporting Information [38, 39, 40, 41, 42, 43, 44, 45, 46, 47, 48, 49, 50, 51, 52, 53, 54, 55, 56, 57, 58, 59, 60, 61, 62, 63, 64, 65, 66, 67, 68, 69, 70, 71, 72, 73, 74, 75, 76].

## Data Availability

The data that support the findings of this study are available from the corresponding author upon reasonable request.
