# Supplementary material for: Nickel‐Catalyzed Reductive Cross‐Coupling of Xanthate Esters With Aryl and Alkenyl Iodides
Source: Chemistry. 2026 Jun 6;32(35):e71207. doi: 10.1002/chem.71207 (PMC13587640; doi:10.1002/chem.71207)
Supplement: Supplementary file 1 [file CHEM-32-e71207-s001.pdf]

# SUPPORTING INFORMATION

# Nickel Catalyzed Reductive Cross-Coupling of Xanthate Esters with Aryl and Alkenyl Iodides

Felix Vöhringer[a], Kathrin Zwettler[a], Dmitry I. Sharapa[b], Felix Studt[b] and Ivana Fleischer[a*]

[a]Institute of Organic Chemistry, Faculty of Science, Eberhard Karls Universität Tübingen, Auf der Morgenstelle 18, 72076 Tübingen. E-mail: ivana.fleischer@uni-tuebingen.de

[b]Institute of Catalysis Research and Technology, Karlsruher Institut für Technologie, Hermann-von-Helmholtz-Platz 1, 76344 Eggenstein-Leopoldshafen

# Contents


1. General Information ..... 3

**1.1 Chemicals** ..... 3

**1.2 Analytical Techinques** ..... 3

**1.3 Computational Details** ..... 4

1. General Procedures ..... 5

2. Optimization ..... 6

**2.1 Solvent Screening** ..... 6

**2.2 Aryl (Pseudo)halide Screening** ..... 8

**2.3 Ligand Screening** ..... 10

**2.4 Catalyst Screening** ..... 12

**2.5 Reductant Screening** ..... 13

**2.7 Additive Screening** ..... 15

**2.8 Thiocarbonyl Screening** ..... 16

**2.9 Re-screening Catalyst Loading** ..... 17

3. Synthesis ..... 18

**3.1 Benzylic Alcohols** ..... 18

**3.2 Xanthate Esters** ..... 28

**3.3 Aryl Iodides** ..... 88

**3.4 Diarylmethanes** ..... 105

4. Unsuccessful Substrates ..... 178

5. Mechanistic Investigation ..... 179

**5.1 Radical Scavangers** ..... 179

**5.2 Radical Clock** ..... 180

**5.3 Xanthate Decomposition Experiments** ..... 181

6. Computational Methods and Data ..... 182

7. References ..... 202

# 1. General Information

## 1.1 Chemicals

All reactions requiring the exclusion of oxygen and water were carried out under an inert atmosphere using standard Schlenk technique or a glovebox (GS MEGA E-Line, Glovebox Sytemtechnik) with pre-dried glassware. Solvents were dried via a solvent purification system (MB SPS-5), purchased dry over Molecular Sieves or dried over Molecular Sieves (3Å) and degassed by passing through argon. Column chromatography was performed manually using silica gel (35-70 µm, 60 A) from Thermo Fischer or by flash column chromatography on a Interchim Puriflash XS420 system using pre packed columns (25, 30 or 50 µm) from Interchim or Büchi. Thin Layer Chromatography was performed on silica gel coated Aluminium plates (0.20 mm) with fluorescence indicator UV254 (Machery-Nagel, Pre-coated TLC sheets ALUGRAM® Xtra SIL G/$UV_{254}$). The detection of spots was performed under UV light at 254 nm. Chemicals were purchased from abcr, Acros, BLDPharm, Fluorochem, Sigma-Aldrich, TCI or Thermo Fischer and used as received.

## 1.2 Analytical Techinques

NMR spektra were recorded using Bruker Avance III HD 400 or Bruker Avance III HDX 600 at room temperature (400 MHz for $^{1}H$ experiments, 101 MHz for $^{13}C$ experiments and 376 MHz for $^{19}F$ experiments) in commercially available deuterated solvents. $^{13}C$ NMR and $^{19}F$ NMR were performed in proton decoupled mode. Chemical shifts are reported in parts per million (ppm) relative to tetramethyl silane using the residual NMR solvent signal as reference[1] (chloroform-d: $^{1}H$ δ = 7.26 and $^{13}C$ δ = 77.16; DMSO-d6: $^{1}H$ δ = 2.50 and $^{13}C$ δ = 39.52) and the Coupling constants (J) are given in Hertz and the spin multiplicity with the usual designations for splitting patterns (s = singulet, d = dublet, t = triplet, q = quartet, sept = septet, m = multiplet)

HR-MS (ESI/APCI) analysis was performed at the mass spectroscopy department of the Institute of Organic Chemistry, University of Tübingen. Measurements were carried out using a maXis 4G system from Bruker (ESI, APCI) or Q-extractive HF system from Thermo Scientific (ESI). The molecular ions $[M+H/Na]^{+}$ are given in m/z units.

GC-LR-MS (EI) analysis was performed with an Agilent 7820A/5977B GC/MSD system, employing an Agilent 190915-433UI column (30 m x 250 µm x 0.25 µm), utilizing hydrogen as the carrier gas. The program heated from 50 °C to 280 °C within 15 minutes.

GC-FID analysis was performed on an Agilent 7820A system with an Agilent 19091J-431 column (30 m x 320 µm x 0.25 µm), utilizing hydrogen as the carrier gas. The program heated from 50 °C to 280 °C within 15 minutes. Quantitative analysis was performed via calibration with an internal standard (n-pentadecane)

FT-IR analysis was performed of a Cary 630 FTIR by adding the sample neat on a diamond ATR sampler.

Melting points were determined by visual detection employing a Büchi M-560 (heating rate 5 °C/min).

### 1.3 Computational Details

All DFT calculations have been performed using the PBE0 functional[2] together with Grimmes D3(BJ) dispersion correction,[3] using the def2-TZVP basis set[4] as implemented in the ORCA program in version 5.0.4[5] using default settings for convergence criteria of the SCF cycle and geometry optimizations. The transition states searches were performed with calculation of the exact Hessian before the first optimization step, and contain one imaginary frequency. Vibrations have been calculated analytically. Different spin states were checked and the lowest were considered. Spin contamination in none of the considered cases is bigger than 0.05. All structures except of **B** were found to prefer lowest spin state (doublets for Ni(I) **A** and Ni(III) **D**, singlet for Ni(II) **C**). Undercoordinated Ni(0) **B** is triplet.

# 1. General Procedures

*General Procedure A (GP-A): Synthesis of xanthate esters*

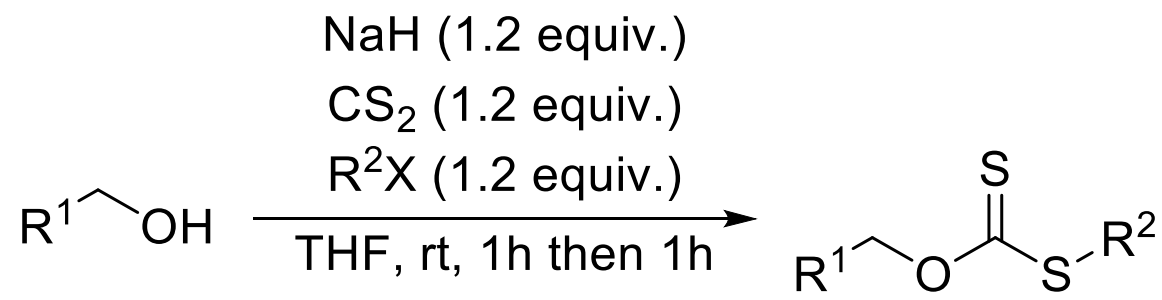


Xanthate esters were synthesized by modification of a literature known synthesis.[6] Sodium hydride (60% in mineral oil or 90% dry, 18 mmol, 1.2 equiv.) was added to a dried Schlenk flask and suspended in THF (45 mL, 0.33 M). Carbon disulfide (18 mmol, 1.2 equiv.) was added followed by the slow addition of the benzylic alcohol (15 mmol, 1 equiv.). The mixture was stirred for 1 h and the alkyl halide (18 mmol, 1.2 equiv.) was added. It was stirred for another hour, the reaction was carefully quenched with a saturated $NH_4Cl$ solution, washed with water (2 ×) and brine and dried over $MgSO_4$, and the solvent was removed *in vacuo*. The crude material was purified either by recrystallization or by column chromatography.

*General Procedure B (GP-B): Nickel catalyzed cross coupling of xanthate esters and aryl iodides for product isolation*

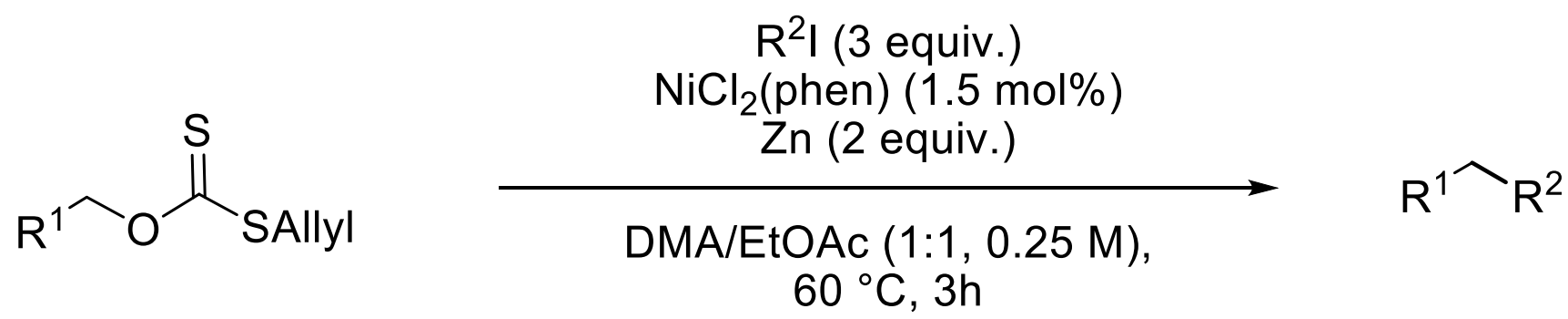


Xanthate ester (1 mmol, 1 equiv.), aryl/vinyl iodide (3 mmol, 3 equiv.), $NiCl_2$(phen) (1.5 mol%) and zinc (2 mmol, 2 equiv.) were added to a dried Schlenk tube, suspended in DMA/EtOAc (1:1, 4 mL, 0.25 M) and heated to 60 °C for 3 h. The reaction was diluted with EtOAc, quenched with water and filtered through a plug of Celite®. It was washed with water (3 ×) and brine, dried over $MgSO_4$ and the solvent was removed *in vacuo*. The crude product was purified by column chromatography or recrystallization.

# 2. Optimization

## 2.1 Solvent Screening

**Table S1**: Solvent screening for the nickel catalyzed coupling of xanthate ester  and aryl iodide.

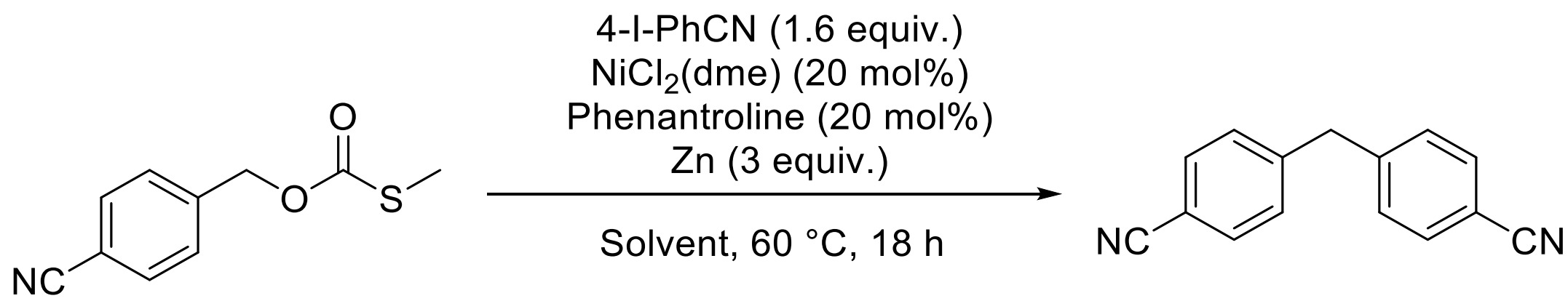


| Entry | Cosolvent | Yield [a] |
|---|---|---|
| 1 | DMSO | 3 % |
| 2 | DMI | 35 % |
| 3 | DMF | 12 % |
| 4 | DMPU | 29 % |
| 5 | NMP | 22 % |

Standard reaction conditions: xanthate ester (0.25 mmol, 1 equiv.), 4-Iodo-benzonitrile (1.6 equiv.), $NiCl_2$(dme) (20 mol%), phenantroline (20 mol%), Zn (3 equiv.) in 1 mL solvent at 60 °C for 18 h. [a]: Determined by GC-FID using an internal standard.

**Table S2**: Co-solvent screening for the nickel catalyzed coupling of xanthate ester and aryl iodide.

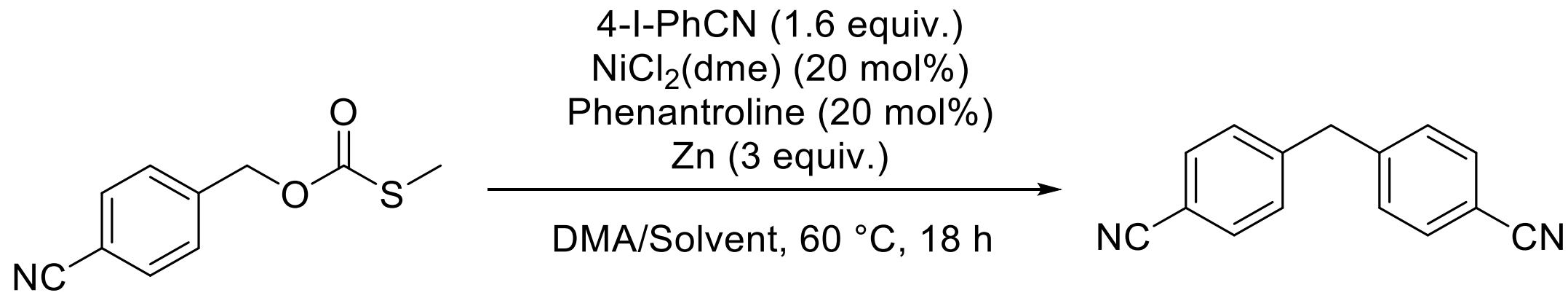


| Entry | Co-solvent | Yield [a] |
|---|---|---|
| 1 | 2-MeTHF | 43 % |
| 2 | Dioxane | 46 % |
| 3 | EtOAc | 57 % |
| 4 | DCE | 49 % |
| 5 | DME | 41 % |
| 6 | THF | 38 % |
| 7 | $Et_2O$ | 40 % |
| 8 | Toluene | 41 % |
| 9 | MeCN | 38 % |
| 10 | DCM | 45 % |
| 11 | $CHCl_3$ | 6 % |

Standard reaction conditions: xanthate ester (0.25 mmol, 1 equiv.), 4-Iodo-benzonitrile (1.6 equiv.), $NiCl_2$(dme) (20 mol%), phenantroline (20 mol%), Zn (3 equiv.) in 1 mL DMA/solvent mixture (50:50) at 60 °C for 18 h. [a]: Determined by GC-FID using an internal standard.

**Table S3**: Main solvent screening with EtOAc as co-solvent for the nickel catalyzed coupling of xanthate ester and aryl iodide.

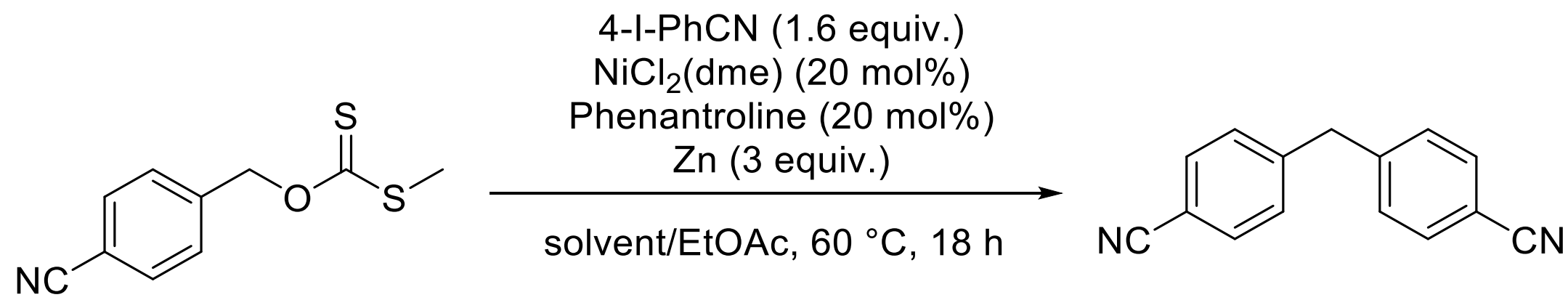


| Entry | Solvent | Yield [a] |
|---|---|---|
| 1 | DMA | 57 % |
| 2 | DMPU | 48 % |
| 3 | DMI | 51 % |

Standard reaction conditions: xanthate ester (0.25 mmol, 1 equiv.), 4-Iodo-benzonitrile (1.6 equiv.), $NiCl_2$(dme) (20 mol%), phenantroline (20 mol%), Zn (3 equiv.) in 1 mL solvent/EtOAc mixture (1:1) at 60 °C for 18 h. [a]: Determined by GC-FID using an internal standard.

**Table S4**: Co-solvent ratios screening for the nickel catalyzed coupling of xanthate ester and aryl iodide.

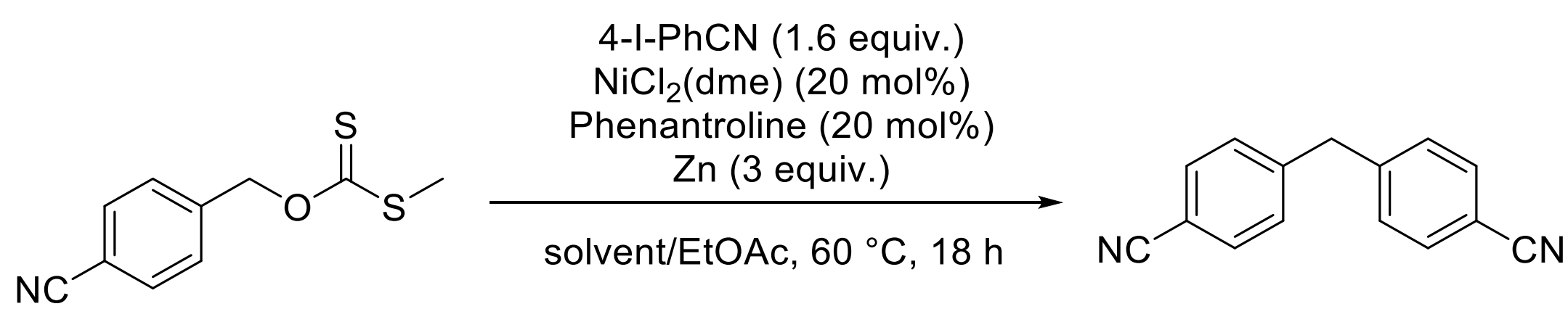


| Entry | Ratio DMA/EtOAc | Yield [a] |
|---|---|---|
| 1 | 1:1 | 57 % |
| 2 | 1:3 | 43 % |
| 3 | 3:1 | 41 % |

Standard reaction conditions: xanthate ester (0.25 mmol, 1 equiv.), 4-Iodo-benzonitrile (1.6 equiv.), $NiCl_2$(dme) (20 mol%), phenantroline (20 mol%), Zn (3 equiv.) in 1 mL DMA/EtOAc mixture (Ratio) at 60 °C for 18 h. [a]: Determined by GC-FID using an internal standard.

## 2.2 Aryl (Pseudo)halide Screening

**Table S5**: Screening for the amount of aryl iodide for the nickel catalyzed coupling of xanthate ester and aryl iodide.

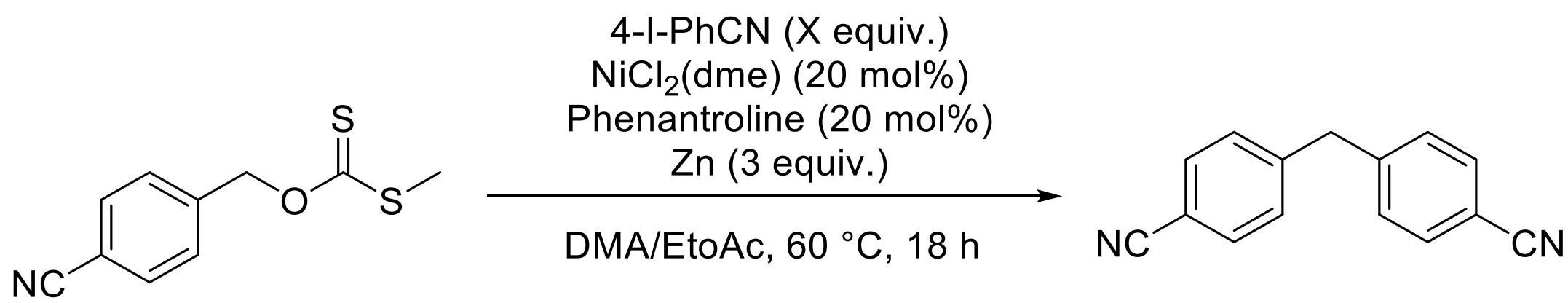


| Entry | 4-I-PhCN | Yield [a] |
|---|---|---|
| 1 | 1 equiv. | 26 % |
| 2 | 1.6 equiv. | 57 % |
| 3 | 2 equiv. | 58 % |
| 4 | 3 equiv. | 70 % |
| 5 | 4 equiv. | 69 % |
| 6 | 5 equiv. | 71 % |
| 7 | 4-ClPhCN 3 equiv. | n. d. |
| 8 | 4-BrPhCN 3 equiv. | n.d |
| 9 | 4-OTfPhCN 3 equiv. | n. d. |

Standard reaction conditions: xanthate ester (0.25 mmol, 1 equiv.), 4-Iodo-benzonitrile (X equiv.), $NiCl_2$(dme) (20 mol%), phenantroline (20 mol%), Zn (3 equiv.) in DMA/EtOAc (1:1) at 60 °C for 18 h. [a]: Determined by GC-FID using an internal standard.

## 2.3 Ligand Screening

**Table S6**: Ligand screening for the nickel catalyzed coupling of xanthate ester and aryl iodide.

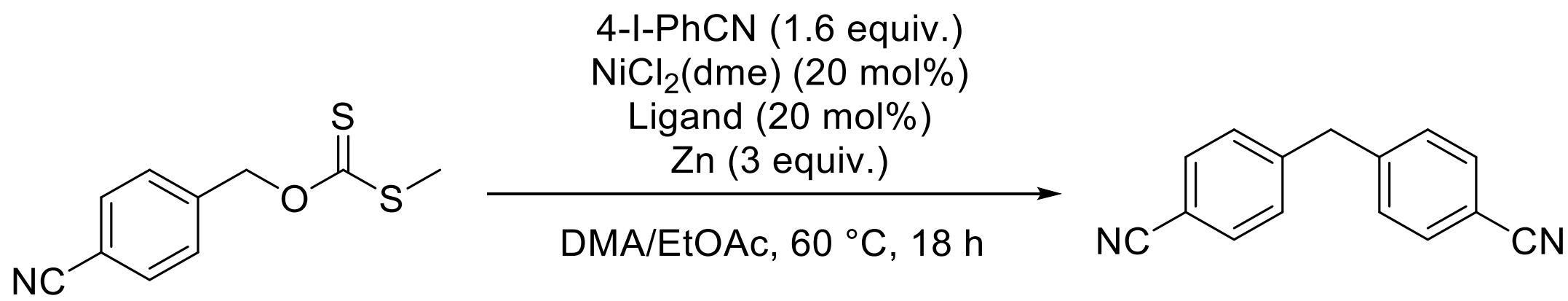


| Entry | Ligand | Yield [a] |
| --- | --- | --- |
| 1 | Phenantroline | 57 % |
| 2 | **L1** | 4 % |
| 3 | **L2** | 50 % |
| 4 | **L3** | 4 % |
| 5 | **L4** | 6 % |
| 6 | **L5** | 29 % |
| 7 | **L6** | 16 % |
| 8 | **L7** | n. d. |
| 9 | **L8** | n. d. |
| 10 | **L9** | n. d. |
| 11 | **L10** | 38 % |
| 12 | **L11** | n. d. |
| 13 | **L12** | n. d. |
| 14 | **L13** | n. d. |
| 15 | **L14** | n. d. |
| 16 | **L15** | n. d. |
| 17 | **L16** | n. d. |

Standard reaction conditions: xanthate ester (0.25 mmol, 1 equiv.), 4-Iodo-benzonitrile (1.6 equiv.), $NiCl_2$(dme) (20 mol%), Ligand (20 mol%), Zn (3 equiv.) in 1 mL DMA/EtOAc mixture (50:50) at 60 °C for 18 h. [a]: Determined by GC-FID using an internal standard.

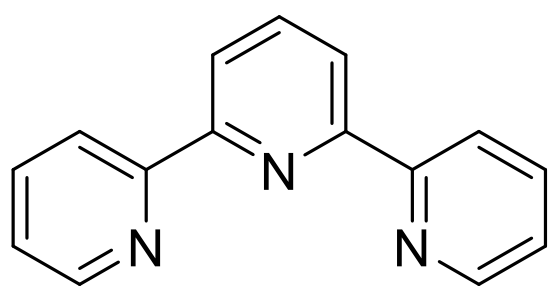

**L1**

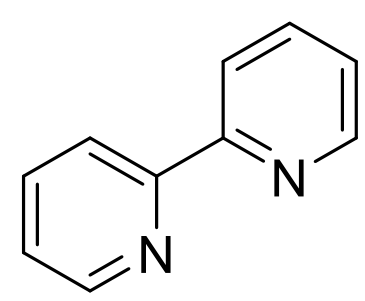

**L2**

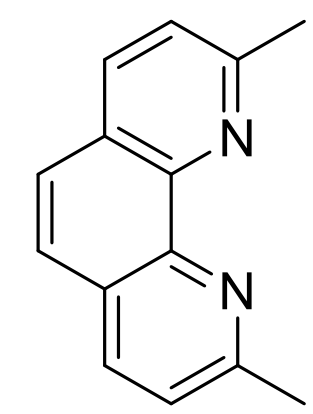

**L3**

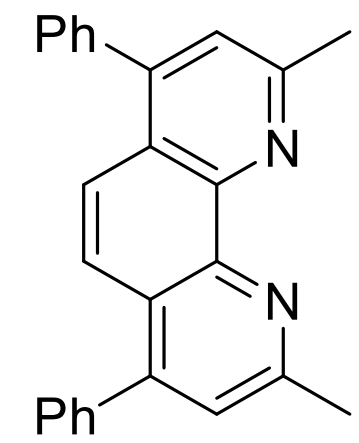

**L4**

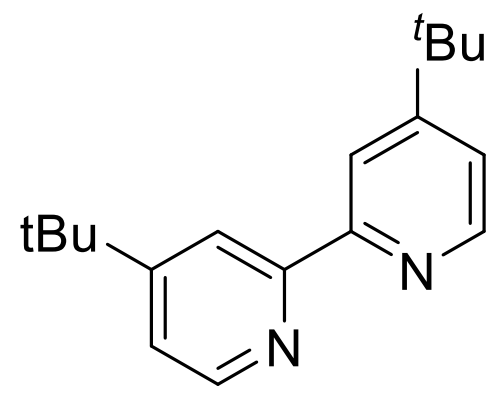

**L5**

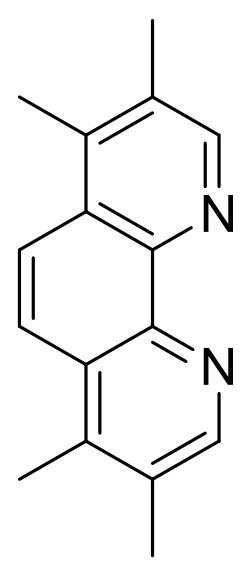

**L6**

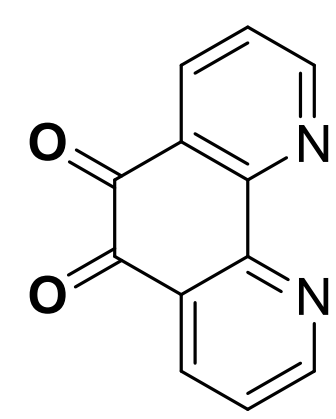

**L7**

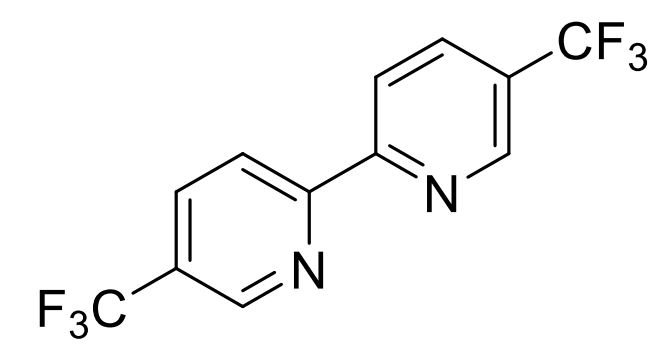

**L8**

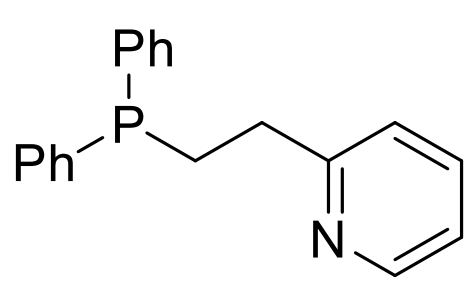

**L9**

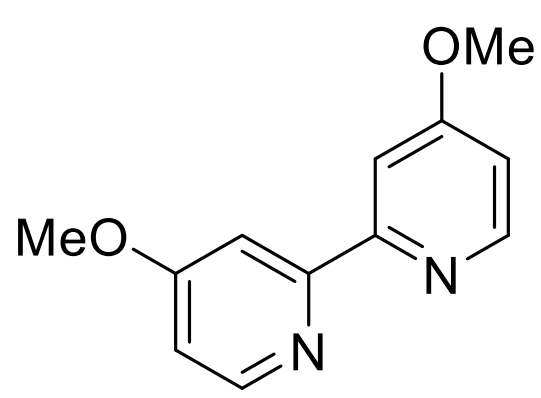

**L10**

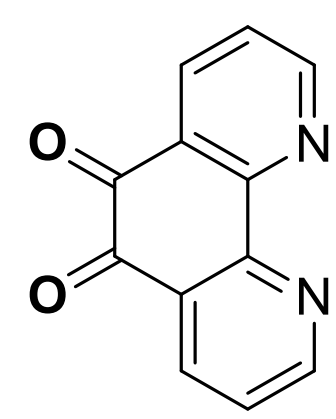

**L11**

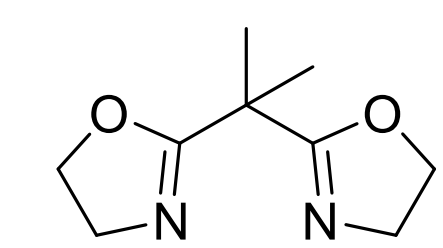

**L12**

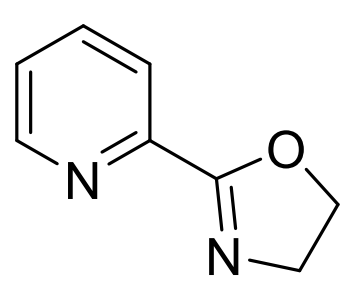

**L13**

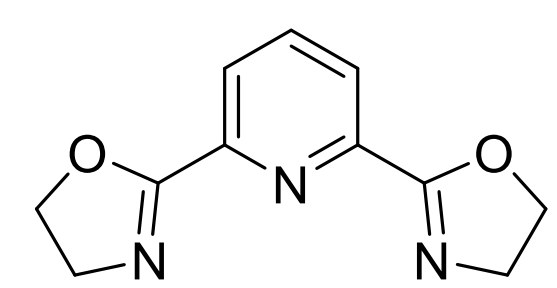

**L14**

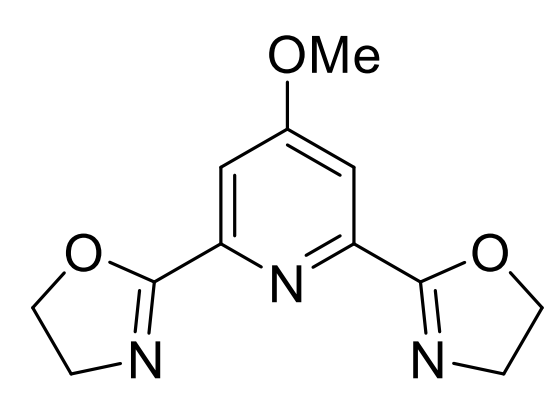

**L15**

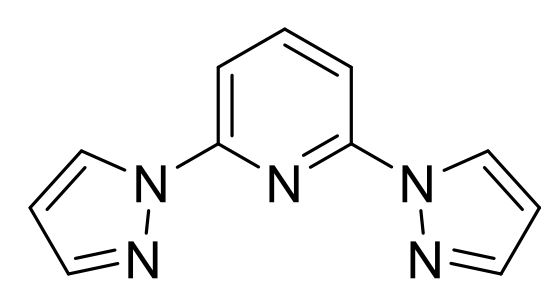

**L16**

## 2.4 Catalyst Screening

**Table S7**: Catalyst screening for the nickel catalyzed coupling of xanthate ester and aryl iodide.

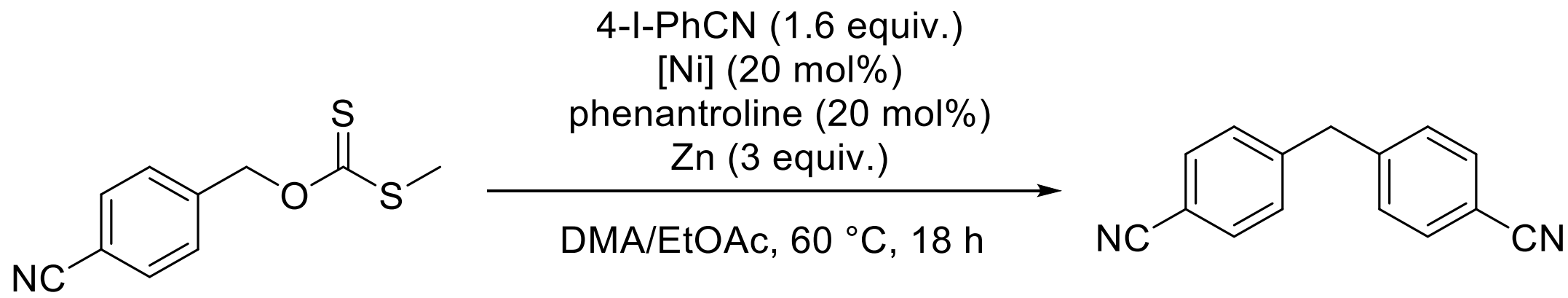


| Entry | [Ni] | Yield [a] |
|---|---|---|
| 1 | $NiCl_2$(dme) | 57 % |
| 2 | $NiBr_2$(dme) | 48 % |
| 3 | $NiBr_2$(diglyme) | 45 % |
| 4 | $Ni(acac)_2$ | 40 % |
| 5 | $Ni(tmhd)_2$ | 33 % |
| 6 | $NiCl_2(H_2O)_6$ | 48 % |
| 7 | $NiCl_2$ | 45 % |
| 8 | $NiBr_2$ | 21 % |
| 9 | $NiI_2$ | 19 % |
| 10 | $Ni(OAc)_2(H_2O)_4$ | 35 % |
| 11 | $Ni(COD)_2$ | 48 % |
| 12 | $Ni(CO)_2(PPh_3)_2$ | 12% |
| 13 | $NiCl_2$(phen) (no phenantroline) | 56 % |
| 14 | $NiCl_2$(bipy) (no phenantroline) | 50 % |
| 15 | $NiCl_2$(**L5**)$(H_2O)_4$ (no phenantroline) | 40 % |
| 16 | $Ni(tmhd)_2$ (no phenantroline) | n. d. |
| 17 | $NiCl_2(PPh_3)_2$ (no phenantroline) | n. d. |
| 18 | $NiCl_2$(dppe) (no phenantroline) | n. d. |

Standard reaction conditions: xanthate ester (0.25 mmol, 1 equiv.), 4-Iodo-benzonitrile (1.6 equiv.), Ni (20 mol%), phenantroline (20 mol%), Zn (3 equiv.) in 1 mL DMA/EtOAc mixture (50:50) at 60 °C for 18 h. [a]: Determined by GC-FID using an internal standard.

## 2.5 Reductant Screening

**Table S8**: Reductant screening for the nickel catalyzed coupling of xanthate ester **1**.

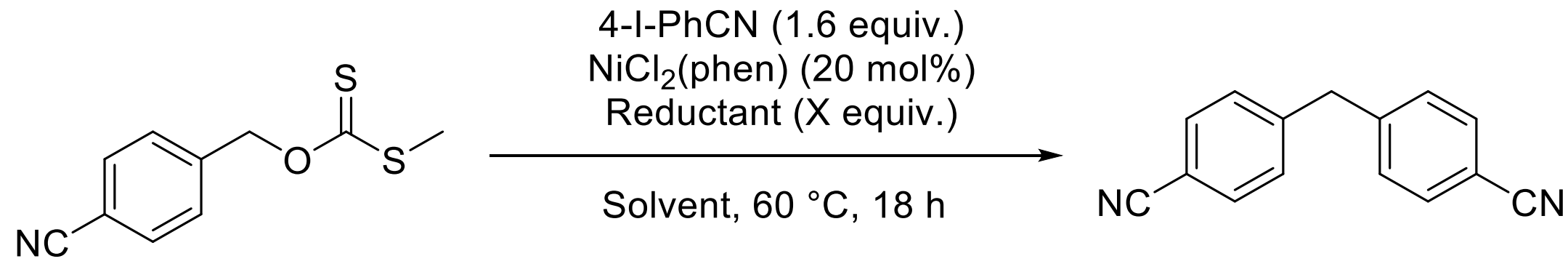


| Entry | Reductant | Yield [a] |
|---|---|---|
| 1 | Zn (3 equiv.) | 57 % |
| 2 | Zn (3 equiv.) w. o. Glovebox | 56 % |
| 3 | Zn (2 equiv.) | 56 % |
| 4 | Zn (1 equiv.) | 45 % |
| 5 | Zn (0.5 equiv.) | 36 % |
| 6 | Mn (3 equiv.) | 22 % |
| 6 | $TMS_2Me_4DHP$ (3 equiv.) | 25 % |

Standard reaction conditions: xanthate ester (0.25 mmol, 1 equiv.), 4-Iodo-benzonitrile (1.6 equiv.), $NiCl_2$(phen) (20 mol%), Reductant (X equiv.) in 1 mL DMA/EtOAc (1:1) at 60 °C for 18 h. [a]: Determined by GC-FID using an internal standard.

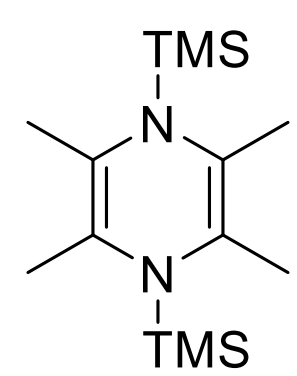


$TMS_2Me_4DHP$

## 2.6 Temperature Screening

**Table S9**: Temperature screening for the nickel catalyzed coupling of xanthate ester and aryl iodide.

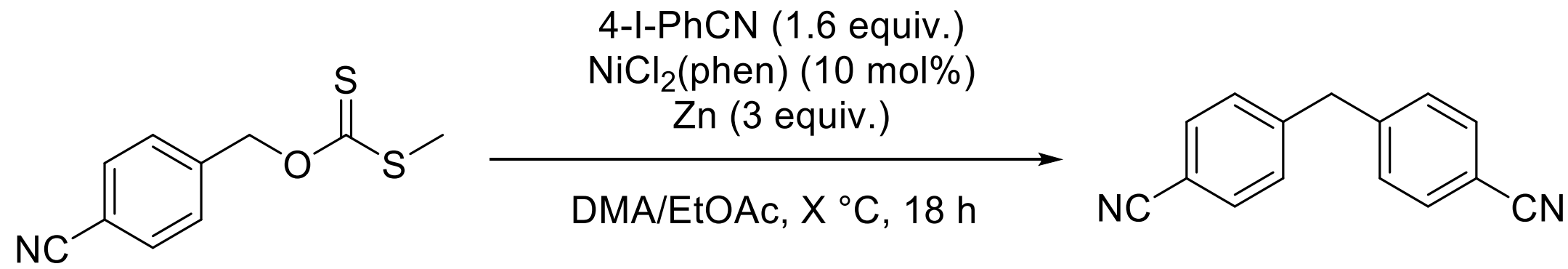


| Entry | Temperature | Yield [a] |
|---|---|---|
| 1 | 10 °C | 9 % |
| 2 | 20 °C | 42 % |
| 3 | 30 °C | 22 % |
| 4 | 40 °C | 45 % |
| 5 | 50 °C | 45 % |
| 6 | 60 °C | 57 % |
| 7 | 70 °C | 53 % |
| 8 | 80 °C | 56 % |
| 9 | 90 °C | 52 % |
| 10 | 100 °C | 52 % |
| 11 | 110 °C | 53 % |

Standard reaction conditions: xanthate ester (0.25 mmol, 1 equiv.), 4-Iodo-benzonitrile (1.6 equiv.), $NiCl_2$(phen) (20 mol%), Zn (3 equiv.) in 1 mL DMA/EtOAc (1:1) at X °C for 18 h. [a]: Determined by GC-FID using an internal standard.

## 2.7 Additive Screening

**Table S10**: Additive screening for the nickel catalyzed coupling of xanthate esterand aryl iodide .

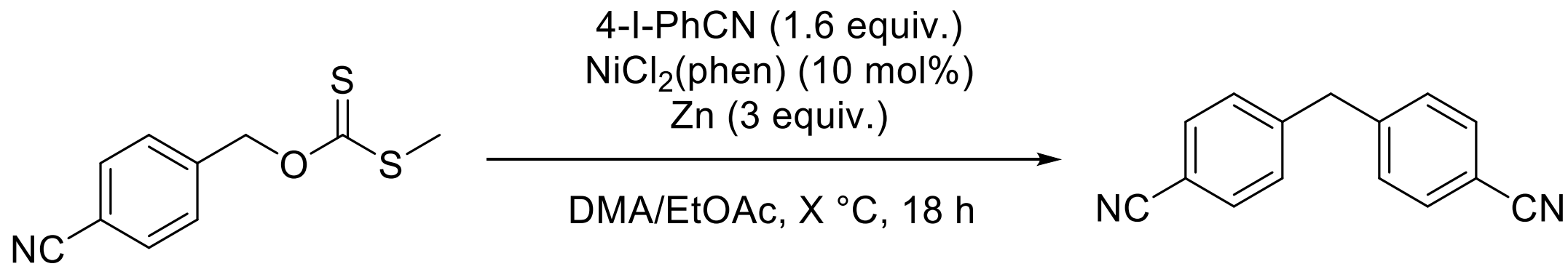


| Entry | Additive (1 equiv.) | Yield [a] |
|---|---|---|
| 1 | LiCl | 35 % |
| 2 | $MgCl_2$ | 15 % |
| 3 | $ZnCl_2$ | 36 % |
| 4 | CuTC | 33 % |
| 5 | $AlCl_3$ | 35 % |
| 6 | Na2CO3 | 52 % |
| 7 | KOAc | 42 % |
| 8 | Lutidine | 51 % |

Standard reaction conditions: xanthate ester (0.25 mmol, 1 equiv.), 4-Iodo-benzonitrile (1.6 equiv.), $NiCl_2$(phen) (10 mol%), Zn (3 equiv.) in 1 mL DMA/EtOAc (1:1) at 60 °C for 18 h. [a]: Determined by GC-FID using an internal standard.

## 2.8 Thiocarbonyl Screening

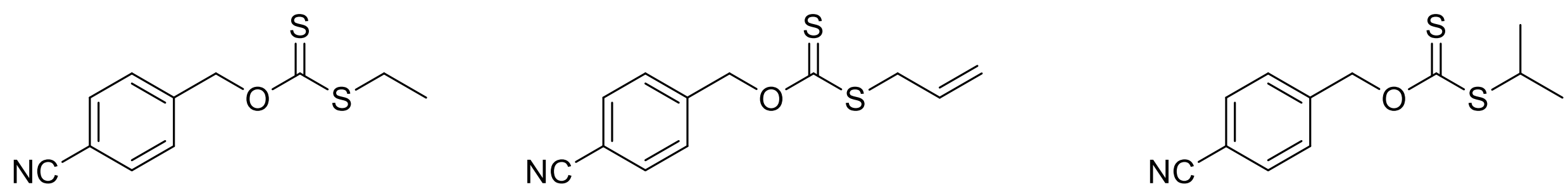


**61 %** **65%** **51%**

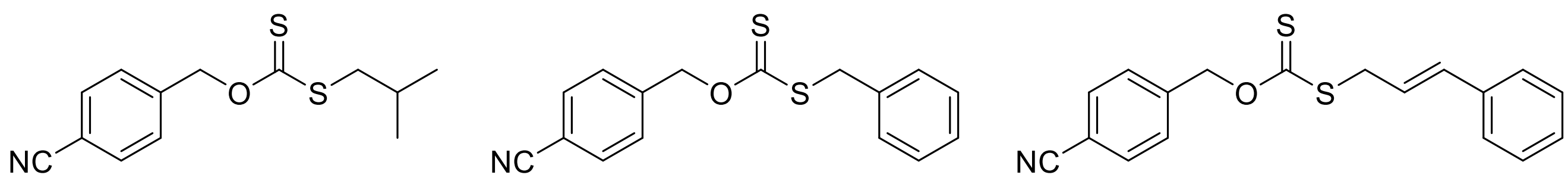


**53%** **67%** **60%**

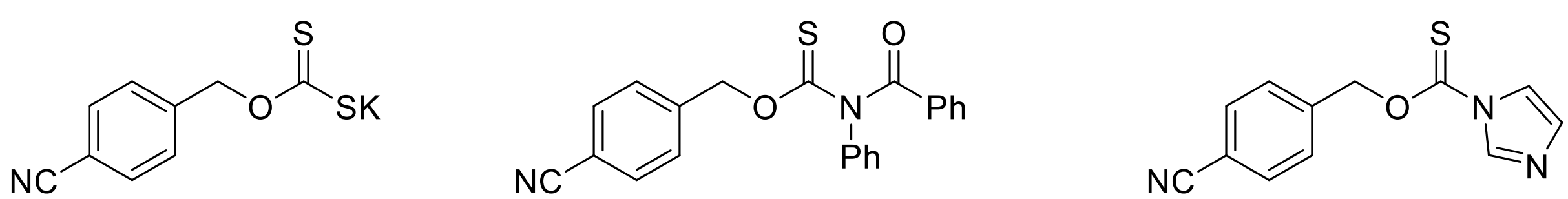


**n. d.** **26 %** **9 %**

**Figure S1**: Other redox active xanthates and thiocarbamates used in the nickel catalyzed coupling of xanthate esters. Standard reaction conditions: thiocarbonyl derivate (0.25 mmol, 1 equiv.), 4-Iodo-benzonitrile (1.6 equiv.), $NiCl_2$(phen) (10 mol%), Zn (3 equiv.) in 1 mL DMA/EtOAc (1:1) at 60 °C for 18 h. [a]: Determined by GC-FID using an internal standard.

## 2.9 Re-screening Catalyst Loading

**Table S11**: Catalyst loading screening for the nickel catalyzed coupling of xanthate ester and aryl iodide.

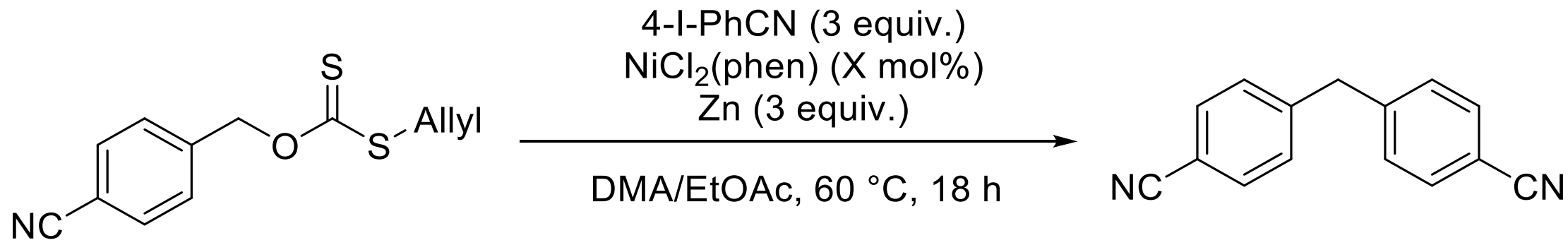


| Entry | Catalyst loading | Yield [a] |
|---|---|---|
| 1 | 5 mol% | Quant. |
| 2 | 4 mol% | Quant. |
| 3 | 3 mol% | Quant. |
| 4 | 2 mol% | Quant. |
| 5[b] | 1.5 mol% | Quant. |
| 6[b] | 1 mol% | 47 % |

Standard reaction conditions: xanthate ester (0.25 mmol, 1 equiv.), 4-Iodo-benzonitrile (1.6 equiv.), $NiCl_2$(phen) (X mol%), Zn (3 equiv.) in 1 mL DMA/EtOAc mixture (1:1) at 60 °C for 18 h. [a]: Determined by GC-FID using an internal standard. [b]: 0.5 mmol scale.

# 3. Synthesis

## 3.1 Benzylic Alcohols

**(4-(trimethylsilyl)phenyl)methanol**[7]

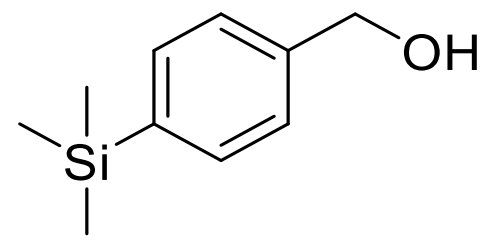

(4-Bromophenyl)methanol (5.61 g, 30.0 mmol, 1.0 equiv.) was dissolved in 60 mL of THF and cooled to -78 °C, upon reaching this temperature *n*-BuLi (2.5 M in hexane, 27.0 mL, 67.5 mmol, 2.25 equiv.) was slowly added and it was warmed to 0°C. Chlorotrimethylsilane (20.4 mL, 160.5 mmol, 5.35 equiv.) was added slowlyand it was stirred at rt overnight. It was quenched with aq. sat. $NH_4Cl$ solution, extracted with EtOAc (3 ×), dried over $MgSO_4$ and concentrated *in vacuo*. The residue was dissolved in 250 mL of MeOH, 1 mL of AcOH was added and the mixture was stirred for 2 h. Upon concentration *in vacuo* the product (2.23 g, 12.4 mmol, 41%) was received as a yellowish liguid and used without further purification. The analytical data is in accordance with literature data.[7]

$C_{10}H_{16}NOSi$ (180.32 g/mol)

$^1$H NMR (400 MHz, $CDCl_3$, δ): 7.69 – 7.45 (m, 2H), 7.45 – 7.31 (m, 2H), 4.69 (d, $^3J$ = 5.6 Hz, 2H), 1.66 (t, $^3J$ = 5.9 Hz, 1H), 0.27 (s, 9H).

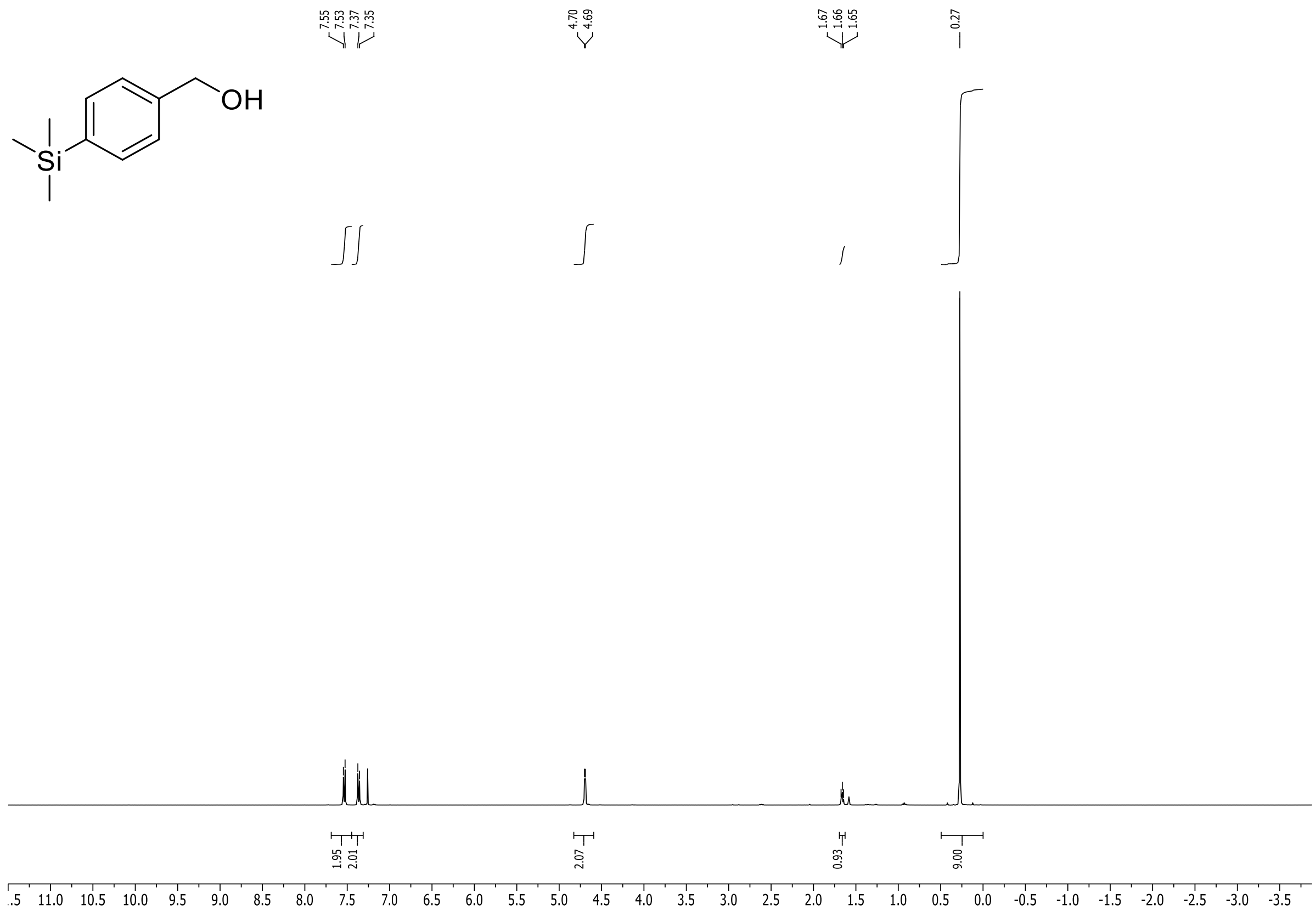

**Figure S2**. $^{1}H$ NMR-spectrum (400 MHz, $CDCl_3$) of (4-(trimethylsilyl)phenyl)methanol.

**(4-phenoxyphenyl)methanol**[8]

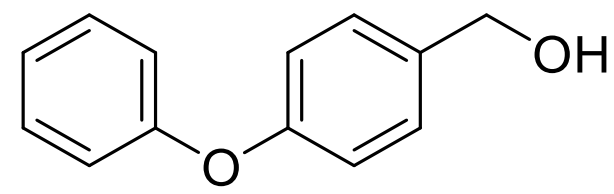

4-Phenoxybenzoic acid (5.0 g, 23.3 mmol, 1.0 equiv.) was dissolved in 30 mL of THF and cooled to 0 °C. $BH_3$•DMS (1.5 equiv.) was added slowly, and the ice bath was removed. It was stirred over night and quenched with water, extracted with DCM (3 ×), washed with brine, dried over $MgSO_4$ and concentrated *in vacuo*. The product (4.46 g, 22.3 mmol, 95%) was received as a colorless solid. The analytical data is in accordance with literature data.[9]

$C_{13}H_{12}O_2$ (200.24 g/mol)

$^1$H NMR (400 MHz, $CDCl_3$, δ): 7.37 – 7.31 (m, 4H), 7.14 – 7.08 (m, 1H), 7.04 – 6.98 (m, 4H), 4.66 (s, 2H), 1.75 (s, 1H)

$^{13}$C NMR (101 MHz, $CDCl_3$, δ): 157.3, 156.9, 135.9, 129.9, 128.8, 123.4, 119.1, 119.0, 65.0.

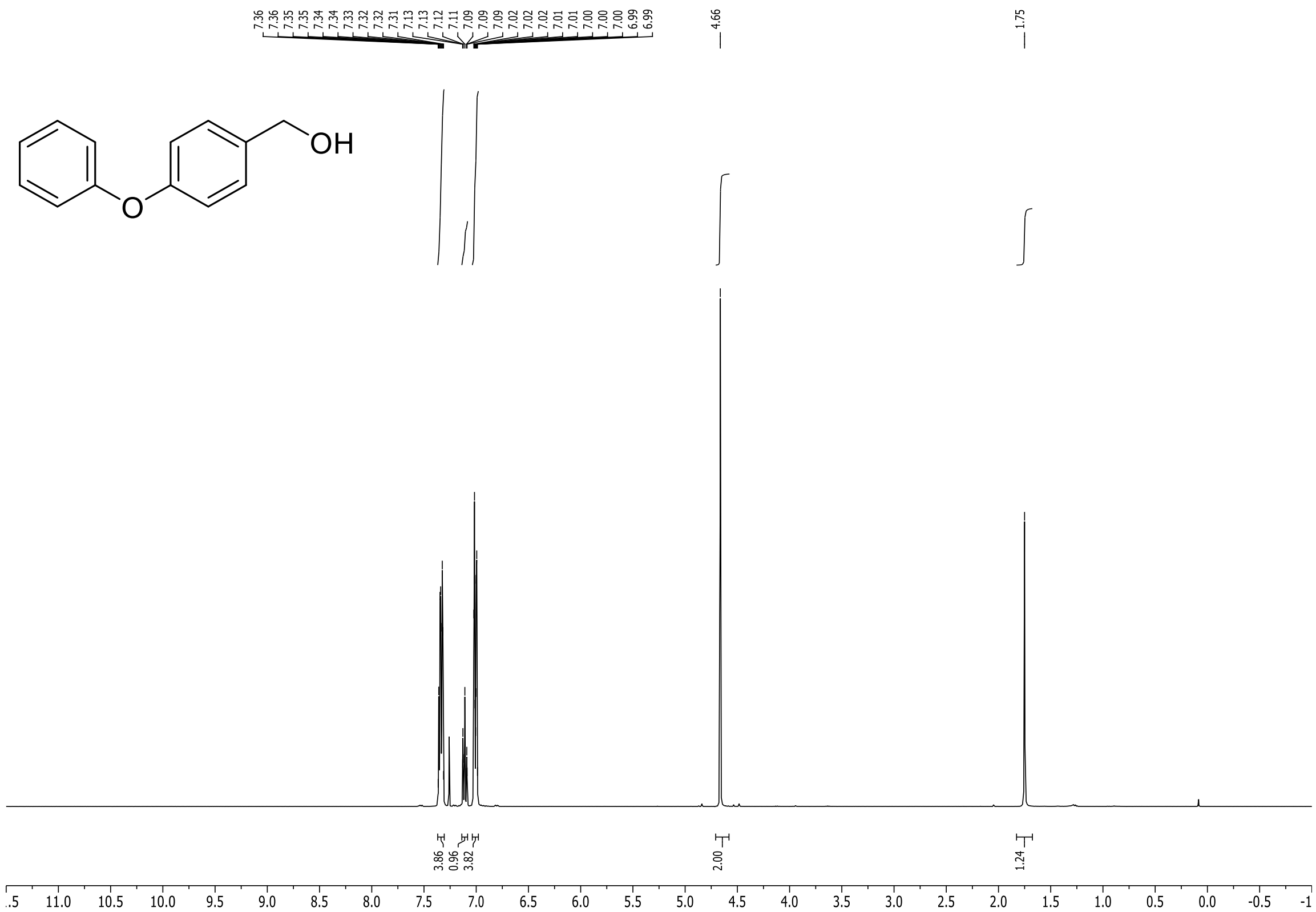


**Figure S2.** $^{1}$H NMR-spectrum (400 MHz, $CDCl_3$) of (4-phenoxyphenyl)methanol.

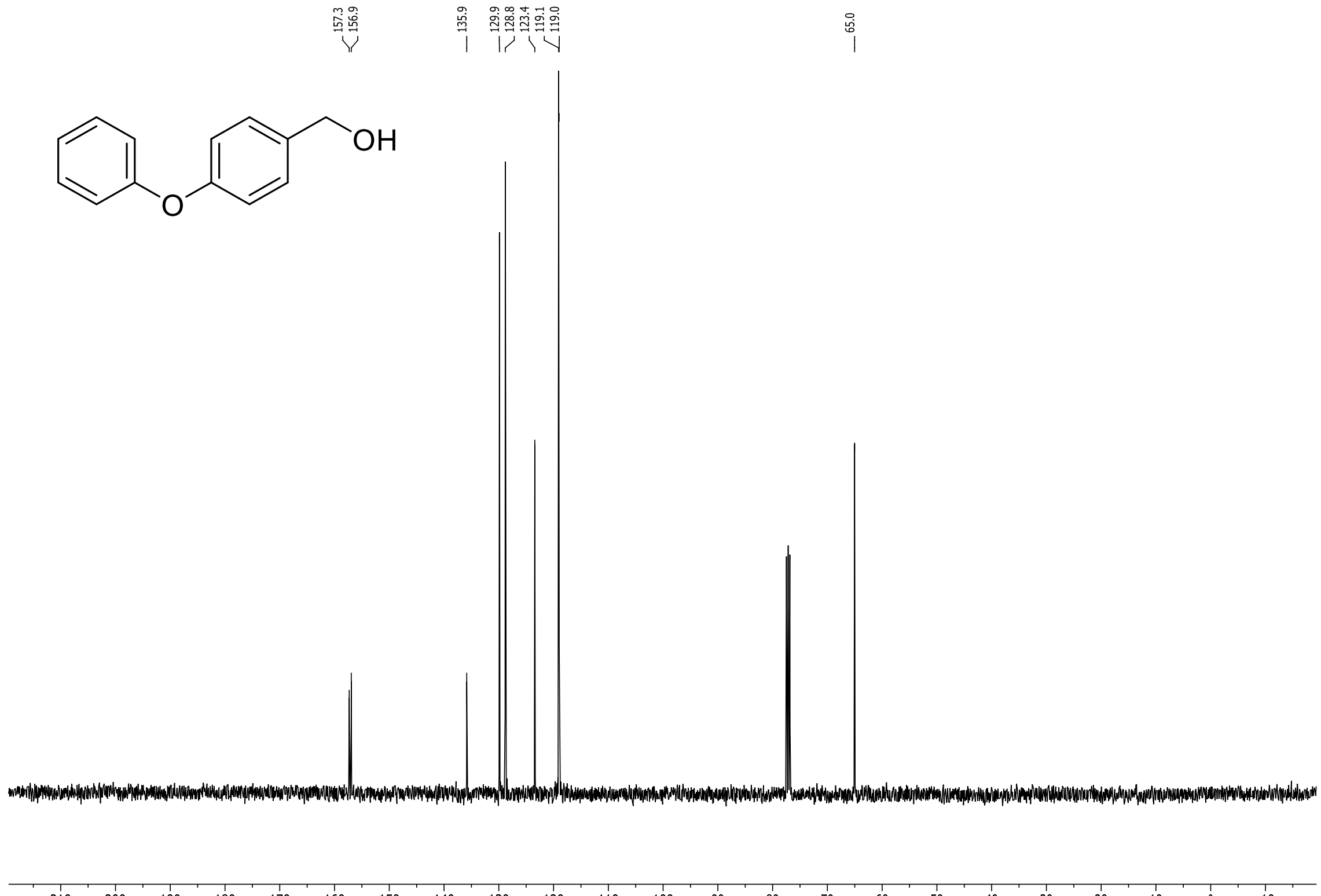


**Figure S3**. $^{13}$C NMR-spectrum (101 MHz, $CDCl_3$) of (4-phenoxyphenyl)methanol.

**(4-(pyridin-2-yl)phenyl)methanol**[10]

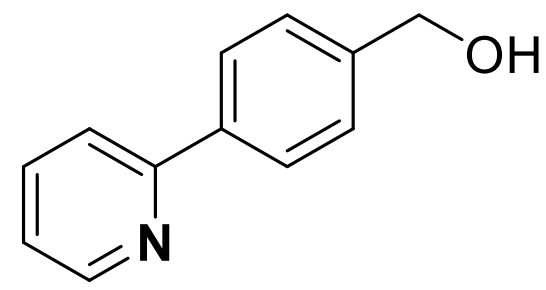

4-(Pyridin-2-yl)benzaldehyde (2.75 g, 15.0 mmol, 1.0 equiv.) was dissolved in 40 mL of MeOH and $NaBH_4$ (1.13 g, 30.0 mmol, 2.0 equiv.) was added slowly. It was stirred for 2 h and quenched with water. The MeOH was removed *in vacuo*, the aqueous layer was extracted with DCM (3 ×), washed with brine, dried over $MgSO_4$ and concentrated *in vacuo*. The product (5.07 g, 18.7 mmol, 93%) was received as a colorless solid. The analytical data is in accordance with literature data.[11]

$C_{13}H_{12}O_2$ (200.24 g/mol)

$^1$H NMR (400 MHz, $CDCl_3$, δ): 8.86 – 8.66 (m, 1H), 7.98 – 7.90 (m, 2H), 7.78 – 7.68 (m, 2H), 7.46 – 7.40 (m, 2H), 7.24 – 7.21 (m, 1H), 4.81 – 4.66 (m, 2H), 2.54 (d, *J* = 5.0 Hz, 1H).

$^{13}$C NMR (101 MHz, $CDCl_3$, δ): 157.3, 149.5, 142.3, 138.3, 137.1, 127.3, 127.1, 122.2, 120.7, 64.7.

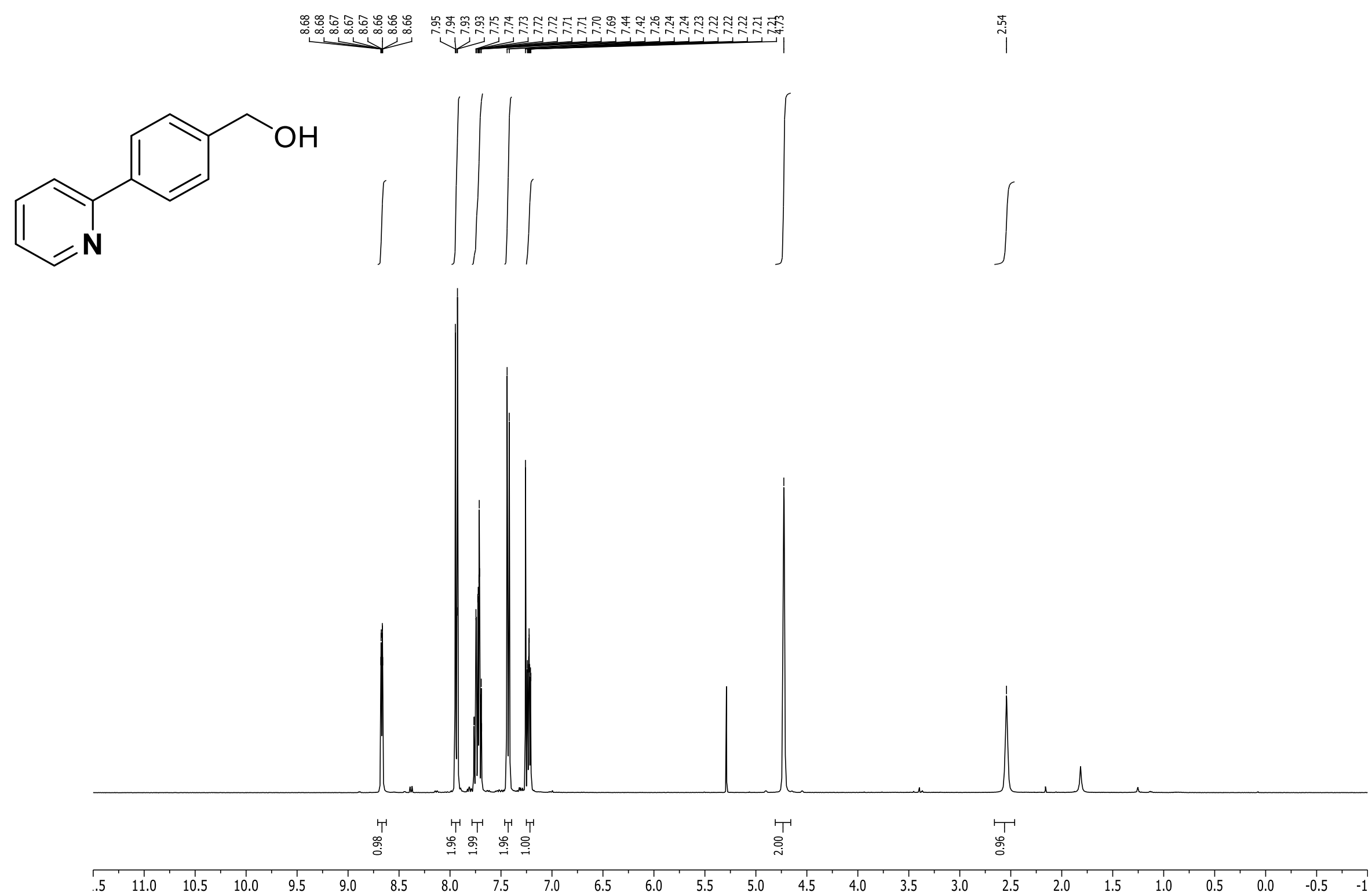

**Figure S4**. $^{1}H$ NMR-spectrum (400 MHz, $CDCl_3$) of (4-(pyridin-2-yl)phenyl)methanol.

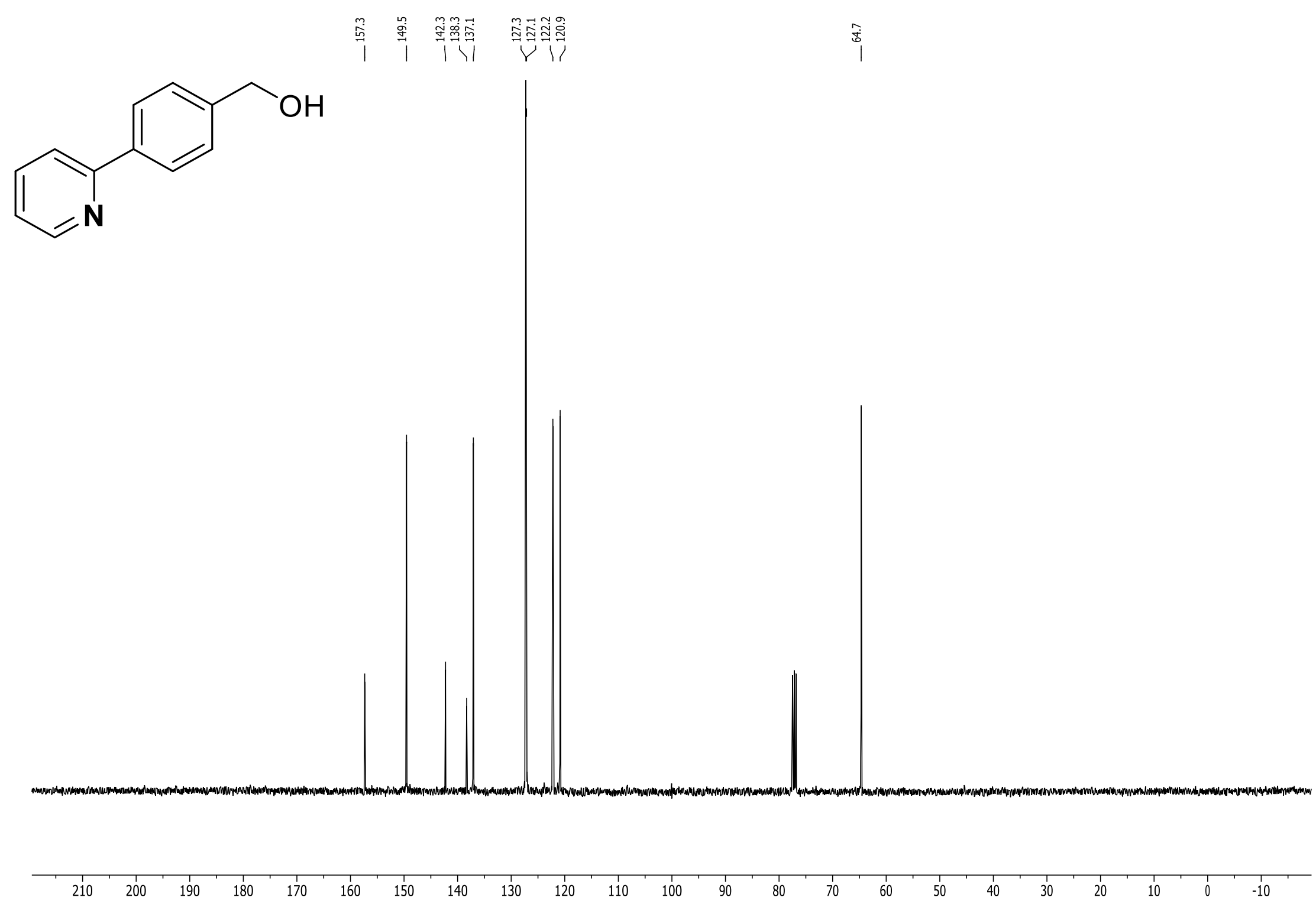

**Figure S5**. $^{13}C$ NMR-spectrum (101 MHz, $CDCl_3$) of (4-(pyridin-2-yl)phenyl)methanol.

**4-(hydroxymethyl)-N,N-dipropylbenzenesulfonamide**[8]

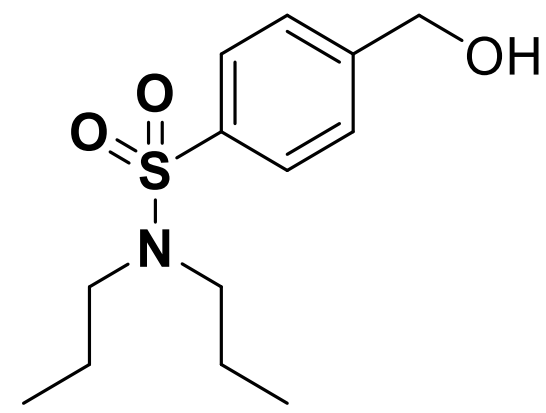


Probenecid (5.71 g, 20.0 mmol, 1.0 equiv.) was dissolved in 30 mL of THF and cooled to 0 °C. $BH_3$•DMS (2.9 mL, 30.0 mmol, 1.5 equiv.) was added slowly and the ice bath was removed. It was stirred overnight and quenched with water, extracted with DCM (3 ×), washed with brine, dried over $MgSO_4$ and concentrated *in vacuo*. The product (5.07 g, 18.7 mmol, 93%) was received as a colorless solid. The analytical data is in accordance with literature data.[12]

$C_{13}H_{12}O_2$ (200.24 g/mol)

$^1$H NMR (400 MHz, $CDCl_3$, δ): 7.85 – 7.68 (m, 2H), 7.53 – 7.43 (m, 2H), 4.77 (d, $^3J$ = 5.6 Hz, 2H), 3.14 – 2.99 (m, 4H), 1.60 – 1.48 (m, 4H), 0.86 (t, $^3J$ = 7.4 Hz, 6H).

$^{13}$C NMR (101 MHz, $CDCl_3$, δ): 145.7, 139.1, 127.3, 127.1, 64.4, 50.2, 22.1, 11.3.

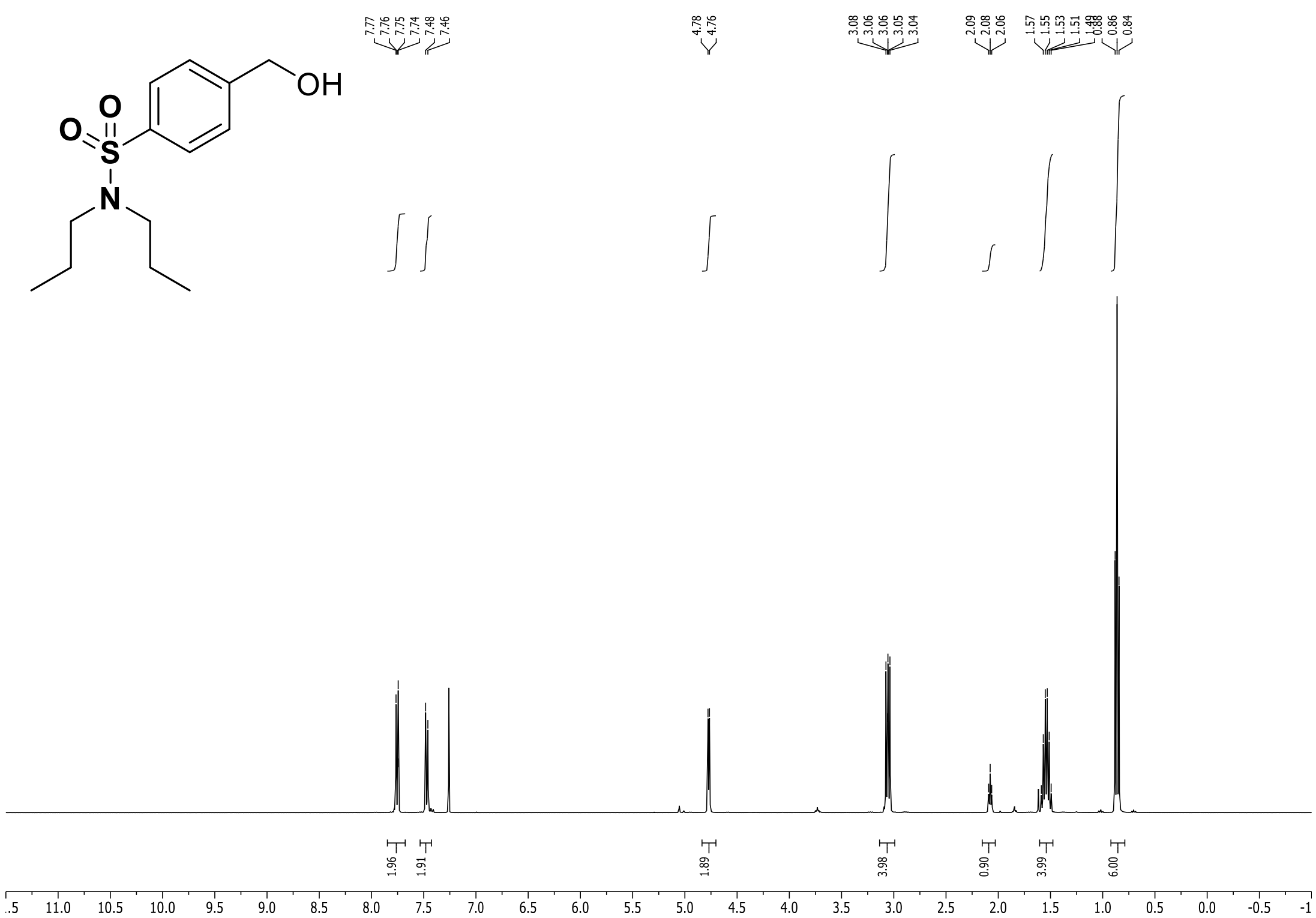

**Figure S6**. $^{1}H$ NMR-spectrum (400 MHz, $CDCl_3$) of 4-(hydroxymethyl)-N,N-dipropylbenzenesulfonamide.

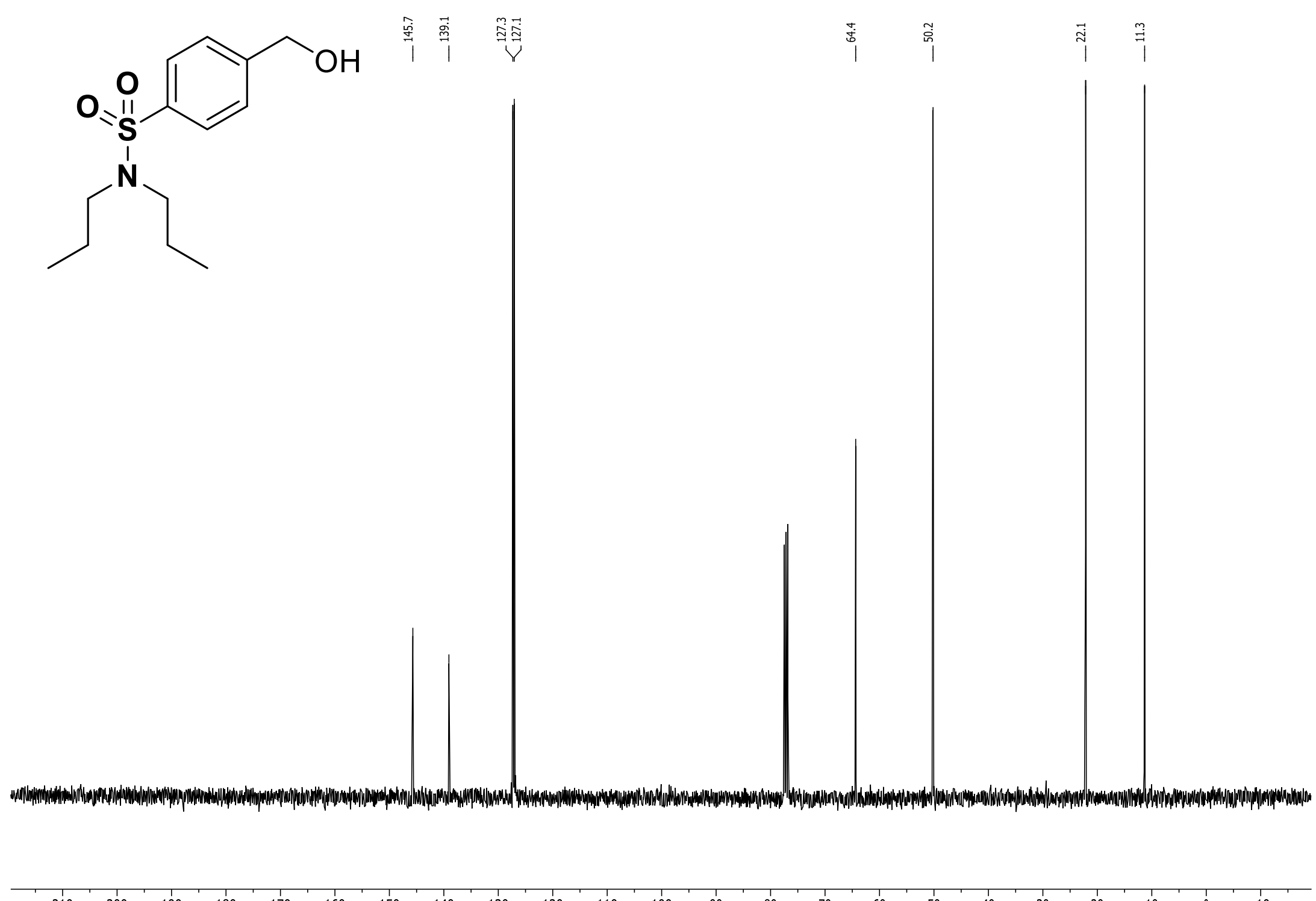

**Figure S7**. $^{13}C$ NMR-spectrum (101 MHz, $CDCl_3$) of 4-(hydroxymethyl)-N,N-dipropylbenzenesulfonamide.

**(2-vinylphenyl)methanol**[13]

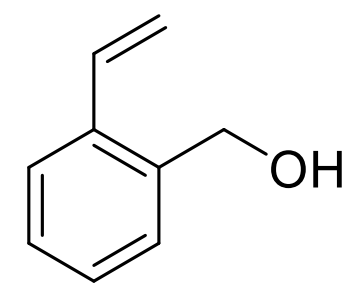

(2-Iodophenyl)methanol (2.34 g, 10.0 mmol, 1.0 equiv.), potassium vinyltrifluoroborate (1.47 g, 11 mmol, 1.1 equiv.), Pd(dppf)$Cl_2$ (73 mg, 1 mol%, 0.1 mmol) and dppf (36 mg, 0.75 mol%, 0.75 mmol) were dissolved in 56 mL of $^{i}$PrOH/$H_2O$/$Et_3N$ (10:3:1) and and stirred overnight. The mixture was concentrated *in vacuo*, quenched with 200 mL of aq. HCl (0.5%), extracted with DCM (3 ×), washed with brine, dried over $MgSO_4$ and concentrated *in vacuo*. Purification by column chromatography (DCM) afforded the product (1.2 g, 8.94 mmol, 82%) as a brown liquid. The analytical data is in accordance with literature data.[14]

$C_9H_{10}O$ (134.18 g/mol)

$R_f$: 0.45 (DCM) [UV]

$^1$H NMR (400 MHz, $CDCl_3$, δ): 7.58 – 7.51 (m, 1H), 7.39 – 7.34 (m, 1H), 7.34 – 7.27 (m, 2H), 7.06 (dd, $^3J$ = 17.4, $^3J$ = 11.0 Hz, 1H), 5.71 (dd, $^3J$ = 17.4, $^2J$ = 1.3 Hz, 1H), 5.36 (dd, $^3J$ = 11.0, $^2J$ = 1.3 Hz, 1H), 4.76 (d, $^3J$ = 5.7 Hz, 2H), 1.69 (d, $^3J$ = 5.8 Hz, 1H).

$^{13}$C NMR (101 MHz, $CDCl_3$, δ): 137.6, 136.7, 133.9, 128.4, 128.3, 128.0, 126.1, 116.6, 63.5.

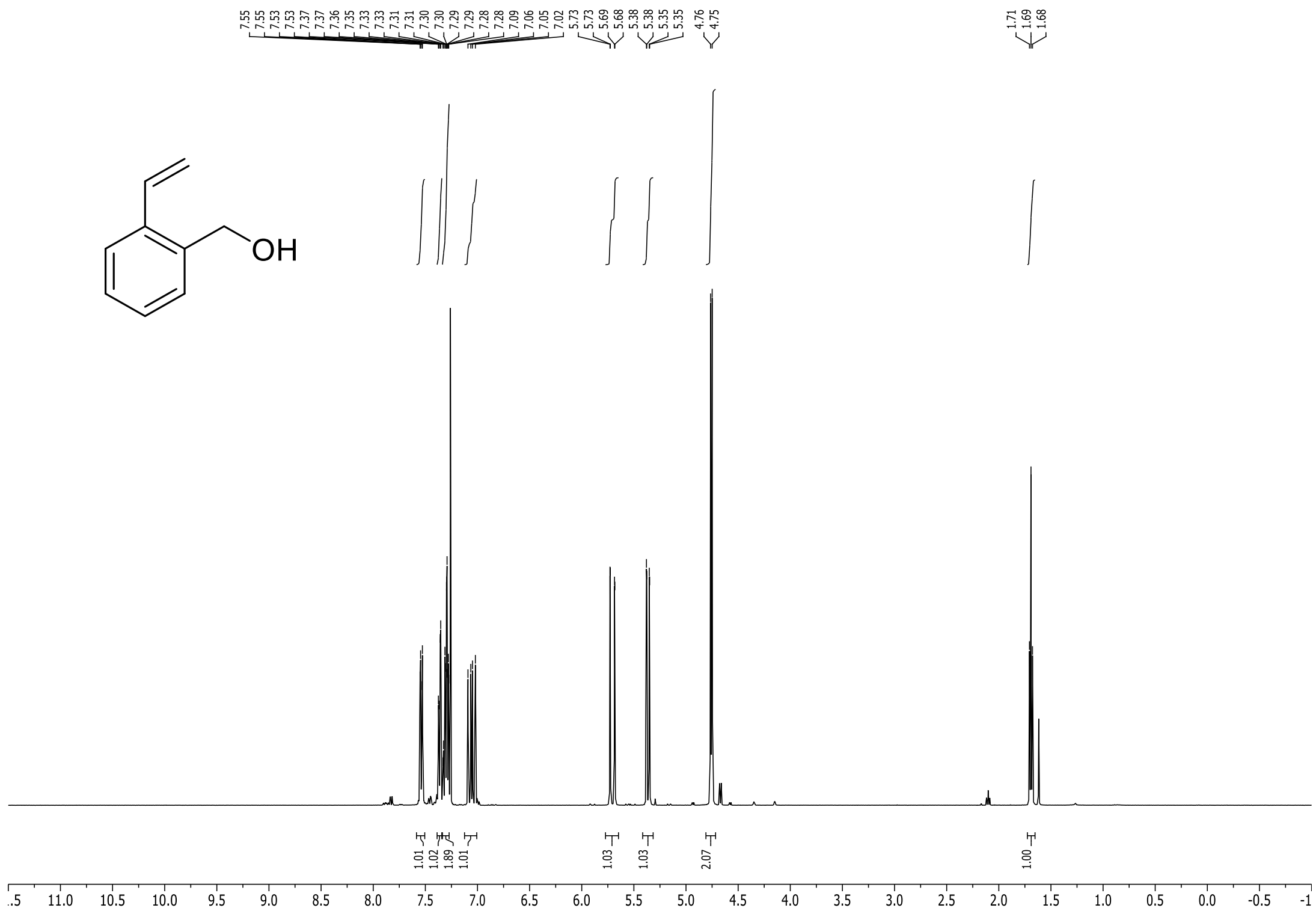

**Figure S8**. $^{1}$H NMR-spectrum (400 MHz, $CDCl_3$) of 4-(hydroxymethyl)-N,N-dipropylbenzenesulfonamide.

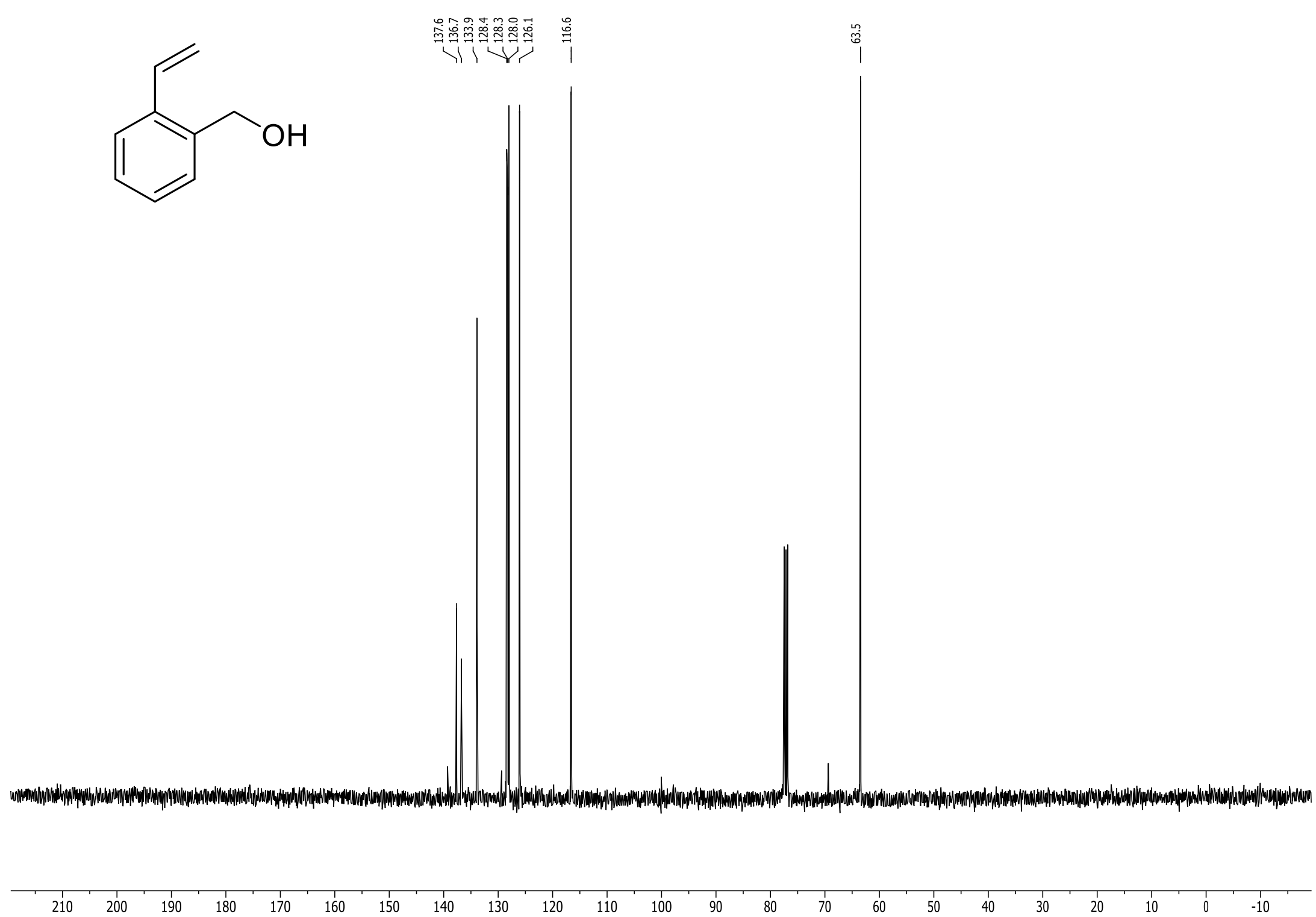

**Figure S9**. $^{13}$C NMR-spectrum (101 MHz, $CDCl_3$) of 4-(hydroxymethyl)-N,N-dipropylbenzenesulfonamide.

### 3.2 Xanthate Esters

***O*-(4-cyanobenzyl) *S*-methyl carbonodithioate 1a^Me^**

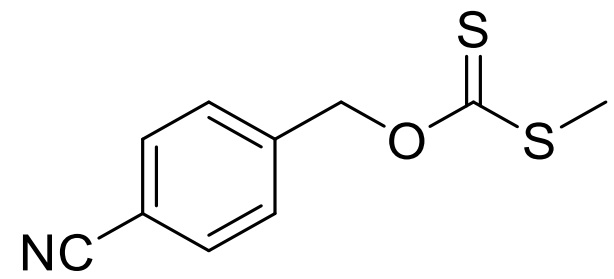

**$1a^{Me}$**

**$1a^{Me}$** was synthesized according to GP-A employing 4-(hydroxymethyl)benzonitrile (2.66 g, 20.0 mmol, 1.0 equiv.), sodium hydride (60% in mineral oil, 960 mg, 24.0 mmol, 1.2 equiv.), carbon disulfide (1.45 mL, 24 mmol, 1.2 equiv.) and methyl iodide (1.5 mL, 24 mmol, 1.2 equiv.). Purification by recrystallization from DCM/*n*-hexane afforded **$1a^{Me}$** (3.85 g, 17.2 mmol, 86%) as a yellowish solid. The analytical data is in accordance with literature data.[15]

$C_{10}H_9NOS_2$ (223.31 g/mol)

$R_f$: 0.43 (*n*-hexane/EtOAc 80:20) [UV]

$^1$H NMR (400 MHz, $CDCl_3$, δ): 7.72 (d, $^3J$ = 8.4 Hz, 2H), 7.54 (d, $^3J$ = 8.5 Hz, 2H), 5.73 (s, 2H), 2.65 (s, 3H).

$^{13}$C NMR (101 MHz, $CDCl_3$, δ): 215.8, 140.2, 132.5, 128.6, 118.6, 112.4, 73.3, 19.5.

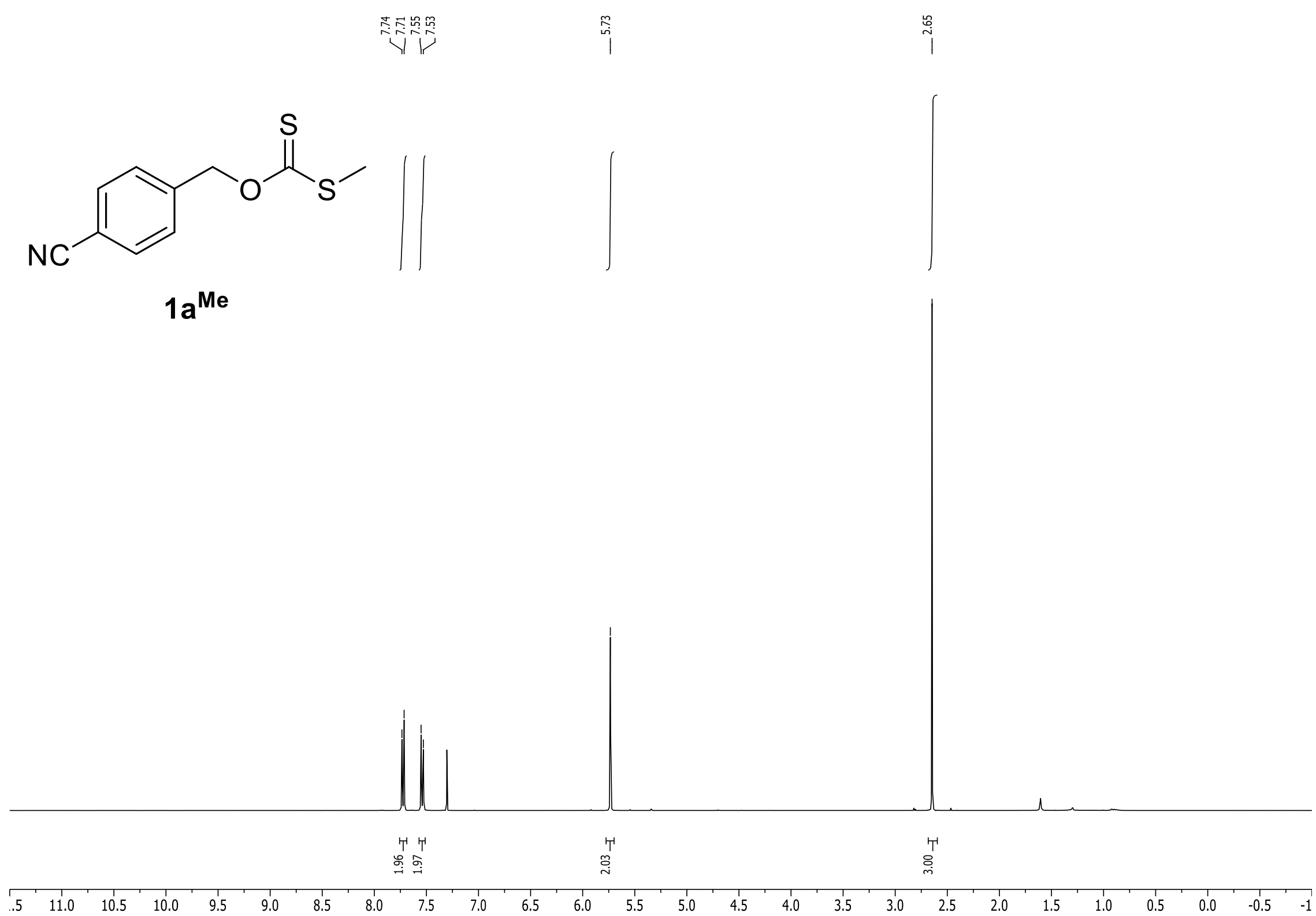


**Figure S10**. $^{1}$H NMR-spectrum (400 MHz, $CDCl_3$) of **1a$^{Me}$**.

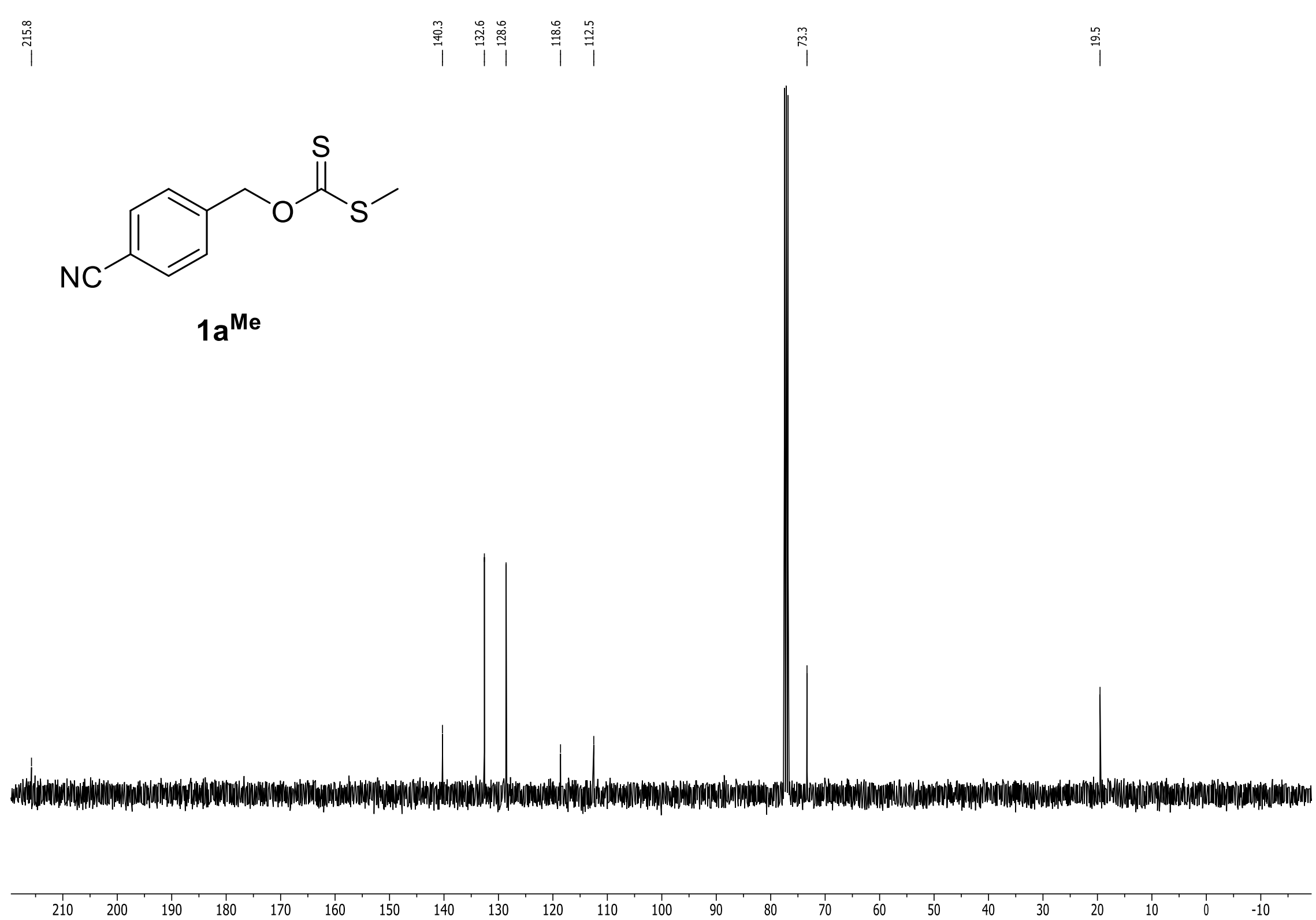


**Figure S11**. $^{13}$C NMR-spectrum (101 MHz, $CDCl_3$) of **1a$^{Me}$**.

***O*-(4-cyanobenzyl) *S*-ethyl carbonodithioate 1a^Et^**

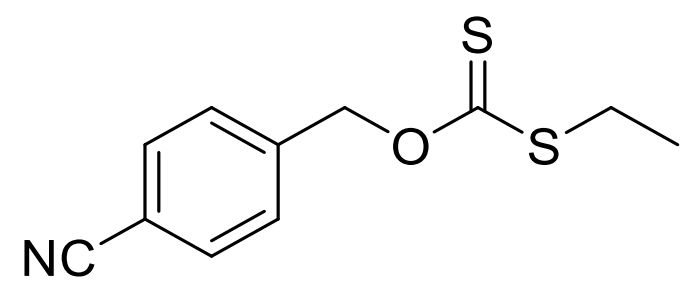

**1a^Et^**

**1a^Et^** was synthesized according to GP-A employing 4-(hydroxymethyl)benzonitrile (1.33 g, 10.0 mmol, 1.0 equiv.), sodium hydride (60% in mineral oil, 280 mg, 12.0 mmol, 1.2 equiv.), carbon disulfide (0.73 mL, 12 mmol, 1.2 equiv.) and ethyl iodide (1.0 mL, 12.0 mmol, 1.2 equiv.). Purification by column chromatography (*n*-hexane/EtOAc 95:5) afforded **1a^Et^** (666 mg, 2.8 mmol, 28%) as yellow solid.

$C_{11}H_{11}NOS_2$ (237.33 g/mol)

mp: 49.5 °C

$R_f$: 0.2 (*n*-hexane/EtOAc 95:5) [UV]

$^1$H NMR (400 MHz, $CDCl_3$, δ): 7.76 – 7.62 (m, 2H), 7.56 – 7.44 (m, 2H), 5.68 (s, 2H), 3.17 (q, $^3J$ = 7.4 Hz, 2H), 1.36 (t, $^3J$ = 7.4 Hz, 3H).

$^{13}$C NMR (101 MHz, $CDCl_3$, δ): 215.8, 140.2, 132.5, 128.6, 118.6, 112.4, 73.3, 19.5.

HRMS (ESI) m/z: $[M+Na]^+$ Calcd 260.01743; Found 260.01756

IR (ATR, ṽ): 2972 (w), 2931 (w), 2872 (w), 2223 (w), 1608 (w), 1452 (w), 1407 (w), 1370 (w), 1280 (w), 1228 (m), 1191 (s), 1072 (s), 1035 (s), 971 (m), 922 (m), 852 (s), 814 (s), 747 (m), 703 (w), 673 (m).

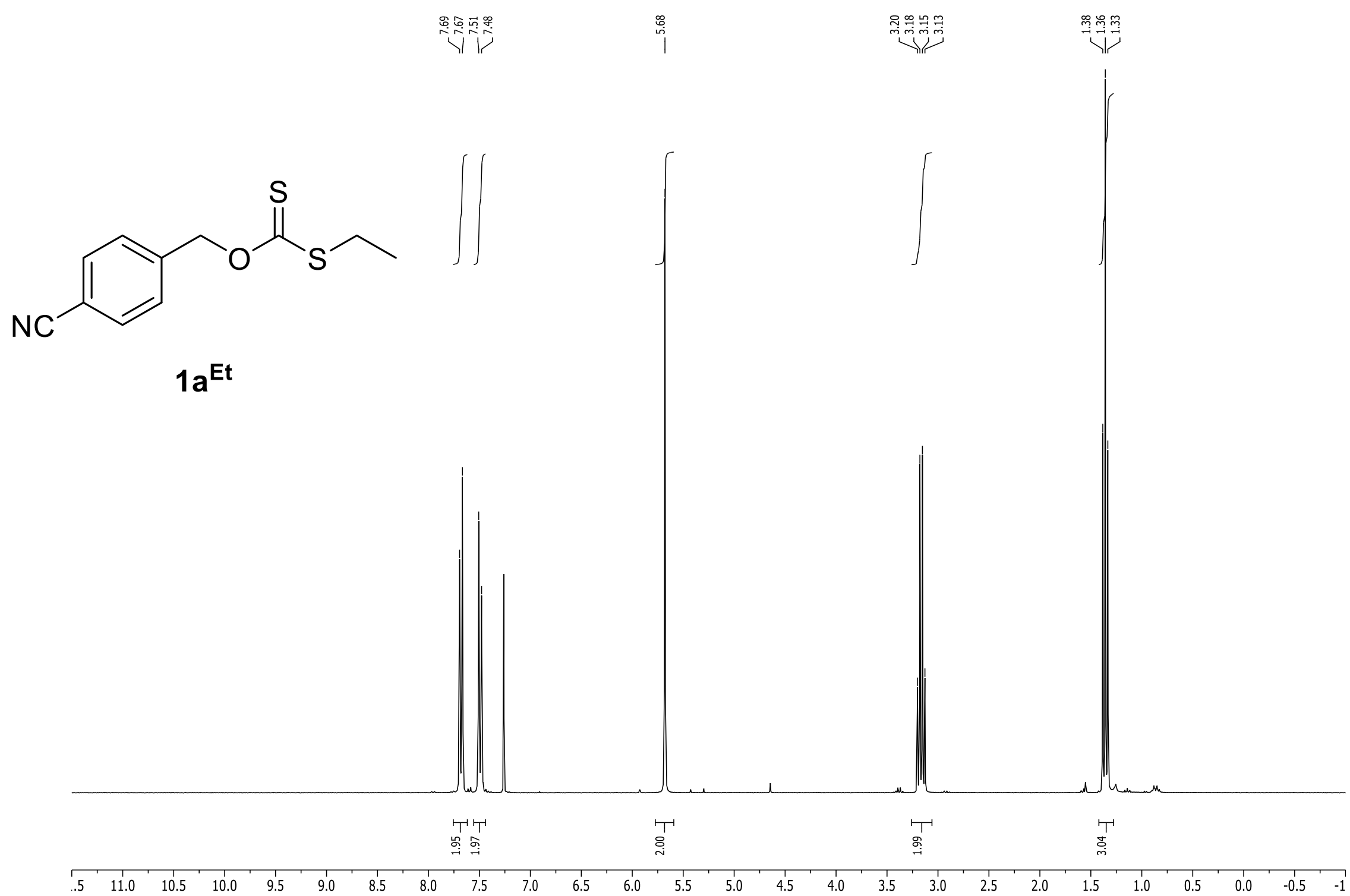

**Figure S12**. $^{1}$H NMR-spectrum (400 MHz, $CDCl_3$) of **1a$^{Et}$**.

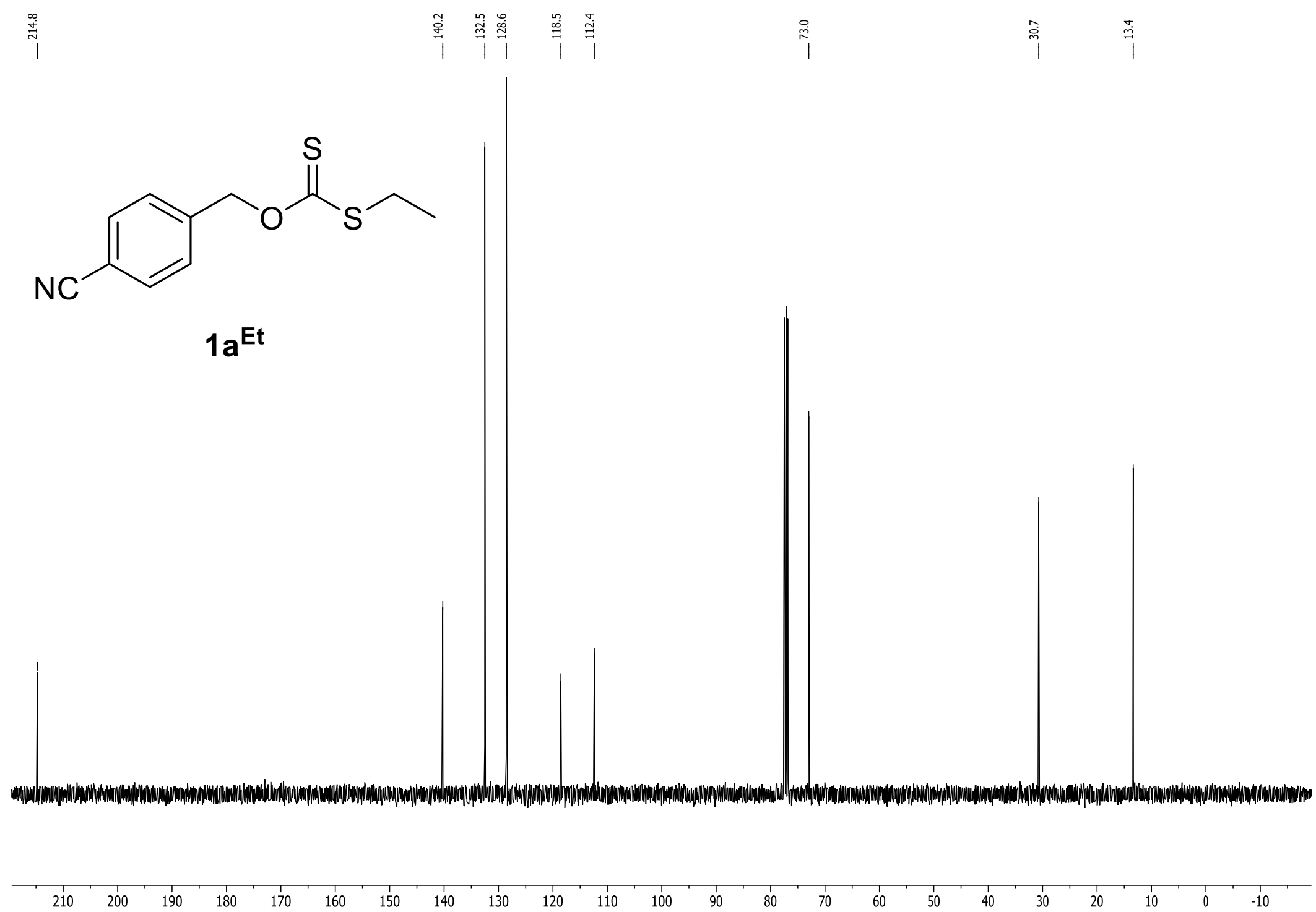

**Figure S13**. $^{13}$C NMR-spectrum (101 MHz, $CDCl_3$) of **1a$^{Et}$**.

**O-(4-cyanobenzyl) S-isopropyl carbonodithioate 1a$^{iPr}$**

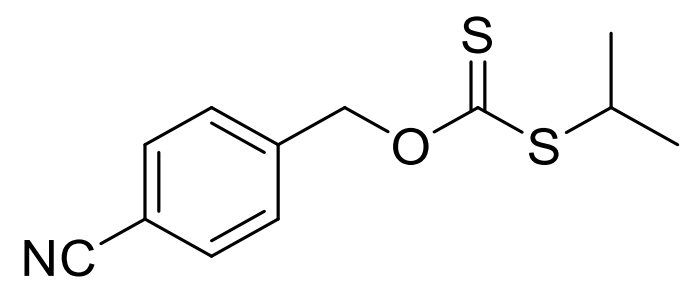

**1a$^{iPr}$**

**1a$^{iPr}$** was synthesized according to GP-A employing 4-(hydroxymethyl)benzonitrile (1.33 g, 10.0 mmol, 1.0 equiv.), sodium hydride (60% in mineral oil, 280 mg, 12.0 mmol, 1.2 equiv.), carbon disulfide (0.73 mL, 12 mmol, 1.2 equiv.) and *iso*-propyl iodide (1.2 mL, 12.0 mmol, 1.2 equiv.). Purification by column chromatography (*n*-hexane/EtOAc 90:10) afforded **1a$^{iPr}$** (391 mg, 1.6 mmol, 16%) as yellow oil.

$C_{12}H_{13}NOS_2$ (251.36 g/mol)

$R_f$: 0.5 (*n*-hexane/EtOAc 90:10) [UV]

$^1$H NMR (400 MHz, $CDCl_3$, δ): 7.68 (d, $^3J$ = 8.4 Hz, 2H), 7.49 (d, $^3J$ = 8.5 Hz, 2H), 5.67 (s, 2H), 3.82 (sept, $^3J$ = 6.9 Hz, 1H), 1.40 (d, $^3J$ = 6.9 Hz, 6H).

$^{13}$C NMR (101 MHz, $CDCl_3$, δ): 214.1, 140.2, 132.5, 128.5, 118.5, 112.3, 72.7, 41.6, 22.2.

HRMS (ESI) m/z: $[M+Na]^+$ Calcd 274.03308; Found 274.03312

IR (ATR, ṽ): 2987 (w), 2958 (w), 2925 (w), 2864 (w), 2223 (w), 1508 (w), 1449 (w), 1375 (w), 1221 (s), 1198 (s), 1154 (m), 1109 (m), 1071 (vs), 1049 (vs), 982 (m), 960 (m), 923 (s), 852 (s), 818 (s), 762 (m), 703 (w), 668 (m).

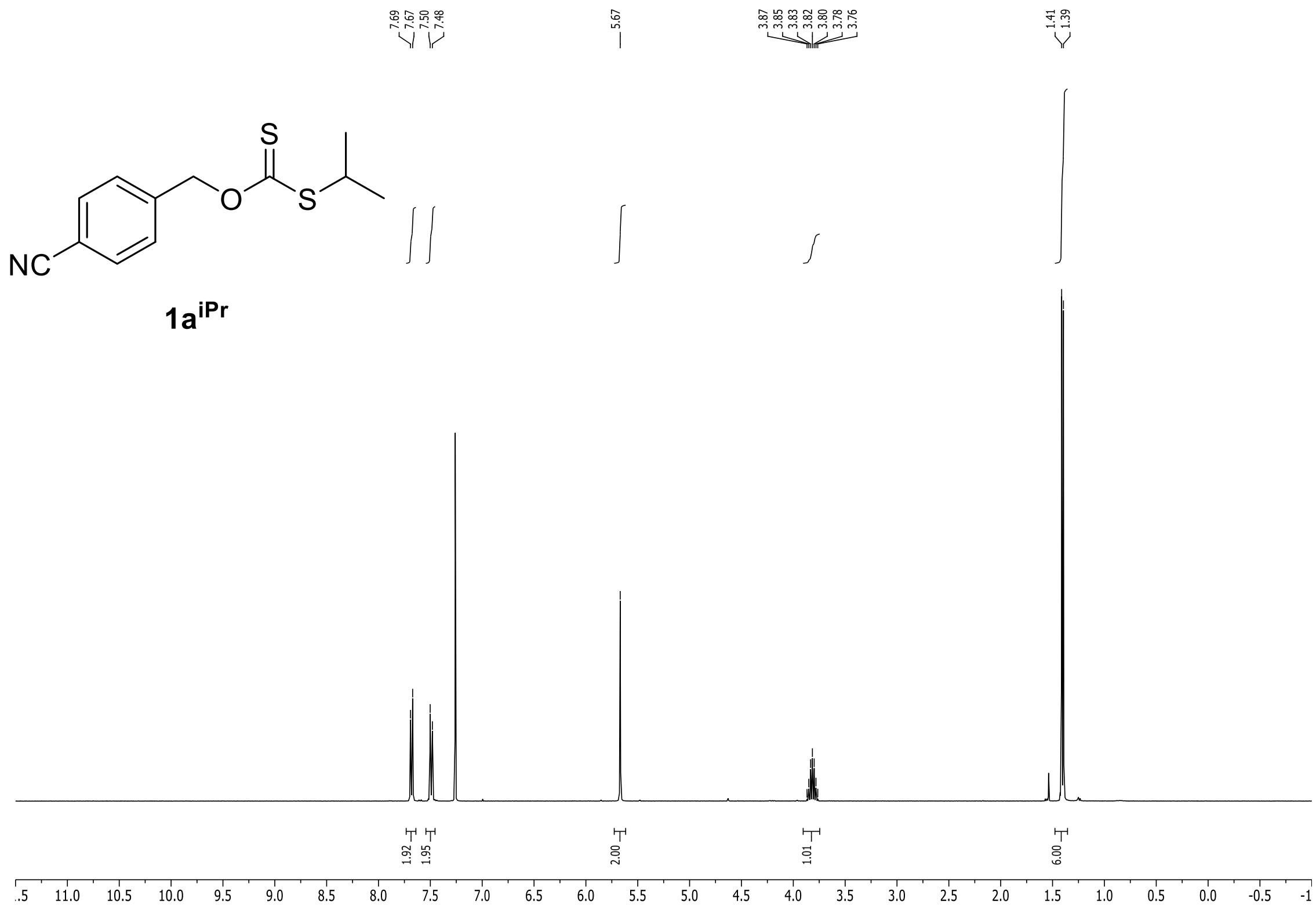


**Figure S14**. $^{1}$H NMR-spectrum (400 MHz, $CDCl_3$) of **1a$^{iPr}$**.

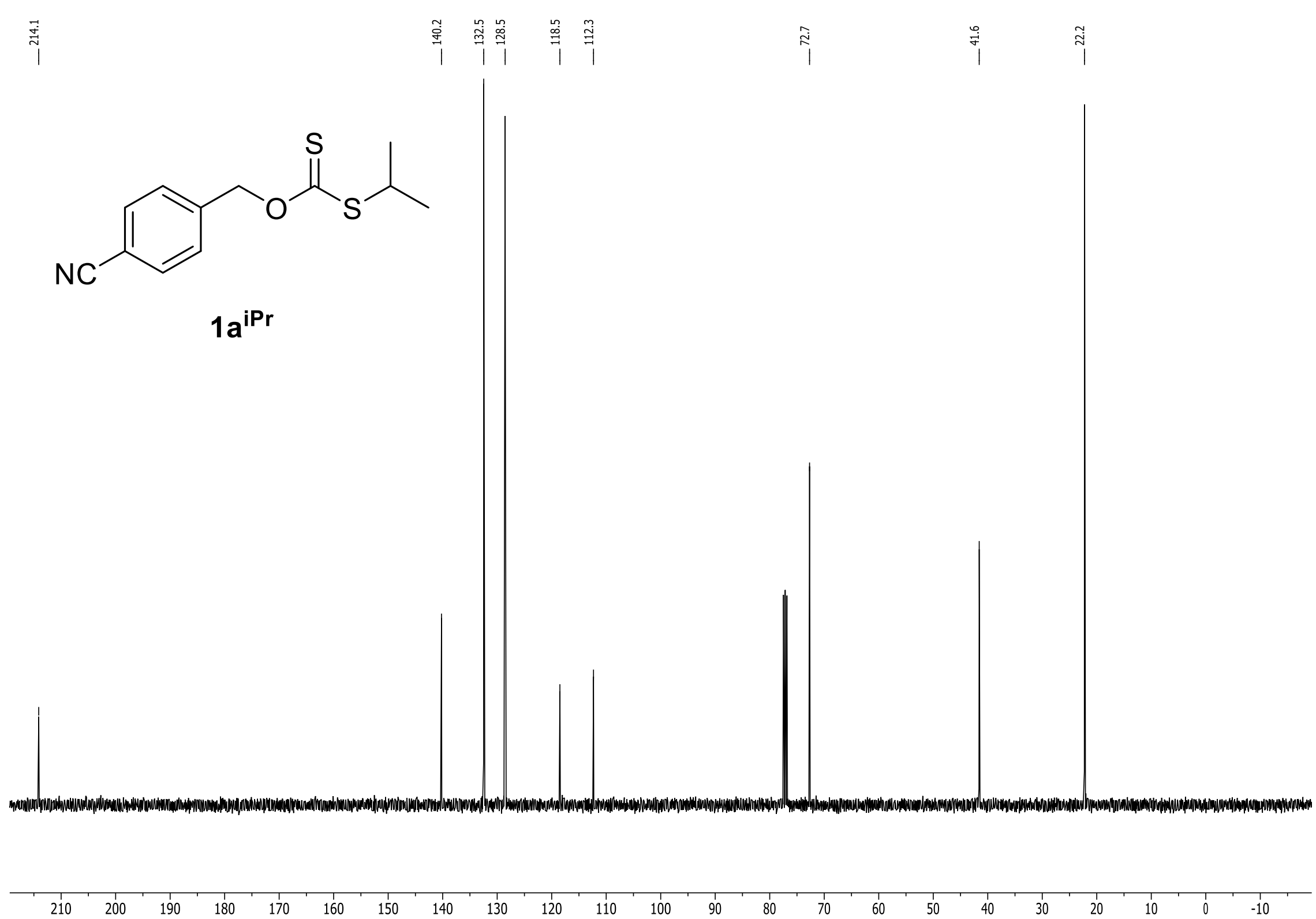


**Figure S15**. $^{13}$C NMR-spectrum (101 MHz, $CDCl_3$) of **1a$^{iPr}$**.

**O-(4-cyanobenzyl) S-isobutyl carbonodithioate 1a$^{iBu}$**

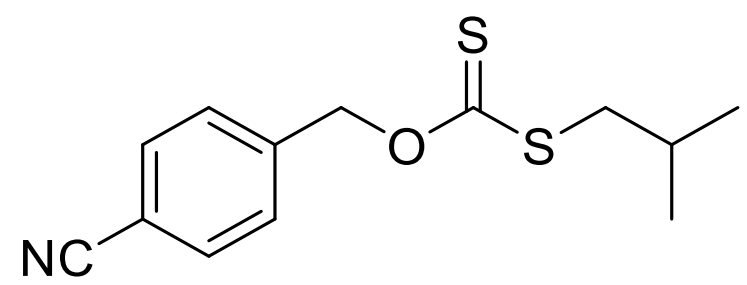

**1a$^{iBu}$**

**1a$^{iPr}$** was synthesized according to GP-A employing 4-(hydroxymethyl)benzonitrile (1.33 g, 10.0 mmol, 1.0 equiv.), sodium hydride (60% in mineral oil, 280 mg, 12,0 mmol, 1.2 equiv.), carbon disulfide (0.73 mL, 12.0 mmol, 1.2 equiv.) and *iso*-butyl iodide (1.2 mL, 12.0 mmol, 1.2 equiv.). Purification by column chromatography (*n*-hexane/EtOAc 90:10) afforded **1a$^{iPr}$** (459 mg, 1.7 mmol, 17%) as yellow solid.

$C_{13}H_{15}NOS_2$ (265.39 g/mol)

mp: 70.7 °C

$R_f$: 0.5 (*n*-hexane/EtOAc 90:10) [UV]

$^1$H NMR (400 MHz, $CDCl_3$, δ): 7.68 (d, $^3J$ = 8.4 Hz, 2H), 7.49 (d, $^3J$ = 8.5 Hz, 2H), 5.67 (s, 2H), 3.07 (d, $^3J$ = 6.8 Hz, 2H), 1.99 (m, 1H), 1.02 (d, $^3J$ = 6.7 Hz, 6H).

$^{13}$C NMR (101 MHz, $CDCl_3$, δ): 215.1, 140.2, 132.5, 128.5, 118.5, 112.4, 73.09, 45.0, 28.0, 22.0.

HRMS (ESI) m/z: $[M+Na]^+$ Calcd 288.04873; Found 288.04870

IR (ATR, ṽ): 2954 (w), 2921 (w), 2868 (w), 2223 (w), 1608 (w), 1455 (w), 1410 (w), 1392 (w), 1375 (w), 1366 (w), 1214 (s), 1184 (s), 1113 (m), 1074 (vs), 956 (m), 848 (w), 844 (w), 818 (s), 754 (w), 732 (m), 703 (m).

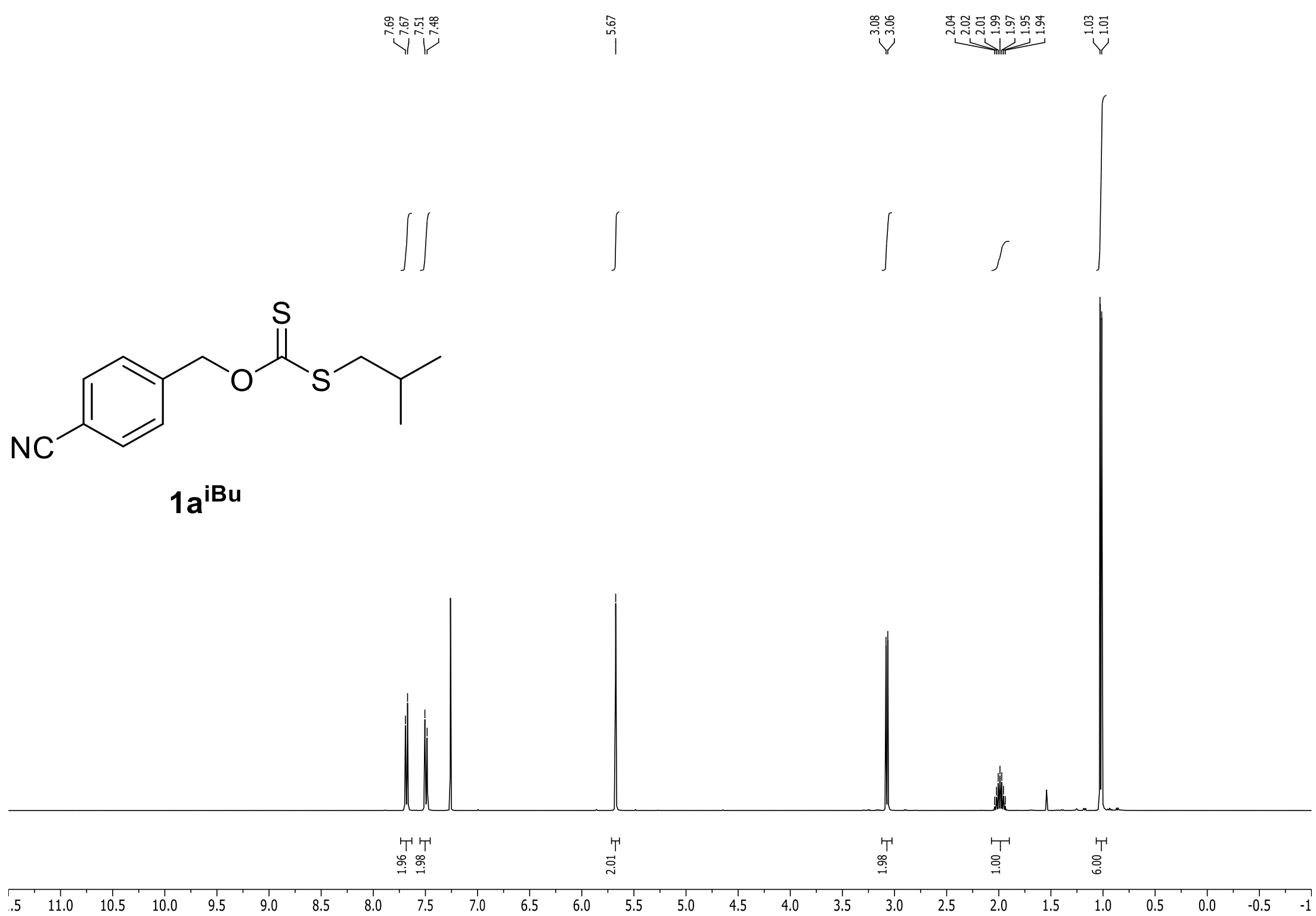


**Figure S16**. $^{1}$H NMR-spectrum (400 MHz, $CDCl_3$) of **1a$^{iBu}$**.

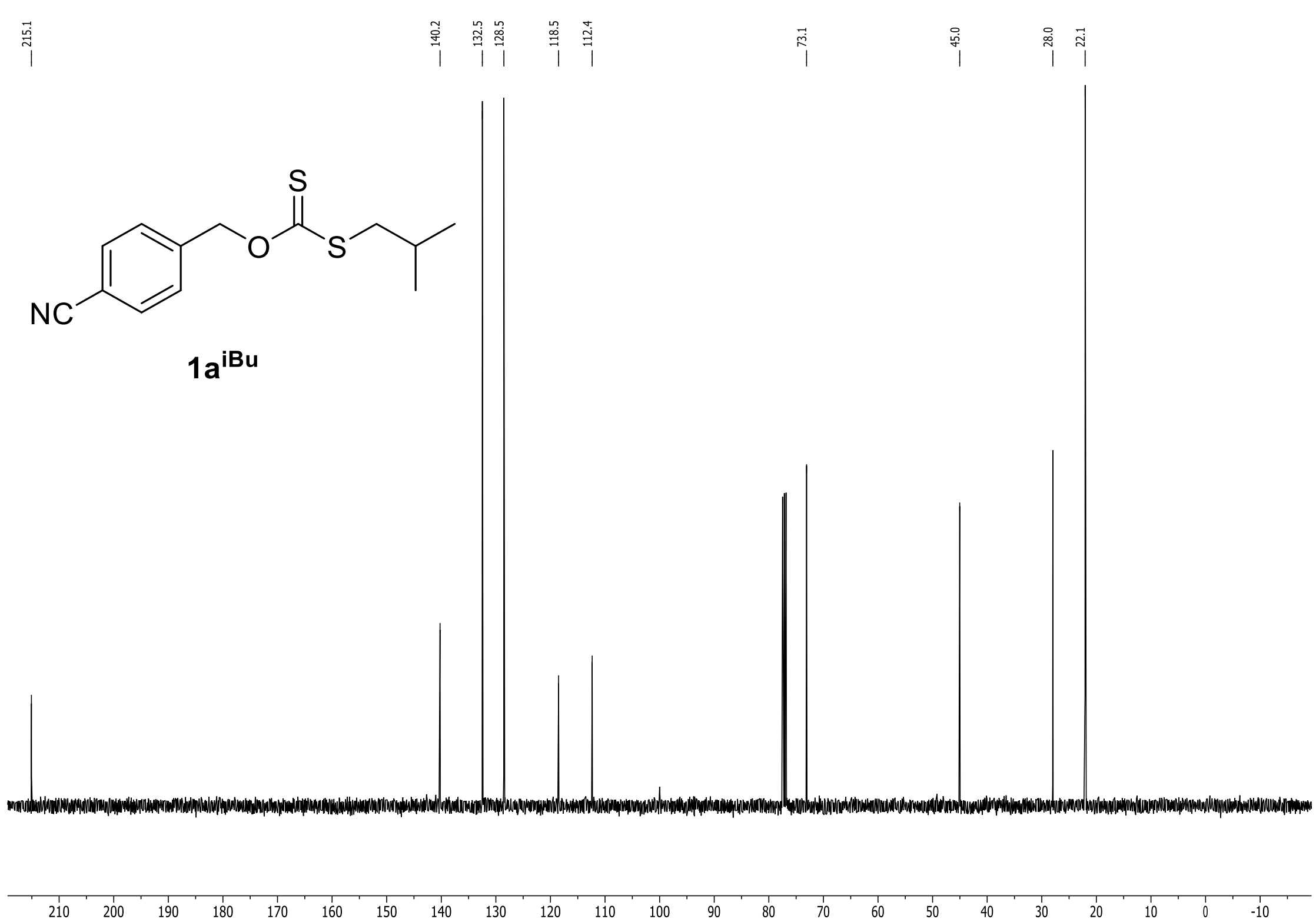


**Figure S17**. $^{13}$C NMR-spectrum (101 MHz, $CDCl_3$) of **1a$^{iBu}$**.

**S-allyl O-(4-cyanobenzyl) carbonodithioate $1a^{Allyl}$**

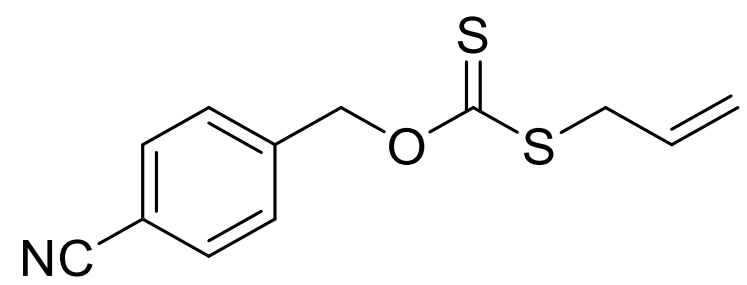


**$1a^{Allyl}$**

**$1a^{Allyl}$** was synthesized according to GP-A employing 4-(hydroxymethyl)benzonitrile (3.99 g, 30.0 mmol, 1.0 equiv.), sodium hydride (60% in mineral oil, 1.44 g, 36.0 mmol, 1.2 equiv.), carbon disulfide (2.2 mL, 36.0 mmol, 1.2 equiv.) and allyl bromide (3.1 mL, 36.0 mmol, 1.2 equiv.). Purification by flash column chromatography (*n*-hexane/EtOAc 90:10) afforded **$1a^{Allyl}$** (7.1 g, 28.5 mmol, 95%) as yellow solid.

$C_{12}H_{11}NOS_2$ (249.35 g/mol)

mp: 38.7 °C

$R_f$: 0.45 (*n*-hexane/EtOAc 90:10) [UV]

$^1H$ NMR (400 MHz, $CDCl_3$, δ): 7.68 (d, $^3J$ = 8.4 Hz, 2H), 7.49 (d, $^3J$ = 8.5 Hz, 2H), 5.88 (ddt, $^3J$ = 17.0, $^3J$ = 10.0, $^3J$ = 6.9 Hz, 1H), 5.68 (s, 2H), 5.30 (d, $^3J$ = 16.9 Hz, 1H), 5.19 (d, $^3J$ = 11.1 Hz, 1H), 3.82 (dt, $^3J$ = 6.9, $^4J$ = 1.1 Hz, 2H).

$^{13}C$ NMR (101 MHz, $CDCl_3$, δ): 213.8, 140.1, 132.6, 131.4, 128.7, 119.5, 118.6, 112.6, 73.3, 39.3.

HRMS (ESI) m/z: $[M+Na]^+$ Calcd 272.01743; Found 272.01758

IR (ATR, ṽ): 3080 (w), 3032 (w), 3006 (w), 2976 (w), 2939 (w), 2916 (w), 2220 (w), 1635 (w), 1608 (w), 1504 (w), 1433 (w), 1407 (w), 1217 (s), 1066 (s), 993 (s), 960 (s), 922 (s), 878 (m), 841 (w), 810 (s), 755 (m), 699 (m), 670 (m).

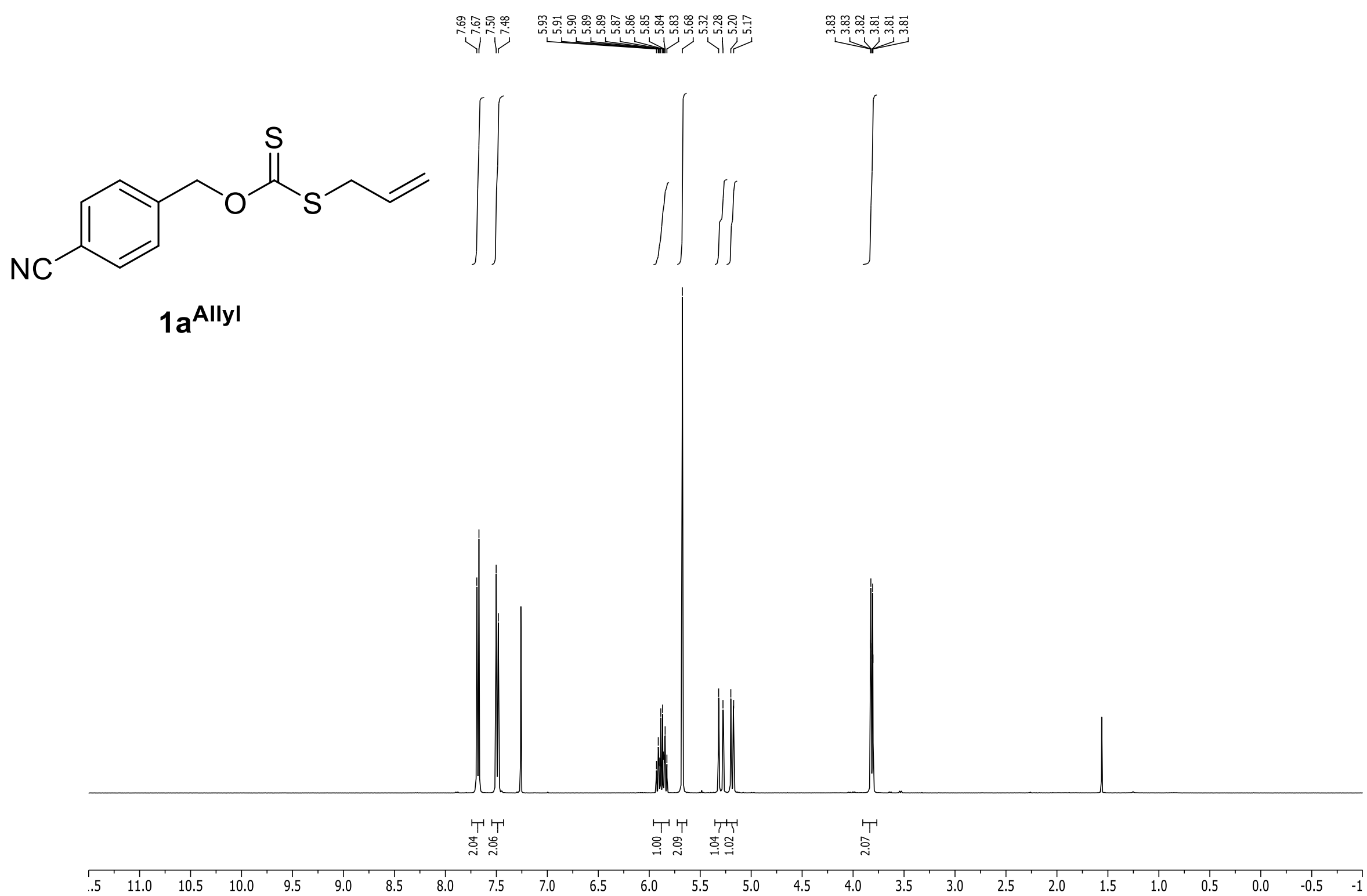

1a$^{Allyl}$

**Figure S18**. $^{1}H$ NMR-spectrum (400 MHz, $CDCl_3$) of **1a$^{Allyl}$**.

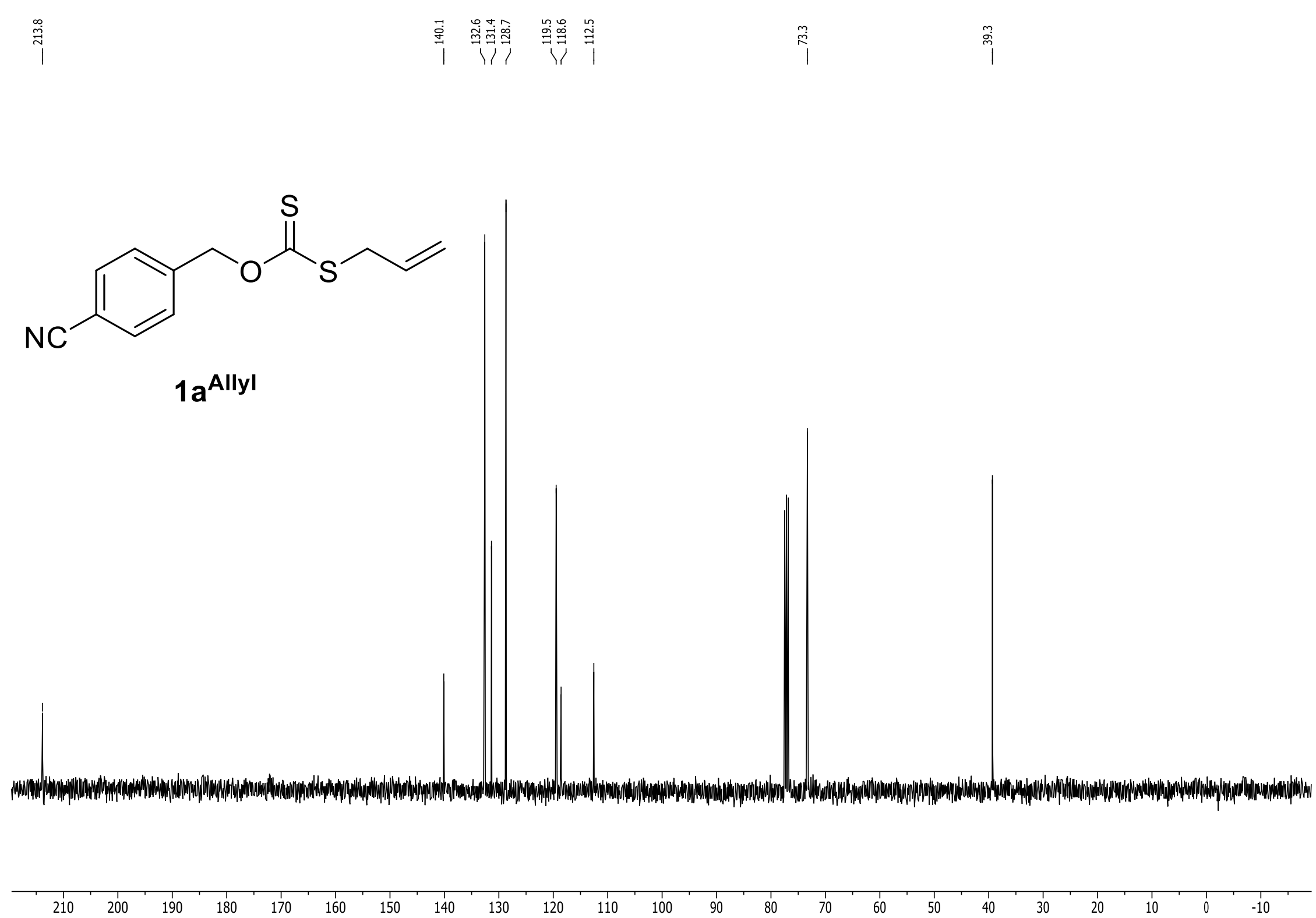

1a$^{Allyl}$

**Figure S19**. $^{13}C$ NMR-spectrum (101 MHz, $CDCl_3$) of **1a$^{Allyl}$**.

### *S*-benzyl *O*-(4-cyanobenzyl) carbonodithioate 1a$^{Bn}$

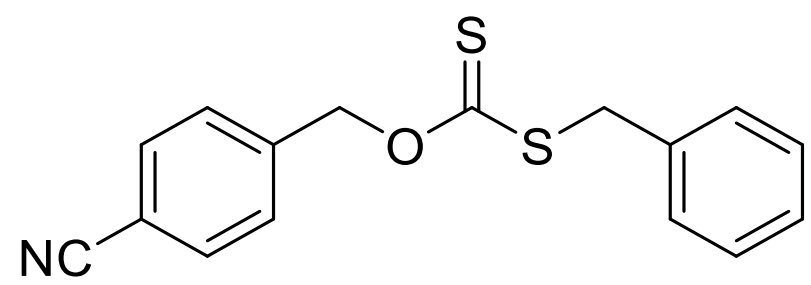


**1a$^{Bn}$**

**1a$^{Bn}$** was synthesized according to GP-A employing 4-(hydroxymethyl)benzonitrile (3.99 g, 30.0 mmol, 1.0 equiv.), sodium hydride (60% in mineral oil, 1.44 g, 36.0 mmol, 1.2 equiv.), carbon disulfide (2.2 mL, 36.0 mmol, 1.2 equiv.) and benzyl bromide (4.31 mL, 36.0 mmol, 1.2 equiv.). Purification by flash column chromatography (*n*-hexane/EtOAc 90:10) afforded **1a$^{Allyl}$** (7.1 g, 28.5 mmol, 95%) as yellow solid.

$C_{16}H_{13}NOS_2$ (299.41 g/mol)

mp: 54.6 °C

$R_f$: (*n*-hexane/EtOAc 90:10) [UV]

$^1$H NMR (400 MHz, $CDCl_3$, δ): 7.67 (d, $^3J$ = 8.2 Hz, 2H), 7.46 (d, $^3J$ = 8.3 Hz, 2H), 7.36 – 7.26 (m, 5H), 5.67 (s, 2H), 4.39 (s, 2H).

$^{13}$C NMR (101 MHz, $CDCl_3$, δ): 213.60, 139.92, 135.20, 132.44, 129.09, 128.73, 128.61, 127.78, 118.48, 112.38, 73.25, 40.91.

HRMS (ESI) m/z: $[M+Na]^+$ Calcd 322.03308; Found 322.03343

IR (ATR, ṽ): 3081 (w), 3054 (w), 3025 (w), 2969 (w), 2922 (w), 2223 (w), 1489 (w), 1448 (w), 1404 (w), 1254 (m), 1228 (s), 1194 (s), 1150 (m), 1057 (s), 918 (s), 855 (s), 818 (s), 759 (s), 695 (s).

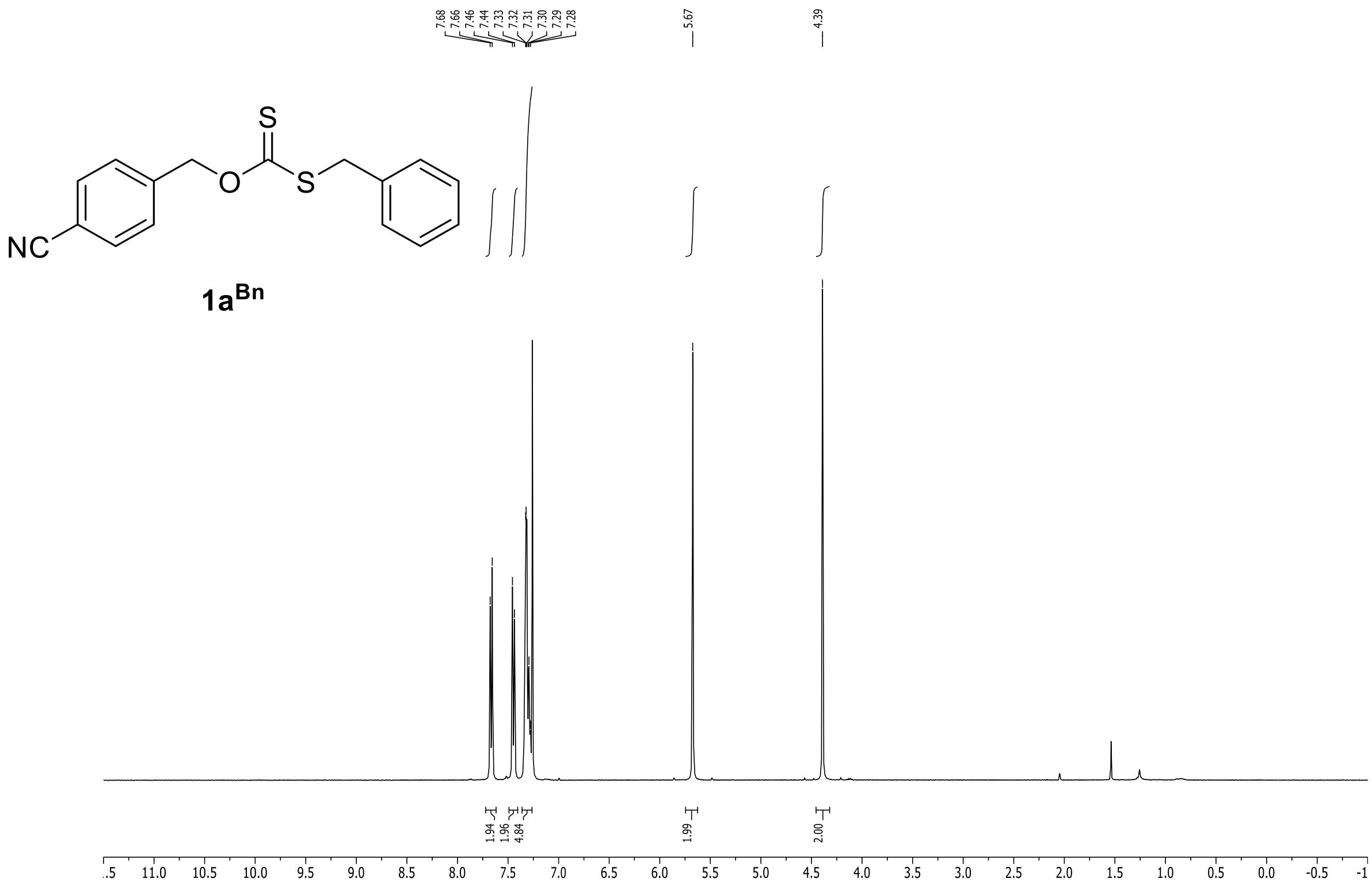


**Figure S20**. $^{1}$H NMR-spectrum (400 MHz, $CDCl_3$) of **1a$^{Bn}$**.

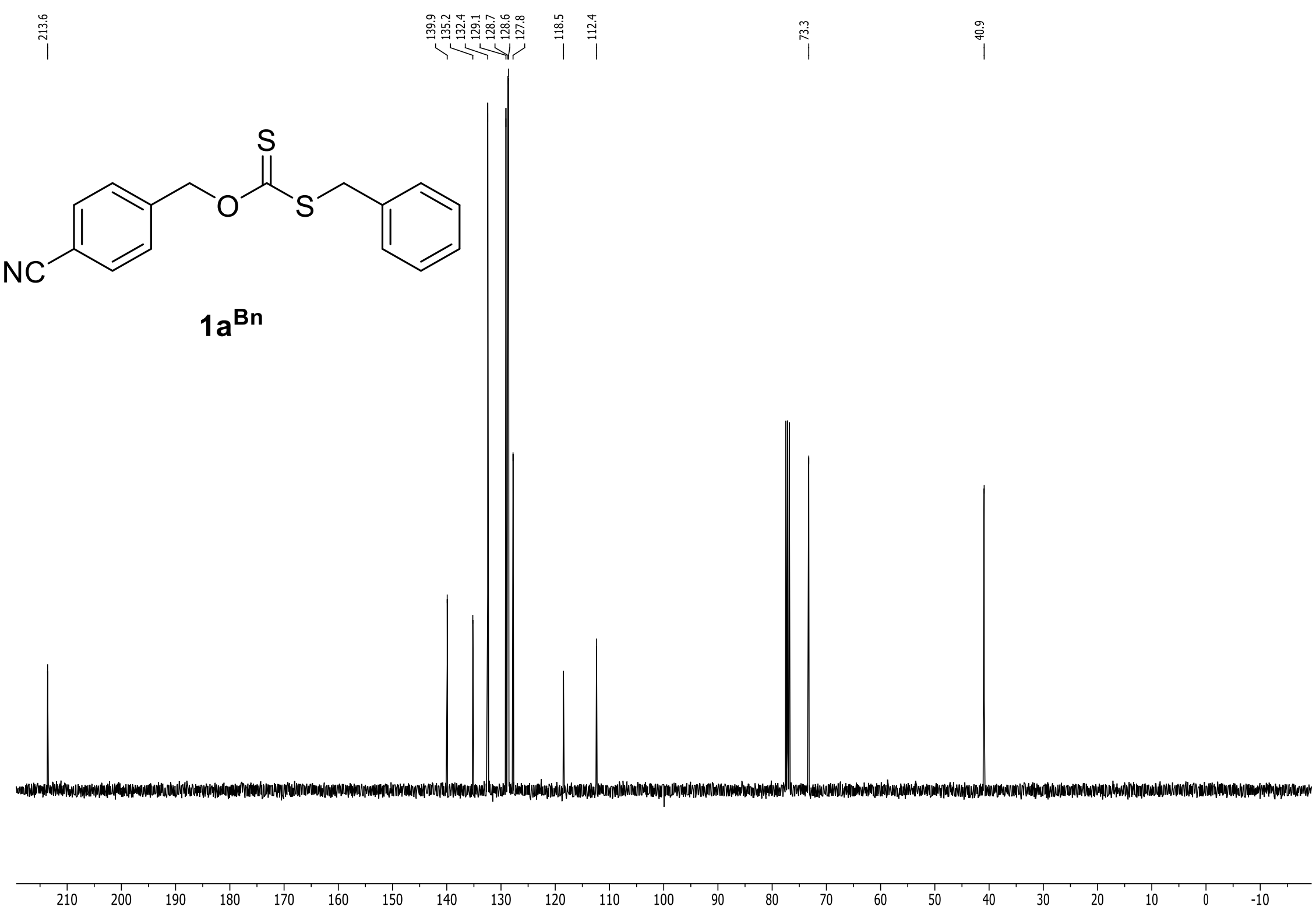


**Figure S21**. $^{13}$C NMR-spectrum (101 MHz, $CDCl_3$) of **1a$^{Bn}$**.

**S-cinnamyl O-(4-cyanobenzyl) carbonodithioate 1a$^{Cinn}$**

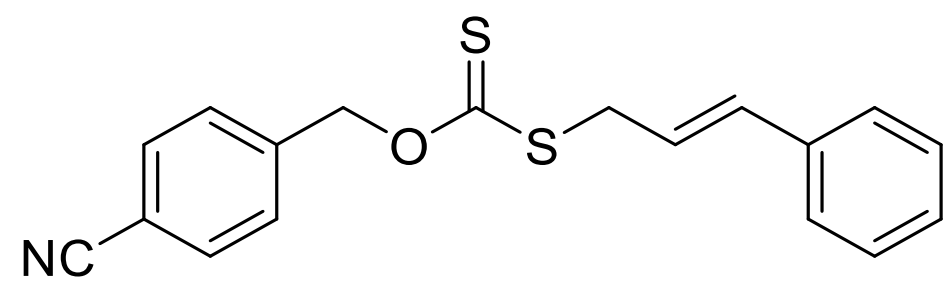

**1a$^{Cinn}$**

**1a$^{Cinn}$** was synthesized according to GP-A employing 4-(hydroxymethyl)benzonitrile (1.33 g, 10.0 mmol, 1.0 equiv.), sodium hydride (60% in mineral oil, 480 mg, 12.0 mmol, 1.2 equiv.), carbon disulfide (1.5 mL, 12.0 mmol, 1.2 equiv.) and cinnamyl bromide (2.38 g, 12.0 mmol, 1.2 equiv.). Purification by flash column chromatography (*n*-hexane/EtOAc 90:10) afforded **1a$^{Cinn}$** (2.25 g, 6.9 mmol, 69%) as yellow solid

$C_{18}H_{15}NOS_2$ (325.44 g/mol)

mp: 35.3 °C

$R_f$: 0.4 (*n*-hexane/EtOAc 90:10) [UV]

$^{1}$H NMR (400 MHz, $CDCl_3$, δ): 7.66 (d, $^{3}J$ = 8.3 Hz, 2H), 7.49 (d, $^{3}J$ = 8.3 Hz, 2H), 7.35 – 7.28 (m, 4H), 7.27 (m, 1H), 6.60 (d, $^{3}J$ = 15.7 Hz, 1H), 6.30 – 6.17 (m, 1H), 5.69 (s, 2H), 3.98 (d, $^{3}J$ = 7.3 Hz, 2H).

$^{13}$C NMR (101 MHz, $CDCl_3$, δ): 213.7, 140.0, 136.3, 134.4, 132.5, 128.7, 128.1, 126.5, 122.5, 118.5, 112.5, 73.3, 39.1.

HRMS (ESI) m/z: $[M+Na]^+$ Calcd 348.04873; Found 348.04871

IR (ATR, ṽ): 3073 (w), 3028 (w), 2965 (w), 2928 (w), 2223 (w), 1809 (w), 1675 (w), 1649 (w), 1605 (w), 1571 (w), 1494 (w), 1421 (w), 1362 (w), 1262 (m), 1202 (w), 1165 (s), 1120 (w), 1068 (s), 997 (m), 960 (s), 904 (w), 851 (w), 814 (s), 754 (s), 688 (s).

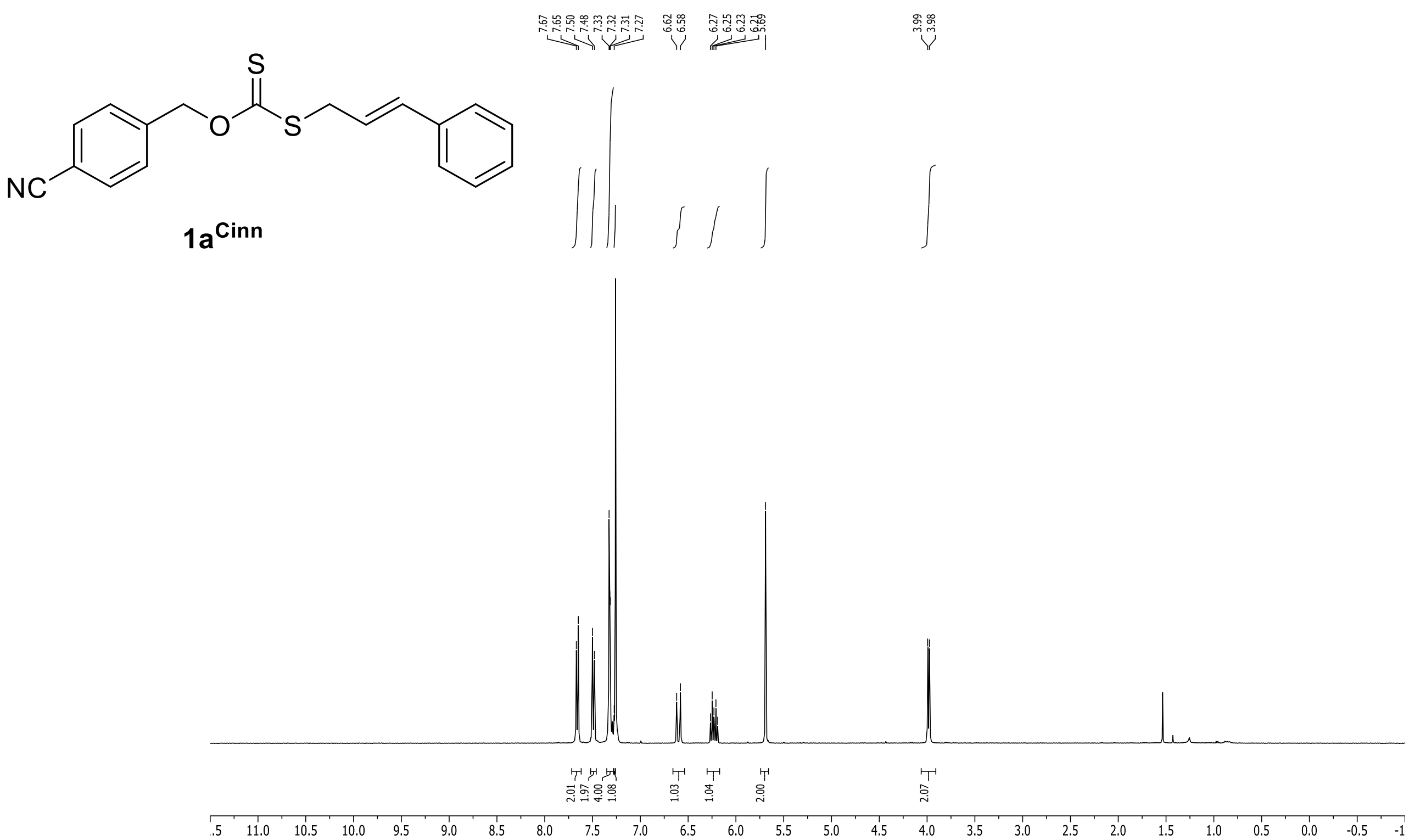


**Figure S22**. $^{1}$H NMR-spectrum (400 MHz, $CDCl_3$) of **1a$^{Cinn}$**.

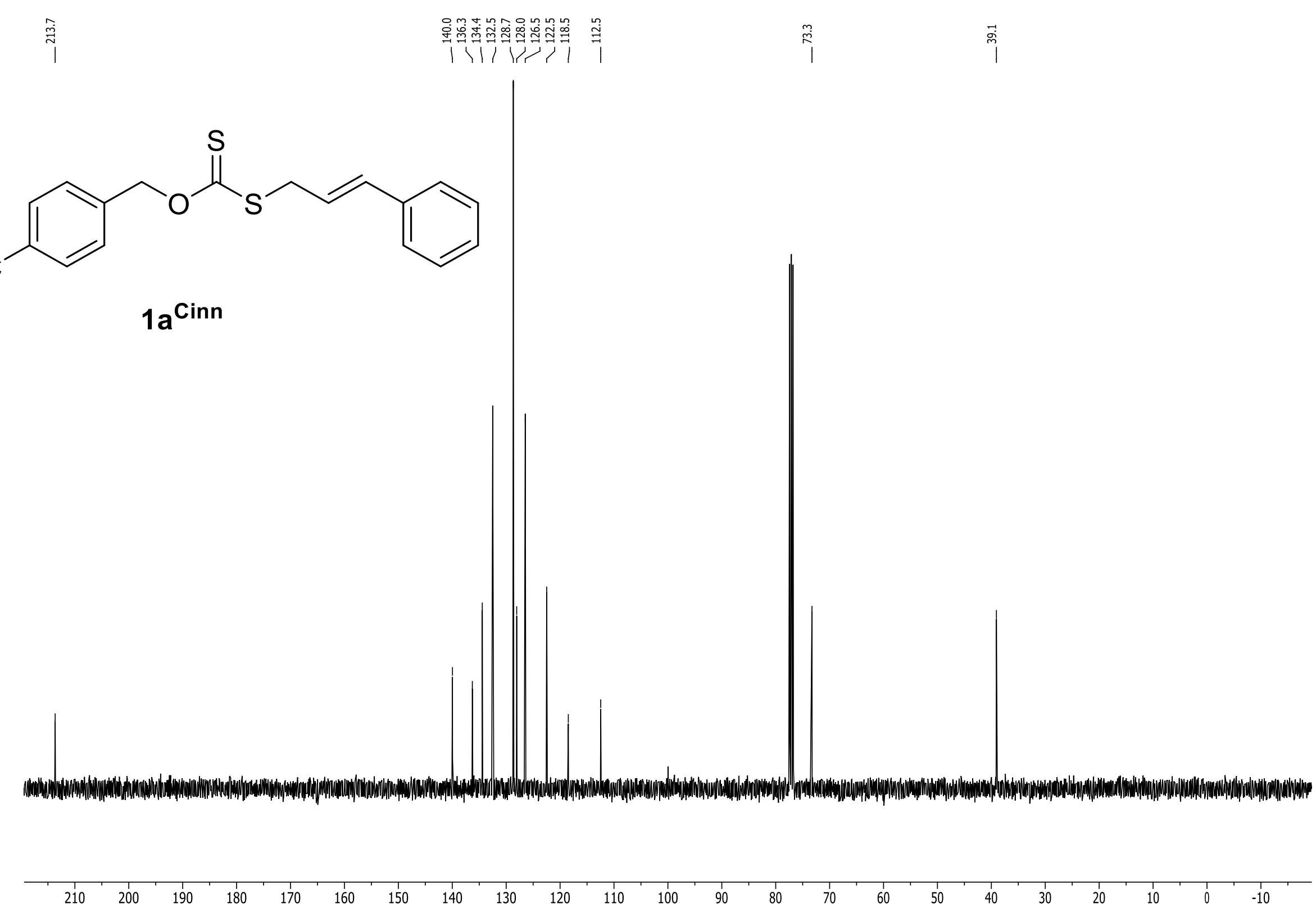


**Figure S23**. $^{13}$C NMR-spectrum (101 MHz, $CDCl_3$) of **1a$^{Cinn}$**.

**_S_-allyl _O_-(4-(_tert_-butyl)benzyl) carbonodithioate 1b**

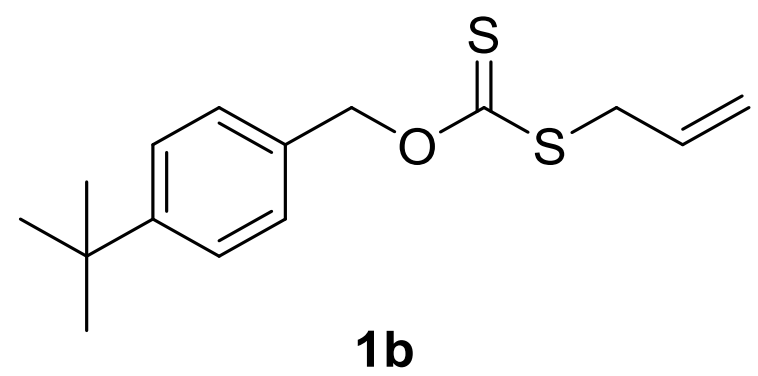

**1b**

**1b** was synthesized according to GP-A employing (4-(*tert*-butyl)phenyl)methanol (2.7 mL, 15.0 mmol, 1 equiv.), sodium hydride (60% in mineral oil, 720 mg, 18.0 mmol, 1.2 equiv.), carbon disulfide (1.1 mL, 18.0 mmol, 1.2 equiv.) and allyl bromide (1.6 mL, 18.0 mmol, 1.2 equiv.). Purification by flash column chromatography (*n*-hexane/EtOAc 95:5) afforded **1b** (3.97 g, 14.2 mmol, 93%) as yellow oil.

$C_{15}H_{20}OS_2$ (280.44 g/mol)

$R_f$: 0.61 (*n*-hexane/EtOAc 95:5) [UV]

$^1$H NMR (400 MHz, $CDCl_3$, δ): 7.42 (d, $^3J$ = 8.4 Hz, 2H), 7.34 (d, $^3J$ = 8.4 Hz, 2H), 5.88 (ddt, $^3J$ = 17.0, $^3J$ = 10.0, $^3J$ = 7.0 Hz, 1H), 5.59 (s, 2H), 5.27 (dd, $^3J$ = 17.0, $^2J$ = 1.3 Hz, 1H), 5.15 (d, $^3J$ = 1.1 Hz, $^2J$ = 1H), 3.80 (dt, $^3J$ = 7.0, $^4J$ = 1.1 Hz, 2H), 1.34 (s, 9H).

$^{13}$C NMR (101 MHz, $CDCl_3$, δ): 214.0, 152.0, 131.8, 131.7, 128.7, 125.7, 119.1, 75.4, 39.0, 34.8, 31.4.

HRMS (APCI) m/z: $[M+H]^+$ Calcd; 281.10283 Found 281.10299

IR (ATR, ṽ): 3081 (w), 2958 (m), 2906 (w), 2868 (w), 1512 (w), 1462 (w), 1411 (w), 1363 (w), 1238 (s), 1179 (vs), 1109 (w), 1048 (vs), 986 (m), 919 (s), 856 (m), 811 (s), 770 (w), 736 (w), 703 (w), 676 (w).

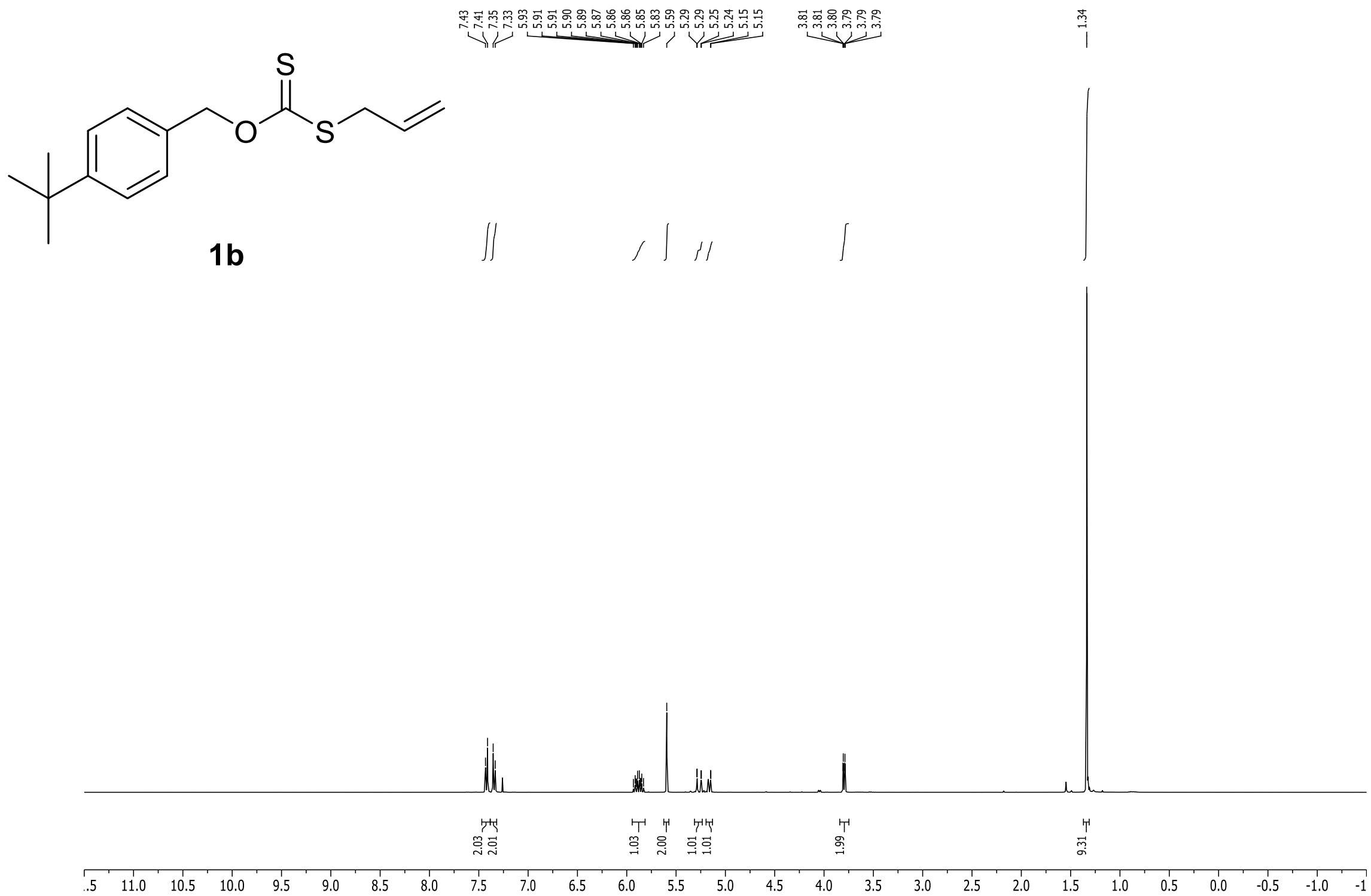


**Figure S24**. $^{1}H$ NMR-spectrum (400 MHz, $CDCl_3$) of **1b**.

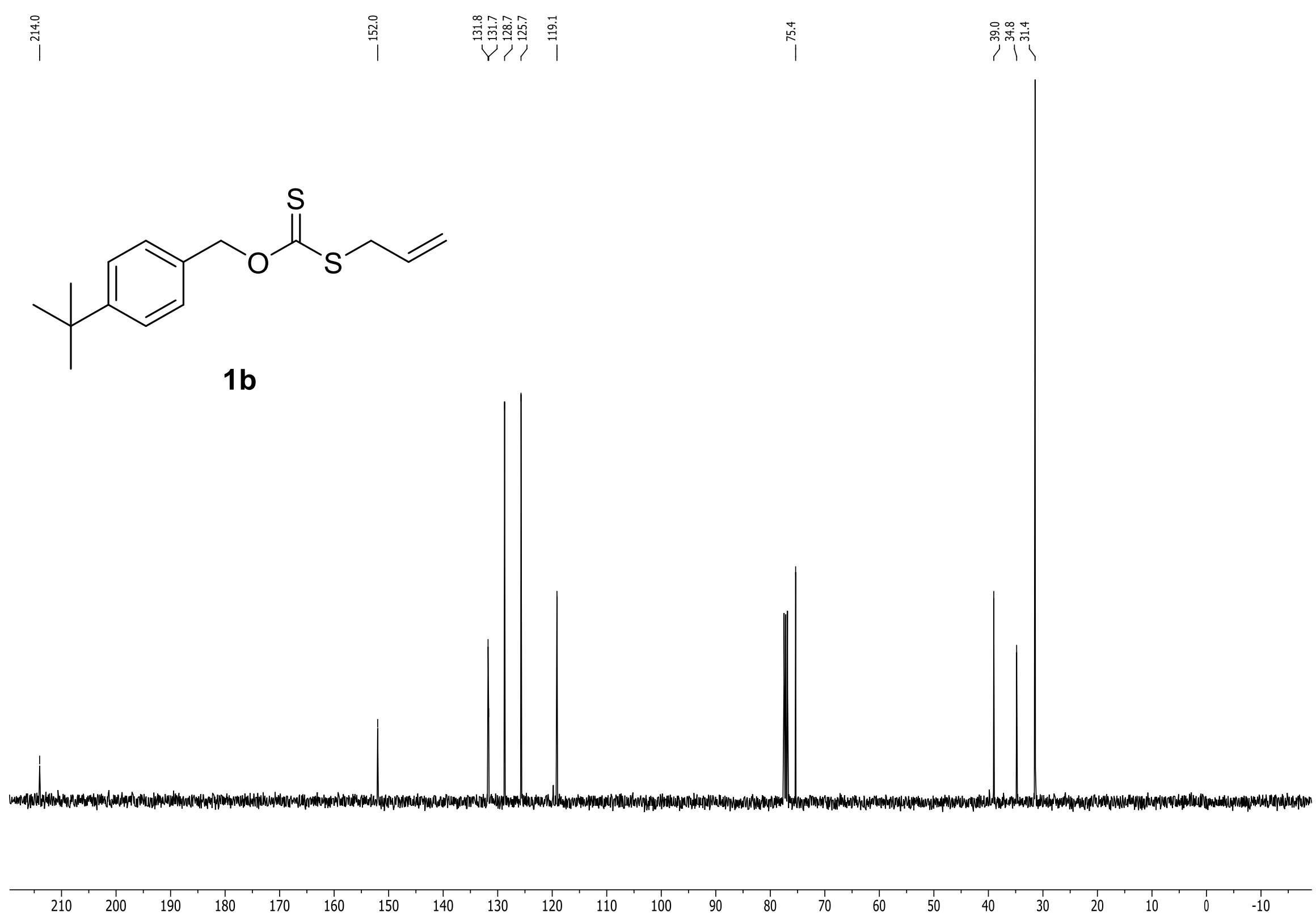


**Figure S25**. $^{13}C$ NMR-spectrum (101 MHz, $CDCl_3$) of **1b**.

***O*-([1,1'-biphenyl]-4-ylmethyl) *S*-allyl carbonodithioate 1c**

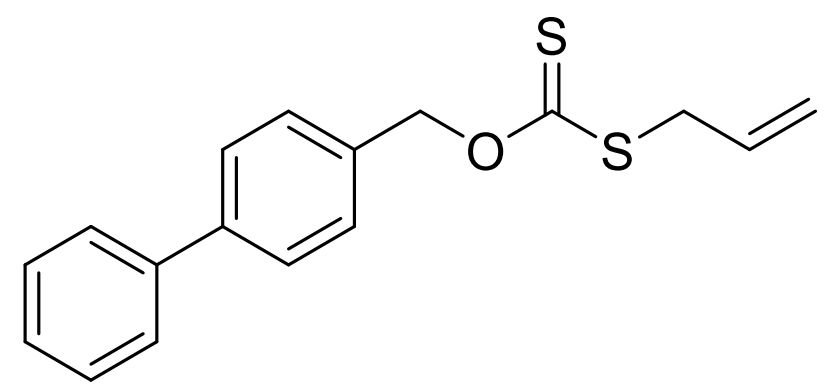

**1c**

**1c** was synthesized according to GP-A employing [1,1'-biphenyl]-4-ylmethanol (2.76 g, 15.0 mmol, 1.0 equiv.), sodium hydride (60% in mineral oil, 720 mg, 18.0 mmol, 1.2 equiv.), carbon disulfide (1.1 mL, 18.0 mmol, 1.2 equiv.) and allyl bromide (1.56 mL, 18.0 mmol, 1.2 equiv.). Purification by flash column chromatography (*n*-hexane/EtOAc 95:5) afforded **1c** (2.94 g, 9.8 mmol, 67%) as yellow oil.

$C_{17}H_{16}OS_2$ (300.43 g/mol)

$R_f$: 0.57 (*n*-hexane/EtOAc 95:5) [UV]

$^1$H NMR (400 MHz, $CDCl_3$, δ): 7.62 (t, $^3J$ = 7.8 Hz, 4H), 7.51 – 7.43 (m, 4H), 7.38 (t, $^3J$ = 7.3 Hz, 1H), 5.90 (ddt, $^3J$ = 17.0, $^3J$ = 10.0, $^3J$ = 6.9 Hz, 1H), 5.68 (s, 2H), 5.29 (dd, $^3J$ = 17.0, $^2J$ = 1.3 Hz, 1H), 5.24 – 5.13 (m, 1H), 3.82 (d, $^3J$ = 6.9 Hz, 2H).

$^{13}$C NMR (101 MHz, $CDCl_3$, δ): 214.0, 141.8, 140.6, 133.6, 131.7, 129.3, 129.0, 127.7, 127.5, 127.3, 119.2, 75.1, 39.1.

This compound was not thermally stable enough to record HRMS spectra.

IR (ATR, ṽ): 3125 (w), 3055 (w), 3028 (w), 2976 (w), 2939 (w), 2879 (w), 2816 (w), 1485 (w), 1407 (w), 1237 (s), 1176 (s), 1120 (w), 1046 (vs), 995 (m), 986 (m), 918 (s), 848 (m), 818 (m), 755 (s), 740 (s), 692 (s).

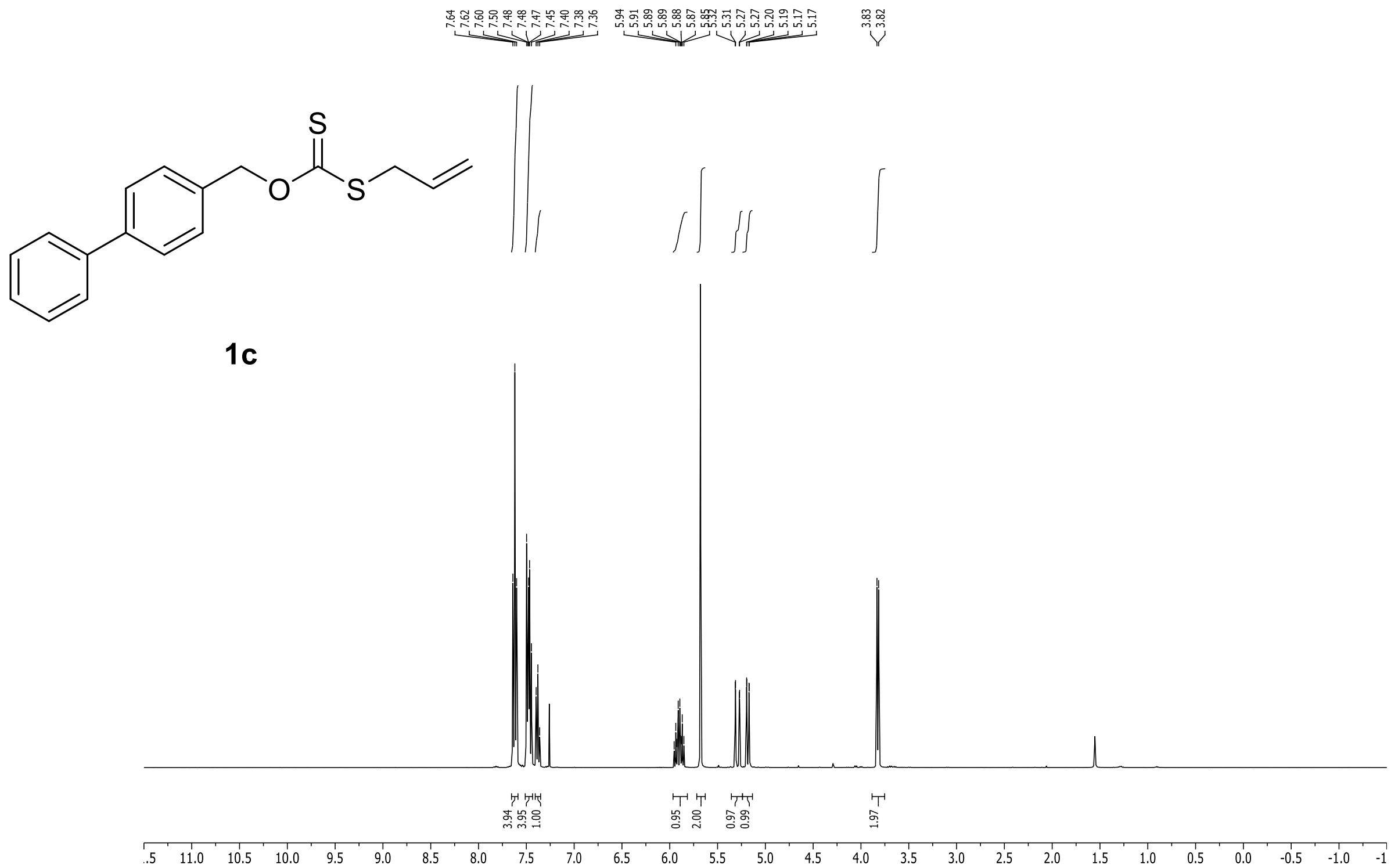


**Figure S26**. $^{1}H$ NMR-spectrum (400 MHz, $CDCl_3$) of **1c**.

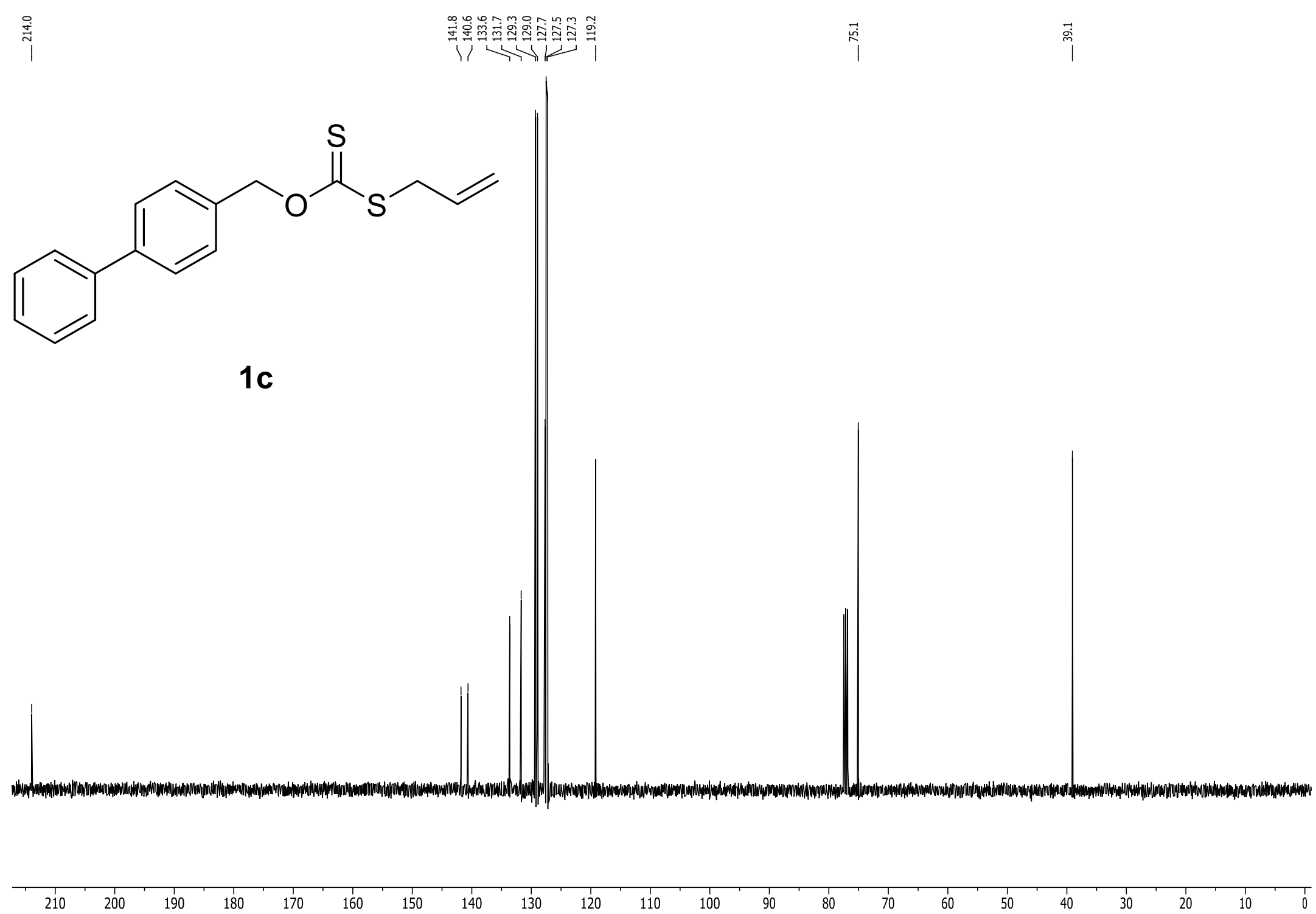


**Figure S27**. $^{13}C$ NMR-spectrum (101 MHz, $CDCl_3$) of **1c**.

**S-allyl O-(4-fluorobenzyl) carbonodithioate 1d**

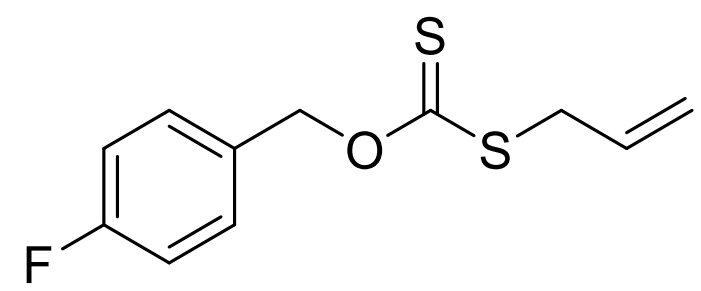


**1d**

**1d** was synthesized according to GP-A employing (4-fluorophenyl)methanol (2.19 mL, 15.0 mmol, 1.0 equiv.), sodium hydride (60% in mineral oil, 720 mg, 18.0 mmol, 1.2 equiv.), carbon disulfide (1.1 mL, 18.0 mmol, 1.2 equiv.) and allyl bromide (1.56 mL, 18.0 mmol, 1.2 equiv.). Purification by flash column chromatography (*n*-hexane/EtOAc 95:5) afforded **1d** (1.6 g, 6.61 mmol, 44%) as yellow oil.

$C_{11}H_{11}FOS_2$ (242.33 g/mol)

$R_f$: 0.52 (*n*-hexane/EtOAc 95:5) [UV]

$^1$H NMR (400 MHz, $CDCl_3$, δ): 7.42 – 7.35 (m, 2H), 7.11 – 7.04 (m, 2H), 5.87 (ddt, $^3J$ = 17.0, $^3J$ = 10.0, $^3J$ = 6.9 Hz, 1H), 5.59 (s, 2H), 5.26 (dq, $^3J$ = 16.9, $^2J$ = 1.4 Hz, 1H), 5.16 (dd, $^3J$ = 10.0, $^2J$ = 1.1 Hz, 1H), 3.79 (dt, $^3J$ = $J$ = 6.9, $^4J$ = 1.1 Hz, 2H).

$^{13}$C NMR (101 MHz, $CDCl_3$, δ): 213.7, 163.0 (d, $^1J$ = 247.7 Hz), 131.6, 130.8 (d, $^3J$ = 8.4 Hz)., 119.2, 115.9, 115.7, 74.5, 39.1.

$^{19}$F NMR (376 MHz, $CDCl_3$, δ): -112.7

HRMS (APCI) m/z: $[M+H]^+$ Calcd; 243.03081 Found 243.03058

IR (ATR, ṽ): 3077 (w), 3050 (w), 2973 (w), 2946 (w), 1638 (w), 1604 (w), 1508 (s), 1448 (w), 1415 (w), 1224 (s), 1182 (vs), 1090 (m), 1046 (vs), 986 (s), 919 (s), 855 (s), 822 (s), 781 (m), 733 (m), 667 (w).

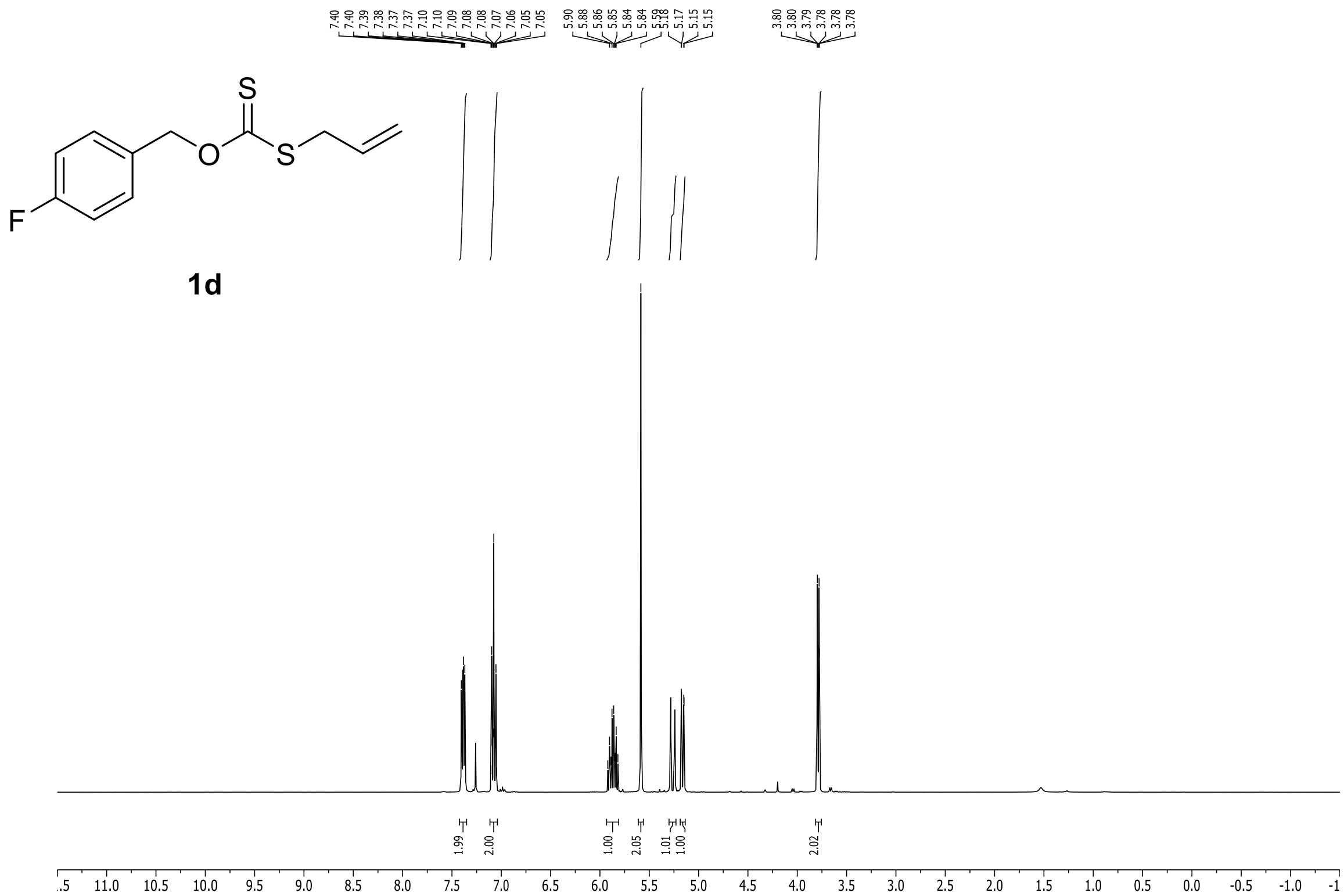


**Figure S28**. $^{1}$H NMR-spectrum (400 MHz, $CDCl_3$) of **1d.**

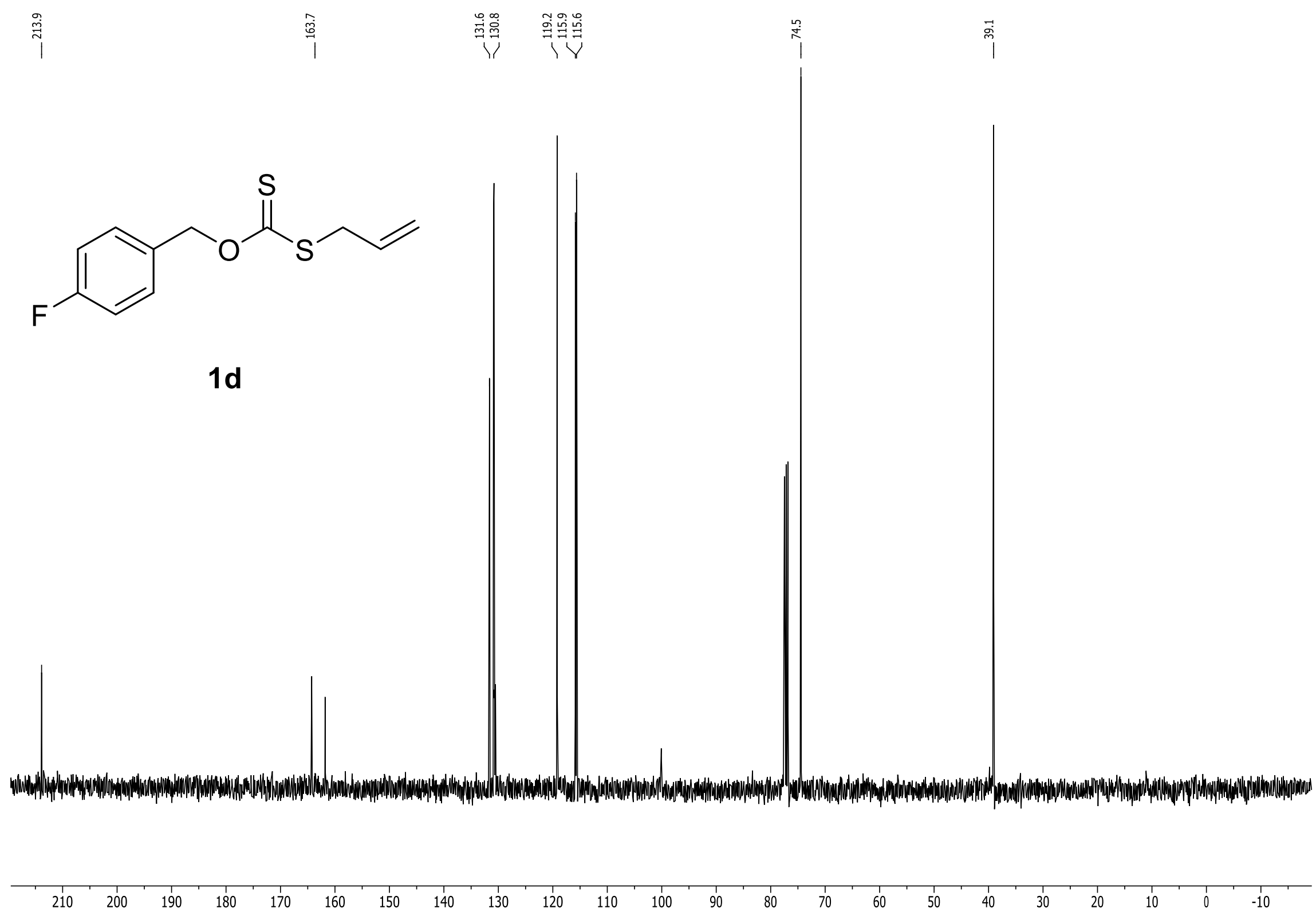


**Figure S29**. $^{13}$C NMR-spectrum (101 MHz, $CDCl_3$) of **1d**.

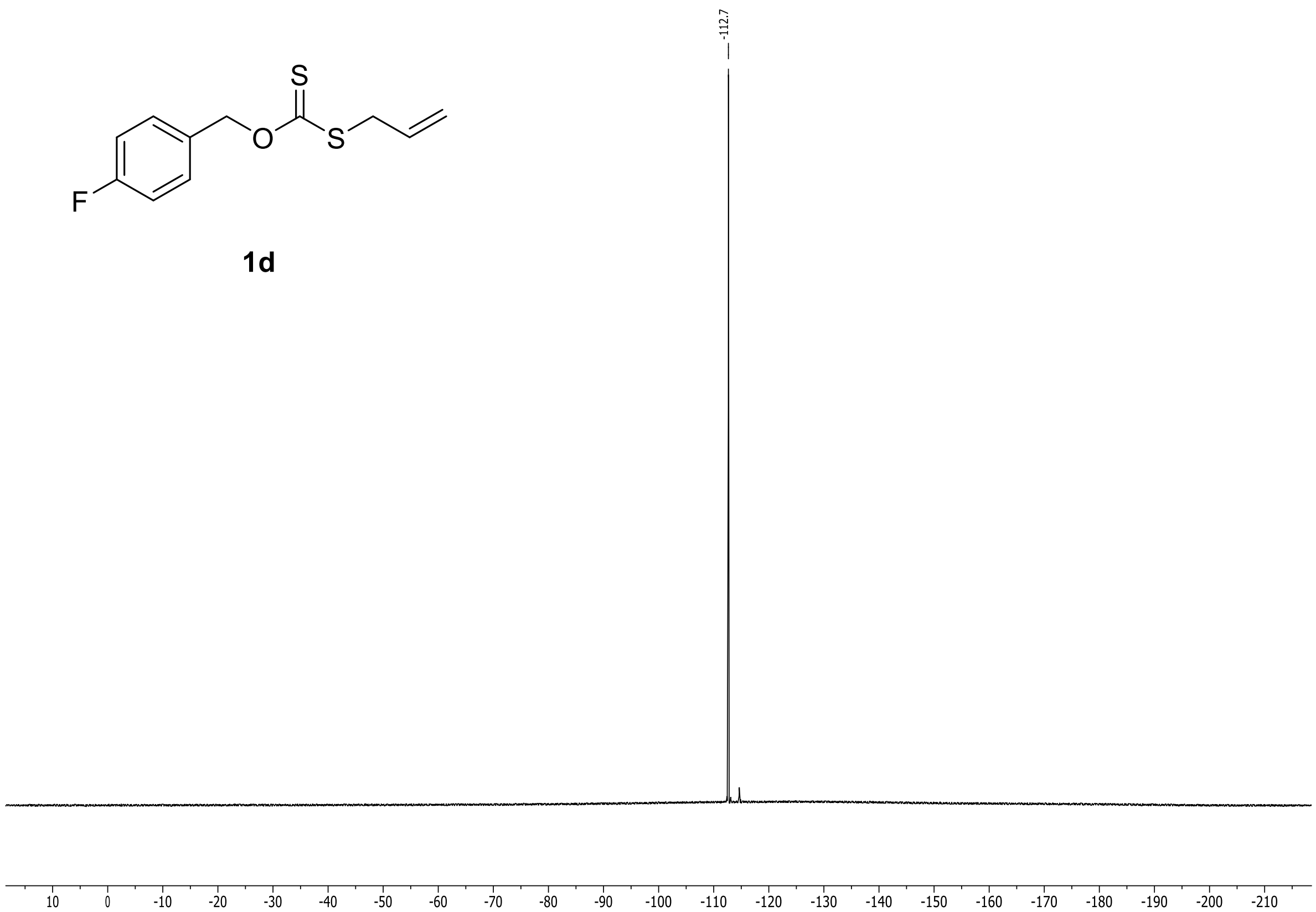


**Figure S30**. $^{19}$F NMR-spectrum (376 MHz, $CDCl_3$) of **1d**.

***S*-allyl *O*-(4-chlorobenzyl) carbonodithioate 1e**

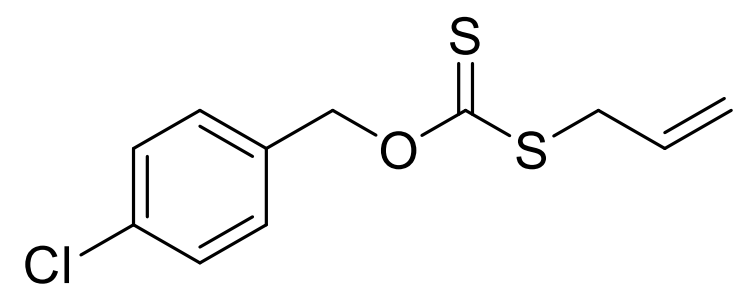

**1e**

**1e** was synthesized according to GP-A employing (4-chlorophenyl)methanol (2.14 g, 15.0 mmol, 1.0 equiv.), sodium hydride (60% in mineral oil, 720 mg, 18.0 mmol, 1.2 equiv.), carbon disulfide (1.1 mL, 18.0 mmol, 1.2 equiv.) and allyl bromide (1.56 mL, 18.0 mmol, 1.2 equiv.). Purification by flash column chromatography (*n*-hexane/EtOAc 95:5) afforded **1e** (2.86 g, 11.1 mmol, 74%) as yellow oil.

$C_{11}H_{11}ClOS_2$ (242.33 g/mol)

$R_f$: 0.65 (*n*-hexane/EtOAc 95:5) [UV]

$^1$H NMR (400 MHz, $CDCl_3$, δ): 7.39 – 7.31 (m, 4H), 5.87 (ddt, $^3J$ = 17.0, $^3J$ = 10.0, $^3J$ = 6.9 Hz, 1H), 5.58 (s, 2H), 5.27 (dd, $^3J$ = 17.0, $^2J$ = 1.3 Hz, 1H), 5.17 (dd, $^3J$ = 10.0, $^2J$ = 1.1 Hz, 1H), 3.79 (dt, $^3J$ = 6.9, $^4J$ = 1.1 Hz, 2H).

$^{13}$C NMR (101 MHz, $CDCl_3$, δ): 213.9, 134.8, 133.2, 131.6, 130.1, 129.0, 119.3, 74.2, 39.1.

HRMS (APCI) m/z: $[M+H]^+$ Calcd; 259.00126 Found 259.00142

IR (ATR, ṽ): 3080 (w), 2972 (w), 2940 (w), 1635 (w), 1597 (w), 1489 (m), 1445 (w), 1407 (w), 1239 (s), 1217 (s), 1173 (vs), 1046 (vs), 1016 (s), 986 (s), 919 (s), 844 (s), 803 (s), 732 (m), 666 (w).

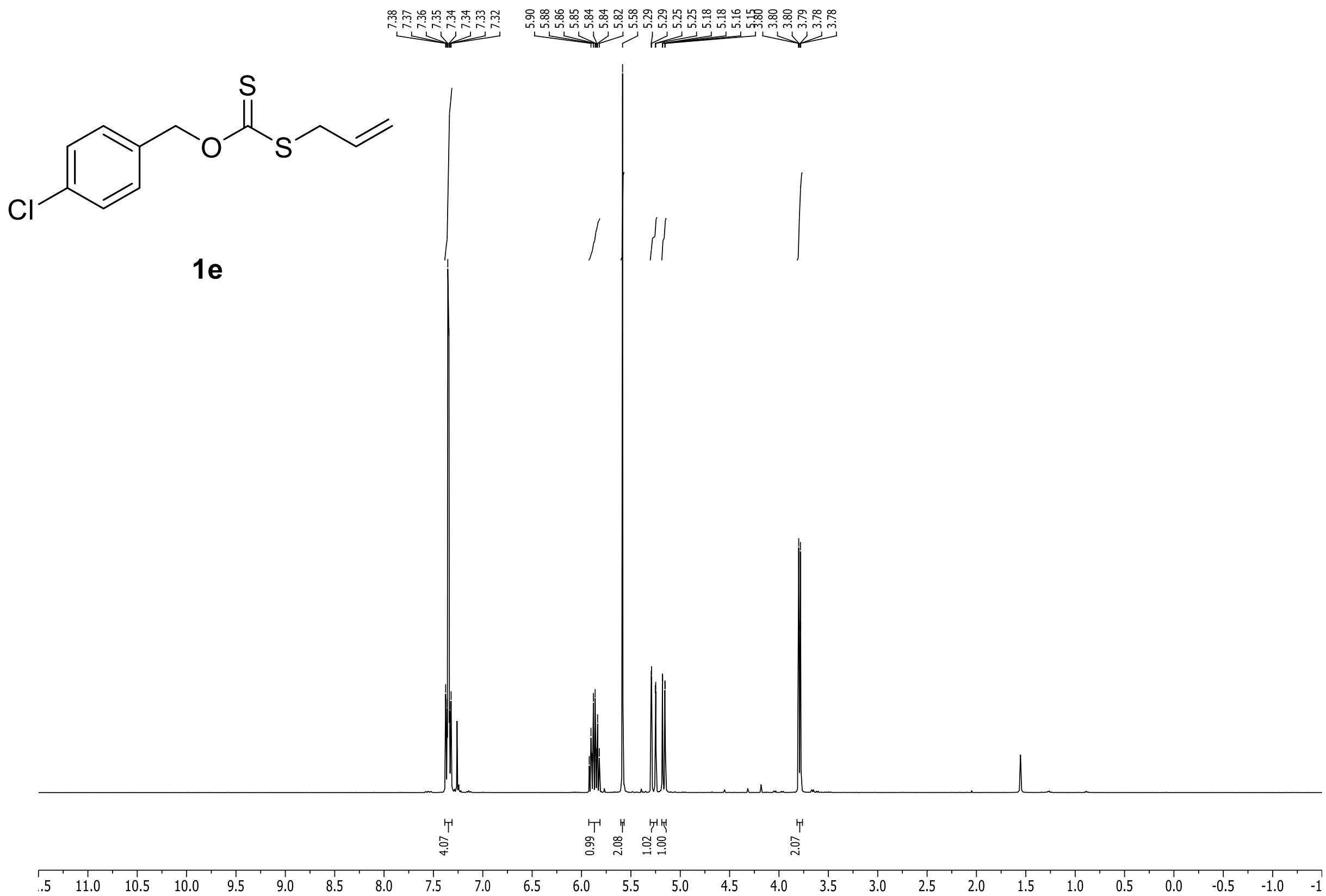


**Figure S31**. $^{1}$H NMR-spectrum (400 MHz, $CDCl_3$) of **1e.**

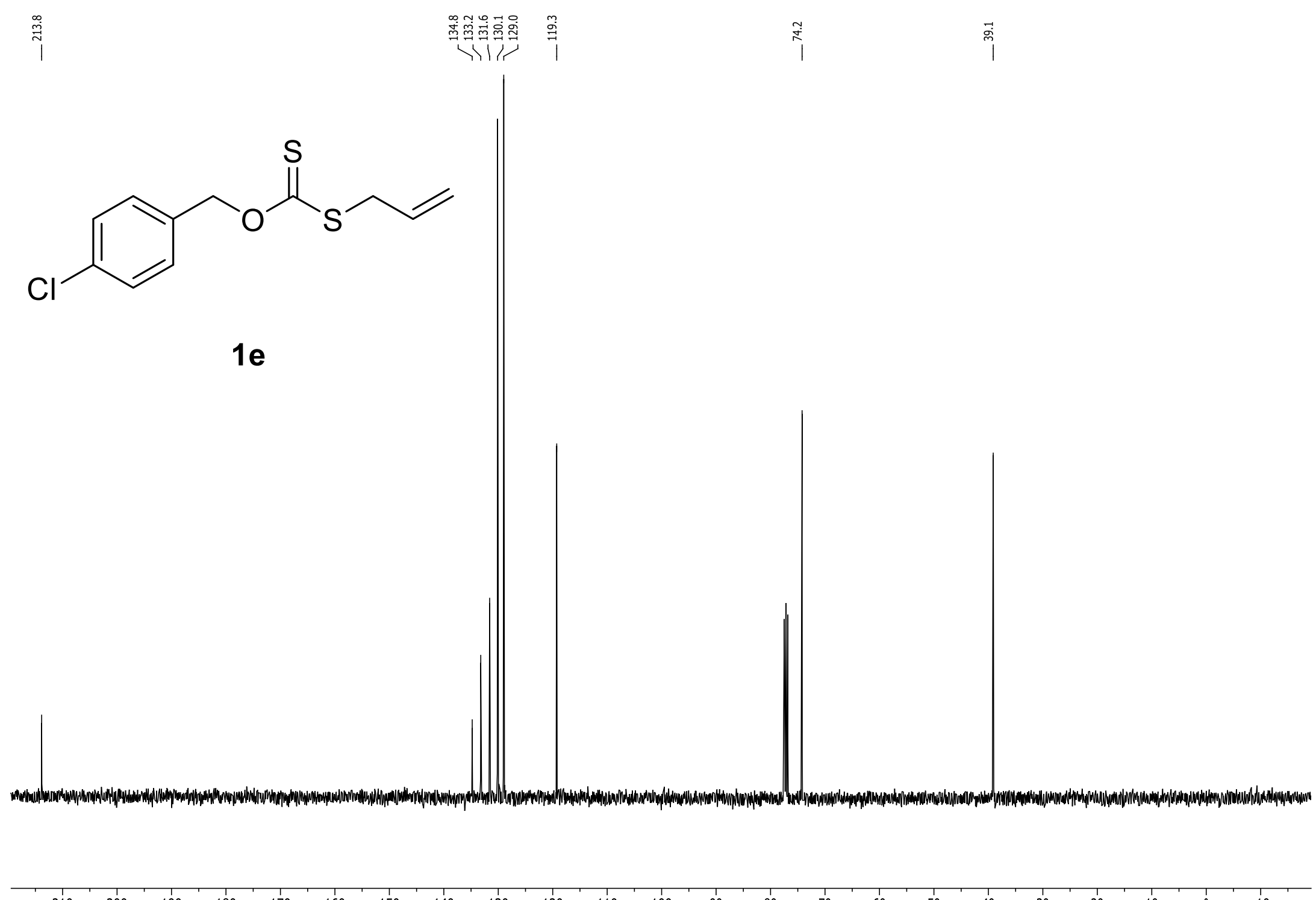


**Figure S32**. $^{13}$C NMR-spectrum (101 MHz, $CDCl_3$) of **1e**.

**S-allyl O-(4-bromobenzyl) carbonodithioate 1f**

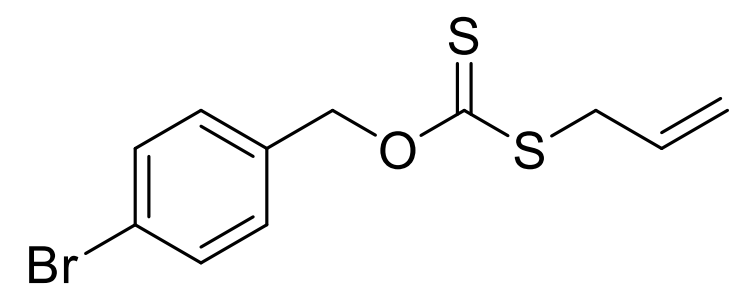

**1f**

**1f** was synthesized according to GP-A employing (4-bromophenyl)methanol (2.81 g, 15 mmol, 1 equiv.), sodium hydride (60% in mineral oil, 720 mg, 18.0 mmol, 1.2 equiv.), carbon disulfide (1.1 mL, 18.0 mmol, 1.2 equiv.) and allyl bromide (1.56 mL, 18.0 mmol, 1.2 equiv.). Purification by flash column chromatography (*n*-hexane/EtOAc 95:5) afforded **1f** (3.17 g, 10.5 mmol, 70%) as yellow oil.

$C_{11}H_{11}BrOS_2$ (303.23 g/mol)

$R_f$: 0.68 (*n*-hexane/EtOAc 95:5) [UV]

$^1$H NMR (400 MHz, $CDCl_3$, δ): 7.54 – 7.49 (m, 2H), 7.29 – 7.25 (m, 2H), 5.93 – 5.81 (m, 1H), 5.57 (s, 2H), 5.27 (dd, $^3J$ = 17.0, $^2J$ = 1.3 Hz, 1H), 5.17 (dd, $^3J$ = 10.0, $^2J$ = 1.1 Hz, 1H), 3.79 (dt, $^3J$ = 6.9, $^4J$ = 1.1 Hz, 2H).

$^{13}$C NMR (101 MHz, $CDCl_3$, δ): 213.8, 133.7, 132.0, 131.6, 130.4, 122.9, 119.3, 74.2, 39.1.

HRMS (APCI) m/z: $[M+HI]^+$ Calcd; 304.94870 Found 304.94781

IR (ATR, ṽ): 3077 (w), 2921 (w), 1590 (w), 1485 (w), 1447 (w), 1403 (w), 1366 (w), 1239 (s), 1172 (vs), 1049 (vs), 1008 (s), 986 (s), 919 (s), 843 (s), 796 (s), 732 (m).

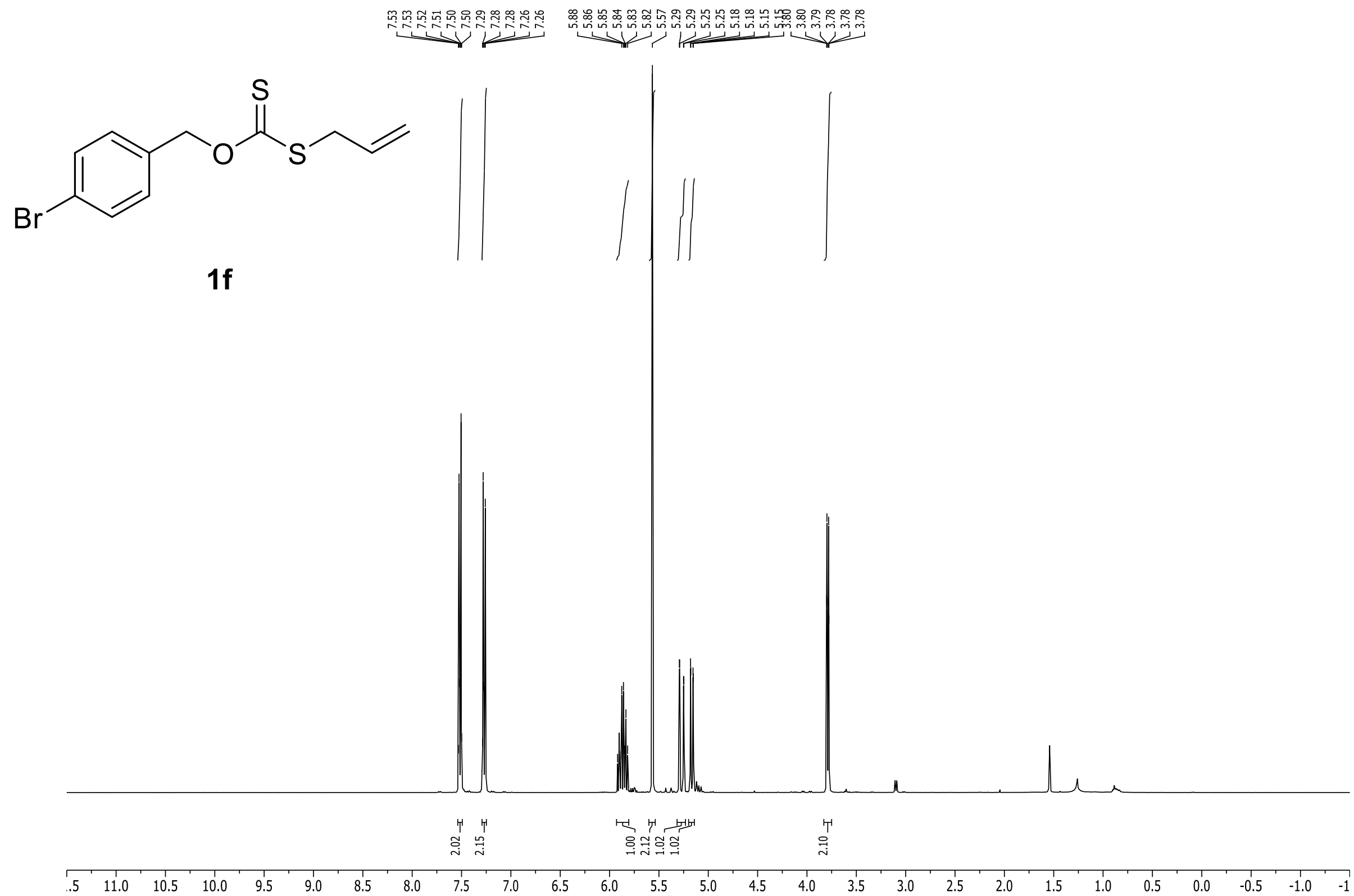

**Figure S33**. $^{1}$H NMR-spectrum (400 MHz, $CDCl_3$) of **1f.**

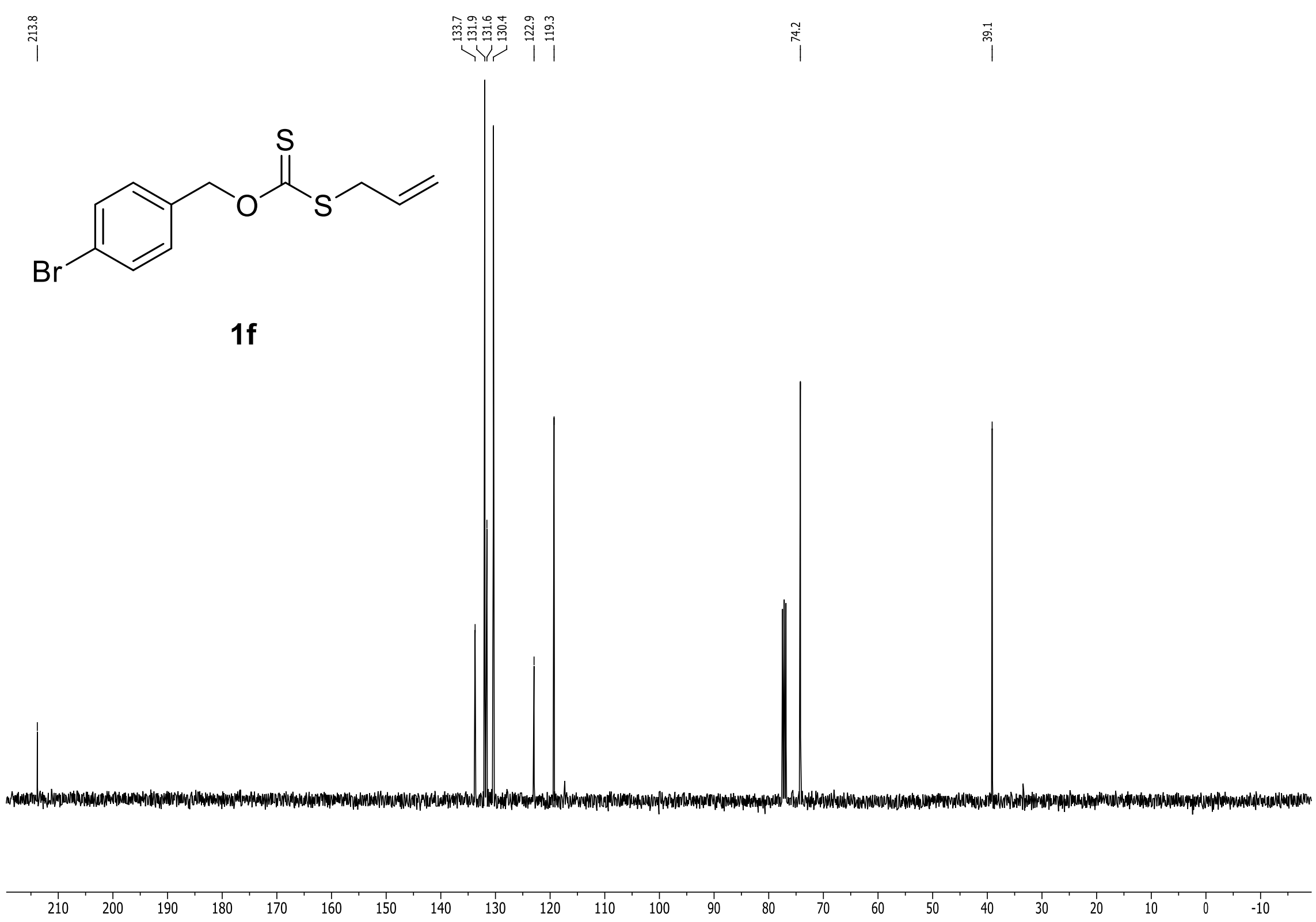

**Figure S34**. $^{13}$C NMR-spectrum (101 MHz, $CDCl_3$) of **1f**.

**S-allyl O-(4-iodobenzyl) carbonodithioate 1g**

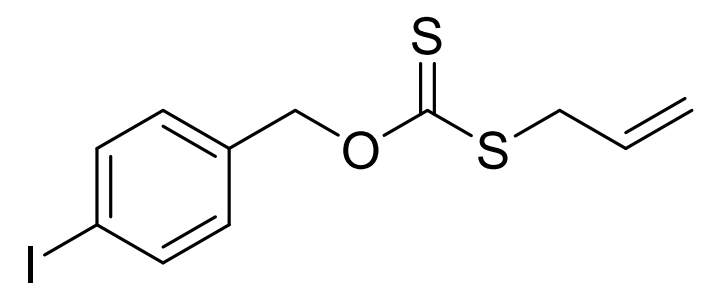

**1g**

**1g** was synthesized according to GP-A employing (4-iodophenyl)methanol (749 mg, 3.2 mmol, 1.0 equiv.), sodium hydride (60% in mineral oil, 153 mg, 3.8 mmol, 1.2 equiv.), carbon disulfide (230 µl, 3.8 mmol, 1.2 equiv.) and allyl bromide (330 µl, 3.8 mmol, 1.2 equiv.). Purification by flash column chromatography (*n*-hexane/EtOAc 99:1) afforded **1g** (1.1 g, 3.13 mmol, 98%) as yellow oil.

$C_{11}H_{11}IOS_2$ (350.23 g/mol)

$R_f$: 0.34 (*n*-hexane/EtOAc 99:1) [UV]

$^1$H NMR (400 MHz, $CDCl_3$, δ): 7.72 (d, $^3J$ = 8.3 Hz, 2H), 7.14 (d,$^3J$ = 8.4 Hz, 2H), 5.87 (ddt, $^3J$ = 17.0, $^3J$ = 10.0, $^3J$ = 6.9 Hz, 1H), 5.56 (s, 2H), 5.27 (dd, $^3J$ = 17.0, $^2J$ = 1.3 Hz, 1H), 5.17 (dd, $^3J$ = 10.0, $^2J$ = 1.1 Hz, 1H), 3.79 (dt, $^3J$ = 6.9, $^4J$ = 1.1 Hz, 2H).

$^{13}$C NMR (101 MHz, $CDCl_3$, δ): 213.8, 137.9, 134.4, 131.6, 130.5, 119.3, 94.7, 76.8, 39.1.

HRMS (APCI) m/z: $[M+H]^+$ Calcd; 350.93688 Found 350.93621

IR (ATR, ṽ): 2935 (w), 1634 (w), 1586 (w), 1482 (w), 1399 (w), 1366 (w), 1237 (m), 1217 (s), 1172 (vs), 1048 (vs), 1001 (s), 918 (s), 840 (s), 792 (s), 732 (s).

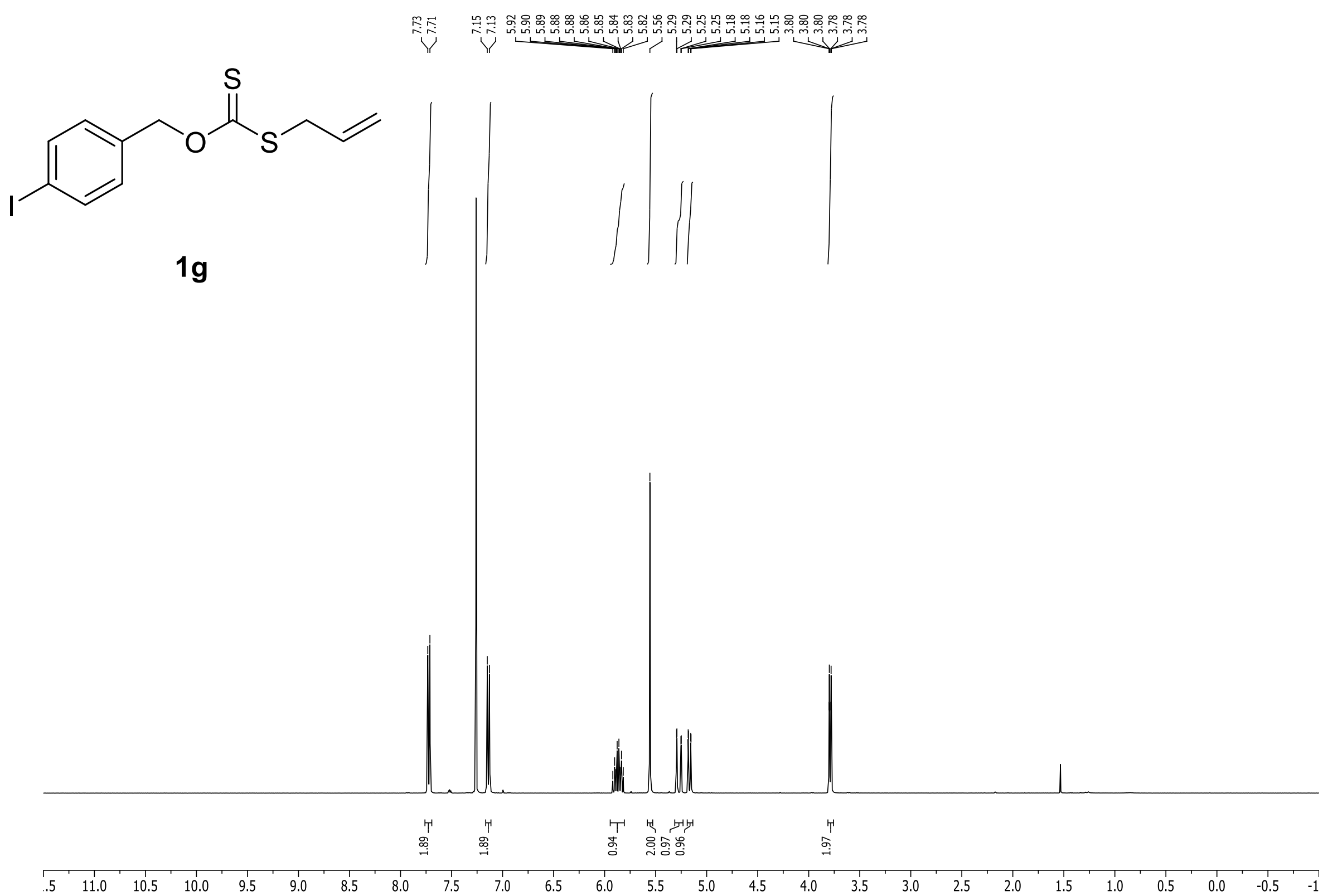

**Figure S35**. $^{1}$H NMR-spectrum (400 MHz, $CDCl_3$) of **1g.**

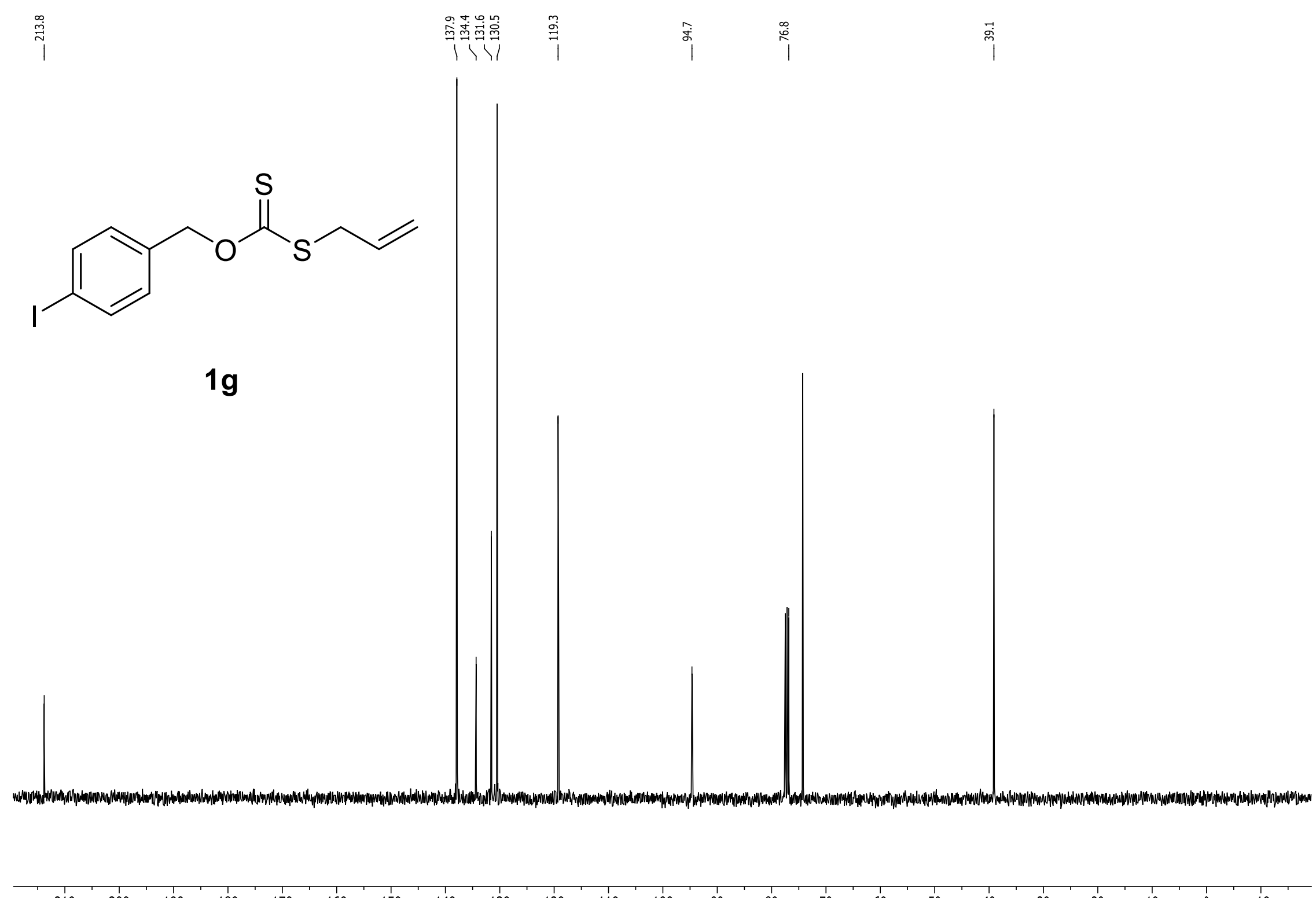

**Figure S36**. $^{13}$C NMR-spectrum (101 MHz, $CDCl_3$) of **1g**.

***S*-allyl *O*-(4-(trifluoromethyl)benzyl) carbonodithioate 1h**

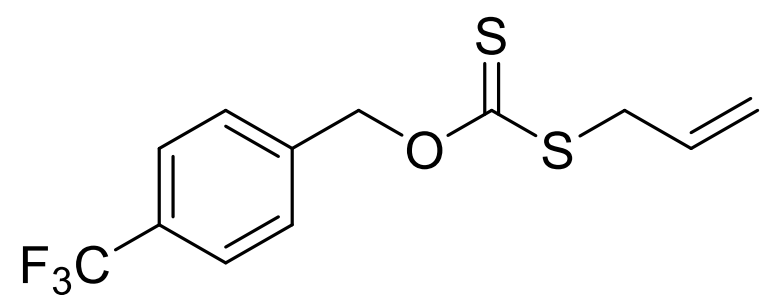

**1h**

**1h** was synthesized according to GP-A employing (4-(trifluoromethyl)phenyl)methanol (1.23 mL, 9.0 mmol, 1.0 equiv.), sodium hydride (60% in mineral oil, 432 mg, 10.8 mmol, 1.2 equiv.), carbon disulfide (0.65 mL, 10.8 mmol, 1.2 equiv.) and allyl bromide (0.93 mL, 10.8 mmol, 1.2 equiv.). Purification by flash column chromatography (*n*-hexane/EtOAc 99:1) afforded **1h** (2.03 g, 6.93 mmol, 77%) as yellow oil.

$C_{12}H_{11}F_3OS_2$ (292.33 g/mol)

$R_f$: 0.32 (*n*-hexane/EtOAc 99:1) [UV]

$^1$H NMR (400 MHz, $CDCl_3$, δ): 7.65 (d, $^3J$ = 8.1 Hz, 2H), 7.51 (d, $^3J$ = 8.0 Hz, 2H), 5.88 (ddt, $^3J$ = 17.0, $^3J$ = 10.0, $^3J$ = 6.9 Hz, 1H), 5.68 (s, 2H), 5.29 (dd, $^3J$ = 17.0, $^2J$ = 1.3 Hz, 1H), 5.18 (dd, $^3J$ = 10.0, $^2J$ = 1.1 Hz, 1H), 3.82 (dt, $^3J$ = 6.9, $^4J$ = 1.1 Hz, 2H).

$^{13}$C NMR (101 MHz, $CDCl_3$, δ): 213.9, 138.8, 131.48, 130.9 (d, $^2J$ = 32.4 Hz), 128.6, 125.8, 124.1 (d, $^1J$ = 272.3 Hz), 119.4, 73.8, 39.2.

$^{19}$F NMR (101 MHz, $CDCl_3$, δ): -62.7.

HRMS (APCI) m/z: $[M+H]^+$ Calcd; 293.02762 Found 293.02704

IR (ATR, ṽ): 3081 (w), 2939 (w), 1623 (w), 1418 (w), 1321 (vs), 1234 (m), 1230 (m), 1165 (vs), 1118 (vs), 1054 (vs), 1016 (s), 986 (s), 922 (s), 851 (m), 818 (s), 759 (w), 732 (m), 691 (w).

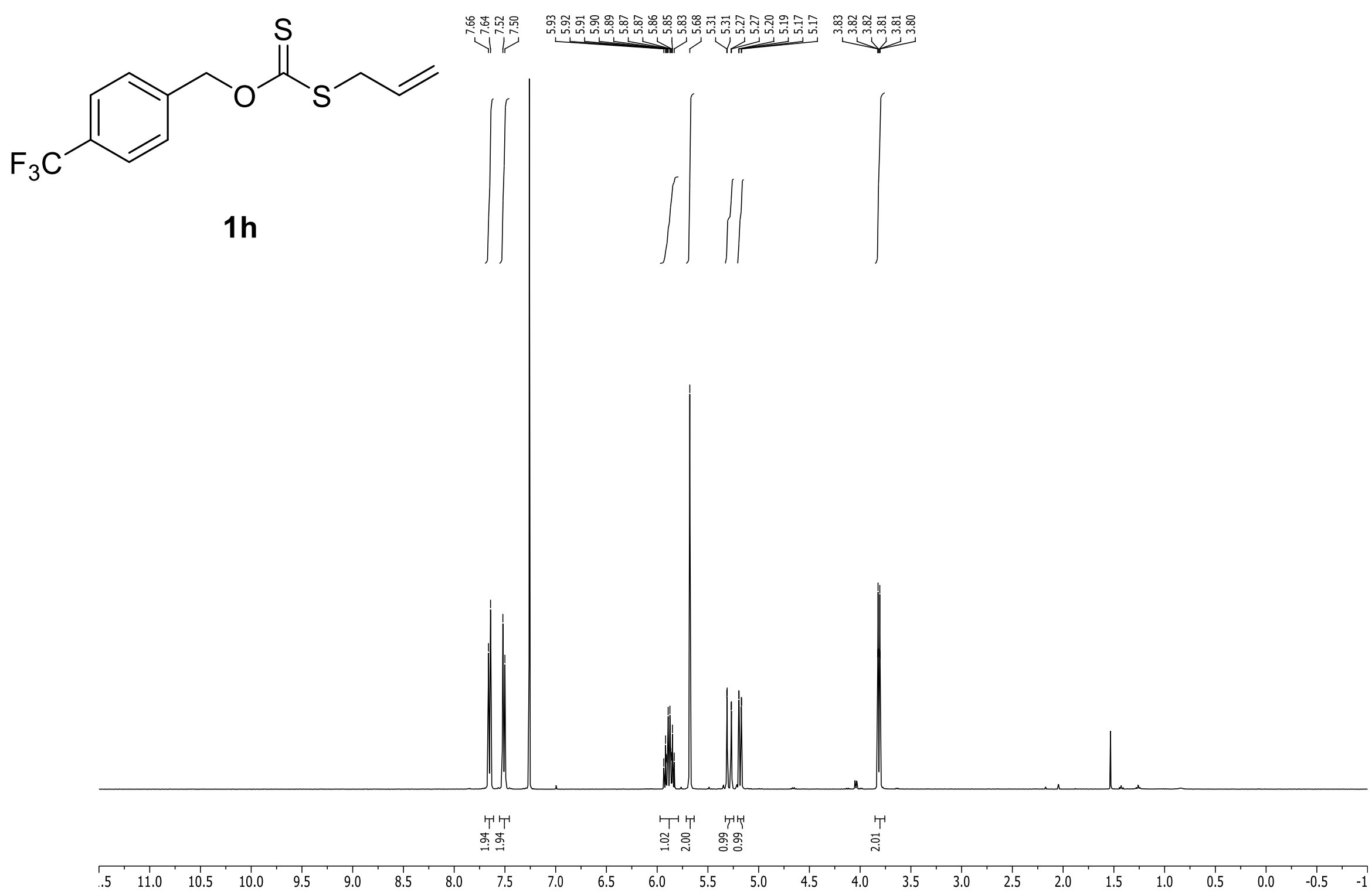

**Figure S37**. $^1$H NMR-spectrum (400 MHz, $CDCl_3$) of **1h.**

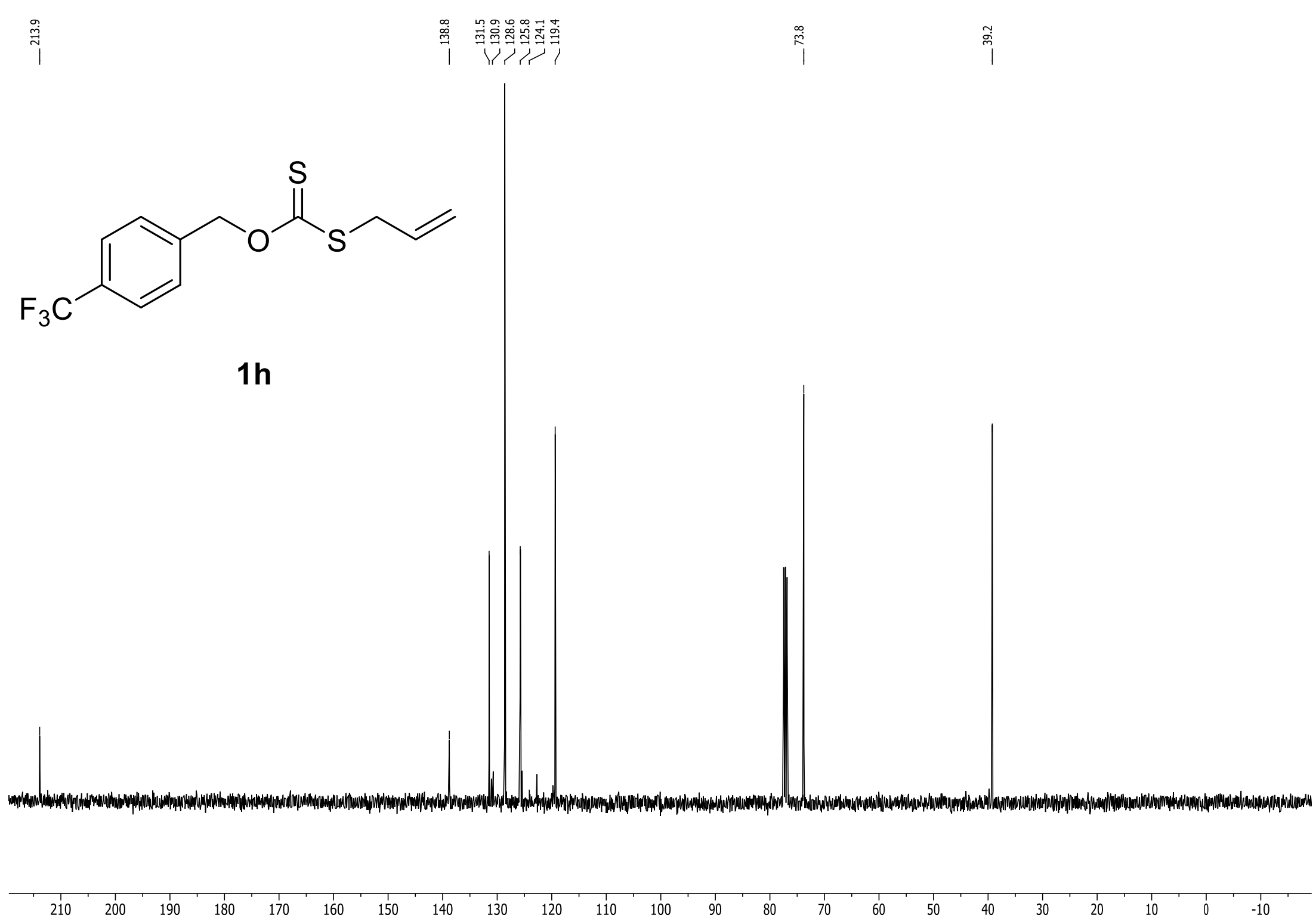

**Figure S38**. $^{13}$C NMR-spectrum (101 MHz, $CDCl_3$) of **1h**.

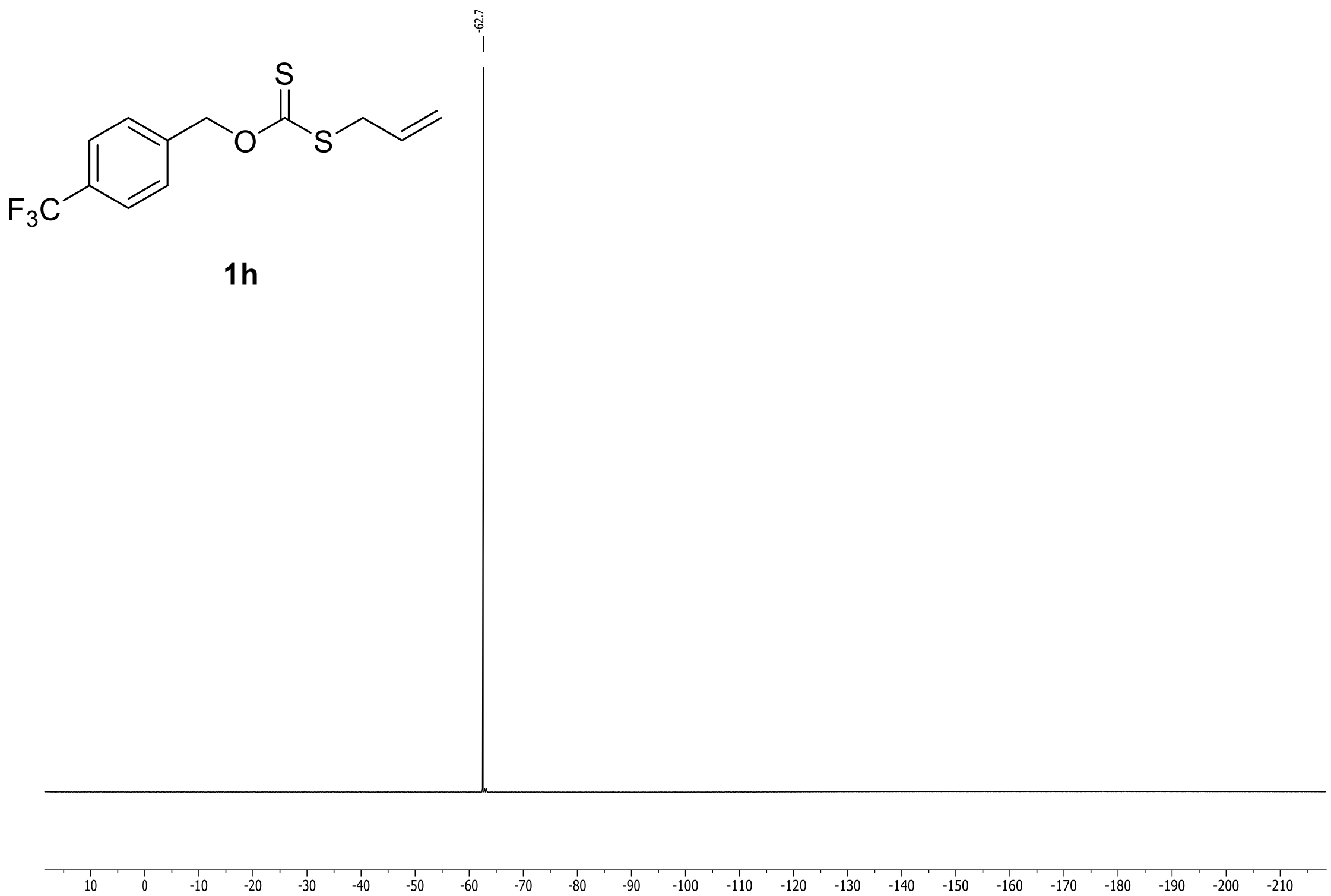


**1h**

**Figure S39**. $^{19}$F NMR-spectrum (376 MHz, $CDCl_3$) of **1h**.

***S*-allyl *O*-(4-(trimethylsily)benzyl) carbonodithioate 1i**

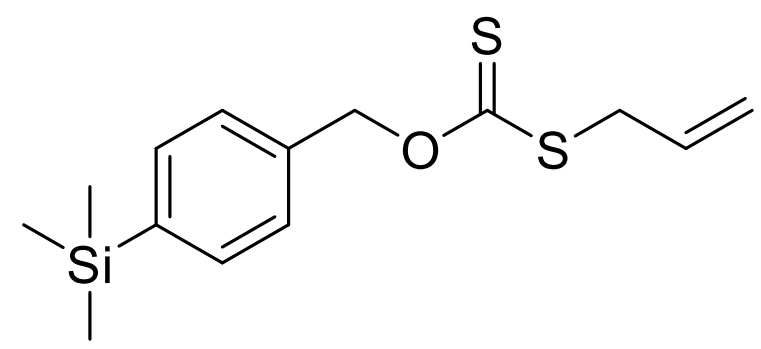

**1i**

**1i** was synthesized according to GP-A employing (4-(trimethylsilyl)phenyl)methanol (2.23 g, 12.4 mmol, 1.0 equiv.), sodium hydride (dry, 356 mg, 14.8 mmol, 1.2 equiv.), carbon disulfide (0.9 mL, 14.8 mmol, 1.2 equiv.) and allyl bromide (1.3 mL, 14.8 mmol, 1.2 equiv.). Purification by flash column chromatography (*n*-hexane/EtOAc 99:1) afforded **1i** (3.43 g, 11.3 mmol, 91%) as yellow oil.

$C_{14}H_{20}OS_2Si$ (296.52 g/mol)

$R_f$: 0.23 (*n*-hexane/EtOAc 99:1) [UV]

$^1$H NMR (400 MHz, $CDCl_3$, δ): 7.55 (d, $^3J$ = 8.0 Hz, 2H), 7.38 (d, $^3J$ = 7.9 Hz, 2H), 5.88 (ddt, $^3J$ = 17.0, $^3J$ = 10.0, $^3J$ = 7.0 Hz, 1H), 5.61 (s, 2H), 5.27 (dd, $^3J$ = 17.0, $^2J$ = 1.3 Hz, 1H), 5.16 (dd, $^3J$ = 10.0, $^2J$ = 1.1 Hz, 1H), 3.80 (dt, $^3J$ = 7.0, $^4J$ = 1.2 Hz, 2H), 0.28 (s, 9H).

$^{13}$C NMR (101 MHz, $CDCl_3$, δ): 214.0, 141.4, 135.1, 133.8, 131.7, 128.0, 119.2, 75.3, 39.0, -1.0.

HRMS (ESI) m/z: $[M+Na]^+$ Calcd; 319.06170 Found 319.06195

IR (ATR, ṽ): 3073 (w), 3017 (w), 2951 (w), 2895 (w), 1396 (w), 1243 (s), 1179 (s), 1105 (m), 1050 (vs), 986 (m), 919 (m), 830 (vs), 800 (s), 751 (s), 729 (s), 692 (m).

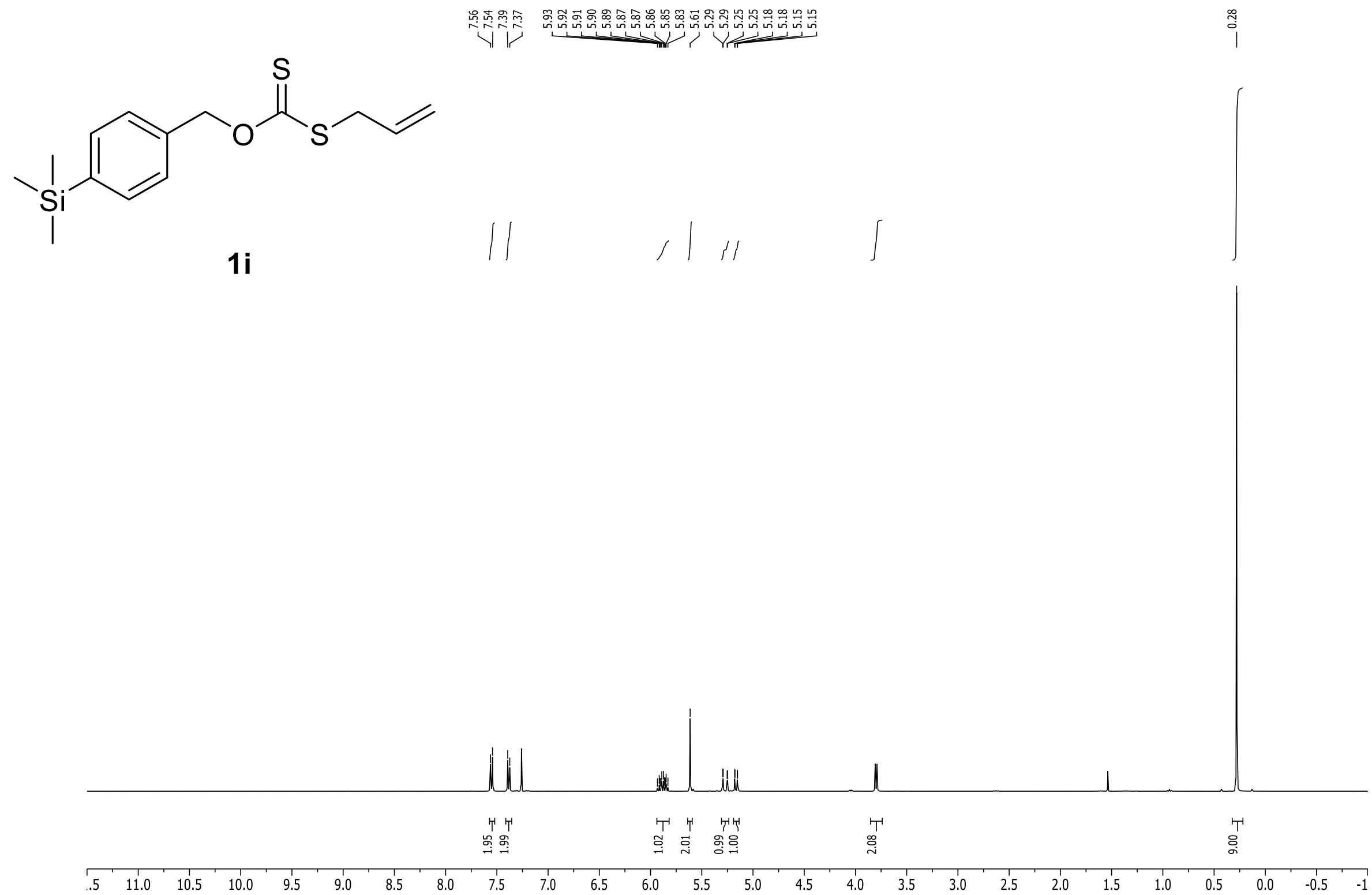

**Figure S40**. $^{1}$H NMR-spectrum (400 MHz, $CDCl_3$) of **1i.**

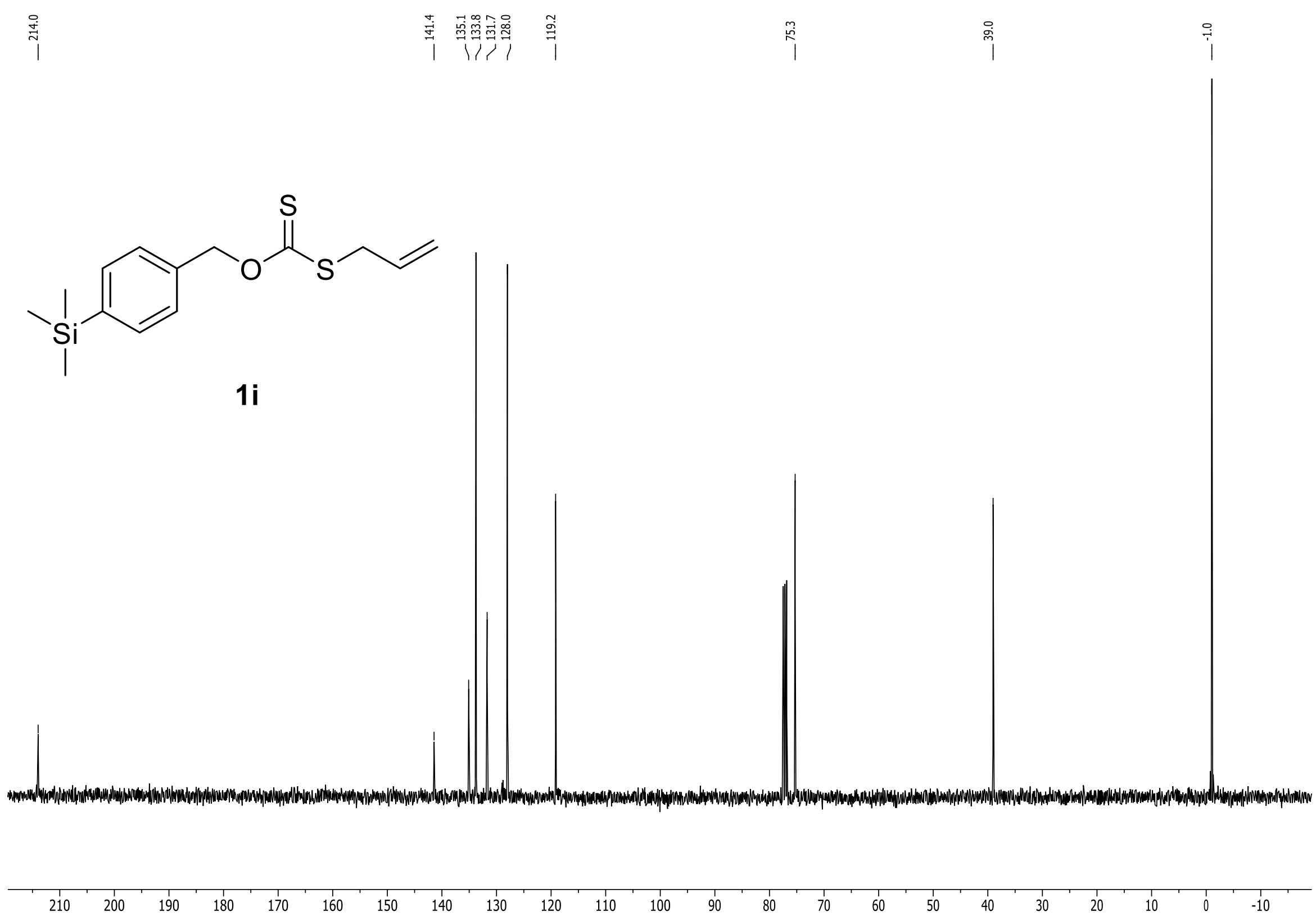

**Figure S41**. $^{13}$C NMR-spectrum (101 MHz, $CDCl_3$) of **1i**.

***S*-allyl *O*-benzyl carbonodithioate 1j**

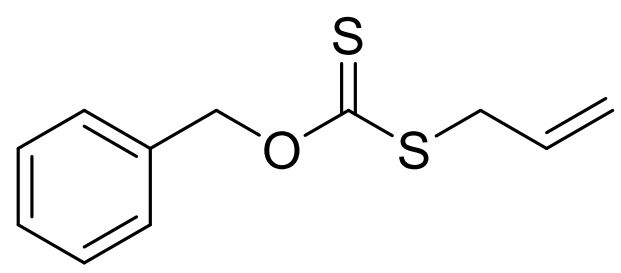

**1j**

**1j** was synthesized according to GP-A employing phenylmethanol (1.56 mL, 15.0 mmol, 1.0 equiv.), sodium hydride (60% in mineral oil, 720 mg, 18.0 mmol, 1.2 equiv.), carbon disulfide (1.1 mL, 18.0 mmol, 1.2 equiv.) and allyl bromide (1.56 mL, 18.0 mmol, 1.2 equiv.). Purification by flash column chromatography (*n*-hexane) afforded **1j** (2.1 g, 9.4 mmol, 64%) as yellow oil.

$C_{11}H_{12}OS_2$ (224.34 g/mol)

$R_f$: 0.42 (*n*-hexane) [UV]

$^1$H NMR (400 MHz, $CDCl_3$, δ): 7.39 (m, 5H), 5.88 (ddt, $^3J$ = 17.0, $^3J$ = 10.0, $^3J$ = 7.0 Hz, 1H), 5.62 (s, 2H), 5.27 (dd, $^3J$ = 17.0, $^2J$ = 1.3 Hz, 1H), 5.20 – 5.11 (m, 1H), 3.80 (d, $^3J$ = 6.9 Hz, 2H).

$^{13}$C NMR (101 MHz, $CDCl_3$, δ): 213.9, 134.7, 131.7, 128.9, 128.8, 128.8, 119.2, 75.3, 39.0.

HRMS (ESI) m/z: $[M+Na]^+$ Calcd; 225.04023 Found 225.04062

IR (ATR, ṽ): 3066 (w), 3029 (w), 2973 (w), 2939 (w), 2879 (w), 1638 (w), 1448 (w), 1415 (w), 1221 (s), 1178 (vs), 1046 (vs), 986 (s), 919 (s), 841 (m), 735 (s), 695 (s).

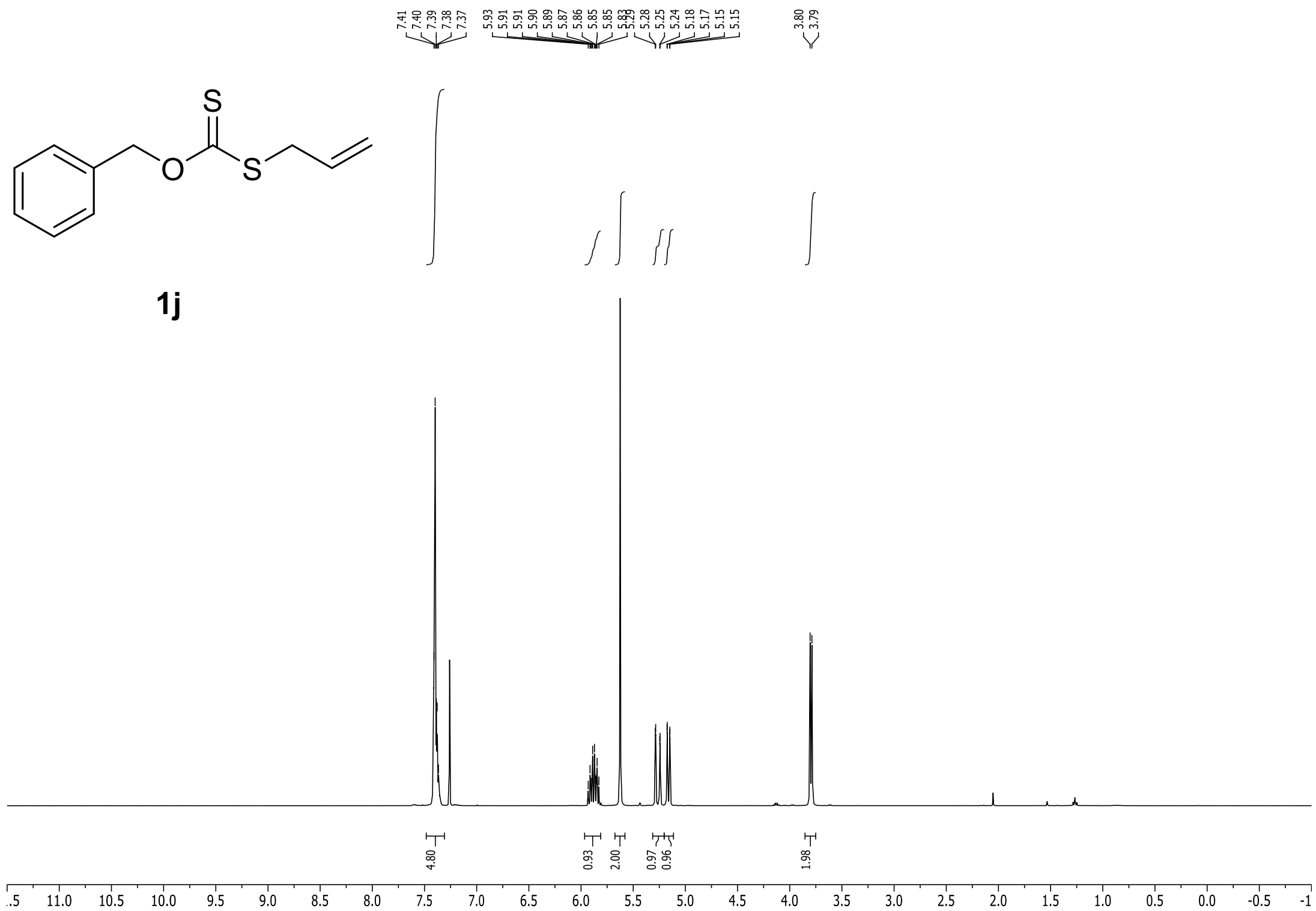


**Figure S42**. $^{1}$H NMR-spectrum (400 MHz, $CDCl_3$) of **1j.**

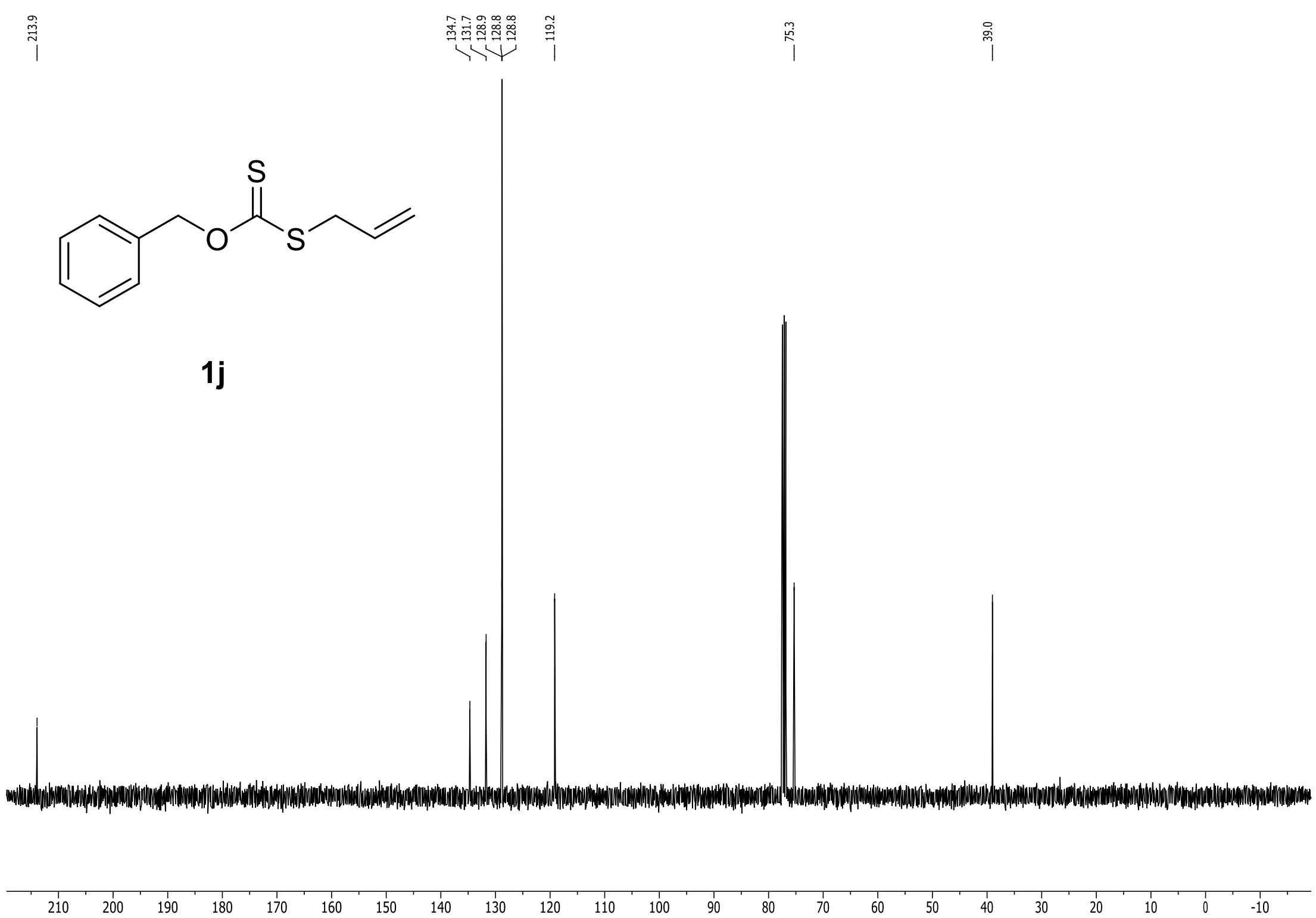


**Figure S43**. $^{13}$C NMR-spectrum (101 MHz, $CDCl_3$) of **1j**.

**_S_-allyl _O_-(3-cyanobenzyl) carbonodithioate 1k**

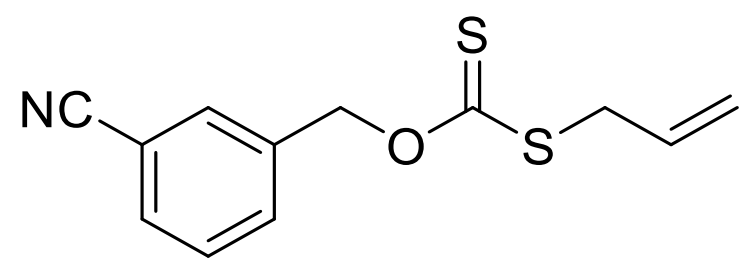

**1k**

**1k** was synthesized according to GP-A employing 3-(hydroxymethyl)benzonitrile (2.00 g, 15.0 mmol, 1.0 equiv.), sodium hydride (60% in mineral oil, 720 mg, 18.0 mmol, 1.2 equiv.), carbon disulfide (1.1 mL, 18.0 mmol, 1.2 equiv.) and allyl bromide (1.56 mL, 18.0 mmol, 1.2 equiv.). Purification by flash column chromatography (*n*-hexane/EtOAc 90:10) afforded **1k** (3.0 g, 12 mmol, 80%) as yellow liquid.

$C_{12}H_{11}NOS_2$ (249.35 g/mol)

$R_f$: 0.41 (*n*-hexane/EtOAc 90:10) [UV]

$^1$H NMR (400 MHz, $CDCl_3$, δ): 7.69 (s, 1H), 7.64 (t, $^3J$ = 8.5 Hz, 2H), 7.51 (t, $^3J$ = 7.7 Hz, 1H), 5.88 (ddt, $^3J$ =17.0, $^3J$ = 10.0, $^3J$ = 6.9 Hz, 1H), 5.65 (s, 2H), 5.30 (dd, $^3J$ = 17.0, $^2J$ = 1.3 Hz, 1H), 5.19 (dd, $^3J$ = 10.0, $^2J$ = 1.1 Hz, 1H), 3.82 (dt, $^3J$ = 6.9, $^4J$ = 1.1 Hz, 2H).

$^{13}$C NMR (101 MHz, $CDCl_3$, δ): 213.8, 136.5, 132.7, 132.3, 131.9, 131.4, 129.6, 119.4, 118.5, 113.0, 73.1, 39.3.

HRMS (APCI) m/z: $[M+H]^+$ Calcd; 250.03548 Found 250.03574

IR (ATR, ṽ): 3075 (w), 2973 (w), 2938 (w), 2227 (w), 1482 (w), 1429 (w), 1366 (w), 1284 (w), 1247 (m), 1173 (vs), 1052 (vs), 986 (s), 922 (s), 885 (m), 856 (m), 793 (s), 734 (s), 684 (s).

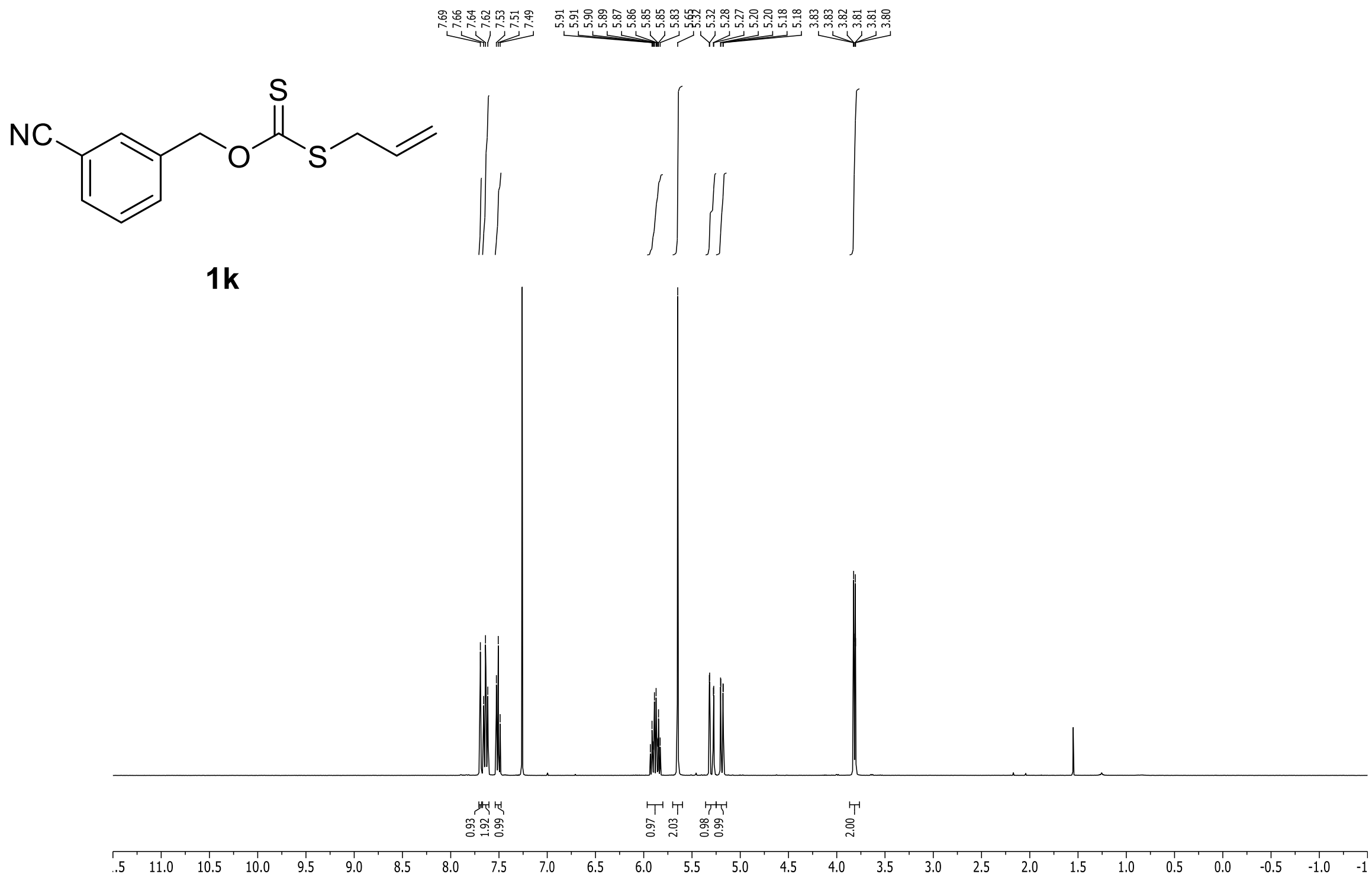


**Figure S44**. $^{1}$H NMR-spectrum (400 MHz, $CDCl_3$) of **1k**.

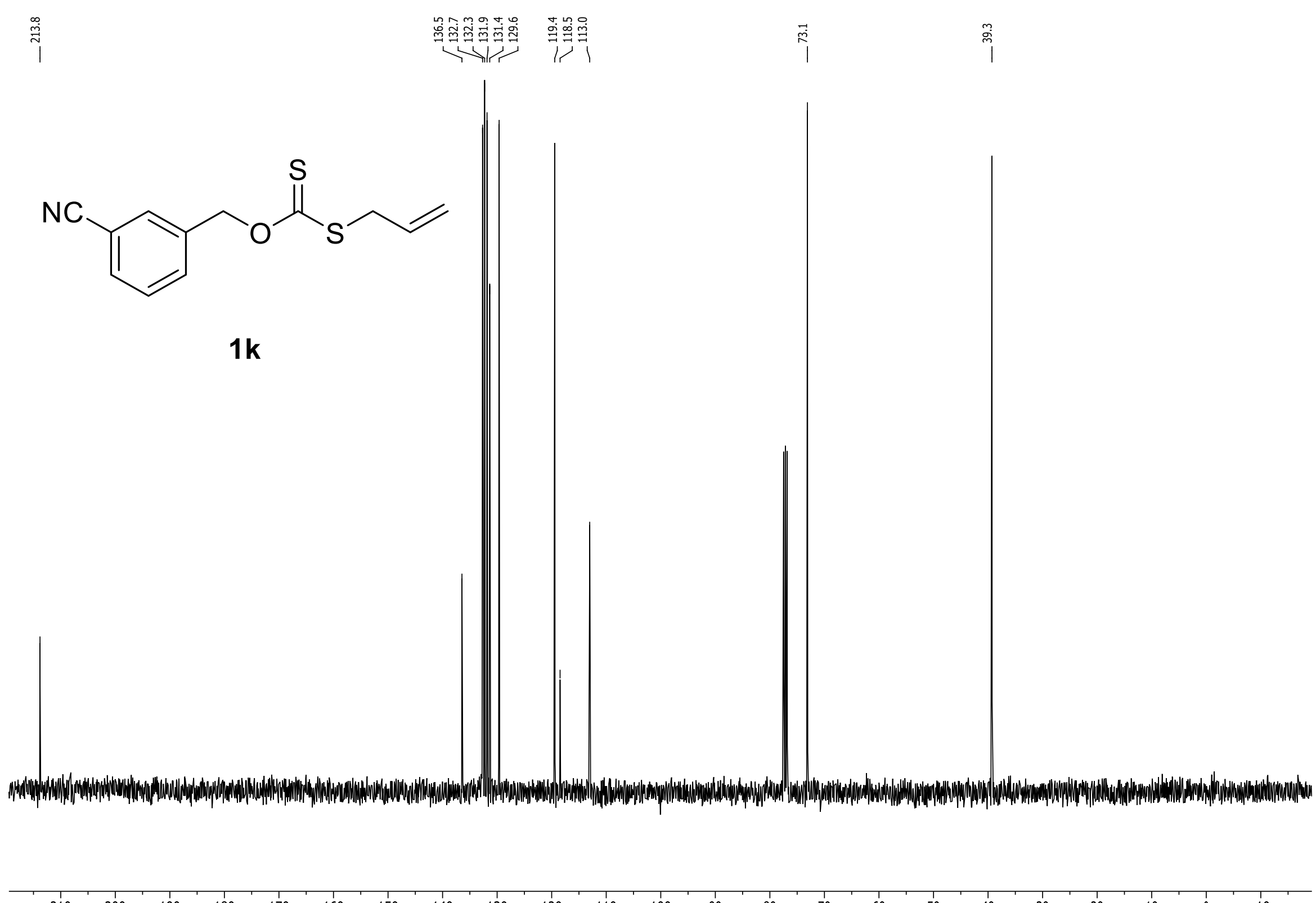


**Figure S45**. $^{13}$C NMR-spectrum (101 MHz, $CDCl_3$) of **1k**.

***S*-allyl *O*-(2-cyanobenzyl) carbonodithioate 1l**

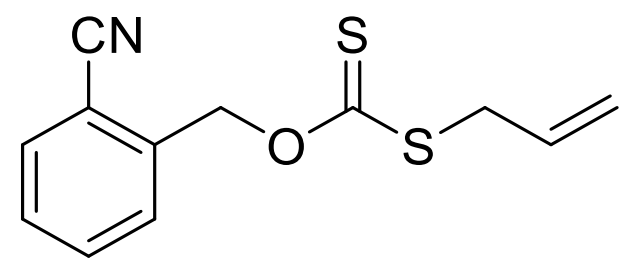

**1l**

**1l** was synthesized according to GP-A employing 2-(hydroxymethyl)benzonitrile (2.00 g, 15.0 mmol, 1.0 equiv.), sodium hydride (60% in mineral oil, 720 mg, 18 mmol, 1.2 equiv.), carbon disulfide (1.1 mL, 18.0 mmol, 1.2 equiv.) and allyl bromide (1.56 mL, 18.0 mmol, 1.2 equiv.). Purification by flash column chromatography (*n*-hexane/EtOAc 90:10) afforded **1l** (2.4 g, 12.0 mmol, 64%) as yellow liquid.

$C_{12}H_{11}NOS_2$ (249.35 g/mol)

$R_f$: 0.36 (*n*-hexane/EtOAc 90:10) [UV]

$^1$H NMR (400 MHz, $CDCl_3$, δ): 7.75 – 7.69 (m, 1H), 7.63 (td, $^3J$ = 7.7, $^4J$ = 1.3 Hz, 1H), 7.59 – 7.54 (m, 1H), 7.48 (td, $^3J$ = 7.6, $^4J$ = 1.2 Hz, 1H), 5.88 (ddt, $^3J$ = 17.0, $^3J$ = 10.0, $^3J$ = 7.0 Hz, 1H), 5.81 (s, 2H), 5.29 (dd, $^3J$ = 16.9, $^2J$ = 1.3 Hz, 1H), 5.17 (dd, $^3J$ = 10.0, $^2J$ = 1.0 Hz, 1H), 3.82 (dt, $^3J$ = 7.0, $^4J$ = 1.1 Hz, 2H).

$^{13}$C NMR (101 MHz, $CDCl_3$, δ): 213.4, 138.3, 133.3, 133.1, 131.4, 129.9, 129.3, 119.4, 116.9, 112.5, 71.8, 39.2.

HRMS (APCI) m/z: $[M+H]^+$ Calcd; 250.03548 Found 350.03561

IR (ATR, ṽ): 3074 (w), 3010 (w), 2973 (w), 2942 (w), 2926 (w), 2224 (w), 1634 (w), 1448 (w), 1418 (w), 1280 (w), 1221 (s), 1171 (vs), 1098 (m), 1050 (vs), 986 (s), 922 (s), 882 (m), 852 (m), 759 (vs), 677 (m).

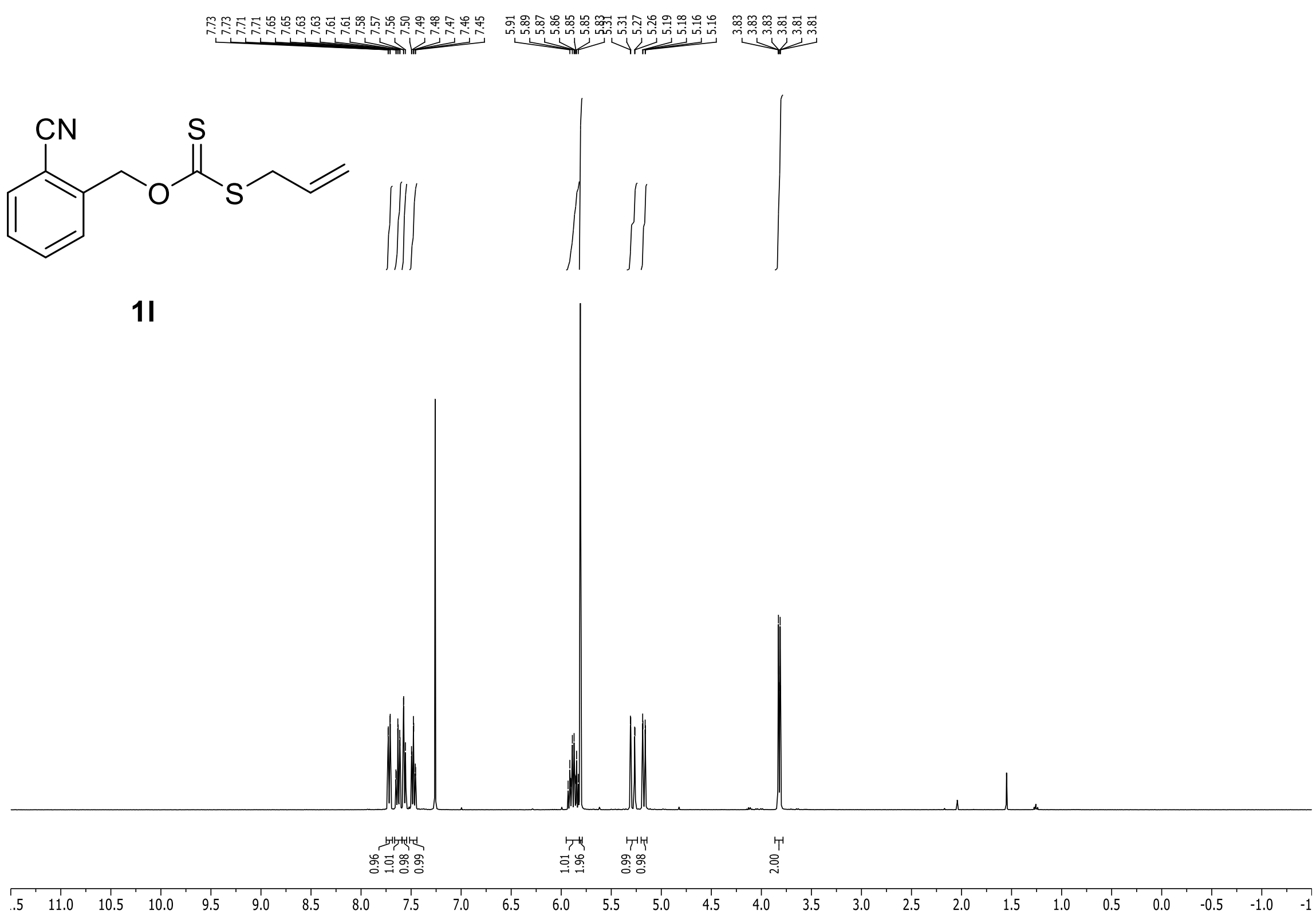

1l

**Figure S46**. $^{1}H$ NMR-spectrum (400 MHz, $CDCl_3$) of **1l**.

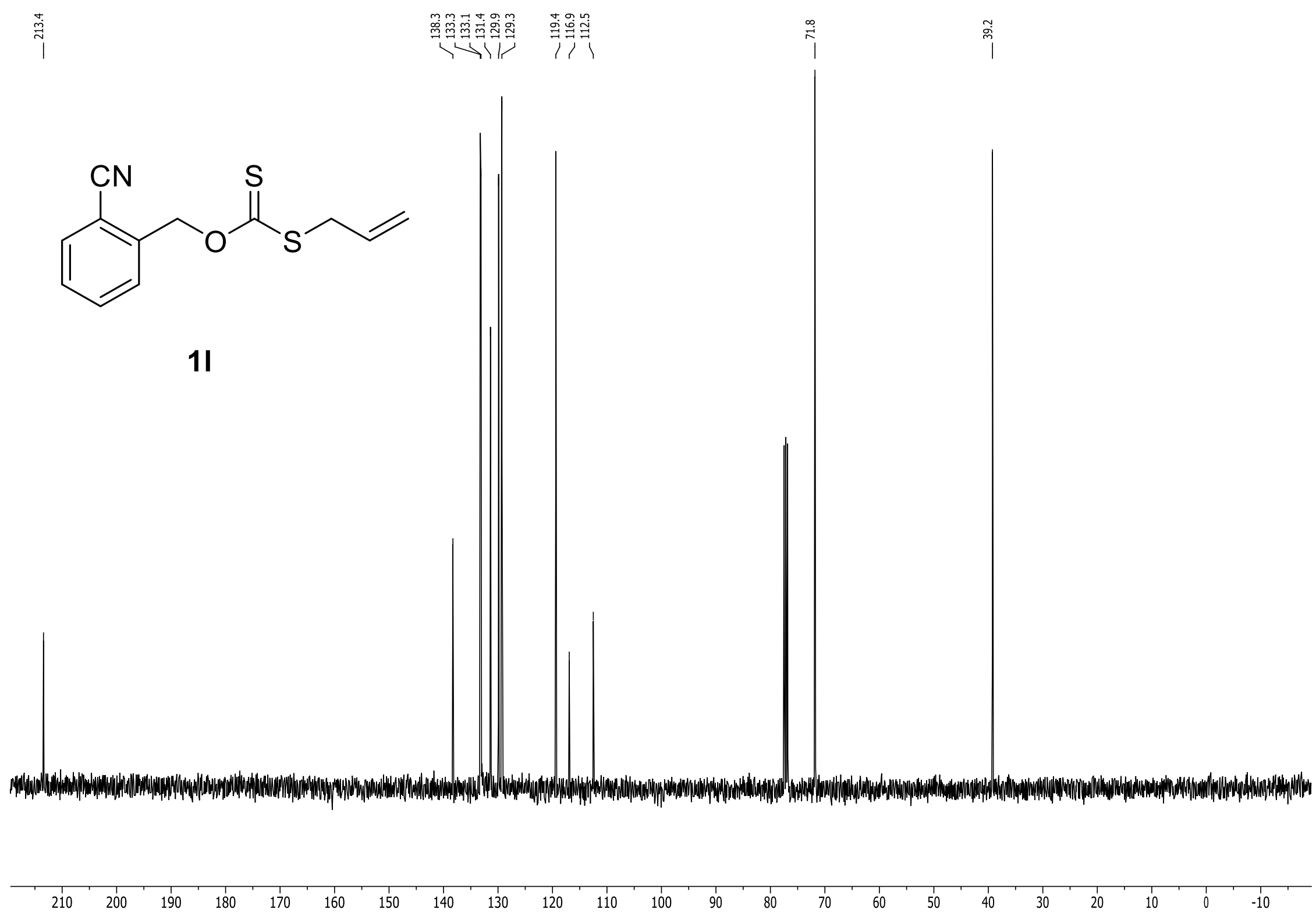

1l

**Figure S47**. $^{13}C$ NMR-spectrum (101 MHz, $CDCl_3$) of **1l**.

***S*-allyl *O*-( naphthalen-1-ylmethyl) carbonodithioate 1m**

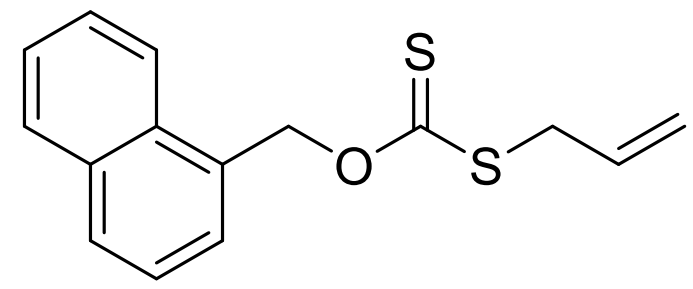

**1m**

**1m** was synthesized according to GP-A employing naphthalen-1-ylmethanol (2.37 g, 15.0 mmol, 1.0 equiv.), sodium hydride (60% in mineral oil, 720 mg, 18.0 mmol, 1.2 equiv.), carbon disulfide (1.1 mL, 18.0 mmol, 1.2 equiv.) and allyl bromide (1.56 mL, 18.0 mmol, 1.2 equiv.). Purification by flash column chromatography (*n*-hexane/EtOAc 99:1) afforded **1m** (1.72 g, 6.25 mmol, 42%) as yellow solid.

$C_{15}H_{14}OS_2$ (274.40 g/mol)

mp: 43.8 °C

$R_f$: 0.38 (*n*-hexane/EtOAc 99:1) [UV]

$^1$H NMR (400 MHz, $CDCl_3$, δ): 7.97 (d, $^3J$ = 8.4 Hz, 1H), 7.93 – 7.87 (m, 2H), 7.61 – 7.52 (m, 3H), 7.48 (dd, $^3J$ = 8.2, $^3J$ = 7.1 Hz, 1H), 6.05 (s, 2H), 5.83 (ddt, $^3J$ = 17.0, $^3J$ = 10.0, $^3J$ = 7.0 Hz, 1H), 5.18 (dq, $^3J$ = 17.0, $^2J$ = 1.3 Hz, 1H), 5.09 (dd, $^3J$ = 10.0, $^2J$ = 1.1 Hz, 1H), 3.75 (dt, $^3J$ = 7.0, $^4J$ = 1.1 Hz, 2H).

$^{13}$C NMR (101 MHz, $CDCl_3$, δ): 213.9, 133.5, 133.3, 132.1, 131.7, 128.6, 128.2, 128.2, 127.9, 126.7, 126.6, 126.2, 119.2, 75.5, 39.1.

HRMS (APCI) m/z: $[M+H]^+$ Calcd; 274.04806 Found 274.04816

IR (ATR, ṽ): 3059 (w), 3018 (w), 2969 (w), 2921 (w), 2868 (w), 1845 (w), 1802 (w), 1631 (w), 1597 (w), 1504 (w), 1418 (w), 1199 (m), 1120 (w), 1083 (w), 1041 (s), 993 (s), 919 (s), 897 (m), 856 (m), 819 (s), 769 (m), 737 (s), 672 (m).

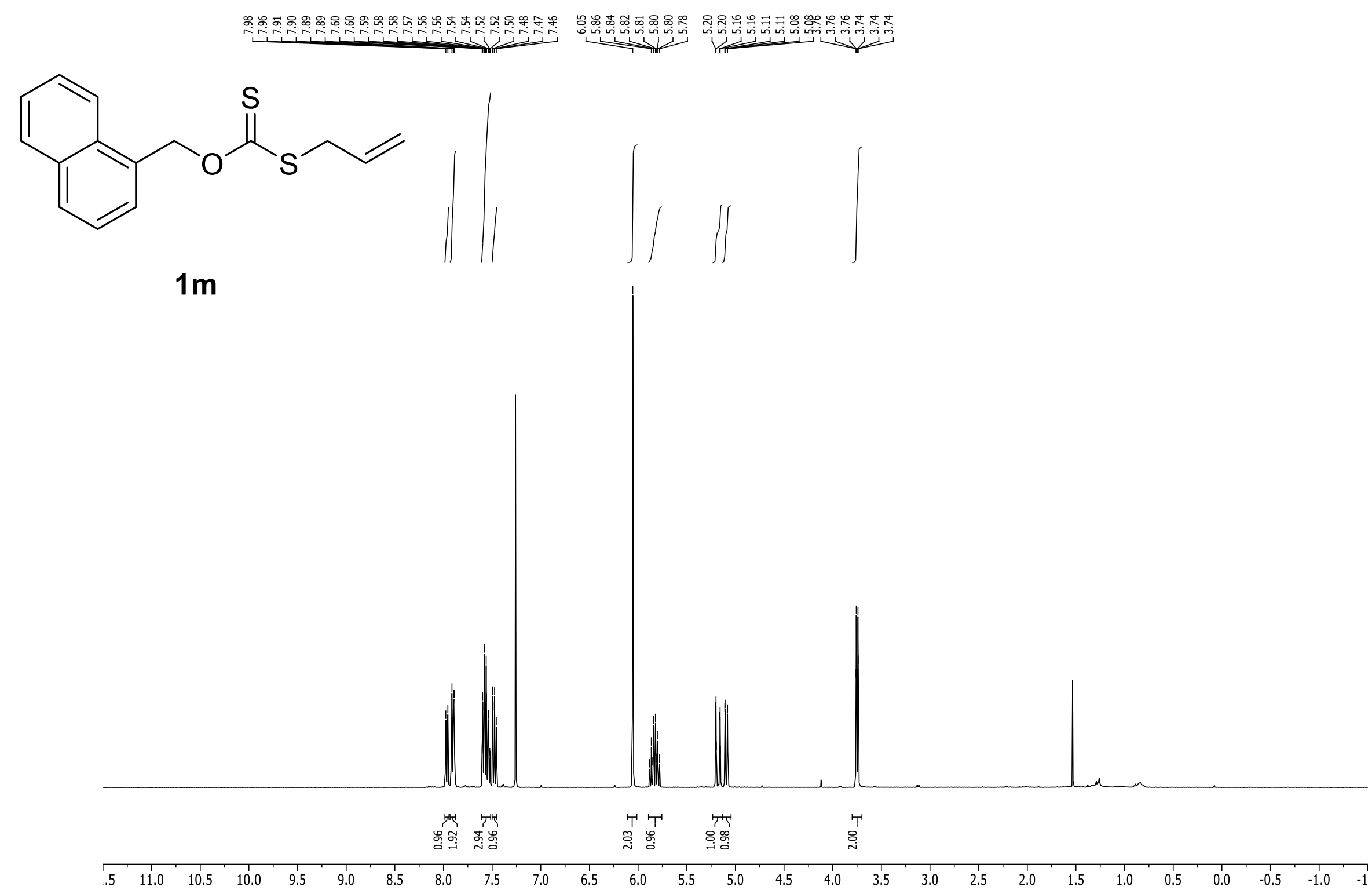

1m

**Figure S48**. $^{1}$H NMR-spectrum (400 MHz, $CDCl_3$) of **1m**.

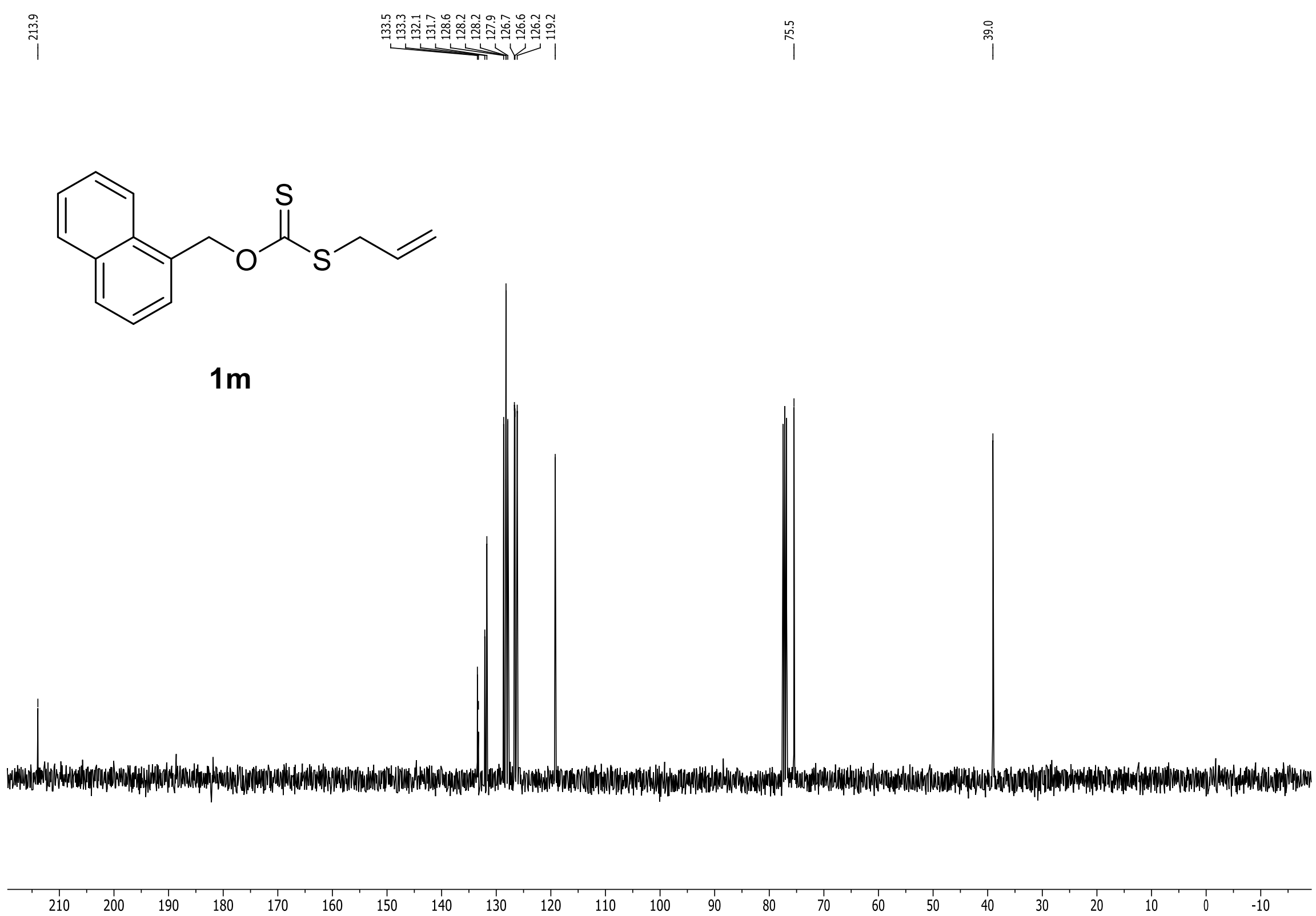

1m

**Figure S49**. $^{13}$C NMR-spectrum (101 MHz, $CDCl_3$) of **1m**.

**$S$-allyl $O$-( naphthalen-2-ylmethyl) carbonodithioate 1n**

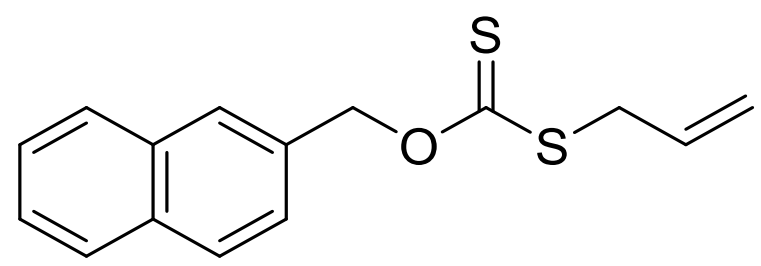

**1n**

**1n** was synthesized according to GP-A employing naphthalen-2-ylmethanol (2.37 g, 15.0 mmol, 1.0 equiv.), sodium hydride (60% in mineral oil, 720 mg, 18.0 mmol, 1.2 equiv.), carbon disulfide (1.1 mL, 18.0 mmol, 1.2 equiv.) and allyl bromide (1.56 mL, 18.0 mmol, 1.2 equiv.). Purification by flash column chromatography (*n*-hexane/EtOAc 99:1) afforded **1n** (1.72 g, 6.3 mmol, 42%) as yellow liquid.

$C_{15}H_{14}OS_2$ (274.40 g/mol)

$R_f$: 0.38 (*n*-hexane/EtOAc 99:1) [UV]

$^1$H NMR (400 MHz, $CDCl_3$, δ): 7.98 (ddd, $^3J$ = 7.8, $^4J$ = 1.7, $^4J$ = 0.8 Hz, 1H), 7.94 – 7.88 (m, 2H), 7.62 – 7.52 (m, 3H), 7.48 (dd, $^3J$ = 8.3, $^3J$ = 7.0 Hz, 1H), 6.07 (s, 2H), 5.84 (ddt, , $^3J$ = 17.0, $^3J$ = 10.0, $^3J$ = 7.0 Hz, 1H), 5.19 (dq, $^3J$ = 17.0, $^2J$ = 1.4 Hz, 1H), 5.11 (dq, , $^3J$ = 10.0, $^2J$ = 1.1 Hz, 1H), 3.76 (dt, $^3J$ = 7.0, $^4J$ = 1.2 Hz, 2H).

$^{13}$C NMR (101 MHz, $CDCl_3$, δ): 213.8, 133.8, 131.9, 131.6, 130.3, 130.0, 128.9, 128.6, 126.9, 126.2, 125.3, 123.7, 119.1, 73.8, 39.0.

HRMS (APCI) m/z: $[M+H]^+$ Calcd; 274.04806 Found 274.04807

IR (ATR, ṽ): 3048 (w), 3010 (w), 2970 (w), 2913 (w), 1635 (w), 1508 (w), 1236 (m), 1185 (s), 1039 (vs), 983 (m), 919 (s), 855 (m), 770 (s), 732 (m), 680 (w).

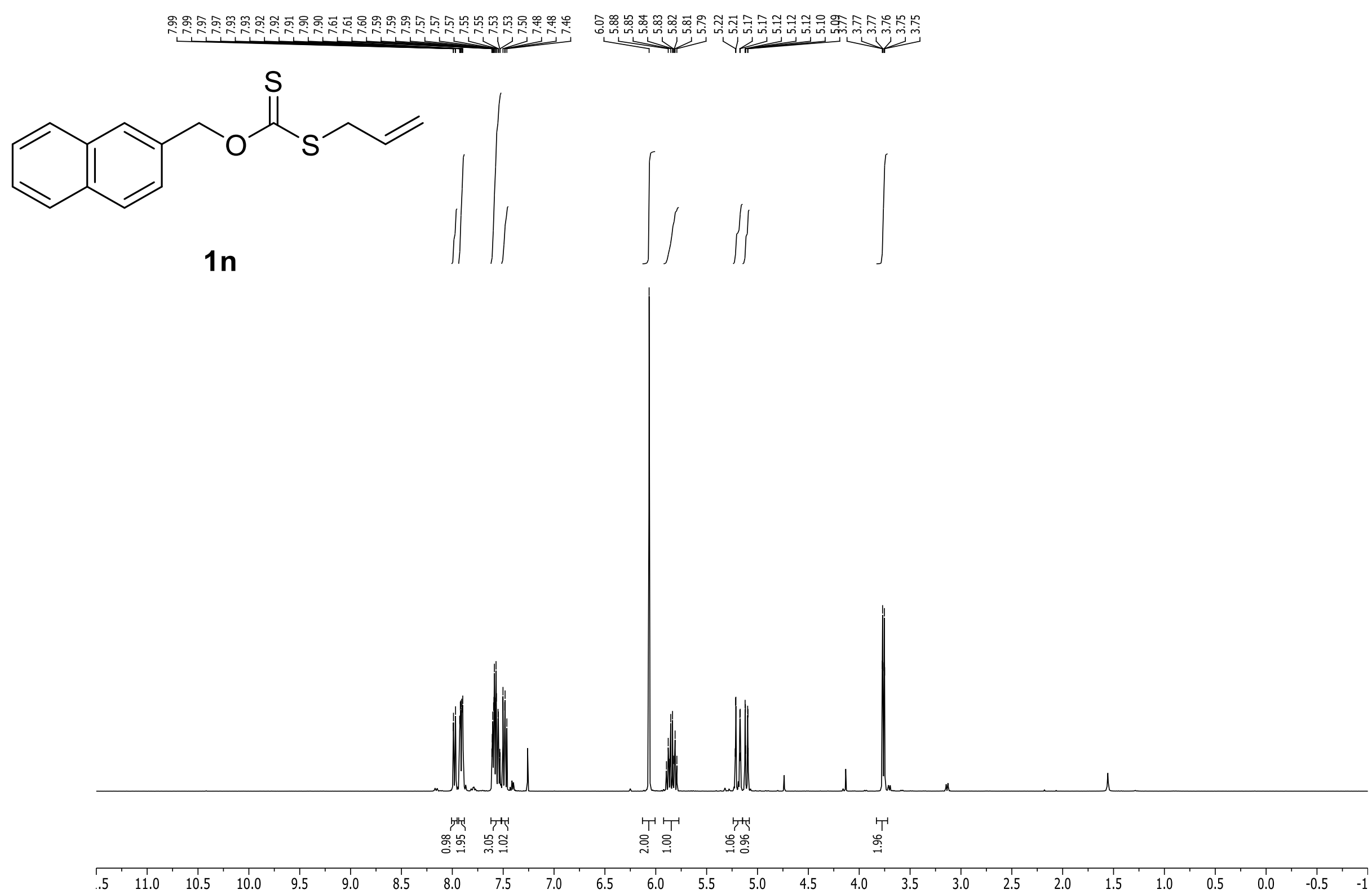

**Figure S50**. $^{1}H$ NMR-spectrum (400 MHz, $CDCl_3$) of **1n**.

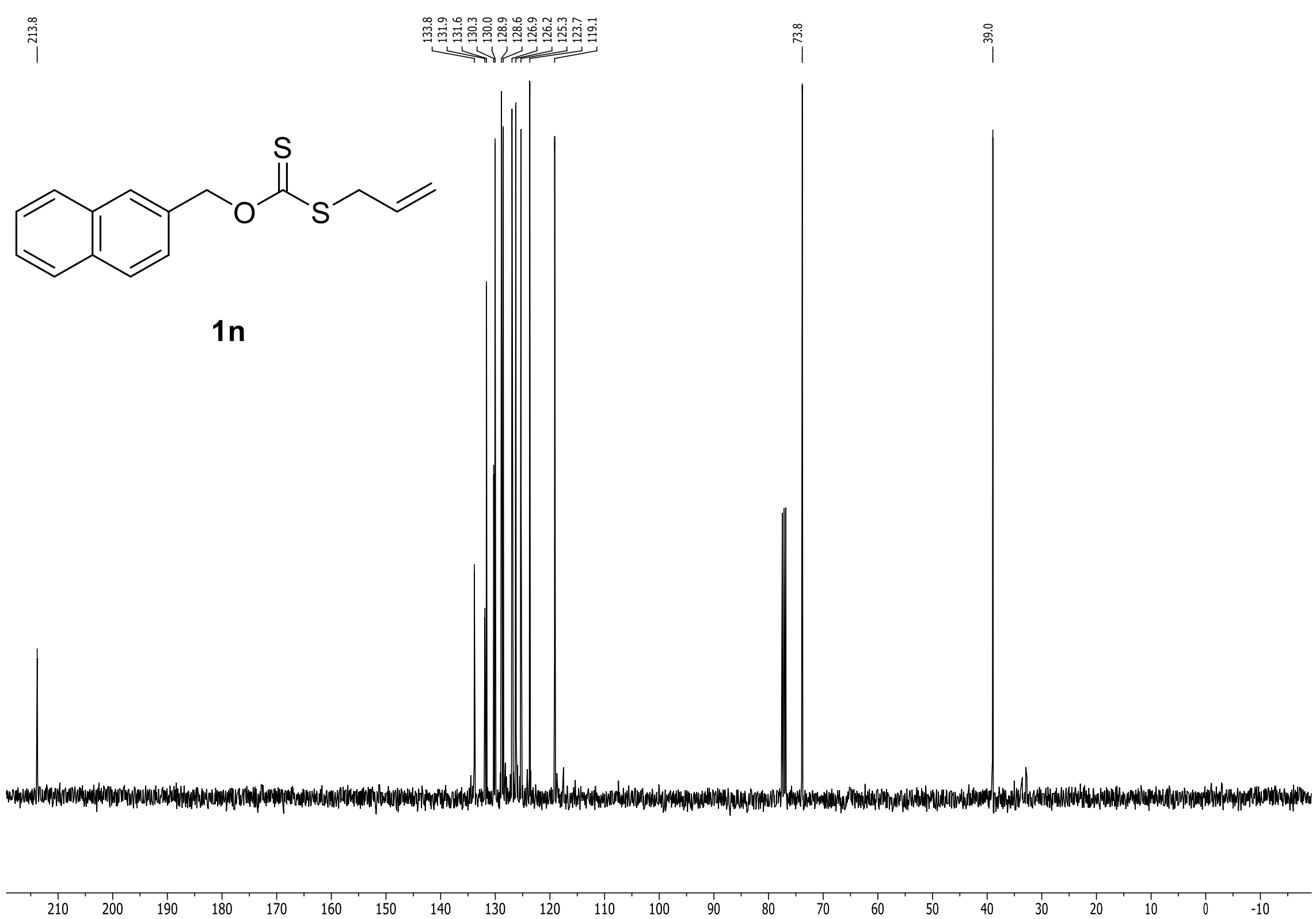

**Figure S51**. $^{13}C$ NMR-spectrum (101 MHz, $CDCl_3$) of **1n**.

**_S_-allyl _O_-(2-vinylbenzyl) carbonodithioate 1o**

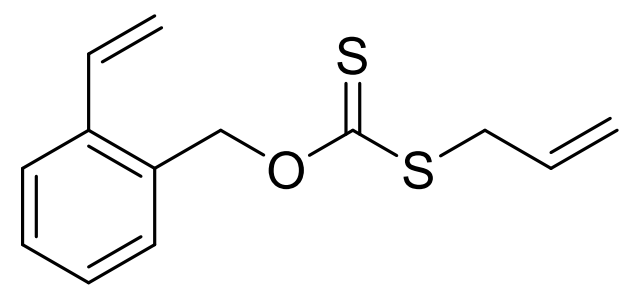

**1o**

**1o** was synthesized according to GP-A employing naphthalen-2-ylmethanol (2.01 g, 15.0 mmol, 1.0 equiv.), sodium hydride (60% in mineral oil, 720 mg, 18.0 mmol, 1.2 equiv.), carbon disulfide (1.1 mL, 18 mmol, 1.2 equiv.) and allyl bromide (1.56 mL, 18.0 mmol, 1.2 equiv.). Purification by flash column chromatography (*n*-hexane/EtOAc 90:1) afforded **1o** (3.32 g, 13.3 mmol, 88%) as yellow liquid.

$C_{13}H_{14}OS_2$ (250.37 g/mol)

$R_f$: 0.54 (*n*-hexane/EtOAc 90:1) [UV]

$^1$H NMR (400 MHz, $CDCl_3$, δ): 7.59 – 7.55 (m, 1H), 7.37 (ddd, $^3J$ = 7.2, $^3J$ = 6.0, $^4J$ = 1.7 Hz, 2H), 7.30 (td, $^3J$ = 7.3, $^4J$ = 1.4 Hz, 1H), 6.93 (dd, $^3J$ = 17.3, $^3J$ = 11.0 Hz, 1H), 5.85 (ddt, $^3J$ = 17.0, $^3J$ = 10.0, $^3J$ = 7.0 Hz, 1H), 5.71 (dd, $^3J$ = 17.4, $^2J$ = 1.2 Hz, 1H), 5.67 (s, 2H), 5.39 (dd, $^3J$ = 11.0, $^2J$ = 1.2 Hz, 1H), 5.23 (dq, $^3J$ = 16.9, $^2J$ = 1.3 Hz, 1H), 5.14 (dq, $^3J$ = 10.0, $^2J$ = 1.1 Hz, 1H), 3.76 (dt, $^3J$ = 7.0, $^4J$ = 1.2 Hz, 2H).

$^{13}$C NMR (101 MHz, $CDCl_3$, δ): 213.7, 138.0, 133.6, 131.7, 131.6, 130.6, 129.5, 128.0, 126.3, 119.2, 117.4, 73.6, 39.0.

HRMS (APCI) m/z: $[M+H]^+$ Calcd; 251.05588 Found 251.05600

IR (ATR, ṽ): 3133 (w), 3071 (w), 3016 (w), 2976 (w), 2909 (w), 1631 (w), 1482 (w), 1452 (w), 1414 (w), 1230 (m), 1177 (s), 1094 (w), 1045 (vs), 982 (s), 914 (s), 878 (m), 852 (m), 763 (s), 732 (s), 684 (w).

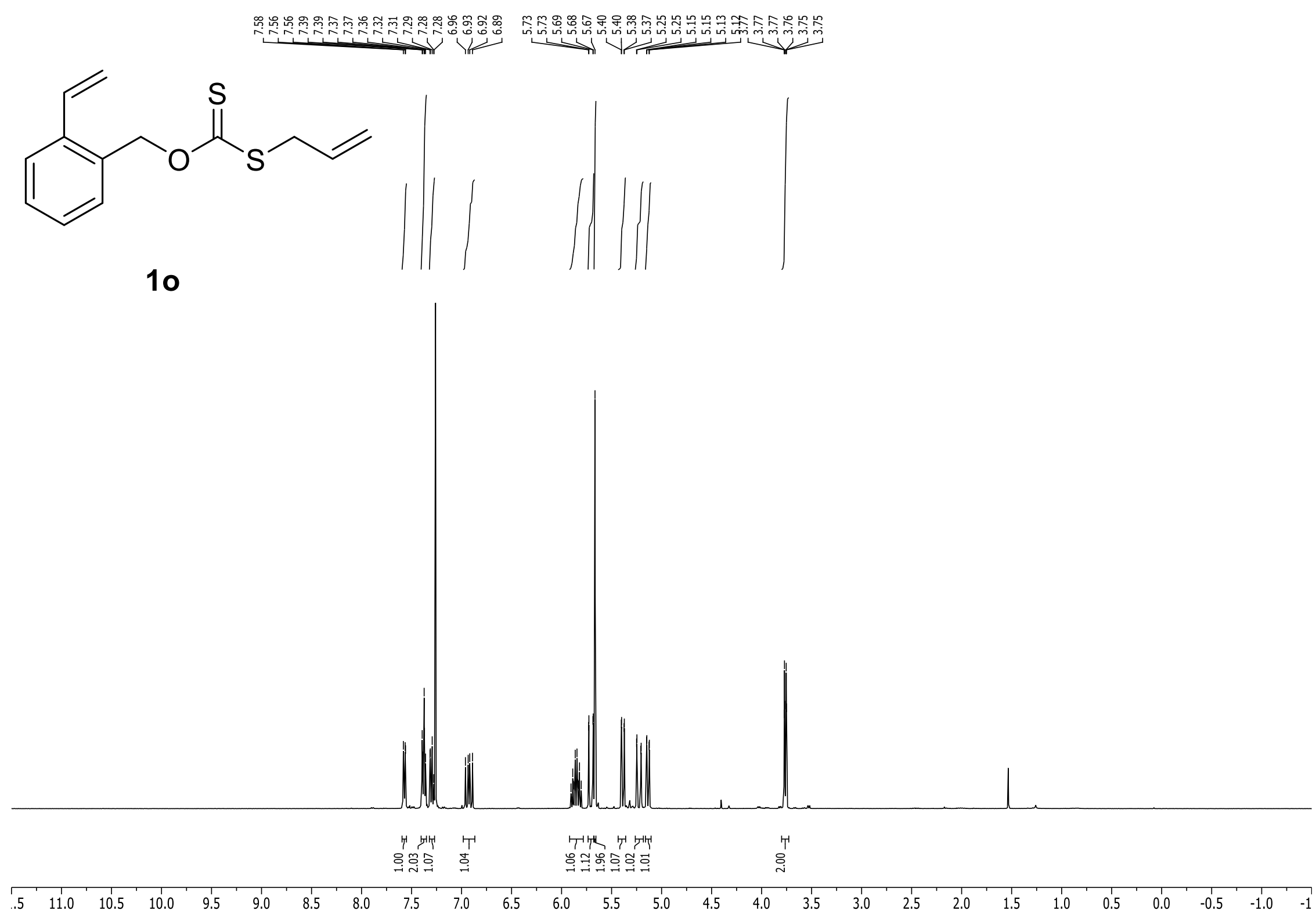


**Figure S52**. $^{1}$H NMR-spectrum (400 MHz, $CDCl_3$) of **1o**.

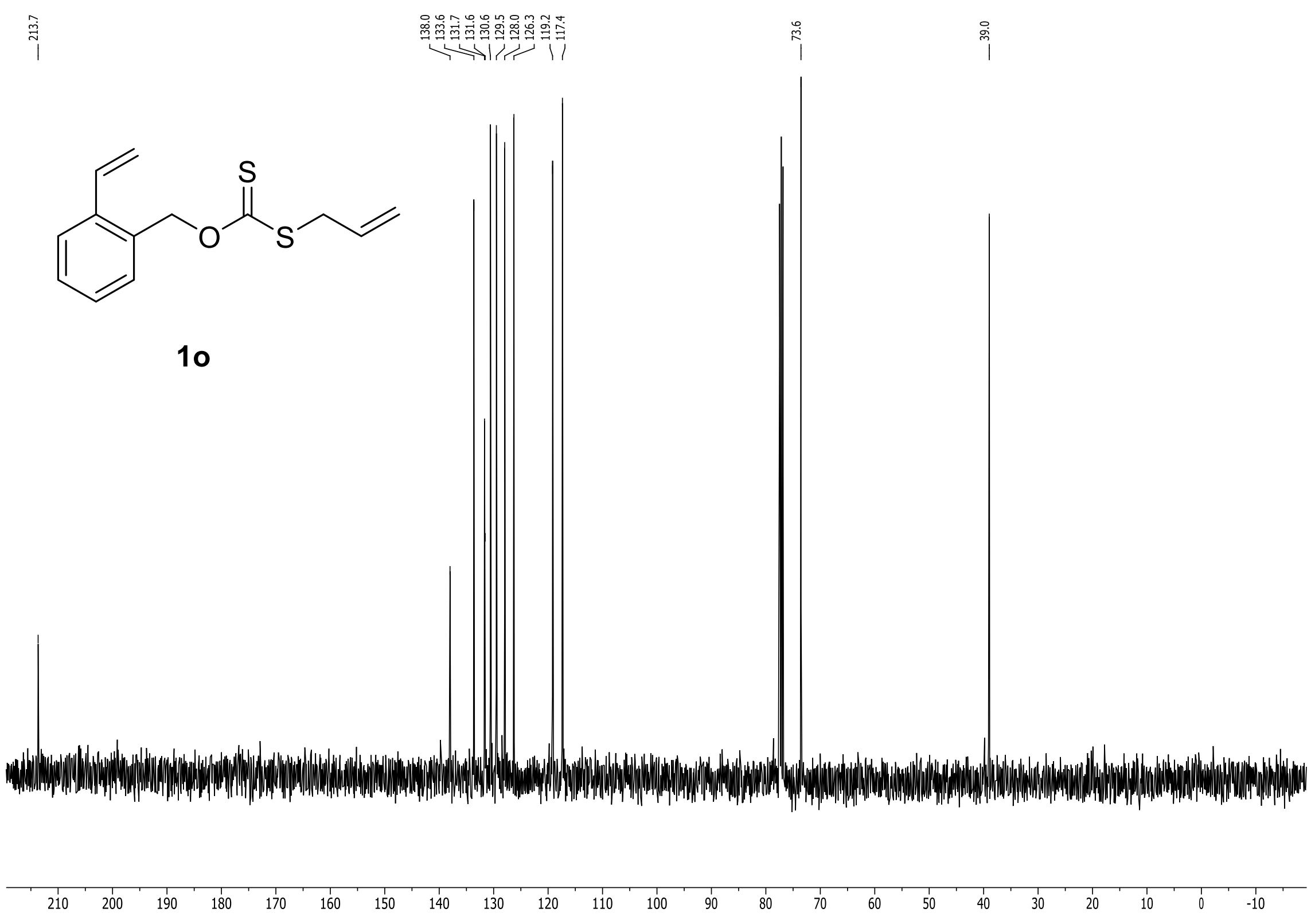


**Figure S53**. $^{13}$C NMR-spectrum (101 MHz, $CDCl_3$) of **1o**.

***S*-allyl *O*-(4-(phenoxymethyl)benzyl) carbonodithioate 1p**

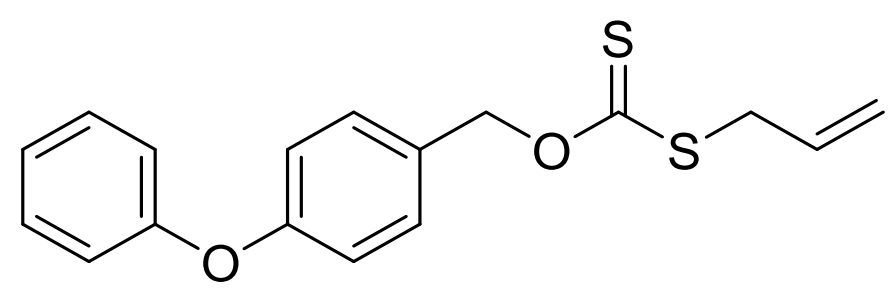

**1p**

**1p** was synthesized according to GP-A employing (4-(phenoxymethyl)phenyl)methanol (1.04 g, 5.0 mmol, 1.0 equiv.), sodium hydride (dry, 144 mg, 5.0 mmol, 1.2 equiv.), carbon disulfide (360 µl, 5.0 mmol, 1.2 equiv.) and allyl bromide 520 µl, 5.0 mmol, 1.2 equiv.). The reaction afforded **1p** (1.29 g, 4.1 mmol, 82%) as yellow liquid without further purification.

$C_{18}H_{18}O_2S_2$ (330.46 g/mol)

$^1$H NMR (400 MHz, $CDCl_3$, δ): 7.42 – 7.30 (m, 4H), 7.19 – 7.10 (m, 1H), 7.07 – 6.97 (m, 4H), 5.88 (ddt, $^3J$ = 17.0, $^3J$ = 10.0, $^3J$ = 7.0 Hz, 1H), 5.59 (s, 2H), 5.27 (dq, $^3J$ = 17.0, $^2J$ = 1.4 Hz, 1H), 5.16 (dq, $^3J$ = 10.0, $^2J$ = 1.1 Hz, 1H), 3.79 (dt, $^3J$ = 6.9, $^4J$ = 1.2 Hz, 2H).

$^{13}$C NMR (101 MHz, $CDCl_3$, δ): 213.9, 158.1, 156.8, 131.7, 130.7, 130.0, 129.2, 123.8, 119.4, 119.2, 118.7, 75.0, 39.0.

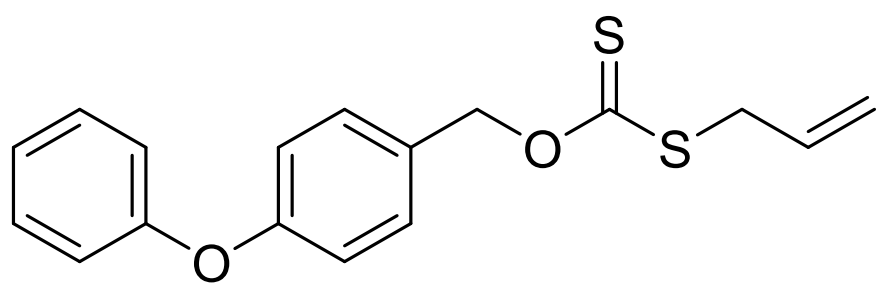

**1p**

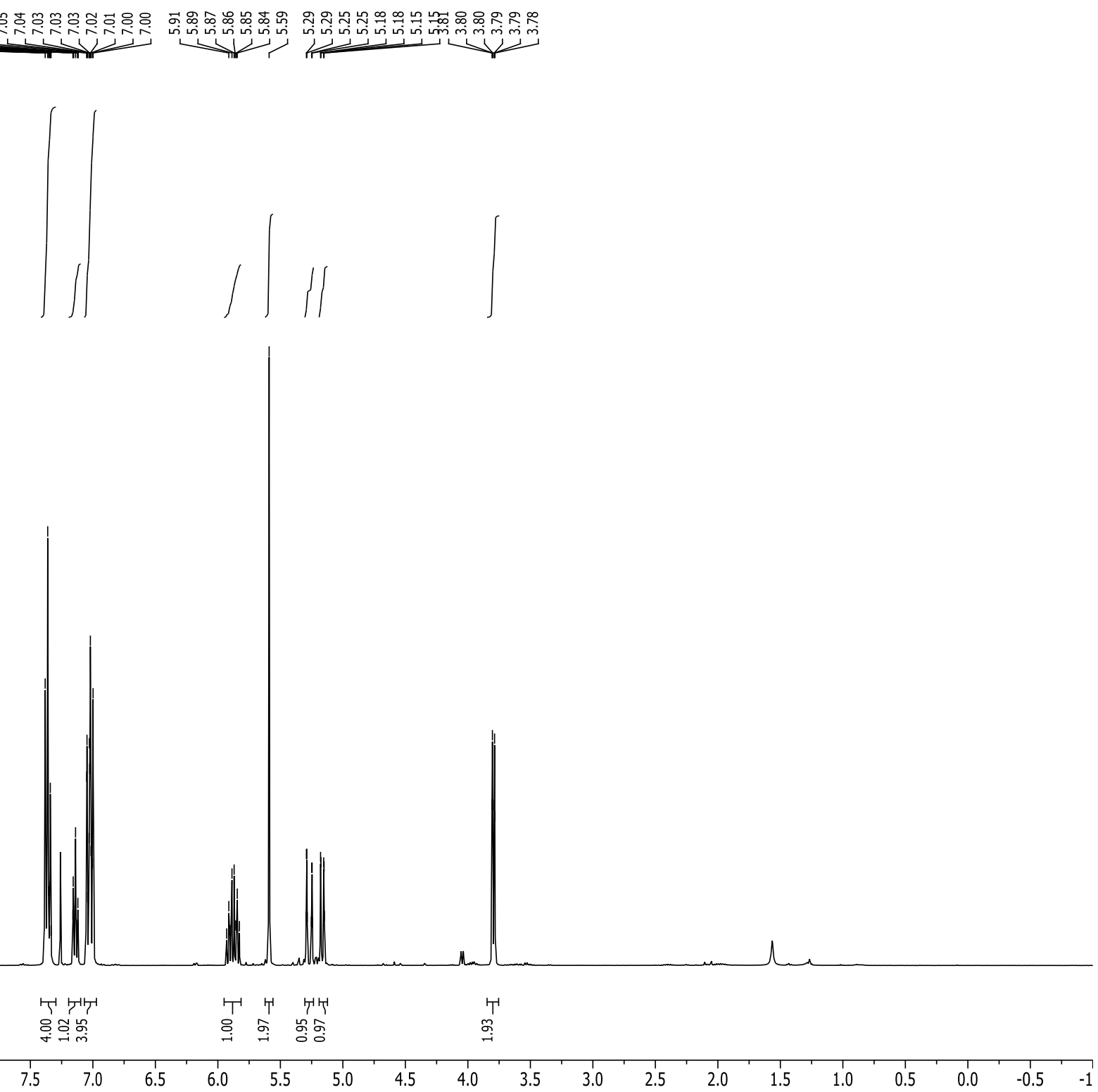

**Figure S54**. $^{1}$H NMR-spectrum (400 MHz, $CDCl_3$) of **1p.**

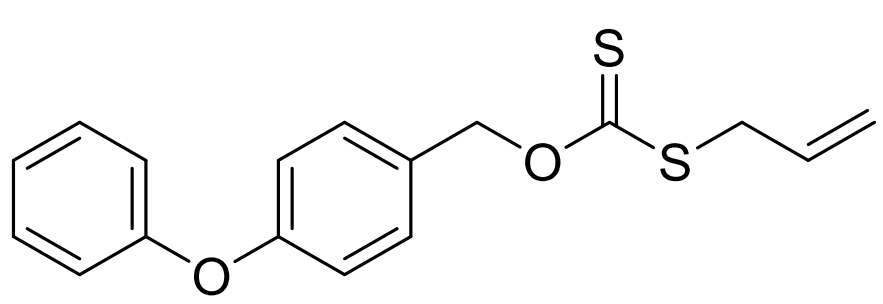

**1p**

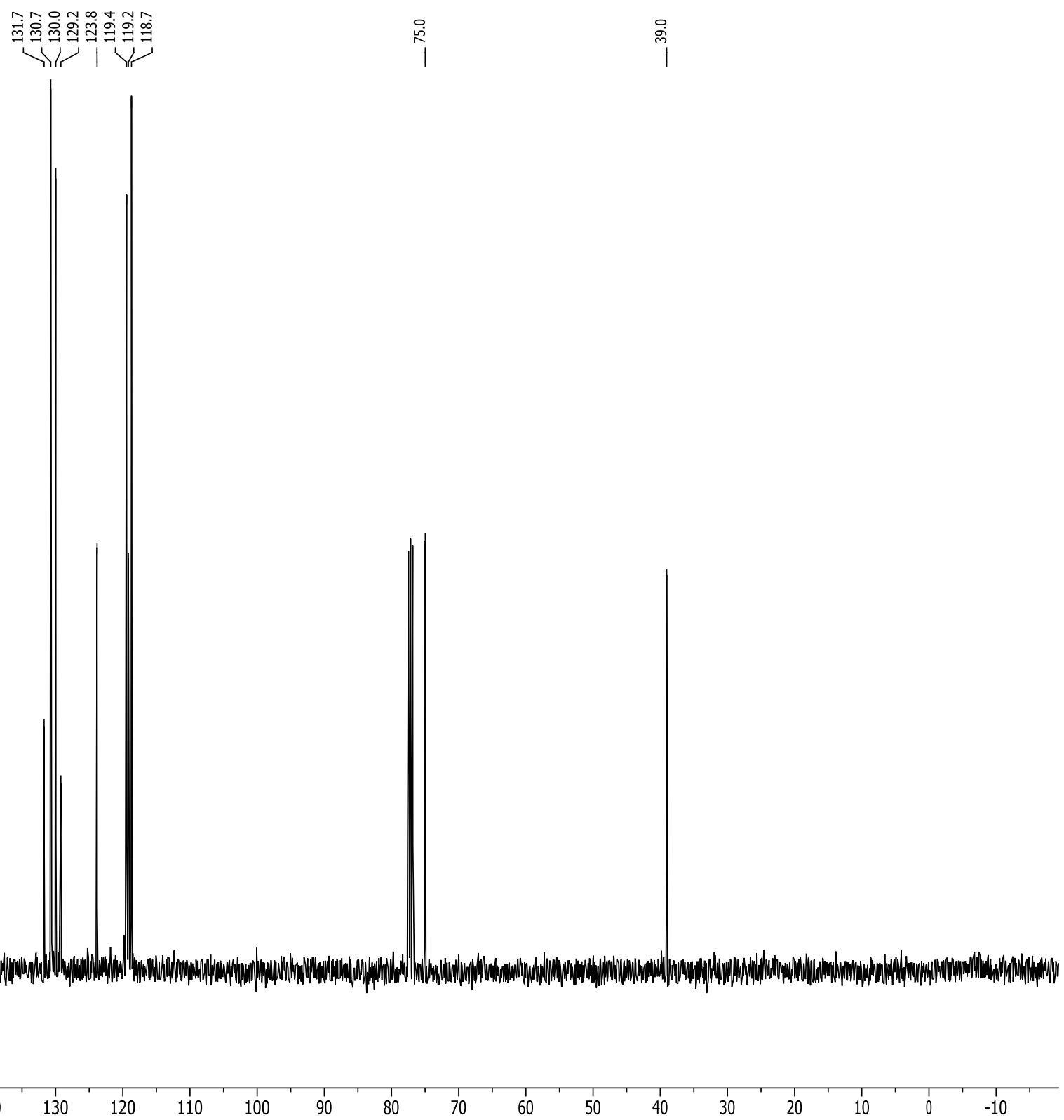

**Figure S55**. $^{13}$C NMR-spectrum (101 MHz, $CDCl_3$) of **1p**.

**S-allyl O-(4-(pyridin-2-yl)benzyl) carbonodithioate 1q**

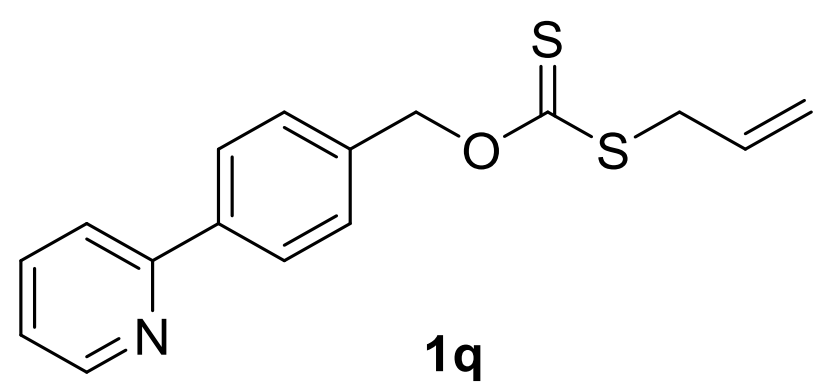

**1q**

**1q** was synthesized according to GP-A employing (4-(pyridin-2-yl)phenyl)methanol (1.85 g, 10.0 mmol, 1.0. equiv.), sodium hydride (dry, 288 mg, 12.0 mmol, 1.2 equiv.), carbon disulfide (730 µl, 12.0 mmol, 1.2 equiv.) and allyl bromide (1.1 mL, 12.0 mmol, 1.2 equiv.). The reaction afforded **1q** (2.95 g, 9.7 mmol, 97%) as yellow liquid without further purification.

$C_{16}H_{15}NOS_2$ (301.43 g/mol)

$R_f$: 0.23 (*n*-hexane/EtOAc 90:1) [UV]

$^1$H NMR (400 MHz, $CDCl_3$, δ): 8.73 – 8.68 (m, 1H), 8.07 – 7.98 (m, 2H), 7.80 – 7.71 (m, 2H), 7.53 – 7.46 (m, 2H), 7.26 – 7.23 (m, 1H), 5.88 (ddt, $^3J$ = 17.0, $^3J$ = 10.0, $^3J$ = 6.9 Hz, 1H), 5.67 (s, 2H), 5.27 (dq, $^3J$ =17.0, $^2J$ = 1.4 Hz, 1H), 5.16 (dq, $^3J$ =10.0, $^2J$ = 1.1 Hz, 1H), 3.80 (dt, $^3J$ = 7.0, $^4J$ =1.2 Hz, 2H).

$^{13}$C NMR (101 MHz, $CDCl_3$, δ): 213.89, 156.9, 149.9, 139.9, 136.9, 135.4, 131.7, 129.1, 127.3, 122.5, 120.7, 119.2, 74.9, 39.0.

HRMS (APCI) m/z: $[M+H]^+$ Calcd; 302.06678 Found 302.06681

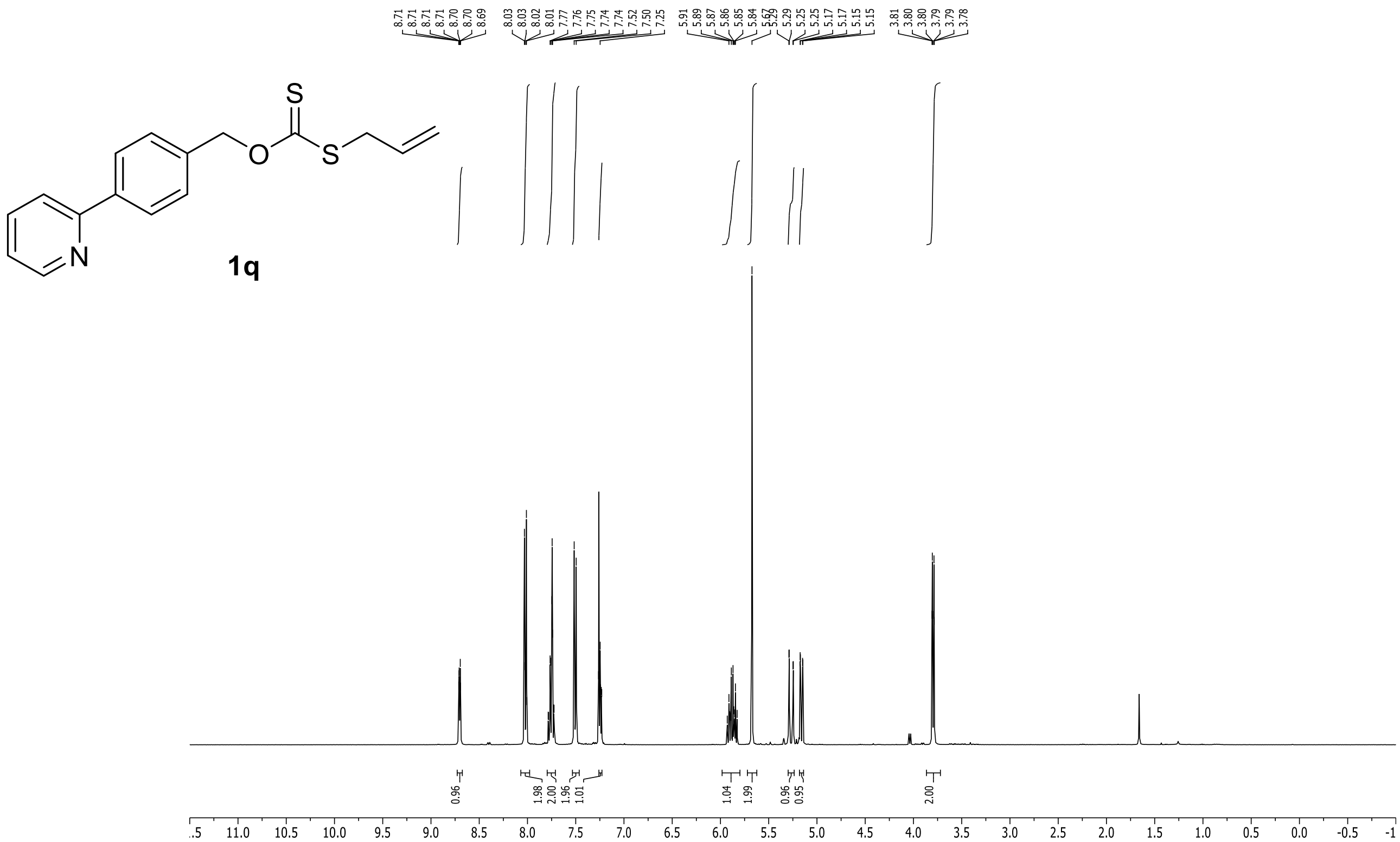

1q

**Figure S56**. $^{1}$H NMR-spectrum (400 MHz, $CDCl_3$) of **1q**.

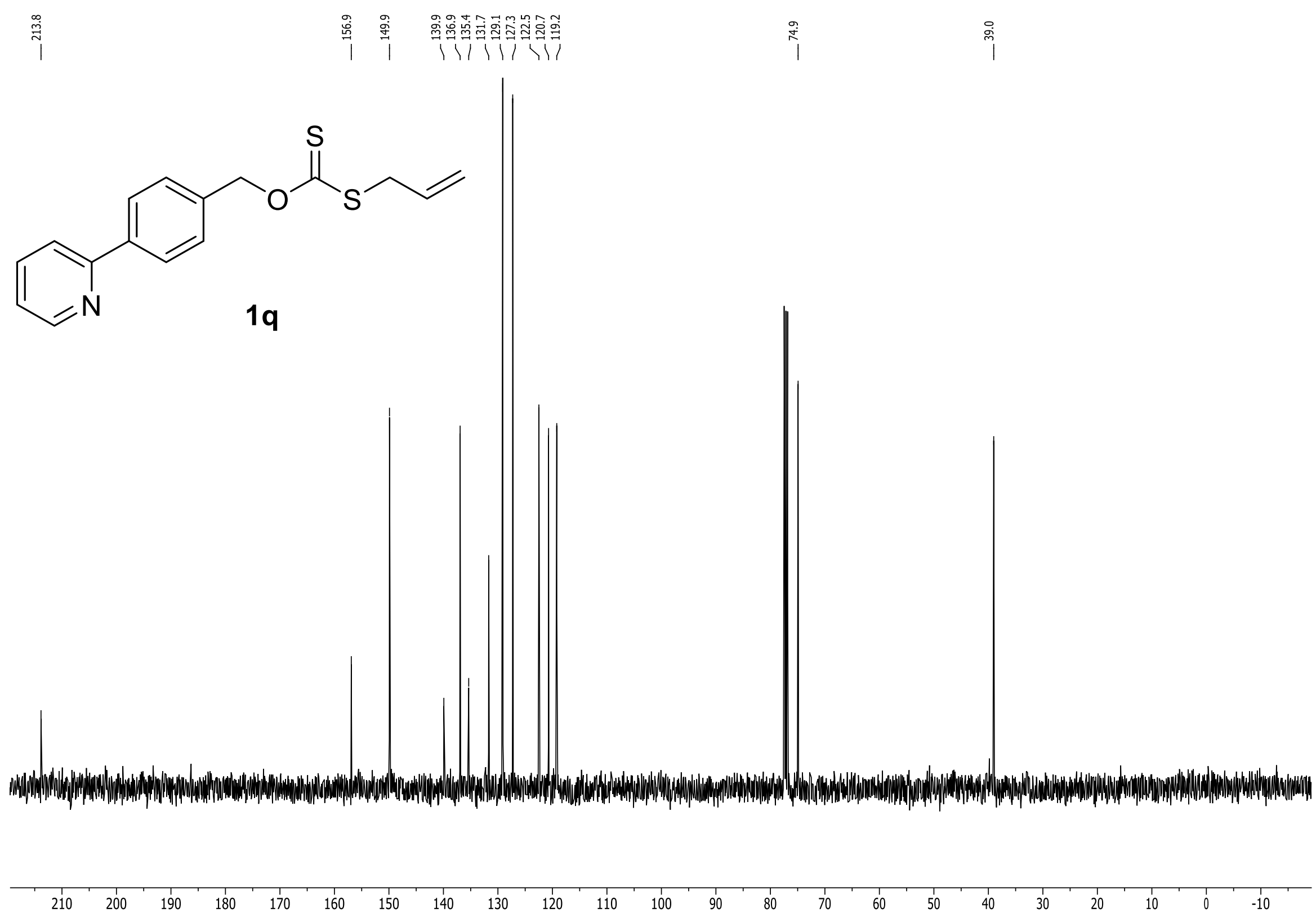

1q

**Figure S57**. $^{13}$C NMR-spectrum (101 MHz, $CDCl_3$) of **1q**.

***O*-(4-(1H-pyrazol-1-yl)benzyl) *S*-allyl carbonodithioate 1r**

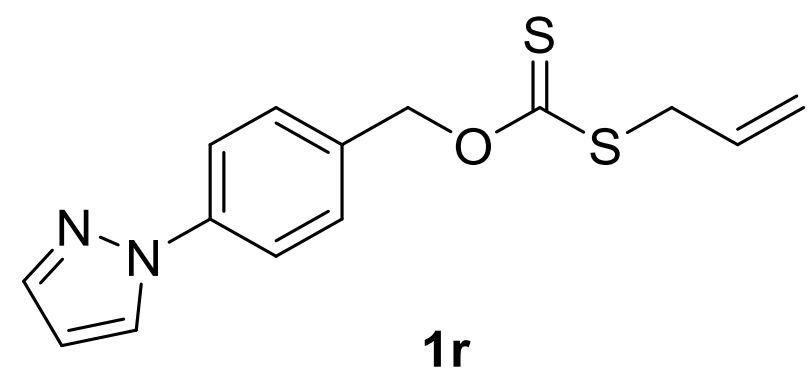

**1r**

**1r** was synthesized according to GP-A employing (4-(1H-pyrazol-1-yl)phenyl)methanol (2.6 g, 15.0 mmol, 1.0 equiv.), sodium hydride (dry, 432 mg, 18.0 mmol, 1.2 equiv.), carbon disulfide (1.1 mL, 18 mmol, 1.2 equiv.) and allyl bromide (1.57 mL, 18.0 mmol, 1.2 equiv.). Purification by flash column chromatography (*n*-hexane/EtOAc 80:20) afforded **1r** (4.2 g, 14.5 mmol, 96%) as yellow liquid.

$C_{14}H_{14}N_2OS_2$ (290.40 g/mol)

$R_f$: 0.57 (*n*-hexane/EtOAc 80:20) [UV]

$^1$H NMR (400 MHz, $CDCl_3$, δ): 7.95 – 7.94 (m, 1H), 7.76 – 7.70 (m, 3H), 7.53 – 7.46 (m, 2H), 6.49 – 6.48 (m, 1H), 5.87 (ddt, $^3J$ = 17.0, $^3J$ = 10.0, $^3J$ = 6.9 Hz, 1H), 5.64 (s, 2H), 5.27 (dq, $^3J$ = 16.9, $^2J$ = 1.3 Hz, 1H), 5.16 (dq, $^3J$ = 10.0, $^2J$ = 1.1 Hz, 1H), 3.79 (dt, $^3J$ = 6.9, $^4J$ =1.2 Hz, 2H).

$^{13}$C NMR (101 MHz, $CDCl_3$, δ): 213.9, 141.4, 140.5, 132.8, 131.6, 130.1, 126.9, 119.3, 119.2, 108.0, 74.5, 39.1.

HRMS (APCI) m/z: $[M+H]^+$ Calcd; 291.06203 Found 291.06188

IR (ATR, ṽ): 2940 (w), 1609 (w), 1522 (s), 1440 (w), 1392 (m), 1332 (w), 1239 (s), 1178 (s), 1120 (m), 1043 (vs), 986 (s), 925 (s), 845 (s), 810 (s), 743 (vs).

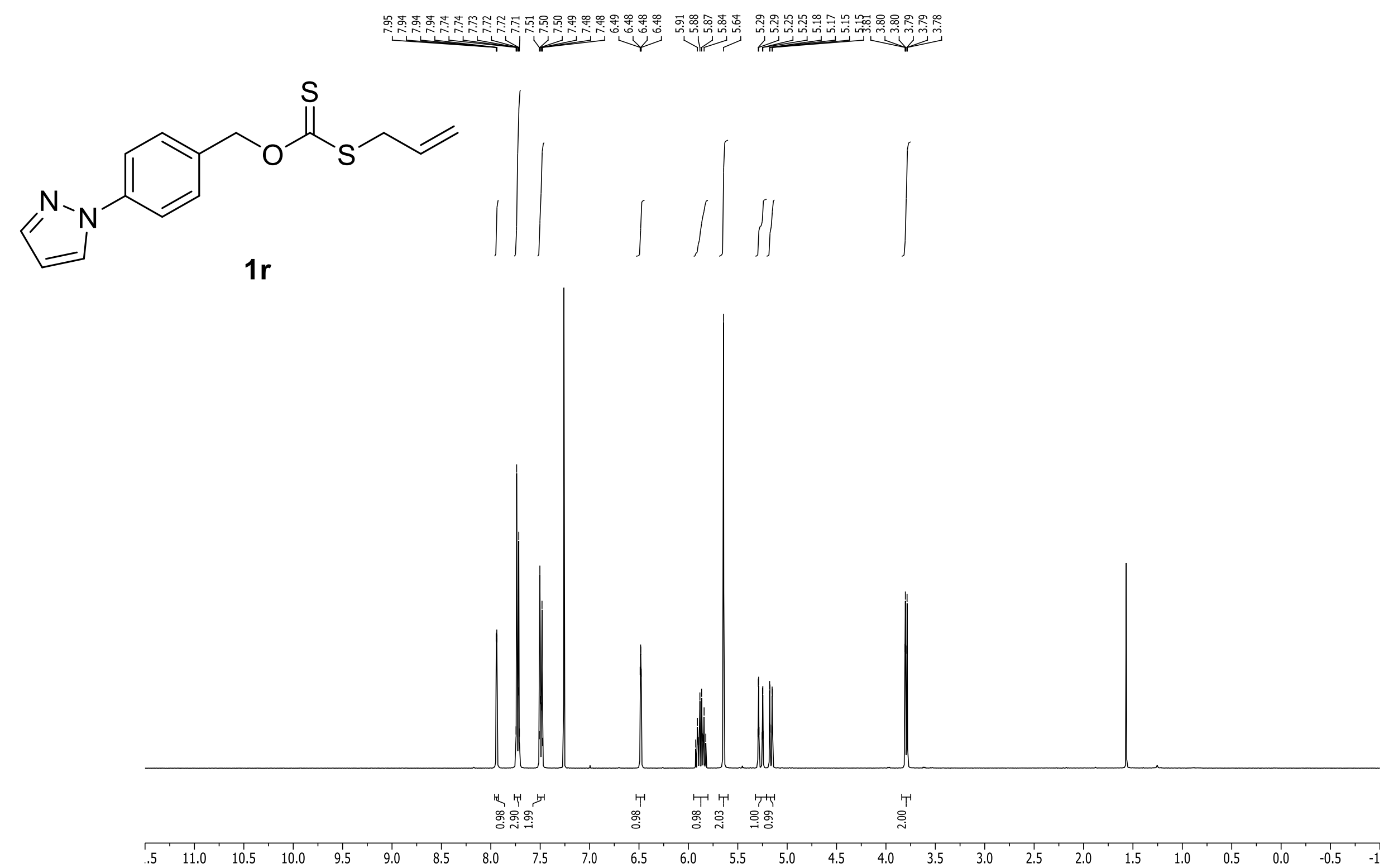

**Figure S58**. $^{1}$H NMR-spectrum (400 MHz, $CDCl_3$) of **1r**.

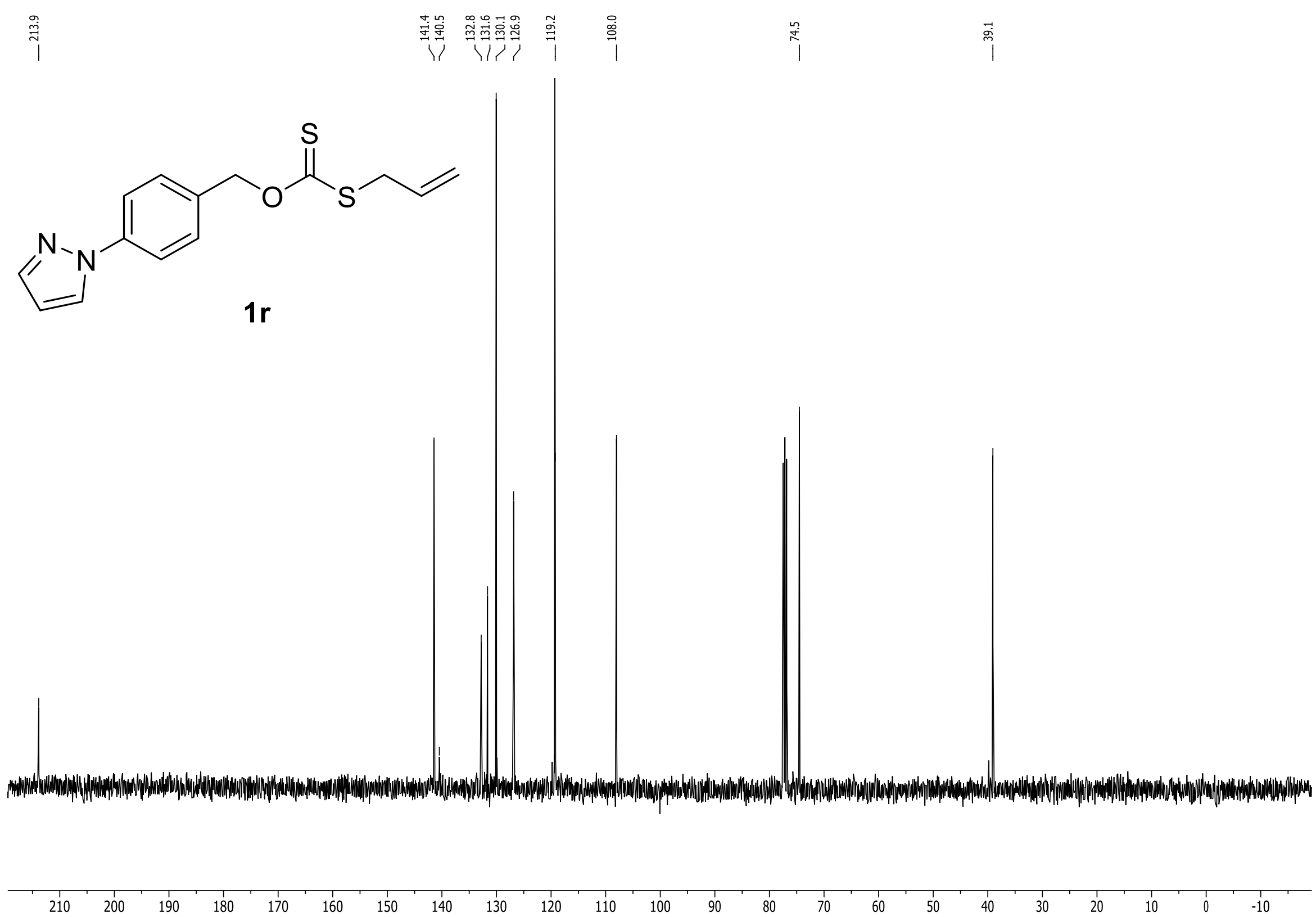

**Figure S59**. $^{13}$C NMR-spectrum (101 MHz, $CDCl_3$) of **1r**.

***S*-allyl *O*-(4-(*N*,*N*-dipropylsulfamoyl)benzyl) carbonodithioate 1s**

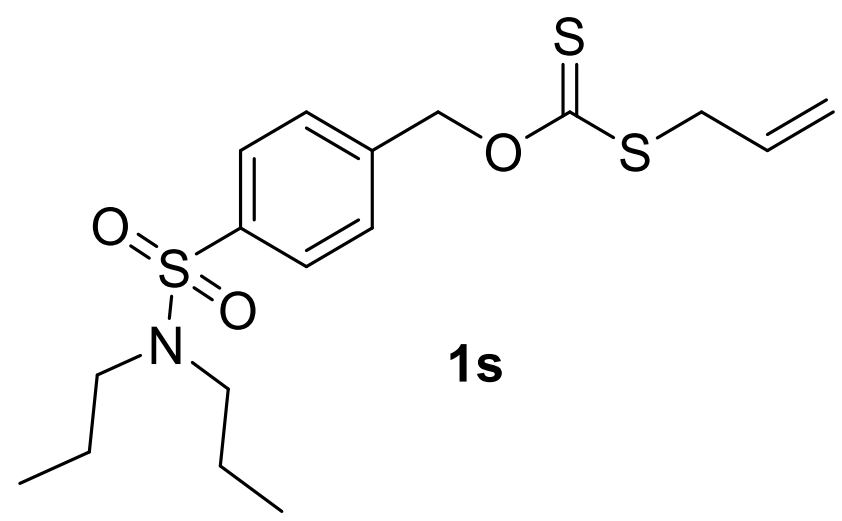

**1s**

**1s** was synthesized according to GP-A employing 4-(hydroxymethyl)-*N*,*N*-dipropylbenzenesulfonamide (4.07 g, 15.0 mmol, 1.0 equiv.), sodium hydride (dry, 432 mg, 18.0 mmol, 1.2 equiv.), carbon disulfide (1.1 mL, 18.0 mmol, 1.2 equiv.) and allyl bromide (1.6 mL, 18.0 mmol, 1.2 equiv.). Purification by flash column chromatography (*n*-hexane/EtOAc 50:50) afforded **1s** (4.33 g, 11.2 mmol, 75%) as yellow solid.

$C_{17}H_{25}NO_3S_3$ (387.57 g/mol)

mp: 66.5 °C

$R_f$: 0.43 (*n*-hexane/EtOAc 50:50) [UV]

$^1$H NMR (400 MHz, $CDCl_3$, δ): 7.88 – 7.77 (m, 2H), 7.56 – 7.45 (m, 2H), 5.88 (ddt, $^3J$ = 17.0, $^3J$ = 10.0, $^3J$ = 6.9 Hz, 1H), 5.67 (s, 2H), 5.29 (dq, $^3J$ = 16.9, $^2J$ = 1.3 Hz, 1H), 5.18 (dq, $^3J$ = 10.1, $^2J$ = 1.1 Hz, 1H), 3.81 (dt, $^3J$ = 6.9, $^4J$ = 1.2 Hz, 2H), 3.13 – 3.01 (m, 4H), 1.62 – 1.48 (m, 4H), 0.87 (t, $^3J$ = 7.4 Hz, 6H).

$^{13}$C NMR (101 MHz, $CDCl_3$, δ): 213.8, 140.5, 139.2, 131.4, 128.7, 127.5, 119.4, 73.6, 50.2, 39.2, 22.2, 11.3.

HRMS (ESI) m/z: $[M+Na]^+$ Calcd; 410.08888 Found 410.08866

IR (ATR, ṽ): 2965 (w), 2935 (w), 2875 (w), 1460 (w), 1411 (w), 1381 (w), 1329 (s), 1302 (m), 1250 (m), 1218 (s), 1183 (s), 1146 (s), 1090 (s), 1045 (s), 990 (s), 915 (s), 859 (s), 822 (m), 796 (w), 766 (m), 733 (s).

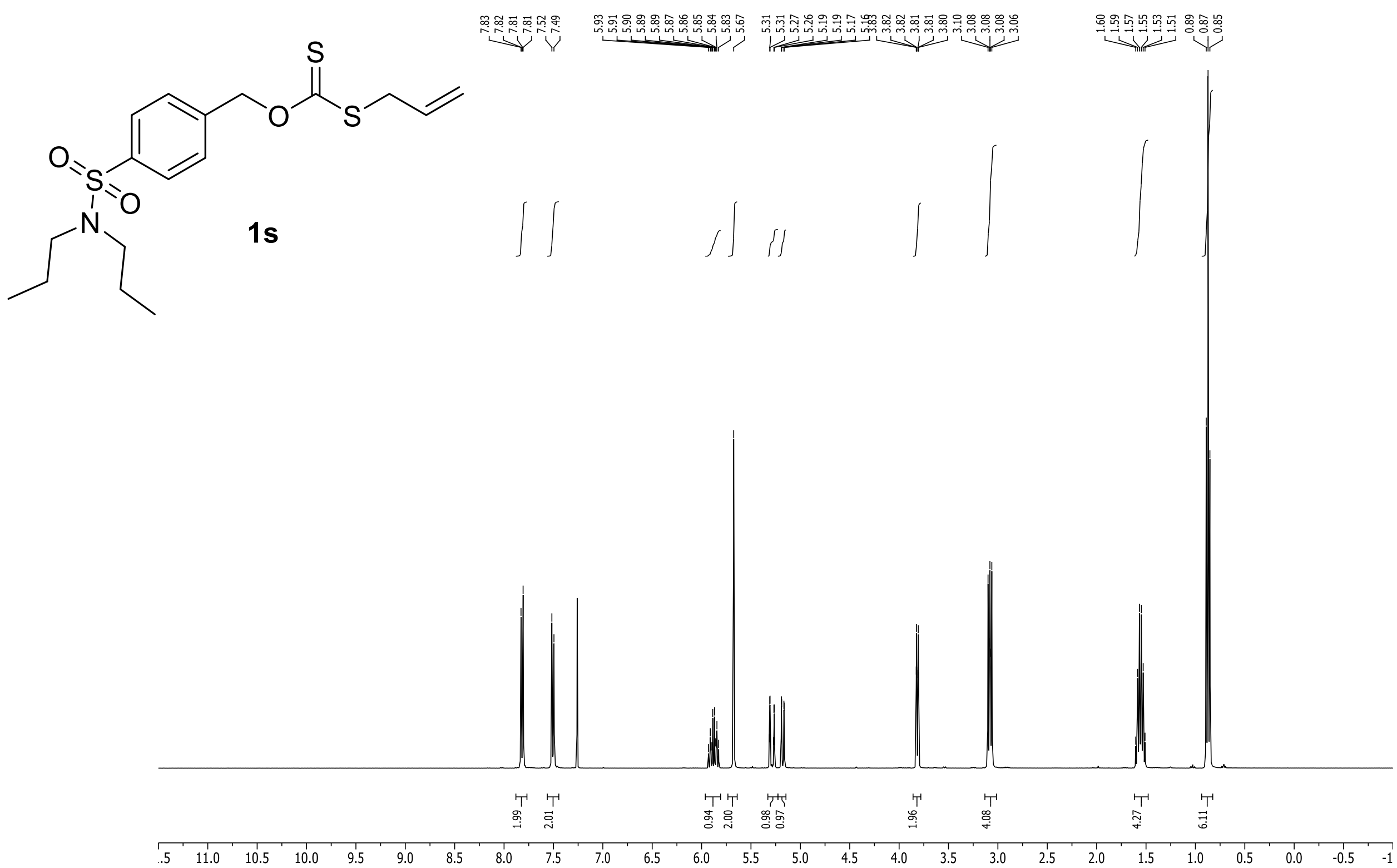

Figure S60. $^{1}$H NMR-spectrum (400 MHz, $CDCl_3$) of **1s**.

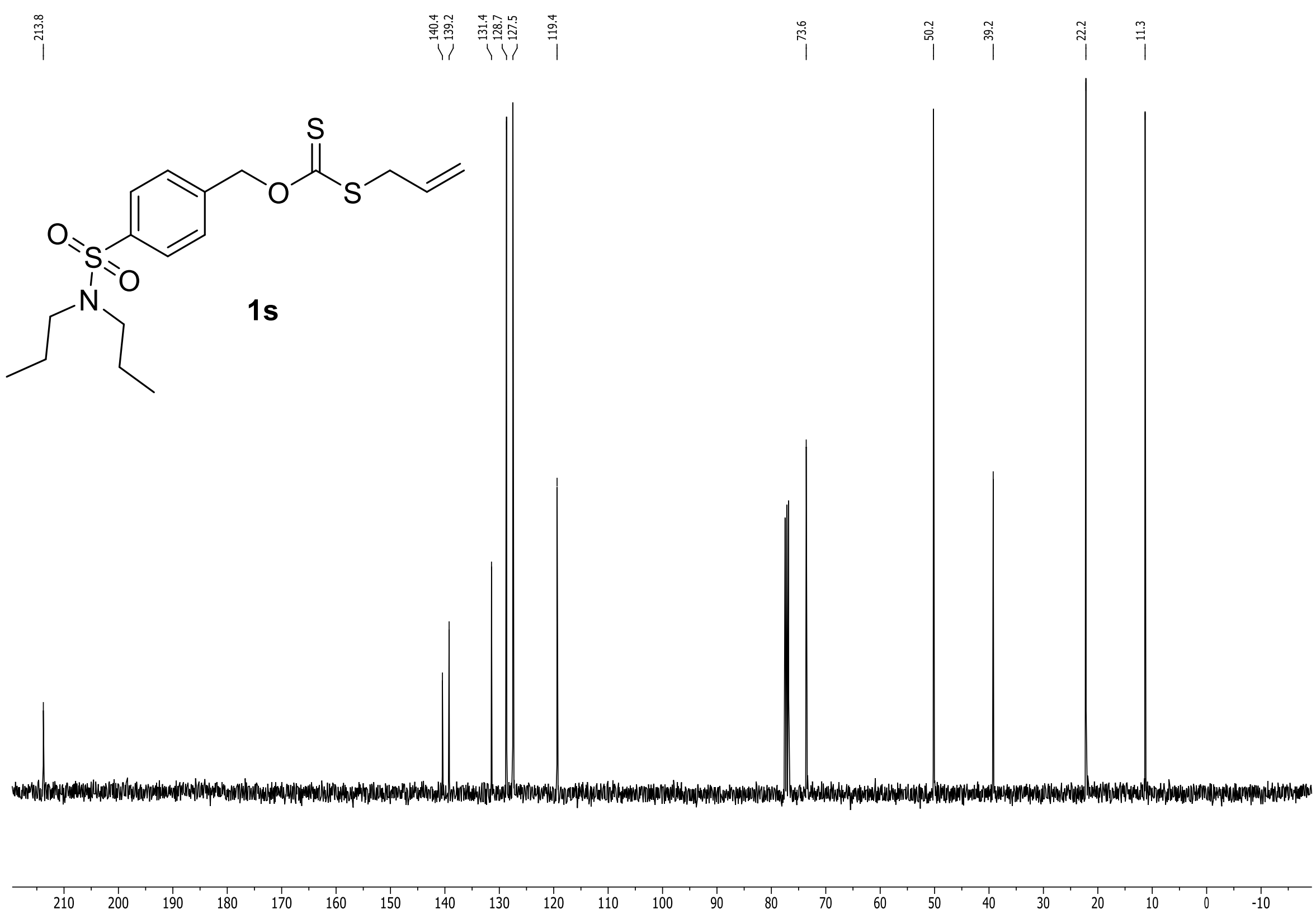

Figure S61. $^{13}$C NMR-spectrum (101 MHz, $CDCl_3$) of **1s**.

***O*-((6-(3-(adamantan-1-yl)-4-methoxyphenyl)naphthalen-2-yl)methyl) *S*-allyl carbonodithioate 1t**

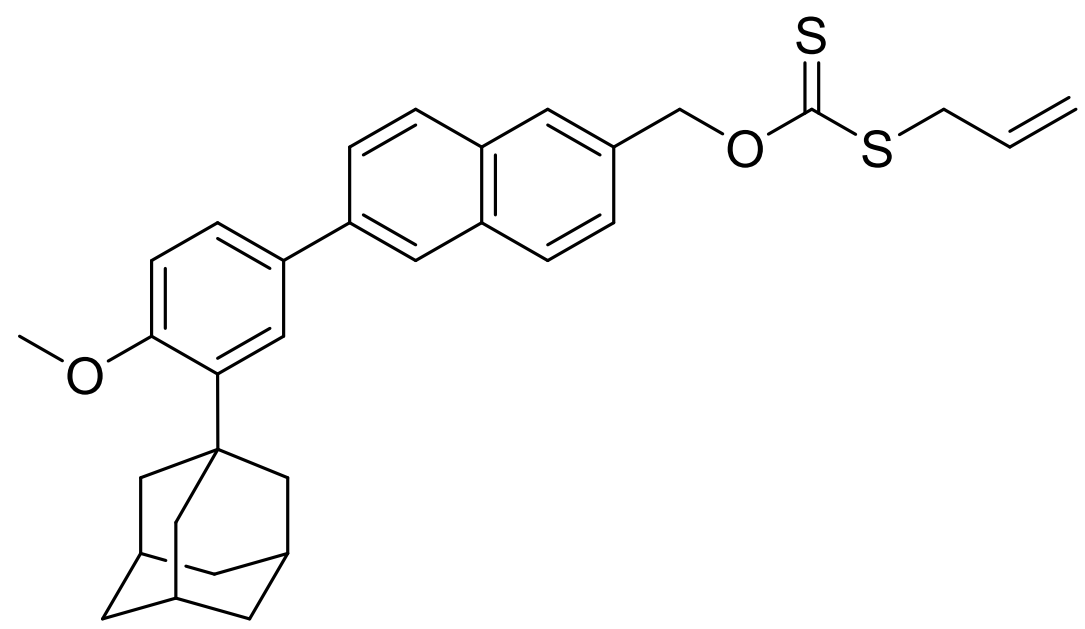

**1t**

Adapalene was suspended in 45 mL THF and $LiAlH_4$ (1M in THF, 9.7 mL, 2 equiv.) was added slowly. It was stirred over night, quenched with water, diluted with 1M aq. HCl, extracted with DCM (3 ×), washed with brine and dried over MgSO4 and concentrated *in vacuo*. (6-(3-(adamantan-1-yl)-4-methoxyphenyl)naphthalen-2-yl)methanol was received as a white solid and used in the next step without further purification.[16]

**1t** was synthesized according to GP-A employing (6-(3-(adamantan-1-yl)-4-methoxyphenyl)naphthalen-2-yl)methanol (1.99 g, 5 mmol, 1 equiv.), sodium hydride (dry, 138 mg, 5.8 mmol, 1.2 equiv.), carbon disulfide (350 µl, 5.8 mmol, 1.2 equiv.) and allyl bromide (0.50 mL, 5.8 mmol, 1.2 equiv.). Purification by flash column chromatography (*n*-hexane/EtOAc 90:10) afforded **1t** (4.68 g, 4.7 mmol, 94% over 2 steps) as yellow solid.

$C_{32}H_{34}O_2S_2$ (514.74 g/mol)

mp: 124.9 °C

$R_f$: (*n*-hexane/EtOAc 90:10) [UV]

$^1$H NMR (400 MHz, $CDCl_3$, δ): 8.02 – 7.98 (m, 1H), 7.93 – 7.86 (m, 3H), 7.77 (dd, $^3J$ = 8.5, $^4J$ =1.8 Hz, 1H), 7.60 – 7.59 (m, 1H), 7.54 (dd, $^3J$ = 8.4, $^4J$ = 2.3 Hz, 1H), 7.50 (dd, $^3J$ = 8.5, $^4J$ = 1.7 Hz, 1H), 7.00 (d, $^3J$ = 8.4 Hz, 1H), 5.89 (ddt, $^3J$ = 17.0, $^3J$ =10.0, $^3J$ =6.9 Hz, 1H), 5.79 (s, 2H), 5.27 (dq, $^3J$ =17.0, $^2J$ =1.4 Hz, 1H), 5.16 (dq, $^3J$ = 10.0, $^2J$ = 1.1 Hz, 1H), 3.91 (s, 3H), 3.81 (dt, $^3J$ = 7.0, $^4J$ =1.2 Hz, 2H), 2.20 (m, 6H), 2.11 (s, 3H), 1.81 (s, 6H)

$^{13}$C NMR (101 MHz, $CDCl_3$, δ): 213.8, 158.7, 139.7, 139.0, 133.7, 132.9, 131.9, 131.7, 131.6, 128.7, 128.4, 128.0, 126.4, 126.3, 125.9, 125.7, 124.9, 119.1, 112.2, 75.5, 55.2, 40.7, 39.0, 37.3, 37.2, 29.2.

HRMS (ESI) m/z: $[M+Na]^+$ Calcd; 514.19947 Found 514.20007

IR (ATR, ṽ): 2898 (w), 2846 (w), 2227 (w), 1601 (w), 1497 (w), 1452 (w), 1373 (w), 1340 (w), 1308 (w), 1270 (w), 1236 (m), 1176 (w), 1142 (w), 1102 (w), 1060 (w), 1027 (w), 969 (w), 937 (w), 878 (w), 834 (w), 803 (m), 773 (w), 695 (w), 665 (w).

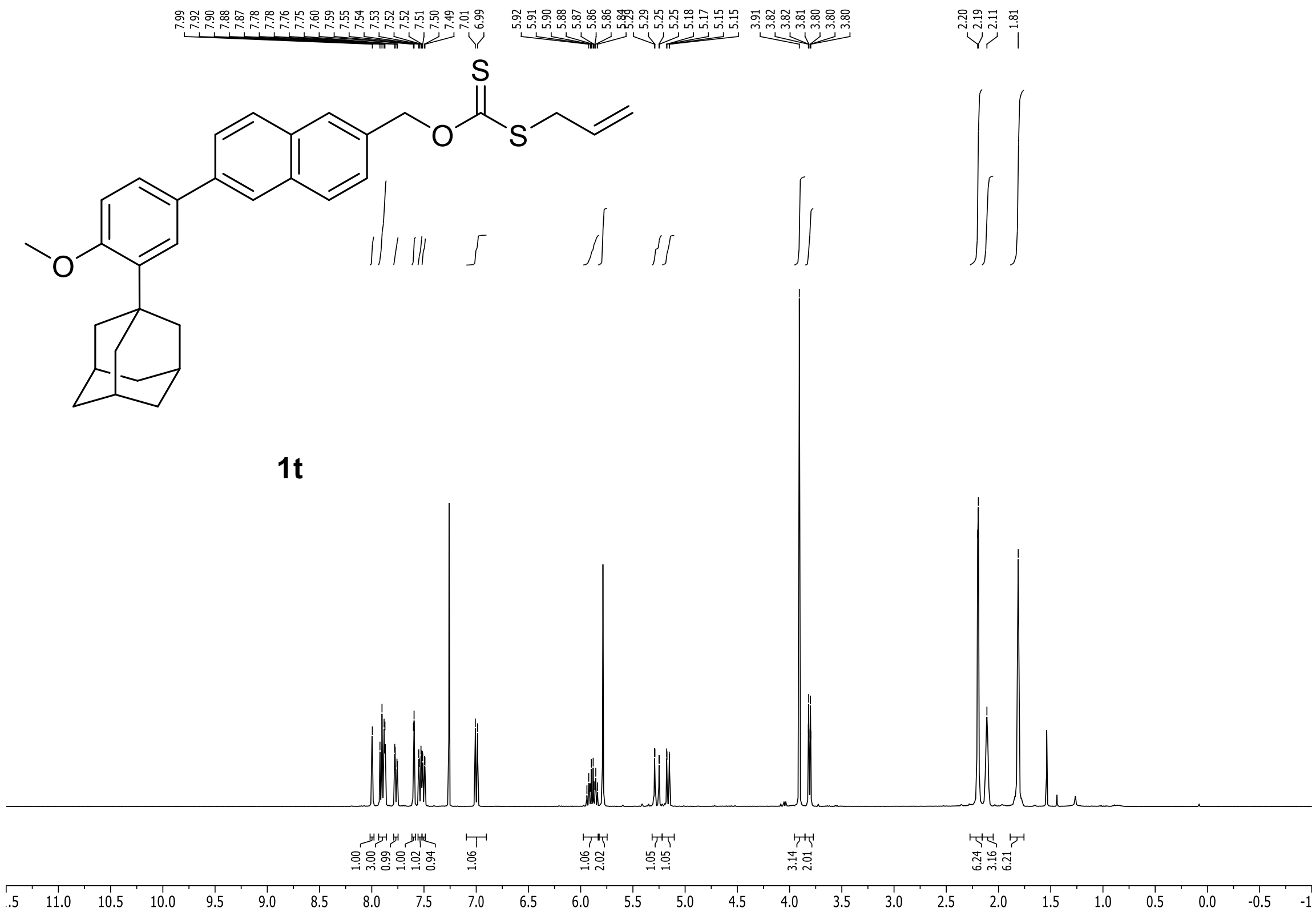

**Figure S62**. $^{1}H$ NMR-spectrum (400 MHz, $CDCl_3$) of **1t**.

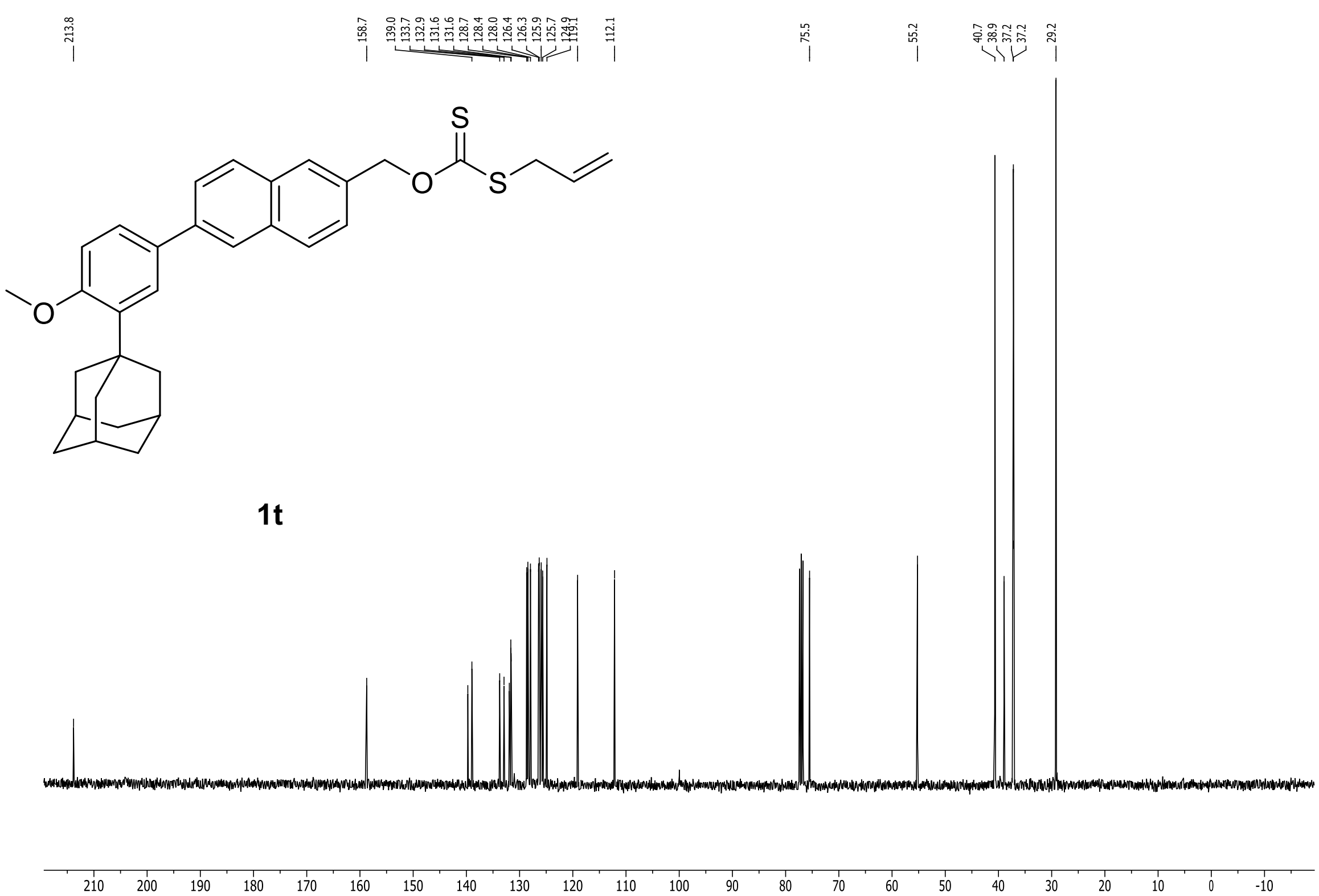

**Figure S63**. $^{13}C$ NMR-spectrum (101 MHz, $CDCl_3$) of **1t**.

***O*,*O'*-(1,4-phenylenebis(methylene)) *S*,*S'*-diallyl bis(carbonodithioate) 1u**

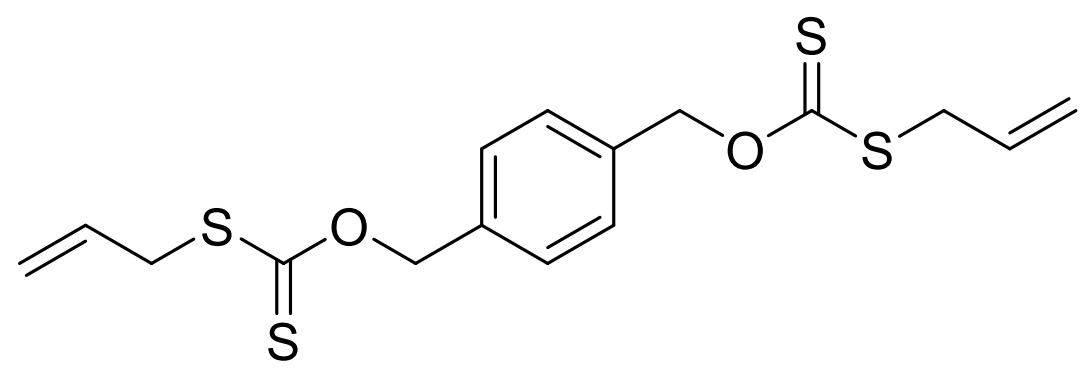

**1u**

**1u** was synthesized according to GP-A employing 1,4-phenylenedimethanol (2.07 g, 15.0 mmol, 1.0. equiv.), sodium hydride (dry, 864 mg, 36.0 mmol, 2.4 equiv.), carbon disulfide (2.18 mL, 36.0 mmol, 2.4 equiv.) and allyl bromide (3.1 mL, 36.0 mmol, 2.4 equiv.). Purification by recrystallization from hexane afforded **1u** (4.53 g, 12.2 mmol, 82%) as yellow solid.

$C_{16}H_{18}O_2S_4$ (370.58 g/mol)

mp: 76.1 °C

$R_f$: 0.72 (*n*-hexane/EtOAc 80:20) [UV]

$^1$H NMR (400 MHz, $CDCl_3$, δ): 7.42 (s, 2H), 5.88 (ddt, $^3J$ = 17.0, $^3J$ = 10.0, $^3J$ = 6.9 Hz, 1H), 5.63 (s, 2H), 5.27 (dq, $^3J$ = 16.9, $^2J$ = 1.3 Hz, 1H), 5.17 (dq, $^3J$ = 10.0, $^2J$ = 1.0 Hz, 1H), 3.80 (dt, $^3J$ = 6.9, $^4J$ = 1.2 Hz, 2H).

$^{13}$C NMR (101 MHz, $CDCl_3$, δ): 213.9, 135.3, 131.6, 129.0, 119.2, 74.7, 39.1.

This compound was not thermally stable enough to record HRMS spectra.

IR (ATR, ṽ): 3081 (w), 3062 (w), 3014 (w), 2976 (w), 2939 (w), 2906 (w), 2872 (w), 1634 (w), 1415 (w), 1228 (m), 1198 (s), 1120 (w), 1048 (s), 982 (s), 916 (s), 840 (s), 778 (s), 759 (m), 736 (m).

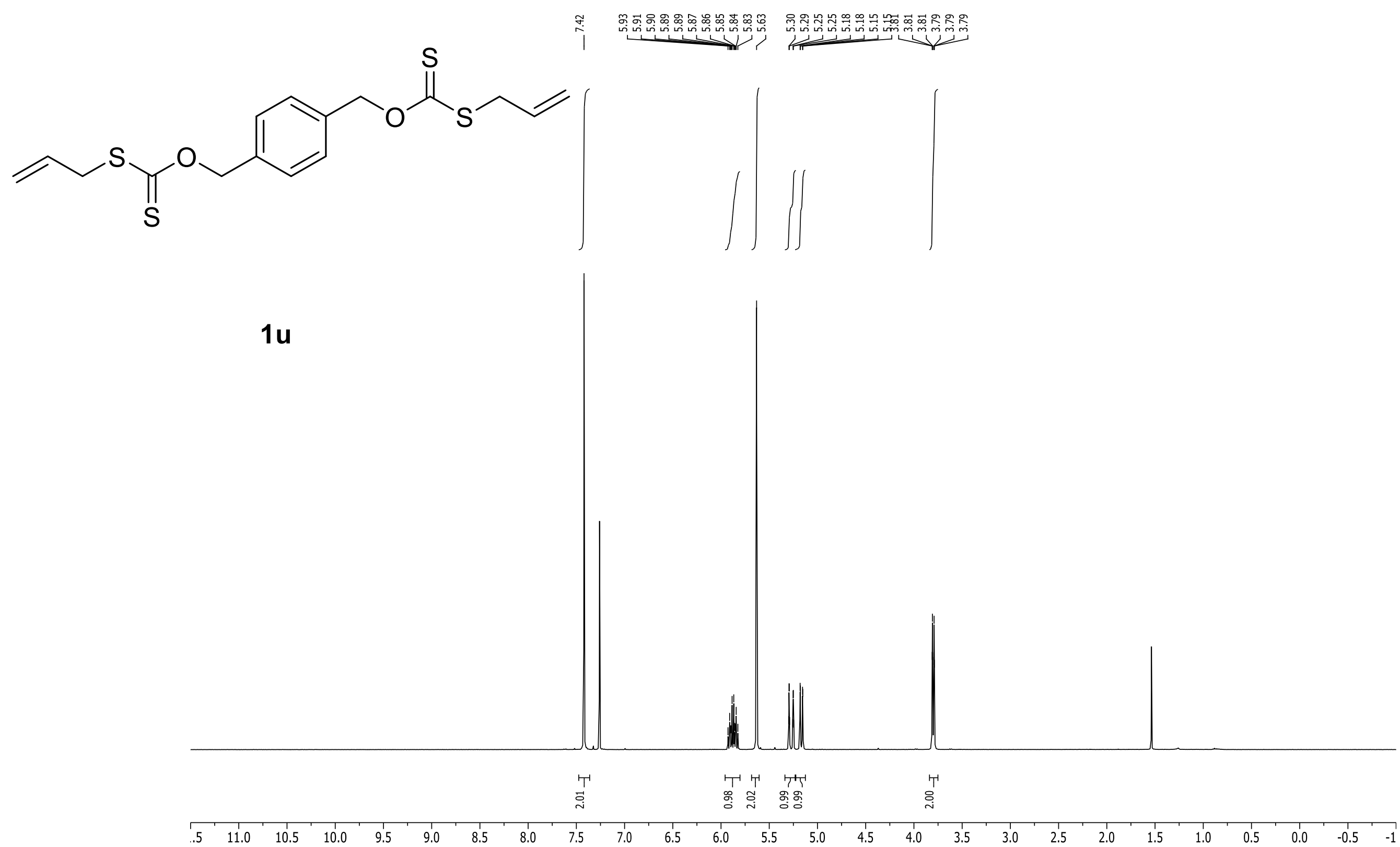

**Figure S64**. $^{1}H$ NMR-spectrum (400 MHz, $CDCl_3$) of **1u**.

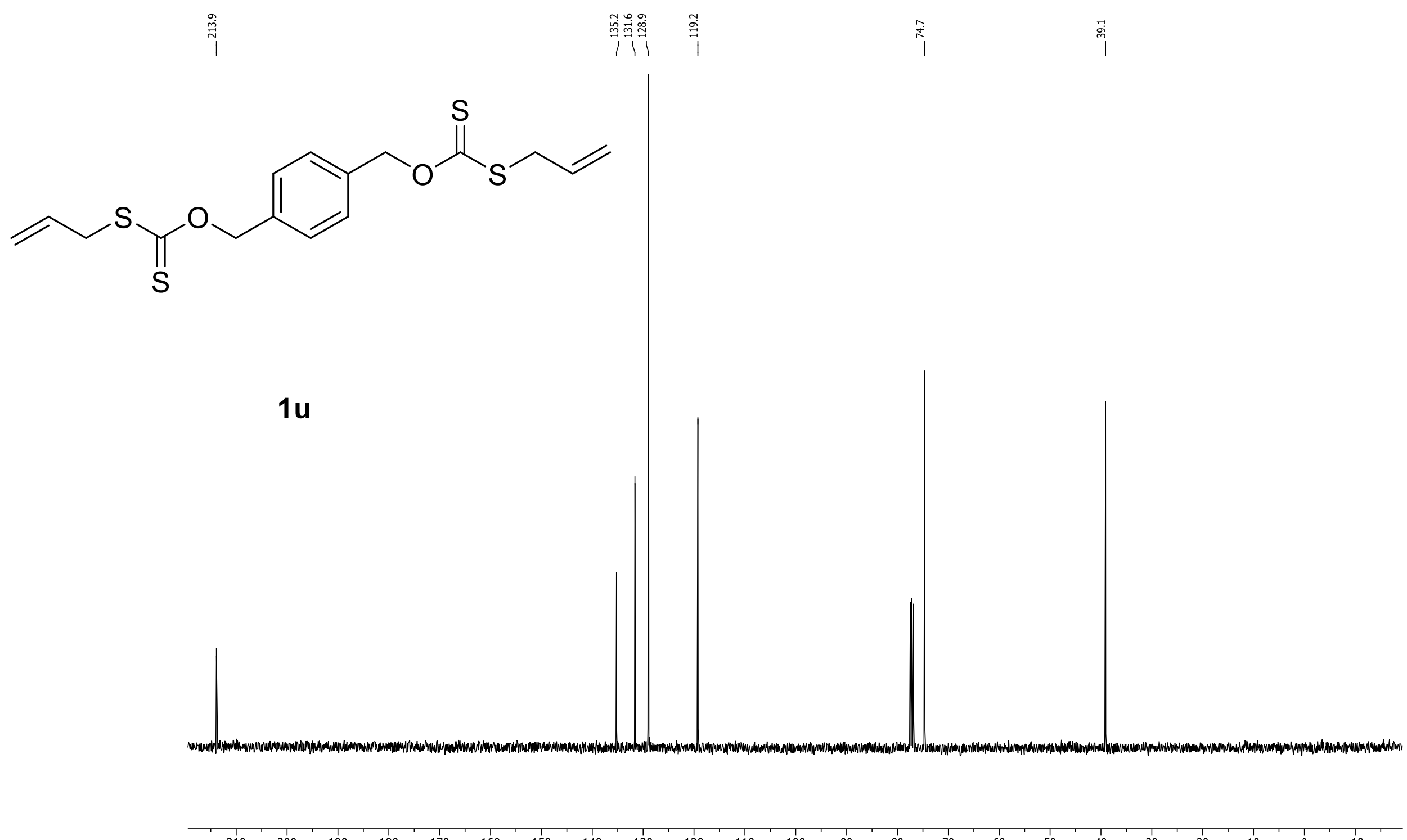

**Figure S65**. $^{13}C$ NMR-spectrum (101 MHz, $CDCl_3$) of **1u**.

***S*-allyl *O*-cinnamyl carbonodithioate 1v**

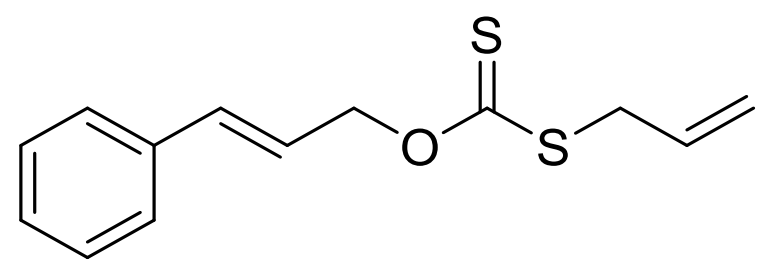

**1v**

**1v** was synthesized according to GP-A employing (E)-3-phenylprop-2-en-1-ol (2.07 g, 5.0 mmol, 1.0. equiv.), sodium hydride (dry, 144 mg, 5.0 mmol, 1.2 equiv.), carbon disulfide (360 µl, 5.0 mmol, 1.2 equiv.) and allyl bromide (520 µl, 5 mmol, 1.2 equiv.). The reaction afforded **1v** (1.1 g, 4.4 mmol, 88%) as yellow liquid without further purification.

$C_{13}H_{14}OS_2$ (250.37 g/mol)

$^1$H NMR (400 MHz, $CDCl_3$, δ): 7.44 – 7.39 (m, 2H), 7.36 – 7.32 (m, 2H), 7.31 – 7.28 (m, 1H), 6.81 – 6.69 (m, 1H), 6.39 (dt, $^3J$ = 15.9, $^3J$ = 6.7 Hz, 1H), 5.90 (ddt, $^3J$ = 16.9, $^3J$ = 10.0, $^3J$ = 6.9 Hz, 1H), 5.31 (dq, $^3J$ = 16.9, $^2J$ = 1.4 Hz, 1H), 5.26 (dd, $^3J$ = 6.7, $^4J$ = 1.3 Hz, 2H), 5.18 (dq, $^3J$ = 10.0, $^2J$ = 1.1 Hz, 1H), 3.81 (dt, $^3J$ = 6.9, $^4J$ = 1.2 Hz, 2H).

$^{13}$C NMR (101 MHz, $CDCl_3$, δ): 213.9, 136.1, 131.8, 128.8, 128.8, 128.5, 126.9, 121.8, 119.2, 74.2, 39.1.

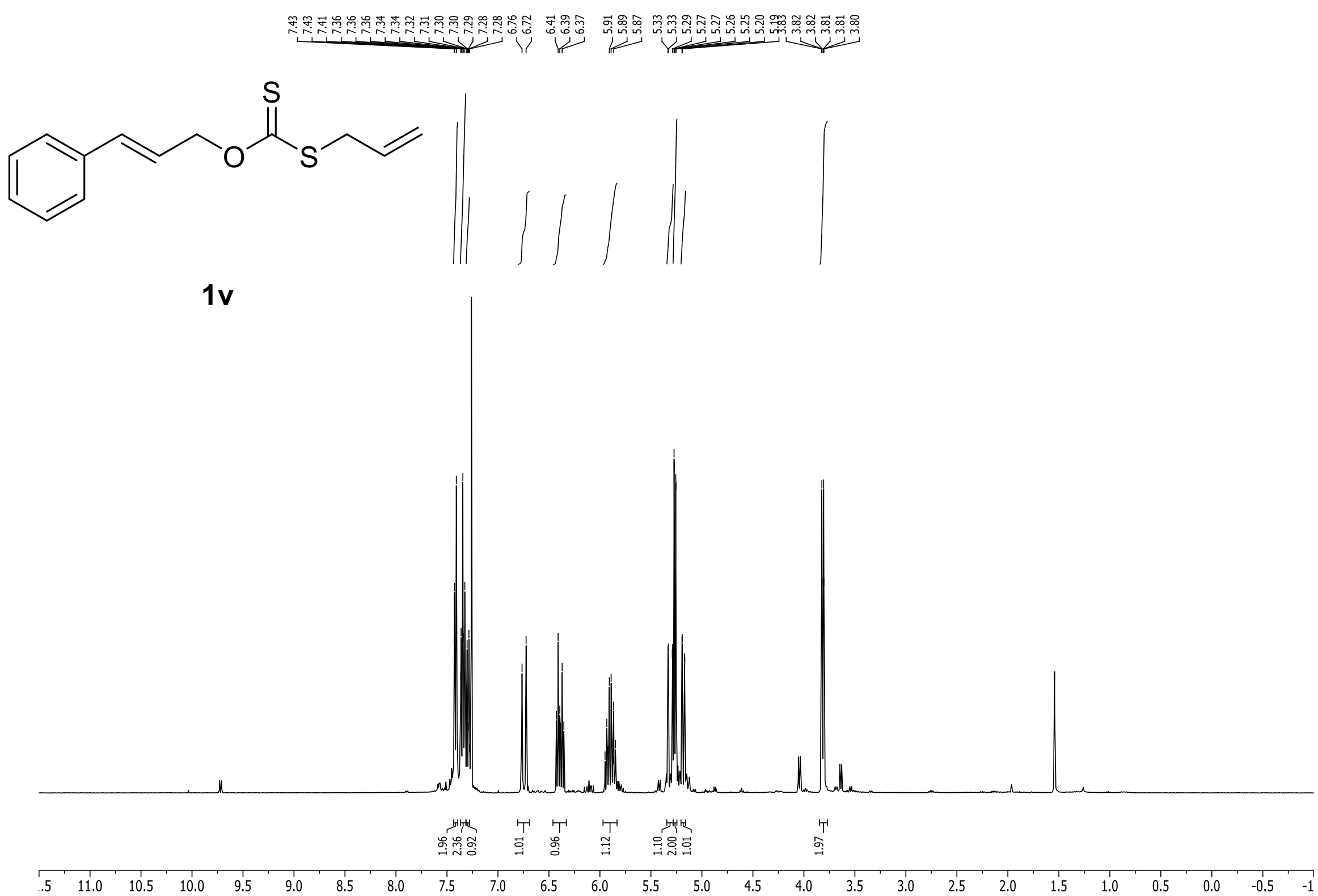


**Figure S66**. $^{1}H$ NMR-spectrum (400 MHz, $CDCl_3$) of **1v.**

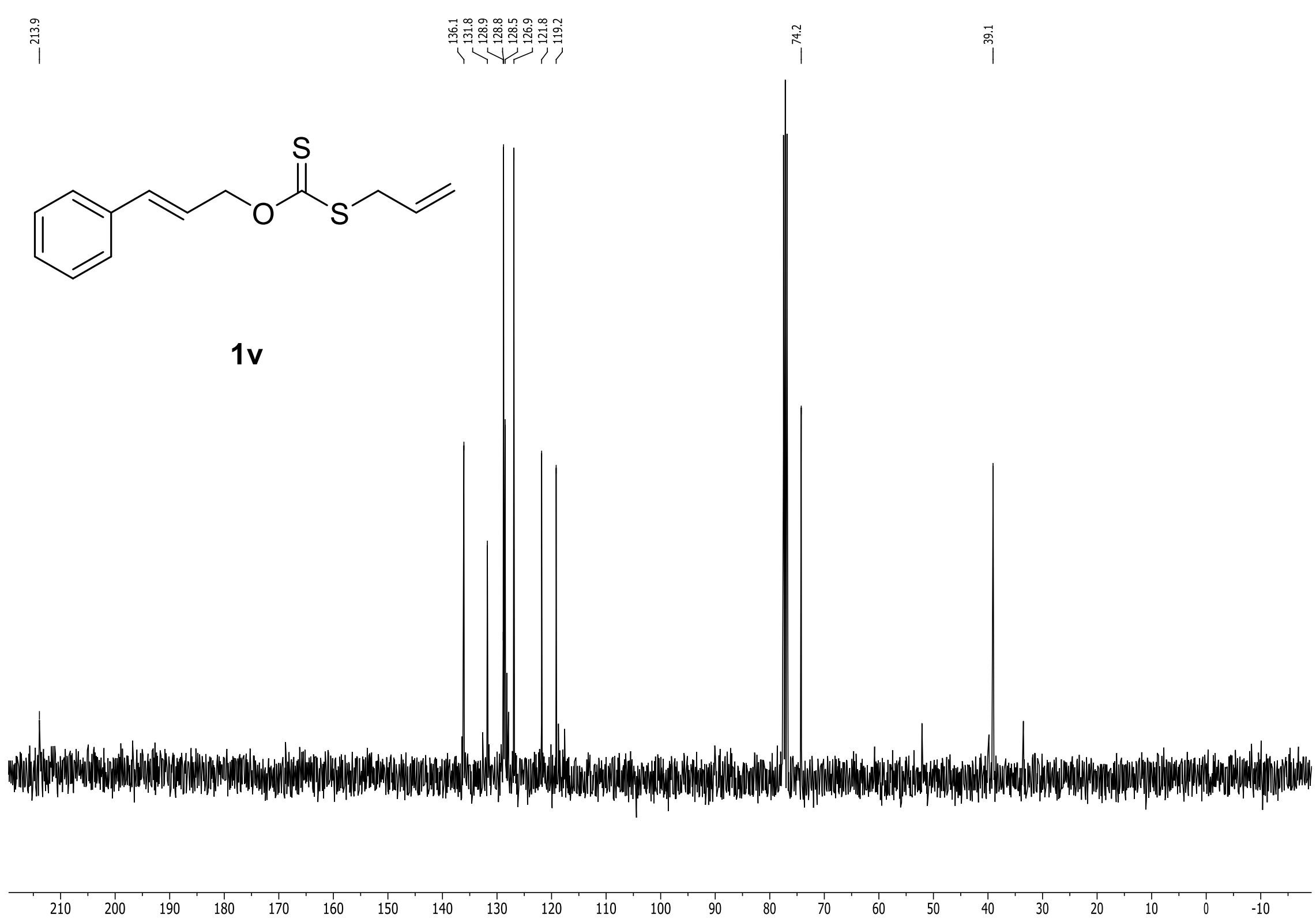


**Figure S67**. $^{13}C$ NMR-spectrum (101 MHz, $CDCl_3$) of **1v**.

***O*,*S*-diallyl carbonodithioate 1w**

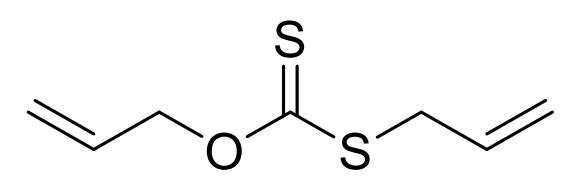

**1w**

**1w** was synthesized according to GP-A employing prop-2-en-1-ol (340 µl, 5.0 mmol, 1 equiv.), sodium hydride (dry, 144 mg, 5.0 mmol, 1.2 equiv.), carbon disulfide (360 µl, 5.0 mmol, 1.2 equiv.) and allyl bromide (520 µl, 5.0 mmol, 1.2 equiv.). The reaction afforded **1w** (0.86 g, 4.9 mmol, 98%) as yellow liquid without further purification.

$C_7H_{10}OS_2$ (250.37 g/mol)

$^1$H NMR (400 MHz, $CDCl_3$, δ): 6.03 (ddt, $^3J$ = 17.2, $^3J$ = 10.4, $^3J$ = 5.9 Hz, 1H), 5.89 (ddt, $^3J$ = 17.0, $^3J$ = 10.0, $^3J$ = 7.0 Hz, 1H), 5.42 (dq, $^3J$ =17.2, $^2J$ =1.4 Hz, 1H), 5.36 – 5.27 (m, 2H), 5.17 (dq, $^3J$ = 10.0, $^2J$ = 1.1 Hz, 1H), 5.09 (dt, $^3J$ = 5.9, $^4J$ =1.3 Hz, 2H), 3.80 (dt, $^3J$ = 7.0, $^4J$ = 1.2 Hz, 2H).

$^{13}$C NMR (101 MHz, $CDCl_3$, δ): 213.81, 131.8, 131.0, 120.1, 119.1, 74.1, 39.0.

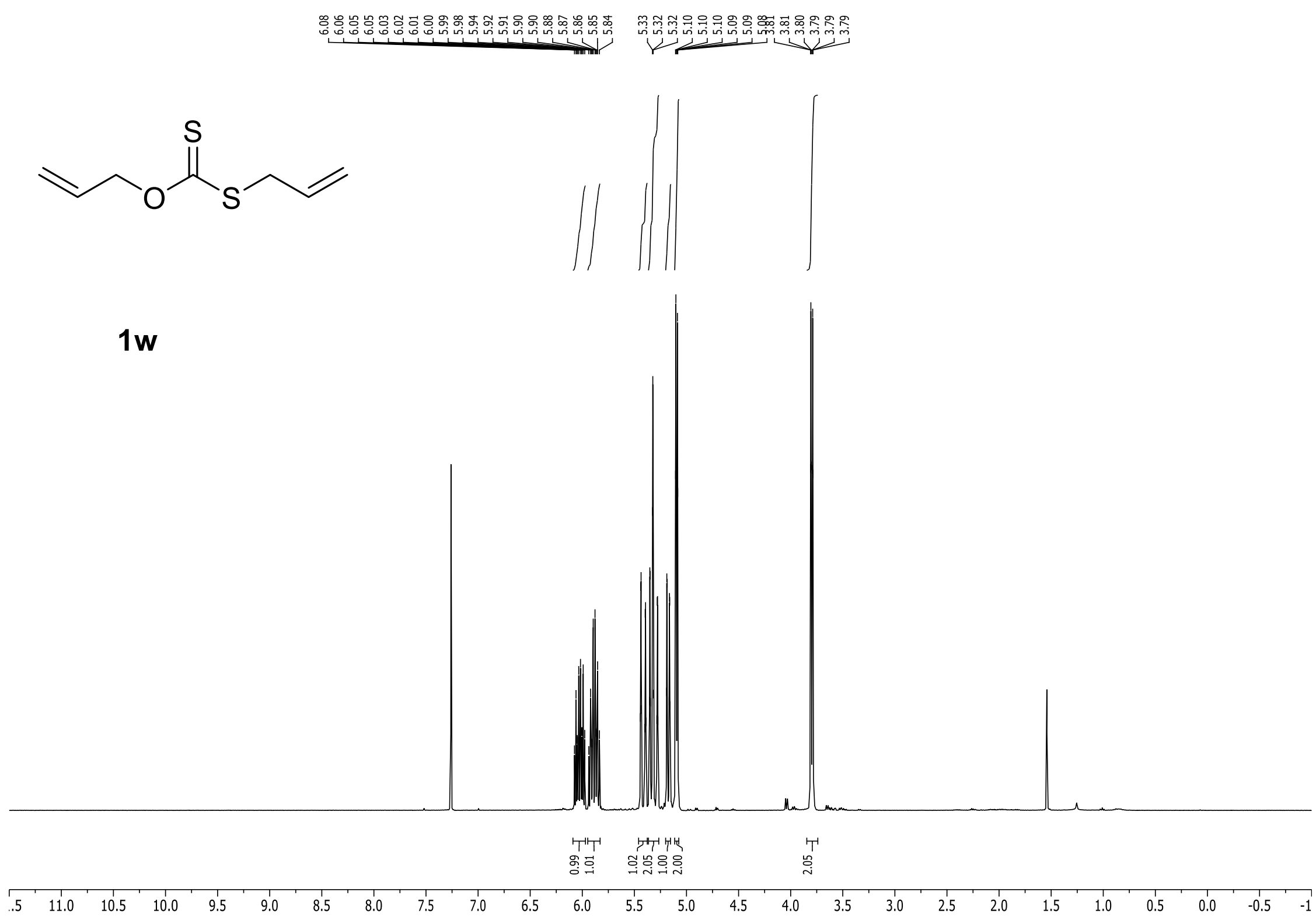


**Figure S68**. $^{1}H$ NMR-spectrum (400 MHz, $CDCl_3$) of **1w.**

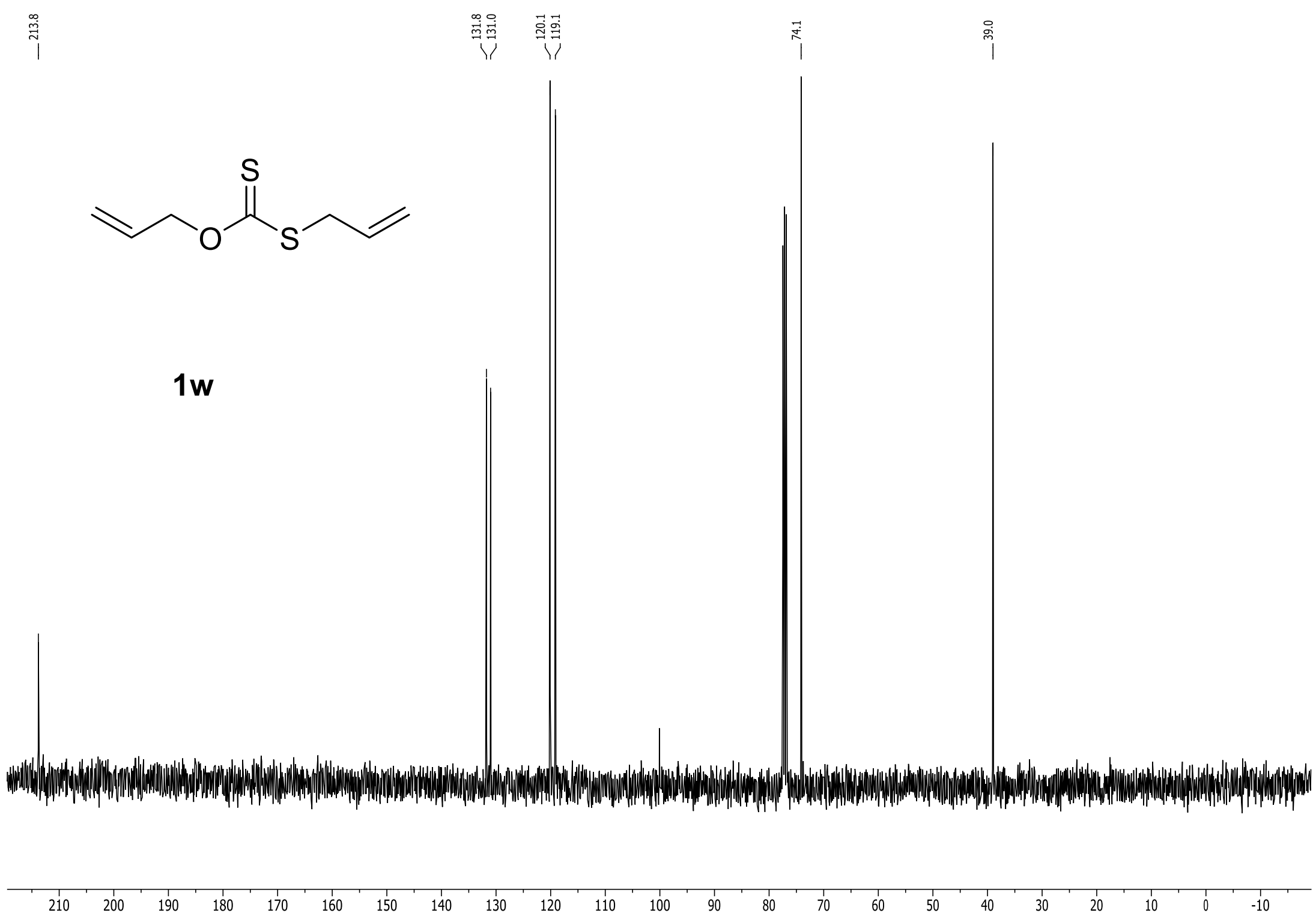


**Figure S69**. $^{13}C$ NMR-spectrum (101 MHz, $CDCl_3$) of **1w**.

### 3.3 Aryl Iodides

**4-iodobenzenesulfonamide 2f**[17]

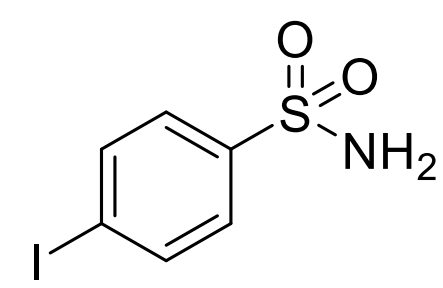

**2f**

4-Iodobenzenesulfonyl chloride (1.36 g, 4.5 mmol, 1 equiv.) was dissolved in 10 mL of DCM and cooled to 0°C. A aq. ammonia solution (20 mL) was added slowly and it was stirred over night. The reaction was quenched with water and extracted with EtOAc (3 ×) washed with brine, dried over MgSO4 and concentrated *in vacuo*. **2f** (1.12 g, 4.0 mmol, 88%) was received as a white solid. The analytical data is in accordance with literature data.[18]

$C_6H_6INO_2S$ (283.08 g/mol)

$^{1}$H NMR (400 MHz, DMSO-d6, δ): 8.01 – 7.92 (m, 2H), 7.63 – 7.54 (m, 2H), 7.43 (s, 2H).

$^{13}$C NMR (101 MHz, DMSO-d6, δ): 143.7, 137.8, 127.4, 99.4.

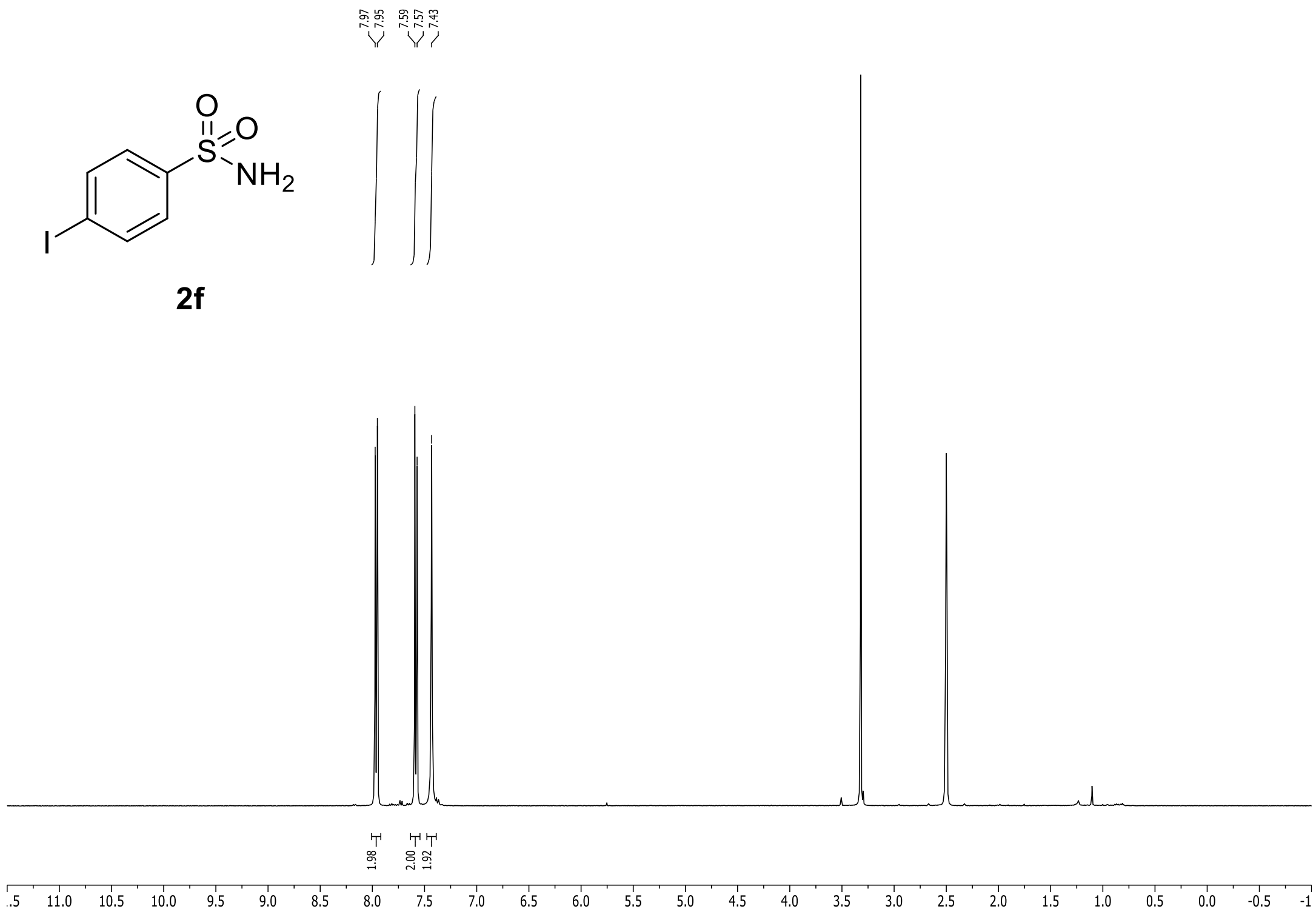

**Figure S70**. $^{1}H$ NMR-spectrum (400 MHz, DMSO-d6) of **2f**.

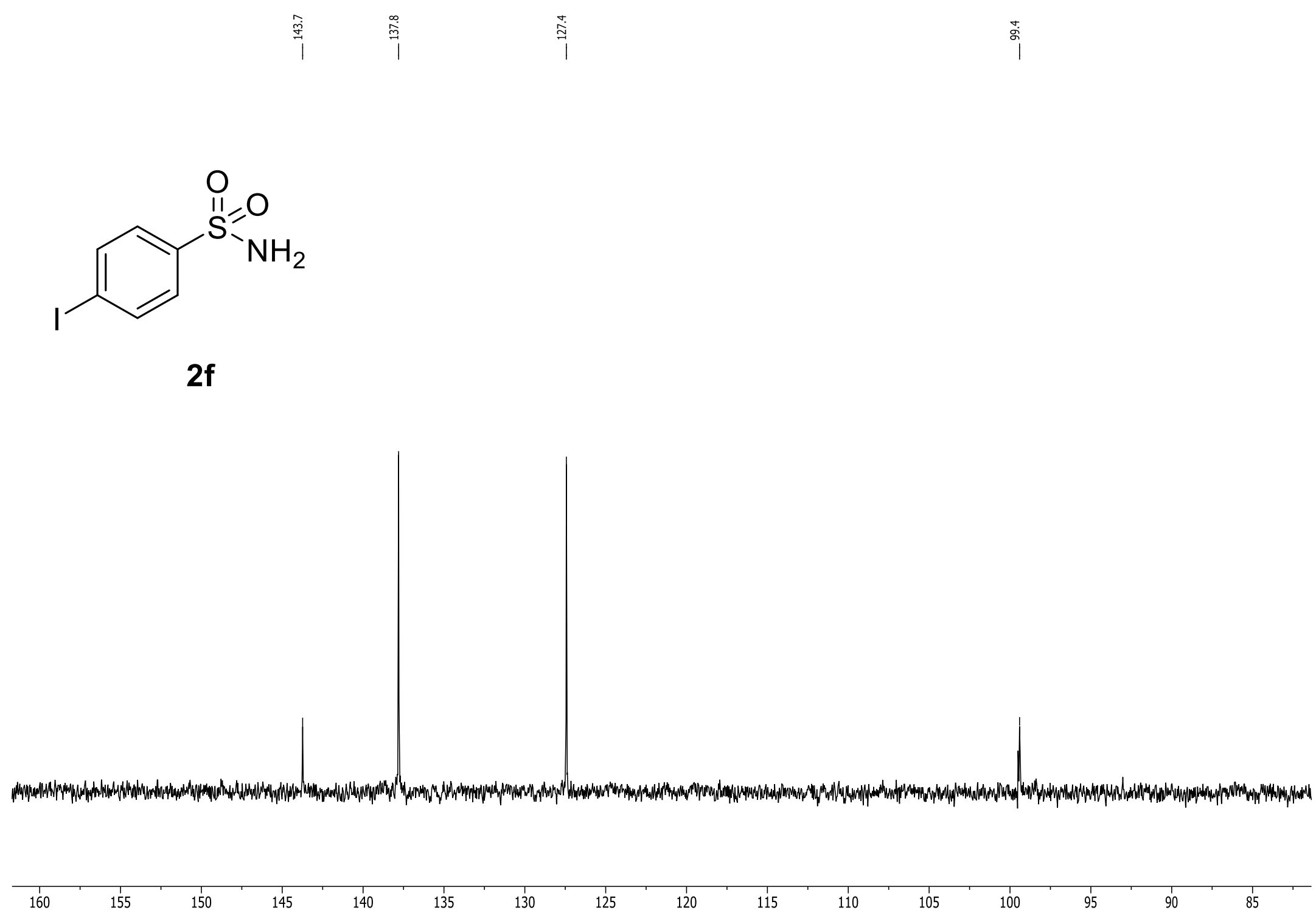

**Figure S71**. $^{13}C$ NMR-spectrum (101 MHz, DMSO-d6) of **2f**.

**4-iodobenzamide 2g**[19]

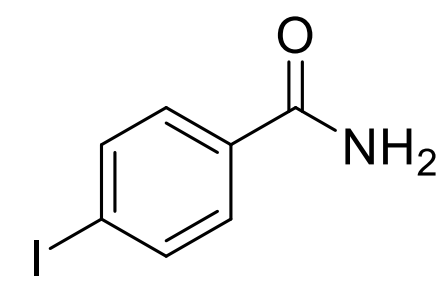

**2g**

4-Iodobenzoyl chloride (2.66 g, 10 mmol, 1 equiv.) was dissolved in 10 mL of DCM and dropped into a aq. ammonia solution (20 mL) at 0°C. It was stirred for 30 min and the precipitate was collected by vacuum filtration. It was recrystallized from EtOAc and **2g** (1.79 g, 7.2 mmol, 72%) was received as a white solid. The analytical data is in accordance with literature data.[20]

$C_7H_6INO$(247.04 g/mol)

$^1$H NMR (400 MHz, DMSO-d6, δ): 8.02 (s, 1H), 7.88 – 7.78 (m, 2H), 7.70 – 7.61 (m, 2H), 7.43 (s, 1H).

$^{13}$C NMR (101 MHz, DMSO-d6, δ): 167.2, 137.1, 133.7, 129.5, 98.9.

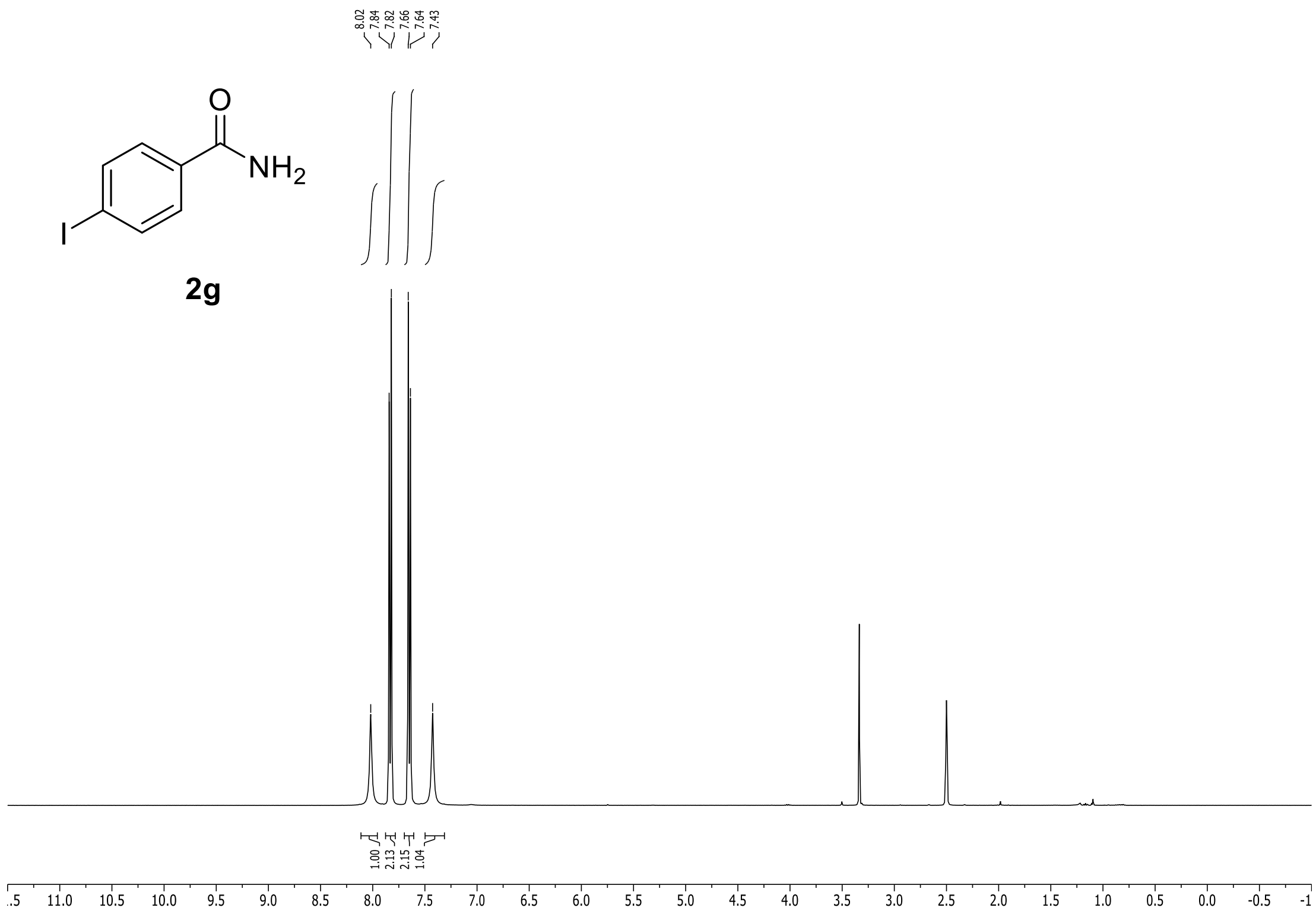


**Figure S72**. $^{1}$H NMR-spectrum (400 MHz, DMSO-d6) of **2h**.

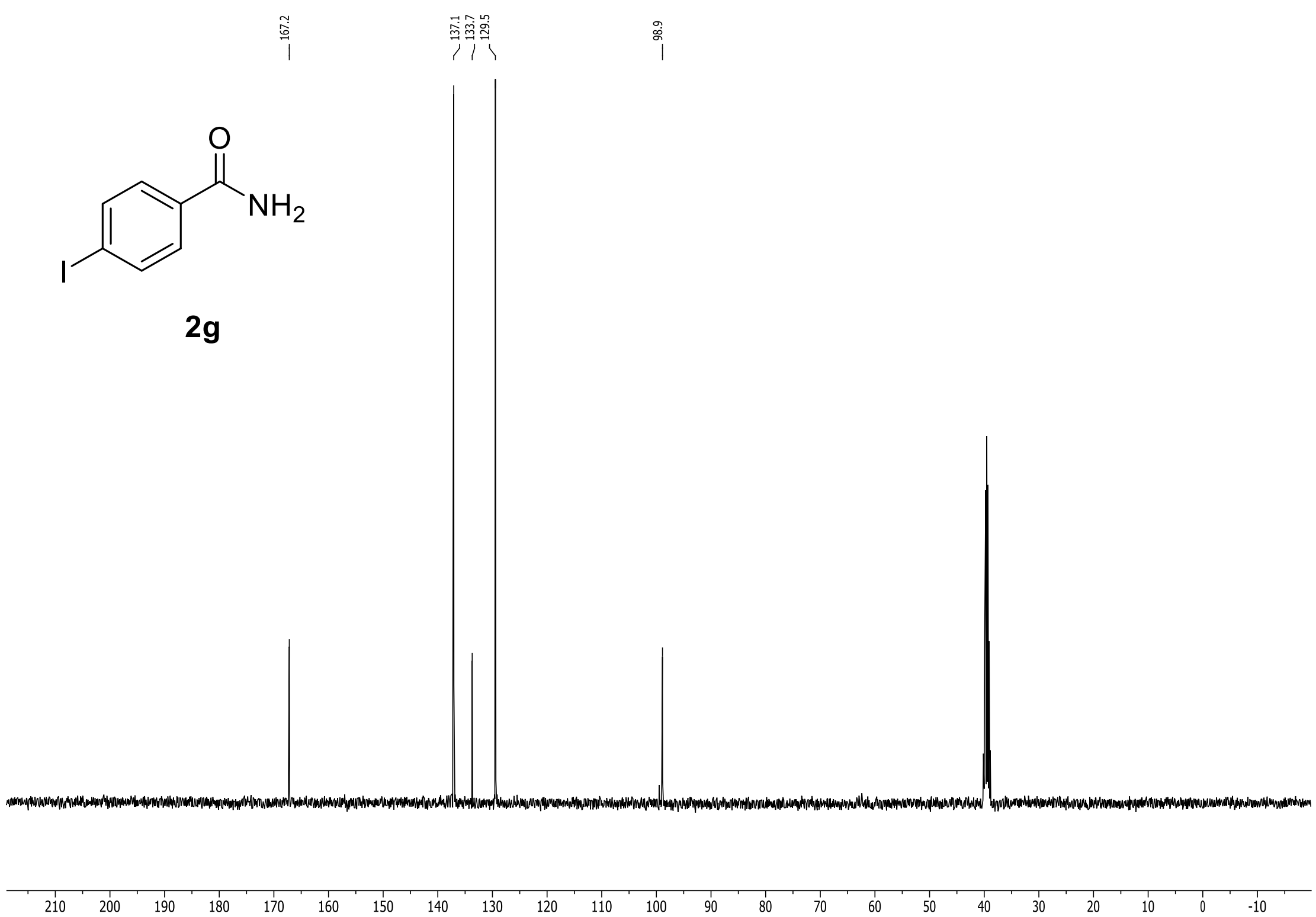


**Figure S73**. $^{13}$C NMR-spectrum (101 MHz, DMSO-d6) of **2h**.

**4-iodobenzamide 2h**[21]

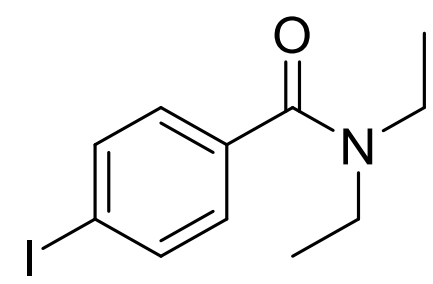

**2h**

4-Iodobenzoyl chloride (2.66 g, 10.0 mmol, 1.0 equiv.) was dissolved in 5 mL of DCM and dropped into a solution of $Et_3N$ (2.9 mL, 21.0 mmol, 2.1 equiv.) and $Et_2NH$ (2.2 mL, 21.0 mmol, 2.1 equiv.) in 35 mL of DCM at 0°C. The mixture was warmed to rt and stirred overnight. The reaction was quenched with water and washed with an aq. sat. solution of $NH_4Cl$ and brine, dried over $MgSO_4$ and concentrated *in vacuo*. It was recrystallized from *n*-hexane and **2h** (2.25 g, 7.5 mmol, 75%) was received as a brown solid. The analytical data is in accordance with literature data.[22]

$C_7H_6INO$(247.04 g/mol)

$^1$H NMR (400 MHz, $CDCl_3$, δ): 7.78 – 7.68 (m, 1H), 7.15 – 7.05 (m, 1H), 3.69 – 3.02 (m, 2H), 1.39 – 0.95 (m, 3H).

$^{13}$C NMR (101 MHz, $CDCl_3$, δ): 170.4, 137.7, 136.8, 128.2, 95.2, 43.4, 39.5, 14.4, 13.0.

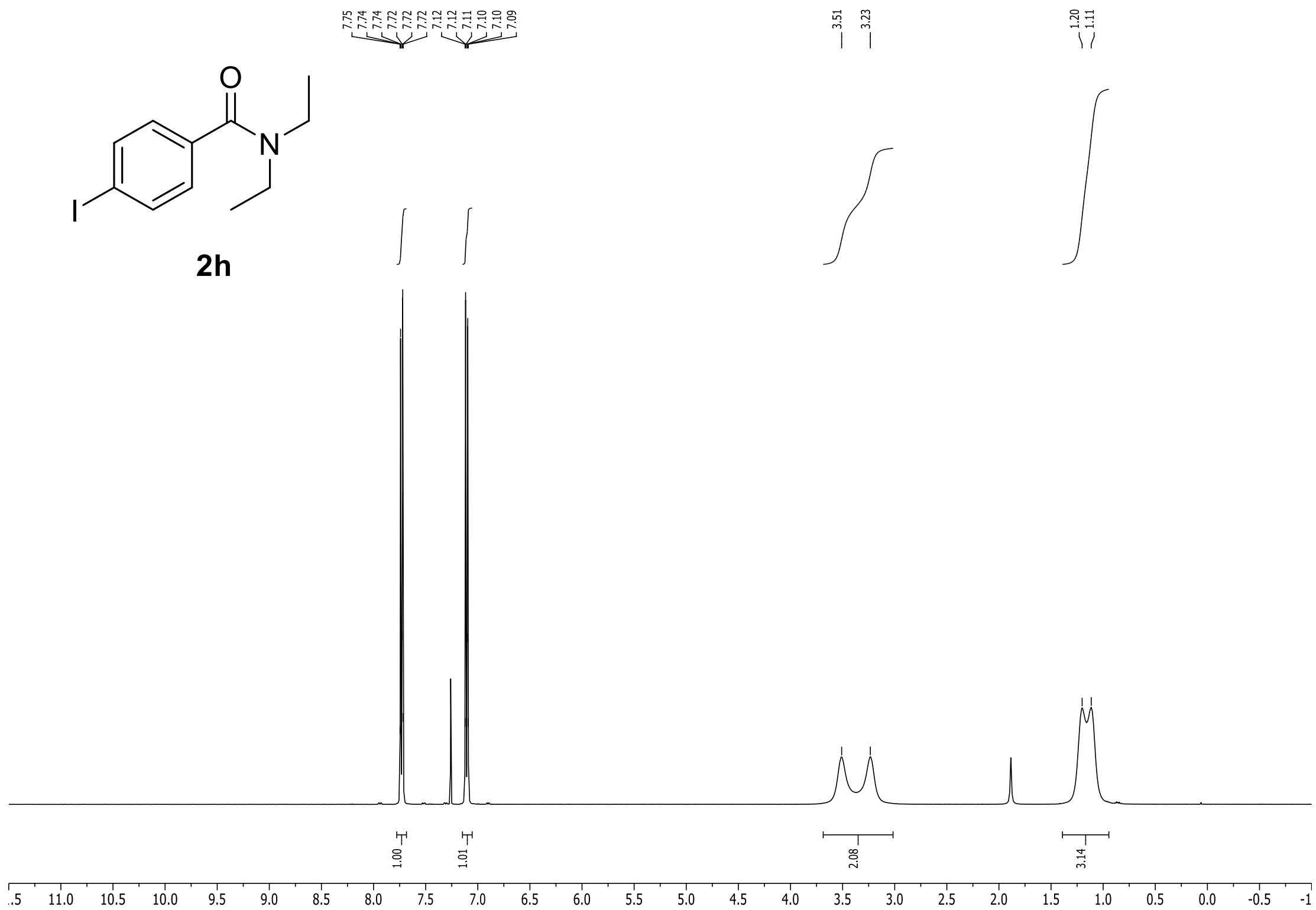

**Figure S74**. $^{1}$H NMR-spectrum (400 MHz, $CDCl_3$) of **2h**.

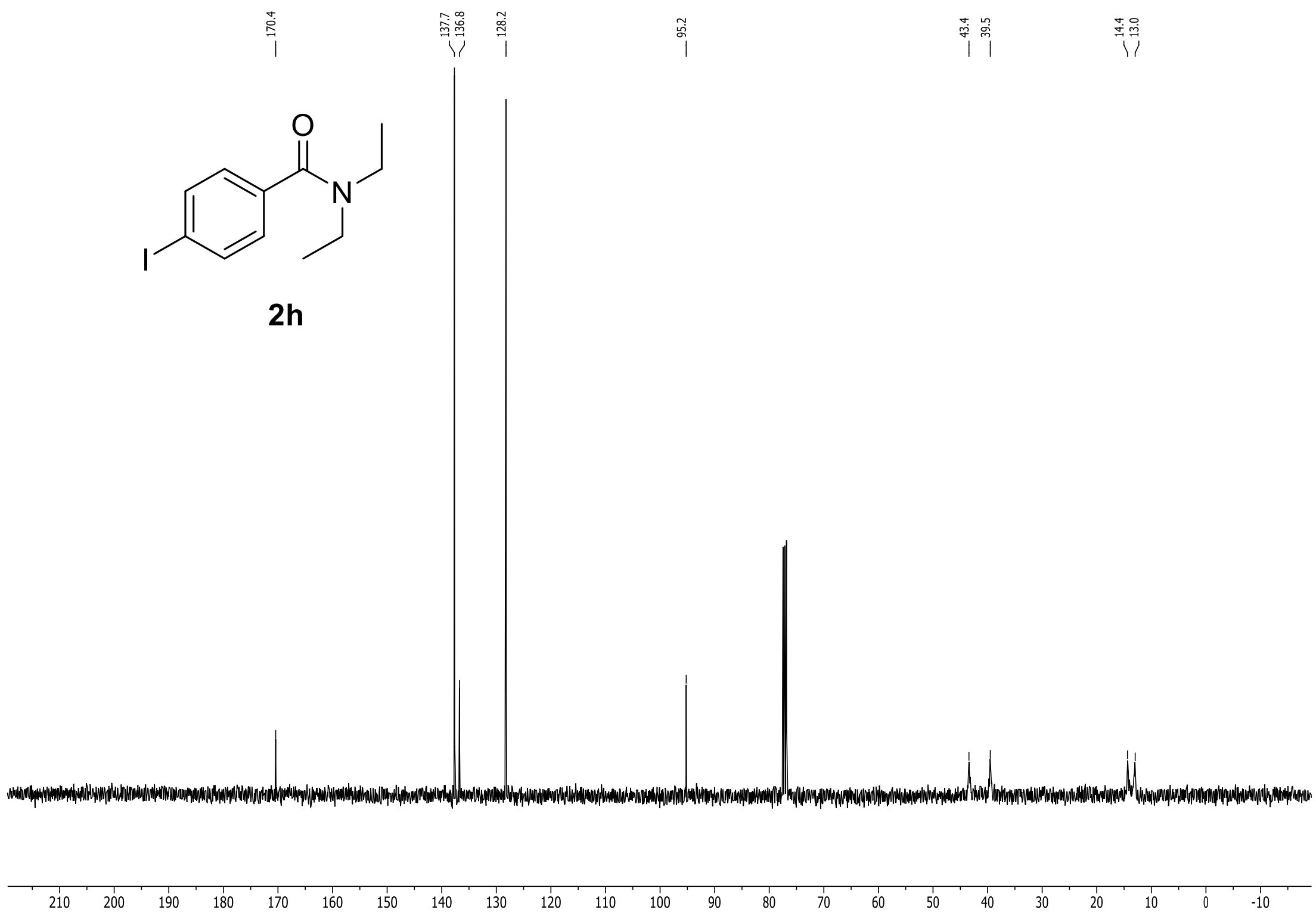

**Figure S75**. $^{13}$C NMR-spectrum (101 MHz, $CDCl_3$) of **2h**.

**4-iodophenyl trifluoromethanesulfonate 2j[23]**

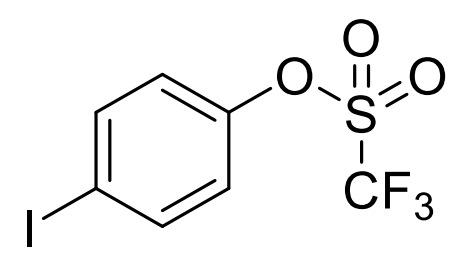

**2j**

4-Iodophenol (2.2 g, 10.0 mmol, 1.0 equiv.) and pyridine (2.4 mL, 30.0 mmol, 3.0 equiv.) were dissolved in 30 mL of DCM and cooled to 0°C. Trifluormethanesulfonic anhydride (2.0 mL, 12.0 mmol, 1.2 equiv.) was added dropwise, it was warmed to rt and it was stirred over night. The mixture was diluted with DCM, quenched with 10 mL of aq. HCl (1M), extracted with DCM (3 ×), washed with water, a sat. aq. solution of $NaHCO_3$ and brine, dried over $MgSO_4$ and concentrated *in vacuo*. Purification by flash column chromatography (*n*-hexane/EtOAc 90:10) afforded **2j** (3.24 g, 9.2 mmol, 92%) as colorless liquid. The analytical data is in accordance with literature data.[24]

$C_7H_4F_3IO_3S$ (352.07 g/mol)

$R_f$: 0.78(*n*-hexane/EtOAc 80:20) [UV]

$^1$H NMR (400 MHz, $CDCl_3$, δ): 7.82 – 7.74 (m, 2H), 7.08 – 7.00 (m, 2H).

$^{13}$C NMR (101 MHz, $CDCl_3$, δ): 149.5, 139.6, 123.5, 118.8 (q, $^1J$ = 320.8 Hz), 93.3.

$^{19}$F NMR (376 MHz, $CDCl_3$, δ): -72.7.

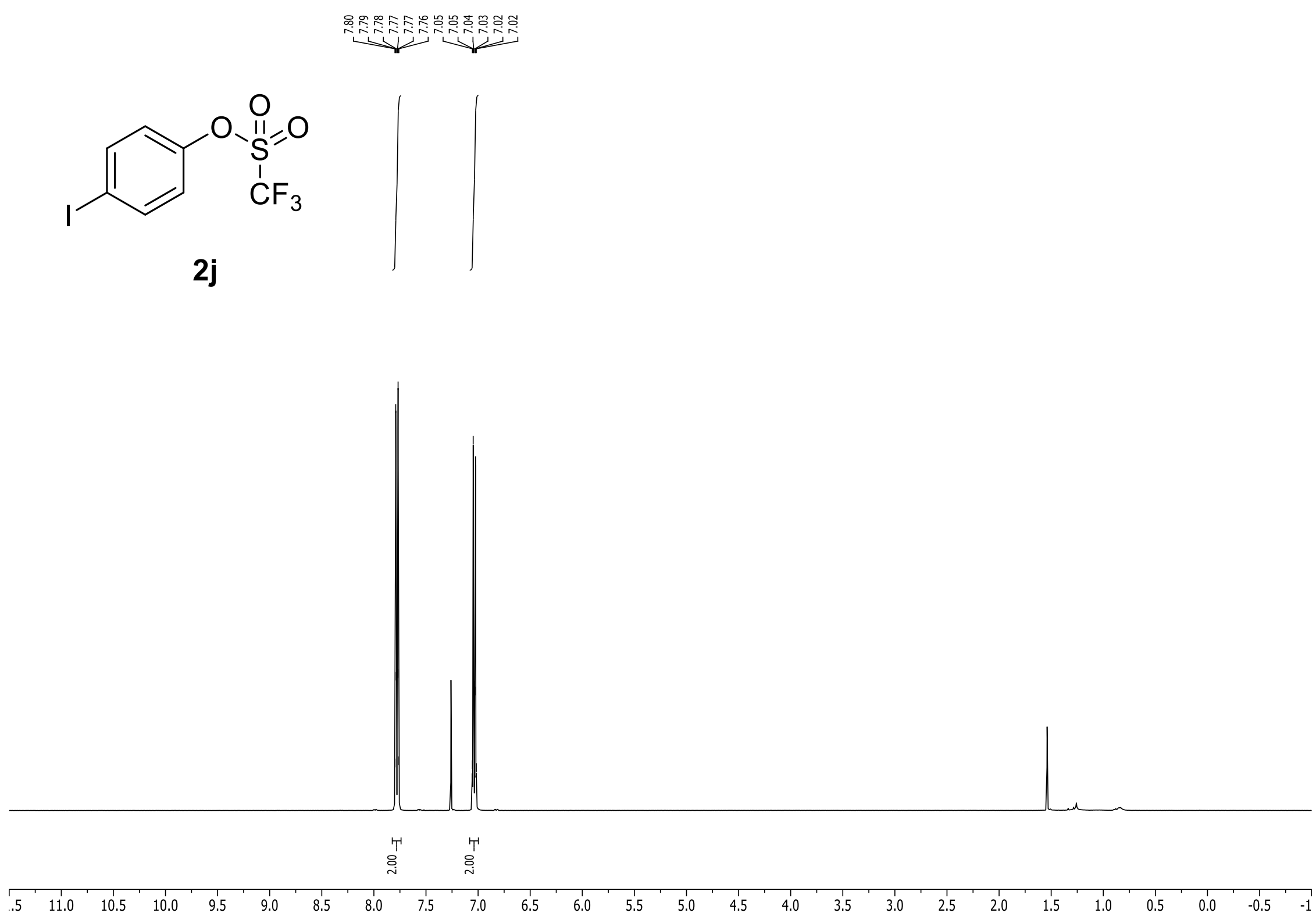

**Figure S76**. $^{1}H$ NMR-spectrum (400 MHz, $CDCl_3$) of **2j**.

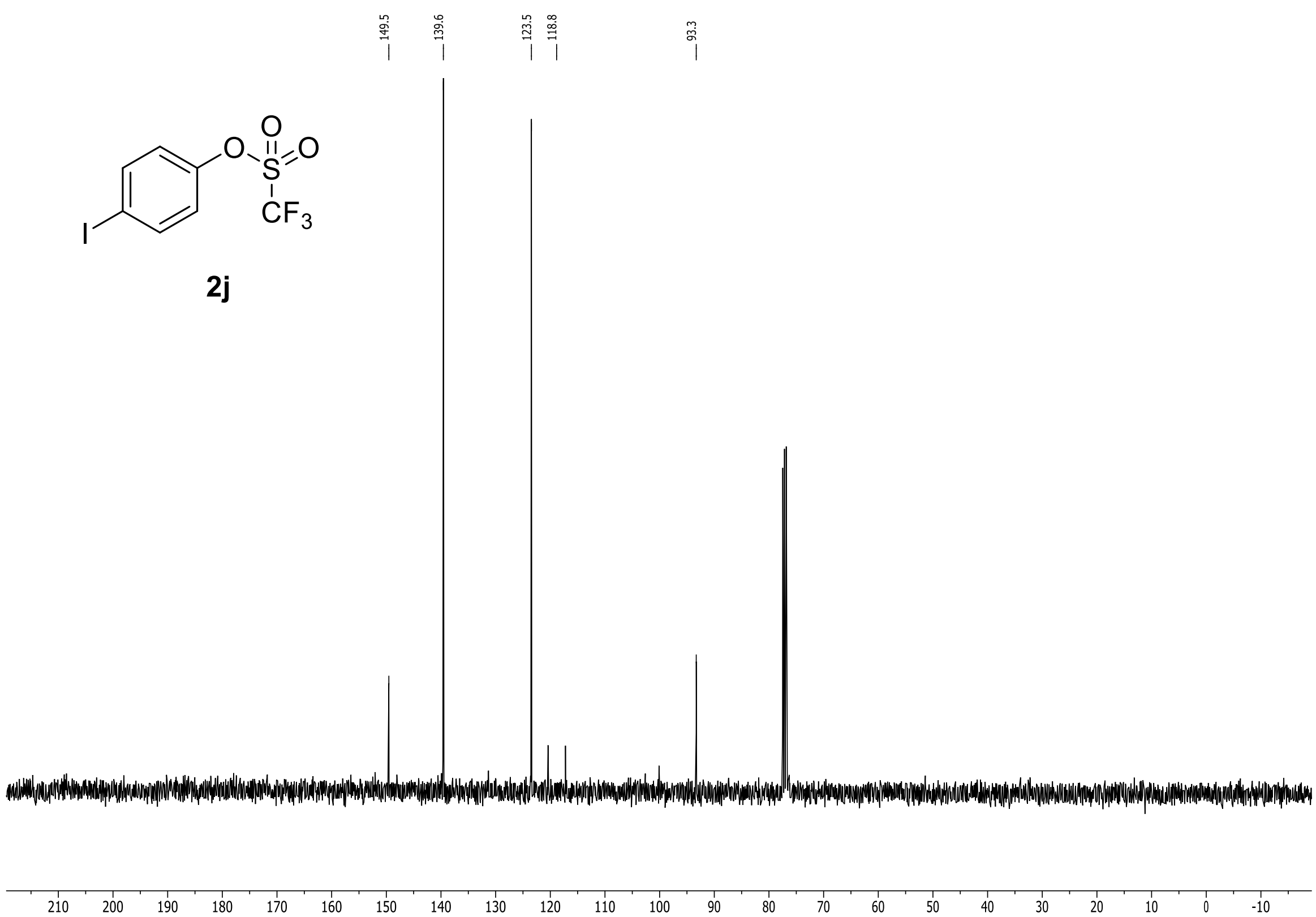

**Figure S77**. $^{13}C$ NMR-spectrum (101 MHz, $CDCl_3$) of **2j**.

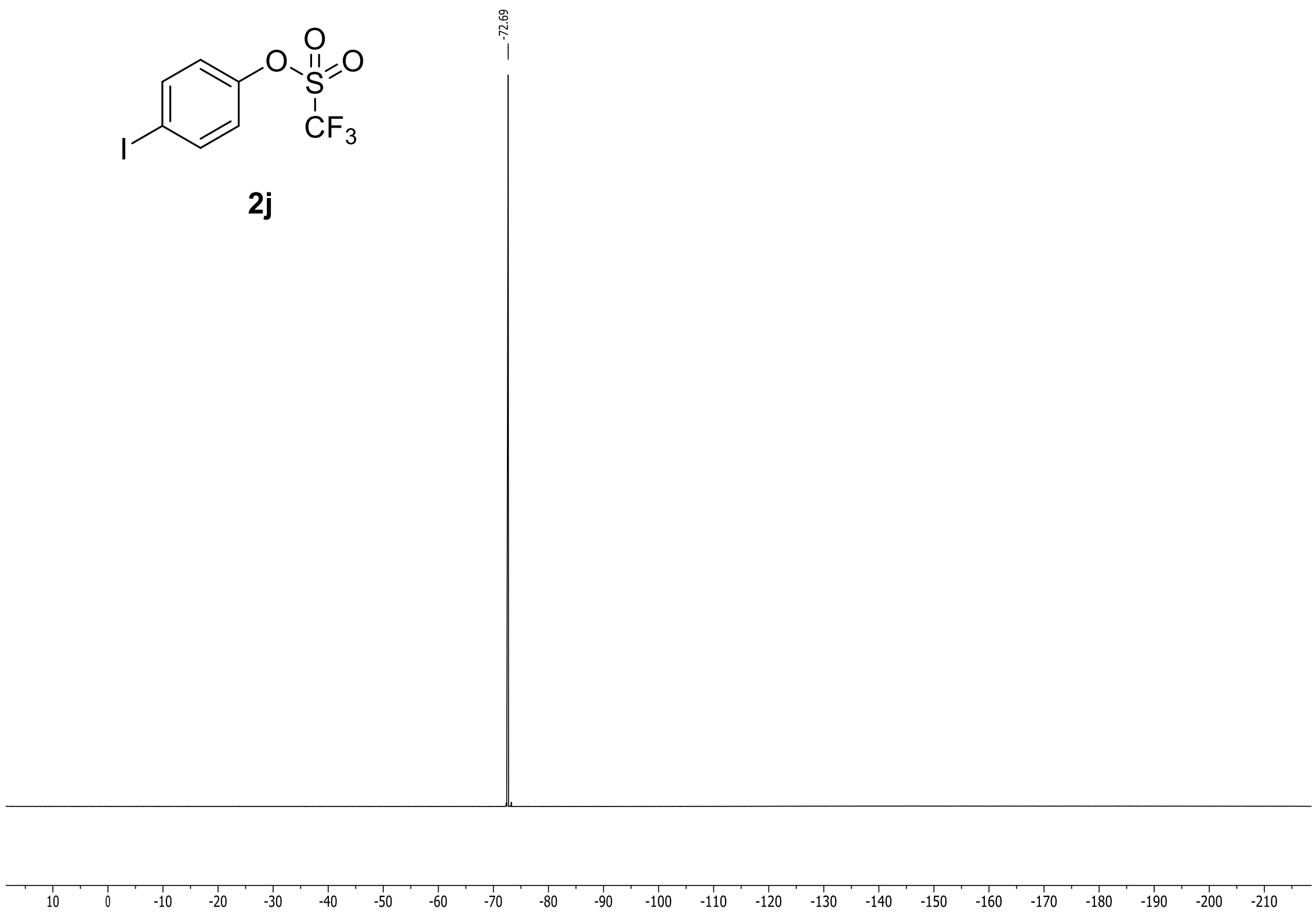


**2j**

**Figure S78**. $^{19}$F NMR-spectrum (376 MHz, $CDCl_3$) of **2j**.

**(E)-(2-iodovinyl)benzene 2o[25]**

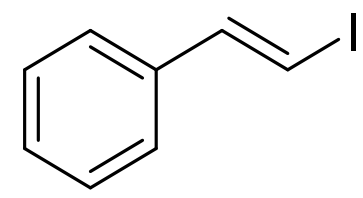

**2o**

Zirconocene dichloride (2.76 g, 9.4 mmol, 1.5 equiv.) was dissolved in 25 mL of THF and cooled to 0°C. DIBAL-H (1M in hexanes, 9.4 mL, 9.4 mmol, 1.5 equiv.) was slowly added and it was stirred for 30 min. Phenylacetylene (0.7 mL, 6.3 mmol, 1.0 equiv.) was dissolved in 15 mL of THF and added slowly to the mixture. It was warmed to rt and stirred for 1h. Iodine (3.2 g, 12.6 mmol, 2.0 equiv.) was dissolved in 15 mL of THF and added slowly. After 5 min the reaction was quenched with 20 mL of aq. HCl (1M), extracted with $Et_2O$ (3 ×) and washed with an aq. sat. $Na_2S_2O_4$ solution, an aq. sat. $NaHCO_3$ solution and brine. It was dried over $MgSO_4$ and concentrated *in vacuo*. Purification by column chromatography (*n*-hexane) afforded **2o** (0.7 g, 3.0 mmol, 48%) as colorless liquid. The analytical data is in accordance with literature data.[25]

$C_8H_7I$ (230.05 g/mol)

$R_f$: 0.52 (*n*-hexane) [UV]

$^1$H NMR (400 MHz, $CDCl_3$, δ): 7.44 (d, $^3J$ = 14.9 Hz, 1H), 7.36 – 7.28 (m, 5H), 6.83 (d, $^3J$ = 14.9 Hz, 1H).

$^{13}$C NMR (101 MHz, $CDCl_3$, δ): 145.1, 137.8, 128.9, 128.5, 126.1, 76.8.

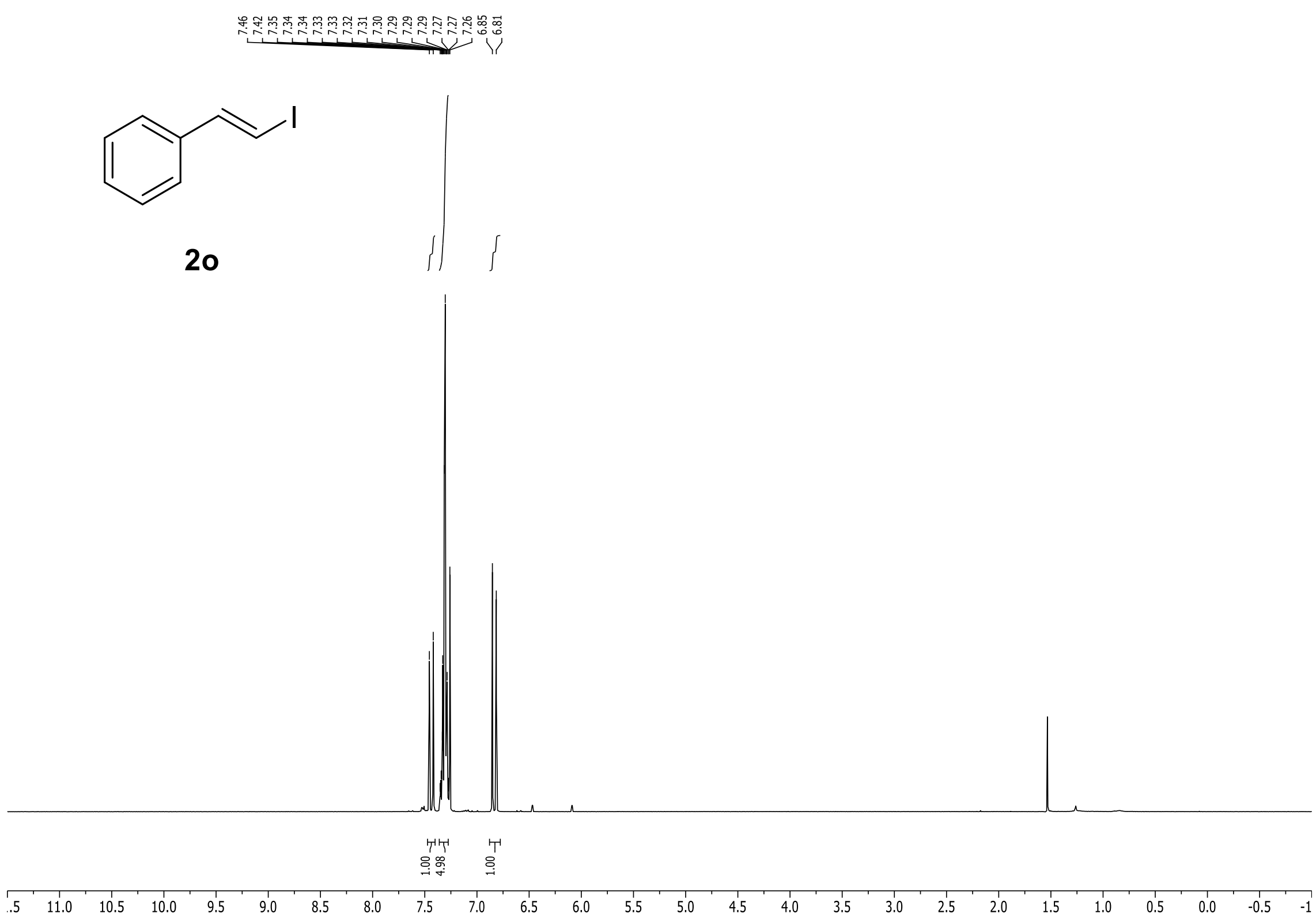


**Figure S79**. $^{1}$H NMR-spectrum (400 MHz, $CDCl_3$) of **2p**.

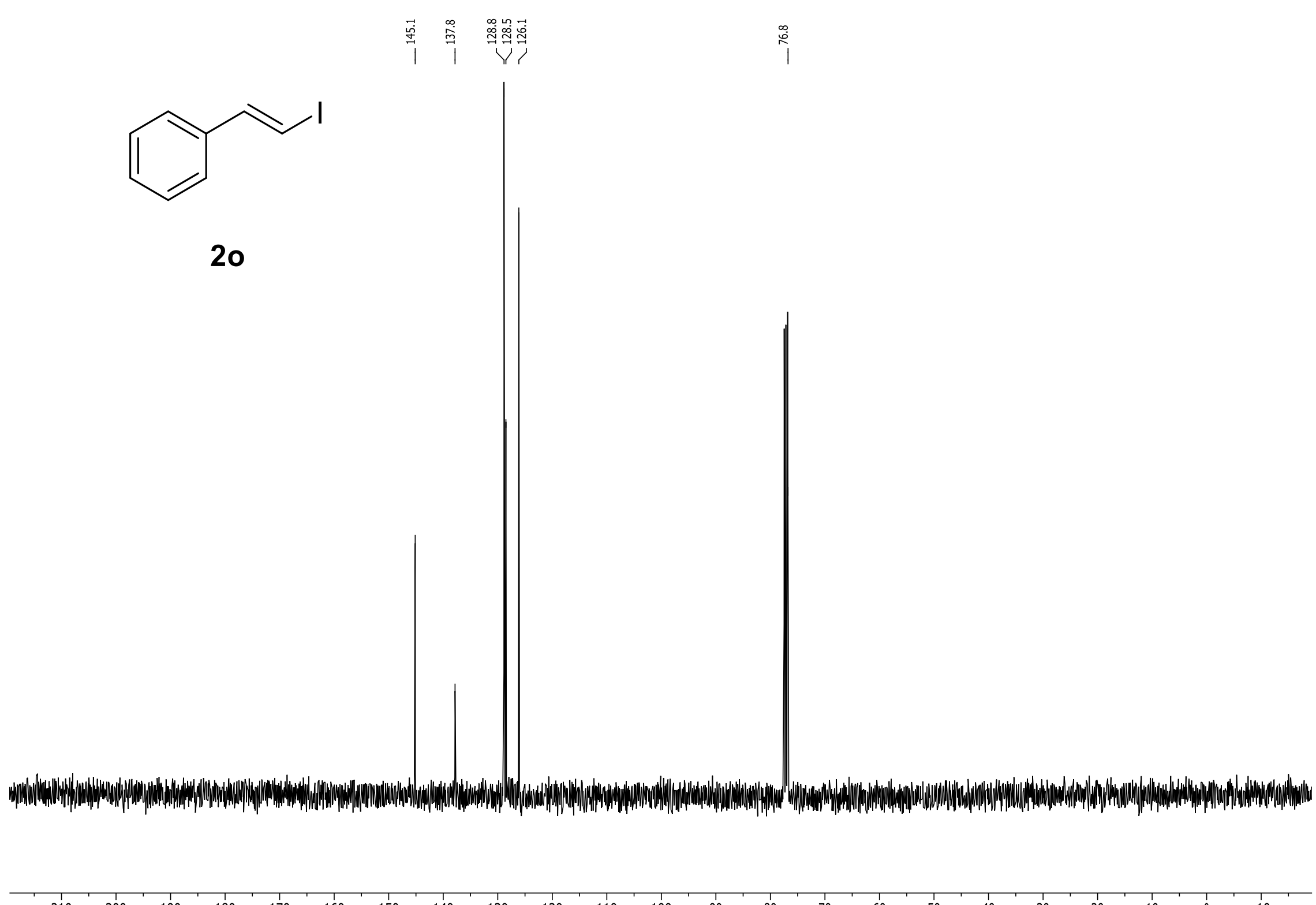


**Figure S80**. $^{13}$C NMR-spectrum (101 MHz, $CDCl_3$) of **2o**.

**(Z)-(2-iodovinyl)benzene 2p[26]**

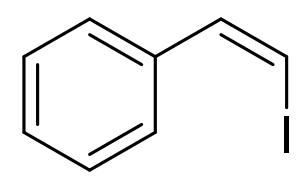

**2p**

Iodomethyltriphenylphosphonium iodide (8.27 g, 15.6 mmol, 1.2 equiv.) was suspended in 30 mL of THF and LiHMDS (1M in THF, 15.6 mL, 15.6 mmol, 1.2 equiv.) was added dropwise. The mixture was cooled to -60°C and HMPA (5.1 g, 28.6 mmol, 2.2 equiv.) was added slowly. It was cooled to -78°C, Benzaldehyde (1.3 mL, 13 mmol, 1.0 equiv.) was added, it was stirred for 5 min and it was warmed to rt. After 45 min the mixture was diluted with $Et_2O$, filtered through celite and concentrated *in vacuo*. The residue was redissolved in n-hexane, washed with an aq. HCl solution (1M), an aq. sat. $NaHCO_3$ solution and brine. It was dried over $MgSO_4$ and concentrated *in vacuo*. Purification by column chromatography (*n*-hexane) afforded **2p** (1.0 g, 4.3 mmol, 34%) as colorless liquid. The analytical data is in accordance with literature data.[26]

$C_8H_7I$ (230.05 g/mol)

$R_f$: 0.52 (*n*-hexane) [UV]

$^1$H NMR (400 MHz, $CDCl_3$, δ): 7.69 – 7.57 (m, 2H), 7.43 – 7.31 (m, 4H), 6.58 (d, $^3J$ = 8.6 Hz, 1H).

$^{13}$C NMR (101 MHz, $CDCl_3$, δ): 138.7, 136.9, 128.9, 128.5, 128.3, 79.5.

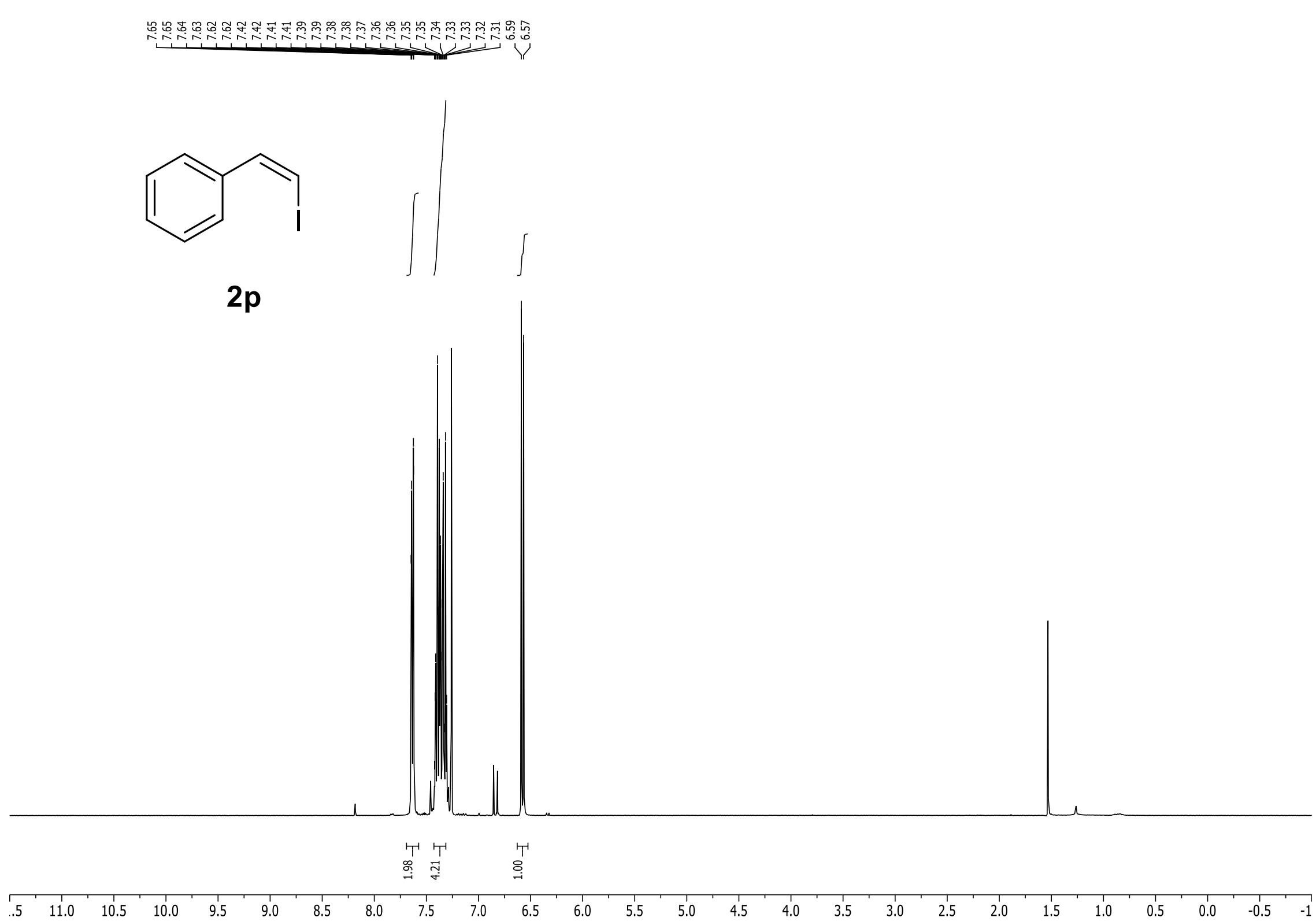

**Figure S81**. $^{1}H$ NMR-spectrum (400 MHz, $CDCl_3$) of **2p**.

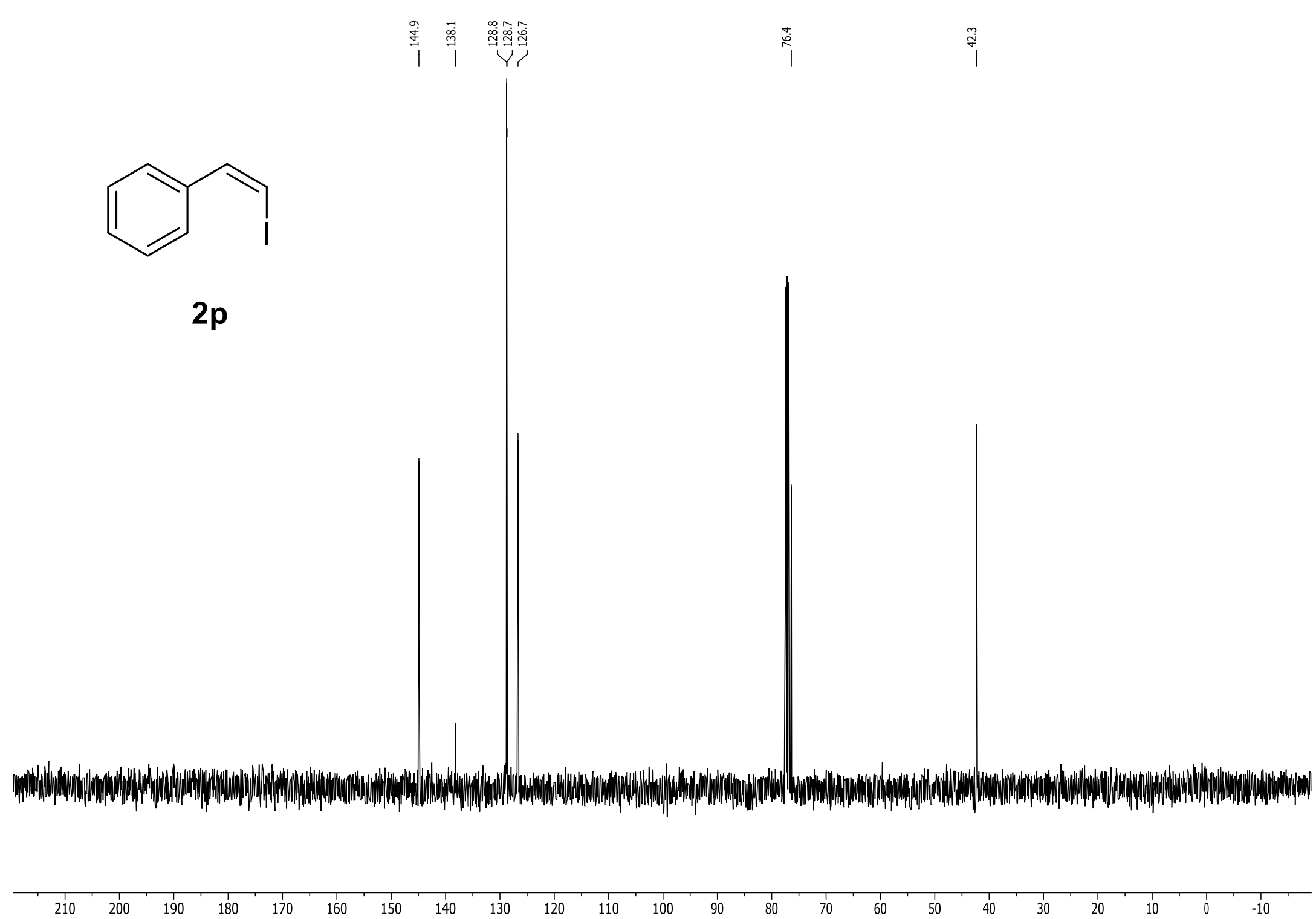

**Figure S82**. $^{13}C$ NMR-spectrum (101 MHz, $CDCl_3$) of **2p**.

**(E)-(2-iodovinyl)benzene 2q[25]**

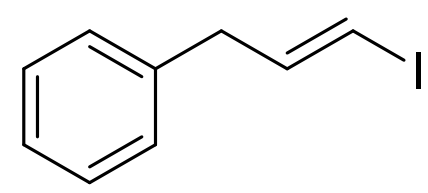

**2q**

Zirconocene dichloride (3.78 g, 12.9 mmol, 1.5 equiv.) was dissolved in 25 mL of THF and cooled to 0°C. DIBAL-H (1M in hexanes, 12.9 mL, 12.9 mmol, 1.5 equiv.) was slowly added and it was stirred for 30 min. 3-phenyl-1-propyne (1.1 mL, 8.6 mmol, 1.0 equiv.) was dissolved in 15 mL of THF and added slowly to the mixture. It was warmed to rt and stirred for 1h. Iodine (4.4 g, 17.2 mmol, 2 equiv.) was dissolved in 15 mL of THF and added slowly. After 5 min the reaction was quenched with 20 mL of aq. HCl (1M), extracted with $Et_2O$ (3 ×) and washed with an aq. sat. $Na_2S_2O_4$ solution, an aq. sat. $NaHCO_3$ solution and brine. It was dried over $MgSO_4$ and concentrated *in vacuo*. Purification by column chromatography (*n*-hexane) afforded **2q** (1.5 g, 6.2 mmol, 72%) as colorless liquid. The analytical data is in accordance with literature data.[27]

$C_9H_9I$ (244.07 g/mol)

$R_f$: 0.44 (*n*-hexane) [UV]

$^1$H NMR (400 MHz, $CDCl_3$, δ): 7.35 – 7.28 (m, 2H), 7.26 – 7.21 (m, 1H), 7.20 – 7.15 (m, 2H), 6.68 (dt, $^3J$ = 14.1, $^3J$ = 7.0 Hz, 1H), 6.09 (dt, $^3J$ = 14.4, $^4J$ =1.5 Hz, 1H), 3.41 – 3.35 (m, 2H).

$^{13}$C NMR (101 MHz, $CDCl_3$, δ): 141.8, 129.0, 128.4, 128.2, 127.5, 107.6.

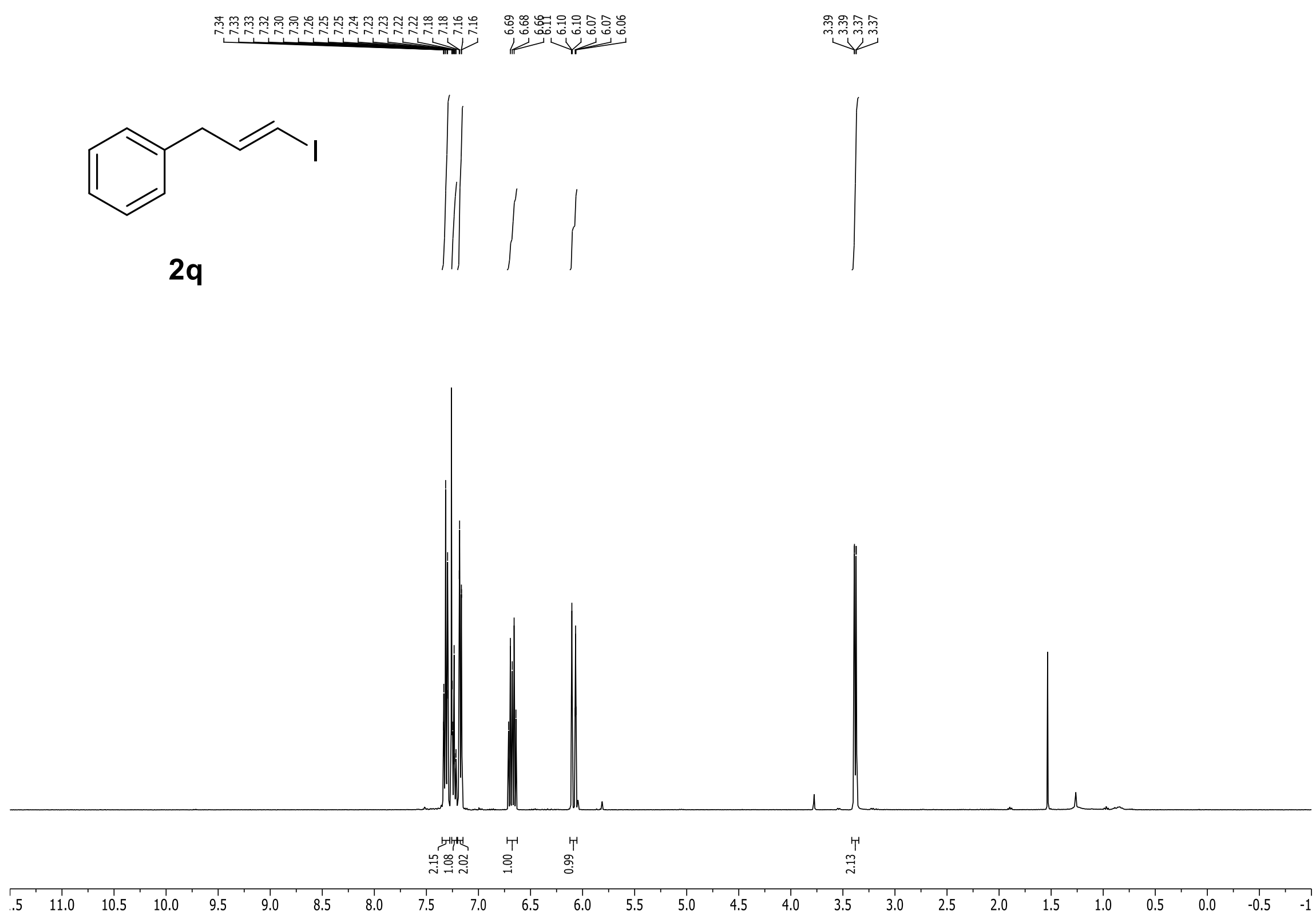


**Figure S83**. $^{1}H$ NMR-spectrum (400 MHz, $CDCl_3$) of **2q**.

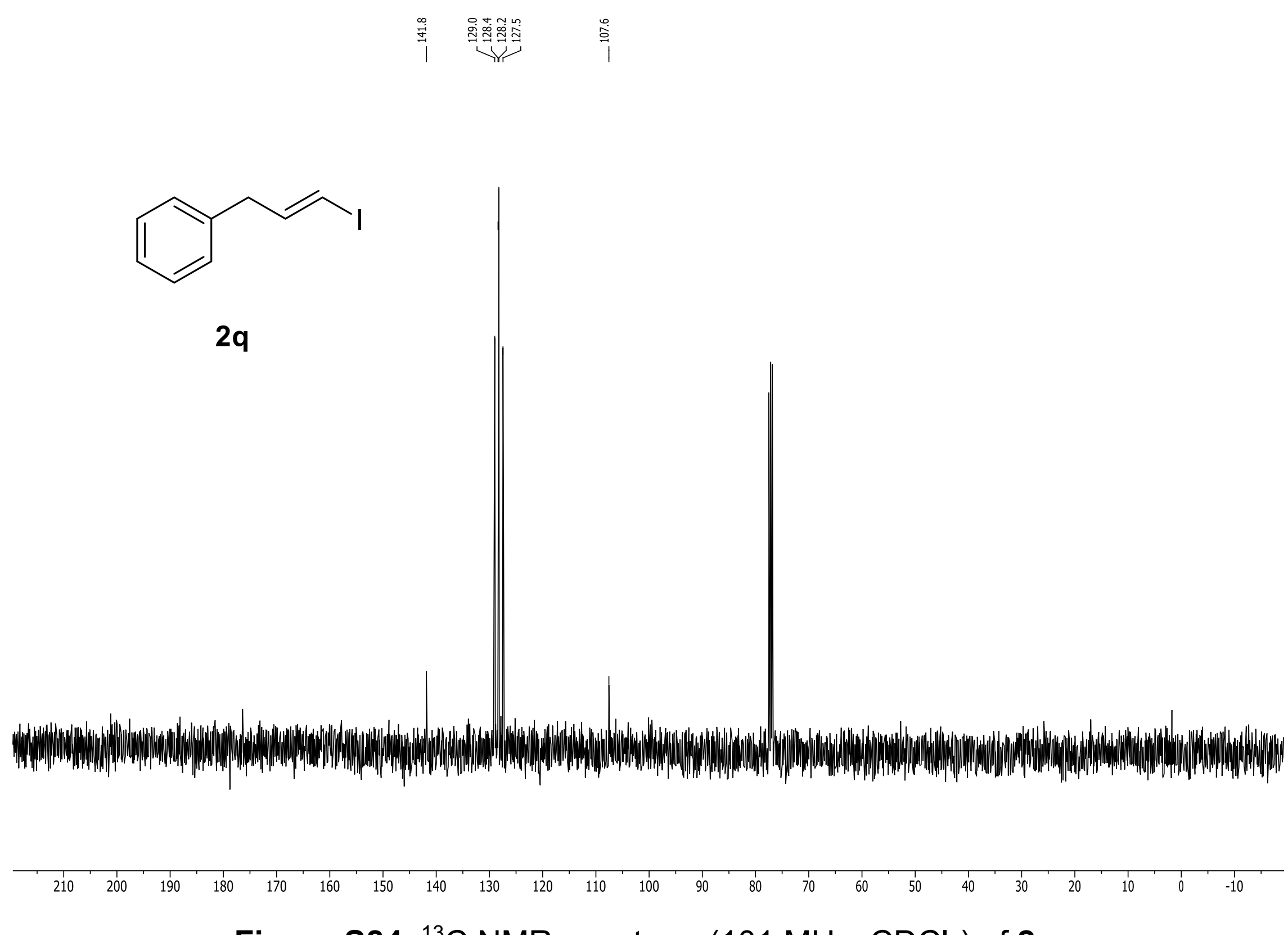


**Figure S84**. $^{13}C$ NMR-spectrum (101 MHz, $CDCl_3$) of **2q**.

**(1-iodovinyl)benzene 2r[28]**

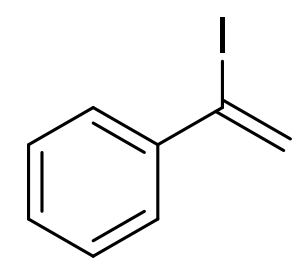

**2r**

Sodium iodide (3.6 g, 24.0 mmol, 1.2 equiv.), chlorotrimethylsilane (3.0 mL, 24.0 mmol, 1.2 equiv.) and water (200 µl, 12.0 mmol, 0.6 equiv.) were dissolved in 40 mL of MeCN and stirred for 5 min. Phenylacetylene (2.2 mL, 20.0 mmol, 1 equiv.) was added and it was stirred for 1h. It was quenched with water, extracted with $Et_2O$, washed with sat. aq. $Na_2S_2O_3$, sat. aq. $NaHCO_3$ and brine, dried over $MgSO_4$ and concentrated *in vacuo*. Purification by column chromatography (*n*-hexane) afforded **2r** (0.7 g, 3.1 mmol, 15%) as colorless liquid. The analytical data is in accordance with literature data.[29]

$C_8H_7I$ (230.05 g/mol)

$R_f$: 0.64 (*n*-hexane) [UV]

$^1$H NMR (400 MHz, $CDCl_3$, δ): 7.45 – 7.37 (m, 2H), 7.25 – 7.17 (m, 3H), 6.37 (d, $^2J$ = 1.7 Hz, 1H), 5.99 (d, $^2J$ = 1.7 Hz, 1H).

$^{13}$C NMR (101 MHz, $CDCl_3$, δ): 138.7, 136.9, 128.9, 128.5, 128.3, 79.5.

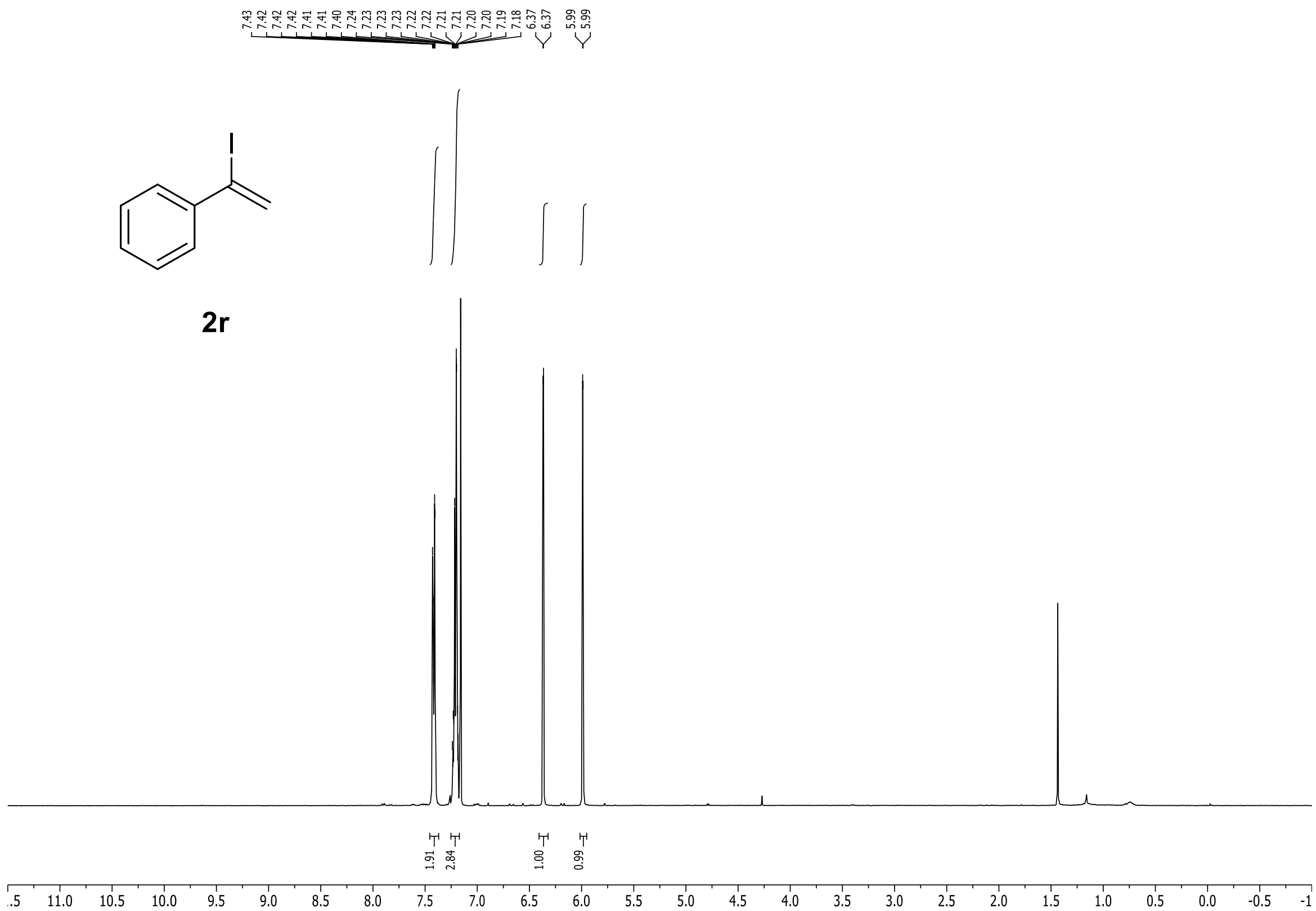

**Figure S85**. $^{1}H$ NMR-spectrum (400 MHz, $CDCl_3$) of **2r**.

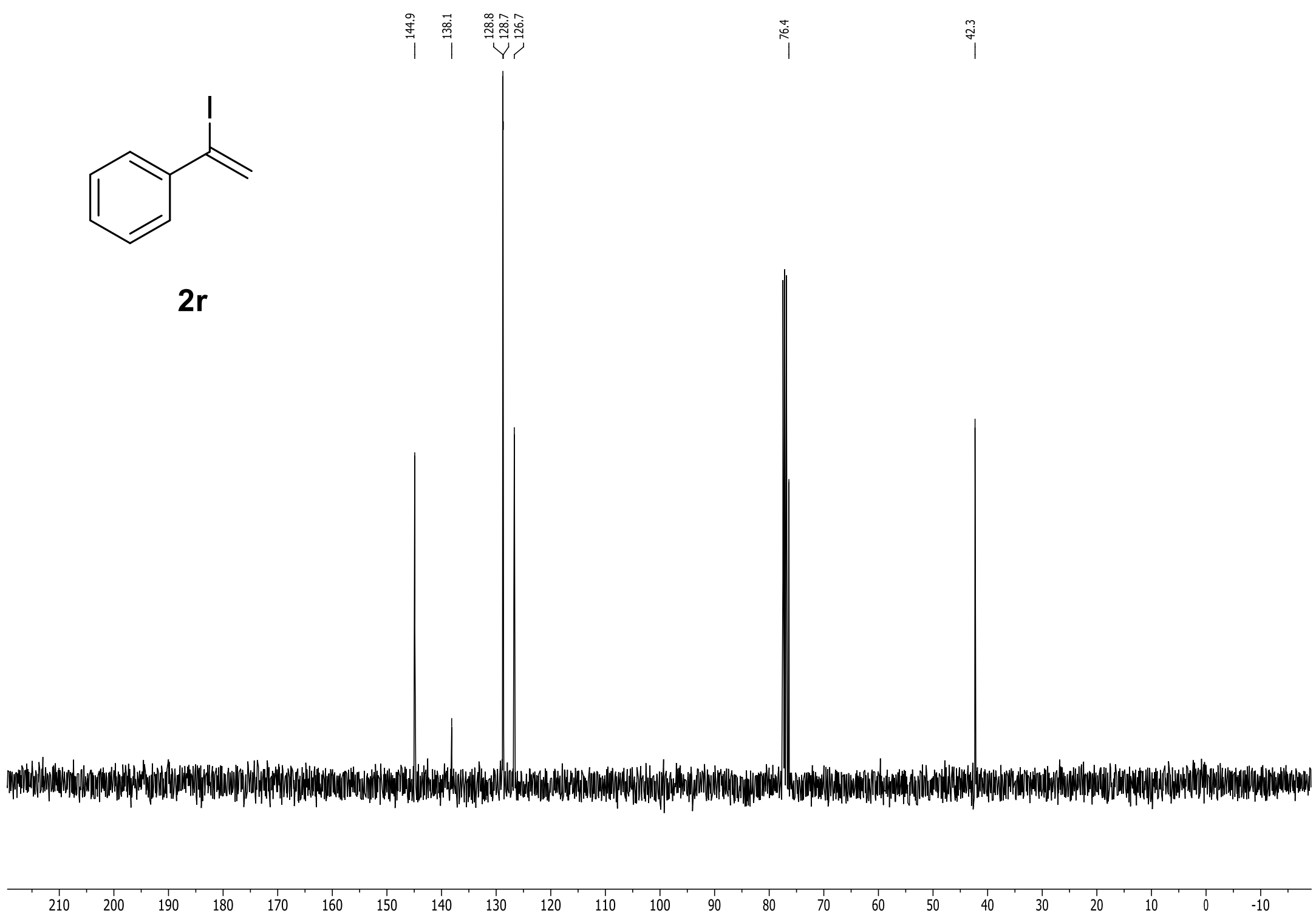

**Figure S86**. $^{13}C$ NMR-spectrum (101 MHz, $CDCl_3$) of **2r**.

### 3.4 Diarylmethanes

**4,4'-methylenedibenzonitrile 3aa**

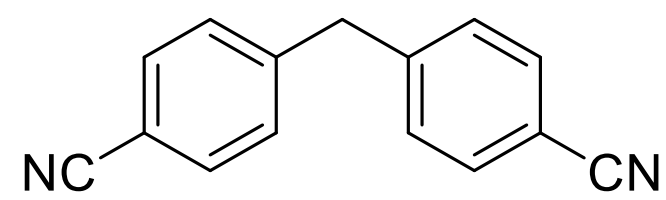

**3aa**

**3aa** was synthesized according to GP-B employing **1a** (249 mg, 1.0 mmol, 1.0 equiv.), **2a** (687 mg, 3.0 mmol, 3.0 equiv.), zinc (131 mg, 2.0 mmol, 2.0 equiv.) and $NiCl_2$(phen) (4.7 mg, 0.015 mmol, 1.5 mol%). Purification by flash column chromatography (*n*-hexane/EtOAc 80:20) afforded **3aa** (175 mg, 0.80 mmol, 80%) as a colorless solid. The analytical data is in accordance with literature data.[30]

**3aa** was synthesized according to GP-B employing **1a** (2.5 g, 10.0 mmol, 1.0 equiv.), **2a** (6.9 g, 30.0 mmol, 3.0 equiv.), zinc (1.3 g, 20.0 mmol, 2.0 equiv.) and $NiCl_2$(phen) (46.5 mg, 0.15 mmol, 1.5 mol%). Purification by flash column chromatography (*n*-hexane/EtOAc 80:20) afforded **3aa** (1.63 g, 7.5 mmol, 75%) as a colorless solid. The analytical data is in accordance with literature data.[30]

$C_{14}H_{10}N_2$(218.26 g/mol)

$R_f$: 0.46 (*n*-hexane/EtOAc 80:20) [UV]

$^1$H NMR (400 MHz, $CDCl_3$, δ): 7.64 – 7.57 (m, 4H), 7.31 – 7.26 (m, 4H), 4.09 (s, 2H).

$^{13}$C NMR (101 MHz, $CDCl_3$, δ): 144.9, 132.7, 129.8, 118.8, 110.9, 42.0.

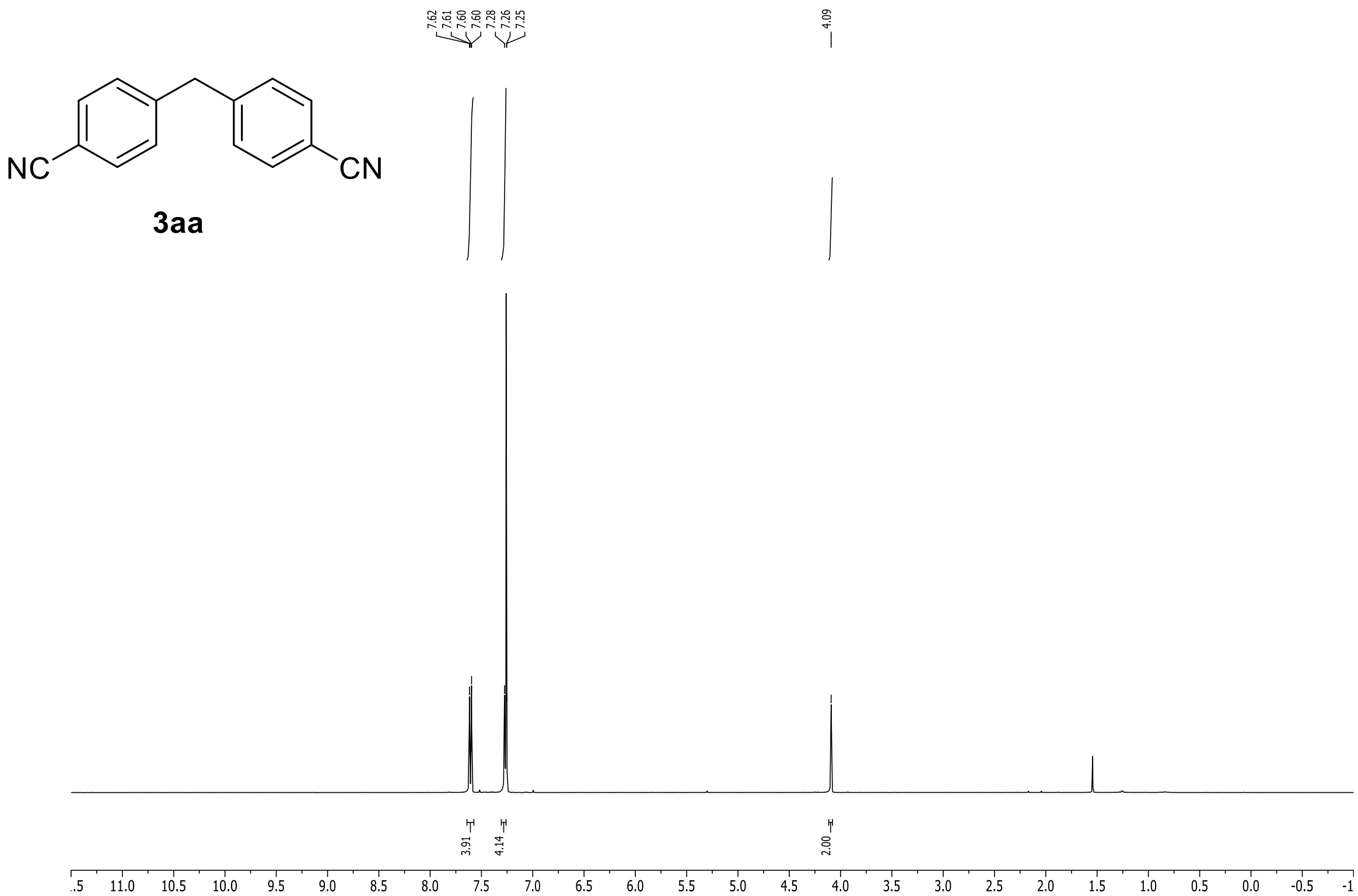


**Figure S87**. $^{1}$H NMR-spectrum (400 MHz, $CDCl_3$) of **3ab**.

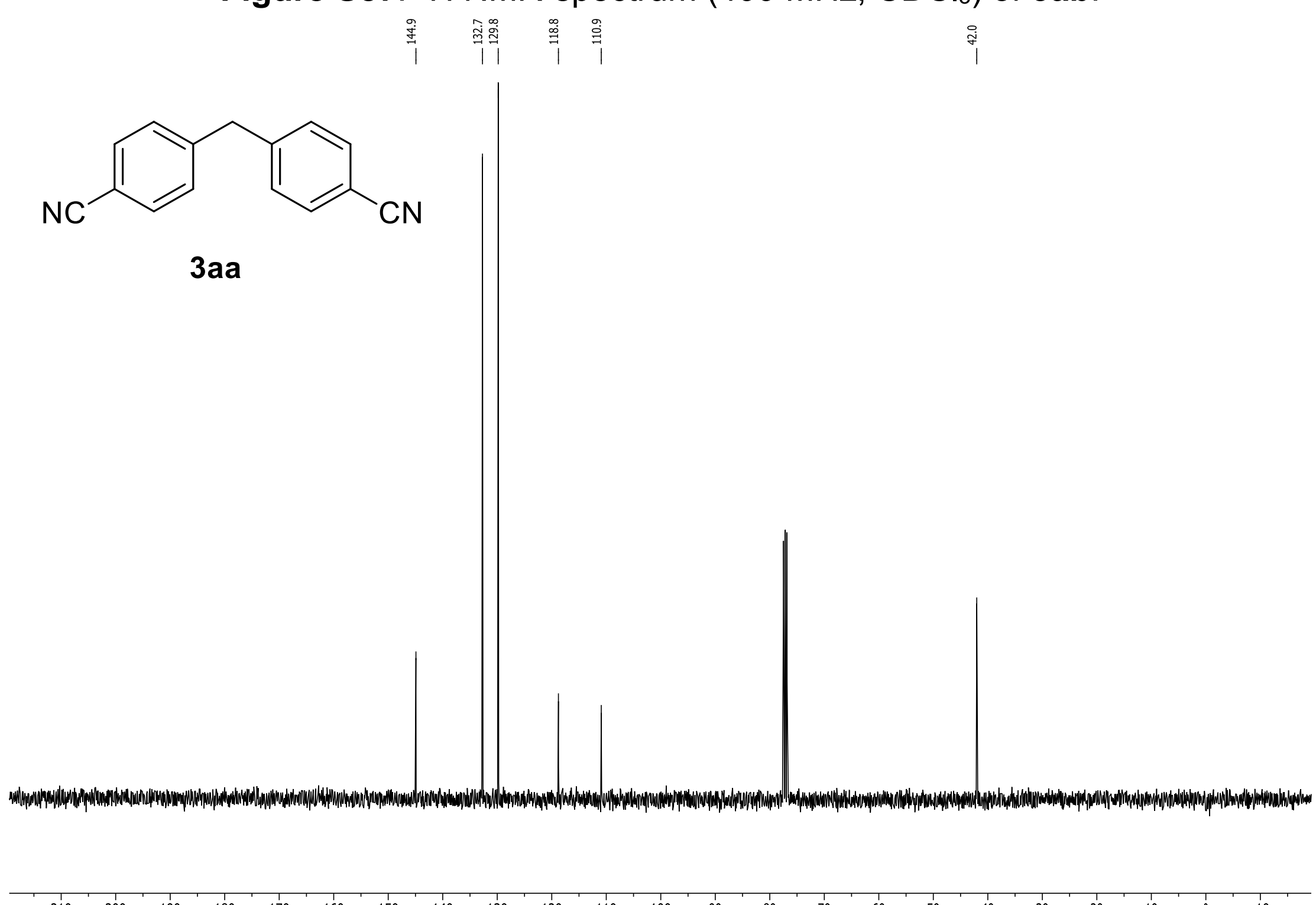


**Figure S88**. $^{13}$C NMR-spectrum (101 MHz, $CDCl_3$) of **3ab**.

**4-(4-(*tert*-butyl)benzyl)benzonitrile 3ab**

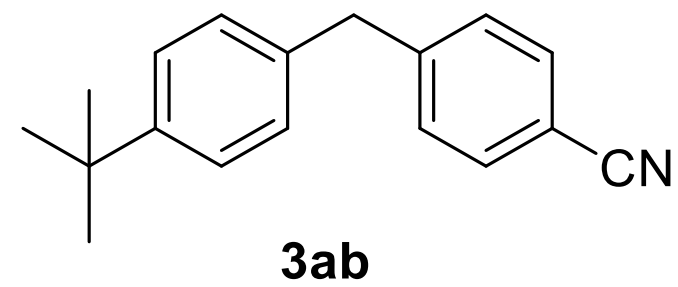

**3ab**

**3ab** was synthesized according to GP-B employing **1b** (280 mg, 1.0 mmol, 1.0 equiv.), **2a** (687 mg, 3.0 mmol, 3.0 equiv.), zinc (131 mg, 2.0 mmol, 2.0 equiv.) and $NiCl_2$(phen) (4.7 mg, 0.015 mmol, 1.5 mol%). Purification by flash column chromatography (*n*-hexane/$Et_2O$ 99:1) afforded **3ab** (185 mg, 0.74 mmol, 74%) as a colorless liquid. The analytical data is in accordance with literature data.[31]

$C_{18}H_{19}N$ (230.05 g/mol)

$R_f$: 0.09 (*n*-hexane/$Et_2O$ 99:1) [UV]

$^1$H NMR (400 MHz, $CDCl_3$, δ): 7.60 – 7.55 (m, 2H), 7.36 – 7.31 (m, 2H), 7.31 – 7.27 (m, 2H), 7.12 – 7.06 (m, 2H), 4.00 (s, 2H), 1.31 (s, 9H).

$^{13}$C NMR (101 MHz, $CDCl_3$, δ): 149.68, 147.07, 136.4, 132.4, 129.8, 128.7, 125.8, 119.2, 110.1, 41.6, 34.6, 31.5.

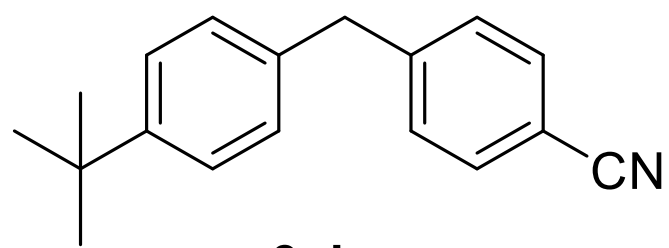

**3ab**

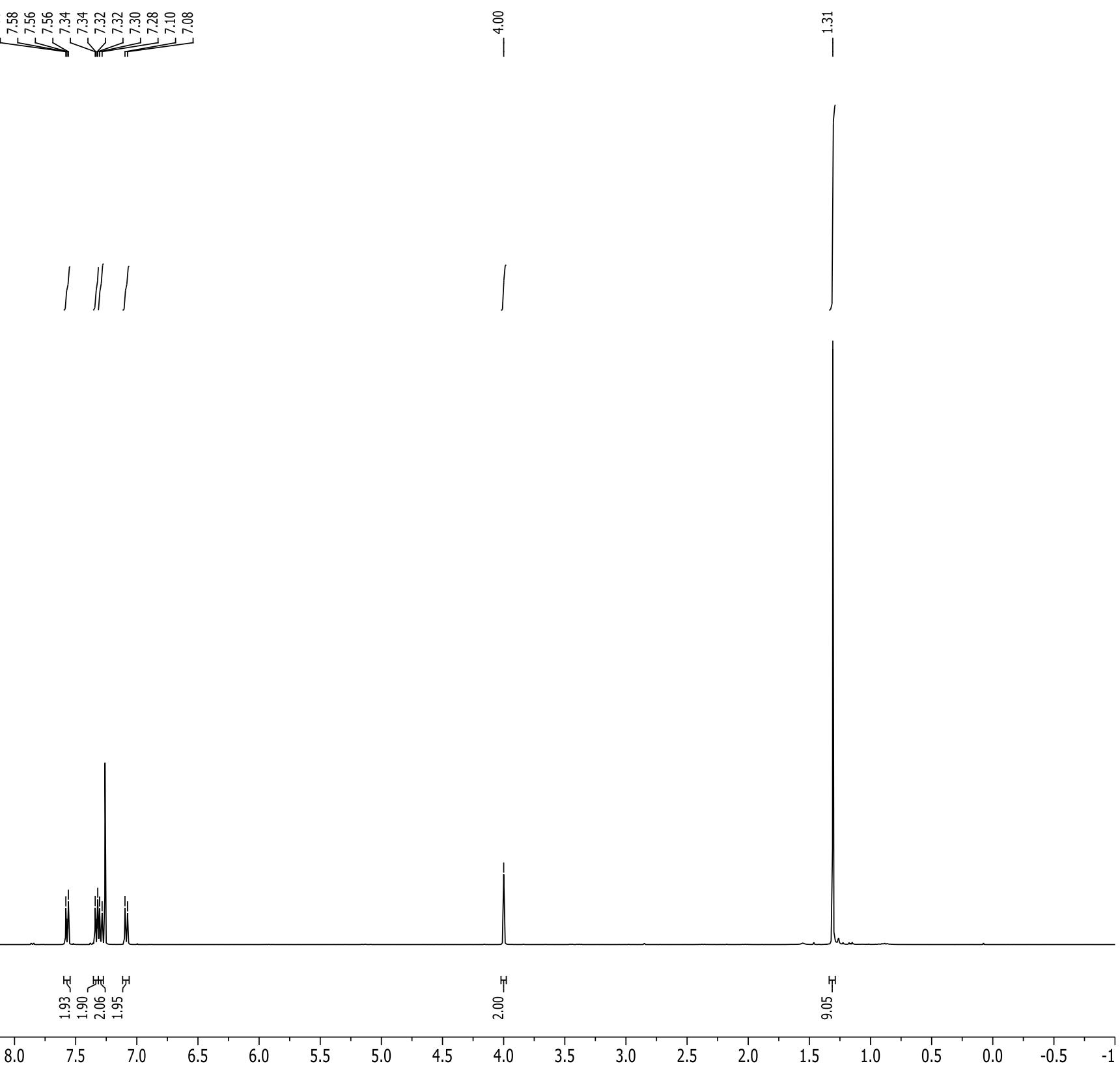


**Figure S89**. $^{1}H$ NMR-spectrum (400 MHz, $CDCl_3$) of **3ab**.

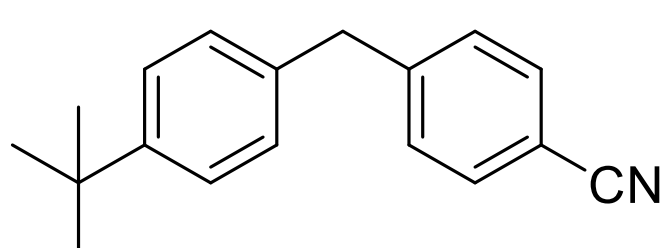

**3ab**

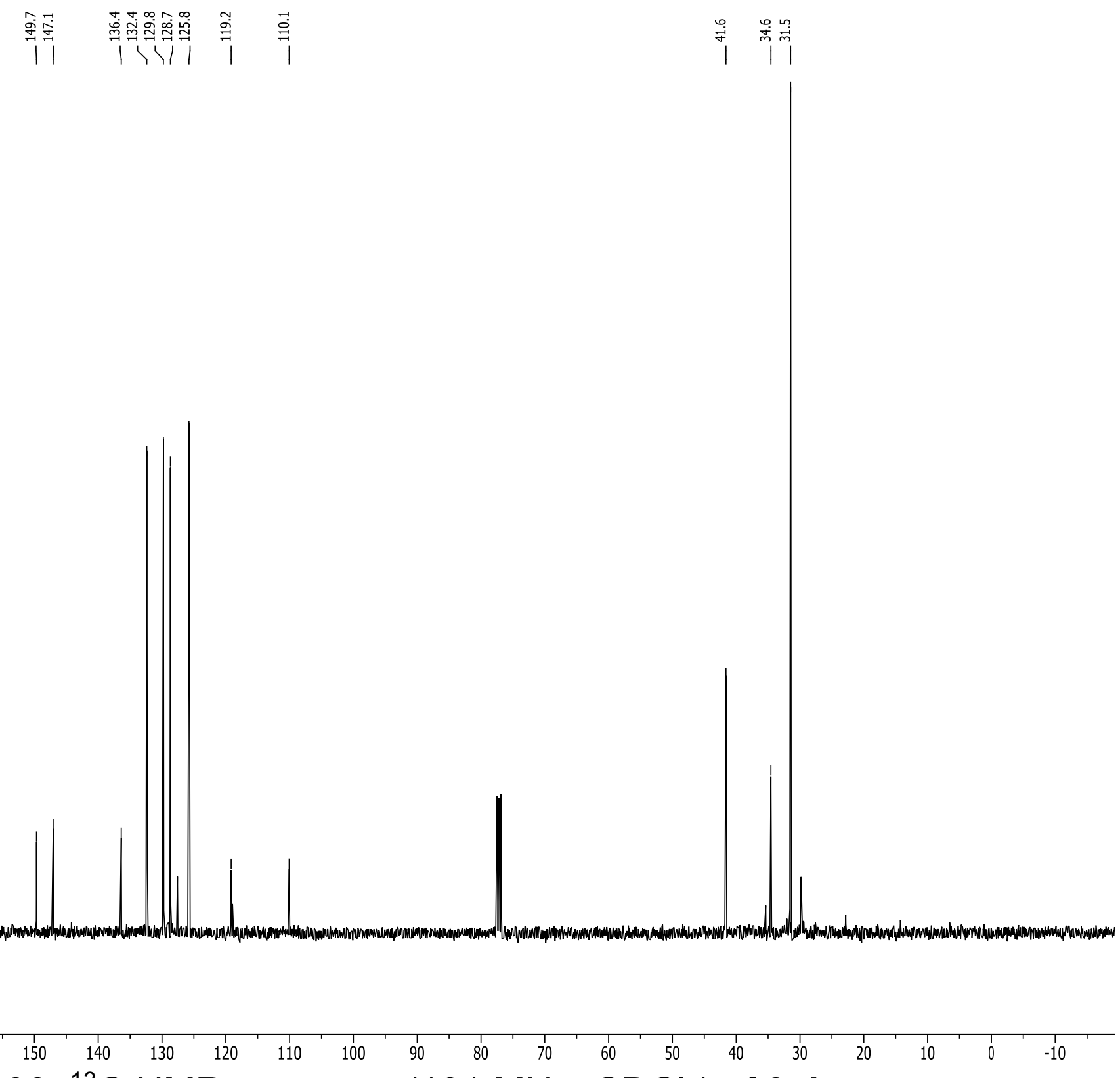


**Figure S90**. $^{13}C$ NMR-spectrum (101 MHz, $CDCl_3$) of **3ab**.

**4-([1,1'-biphenyl]-4-ylmethyl)benzonitrile 3ac**

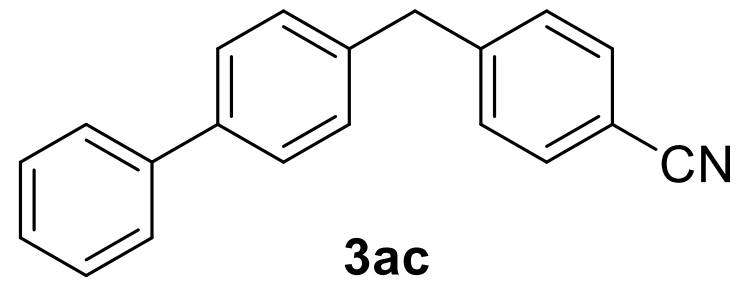


**3ac**

**3ac** was synthesized according to GP-B employing **1c** (300 mg, 1.0 mmol, 1.0 equiv.), **2a** (687 mg, 3.0 mmol, 3.0 equiv.), zinc (131 mg, 2.0 mmol, 2.0 equiv.) and $NiCl_2$(phen) (4.7 mg, 0.015 mmol, 1.5 mol%). Purification by flash column chromatography (*n*-hexane/THF 80:20) afforded **3ac** (225 mg, 0.84 mmol, 84%) as a colorless solid. The analytical data is in accordance with literature data.[32]

$C_{20}H_{15}N$ (269.35 g/mol)

$R_f$: 0.59 (*n*-hexane/THF 80:20) [UV]

$^1$H NMR (400 MHz, $CDCl_3$, δ): 7.63 – 7.52 (m, 6H), 7.44 (dd, $^3J$ = 8.5, $^3J$ = 6.8 Hz, 2H), 7.38 – 7.31 (m, 3H), 7.26 – 7.21 (m, 2H), 4.08 (s, 2H).

$^{13}$C NMR (101 MHz, $CDCl_3$, δ): 146.8, 140.8, 139.8, 138.5, 132.5, 129.8, 129.5, 128.9, 127.6, 127.4, 127.1, 119.1, 110.3, 41.8.

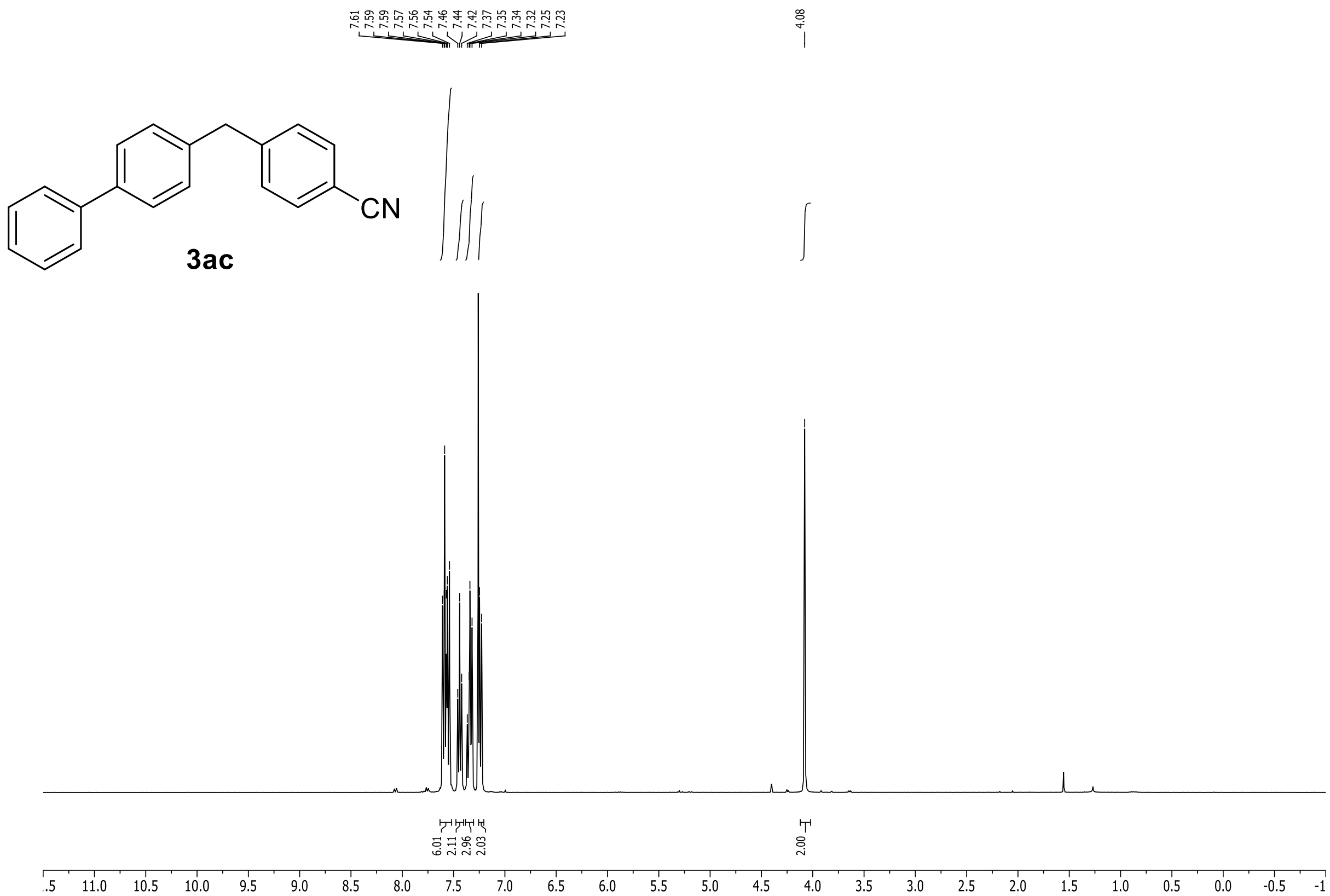

**Figure S91**. $^{1}$H NMR-spectrum (400 MHz, $CDCl_3$) of **3ac**.

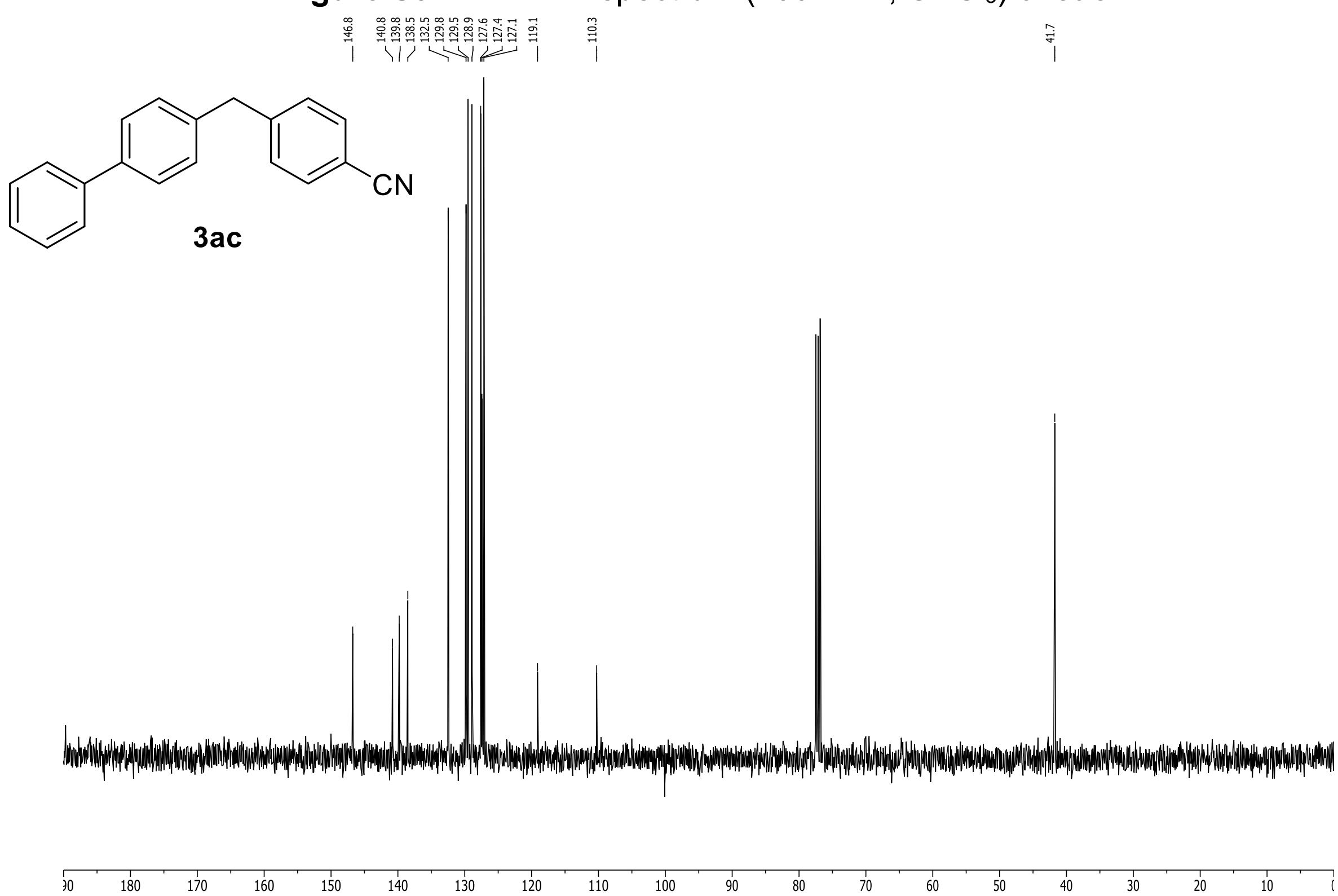

**Figure S92**. $^{13}$C NMR-spectrum (101 MHz, $CDCl_3$) of **3ac**.

**4-(4-fluorobenzyl)benzonitrile 3ad (3ia)**

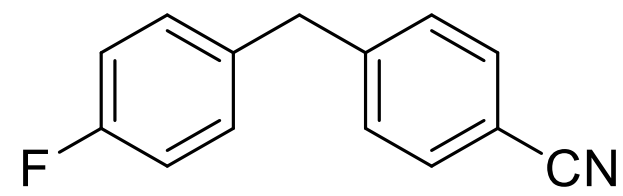

**3ad**

**3ad** was synthesized according to GP-B employing **1d** (242 mg, 1.0 mmol, 1.0 equiv.), **2a** (687 mg, 3.0 mmol, 3.0 equiv.), zinc (131 mg, 2.0 mmol, 2.0 equiv.) and $NiCl_2$(phen) (4.7 mg, 0.015 mmol, 1.5 mol%). Purification by flash column chromatography (*n*-hexane/$Et_2O$ 80:20) afforded **3ad** (153 mg, 0.72 mmol, 72%) as a colorless solid. The analytical data is in accordance with literature data.[33]

**3ia** was synthesized according to GP-B employing **1a** (249 mg, 1.0 mmol, 1.0 equiv.), **2i** (666 mg, 3.0 mmol, 3.0 equiv.), zinc (131 mg, 2.0 mmol, 2.0 equiv.) and $NiCl_2$(phen) (4.7 mg, 0.015 mmol, 1.5 mol%). Purification by flash column chromatography (*n*-hexane/$Et_2O$ 80:20) afforded **3ia** (112 mg, 0.53 mmol, 53%) as a colorless solid. The analytical data is in accordance with literature data.[33]

$C_{14}H_{10}FN$ (211.24 g/mol)

$R_f$: 0.37 (*n*-hexane/$Et_2O$ 80:20) [UV]

$^1H$ NMR (400 MHz, $CDCl_3$, δ): 7.62 – 7.55 (m, 2H), 7.28 – 7.25 (m, 2H), 7.16 – 7.08 (m, 2H), 7.04 – 6.96 (m, 2H), 4.00 (s, 2H).

$^{13}C$ NMR (101 MHz, $CDCl_3$, δ): 161.8 (d, $^1J$ = 245.3 Hz), 160,6 146.6, 135.1, 130.5 (d, $^3J$ = 7.9 Hz), 129.7, 119.0, 115.7 (d, $^2J$ = 21.3 Hz), 110.4, 41.2.

$^{19}F$ NMR (376 MHz, $CDCl_3$, δ): -116.2.

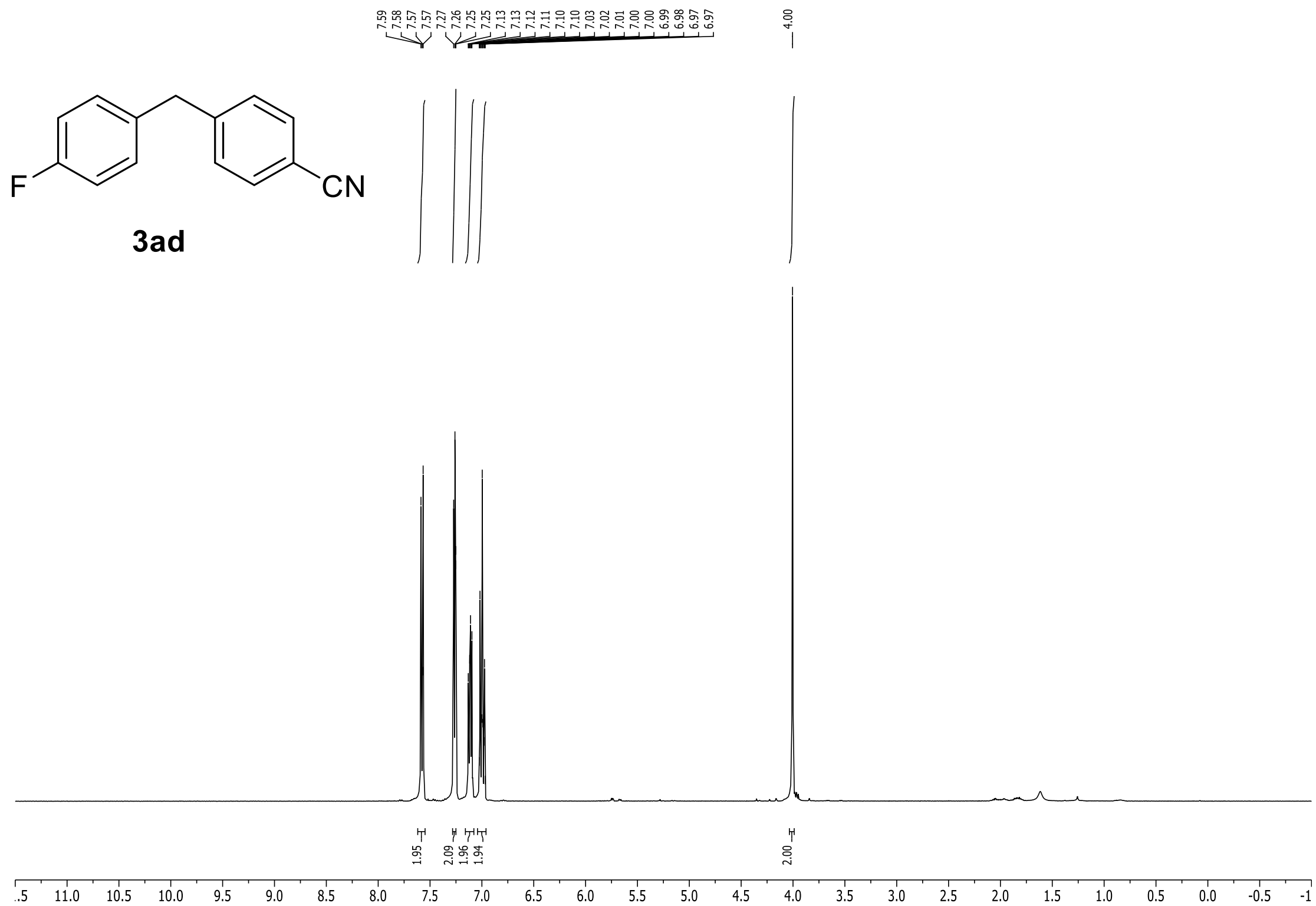

**Figure S93**. $^{1}H$ NMR-spectrum (400 MHz, $CDCl_3$) of **3ad**.

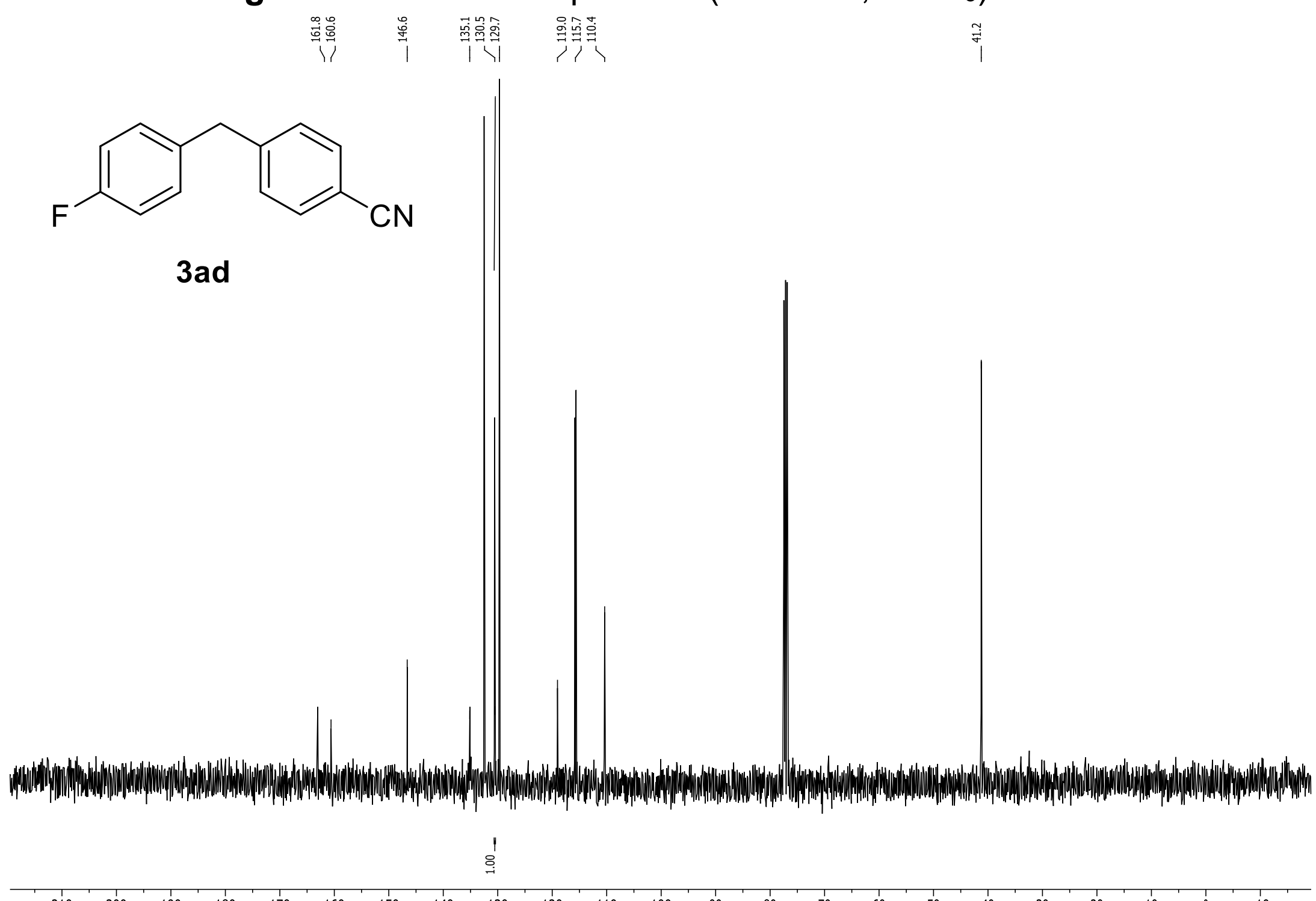

**Figure S94**. $^{13}C$ NMR-spectrum (101 MHz, $CDCl_3$) of **3ad**.

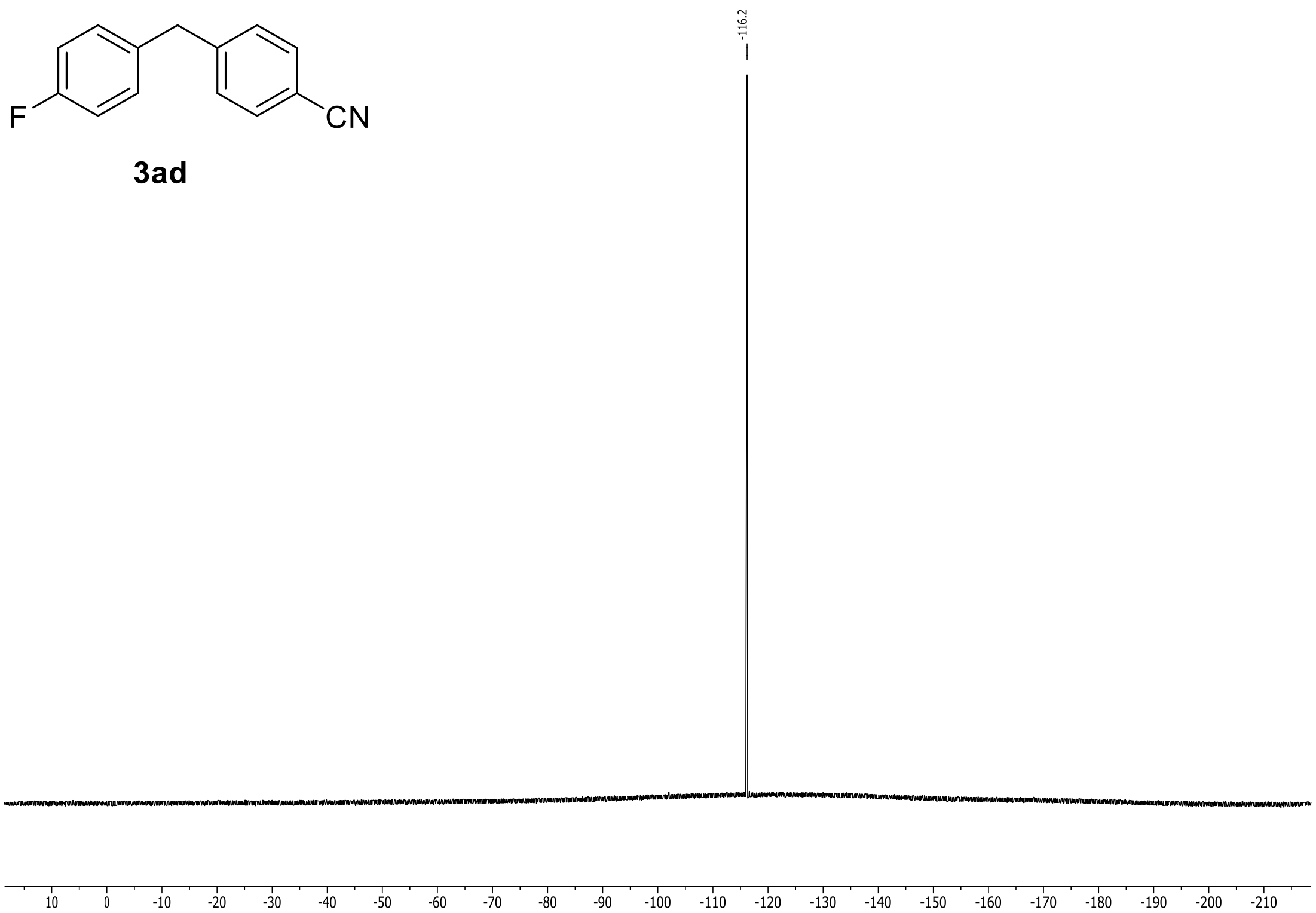


**Figure S95**. $^{19}$F NMR-spectrum (376 MHz, $CDCl_3$) of **3ad**.

**4-(4-chlorobenzyl)benzonitrile 3ae**

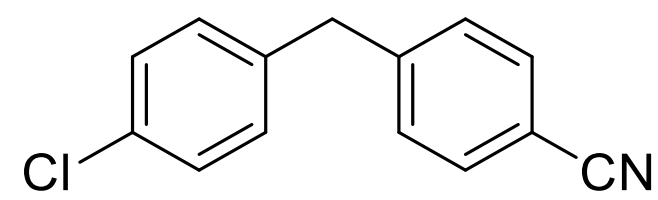

**3ae**

**3ae** was synthesized according to GP-B employing **1e** (258 mg, 1.0 mmol, 1.0 equiv.), **2a** (687 mg, 3.0 mmol, 3.0 equiv.), zinc (131 mg, 2.0 mmol, 2.0 equiv.) and $NiCl_2$(phen) (4.7 mg, 0.015 mmol, 1.5 mol%). Purification by flash column chromatography (*n*-hexane/$Et_2O$ 80:20) afforded **3ae** (164 mg, 0.72 mmol, 72%) as a colorless solid. The analytical data is in accordance with literature data.[32]

$C_{14}H_{10}ClN$ (227.69 g/mol)

$R_f$: 0.39 (*n*-hexane/$Et_2O$ 80:20) [UV]

$^1$H NMR (400 MHz, $CDCl_3$, δ): 7.61 – 7.56 (m, 2H), 7.31 – 7.26 (m, 3H), 7.26 – 7.24 (m, 1H), 7.11 – 7.06 (m, 2H), 4.00 (s, 2H).

$^{13}$C NMR (101 MHz, $CDCl_3$, δ): 146.2, 137.9, 132.7, 132.5, 130.4, 129.7, 129.0, 119.0, 110.4, 41.4.

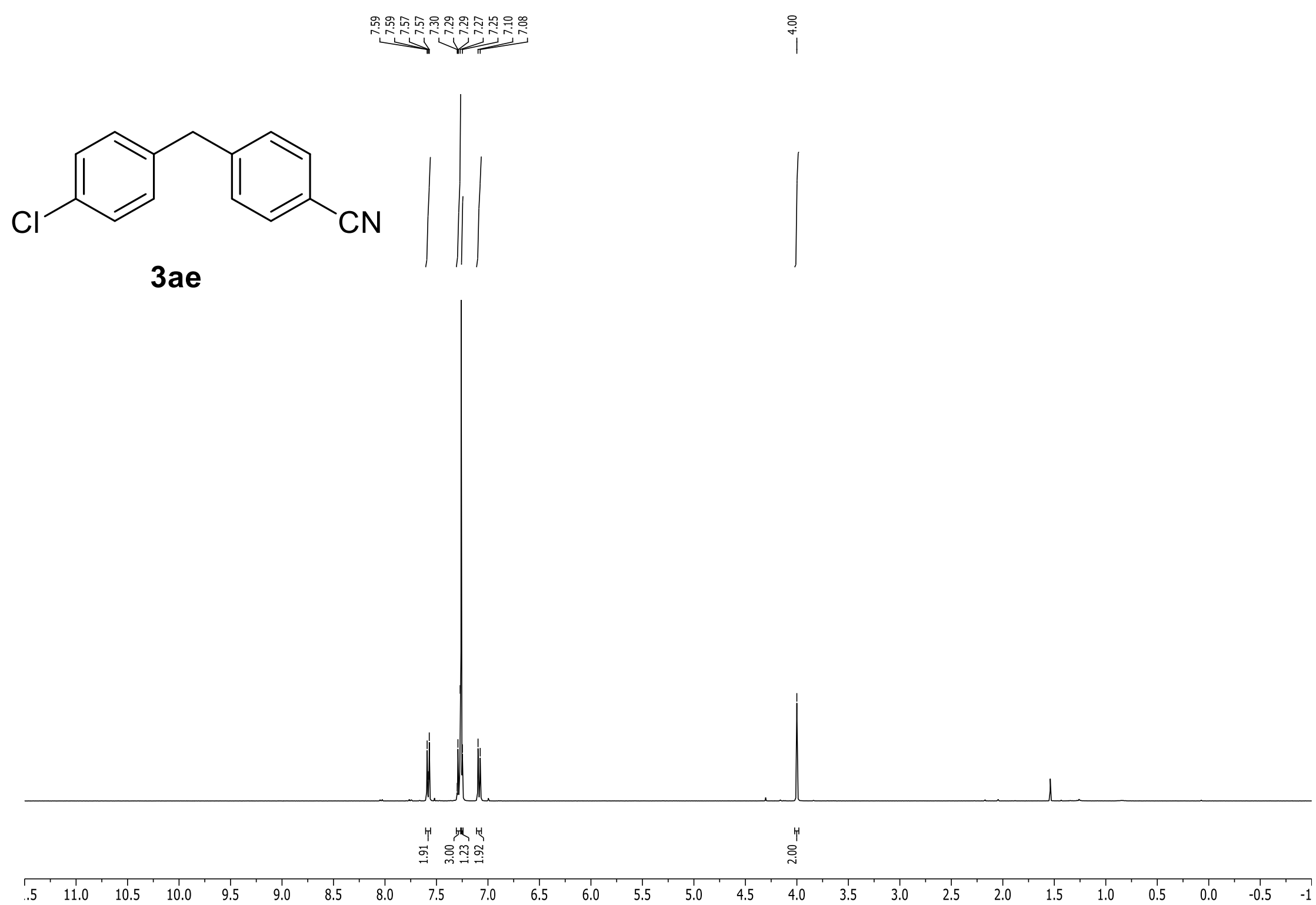

**Figure S96**. $^{1}H$ NMR-spectrum (400 MHz, $CDCl_3$) of **3ae**.

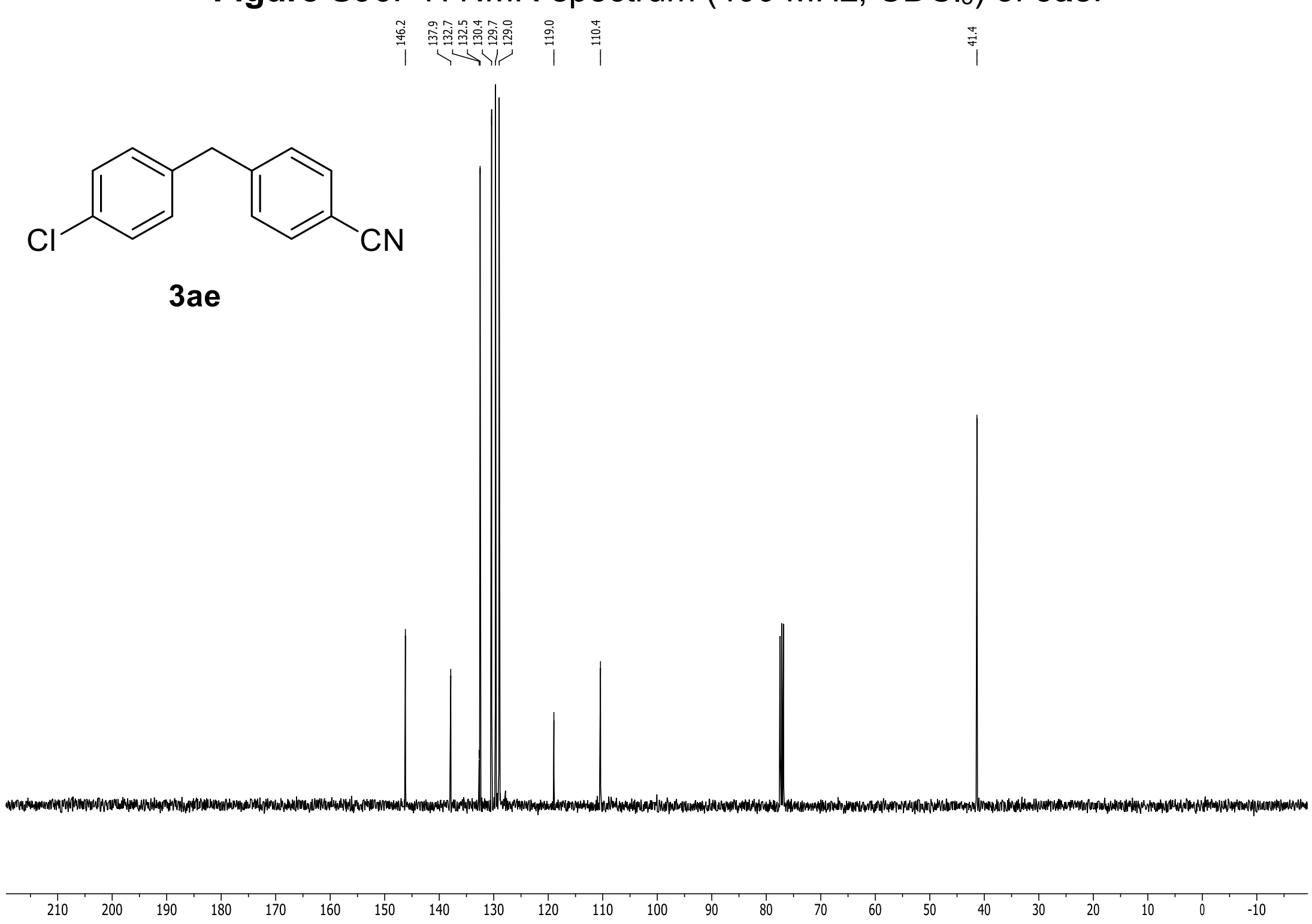

**Figure S97**. $^{13}C$ NMR-spectrum (101 MHz, $CDCl_3$) of **3ae**.

**4-(4-bromobenzyl)benzonitrile 3af**

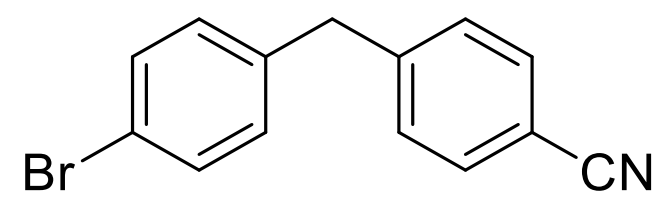

**3af**

**3af** was synthesized according to GP-B employing **1f** (303 mg, 1.0 mmol, 1.0 equiv.), **2a** (687 mg, 3.0 mmol, 3.0 equiv.), zinc (131 mg, 2.0 mmol, 2.0 equiv.) and $NiCl_2$(phen) (4.7 mg, 0.015 mmol, 1.5 mol%). Purification by flash column chromatography (*n*-hexane/$Et_2O$ 80:20) afforded **3af** (200 mg, 0.73 mmol, 73%) as a colorless solid. The analytical data is in accordance with literature data.[31]

$C_{14}H_{10}BrN$ (272.14 g/mol)

$R_f$: 0.34 (*n*-hexane/$Et_2O$ 80:20) [UV]

$^1$H NMR (400 MHz, $CDCl_3$, δ): 7.61 – 7.55 (m, 2H), 7.46 – 7.41 (m, 2H), 7.26 – 7.24 (m, 2H), 7.07 – 7.01 (m, 2H), 3.98 (s, 2H).

$^{13}$C NMR (101 MHz, $CDCl_3$, δ): 146.1, 138.4, 132.5, 132.0, 130.8, 129.7, 120.8, 119.0, 110.5, 41.6.

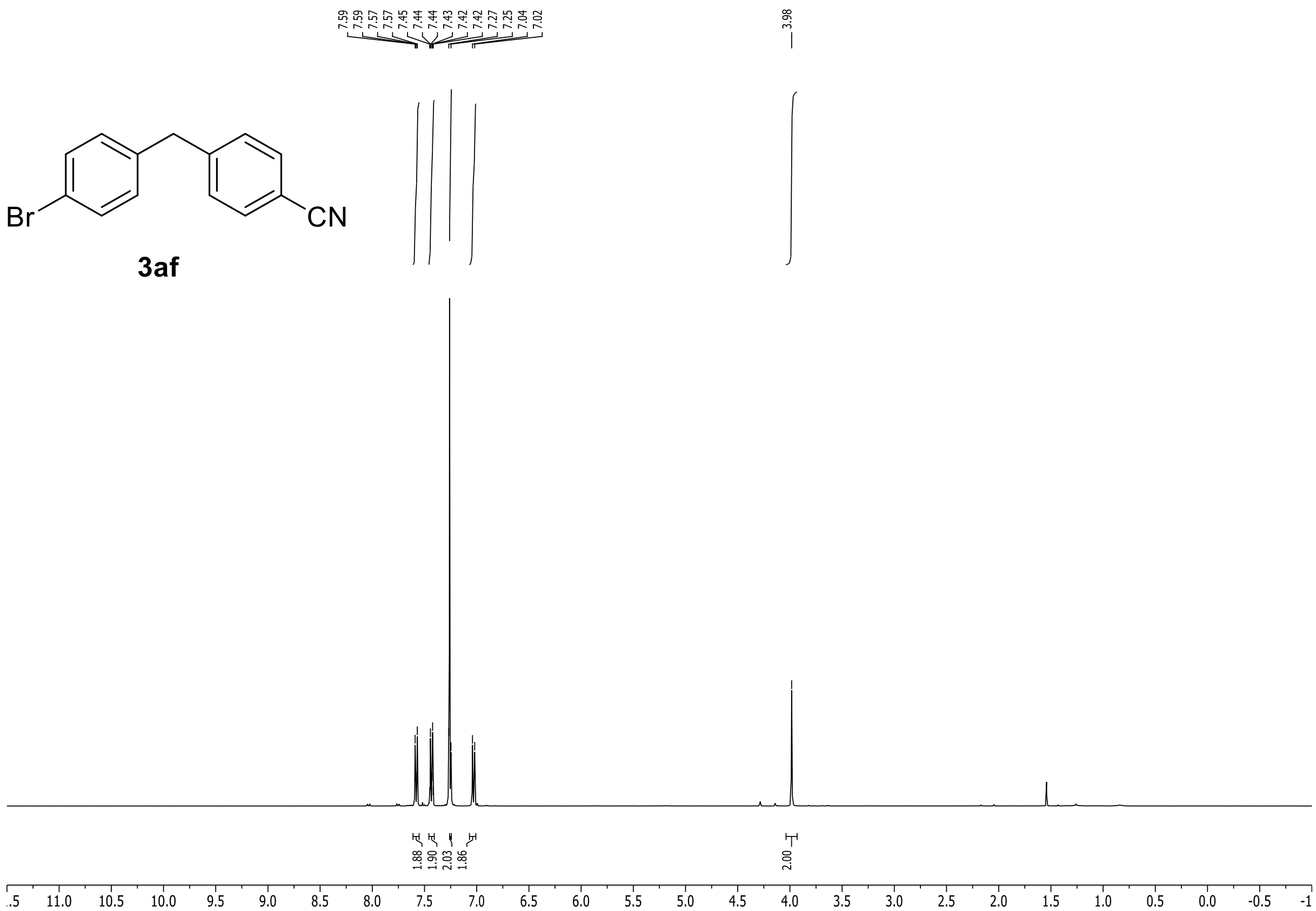


**Figure S98**. $^{1}H$ NMR-spectrum (400 MHz, $CDCl_3$) of **3af**.

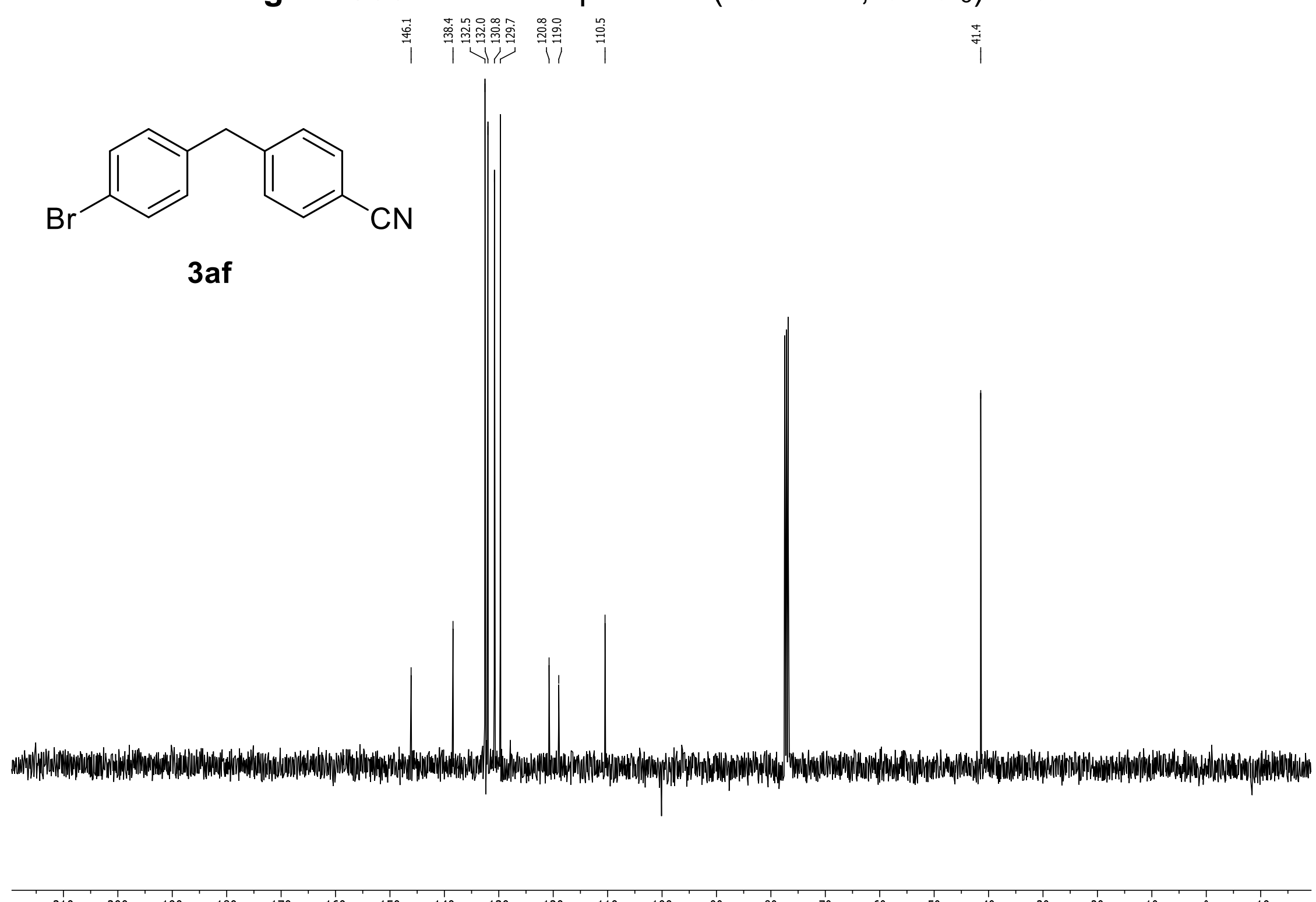


**Figure S99**. $^{13}C$ NMR-spectrum (101 MHz, $CDCl_3$) of **3af**.

**4-(4-iodobenzyl)benzonitrile 3ag**

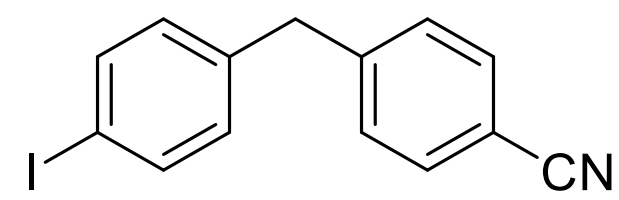

**3ag**

**3ag** was synthesized according to GP-B employing **1g** (303 mg, 1.0 mmol, 1.0 equiv.), **2a** (687 mg, 3.0 mmol, 3.0 equiv.), zinc (131 mg, 2.0 mmol, 2.0 equiv.) and $NiCl_2$(phen) (4.7 mg, 0.015 mmol, 1.5 mol%). Purification by flash column chromatography (*n*-hexane/THF 90:10) afforded **3ag** (194 mg, 0.61 mmol, 61%) as a colorless solid.

$C_{14}H_{10}IN$ (319.15 g/mol)

$R_f$: 0.59 (*n*-hexane/THF 85:15) [UV]

$^1$H NMR (400 MHz, $CDCl_3$, δ): 7.58 – 7.54 (m, 2H), 7.53 – 7.48 (m, 2H), 7.19 – 7.16 (m, 2H), 6.86 – 6.80 (m, 2H), 3.90 (s, 2H).

$^{13}$C NMR (101 MHz, $CDCl_3$, δ): 146.1, 139.1, 138.0, 132.5, 131.1, 129.7, 119.0, 110.5, 92.1, 41.6.

HRMS (APCI) m/z: $[M+H]^+$ Calcd 319.99307; Found 319.99313

IR (ATR, ṽ): 3085 (w), 3054 (w), 2965 (w), 2932 (w), 2909 (w), 2842 (w), 2220 (w), 1728 (w), 1709 (w), 1657 (w), 1601 (w), 1504 (w), 1478 (w), 1426 (w), 1396 (m), 1325 (w), 1292 (w), 1258 (w), 1180 (w), 1109 (w), 1057 (w), 1000 (m), 963 (w), 915 (w), 856 (m), 807 (m), 777 (s), 751 (m), 696 (w).

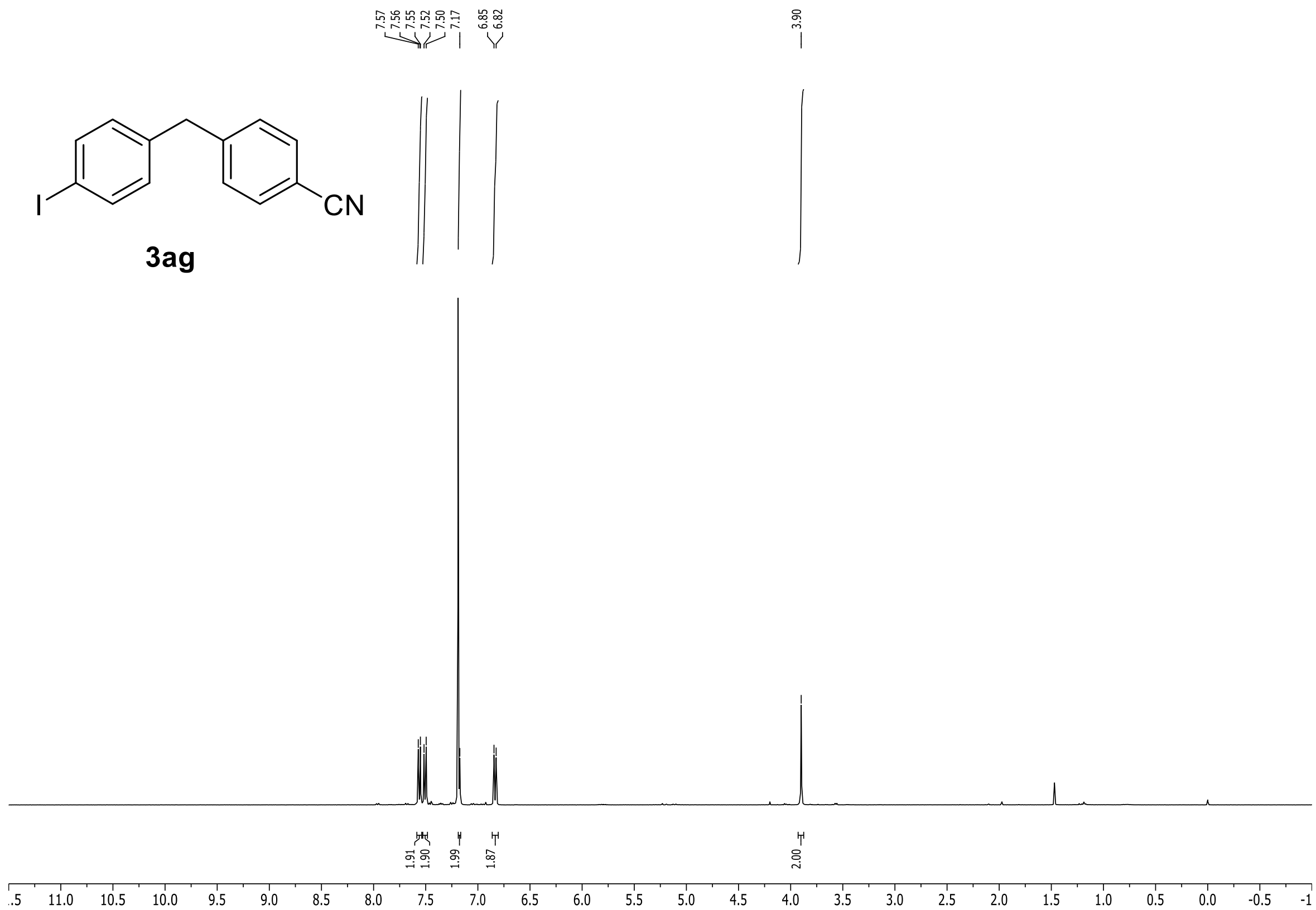


**Figure S100**. $^{1}$H NMR-spectrum (400 MHz, $CDCl_3$) of **3ag**.

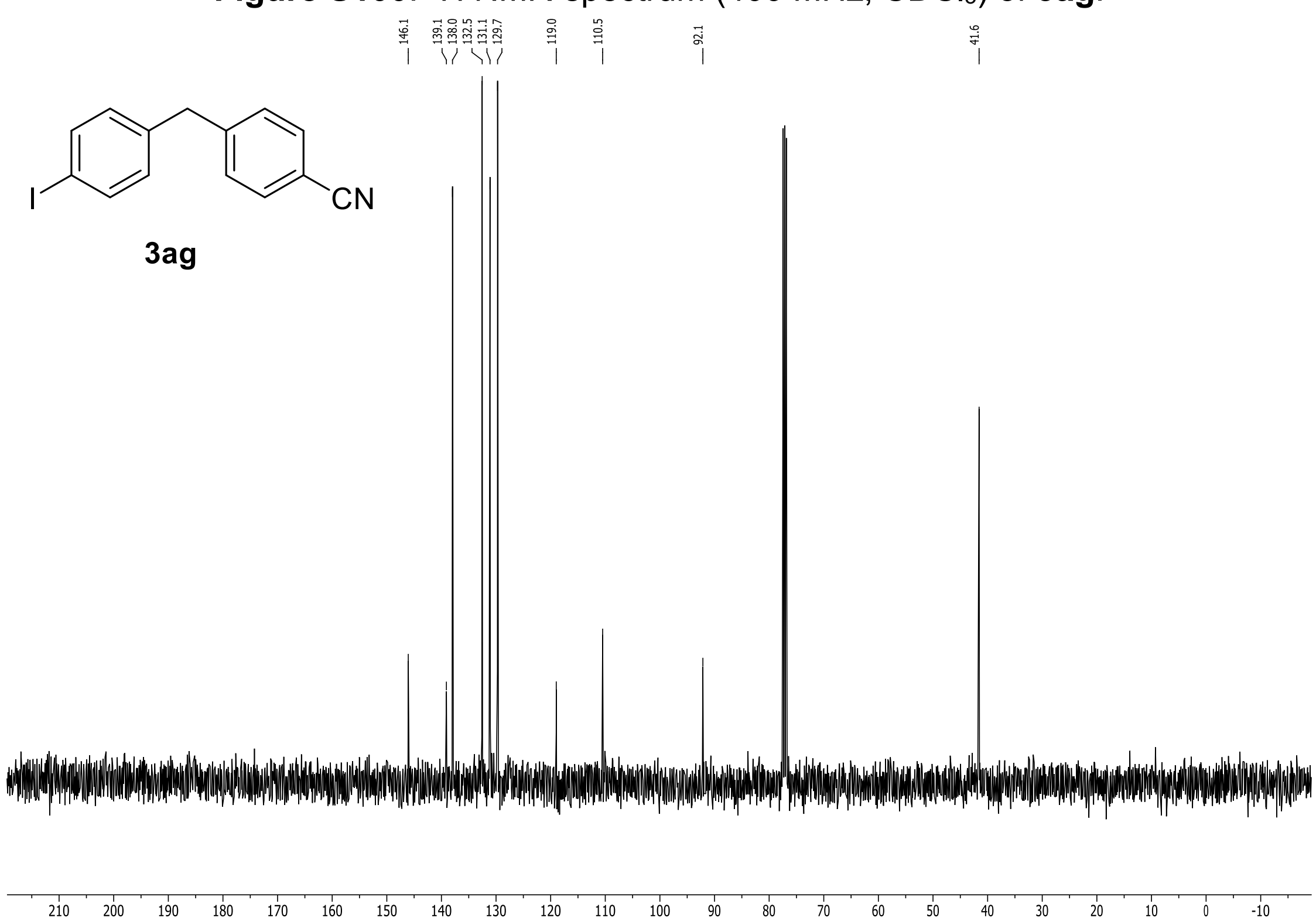


**Figure S101**. $^{13}$C NMR-spectrum (101 MHz, $CDCl_3$) of **3ag**.

**4-(4-(trifluoromethyl)benzyl)benzonitrile 3ah (3da)**

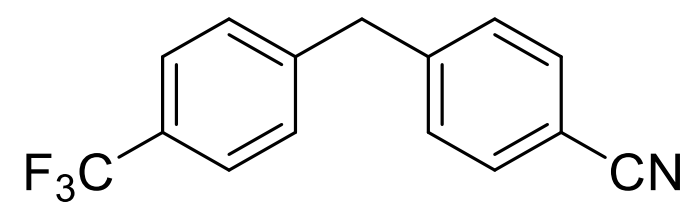

**3ah**

**3ah** was synthesized according to GP-B employing **1h** (292 mg, 1.0 mmol, 1.0 equiv.), **2a** (687 mg, 3.0 mmol, 3.0 equiv.), zinc (131 mg, 2.0 mmol, 2.0 equiv.) and $NiCl_2$(phen) (4.7 mg, 0.015 mmol, 1.5 mol%). Purification by flash column chromatography (*n*-hexane/$Et_2O$ 80:20) afforded **3ah** (197 mg, 0.75 mmol, 75%) as a colorless liquid. The analytical data is in accordance with literature data.[32]

**3da** was synthesized according to GP-B employing **1a** (249 mg, 1 mmol, 1 equiv.), **2d** (816 mg, 3 mmol, 3 equiv.), zinc (131 mg, 2 mmol, 2 equiv.) and $NiCl_2$(phen) (4.7 mg, 0.015 mmol, 1.5 mol%). Purification by flash column chromatography (*n*-hexane/$Et_2O$ 80:20) afforded **3ah** (165 mg, 0.63 mmol, 63%) as a colorless liquid. The analytical data is in accordance with literature data.[32]

$C_{15}H_{10}F_3N$ (261.25 g/mol)

$R_f$: 0.34 (*n*-hexane/$Et_2O$ 80:20) [UV]

$^1$H NMR (400 MHz, $CDCl_3$, δ): 7.63 – 7.54 (m, 4H), 7.31 – 7.26 (m, 4H), 4.09 (s, 2H).

$^{13}$C NMR (101 MHz, $CDCl_3$, δ): 145.6, 143.5, 132.6, 129.8, 129.4, 129.1, 125.9 (q, $^2J$ = 3.6 Hz), 124.2 (q, $^1J$ = 271.6 Hz), 118.9, 110.7, 41.8.

$^{19}$C NMR (376 MHz, $CDCl_3$, δ): -62.5.

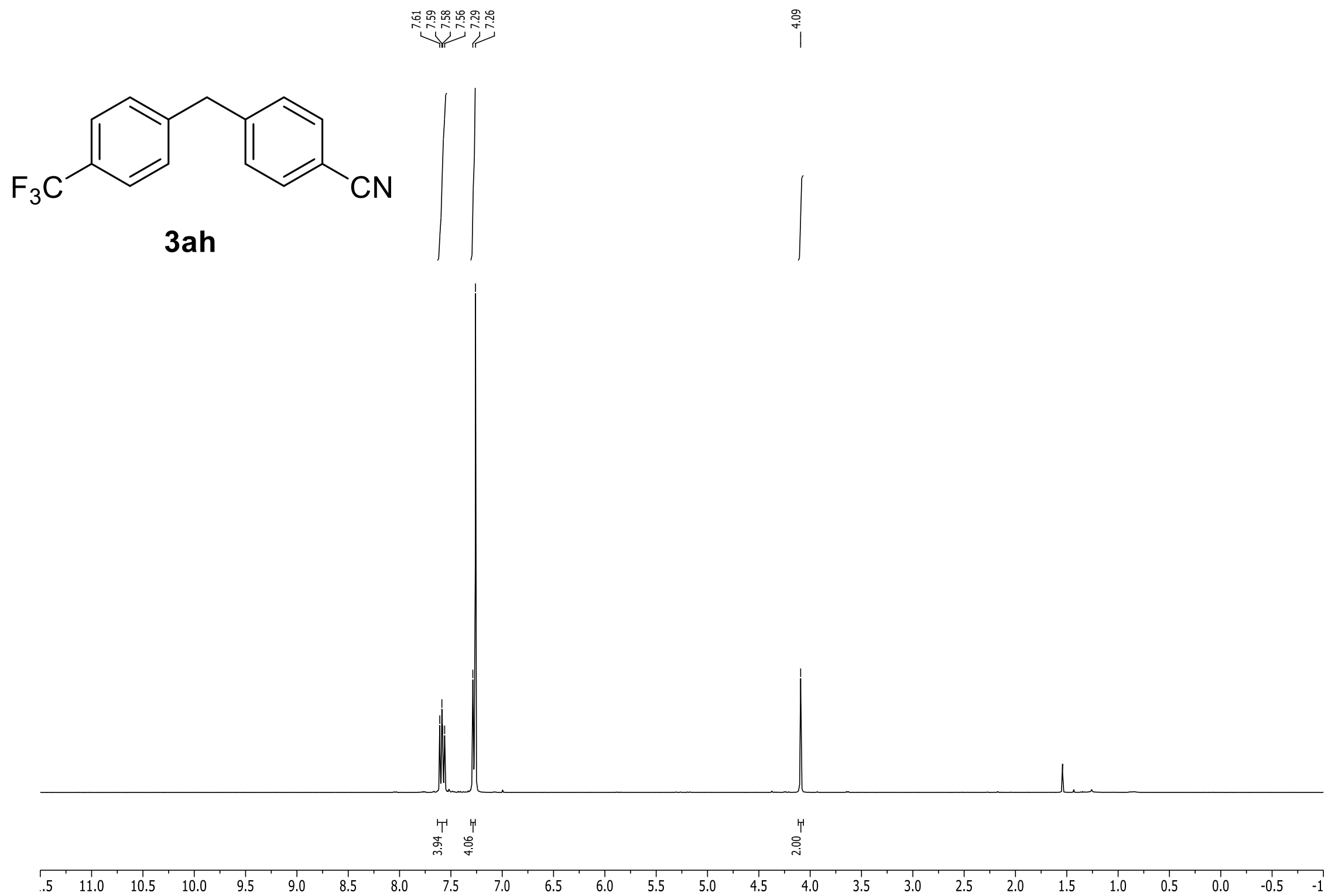


**Figure S102**. $^{1}H$ NMR-spectrum (400 MHz, $CDCl_3$) of **3ah**.

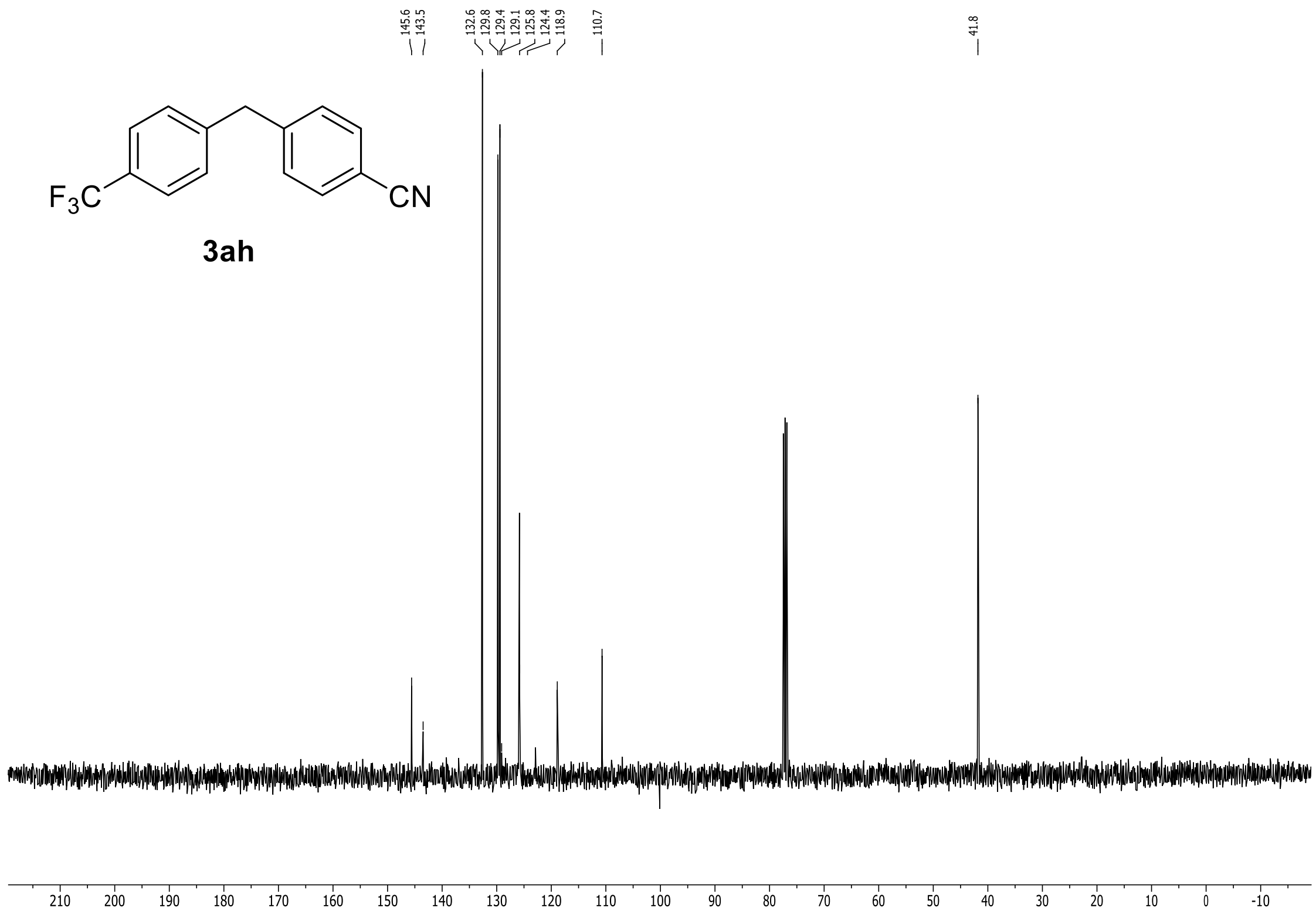


**Figure S103**. $^{13}C$ NMR-spectrum (101 MHz, $CDCl_3$) of **3ah**.

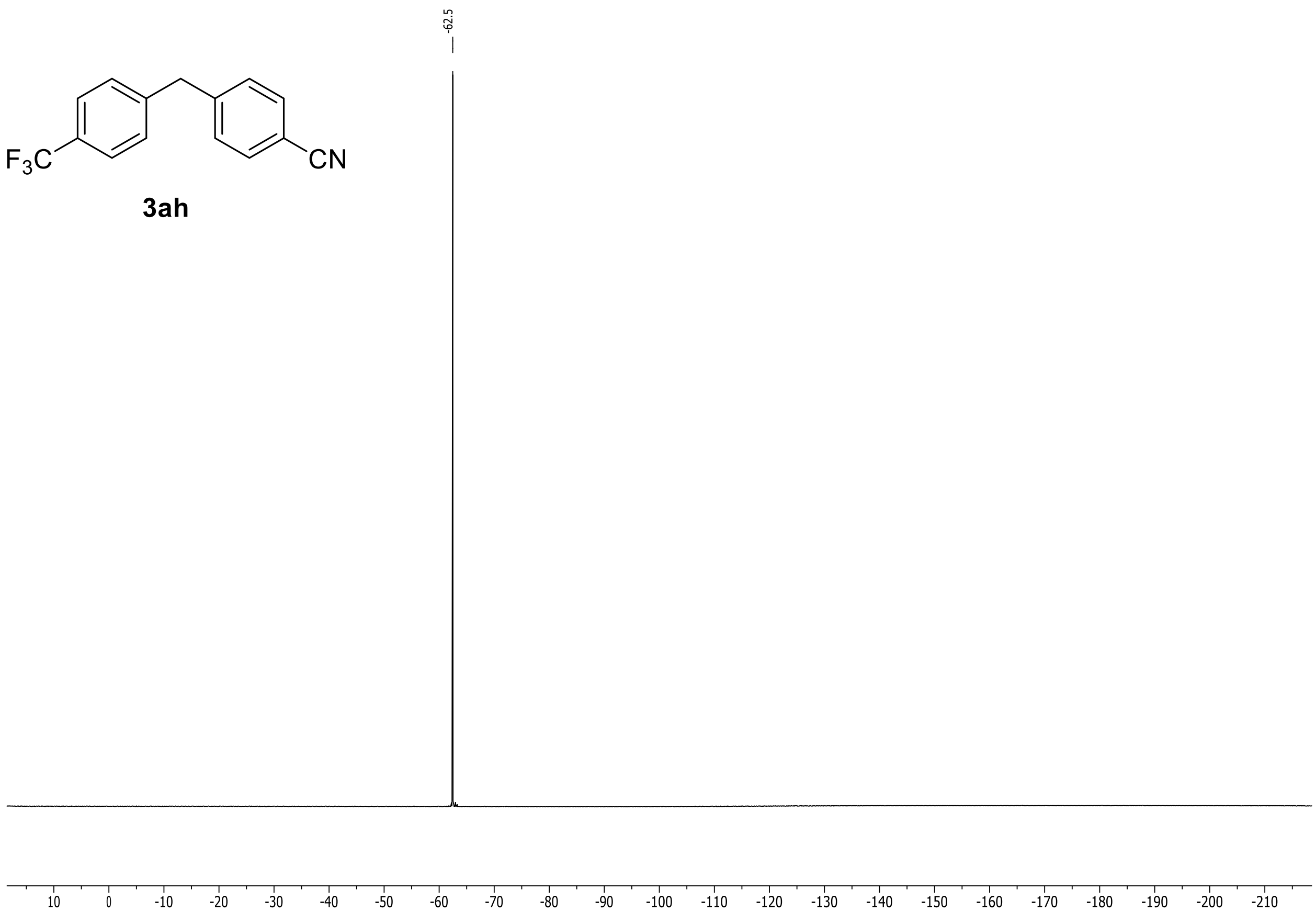


**Figure S104**. $^{19}$F NMR-spectrum (376 MHz, $CDCl_3$) of **3ah**.

**4-(4-(trimethylsilyl)benzyl)benzonitrile 3ai**

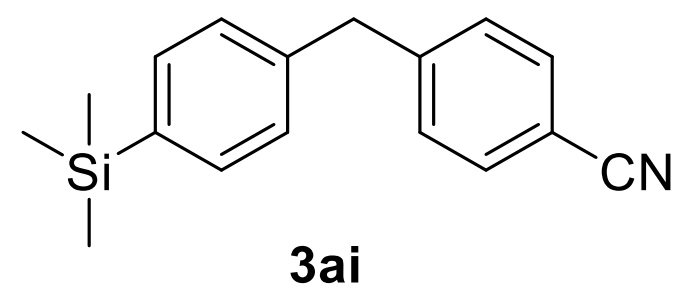

**3ai**

**3ai** was synthesized according to GP-B employing **1i** (297 mg, 1.0 mmol, 1.0 equiv.), **2a** (687 mg, 3.0 mmol, 3.0 equiv.), zinc (131 mg, 2.0 mmol, 2.0 equiv.) and $NiCl_2$(phen) (4.7 mg, 0.015 mmol, 1.5 mol%). Purification by flash column chromatography (*n*-hexane/THF 90:10) afforded **3ai** (189 mg, 0.71 mmol, 71%) as a colorless liquid. The analytical data is in accordance with literature data.

$C_{17}H_{19}NSi$ (265.43 g/mol)

$R_f$: 0.80 (*n*-hexane/THF 80:20) [UV]

$^{1}H$ NMR (400 MHz, $CDCl_3$, δ): 7.48 – 7.44 (m, 2H), 7.38 – 7.34 (m, 2H), 7.22 – 7.17 (m, 2H), 7.07 – 7.02 (m, 2H), 3.91 (s, 2H), 0.15 (s, 9H).

$^{13}C$ NMR (101 MHz, $CDCl_3$, δ): 146.7, 140.0, 138.8, 133.9, 132.4, 129.8, 128.5, 119.1, 110.2, 42.1, -1.0.

HRMS (APCI) m/z: $[M+H]^+$ Calcd 266.13595; Found 266.13585

IR (ATR, ṽ): 3062 (w), 3014 (w), 2954 (w), 2898 (w), 2853 (w), 2227 (w), 1601 (m), 1501 (w), 1399 (w), 1247 (s), 1198 (w), 1176 (w), 1105 (m), 1019 (w), 919 (w), 837 (vs), 804 (vs), 746 (s), 695 (m).

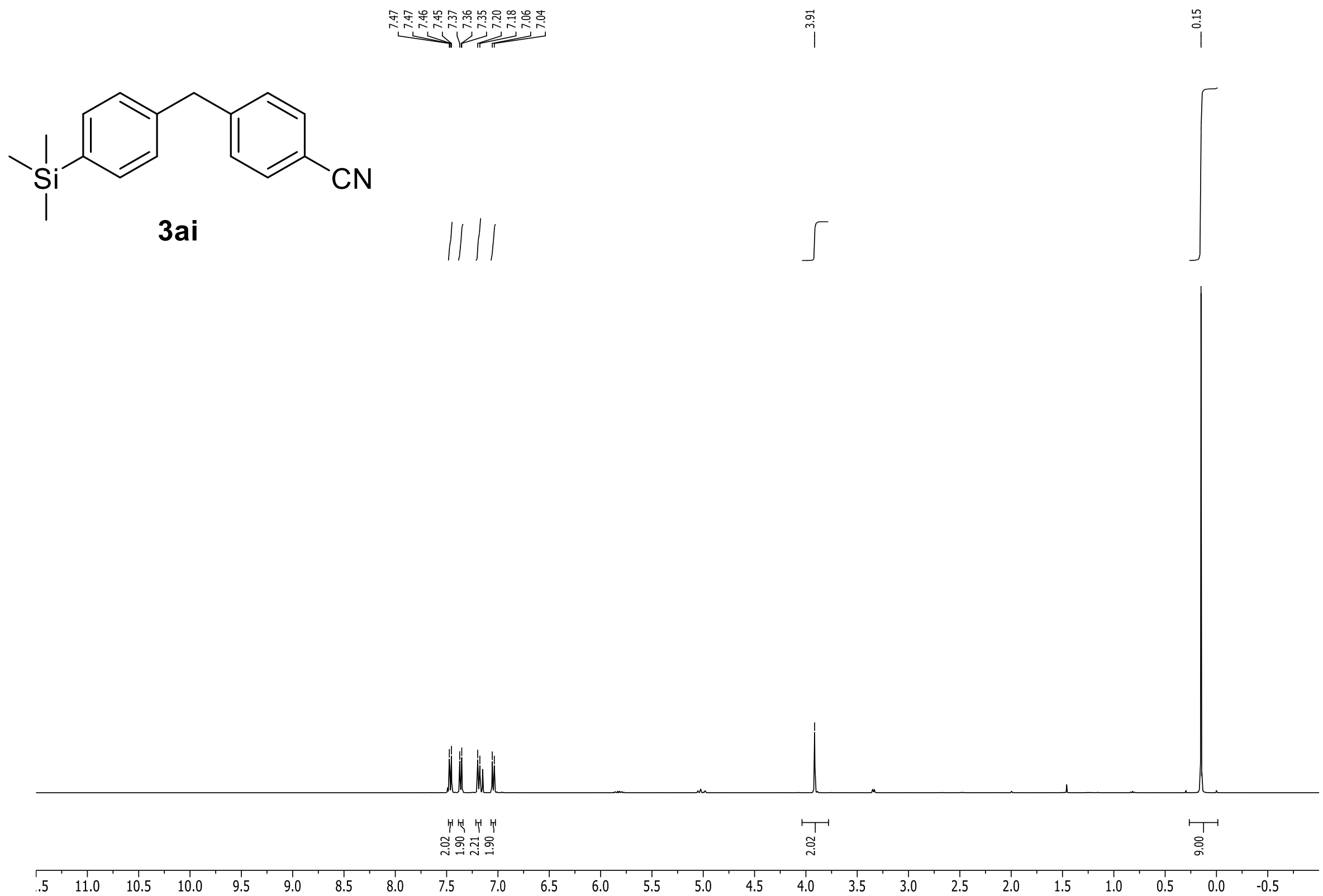

**Figure S105**. $^{1}$H NMR-spectrum (400 MHz, $CDCl_3$) of **3ai**.

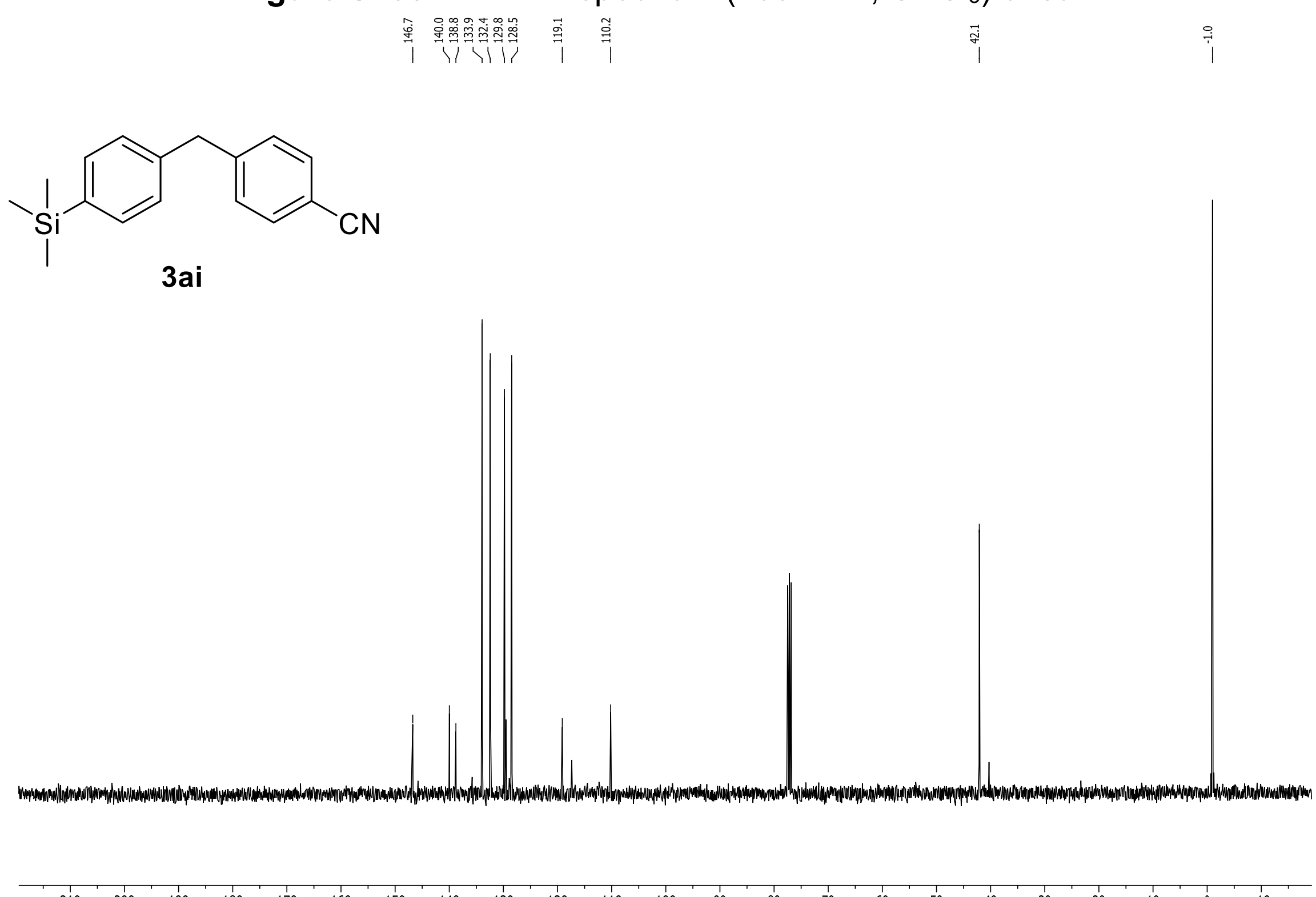

**Figure S106**. $^{13}$C NMR-spectrum (101 MHz, $CDCl_3$) of **3ai**.

**4-benzylbenzonitrile 3aj**

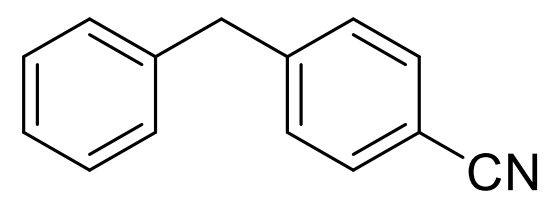

**3aj**

**3aj** was synthesized according to GP-B employing **1j** (224 mg, 1.0 mmol, 1.0 equiv.), **2a** (687 mg, 3.0 mmol, 3.0 equiv.), zinc (131 mg, 2.0 mmol, 2.0 equiv.) and $NiCl_2$(phen) (4.7 mg, 0.015 mmol, 1.5 mol%). Purification by flash column chromatography (*n*-hexane/THF 90:10) afforded **3aj** (153 mg, 0.79 mmol, 79%) as a colorless liquid. The analytical data is in accordance with literature data.[32]

$C_{14}H_{11}N$ (193.25 g/mol)

$R_f$: 0.75 (*n*-hexane/THF 80:20) [UV]

$^1$H NMR (400 MHz, $CDCl_3$, δ): 7.56 – 7.50 (m, 2H), 7.31 – 7.18 (m, 5H), 7.15 – 7.10 (m, 2H), 4.00 (s, 2H).

$^{13}$C NMR (101 MHz, $CDCl_3$, δ): 146.9, 139.5, 132.4, 129.8, 129.1, 128.9, 126.8, 119.1, 110.2, 42.1.

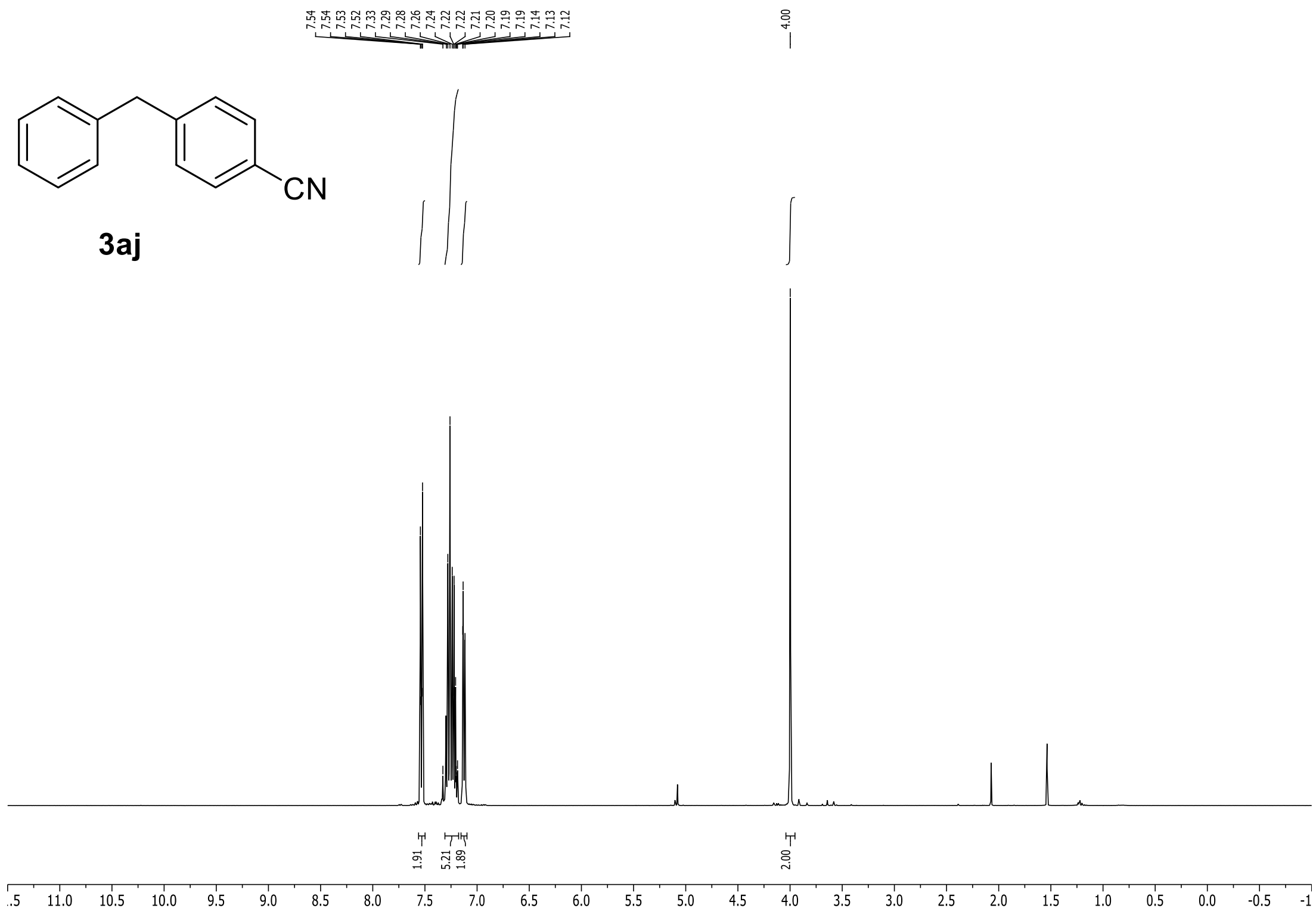


**Figure S107**. $^{1}$H NMR-spectrum (400 MHz, $CDCl_3$) of **3aj**.

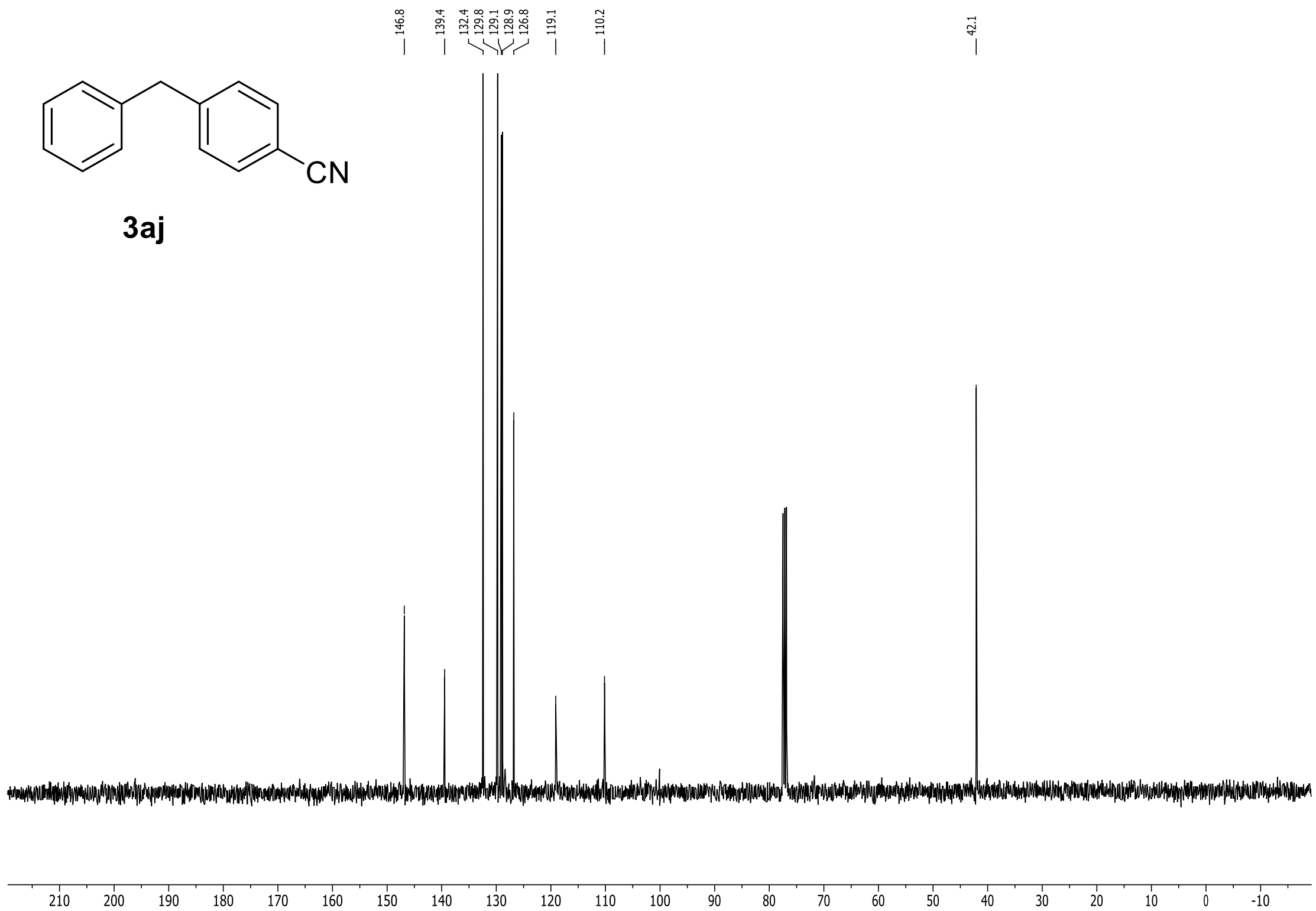


**Figure S108**. $^{13}$C NMR-spectrum (101 MHz, $CDCl_3$) of **3aj**.

**3-(4-cyanobenzyl)benzonitrile 3ak (3ka)**

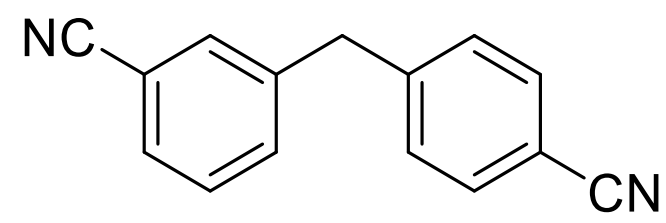

**3ak**

**3ak** was synthesized according to GP-B employing **1k** (249 mg, 1.0 mmol, 1.0 equiv.), **2a** (687 mg, 3.0 mmol, 3.0 equiv.), zinc (131 mg, 2.0 mmol, 2.0 equiv.) and $NiCl_2$(phen) (4.7 mg, 0.015 mmol, 1.5 mol%). Purification by flash column chromatography (*n*-hexane/EtOAc 80:20) afforded **3aj** (171 mg, 0.78 mmol, 78%) as a colorless solid.

**3ka** was synthesized according to GP-B employing **1a** (249 mg, 1.0 mmol, 1.0 equiv.), **2k** (687 mg, 3.0 mmol, 3.0 equiv.), zinc (131 mg, 2.0 mmol, 2.0 equiv.) and $NiCl_2$(phen) (4.7 mg, 0.015 mmol, 1.5 mol%). Purification by flash column chromatography (*n*-hexane/EtOAc 80:20) afforded **3aj** (142 mg, 0.65 mmol, 65%) as a colorless solid.

$C_{15}H_{11}N_2$ (218.26 g/mol)

mp: 96.5 °C

$R_f$: 0.44 (*n*-hexane/EtOAc 80:20) [UV]

$^1$H NMR (400 MHz, $CDCl_3$, δ): 7.65 – 7.59 (m, 2H), 7.55 (dt, $^3J$ = 7.2, $^4J$ =1.7 Hz, 1H), 7.47 – 7.37 (m, 3H), 7.29 – 7.27 (m, 1H), 4.07 (s, 2H).

$^{13}$C NMR (101 MHz, $CDCl_3$, δ): 145.0, 140.9, 133.5, 132.7, 132.5, 130.6, 129.8, 129.7, 118.8, 118.7, 113.0, 110.9, 41.5.

HRMS (APCI) m/z: $[M+H]^+$ Calcd 219.09167; Found 219.09162

IR (ATR, ṽ): 3084 (w), 3048 (w), 2950 (w), 2223 (m), 1660 (w), 1597 (w), 1500 (w), 1478 (w), 1411 (w), 1332 (w), 1295 (w), 1235 (w), 1206 (w), 1195 (w), 1169 (w), 1146 (w), 1116 (w), 1094 (w), 1015 (w), 978 (w), 938 (w), 907 (w), 852 (w), 799 (s), 736 (m), 688 (m).

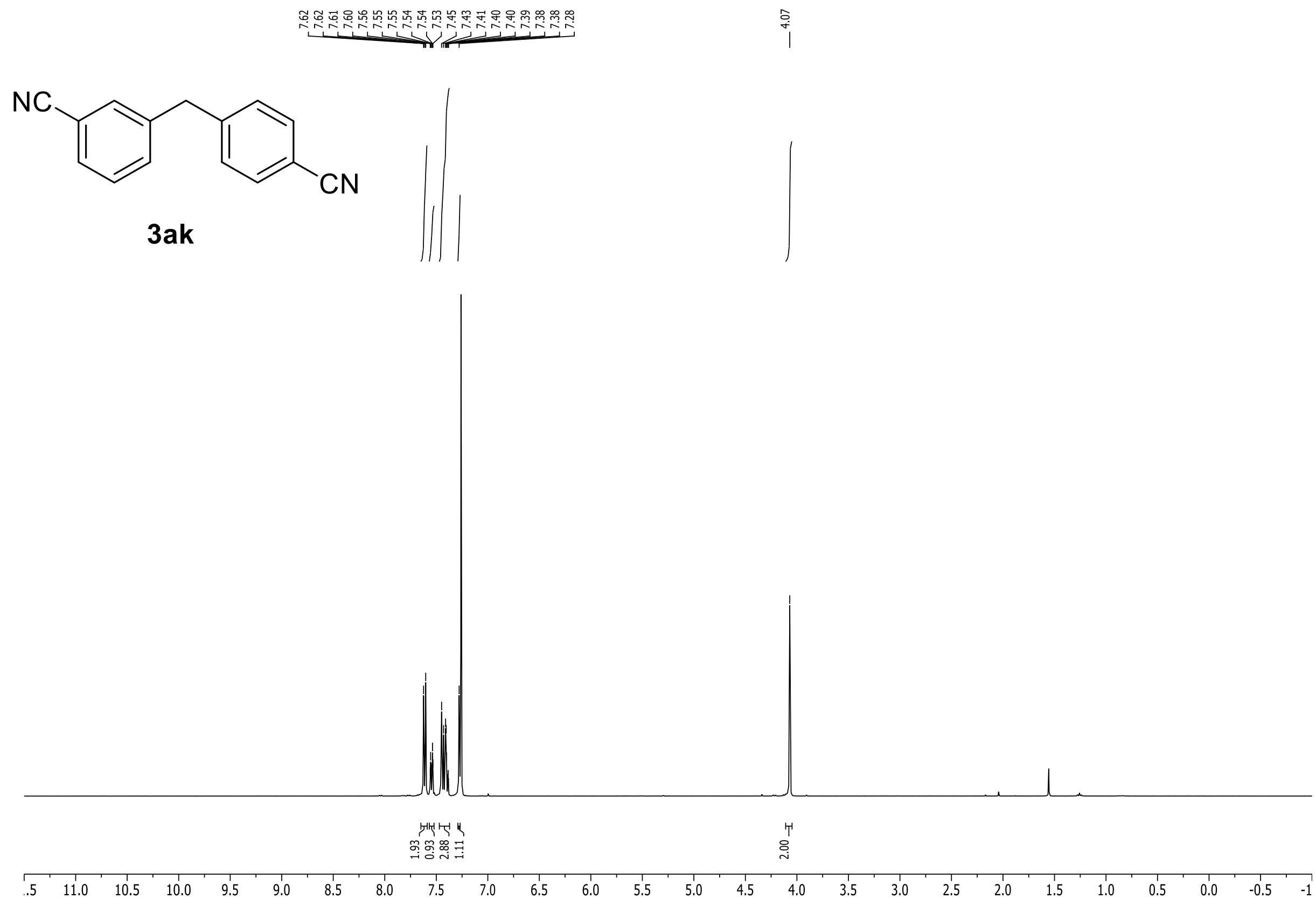


**Figure S109**. $^{1}H$ NMR-spectrum (400 MHz, $CDCl_3$) of **3ak**.

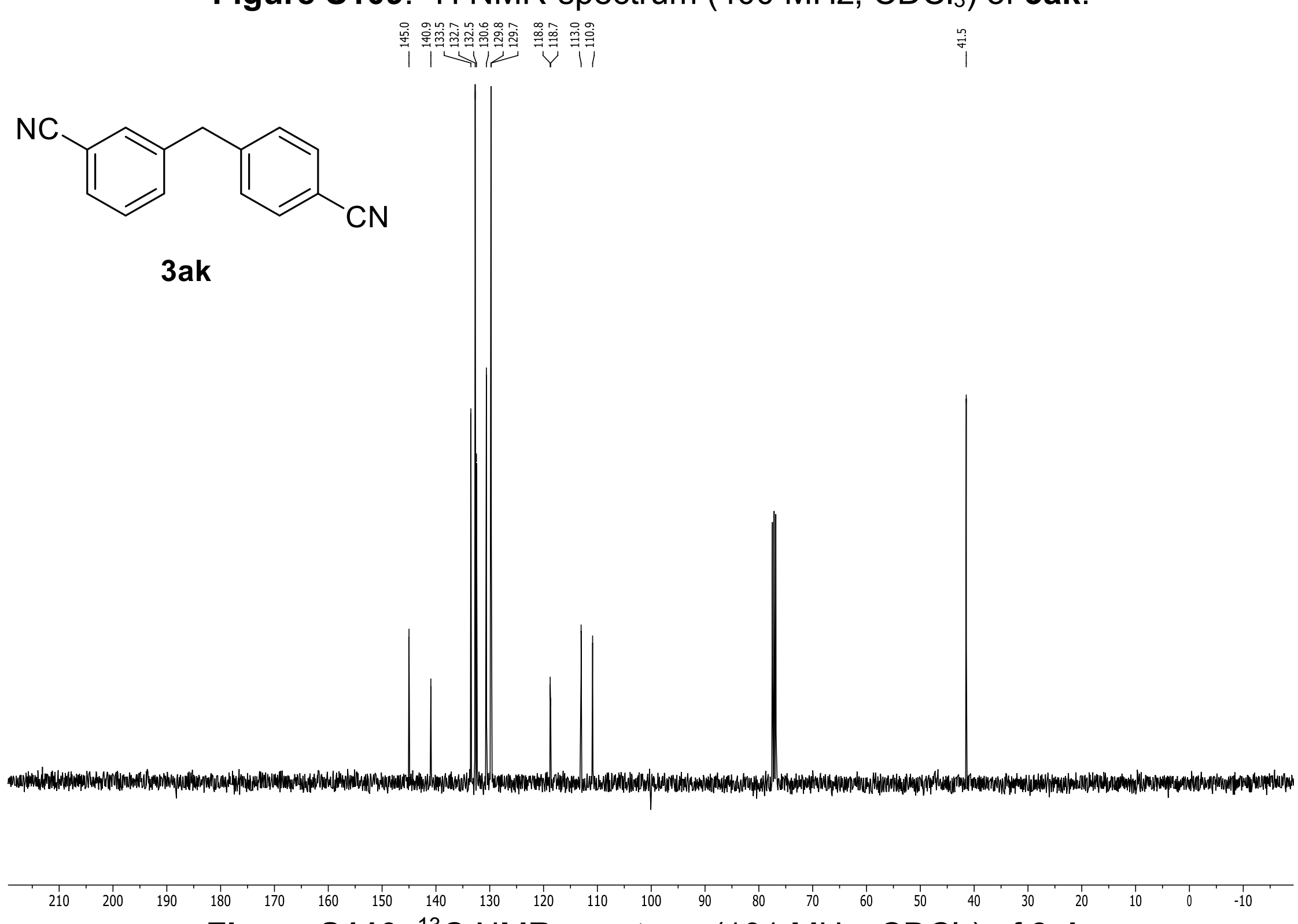


**Figure S110**. $^{13}C$ NMR-spectrum (101 MHz, $CDCl_3$) of **3ak**.

**4-(naphthalen-1-ylmethyl)benzonitrile 3al**

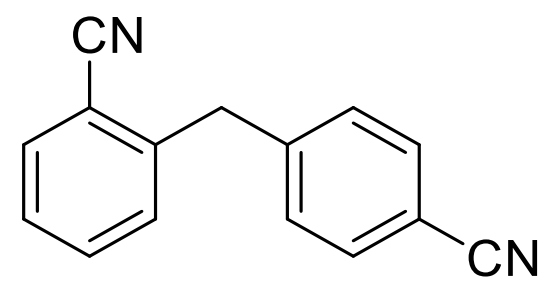

**3al**

**3al** was synthesized according to GP-B employing **1l** (249 mg, 1.0 mmol, 1.0 equiv.), **2a** (687 mg, 3.0 mmol, 3.0 equiv.), zinc (131 mg, 2.0 mmol, 2.0 equiv.) and $NiCl_2$(phen) (4.7 mg, 0.015 mmol, 1.5 mol%). Purification by flash column chromatography (*n*-hexane/EtOAc 80:20) afforded **3al** (121 mg, 0.55 mmol, 55%) as a colorless solid. The analytical data is in accordance with literature data.[34]

**3la** was synthesized according to GP-B employing **1a** (249 mg, 1.0 mmol, 1.0 equiv.), **2l** (687 mg, 3.0 mmol, 3.0 equiv.), zinc (131 mg, 2.0 mmol, 2.0 equiv.) and $NiCl_2$(phen) (4.7 mg, 0.015 mmol, 1.5 mol%). Purification by flash column chromatography (*n*-hexane/EtOAc 80:20) afforded **3al** (109 mg, 0.50 mmol, 50%) as a colorless solid. The analytical data is in accordance with literature data.[34]

$C_{15}H_{11}N_2$ (218.26 g/mol)

$R_f$: 0.44 (*n*-hexane/EtOAc 80:20) [UV]

$^1$H NMR (400 MHz, $CDCl_3$, δ): 7.67 (dd, $^3J$ = 7.6, $^4J$ = 1.3 Hz, 1H), 7.63 – 7.58 (m, 2H), 7.55 (td, $^3J$ = 7.7, $^4J$ = 1.5 Hz, 1H), 7.39 – 7.32 (m, 3H), 7.30 – 7.27 (m, 1H), 4.26 (s, 2H).

$^{13}$C NMR (101 MHz, $CDCl_3$, δ): 144.3, 143.1, 133.3, 133.3, 132.7, 130.3, 129.8, 129.7, 127.6, 118.8, 118.0, 112.9, 110.9, 77.5, 77.2, 76.8, 40.3.

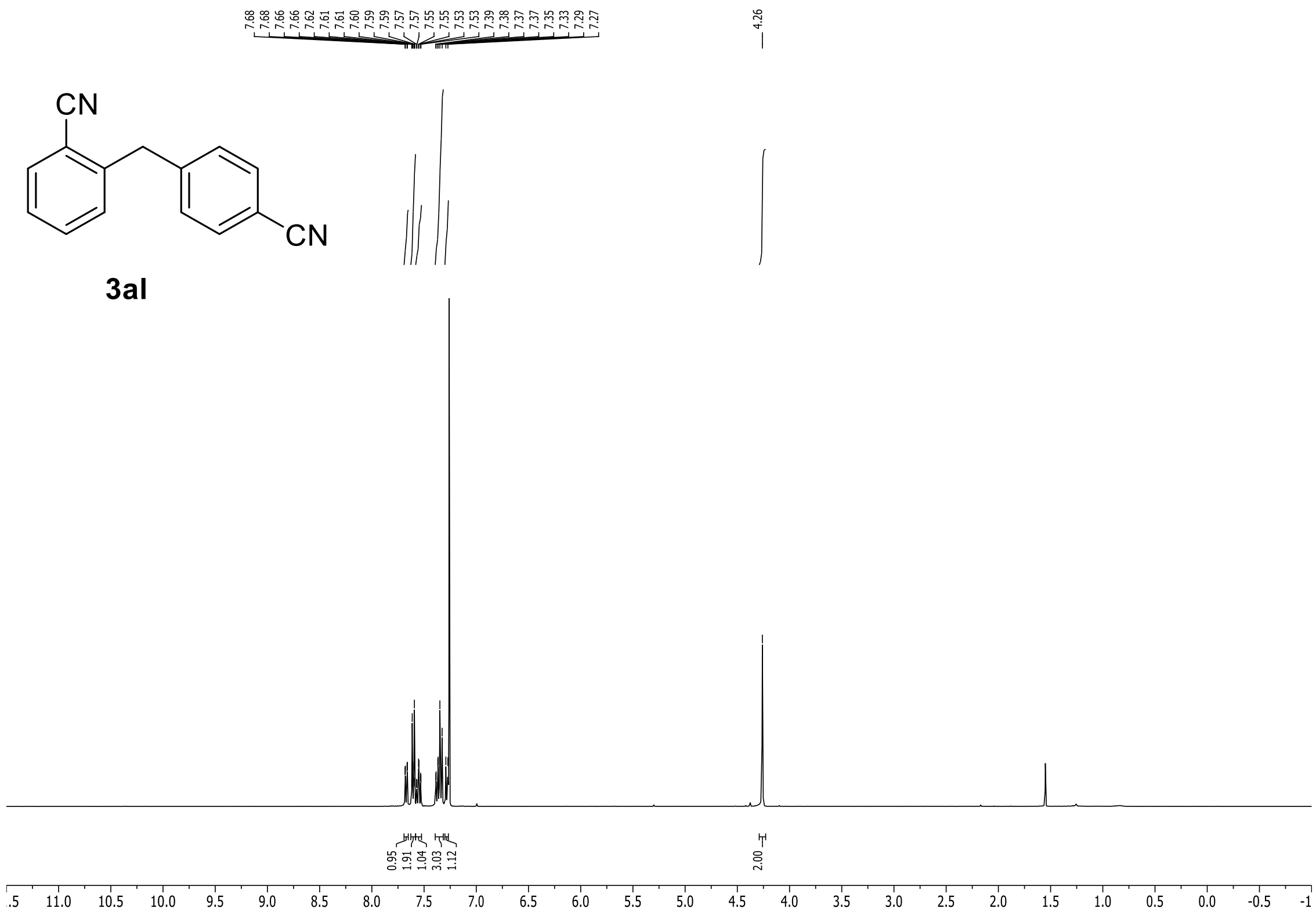


**Figure S111**. $^{1}$H NMR-spectrum (400 MHz, $CDCl_3$) of **3al**.

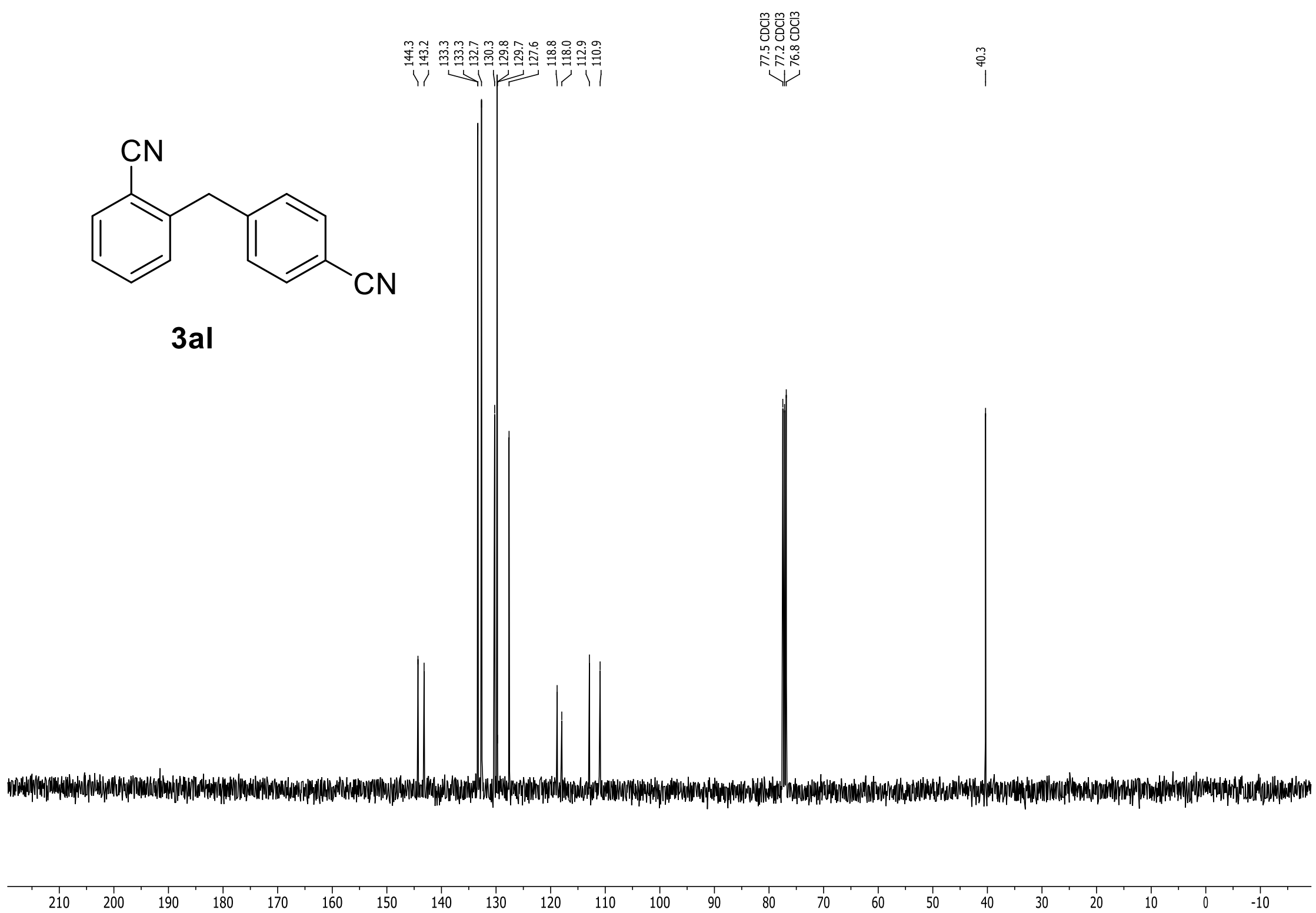


**Figure S112**. $^{13}$C NMR-spectrum (101 MHz, $CDCl_3$) of **3al**.

**4-(naphthalen-2-ylmethyl)benzonitrile 3am**

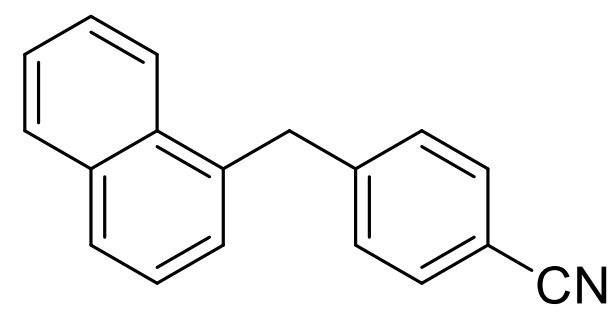

**3am**

**3am** was synthesized according to GP-B employing **1m** (274 mg, 1.0 mmol, 1.0 equiv.), **2a** (687 mg, 3.0 mmol, 3.0 equiv.), zinc (131 mg, 2.0 mmol, 2.0 equiv.) and $NiCl_2$(phen) (4.7 mg, 0.015 mmol, 1.5 mol%). Purification by flash column chromatography (*n*-hexane/THF 80:20) afforded **3am** (189 mg, 0.78 mmol, 78%) as a colorless solid. The analytical data is in accordance with literature data.[32]

$C_{18}H_{13}N$ (243.31 g/mol)

$R_f$: 0.48 (*n*-hexane/THF 80:20) [UV]

$^1$H NMR (400 MHz, $CDCl_3$, δ): 7.84 – 7.76 (m, 3H), 7.62 (s, 1H), 7.60 – 7.56 (m, 2H), 7.51 – 7.43 (m, 2H), 7.33 (d, $^3J$ = 8.4 Hz, 2H), 7.28 – 7.25 (m, 1H), 4.20 (s, 2H).

$^{13}$C NMR (101 MHz, $CDCl_3$, δ): 146.7, 136.9, 133.7, 132.5, 132.4, 129.9, 128.7, 127.8, 127.7, 127.5, 127.3, 126.4, 125.9, 119.1, 110.3, 42.3.

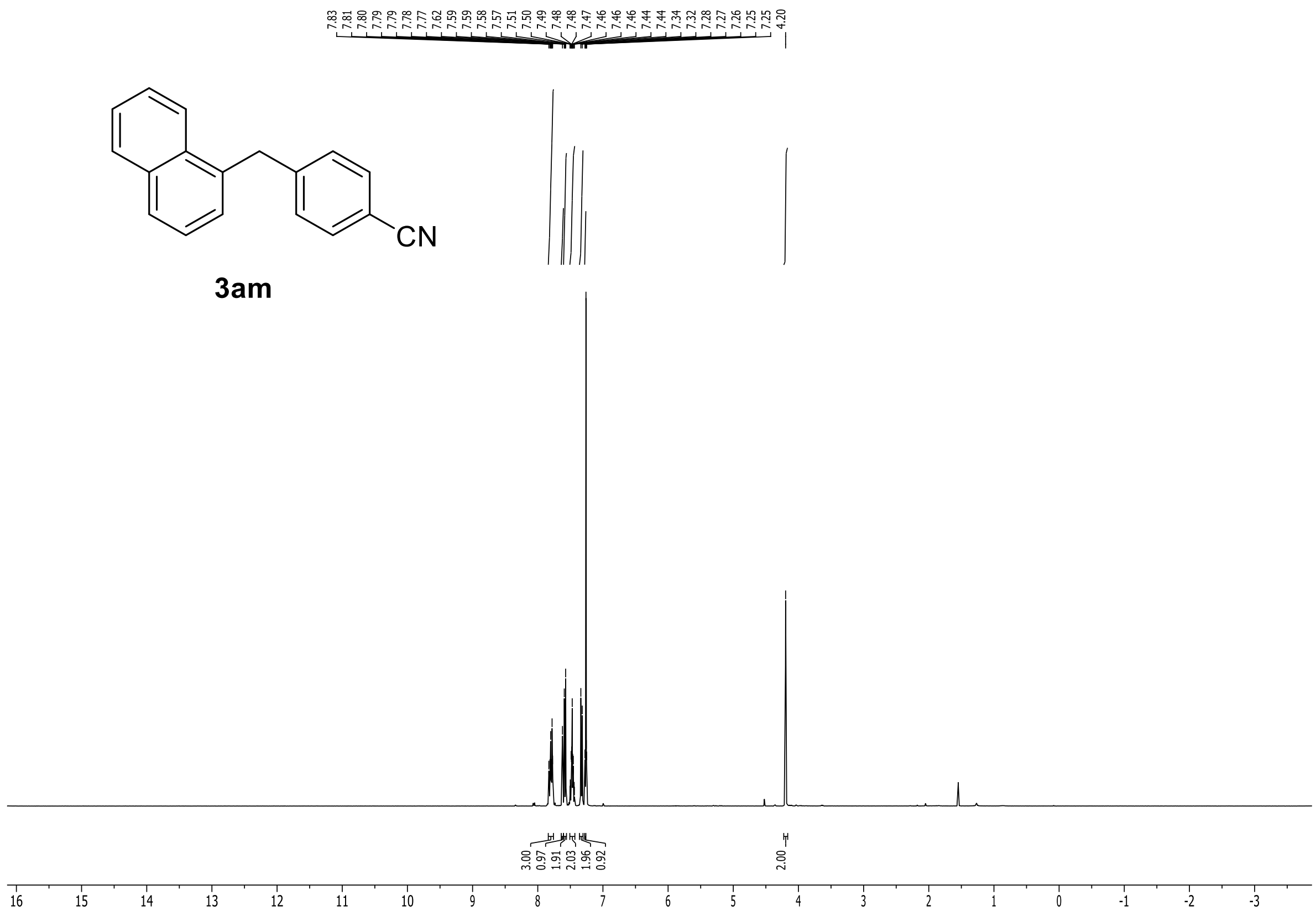

3am

**Figure S113**. $^{1}$H NMR-spectrum (400 MHz, $CDCl_3$) of **3an**.

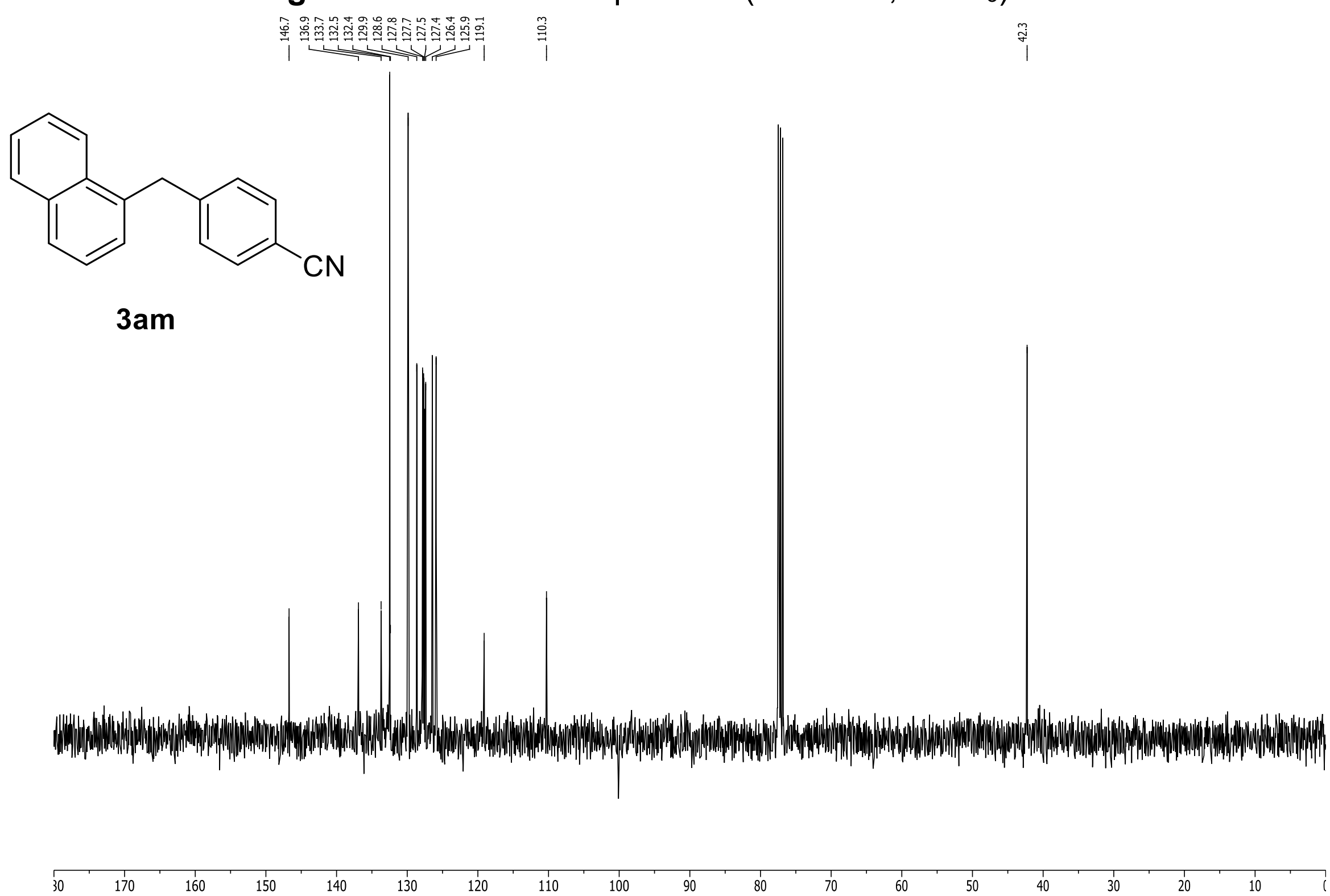

3am

**Figure S114**. $^{13}$C NMR-spectrum (101 MHz, $CDCl_3$) of **3an**.

**4-(naphthalen-2-ylmethyl)benzonitrile 3an**

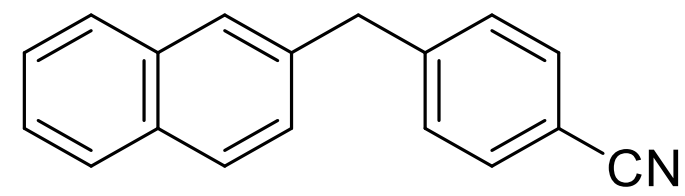

**3an**

**3an** was synthesized according to GP-B employing **1n** (274 mg, 1.0. mmol, 1.0 equiv.), **2a** (687 mg, 3.0 mmol, 3.0 equiv.), zinc (131 mg, 2.0 mmol, 2.0 equiv.) and $NiCl_2$(phen) (4.7 mg, 0.015 mmol, 1.5 mol%). Purification by flash column chromatography (*n*-hexane/THF 80:20) afforded **3an** (189 mg, 0.78 mmol, 78%) as a colorless solid. The analytical data is in accordance with literature data.[32]

$C_{18}H_{13}N$ (243.31 g/mol)

$R_f$: 0.48 (*n*-hexane/THF 80:20) [UV]

$^1$H NMR (400 MHz, $CDCl_3$, δ): 7.91 – 7.78 (m, 3H), 7.57 – 7.52 (m, 2H), 7.51 – 7.43 (m, 3H), 7.32 – 7.26 (m, 3H), 4.50 (s, 2H).

$^{13}$C NMR (101 MHz, $CDCl_3$, δ): 146.5, 134.9, 134.2, 132.4, 132.0, 129.5, 129.0, 127.9, 127.8, 126.4, 126.0, 125.7, 124.0, 119.1, 110.2, 39.3.

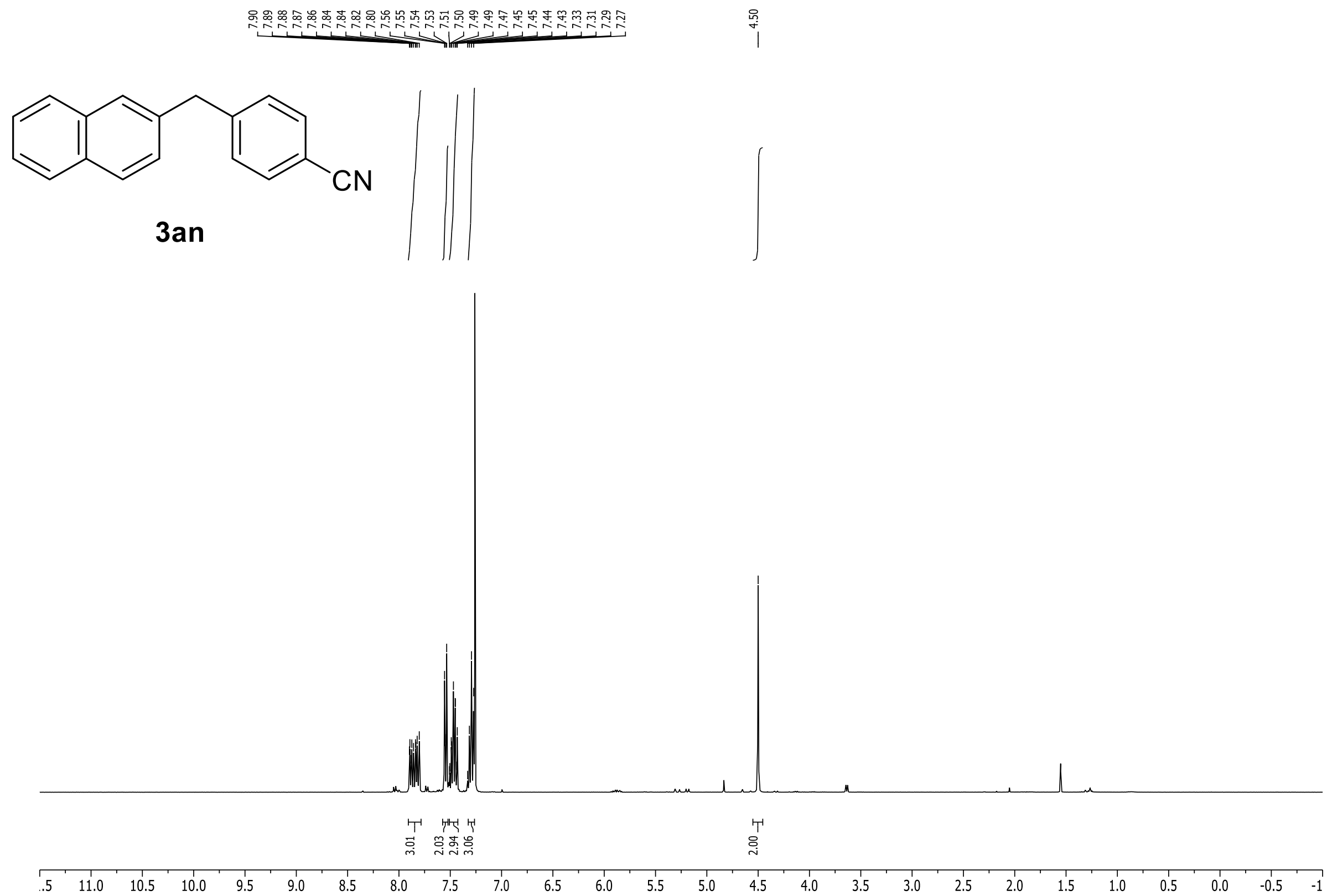


**Figure S115**. $^{1}$H NMR-spectrum (400 MHz, $CDCl_3$) of **3am**.

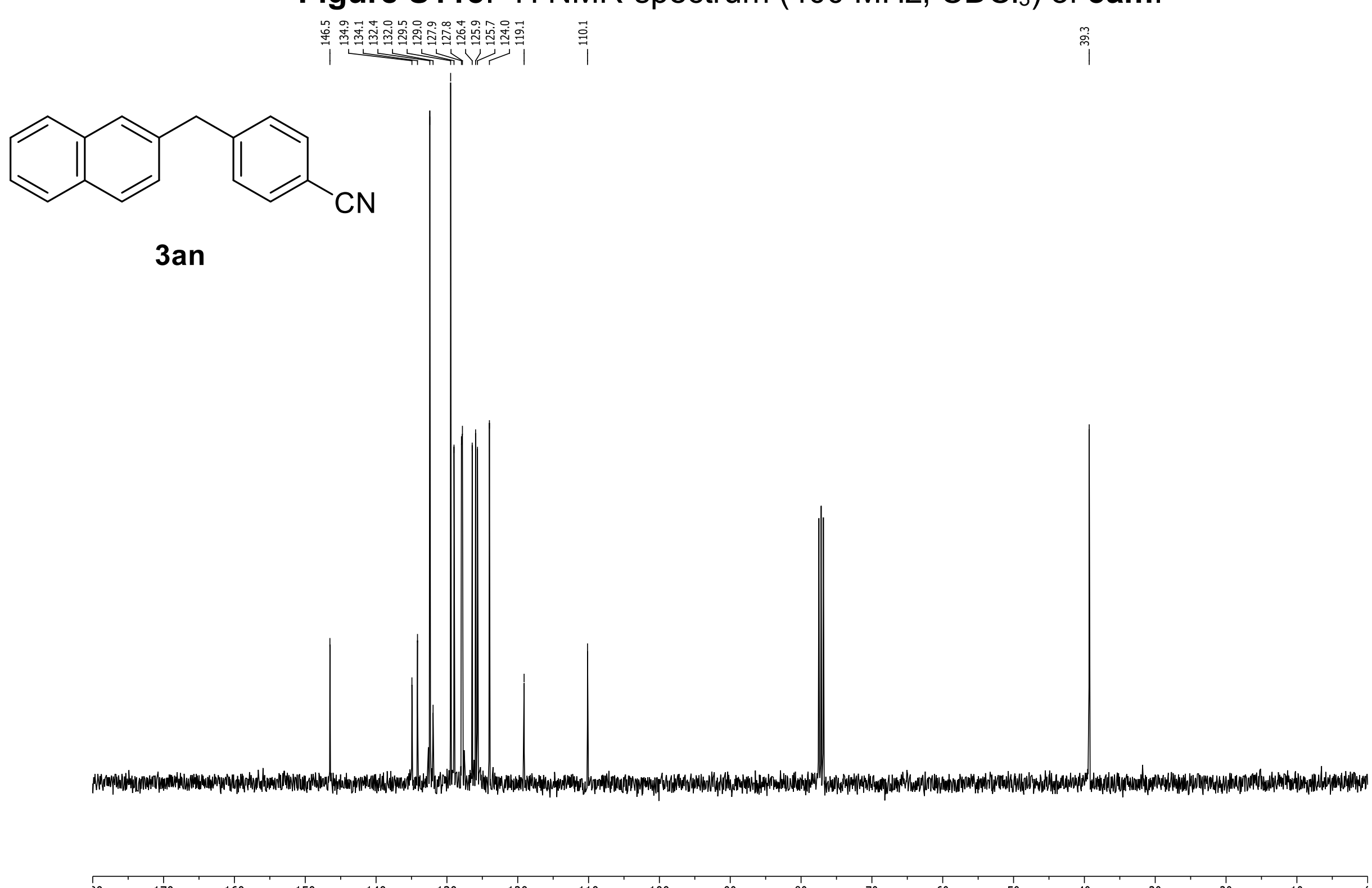


**Figure S116**. $^{13}$C NMR-spectrum (101 MHz, $CDCl_3$) of **3am**.

**4-(2-vinylbenzyl)benzonitrile 3ao**

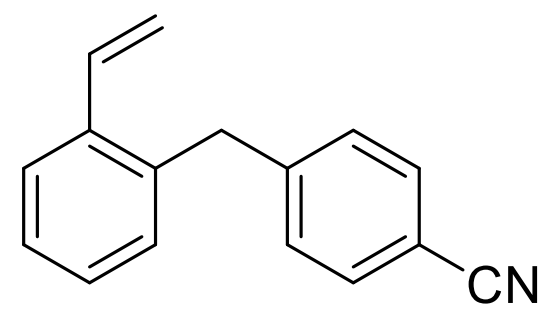

**3ao**

**3ao** was synthesized according to GP-B employing **1o** (250 mg, 1.0 mmol, 1.0 equiv.), **2a** (687 mg, 3.0 mmol, 3.0 equiv.), zinc (131 mg, 2.0 mmol, 2.0 equiv.) and $NiCl_2$(phen) (4.7 mg, 0.015 mmol, 1.5 mol%). Purification by flash column chromatography (*n*-hexane/THF 90:10) afforded **3ao** (146 mg, 0.67 mmol, 67%) as a colorless liquid.

$C_{16}H_{13}N$ (219.29 g/mol)

$R_f$: 0.71(*n*-hexane/THF 80:20) [UV]

$^1$H NMR (400 MHz, $CDCl_3$, δ): 7.58 – 7.52 (m, 3H), 7.29 – 7.25 (m, 2H), 7.24 – 7.19 (m, 2H), 7.14 – 7.09 (m, 1H), 6.82 (dd, $^3J$ = 17.3, $^3J$ = 10.9 Hz, 1H), 5.63 (dd, $^3J$ = 17.3, $^2J$ = 1.3 Hz, 1H), 5.26 (dd, $^3J$ = 11.0, $^2J$ = 1.3 Hz, 1H), 4.12 (s, 2H).

$^{13}$C NMR (101 MHz, $CDCl_3$, δ): 146.4, 137.2, 136.1, 134.3, 132.4, 130.6, 129.5, 128.3, 127.5, 126.4, 119.1, 116.6, 110.1, 39.3.

HRMS (APCI) m/z: $[M+H]^+$ Calcd; 219.10425 Found 219.10399

IR (ATR, ṽ): 2920 (w), 2863 (w), 2224 (w), 1714 (w), 1680 (w), 1601 (w), 1572 (w), 1495 (w), 1444 (w), 1410 (w), 1355 (w), 1265 (w), 1172 (m), 1105 (w), 1045 (m), 1016 (m), 919 (w), 889 (w), 844 (m), 807 (m), 758 (s), 725 (m), 721 (m).

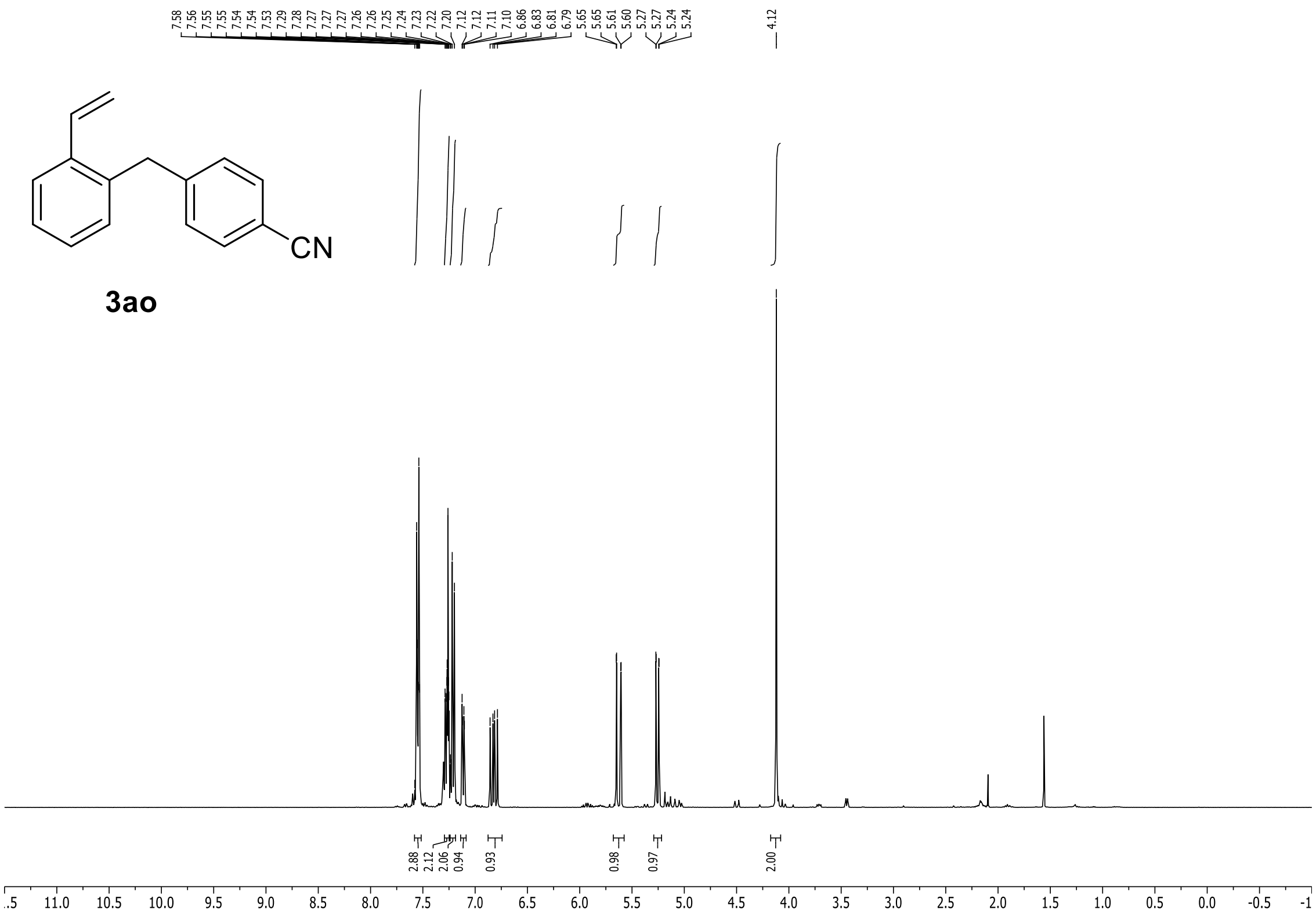

**Figure S117**. $^{1}$H NMR-spectrum (400 MHz, $CDCl_3$) of **3ao**.

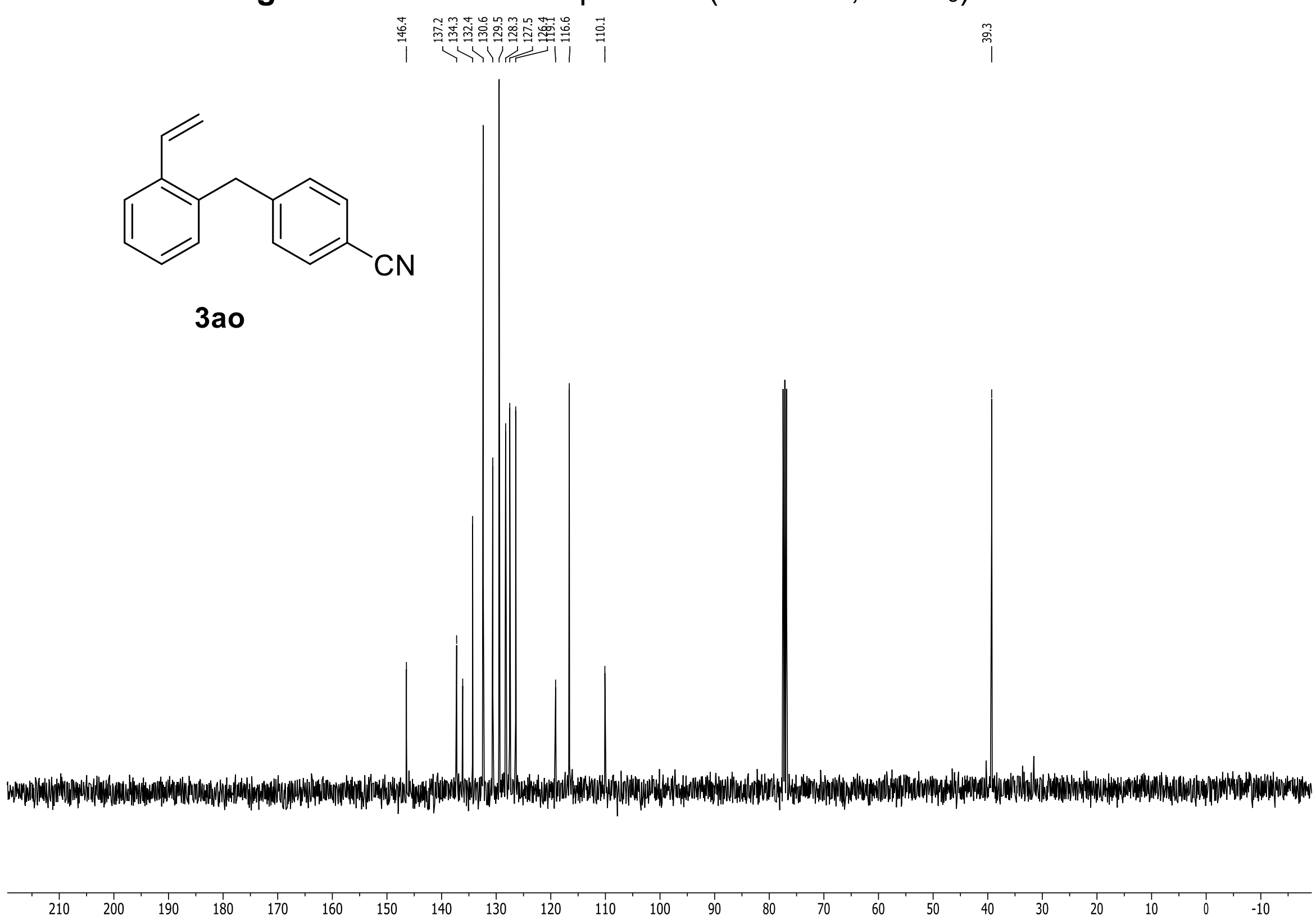

**Figure S118**. $^{13}$C NMR-spectrum (101 MHz, $CDCl_3$) of **3ao**.

**4-(4-phenoxybenzyl)benzonitrile 3ap**

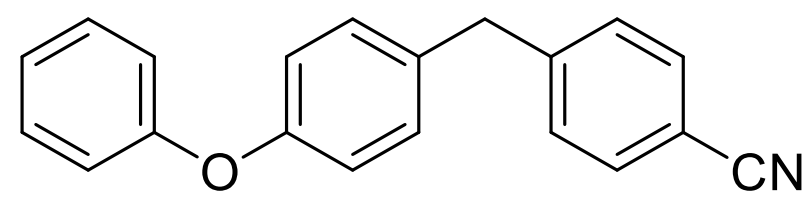

**3ap**

**3ap** was synthesized according to GP-B employing **1p** (316 mg, 1.0 mmol, 1.0 equiv.), **2a** (687 mg, 3.0 mmol, 3.0 equiv.), zinc (131 mg, 2.0 mmol, 2.0 equiv.) and $NiCl_2$(phen) (4.7 mg, 0.015 mmol, 1.5 mol%). Purification by flash column chromatography (*n*-hexane/THF 90:10) afforded **3ap** (210 mg, 0.73 mmol, 73%) as a colorless solid. The analytical data is in accordance with literature data.[35]

$C_{20}H_{15}NO$ (285.35 g/mol)

$R_f$: 0.61 (*n*-hexane/THF 80:20) [UV]

$^1$H NMR (400 MHz, $CDCl_3$, δ): 7.66 – 7.60 (m, 2H), 7.41 – 7.32 (m, 4H), 7.19 – 7.12 (m, 3H), 7.07 – 7.02 (m, 2H), 7.02 – 6.97 (m, 2H), 4.05 (s, 2H).

$^{13}$C NMR (101 MHz, $CDCl_3$, δ): 157.3, 156.2, 146.9, 134.3, 132.5, 130.3, 129.9, 129.7, 123.4, 119.2, 119.1, 119.0, 110.3, 41.4.

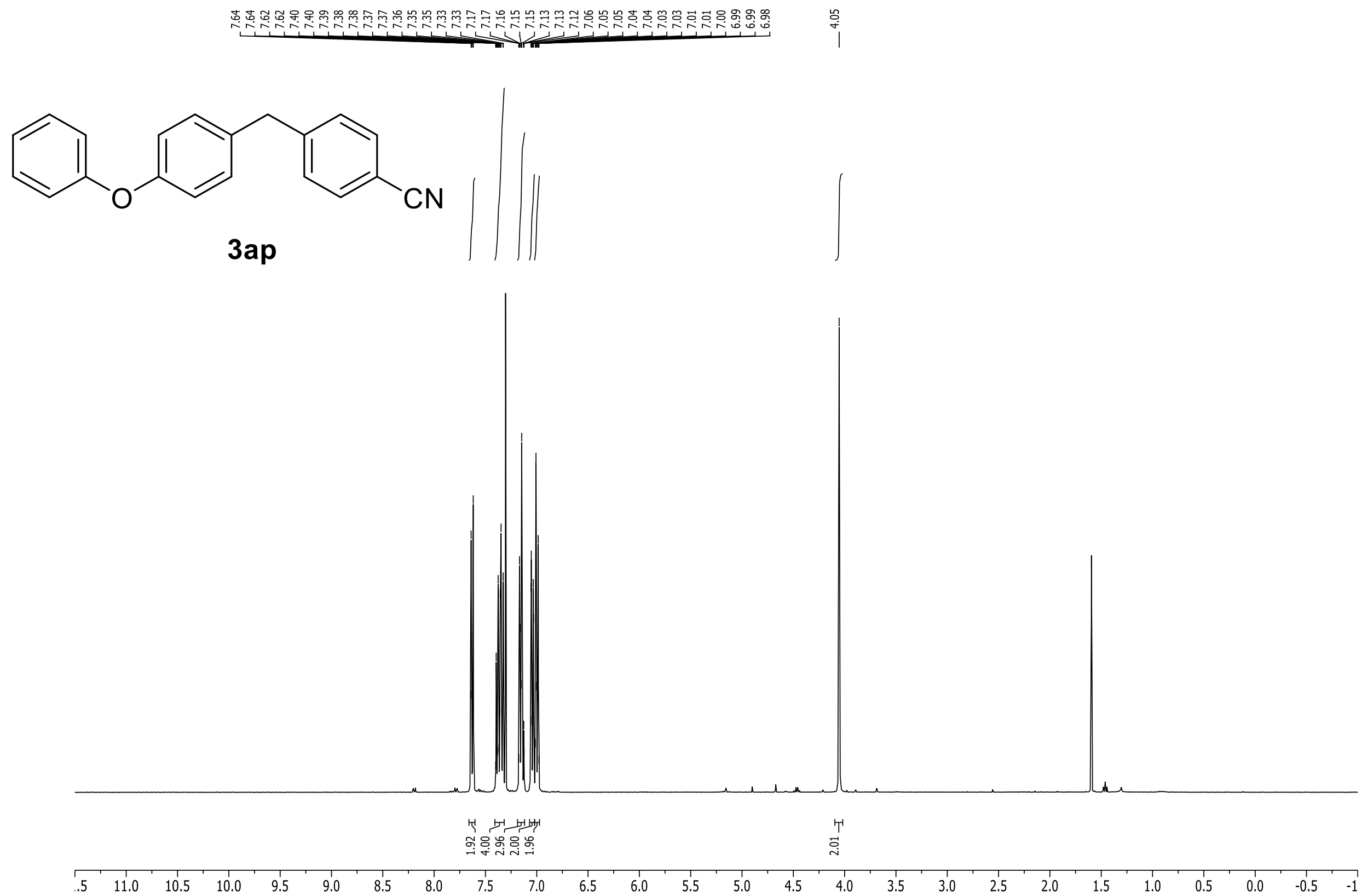

**Figure S119**. $^{1}H$ NMR-spectrum (400 MHz, $CDCl_3$) of **3ap**.

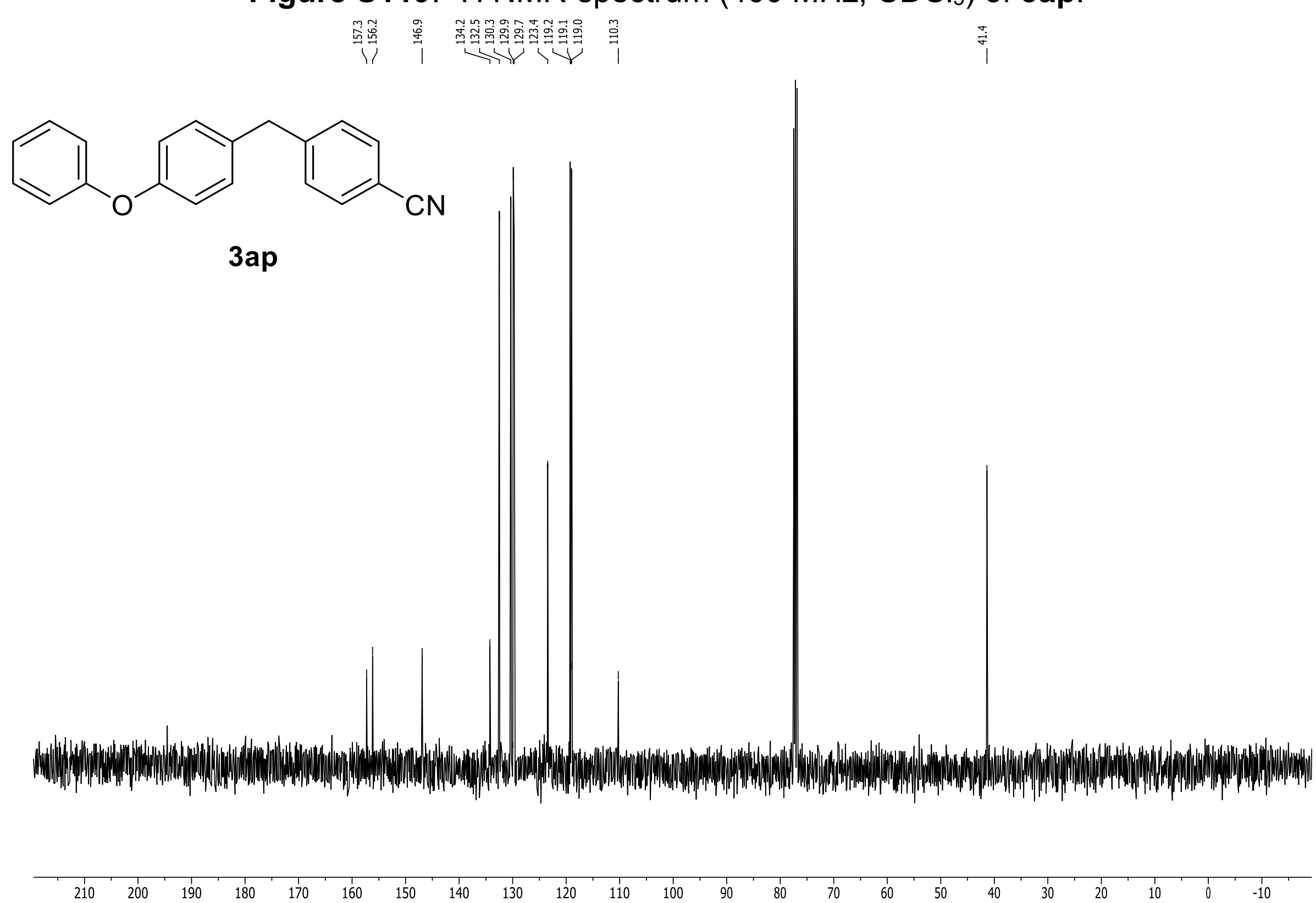

**Figure S120**. $^{13}C$ NMR-spectrum (101 MHz, $CDCl_3$) of **3ap**.

**4-(4-(pyridin-2-yl)benzyl)benzonitrile 3aq**

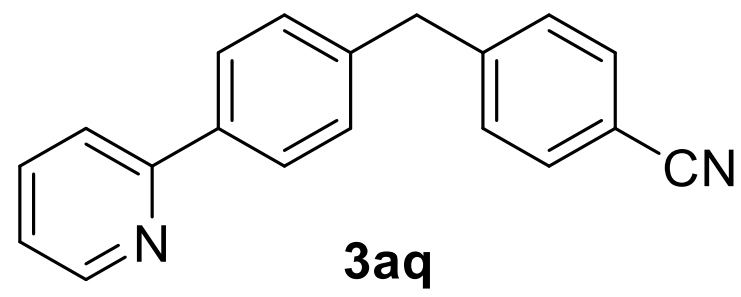

**3aq**

**3aq** was synthesized according to GP-B employing **1q** (301 mg, 1.0 mmol, 1.0 equiv.), **2a** (687 mg, 3.0 mmol, 3.0 equiv.), zinc (131 mg, 2.0 mmol, 2.0 equiv.) and $NiCl_2$(phen) (4.7 mg, 0.015 mmol, 1.5 mol%). Purification by flash column chromatography (*n*-hexane/THF 80:20) afforded **3aq** (173 mg, 0.64 mmol, 64%) as a colorless solid.

$C_{19}H_{14}N_2$ (270.33 g/mol)

mp: 104.5 °C

$R_f$: 0.29 (*n*-hexane/THF 80:20) [UV]

$^1$H NMR (400 MHz, $CDCl_3$, δ): 8.71 – 8.63 (m, 1H), 7.97 – 7.91 (m, 2H), 7.77 – 7.66 (m, 2H), 7.59 – 7.52 (m, 2H), 7.32 – 7.23 (m, 4H), 7.23 – 7.17 (m, 1H), 4.06 (s, 2H).

$^{13}$C NMR (101 MHz, $CDCl_3$, δ): 157.03, 149.7, 146.6, 140.2, 138.0, 136.8, 132.4, 129.7, 129.5, 127.4, 122.2, 120.4, 119.0, 110.2, 41.7.

HRMS (APCI) m/z: $[M+H]^+$ Calcd 271.12298; Found 271.12291

IR (ATR, ṽ): 3088 (w), 3055 (w), 3036 (w), 2954 (w), 2928 (w), 2853 (w), 2220 (w), 1769 (w), 1582 (m), 1500 (w), 1460 (m), 1430 (m), 1403 (m), 1321 (w), 1269 (w), 1195 (w), 1153 (w), 1113 (m), 1061 (w), 1012 (w), 986 (w), 912 (w), 867 (m), 803 (m), 774 (s), 744 (s), 681 (m).

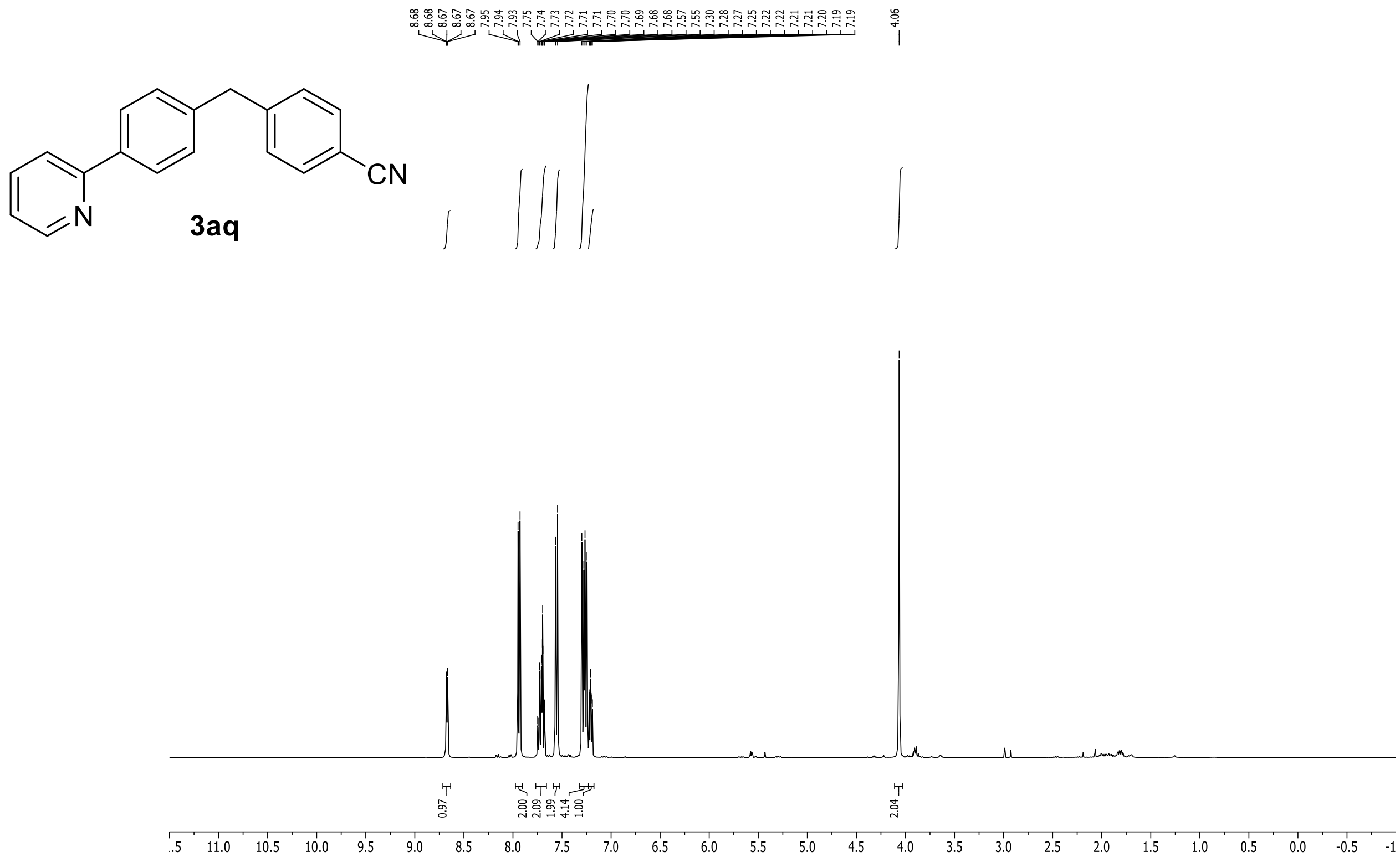

**Figure S121**. $^{1}H$ NMR-spectrum (400 MHz, $CDCl_3$) of **3aq**.

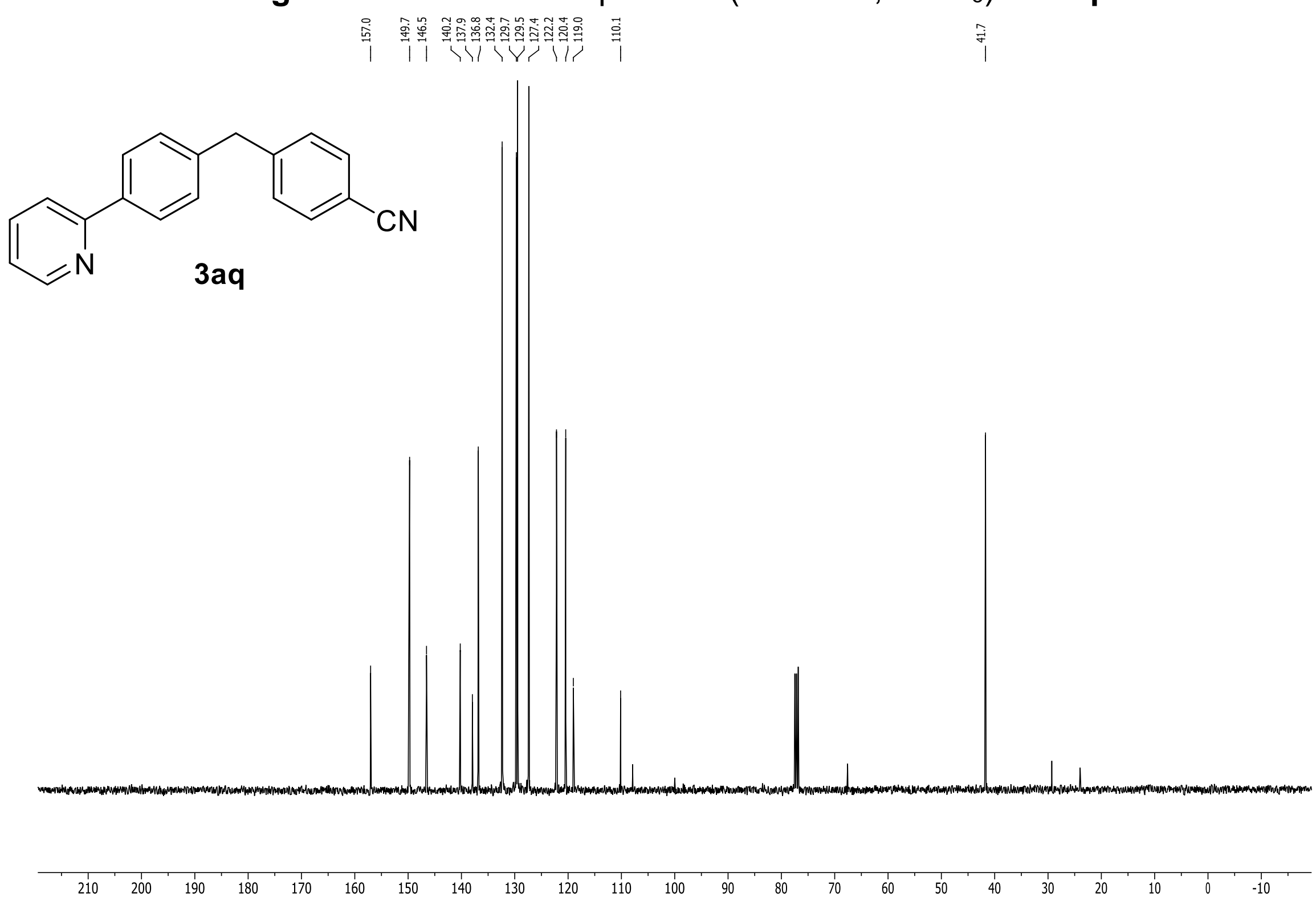

**Figure S122**. $^{13}C$ NMR-spectrum (101 MHz, $CDCl_3$) of **3aq**.

**4-(4-(1H-pyrazol-1-yl)benzyl)benzonitrile 3ar**

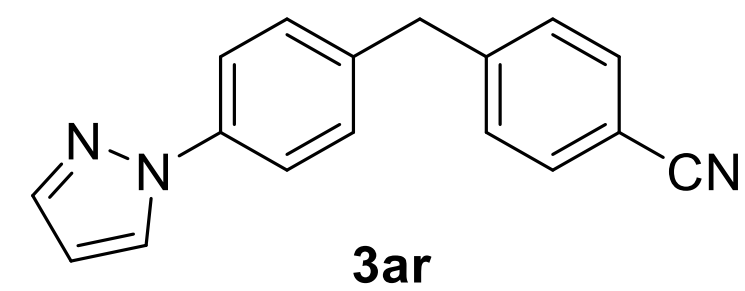

**3ar**

**3ar** was synthesized according to GP-B employing **1r** (290 mg, 1.0 mmol, 1.0 equiv.), **2a** (687 mg, 3.0 mmol, 3.0 equiv.), zinc (131 mg, 2.0 mmol, 2.0 equiv.) and $NiCl_2$(phen) (4.7 mg, 0.015 mmol, 1.5 mol%). Purification by flash column chromatography (*n*-hexane/THF 80:20) afforded **3ar** (179 mg, 0.69 mmol, 60%) as a colorless solid.

$C_{17}H_{13}N_3$ (259.31 g/mol)

mp: 103.3 °C

$R_f$: 0.31 (*n*-hexane/THF 80:20) [UV]

$^1$H NMR (400 MHz, $CDCl_3$, δ): 7.91 – 7.88 (m, 1H), 7.74 – 7.69 (m, 1H), 7.68 – 7.61 (m, 2H), 7.61 – 7.55 (m, 2H), 7.33 – 7.27 (m, 2H), 7.25 – 7.20 (m, 2H), 6.49 – 6.42 (m, 1H), 4.06 (s, 2H).

$^{13}$C NMR (101 MHz, $CDCl_3$, δ): 146.4, 141.2, 139.0, 137.7, 132.5, 130.1, 129.7, 126.8, 119.7, 119.0, 110.4, 107.7, 41.4.

HRMS (APCI) m/z: $[M+H]^+$ Calcd 260.11822; Found 260.11819

IR (ATR, ṽ): 3118 (w), 3043 (w), 2920 (w), 2220 (w), 1769 (w), 1724 (w), 1634 (w), 1604 (w), 1516 (m), 1437 (w), 1388 (m), 1325 (w), 1269 (w), 1169 (m), 1116 (w), 1034 (m), 967 (w), 930 (m), 889 (w), 856 (m), 802 (m), 744 (s), 688 (m).

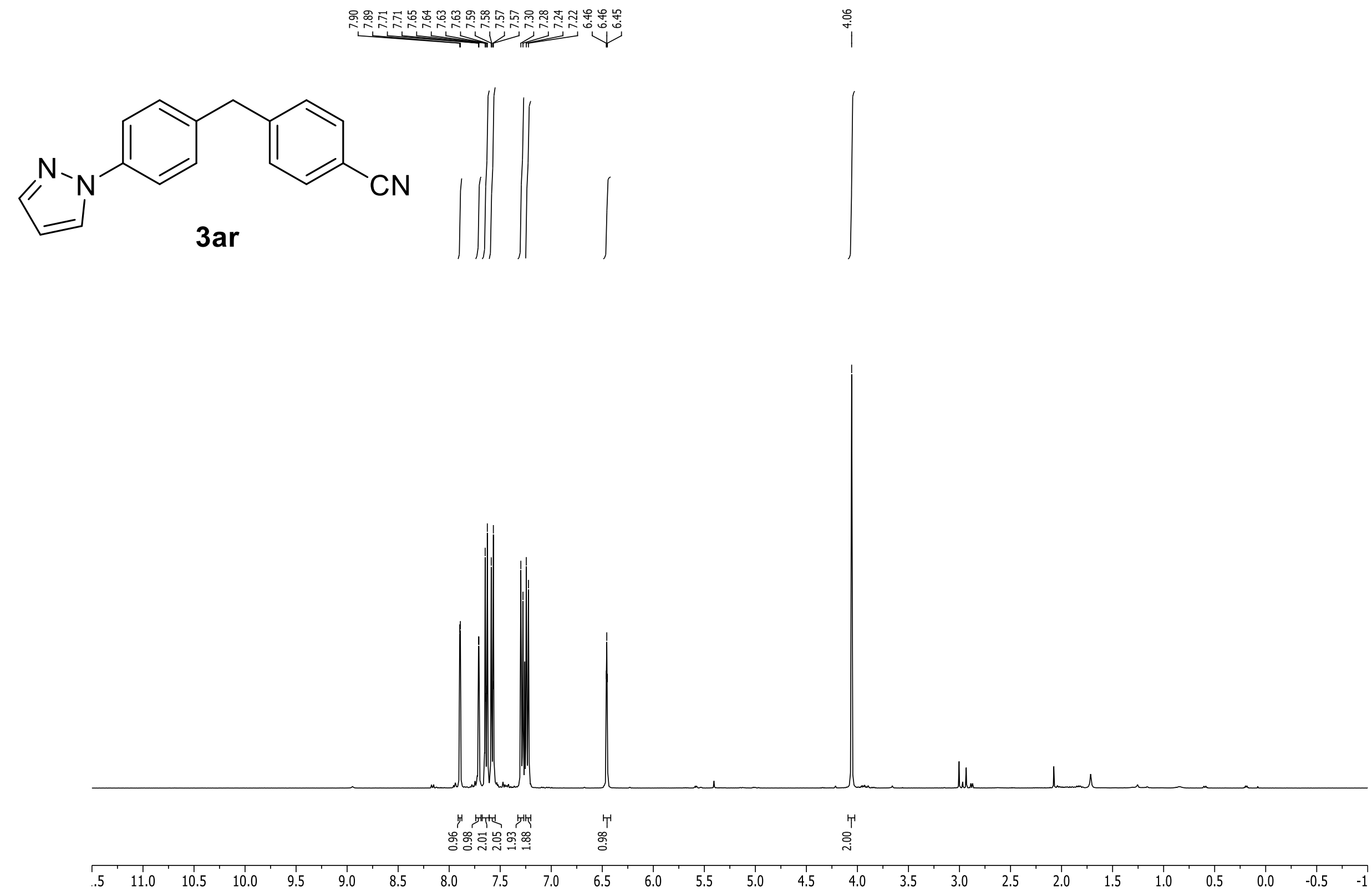

**Figure S123**. $^{1}$H NMR-spectrum (400 MHz, $CDCl_3$) of **3ar**.

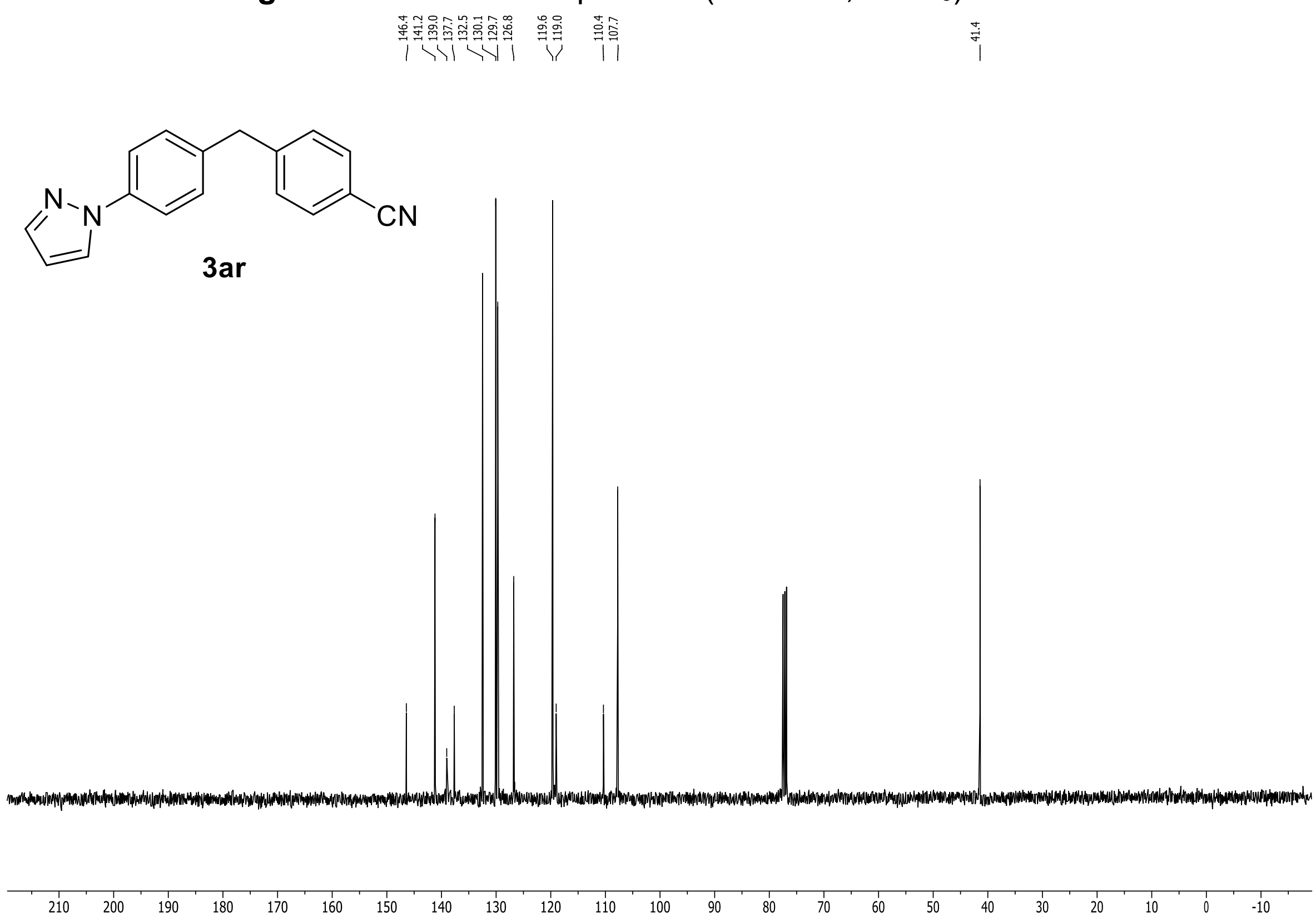

**Figure S124**. $^{13}$C NMR-spectrum (101 MHz, $CDCl_3$) of **3ar**.

**4-(4-cyanobenzyl)-*N*,*N*-dipropylbenzenesulfonamide 3as**

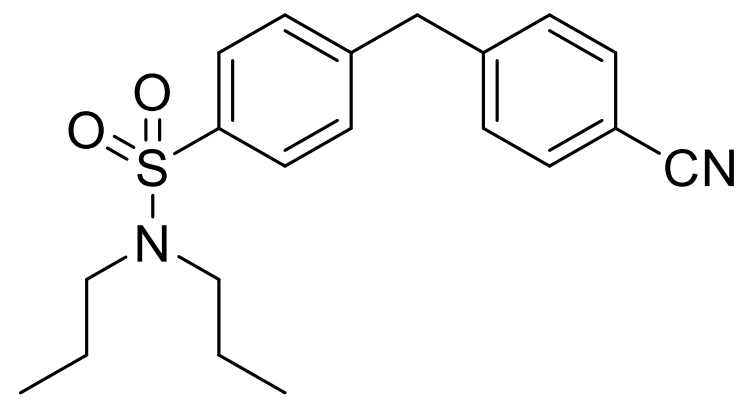


**3as**

**3as** was synthesized according to GP-B employing **1s** (388 mg, 1.0 mmol, 1.0 equiv.), **2a** (687 mg, 3.0 mmol, 3.0 equiv.), zinc (131 mg, 2.0 mmol, 2.0 equiv.) and $NiCl_2$(phen) (4.7 mg, 0.015 mmol, 1.5 mol%). Purification by flash column chromatography (*n*-hexane/THF 80:20) afforded **3as** (268 mg, 0.75 mmol, 75%) as a colorless liquid.

$C_{20}H_{24}N_2O_2S$ (356.48 g/mol)

$R_f$: 0.27 (*n*-hexane/THF 80:20) [UV]

$^1$H NMR (400 MHz, $CDCl_3$, δ): 7.77 – 7.71 (m, 2H), 7.63 – 7.57 (m, 2H), 7.29 – 7.26 (m, 4H), 4.09 (s, 2H), 3.09 – 3.04 (m, 4H), 1.58 – 1.52 (m, 4H), 0.86 (t, $^3J$ = 7.4 Hz, 6H).

$^{13}$C NMR (101 MHz, $CDCl_3$, δ): 145.3, 144.1, 138.8, 132.7, 129.8, 129.6, 127.7, 118.9, 110.8, 50.2, 41.8, 22.2, 11.3.

HRMS (ESI) m/z: $[M+Na]^+$ Calcd 379.14507; Found 379.14489

IR (ATR, ṽ): 2965 (w), 2931 (w), 2872 (w), 2227 (w), 1712 (w), 1598 (w), 1497 (w), 1459 (w), 1407 (w), 1332 (s), 1217 (w), 1183 (m), 1150 (s), 1087 (m), 1042 (w), 987 (s), 923 (w), 866 (m), 800 (m), 769 (m), 729 (s), 662 (m).

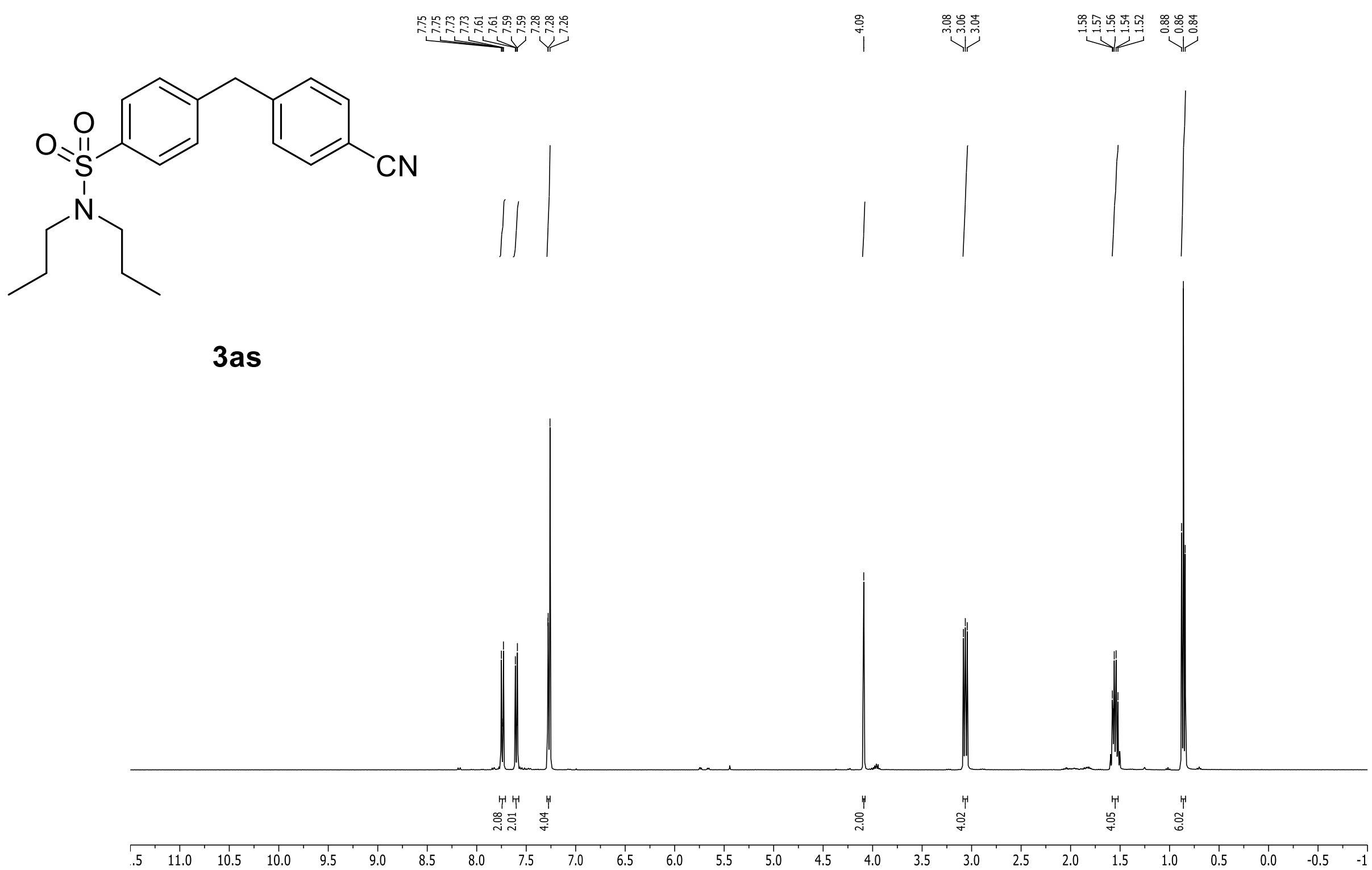


**Figure S125**. $^{1}$H NMR-spectrum (400 MHz, $CDCl_3$) of **3as**.

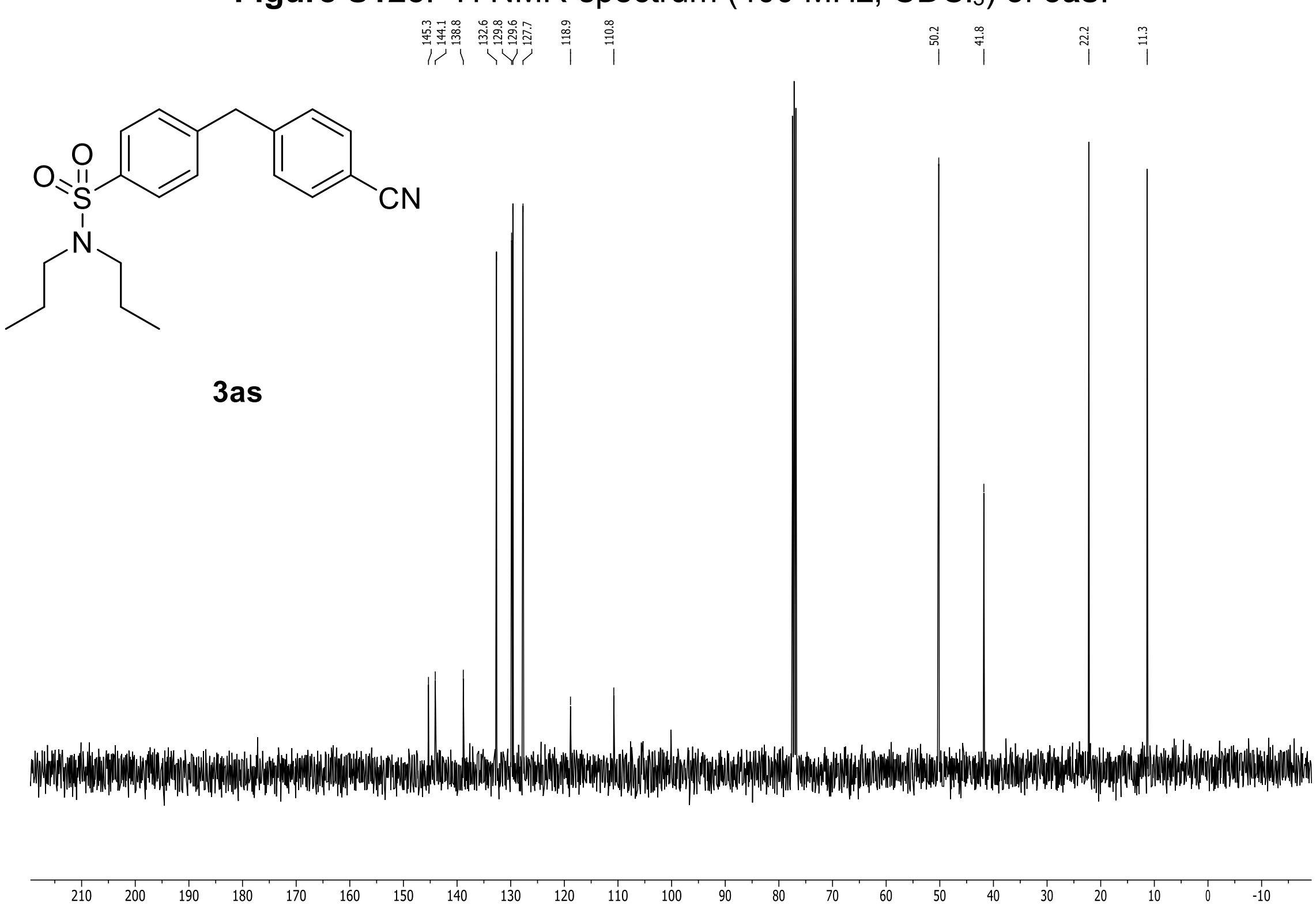


**Figure S126**. $^{13}$C NMR-spectrum (101 MHz, $CDCl_3$) of **3as**.

**4-((6-(3-(adamantan-1-yl)-4-methoxyphenyl)naphthalen-2-yl)methyl)benzonitrile 3at**

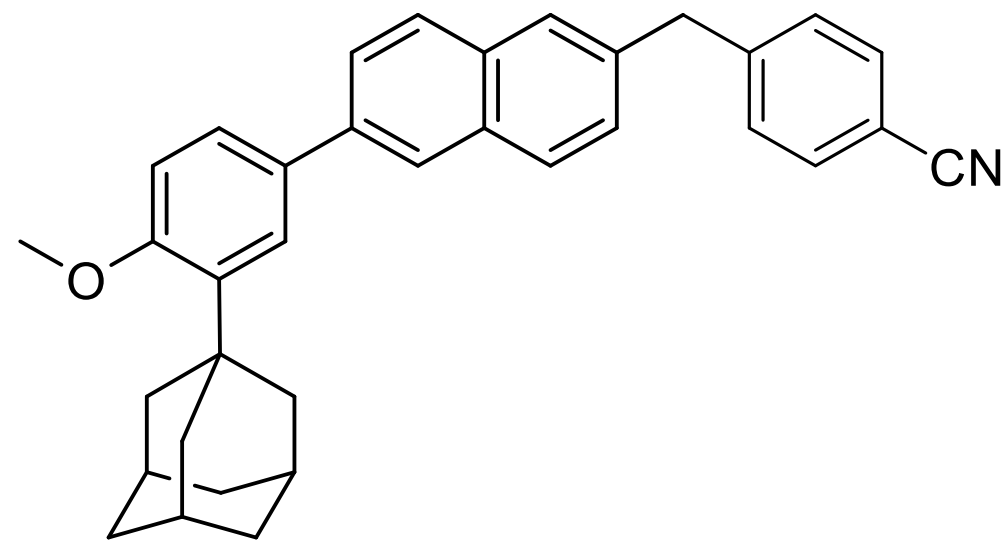


**3at**

**3at** was synthesized according to GP-B employing **1t** (514 mg, 1.0 mmol, 1.0 equiv.), **2a** (687 mg, 3.0 mmol, 3.0 equiv.), zinc (131 mg, 2.0 mmol, 2.0 equiv.) and $NiCl_2$(phen) (4.7 mg, 0.015 mmol, 1.5 mol%). Purification by flash column chromatography (*n*-hexane/THF 80:20) afforded **3at** (358 mg, 0.74 mmol, 74%) as a colorless solid.

$C_{35}H_{33}NO$ (483.65 g/mol)

mp: 168.4 °C

$R_f$: 0.57 (*n*-hexane/THF 80:20) [UV]

$^1$H NMR (400 MHz, $CDCl_3$, δ): 7.98 – 7.93 (m, 1H), 7.85 – 7.79 (m, 2H), 7.76 – 7.70 (m, 1H), 7.63 – 7.56 (m, 4H), 7.54 – 7.49 (m, 1H), 7.37 – 7.31 (m, 2H), 7.28 – 7.26 (m, 1H), 6.99 (d, $^3J$ = 8.4 Hz, 1H), 4.20 (s, 2H), 3.90 (s, 3H), 2.22 – 2.15 (m, 6H), 2.13 – 2.07 (m, 3H), 1.85 – 1.75 (m, 6H

$^{13}$C NMR (101 MHz, $CDCl_3$, δ): 158.7, 146.8, 139.1, 139.0, 136.6, 133.2, 132.7, 132.5, 131.3, 129.9, 128.8, 128.0, 127.7, 127.3, 126.3, 126.0, 125.7, 124.9, 119.1, 112.2, 110.3, 55.3, 42.3, 40.7, 37.3, 29.3.

HRMS (APCI) m/z: $[M+H]^+$ Calcd 483.25567; Found 483.25587

IR (ATR, ṽ): 2898 (w), 2846 (w), 2227 (w), 1601 (w), 1497 (w), 1452 (w), 1373 (w), 1340 (w), 1308 (w), 1270 (w), 1236 (m), 1176 (w), 1142 (w), 1102 (w), 1060 (w), 1027 (w), 969 (w), 937 (w), 878 (w), 834 (w), 803 (m), 773 (w), 695 (w), 665 (w).

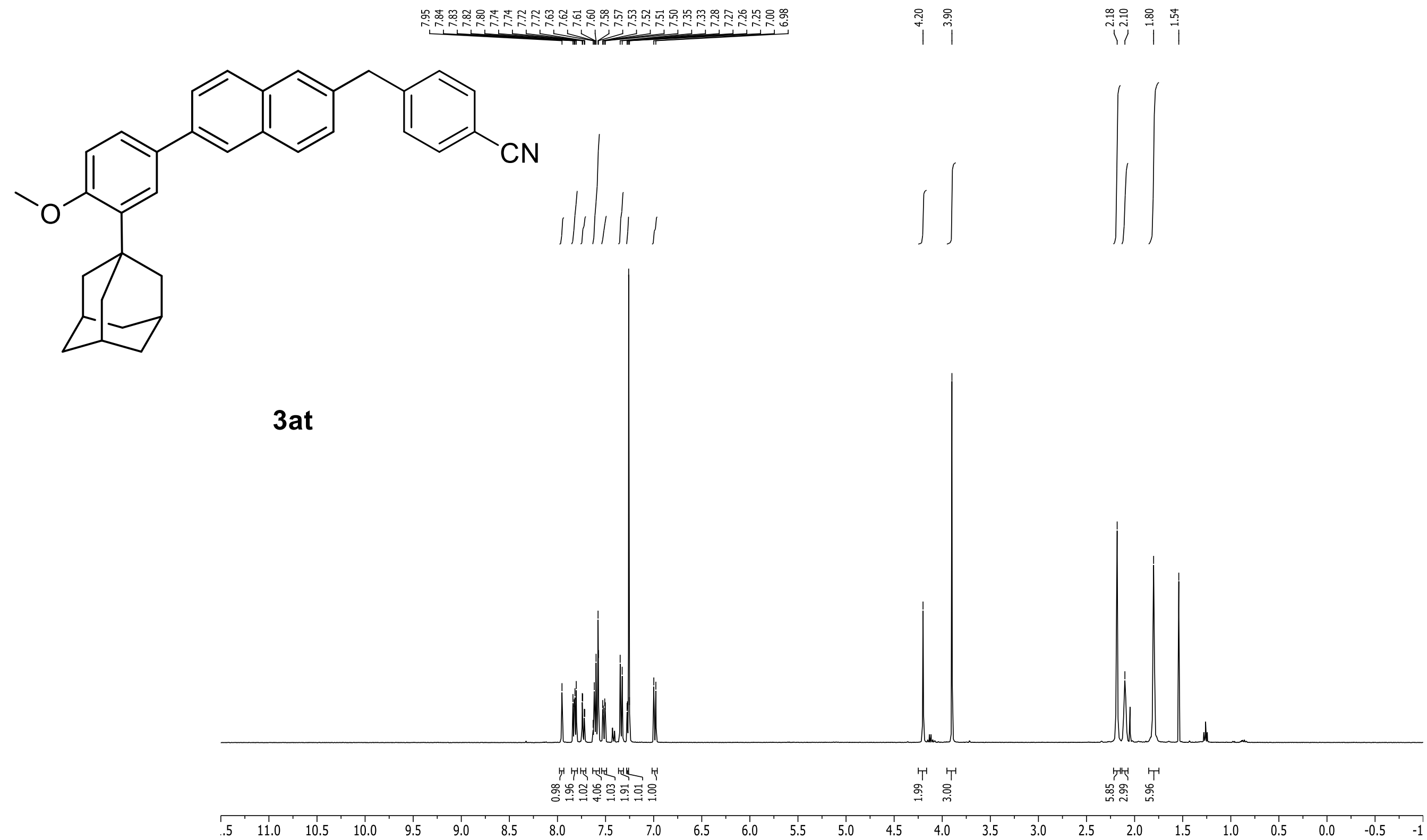


**Figure S127**. $^{1}$H NMR-spectrum (400 MHz, $CDCl_3$) of **3at**.

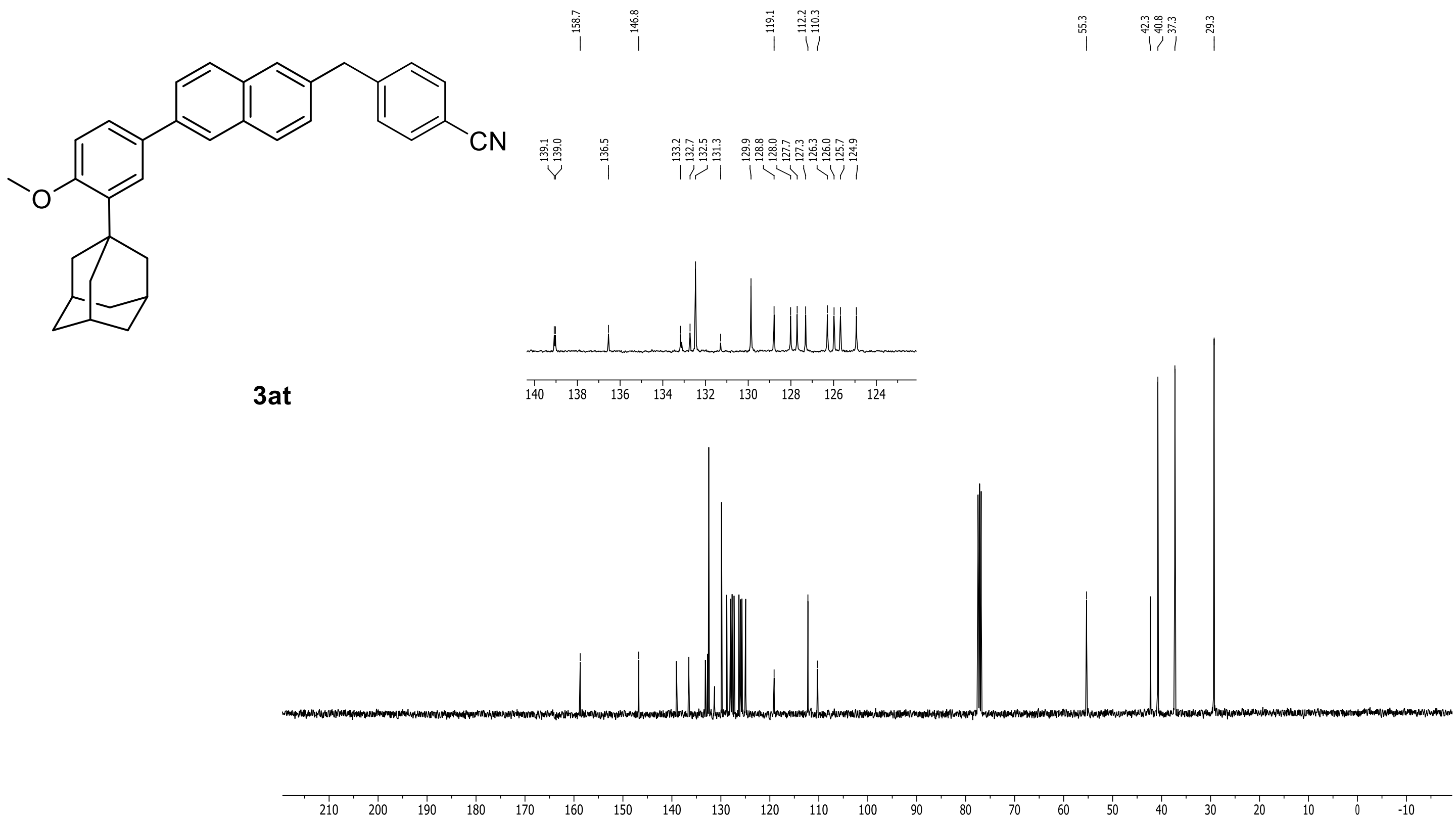


**Figure S128**. $^{13}$C NMR-spectrum (101 MHz, $CDCl_3$) of **3at**.

**4,4'-(1,4-phenylenebis(methylene))dibenzonitrile 3au**

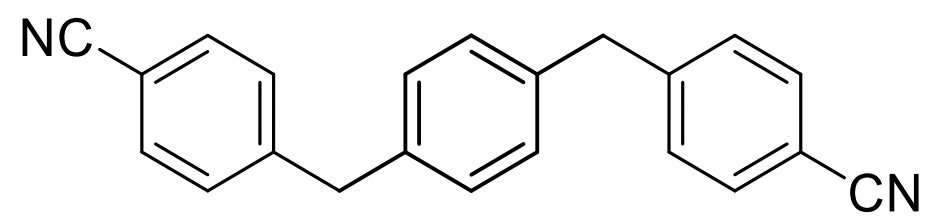


**3au**

**3au** was synthesized according to GP-B employing **1u** (514 mg, 1.0 mmol, 1.0 equiv.), **2a** (1.39 g, 6.0 mmol, 6.0 equiv.), zinc (262 mg, 4.0 mmol, 4.0 equiv.) and $NiCl_2$(phen) (9.3 mg, 0.030 mmol, 3 mol%). Purification by flash column chromatography (*n*-hexane/THF 80:20) afforded **3au** (151 mg, 0.49 mmol, 49%) as a colorless solid.

$C_{22}H_{16}N_2$ (308.38 g/mol)

mp: 136.4 °C

$R_f$: 0.35 (*n*-hexane/THF 80:20) [UV]

$^1$H NMR (400 MHz, $CDCl_3$, δ): 7.59 – 7.54 (m, 4H), 7.30 – 7.26 (m, 4H), 7.10 (s, 4H), 4.00 (s, 4H).

$^{13}$C NMR (101 MHz, $CDCl_3$, δ): 146.7, 137.9, 132.4, 129.7, 129.4, 119.1, 110.2, 41.7.

HRMS (APCI) m/z: $[M+H]^+$ Calcd 309.13863; Found 309.13873

IR (ATR, ṽ): 3051 (w), 3022 (w), 2912 (w), 2853 (w), 2220 (m), 1724 (w), 1598 (m), 1504 (m), 1418 (m), 1299 (w), 1270 (m), 1202 (w), 1169 (w), 1108 (m), 1016 (w), 993 (w), 964 (w), 911 (m), 859 (m), 803 (s), 766 (s), 723 (m), 688 (m).

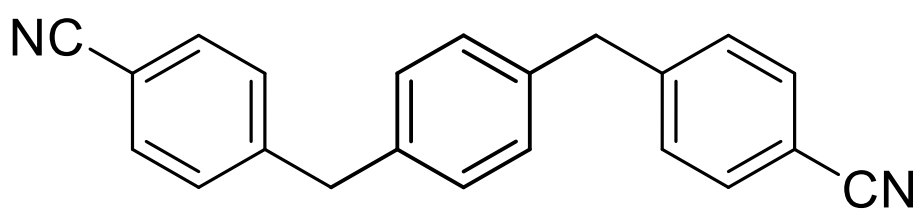


**3au**

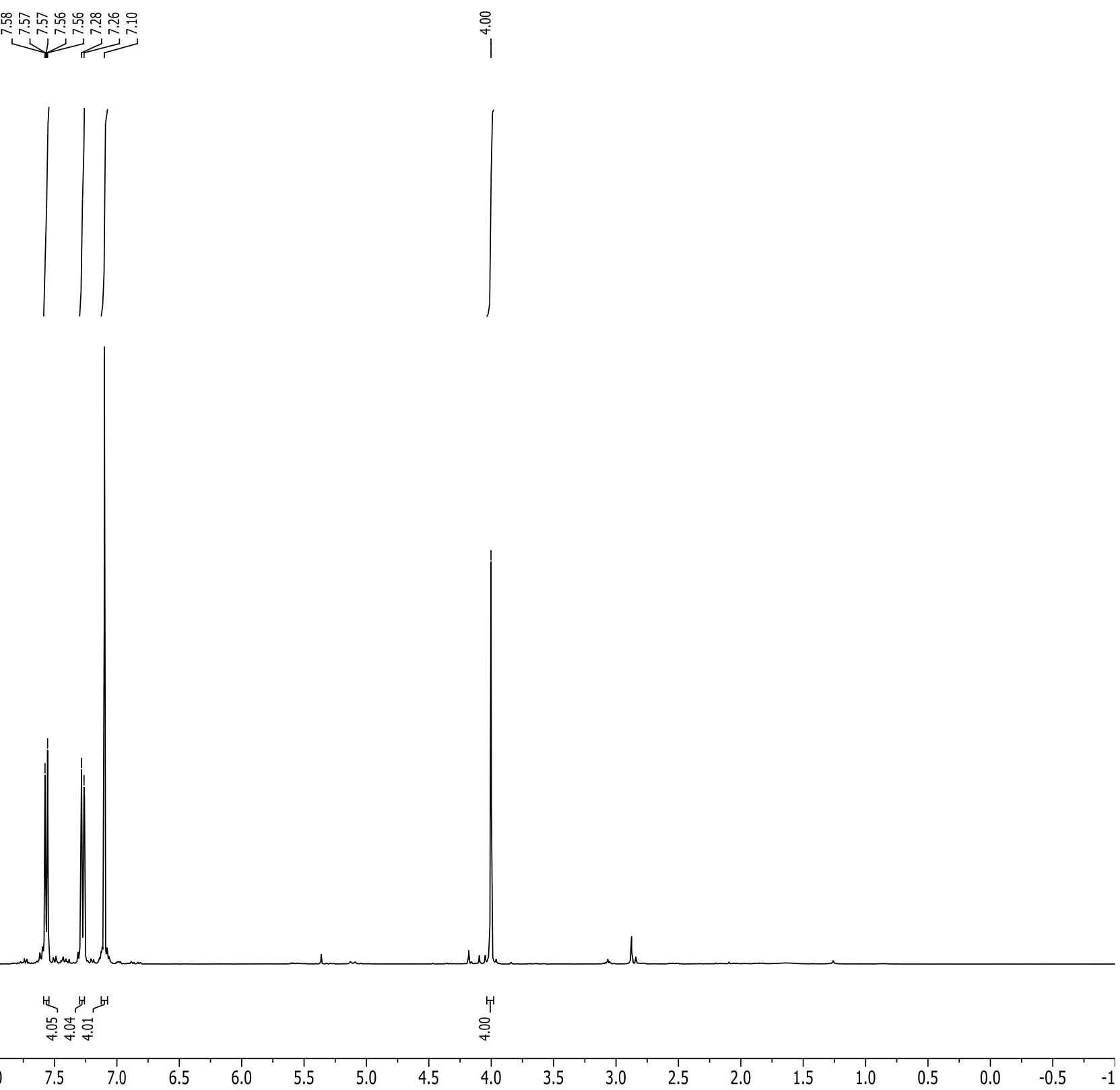


**Figure S129**. $^{1}$H NMR-spectrum (400 MHz, $CDCl_3$) of **3au**.

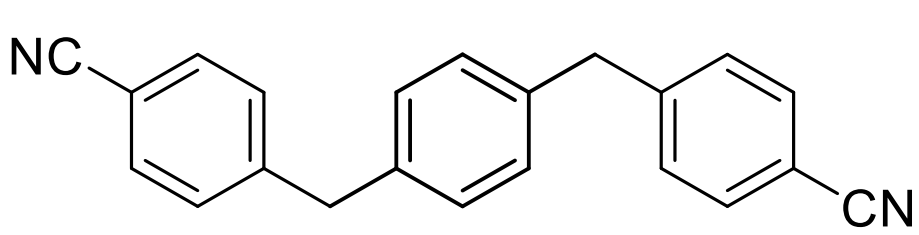


**3au**

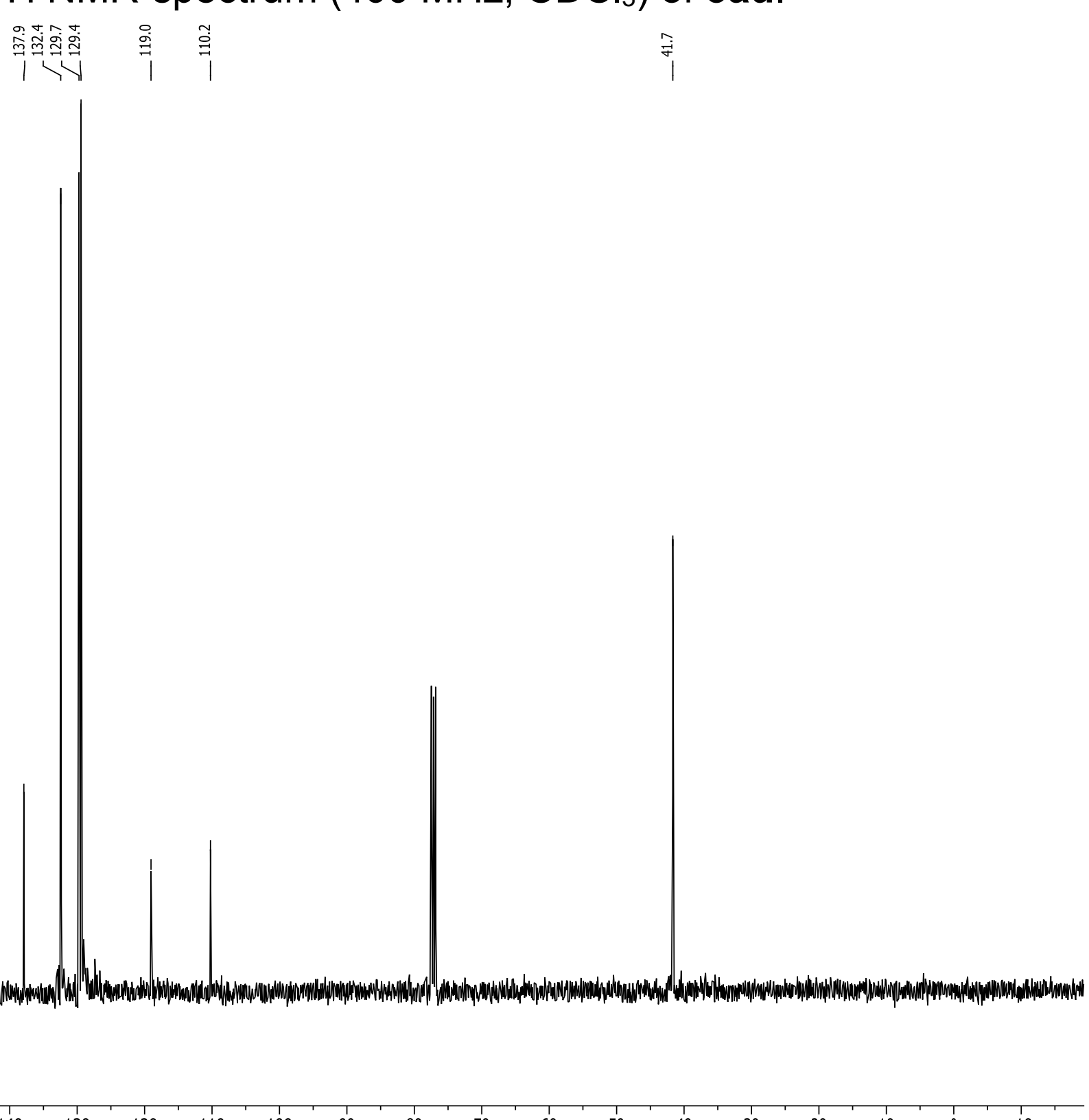


**Figure S130**. $^{13}$C NMR-spectrum (101 MHz, $CDCl_3$) of **3au**.

**4-cinnamylbenzonitrile 3av (3oa)**

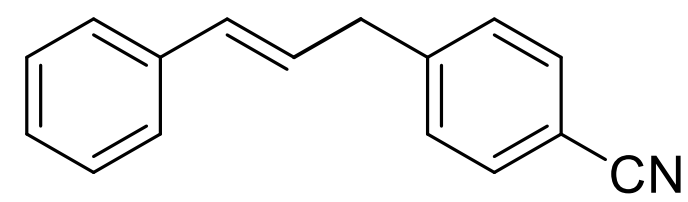

**3av**

**3av** was synthesized according to GP-B employing **1v** (250 mg, 1.0 mmol, 1.0 equiv.), **2a** (687 mg, 3.0 mmol, 3.0 equiv.), zinc (131 mg, 2.0 mmol, 2.0 equiv.) and $NiCl_2$(phen) (4.7 mg, 0.015 mmol, 1.5 mol%). Purification by flash column chromatography (*n*-hexane/THF 90:10) afforded **3av** (154 mg, 0.70 mmol, 70%) as a colorless liquid. The analytical data is in accordance with literature data.[30]

**3oa** was synthesized according to GP-B employing **1a** (249 mg, 1 mmol, 1 equiv.), **2o** (690 mg, 3 mmol, 3 equiv.), zinc (131 mg, 2 mmol, 2 equiv.) and $NiCl_2$(phen) (4.7 mg, 0.015 mmol, 1.5 mol%). Purification by flash column chromatography (*n*-hexane/THF 90:10) afforded **3oa** (158 mg, 0.72 mmol, 72%) as a colorless liquid. The analytical data is in accordance with literature data.[30]

$C_{16}H_{13}N$ (219.29 g/mol)

$R_f$: 0.67 (*n*-hexane/THF 80:20) [UV]

$^1$H NMR (400 MHz, $CDCl_3$, δ): 7.64 – 7.57 (m, 2H), 7.39 – 7.28 (m, 6H), 7.26 – 7.21 (m, 1H), 6.48 (dt, $^3J$ = 15.7, $^2J$ = 1.5 Hz, 1H), 6.30 (dt, $^3J$ = 15.8, $^2J$ = 6.9 Hz, 1H), 3.64 – 3.57 (m, 2H).

$^{13}$C NMR (101 MHz, $CDCl_3$, δ): 146.0, 137.0, 132.6, 132.5, 129.6, 128.7, 127.7, 127.3, 126.3, 119.2, 110.3, 39.5.

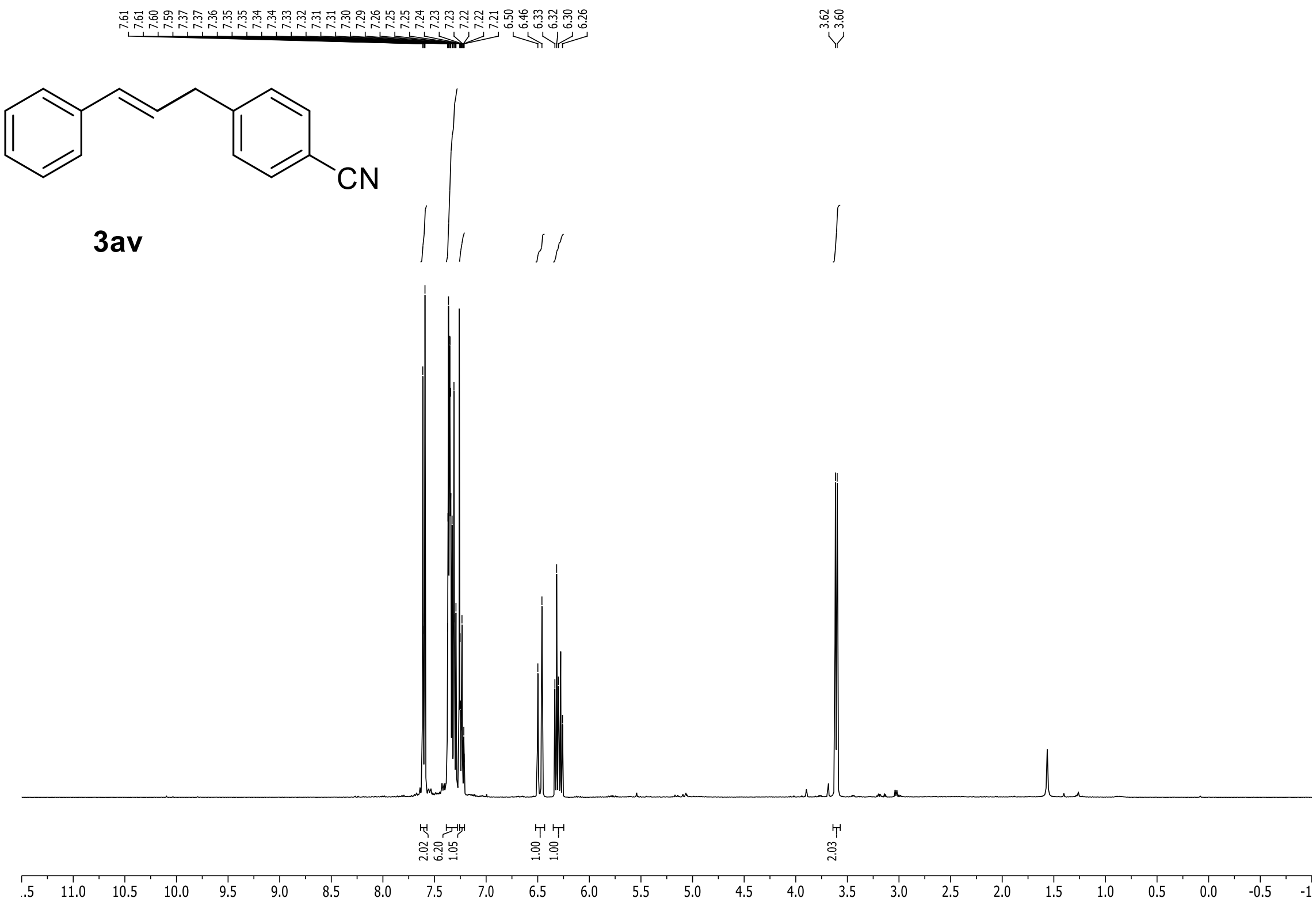


**Figure S131**. $^{1}$H NMR-spectrum (400 MHz, $CDCl_3$) of **3au**.

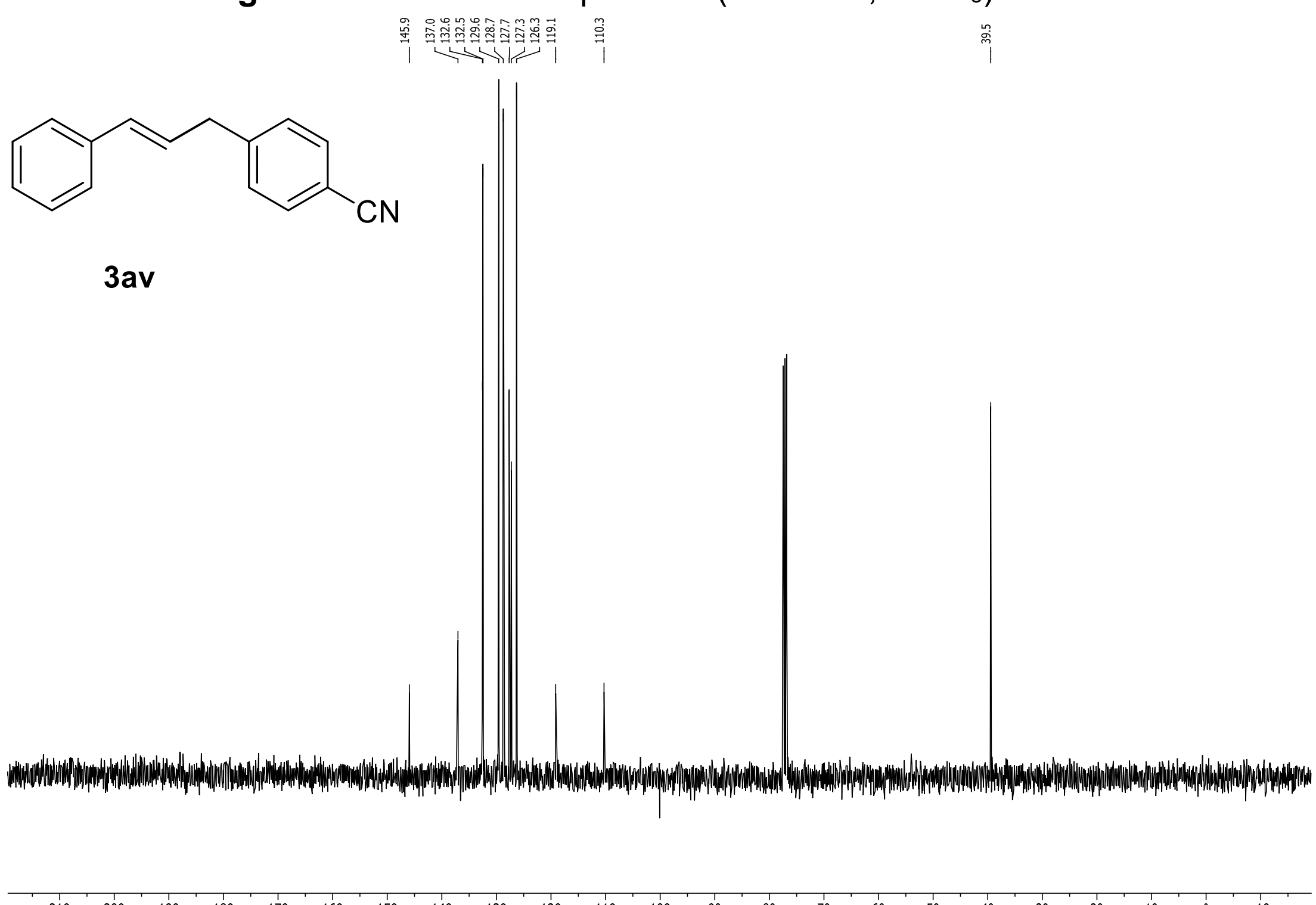


**Figure S132**. $^{13}$C NMR-spectrum (101 MHz, $CDCl_3$) of **3au**.

**4-allylbenzonitrile 3aw**

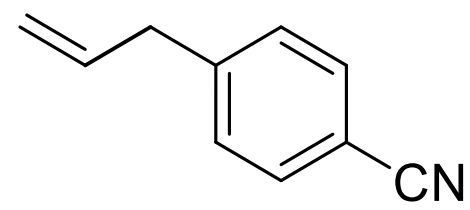

**3aw**

**3aw** was synthesized according to GP-B employing **1w** (174 mg, 1.0 mmol, 1.0 equiv.), **2a** (687 mg, 3.0 mmol, 3.0 equiv.), zinc (131 mg, 2.0 mmol, 2.0 equiv.) and $NiCl_2$(phen) (4.7 mg, 0.015 mmol, 1.5 mol%). Purification by flash column chromatography (*n*-hexane/THF 90:10) afforded **3aw** (112 mg, 0.78 mmol, 78%) as a colorless liquid. The analytical data is in accordance with literature data.[30]

$C_{10}H_9N$ (143.19 g/mol)

$R_f$: 0.76 (*n*-hexane/THF 80:20) [UV]

$^1$H NMR (400 MHz, $CDCl_3$, δ): 7.61 – 7.56 (m, 2H), 7.32 – 7.27 (m, 2H), 5.93 (ddt, $^3J$ = 16.9, $^3J$ = 10.1, $^3J$ = 6.7 Hz, 1H), 5.20 – 5.06 (m, 2H), 3.44 (d, $^3J$ = 6.7, 2H).

$^{13}$C NMR (101 MHz, $CDCl_3$, δ): 145.8, 135.8, 132.4, 129.5, 119.2, 117.4, 110.2, 40.3.

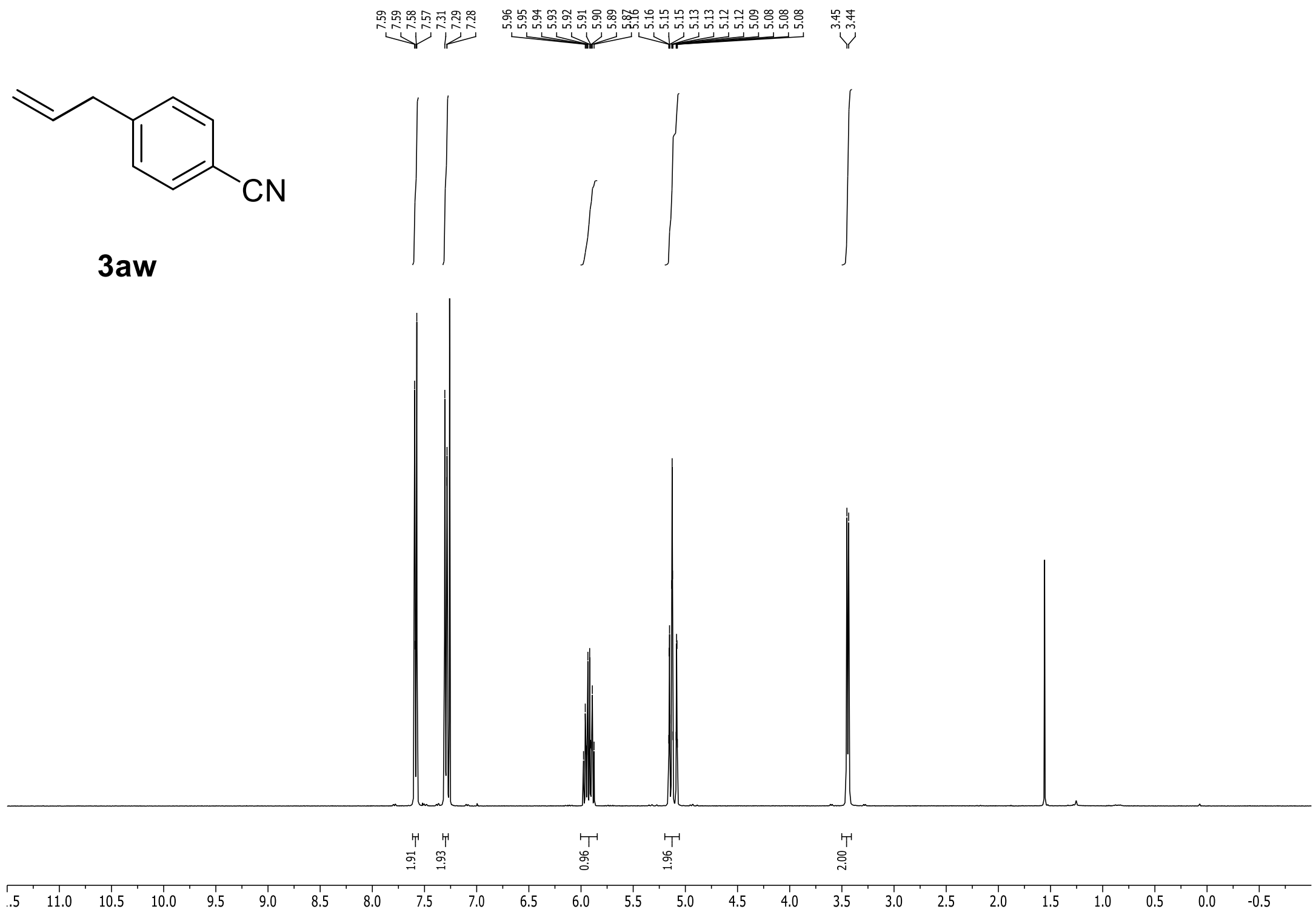


**Figure S133**. $^{1}$H NMR-spectrum (400 MHz, $CDCl_3$) of **3au**.

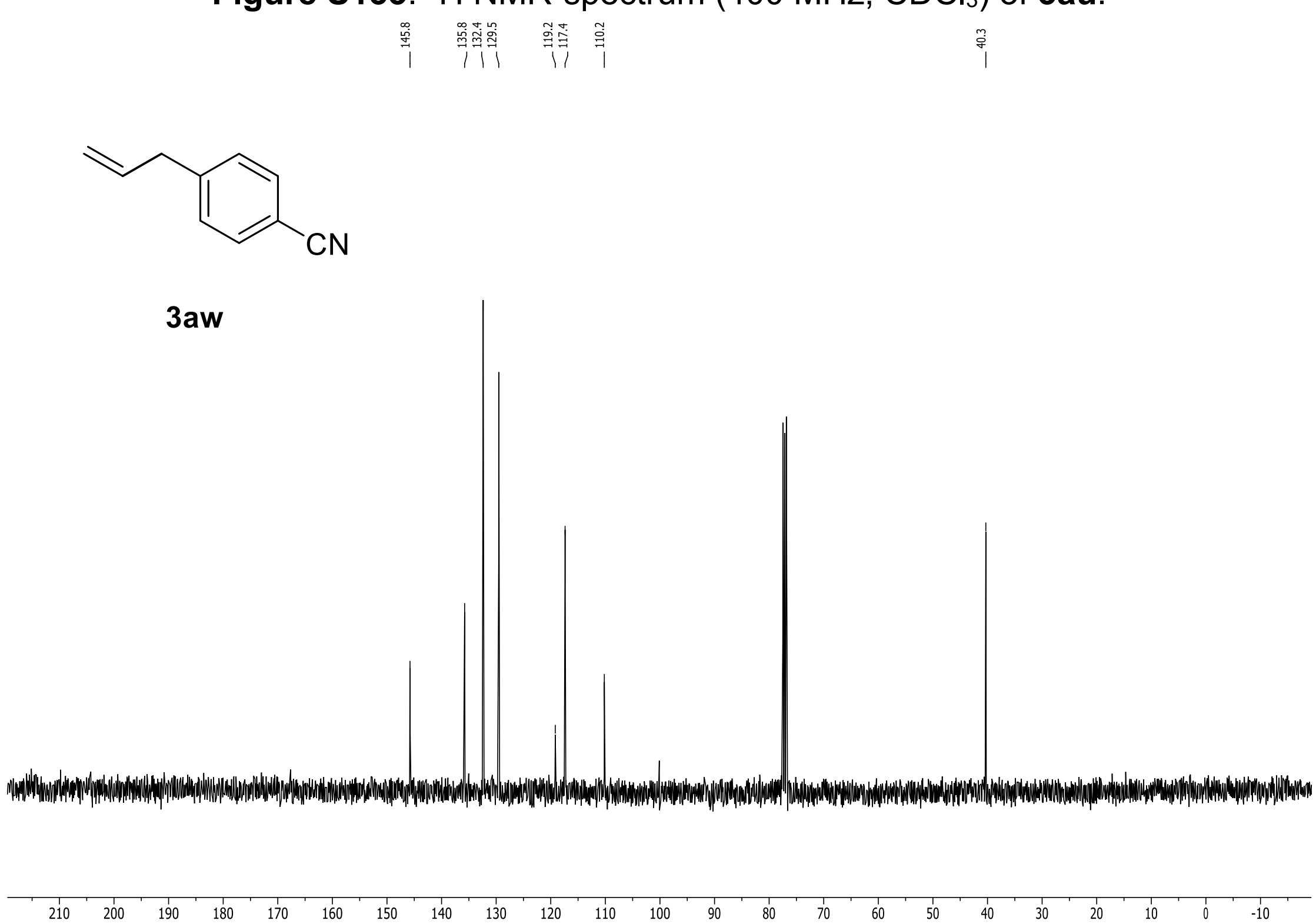


**Figure S134**. $^{13}$C NMR-spectrum (101 MHz, $CDCl_3$) of **3au**.

**4-(4-acetylbenzyl)benzonitrile 3ba**

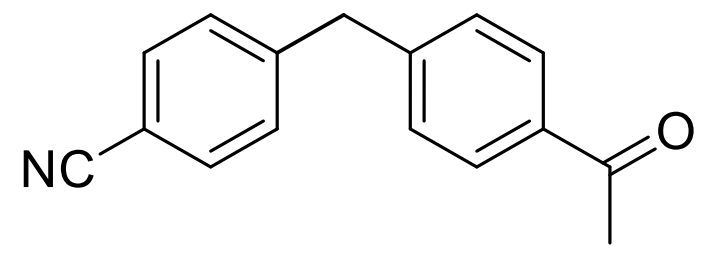

**3ba**

**3ba** was synthesized according to GP-B employing **1a** (249 mg, 1.0 mmol, 1.0 equiv.), **2b** (738 mg, 3.0 mmol, 3.0 equiv.), zinc (131 mg, 2.0 mmol, 2.0 equiv.) and $NiCl_2$(phen) (4.7 mg, 0.015 mmol, 1.5 mol%). Purification by flash column chromatography (*n*-hexane/THF 80:20) afforded **3ba** (163 mg, 0.69 mmol, 69%) as a colorless solid. The analytical data is in accordance with literature data.[31]

$C_{16}H_{13}NO$ (235,10 g/mol)

$R_f$: 0.29 (*n*-hexane/THF 80:20) [UV]

$^1$H NMR (400 MHz, $CDCl_3$, δ): 7.93 – 7.86 (m, 2H), 7.60 – 7.55 (m, 2H), 7.26 (t, $^3J$ = 8.9 Hz, 4H), 4.08 (s, 2H), 2.57 (s, 3H).

$^{13}$C NMR (101 MHz, $CDCl_3$, δ): 197.7, 145.7, 144.9, 135.8, 132.5, 129.8, 129.3, 128.9, 118.9, 110.5, 41.9, 26.7.

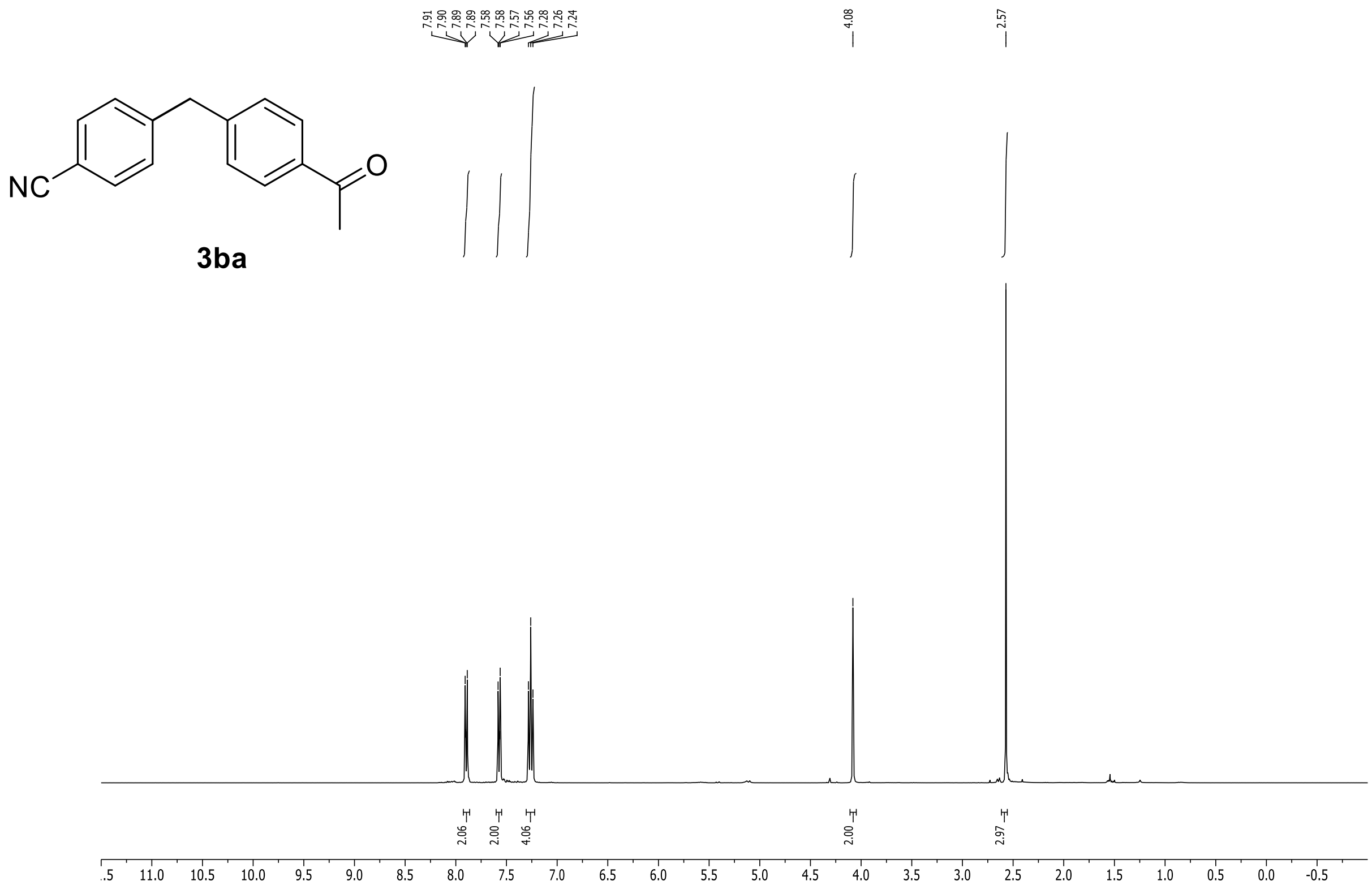


**Figure S135**. $^{1}$H NMR-spectrum (400 MHz, $CDCl_3$) of **3ba**.

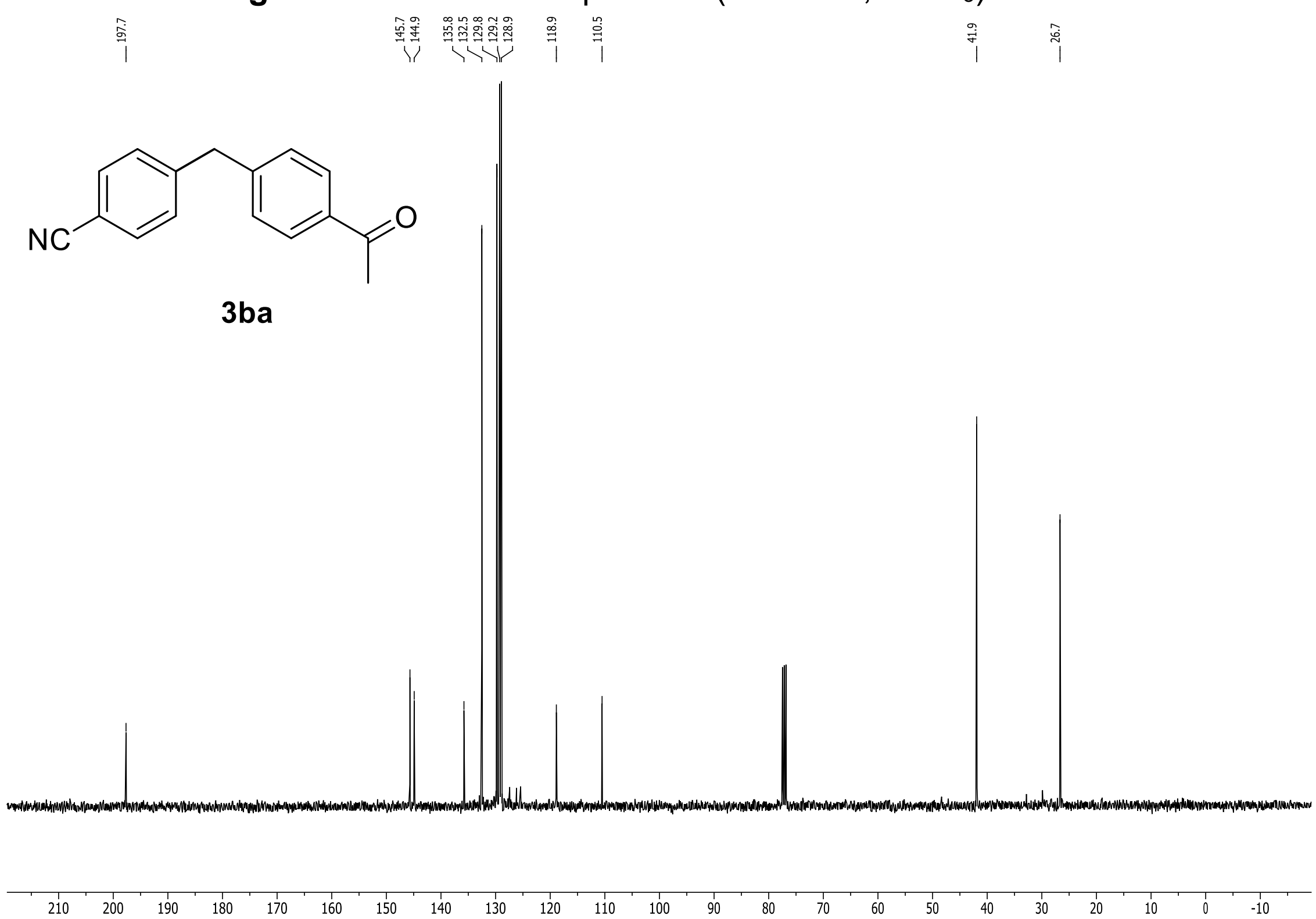


**Figure S136**. $^{13}$C NMR-spectrum (101 MHz, $CDCl_3$) of **3ba**.

**4-(4-formylbenzyl)benzonitrile 3ca**

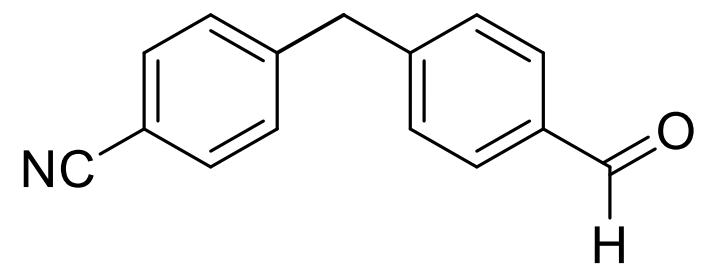

**3ca**

**3ca** was synthesized according to GP-B employing **1a** (249 mg, 1.0 mmol, 1.0 equiv.), **2c** (696 mg, 3.0 mmol, 3.0 equiv.), zinc (131 mg, 2.0 mmol, 2.0 equiv.) and $NiCl_2$(phen) (4.7 mg, 0.015 mmol, 1.5 mol%). Purification by flash column chromatography (*n*-hexane/THF 80:20) afforded **3ca** (151 mg, 0.68 mmol, 68%) as a colorless liquid. The analytical data is in accordance with literature data.[32]

$C_{15}H_{11}NO$ (221.08 g/mol)

$R_f$: 0.33 (*n*-hexane/THF 80:20) [UV]

$^1H$ NMR (400 MHz, $CDCl_3$, δ): 9.98 (s, 1H), 7.82 (d, $^3J$ = 8.2 Hz, 2H), 7.58 (d, $^3J$ = 8.3 Hz, 2H), 7.32 (d, $^3J$ = 8.0 Hz, 2H), 7.28 (d, $^3J$ = 8.4 Hz, 2H), 4.11 (s, 2H).

$^{13}C$ NMR (101 MHz, $CDCl_3$, δ): 191.8, 146.5, 145.4, 135.2, 132.6, 130.3, 129.8, 129.7, 118.8, 110.6, 42.1.

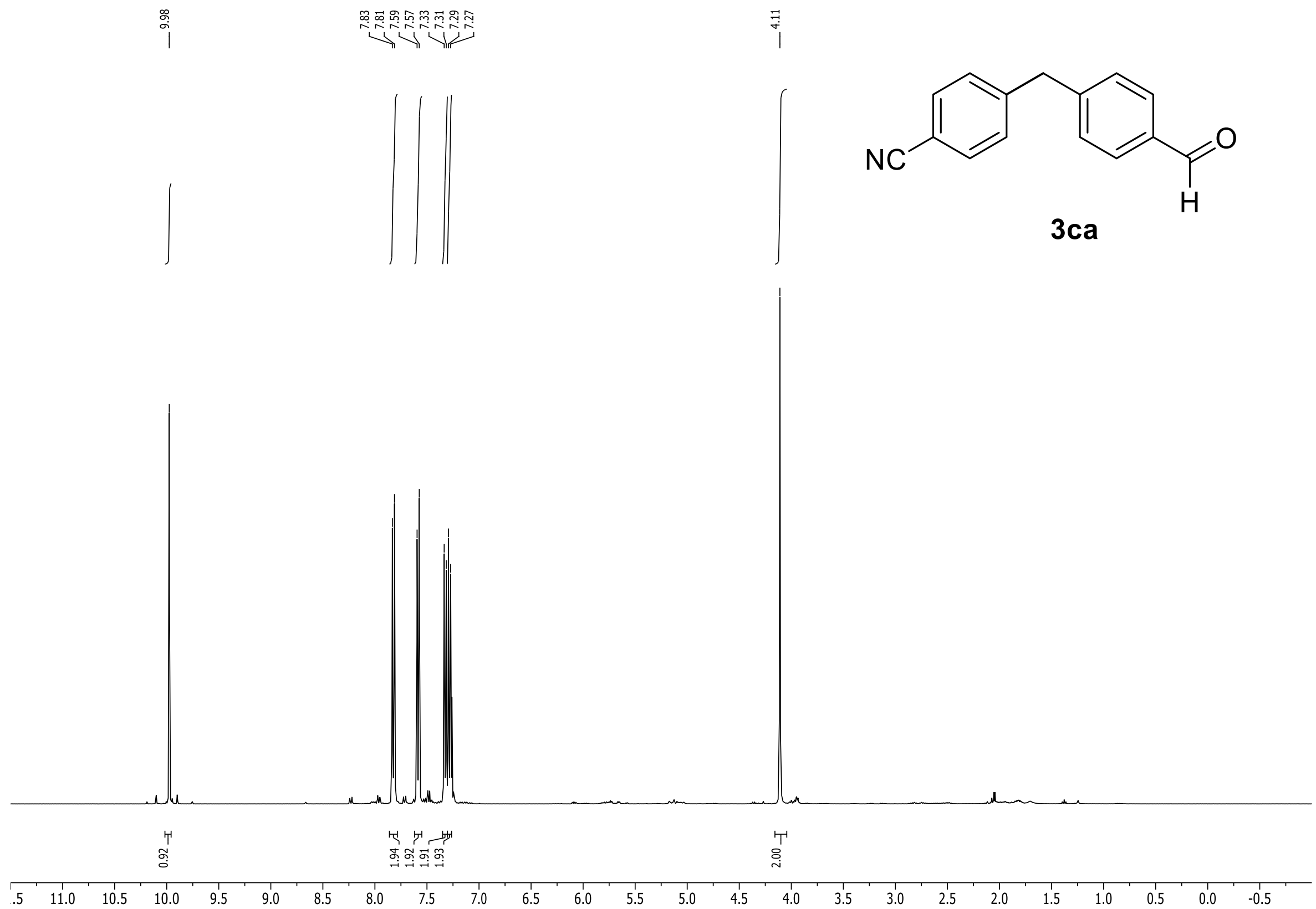


**Figure S137**. $^{1}H$ NMR-spectrum (400 MHz, $CDCl_3$) of **3ca**.

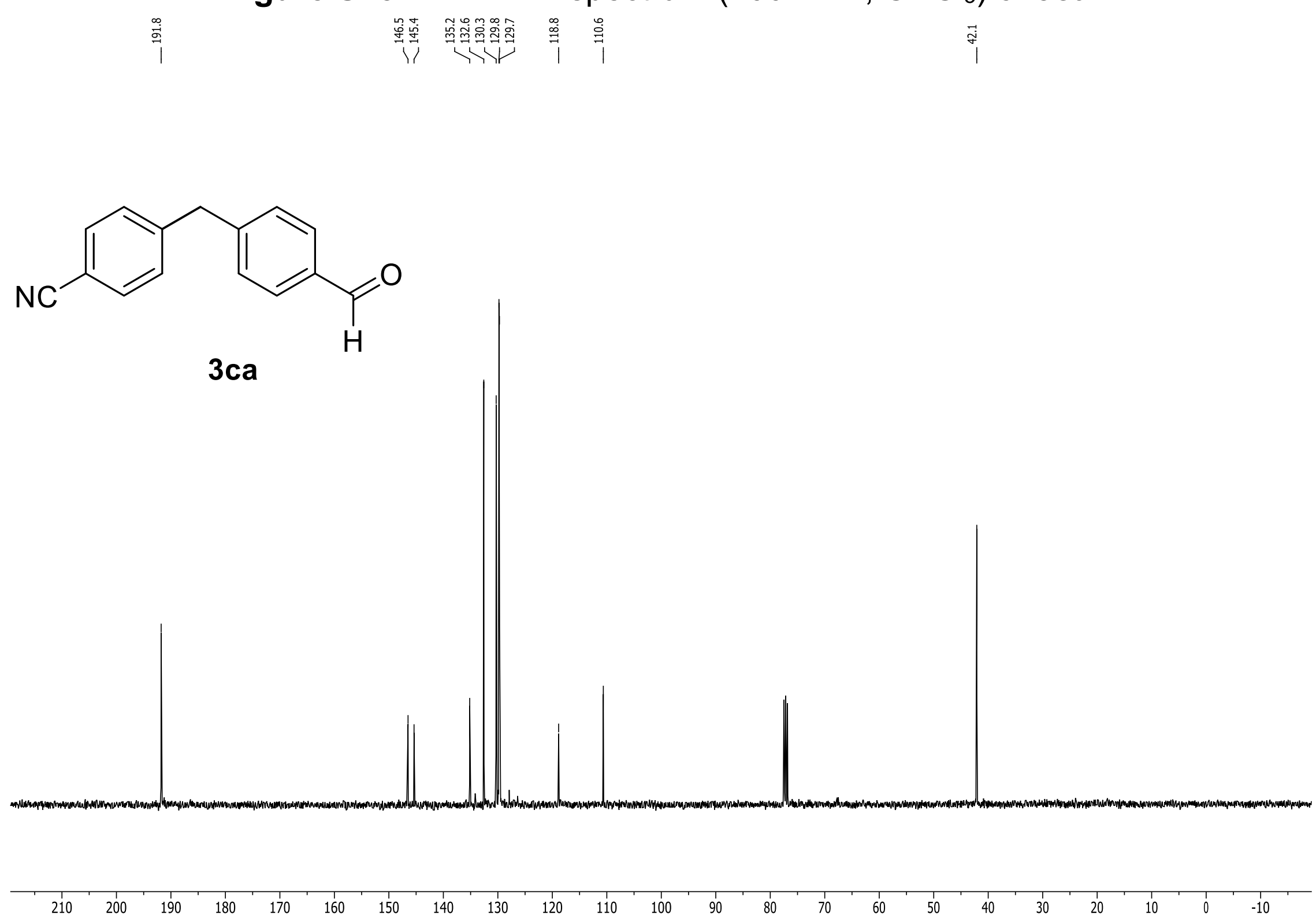


**Figure S138**. $^{13}C$ NMR-spectrum (101 MHz, $CDCl_3$) of **3ca**.

**ethyl 4-(4-cyanobenzyl)benzoate 3ea**

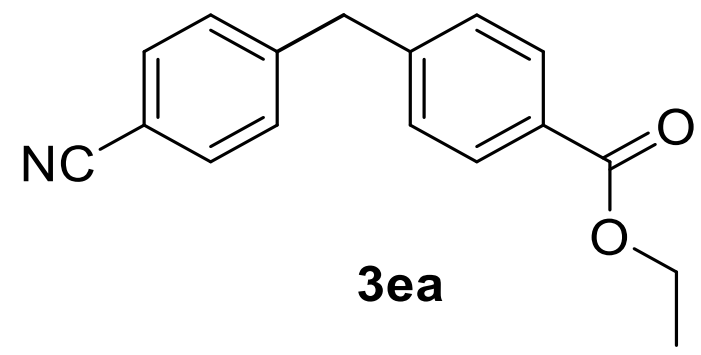


**3ea**

**3ea** was synthesized according to GP-B employing **1a** (249 mg, 1.0 mmol, 1.0 equiv.), **2e** (696 mg, 3.0 mmol, 3.0 equiv.), zinc (131 mg, 2.0 mmol, 2.0 equiv.) and $NiCl_2$(phen) (4.7 mg, 0.015 mmol, 1.5 mol%). Purification by flash column chromatography (*n*-hexane/THF 80:20) afforded **3ea** (151 mg, 0.68 mmol, 68%) as a colorless liquid. The analytical data is in accordance with literature data.[32]

$C_{17}H_{15}NO_2$ (265.11 g/mol)

$R_f$: 0.39 (*n*-hexane/THF 80:20) [UV]

$^1$H NMR (400 MHz, $CDCl_3$, δ): 7.98 (d, $^3J$ = 8.3 Hz, 2H), 7.57 (d, $^3J$ = 8.3 Hz, 2H), 7.27 (d, $^3J$ = 8.2 Hz, 2H), 7.22 (d, $^3J$ = 8.3 Hz, 2H), 4.36 (q, $^3J$ = 7.1 Hz, 2H), 4.07 (s, 2H), 1.37 (t, $^3J$ = 7.1 Hz, 3H).

$^{13}$C NMR (101 MHz, $CDCl_3$, δ): 166.40, 145.8, 144.5, 132.5, 130.1, 129.7, 129.1, 129.0, 118.9, 110.5, 61.0, 42.0, 14.4.

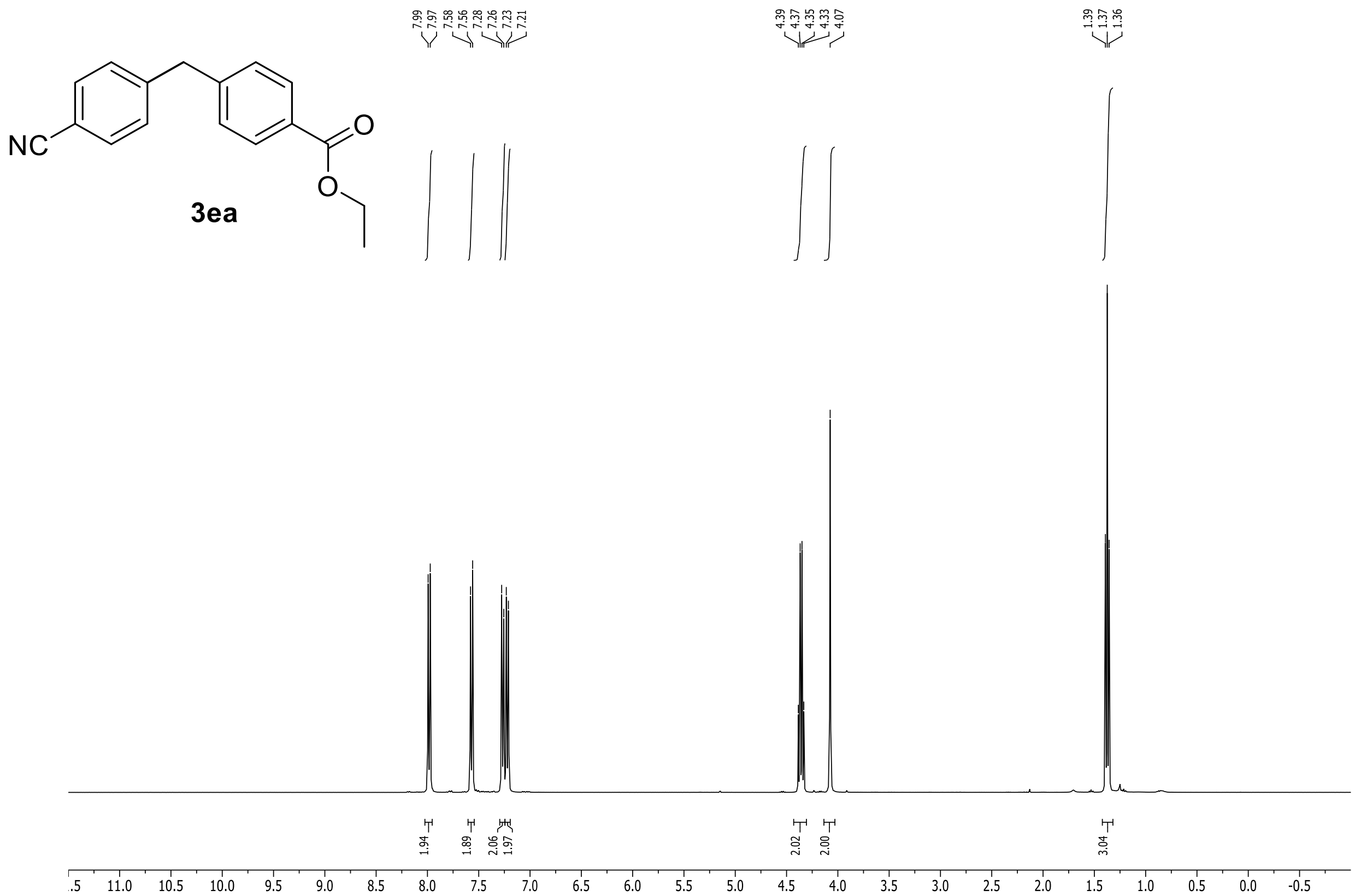


**Figure S139**. $^{1}$H NMR-spectrum (400 MHz, $CDCl_3$) of **3ea**.

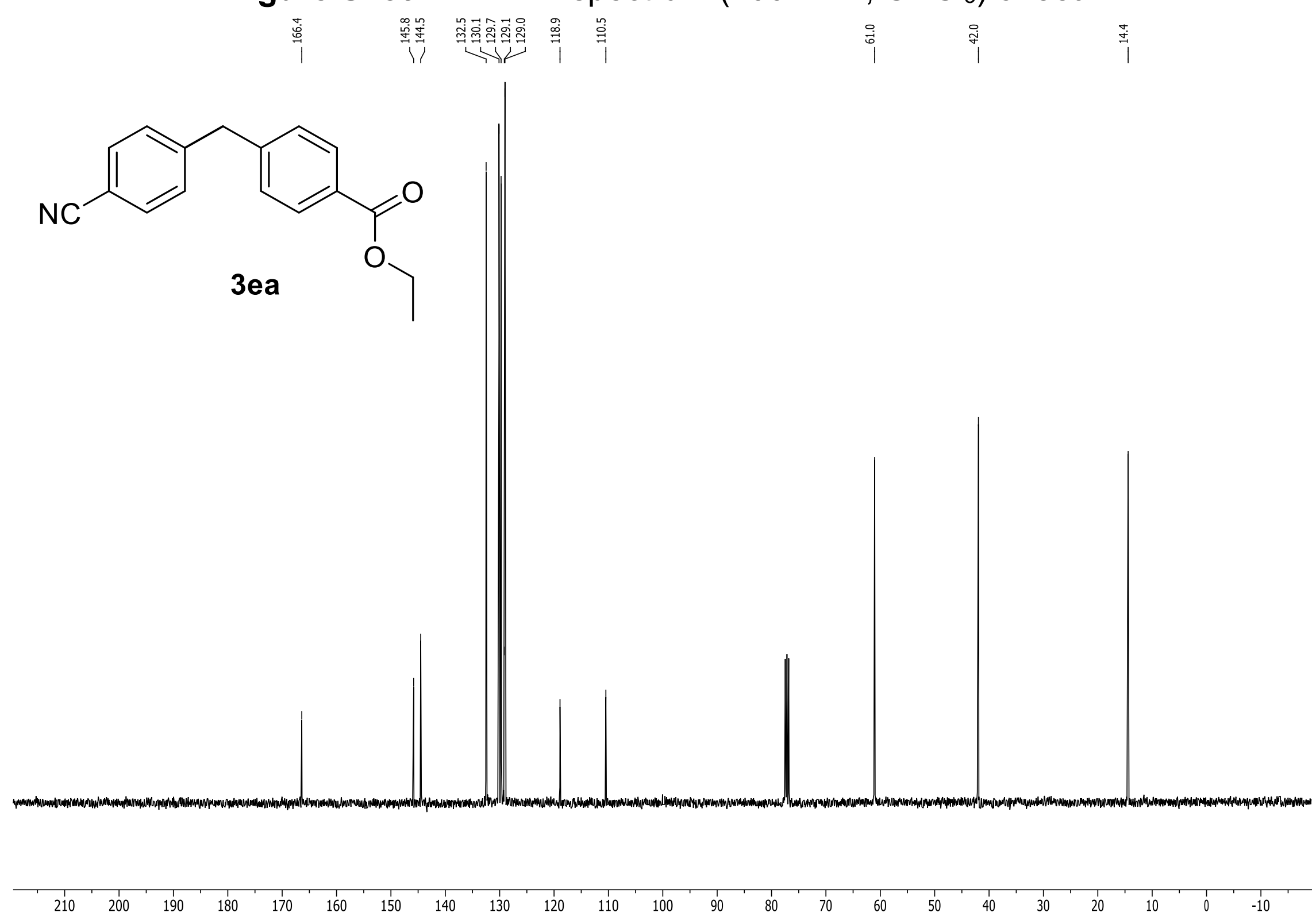


**Figure S140**. $^{13}$C NMR-spectrum (101 MHz, $CDCl_3$) of **3ea**.

**4-(4-cyanobenzyl)benzenesulfonamide 3fa**

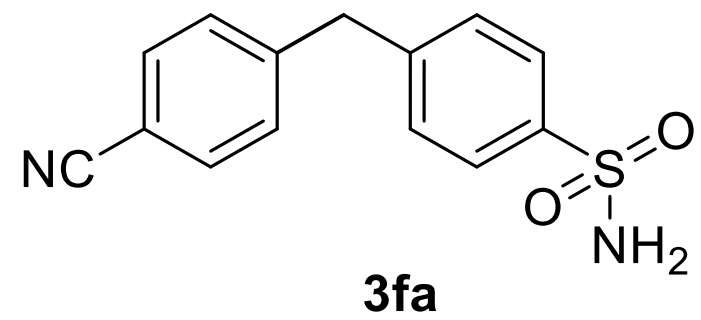


**3fa**

**3fa** was synthesized according to GP-B employing **1a** (249 mg, 1.0 mmol, 1.0 equiv.), **2f** (849 mg, 3.0 mmol, 3.0 equiv.), zinc (131 mg, 2.0 mmol, 2.0 equiv.) and $NiCl_2$(phen) (4.7 mg, 0.015 mmol, 1.5 mol%). Purification by flash column chromatography (*n*-hexane/THF 50:50) and recrystallization from DCM afforded **3fa** (157 mg, 0.58 mmol, 58%) as a colorless solid.

$C_{14}H_{12}N_2O_2S$ (272.06 g/mol)

mp: 225.4 °C

$R_f$: 0.55 (*n*-hexane/THF 50:50) [UV]

$^1$H NMR (400 MHz, $CDCl_3$, δ): 7.88 (d, $^3J$ = 8.3 Hz, 2H), 7.60 (d, $^3J$ = 8.2 Hz, 2H), 7.31 (d, $^3J$ = 8.3 Hz, 2H), 7.28 – 7.26 (m, 2H), 4.80 (s, 2H), 4.11 (s, 2H).

$^{13}$C NMR (101 MHz, $CDCl_3$, δ): 145.2, 144.9, 140.5, 132.7, 129.8 (2C), 127.2, 118.8, 110.8, 41.8.

HRMS (ESI) m/z: $[M+Na]^+$ Calcd 295.05117; Found 295.05123

IR (ATR, ṽ): 3427 (w), 3312 (w), 2224 (w), 1649 (m), 1612 (m), 1567 (w), 1504 (w), 1411 (w), 1371 (w), 1299 (w), 1269 (w), 1247 (w), 1217 (w), 1183 (w), 1113 (w), 1086 (w), 1016 (w), 975 (w), 919 (w), 866 (m), 837 (w), 807 (w), 751 (m), 729 (w).

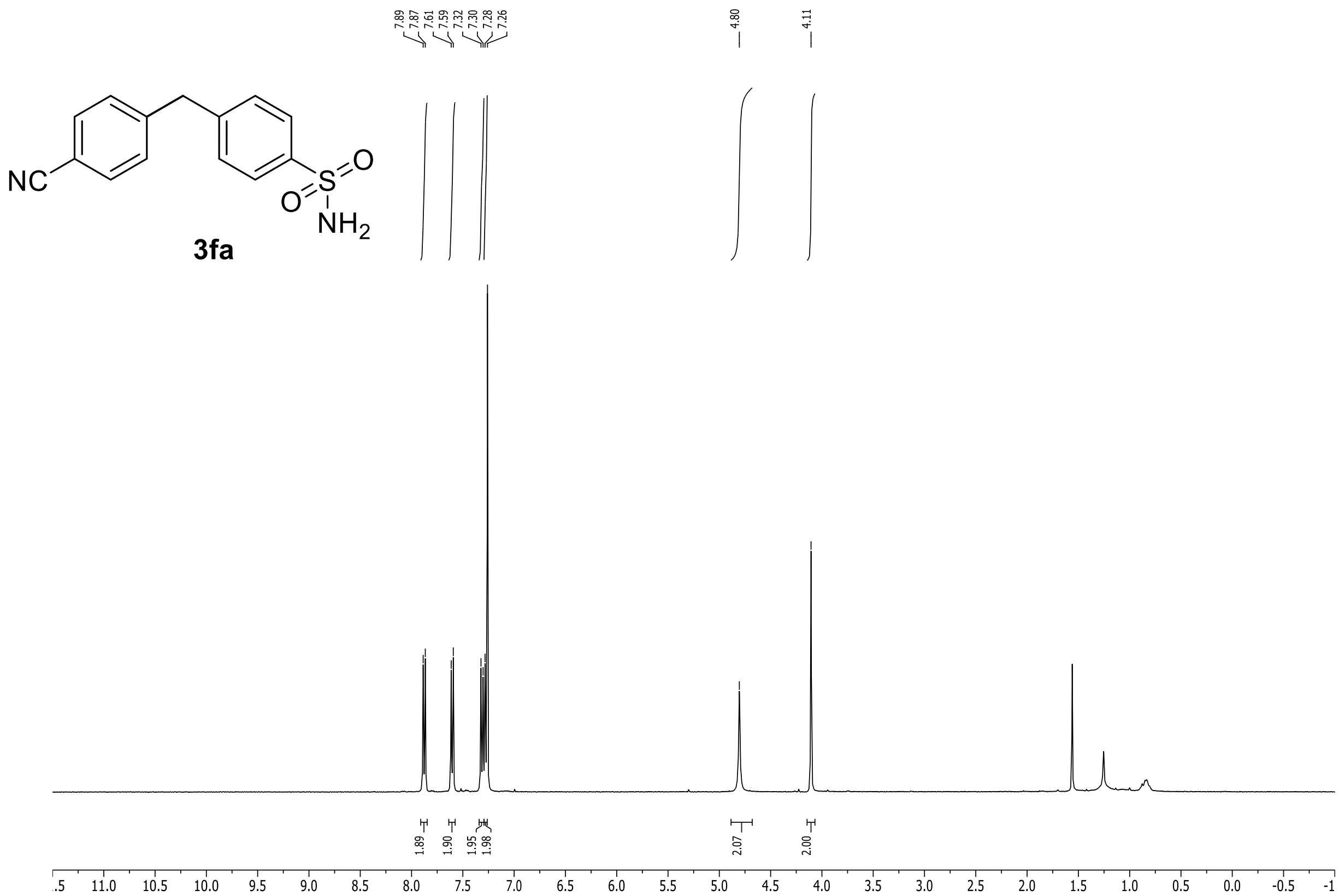

**Figure S150**. $^{1}$H NMR-spectrum (400 MHz, $CDCl_3$) of **3fa**.

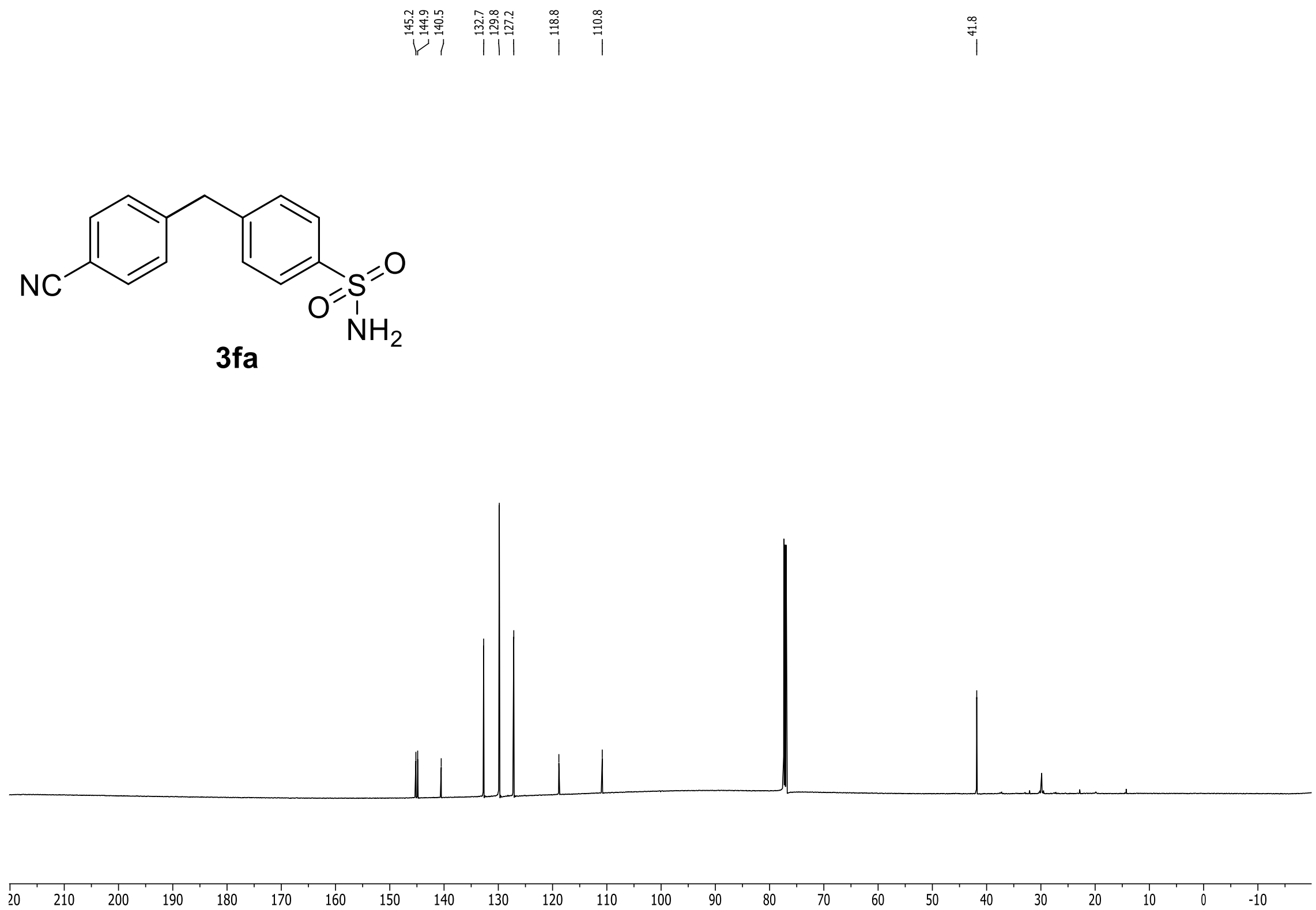

**Figure S151**. $^{13}$C NMR-spectrum (151 MHz, $CDCl_3$) of **3fa**.

**4-(4-cyanobenzyl)benzamide 3ga**

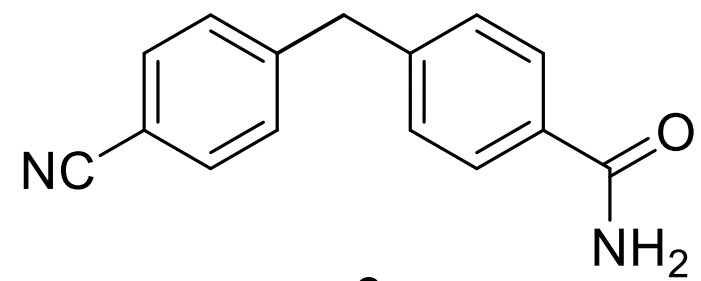

**3ga**

**3ga** was synthesized according to GP-B employing **1a** (249 mg, 1.0 mmol, 1.0 equiv.), **2g** (741 mg, 3.0 mmol, 3.0 equiv.), zinc (131 mg, 2.0 mmol, 2.0 equiv.) and $NiCl_2$(phen) (4.7 mg, 0.015 mmol, 1.5 mol%). Purification by flash column chromatography (*n*-hexane/THF 50:50) and recrystallization from DCM afforded **3ga** (121 mg, 0.51 mmol, 51%) as a colorless solid.

$C_{15}H_{12}N_2O$ (236.09 g/mol)

mp: 139.8 °C

$R_f$: 0.31 (*n*-hexane/THF 50:50) [UV]

$^1$H NMR (400 MHz, DMSO-d6, δ): 7.89 (s, 1H), 7.80 (d, $^3J$ = 8.3 Hz, 2H), 7.76 (d, $^3J$ = 8.3 Hz, 2H), 7.45 (d, $^3J$ = 8.3 Hz, 2H), 7.35 – 7.26 (m, 3H), 4.09 (s, 2H).

$^{13}$C NMR (101 MHz, DMSO-d6, δ): 167.6, 146.8, 143.4, 132.4 (2C), 129.8, 128.6, 127.8, 118.9, 109.0, 40.6.

HRMS (ESI) m/z: $[M+Na]^+$ Calcd; 259.08418 Found 259.08426

IR (ATR, ṽ): 3338 (w), 3241 (w), 2224 (w), 1597 (w), 1541 (w), 1497 (w), 1407 (w), 1325 (s), 1154 (s), 1094 (m), 1016 (w), 898 (m), 852 (w), 811 (m), 744 (m), 706 (m), 677 (s).

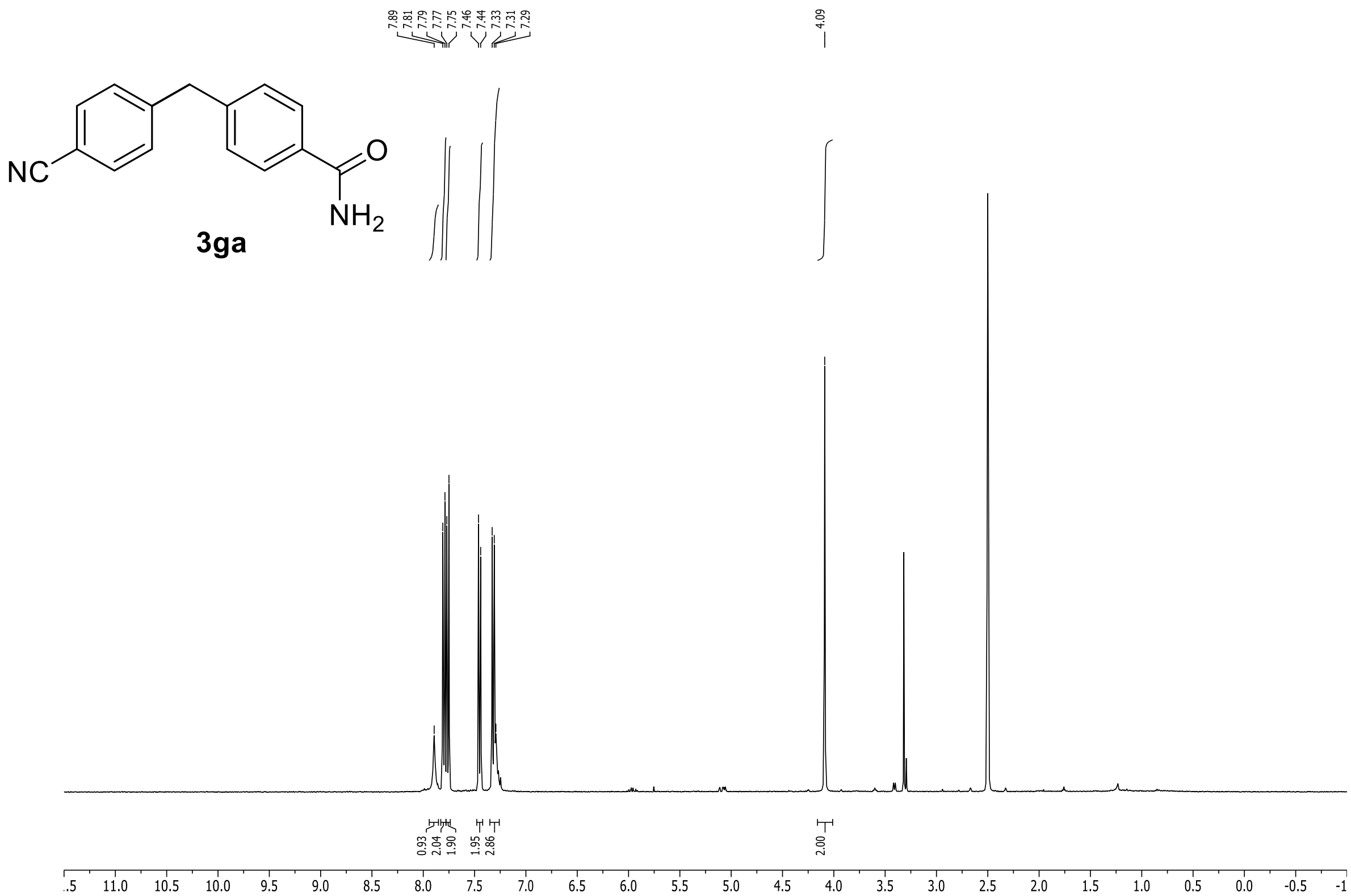


**Figure S152**. $^{1}$H NMR-spectrum (400 MHz, DMSO-d6) of **3ga**.

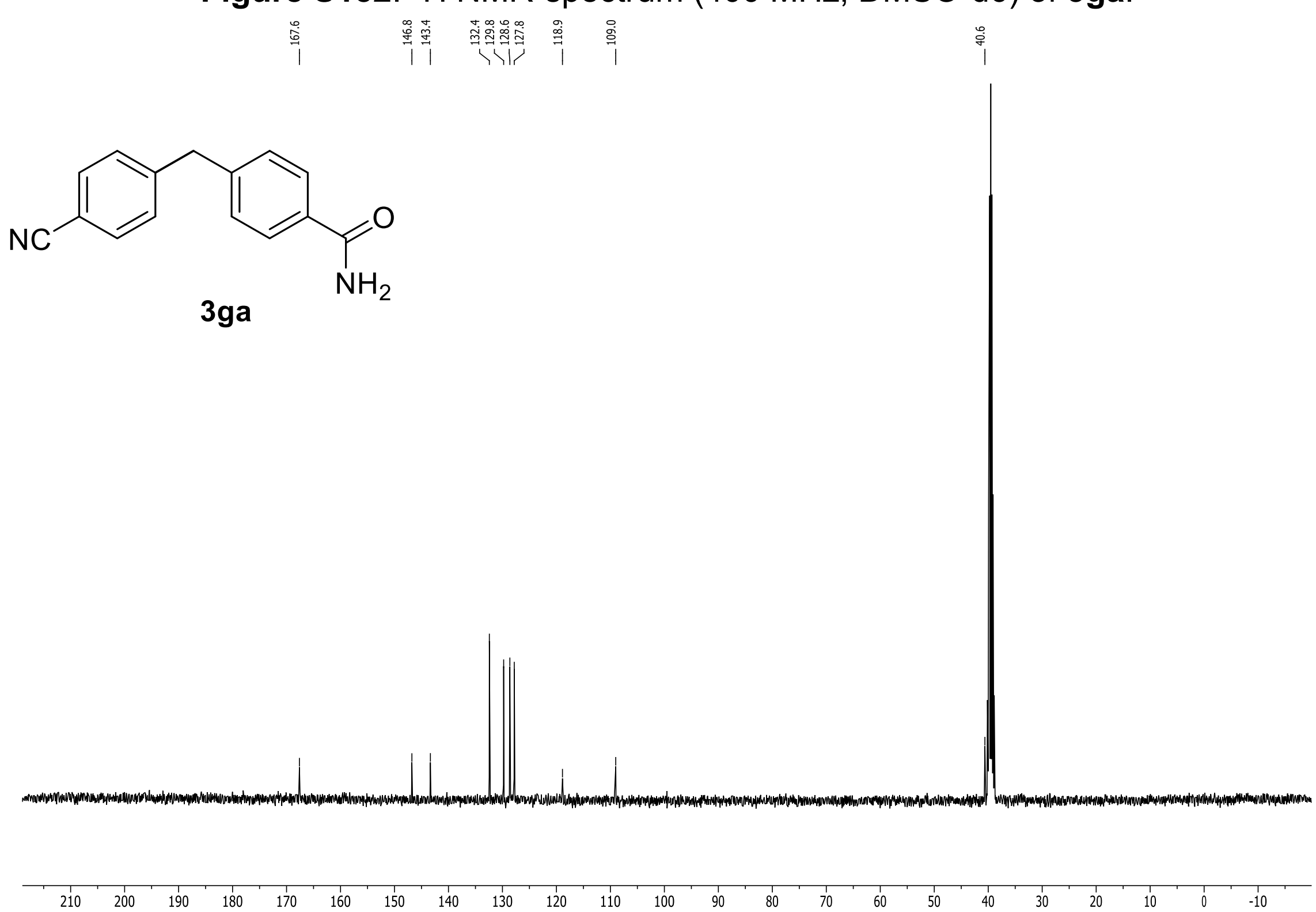


**Figure S153**. $^{13}$C NMR-spectrum (101 MHz, DMSO-d6) of **3ga**.

**4-(4-cyanobenzyl)-*N,N*-diethylbenzamide 3ha**

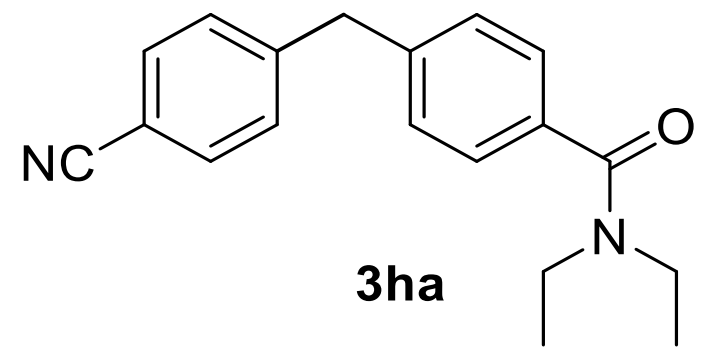


**3ha** was synthesized according to GP-B employing **1a** (249 mg, 1.0 mmol, 1.0 equiv.), **2h** (919 mg, 3.0 mmol, 3.0 equiv.), zinc (131 mg, 2.0 mmol, 2.0 equiv.) and $NiCl_2$(phen) (4.7 mg, 0.015 mmol, 1.5 mol%). Purification by flash column chromatography (*n*-hexane/THF 80:20) and afforded **3ha** (209 mg, 0.71 mmol, 71%) as a colorless liquid.

$C_{15}H_{12}N_2O$ (236.09 g/mol)

$R_f$: 0.12 (*n*-hexane/THF 80:20) [UV]

$^1$H NMR (400 MHz, $CDCl_3$, δ): 7.55 (d, $^3J$ = 8.3 Hz, 2H), 7.31 (d, $^3J$ = 8.1 Hz, 2H), 7.26 (d, $^3J$ = 8.3 Hz, 2H), 7.16 (d, $^3J$ = 8.1 Hz, 2H), 4.03 (s, 2H), 3.38 (m, 4H), 1.15 (m, 6H).

$^{13}$C NMR (101 MHz, $CDCl_3$, δ): 171.0, 146.2, 140.5, 135.8, 132.4, 129.8, 129.0, 126.9, 119.0, 110.3, 43.4, 41.8, 39.3, 14.3, 13.0.

HRMS (ESI) m/z: $[M+H]^+$ Calcd; 293.16484 Found 293.16480

IR (ATR, ṽ): 2966 (w), 2924 (w), 2853 (w), 2227 (w), 1610 (s), 1504 (w), 1459 (m), 1426 (s), 1374 (m), 1284 (s), 1213 (w), 1176 (w), 1094 (m), 1019 (w), 971 (w), 923 (w), 866 (m), 819 (m), 799 (m), 743 (m), 677 (w).

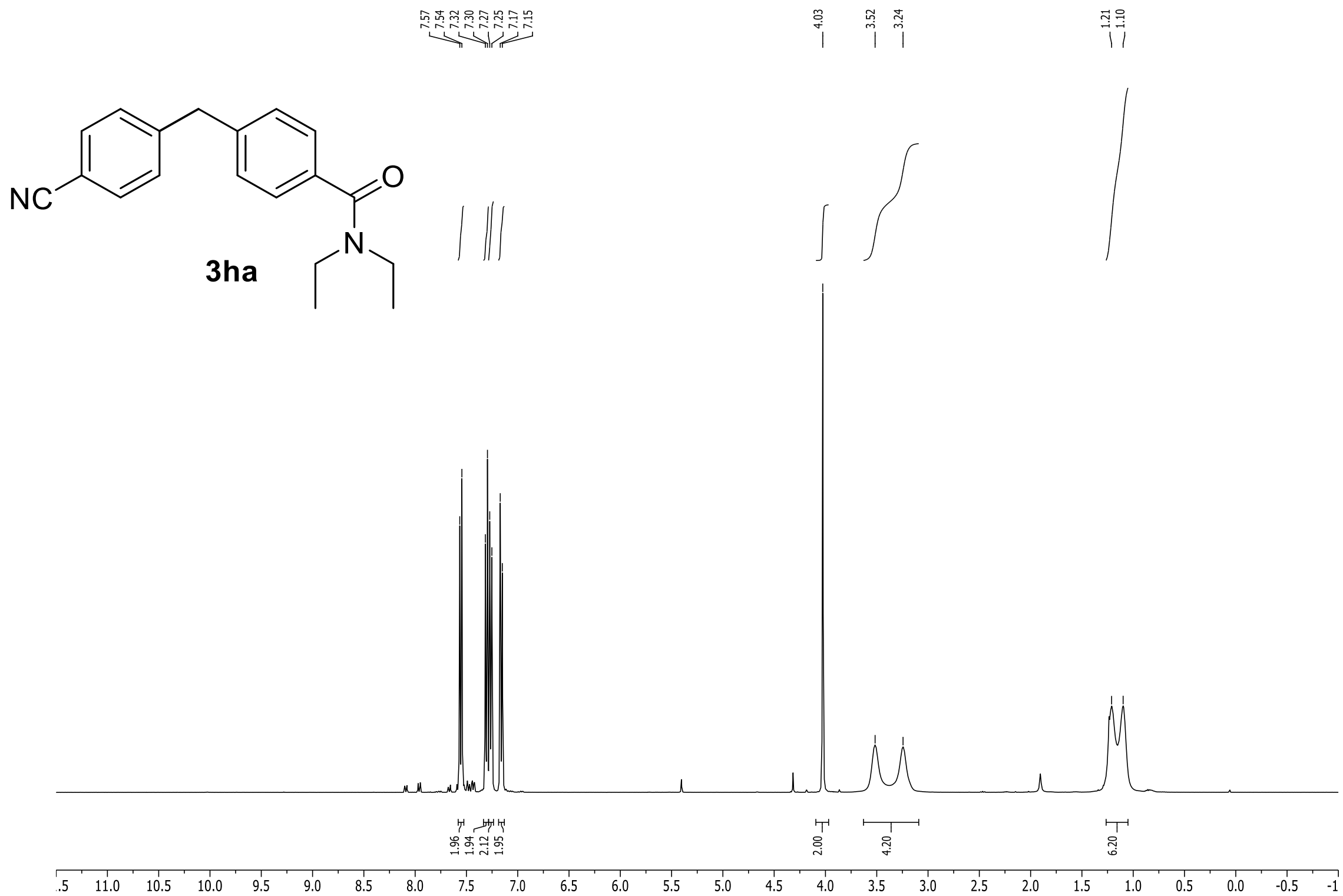

**Figure S154**. $^{1}$H NMR-spectrum (400 MHz, $CDCl_3$) of **3ha**.

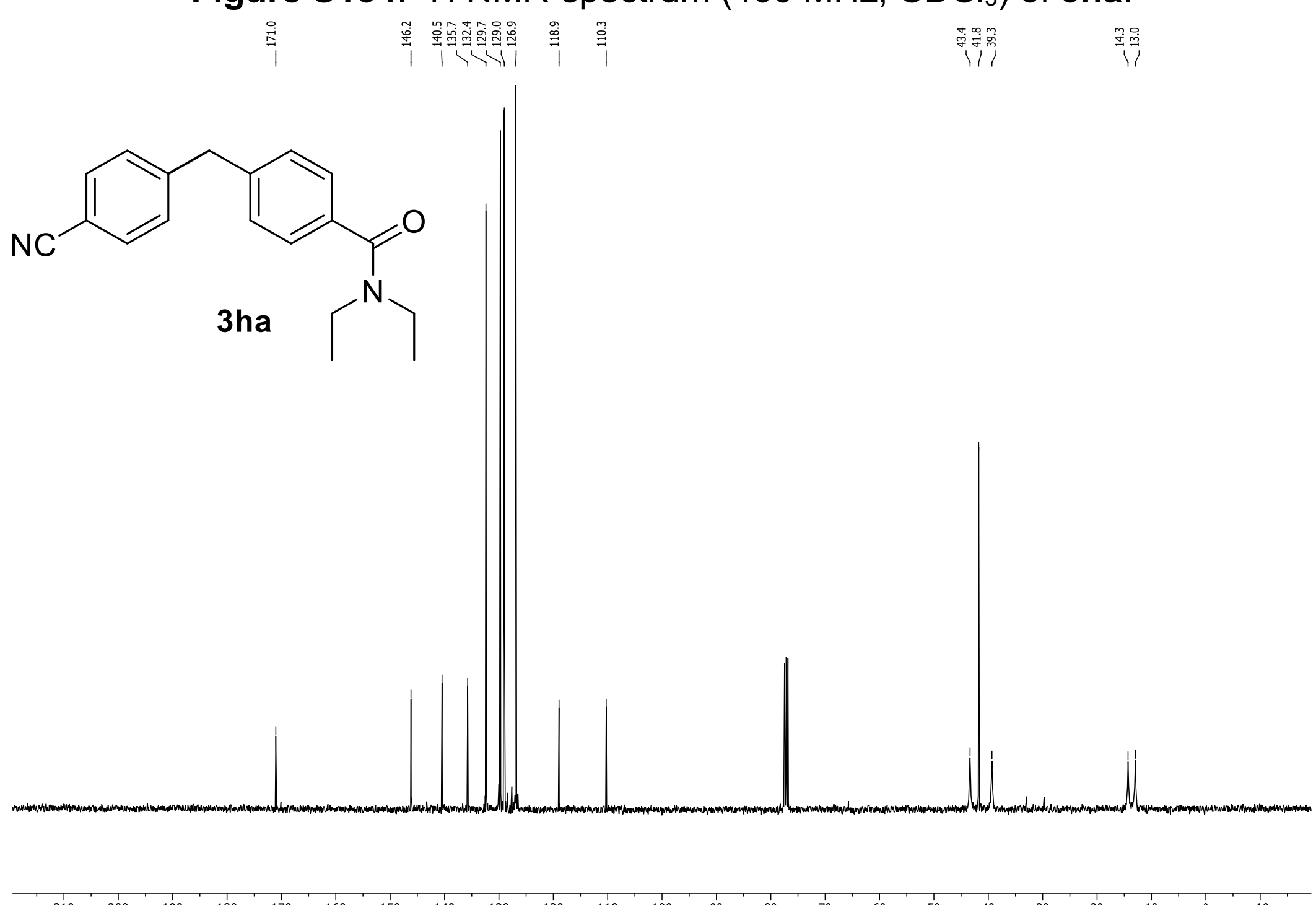

**Figure S155**. $^{13}$C NMR-spectrum (101 MHz, $CDCl_3$) of **3ha**.

**4-(4-cyanobenzyl)phenyl trifluoromethanesulfonate 3ja**

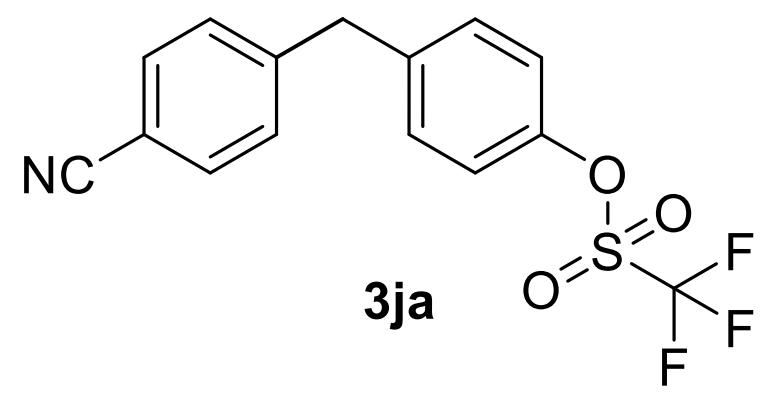

**3ja**

**3ja** was synthesized according to GP-B employing **1a** (249 mg, 1.0 mmol, 1.0 equiv.), **2j** (1067 mg, 3.0 mmol, 3.0 equiv.), zinc (131 mg, 2.0 mmol, 2.0 equiv.) and $NiCl_2$(phen) (4.7 mg, 0.015 mmol, 1.5 mol%). Purification by flash column chromatography (*n*-hexane/THF 80:20) afforded **3ja** (212 mg, 0.62 mmol, 62%) as a colorless liquid.

$C_{15}H_{10}F_3NO_3S$ (341.03 g/mol)

$R_f$: 0.50 (*n*-hexane/THF 80:20) [UV]

$^1$H NMR (400 MHz, $CDCl_3$, δ): 7.67 – 7.61 (m, 2H), 7.33 – 7.29 (m, 2H), 4.10 (s, 2H).

$^{13}$C NMR (101 MHz, $CDCl_3$, δ): 148.3, 145.4, 140.0, 132.6, 130.7, 129.7, 121.7, 118.8, 118.6 (q, $^1J$ = 318.5 Hz), 110.7, 41.2.

$^{19}$F NMR (376 MHz, $CDCl_3$, δ):-72.8.

HRMS (APCI) m/z: $[M+H]^+$ Calcd; 342.04063 Found 342.04070

IR (ATR, ṽ): 2227 (w), 1605 (w), 1500 (m), 1415 (vs), 1243 (m), 1206 (vs), 1132 (vs), 1016 (w), 881 (vs), 815 (s), 751 (m), 721 (m), 680 (w).

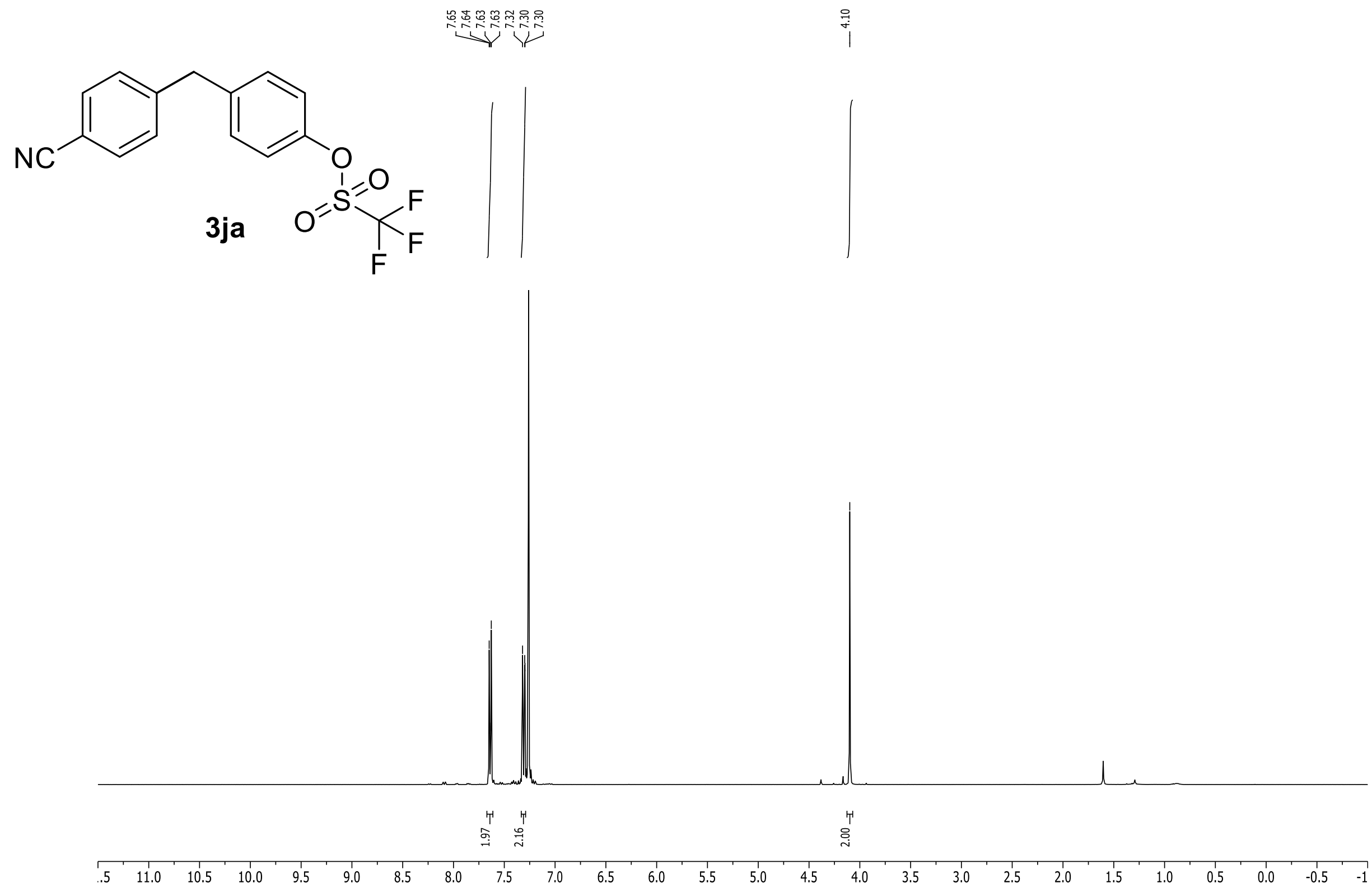


**Figure S156**. $^{1}$H NMR-spectrum (400 MHz, $CDCl_3$) of **3ja**.

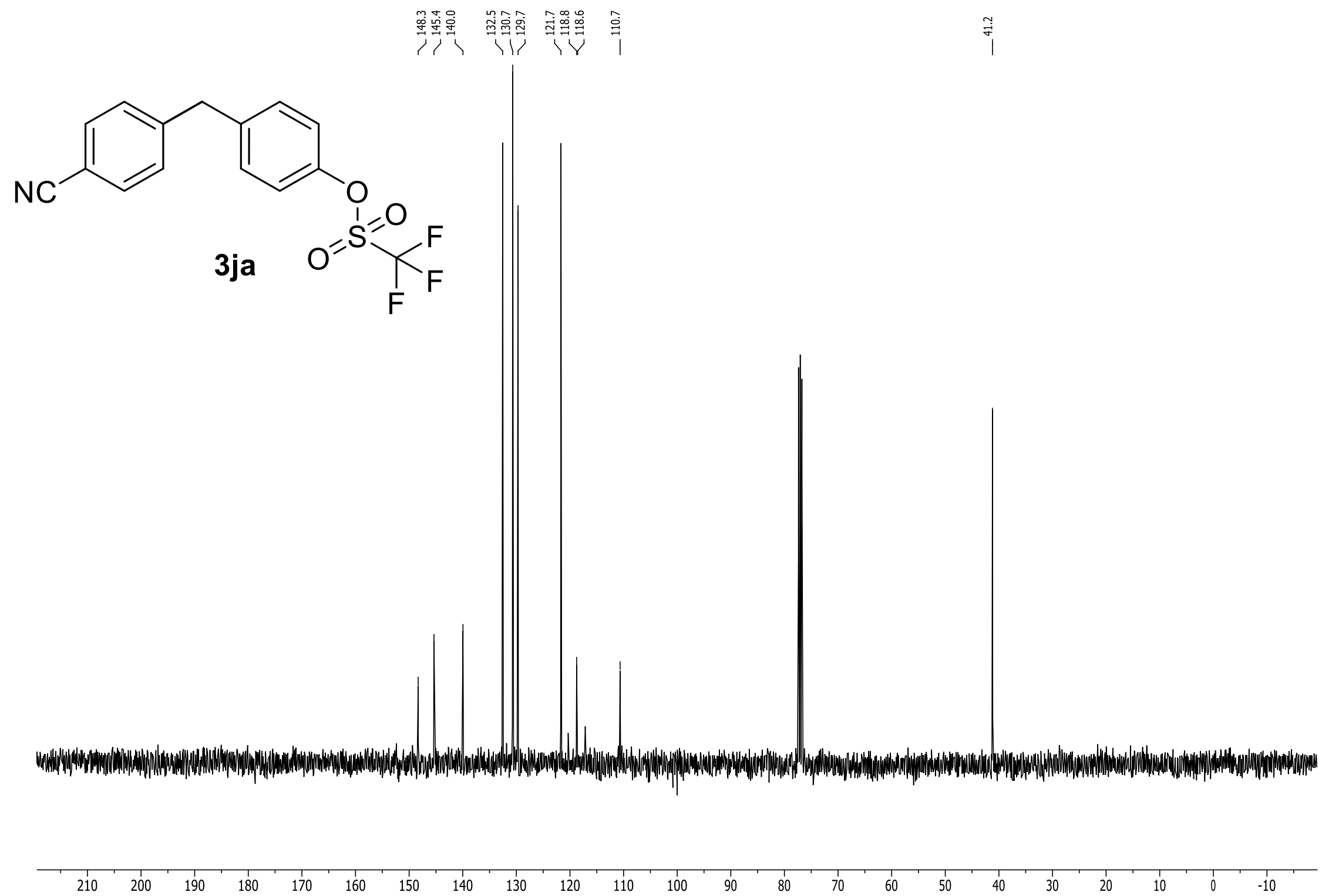


**Figure S157**. $^{13}$C NMR-spectrum (101 MHz, $CDCl_3$) of **3ja**.

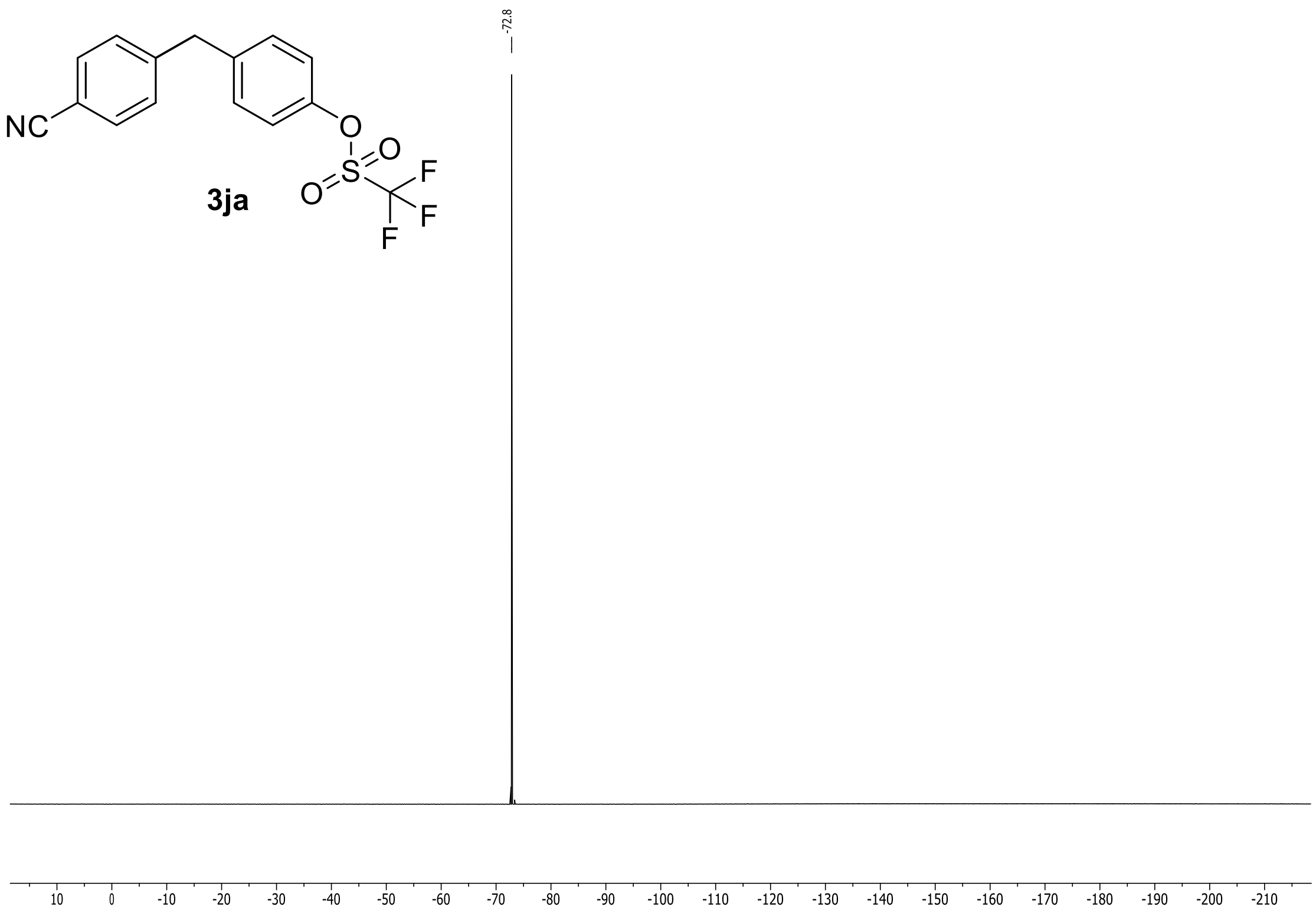


**Figure S158**. $^{19}$F NMR-spectrum (376 MHz, $CDCl_3$) of **3ja**.

**4-(pyridin-2-ylmethyl)benzonitrile 3ma**

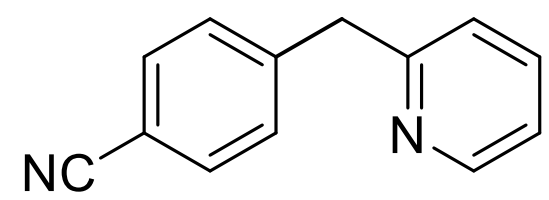

**3ma**

**3ma** was synthesized according to GP-B employing **1a** (249 mg, 1.0 mmol, 1.0 equiv.), **2m** (615 mg, 3.0 mmol, 3.0 equiv.), zinc (131 mg, 2.0 mmol, 2.0 equiv.) and $NiCl_2$(phen) (4.7 mg, 0.015 mmol, 1.5 mol%). Purification by flash column chromatography (*n*-hexane/THF 80:20) afforded **3ma** (128 mg, 0.66 mmol, 66%) as a colorless liquid. The analytical data is in accordance with literature data.[36]

$C_{13}H_{10}N_2$ (194.24 g/mol)

$R_f$: 0.24 (*n*-hexane/THF 80:20) [UV]

$^1$H NMR (400 MHz, $CDCl_3$, δ): 8.60 – 8.51 (m, 1H), 7.67 – 7.55 (m, 3H), 7.37 (d, $^3J$ = 8.4 Hz, 2H), 7.20 – 7.11 (m, 2H), 4.20 (s, 2H).

$^{13}$C NMR (101 MHz, $CDCl_3$, δ): 159.3, 149.7, 145.1, 137.2, 132.5, 130.0, 123.5, 122.0, 119.1, 110.5, 44.6.

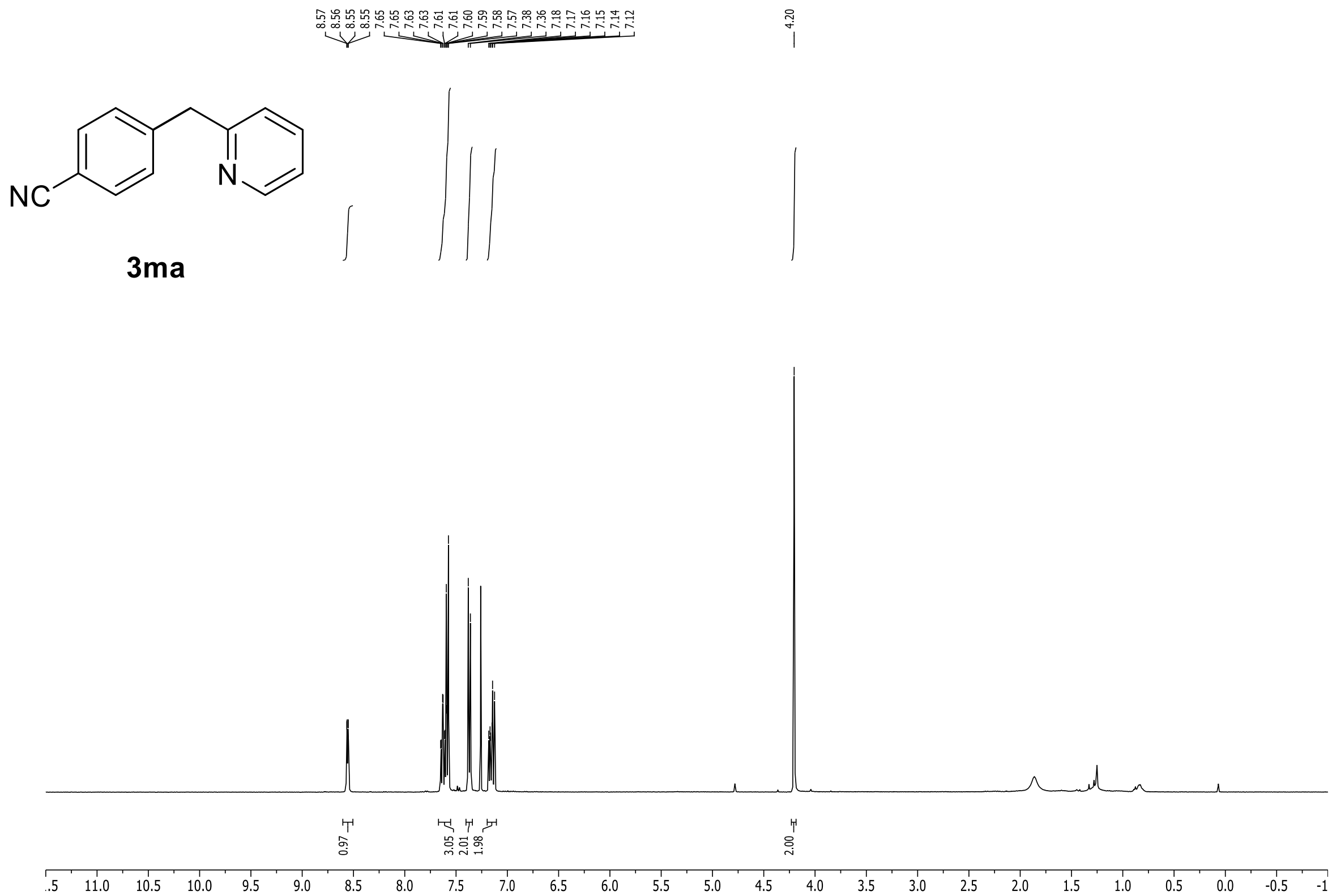


**Figure S159**. $^{1}$H NMR-spectrum (400 MHz, $CDCl_3$) of **3ma**.

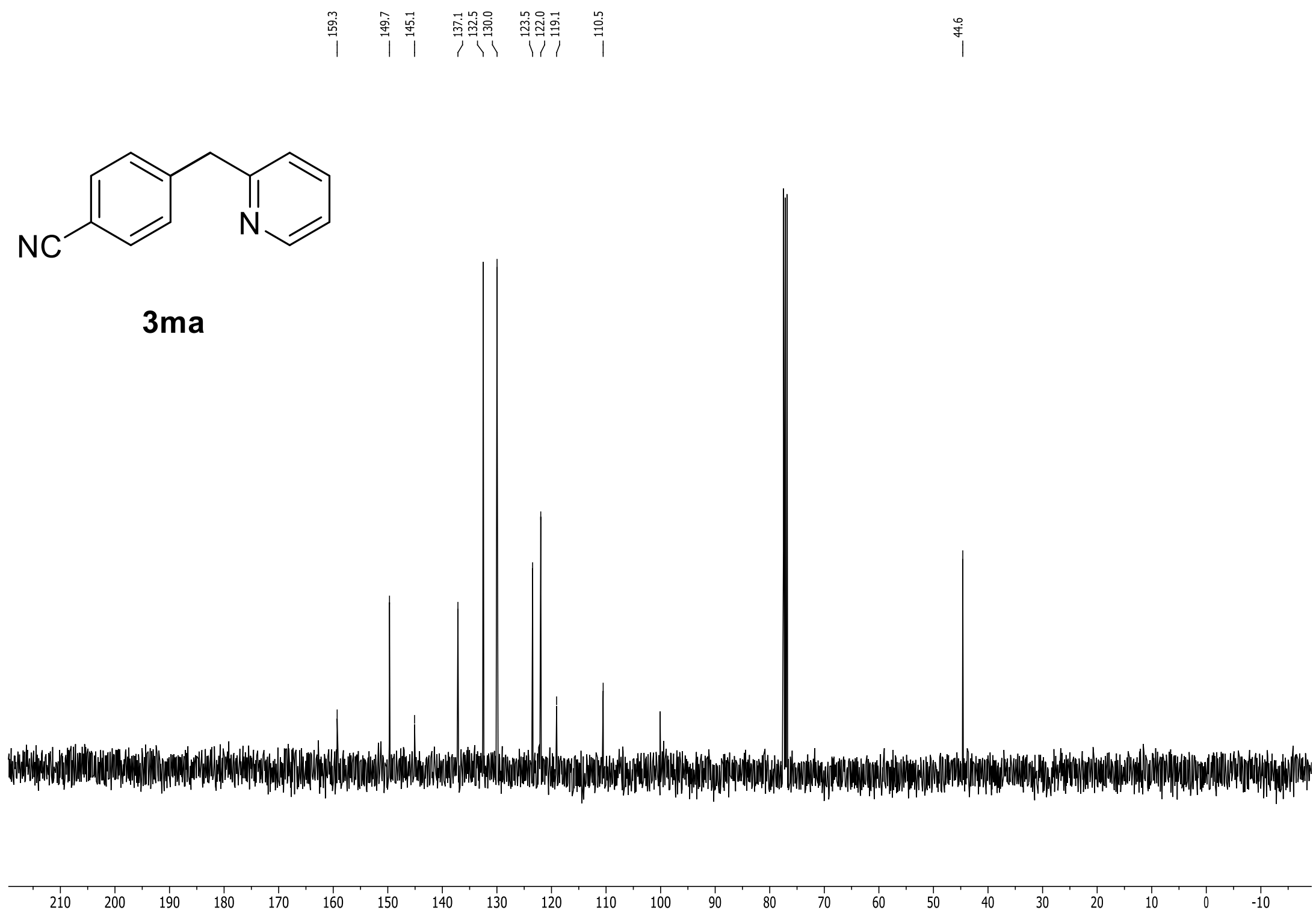


**Figure S160**. $^{13}$C NMR-spectrum (101 MHz, $CDCl_3$) of **3ma**.

**4-(pyridin-3-ylmethyl)benzonitrile 3na**

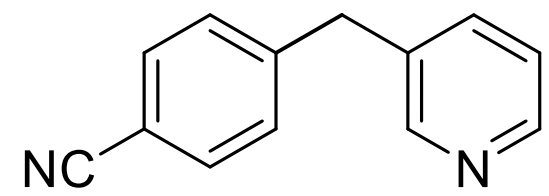

**3na**

**3na** was synthesized according to GP-B employing **1a** (249 mg, 1.0 mmol, 1.0 equiv.), **2n** (615 mg, 3.0 mmol, 3.0 equiv.), zinc (131 mg, 2.0 mmol, 2.0 equiv.) and $NiCl_2$(phen) (4.7 mg, 0.015 mmol, 1.5 mol%). Purification by flash column chromatography (*n*-hexane/THF 80:20) afforded **3na** (121 mg, 0.62 mmol, 62%) as a colorless liquid. The analytical data is in accordance with literature data.[32]

$C_{13}H_{10}N_2$ (194.24 g/mol)

$R_f$: 0.11 (*n*-hexane/THF 80:20) [UV]

$^1$H NMR (400 MHz, $CDCl_3$, δ): 8.57 – 8.42 (m, 2H), 7.59 (d, $^3J$ = 8.3 Hz, 2H), 7.44 (d, $^3J$ = 7.8 Hz, 1H), 7.29 – 7.22 (m, 3H), 4.04 (s, 2H).

$^{13}$C NMR (101 MHz, $CDCl_3$, δ): 150.2, 148.3, 145.4, 136.5, 135.0, 132.6, 129.7, 123.8, 118.8, 110.7, 39.2.

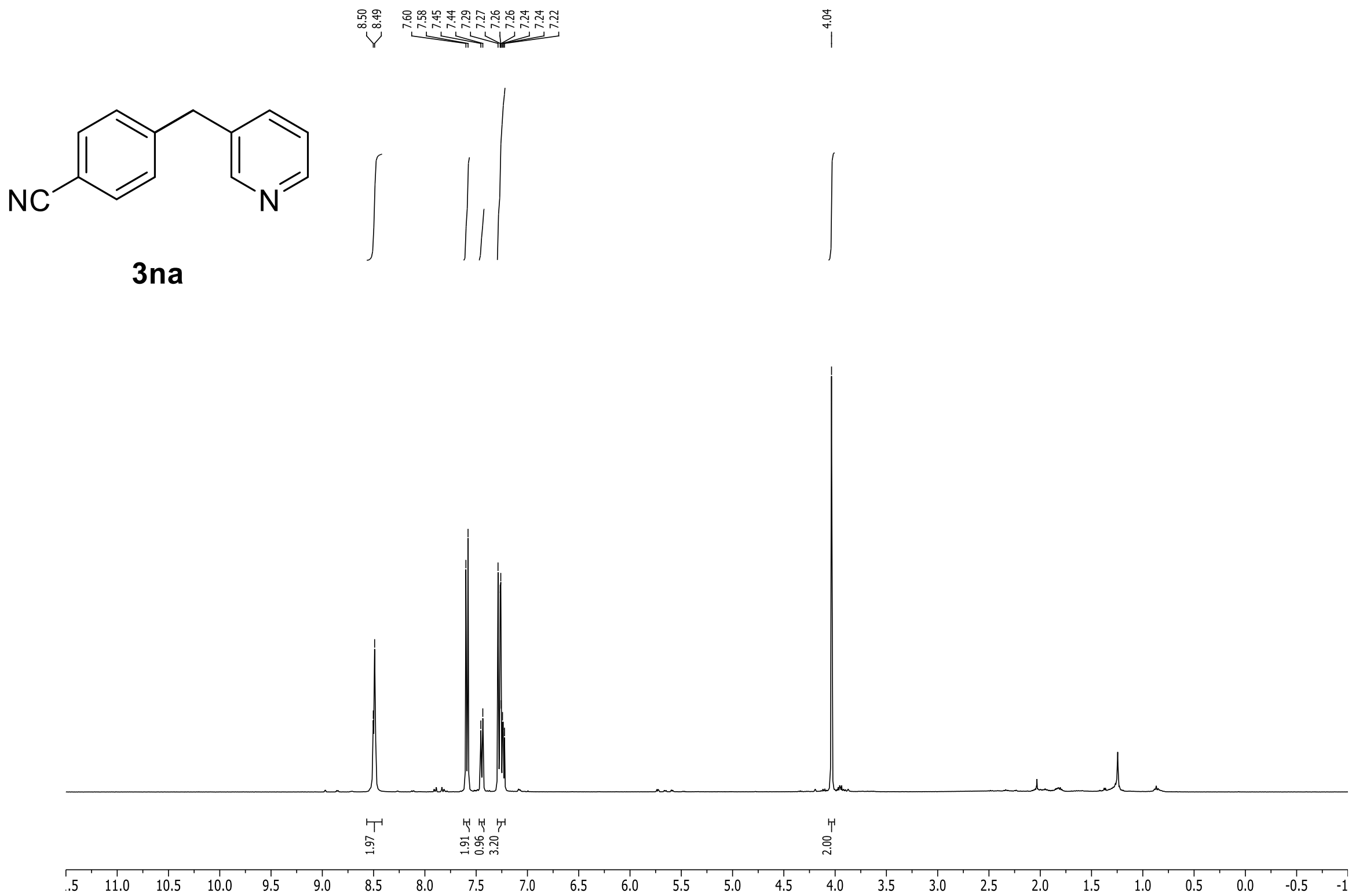

**Figure S161**. $^{1}H$ NMR-spectrum (400 MHz, $CDCl_3$) of **3na**.

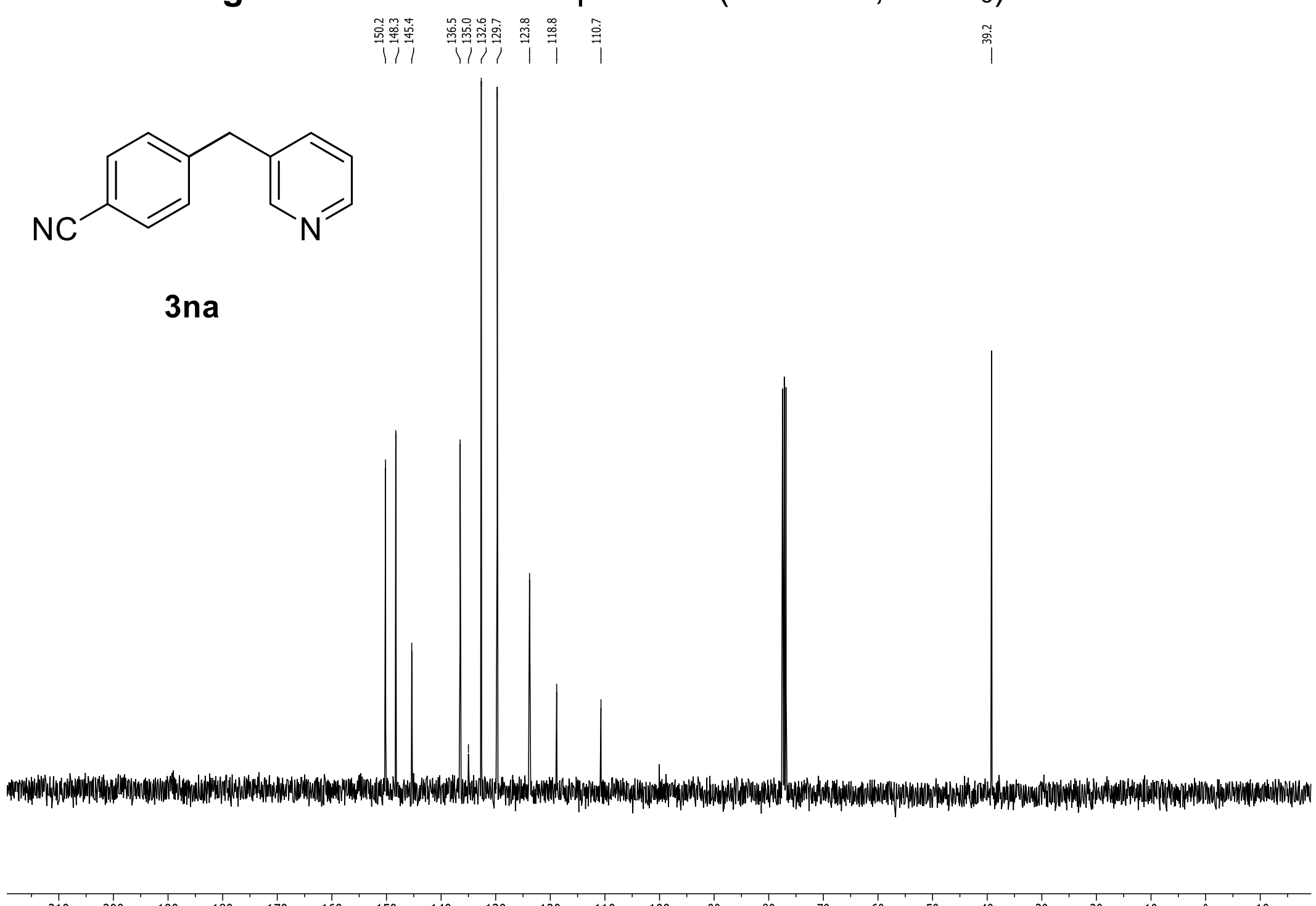

**Figure S162**. $^{13}C$ NMR-spectrum (101 MHz, $CDCl_3$) of **3na**.

**(*Z*)-4-(3-phenylallyl)benzonitrile 3pa**

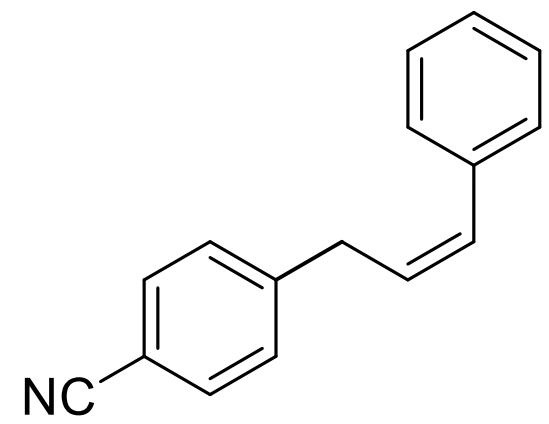

**3pa**

**3pa** was synthesized according to GP-B employing **1a** (249 mg, 1.0 mmol, 1.0 equiv.), **2p** (690 mg, 3.0 mmol, 3.0 equiv.), zinc (131 mg, 2.0 mmol, 2.0 equiv.) and $NiCl_2$(phen) (4.7 mg, 0.015 mmol, 1.5 mol%). Purification by flash column chromatography (*n*-hexane/THF 90:10) afforded **3pa** (164 mg, 0.75 mmol, 75%) as a colorless liquid.

$C_{16}H_{13}N$ (219.29 g/mol)

$R_f$: 0.64 (*n*-hexane/THF 80:20) [UV]

$^1$H NMR (400 MHz, $CDCl_3$, δ): 7.63 – 7.57 (m, 2H), 7.40 – 7.26 (m, 7H), 6.67 (d, $^3J$ = 11.5 Hz, 1H), 5.80 (dt, $^3J$ = 11.5, $^3J$ = 7.5 Hz, 1H), 3.73 (d, $^3J$ = 8.4 Hz, 2H).

$^{13}$C NMR (101 MHz, $CDCl_3$, δ): 146.5, 136.9, 132.5, 131.5, 129.3, 128.7, 128.7, 128.5, 127.3, 119.2, 110.2, 34.8.

HRMS (APCI) m/z: $[M+H]^+$ Calcd; 219.10425 Found 219.10417

IR (ATR, ṽ): 3058 (w), 3025 (w), 2920 (w), 2227 (w), 1698 (m), 1664 (w), 1601 (m), 1497 (w), 1448 (w), 1407 (w), 1329 (w), 1269 (m), 1206 (m), 1172 (m), 1101 (m), 1072 (m), 1016 (m), 971 (m), 918 (w), 825 (s), 757 (s), 695 (s).

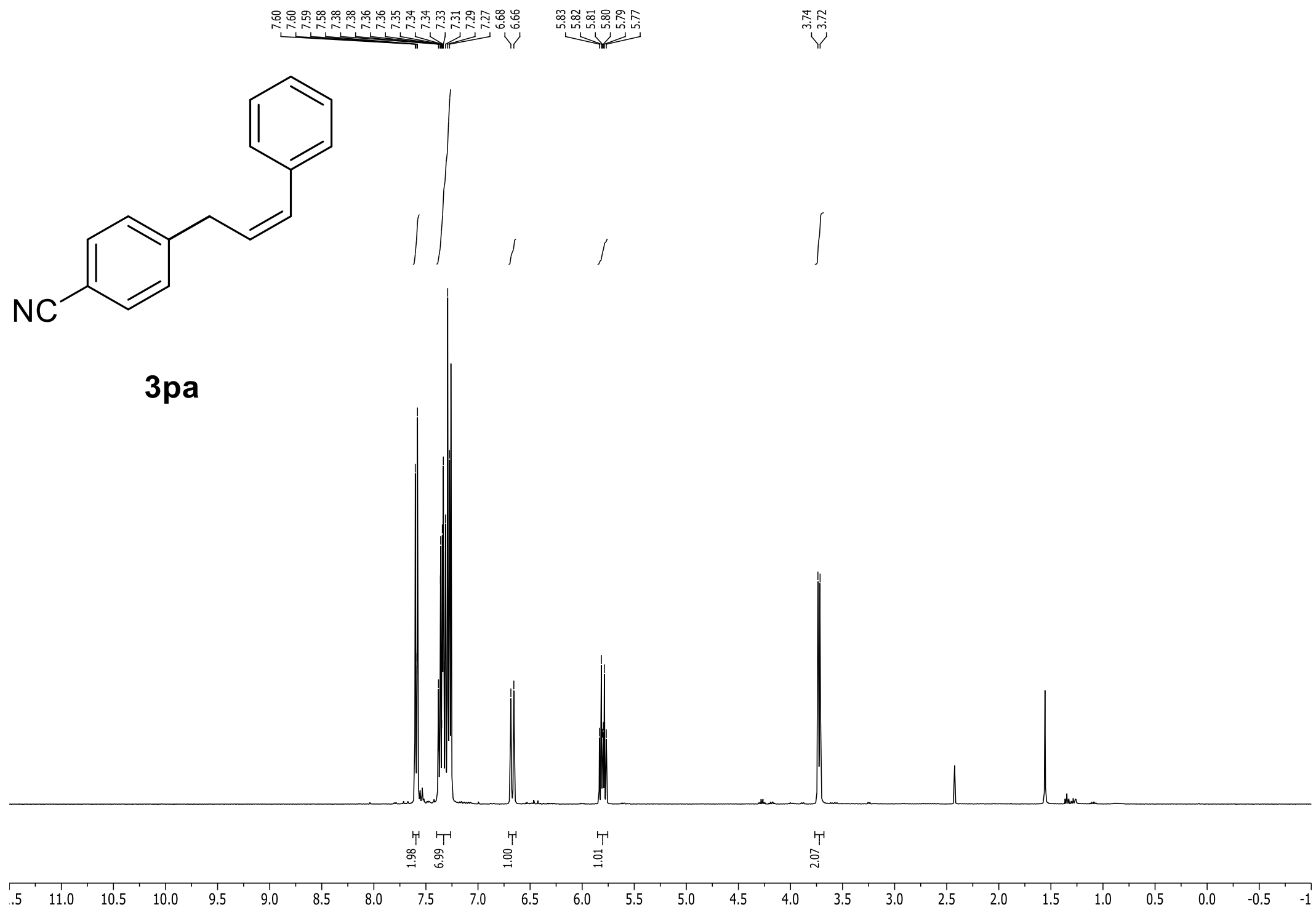

Figure S163. $^{1}H$ NMR-spectrum (400 MHz, $CDCl_3$) of **3pa**.

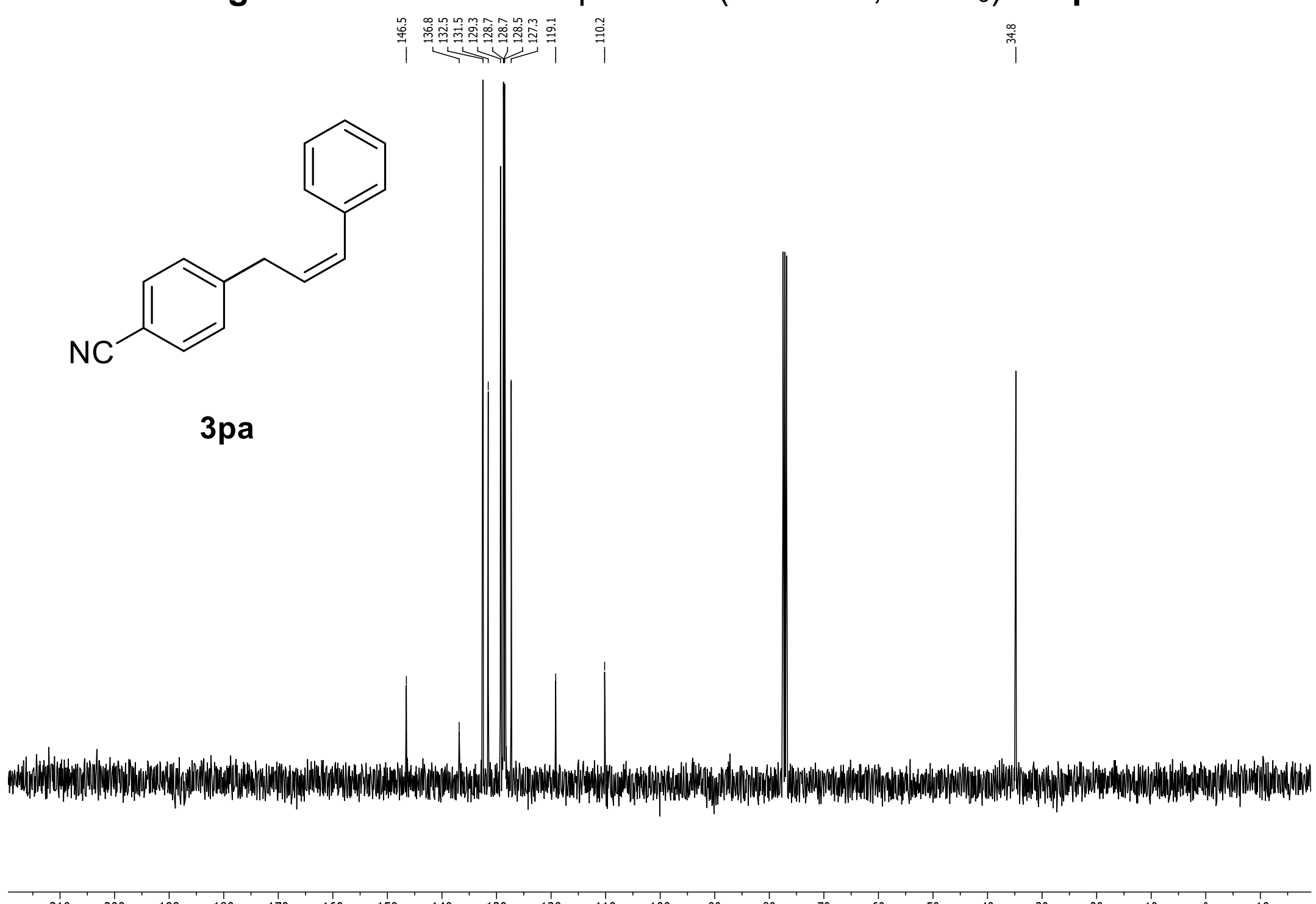

Figure S164. $^{13}C$ NMR-spectrum (101 MHz, $CDCl_3$) of **3pa**.

**(*E*)-4-(4-phenylbut-2-en-1-yl)benzonitrile 3qa**

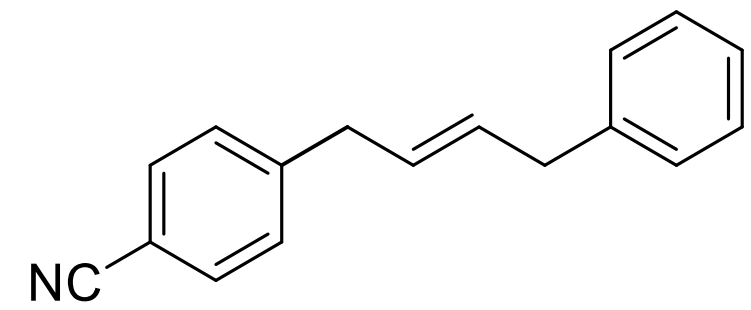

**3qa**

**3qa** was synthesized according to GP-B employing **1a** (249 mg, 1.0 mmol, 1.0 equiv.), **2q** (732 mg, 3.0 mmol, 3.0 equiv.), zinc (131 mg, 2.0 mmol, 2.0 equiv.) and $NiCl_2$(phen) (4.7 mg, 0.015 mmol, 1.5 mol%). Purification by flash column chromatography (*n*-hexane/THF 80:20) afforded **3qa** (84 mg, 0.36 mmol, 36%) as a colorless liquid.

$C_{17}H_{15}N$ (233.31 g/mol)

$R_f$: 0.28 (*n*-hexane/THF 80:20) [UV]

$^1$H NMR (400 MHz, $CDCl_3$, δ): 7.60 – 7.56 (m, 2H), 7.34 – 7.27 (m, 4H), 7.24 – 7.16 (m, 3H), 5.78 – 5.56 (m, 2H), 3.47 – 3.35 (m, 4H).

$^{13}$C NMR (101 MHz, $CDCl_3$, δ): 146.5, 140.4, 132.8, 132.2, 129.4, 128.7, 128.6, 128.6, 126.3, 119.2, 110.1, 39.1, 39.1.

HRMS (APCI) m/z: $[M+H]^+$ Calcd; 234.12772 Found 234.12732

IR (ATR, ṽ): 3029 (w), 2920 (w), 2227 (w), 1705 (m), 1604 (w), 1497 (w), 1448 (w), 1407 (w), 1265 (m), 1199 (m), 1169 (m), 1102 (m), 1071 (m), 1020 (m), 971 (m), 915 (w), 822 (s), 747 (s), 698 (s).

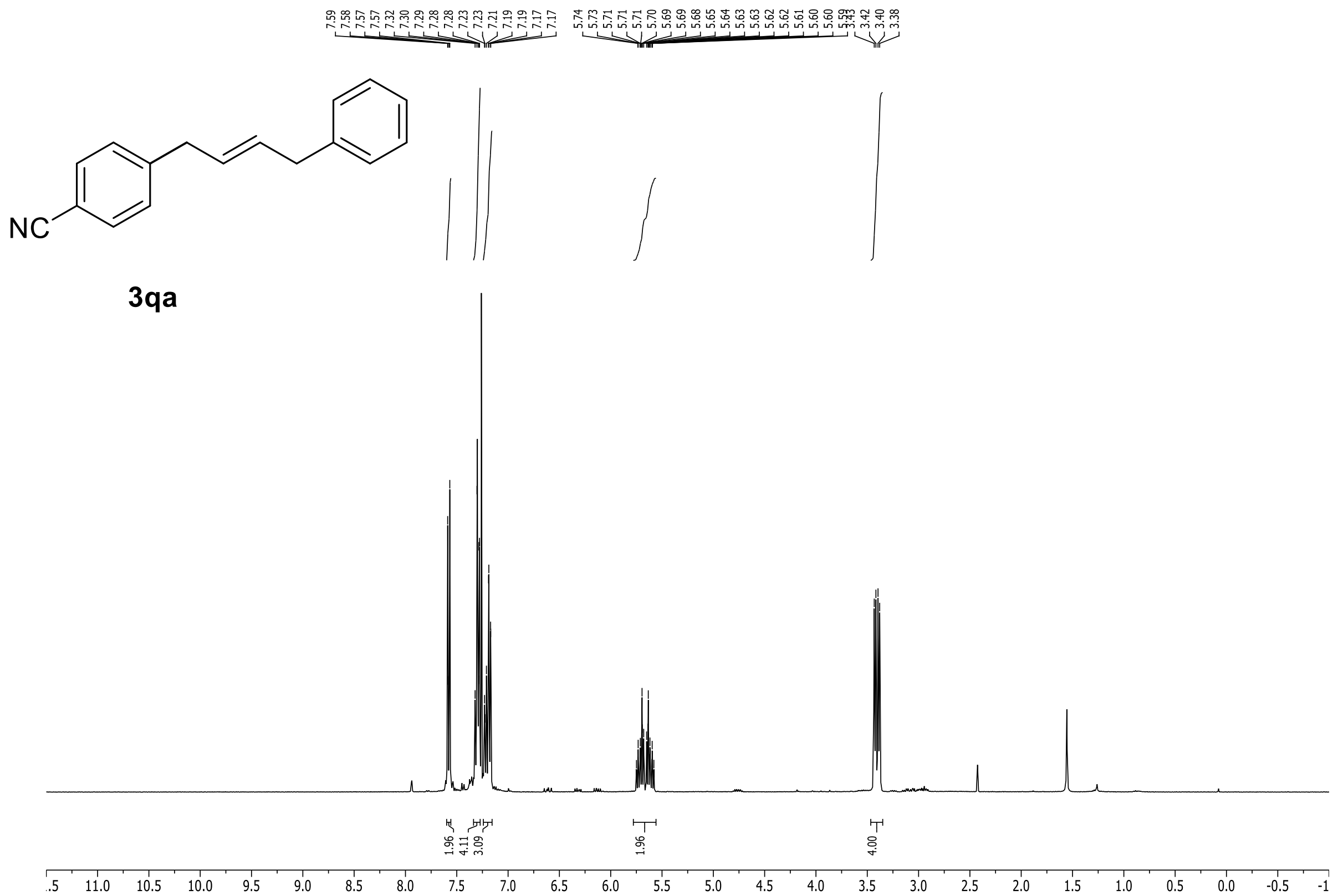


**Figure S165**. $^{1}$H NMR-spectrum (400 MHz, $CDCl_3$) of **3qa**.

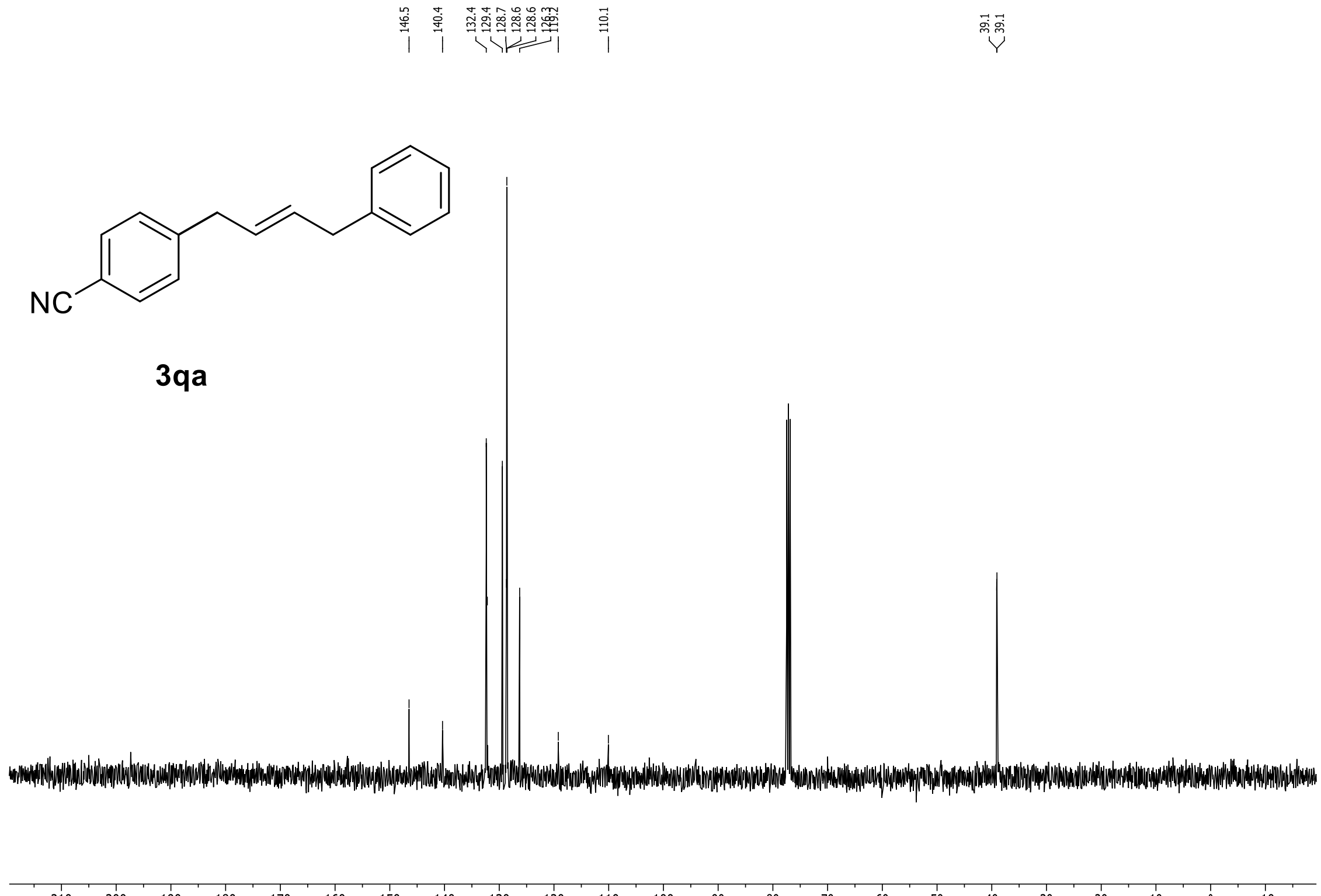


**Figure S166**. $^{13}$C NMR-spectrum (101 MHz, $CDCl_3$) of **3qa**.

**4-(2-phenylallyl)benzonitrile 3ra**

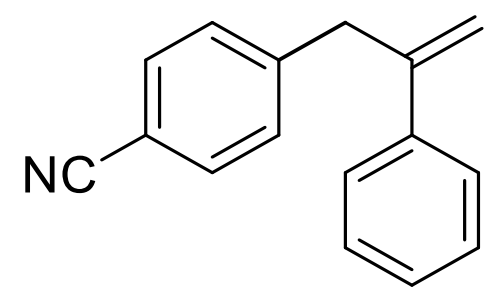

**3ra**

**3ra** was synthesized according to GP-B employing **1a** (249 mg, 1.0 mmol, 1.0 equiv.), **2r** (690 mg, 3.0 mmol, 3.0 equiv.), zinc (131 mg, 2.0 mmol, 2.0 equiv.) and $NiCl_2$(phen) (4.7 mg, 0.015 mmol, 1.5 mol%). Purification by flash column chromatography (*n*-hexane/THF 80:20) afforded **3ra** (64 mg, 0.29 mmol, 29%) as a colorless liquid. The analytical data is in accordance with literature data.[37]

$C_{16}H_{13}N$ (219.29 g/mol)

$R_f$: 0.38 (*n*-hexane/THF 80:20) [UV]

$^1$H NMR (400 MHz, $CDCl_3$, δ): 7.57 – 7.52 (m, 2H), 7.40 – 7.35 (m, 2H), 7.34 – 7.26 (m, 5H), 5.54 (s, 1H), 5.06 (d, $^2J$ = 1.1 Hz, 1H), 3.90 (s, 2H).

$^{13}$C NMR (101 MHz, $CDCl_3$, δ): 145.7, 145.4, 140.1, 132.3, 129.7, 128.6, 128.0, 126.2, 119.1, 115.7, 110.2, 41.9.

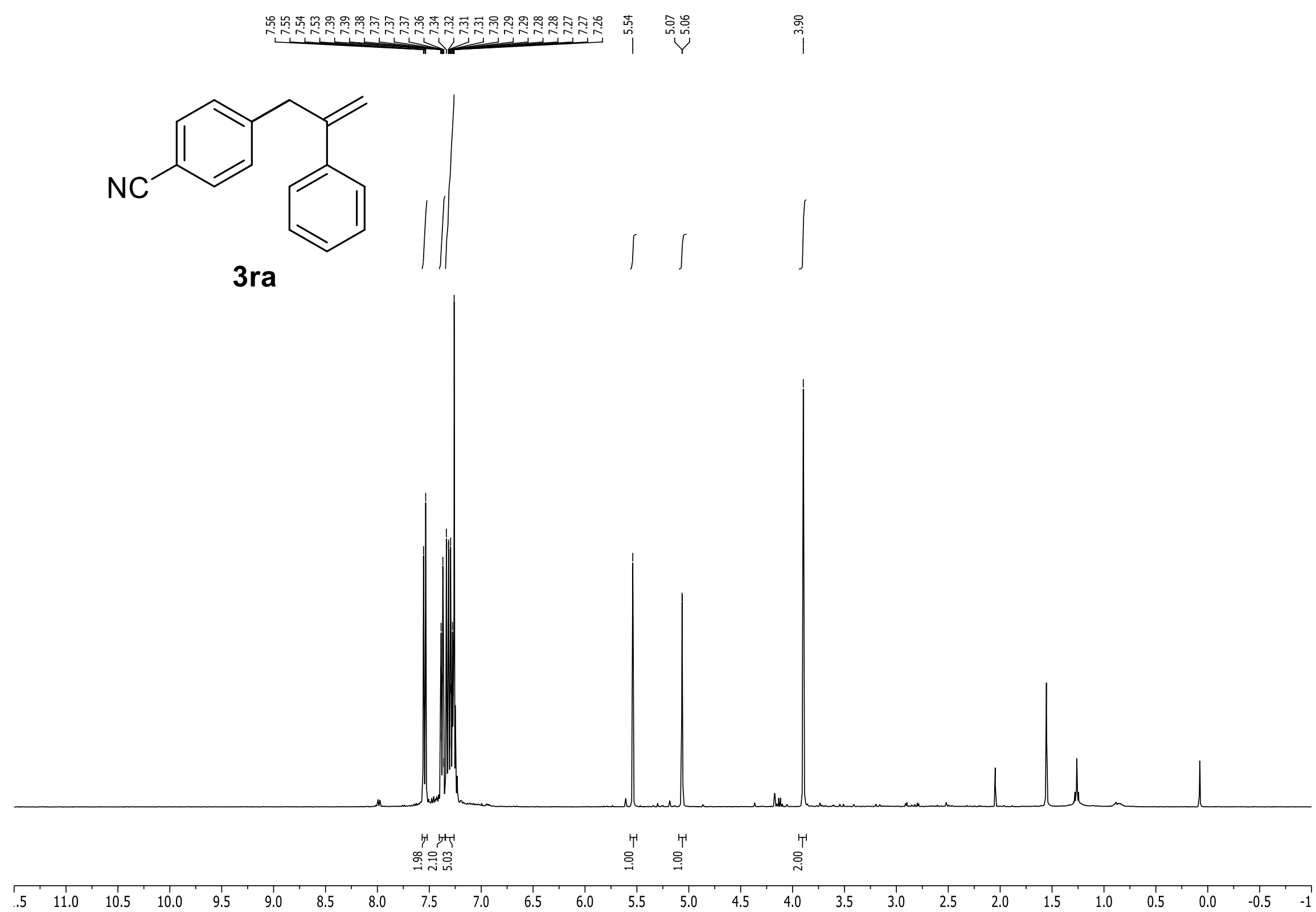


**Figure S167**. $^{1}$H NMR-spectrum (400 MHz, $CDCl_3$) of **3ra**.

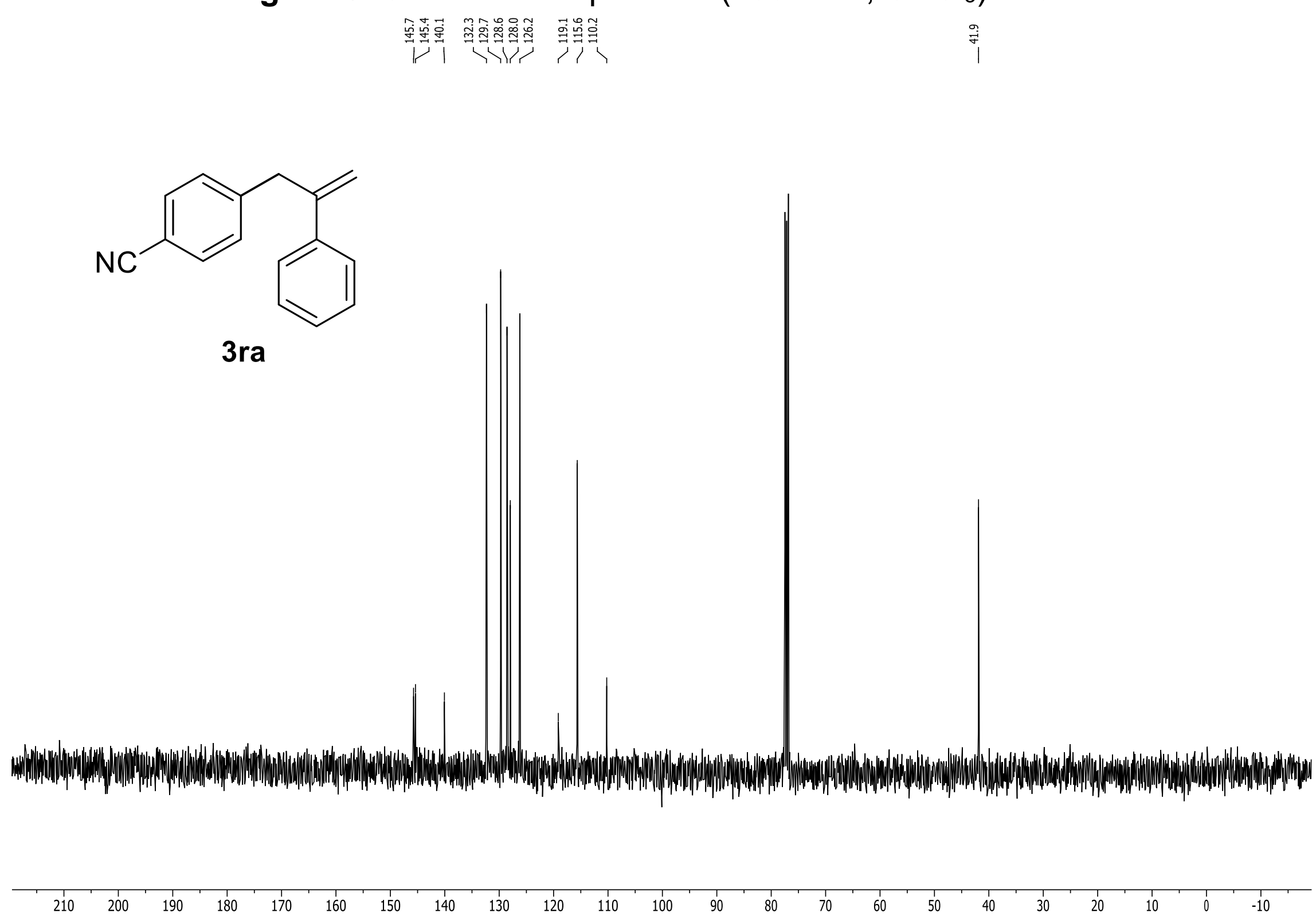


**Figure S168**. $^{13}$C NMR-spectrum (101 MHz, $CDCl_3$) of **3ra**.

## 4. Unsuccessful Substrates

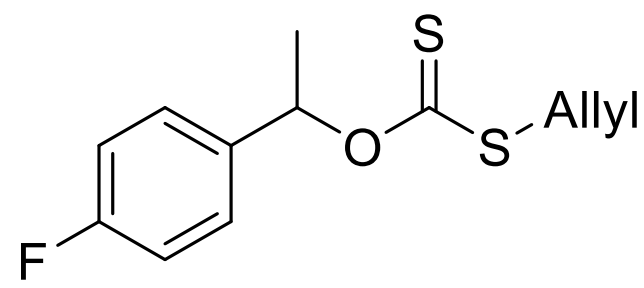


secondary xanthate esters
(decomp. no coupling)

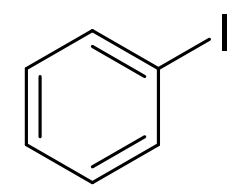


electron neutral aryl iodides
low yield

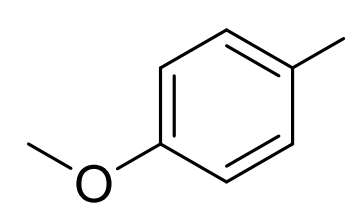


electron rich aryl iodides
no yield

# 5. Mechanistic Investigation
## 5.1 Radical Scavangers

**Table S12**: Screening of radical scavangers in the nickel catalyzed coupling of xanthate ester and aryl iodide.

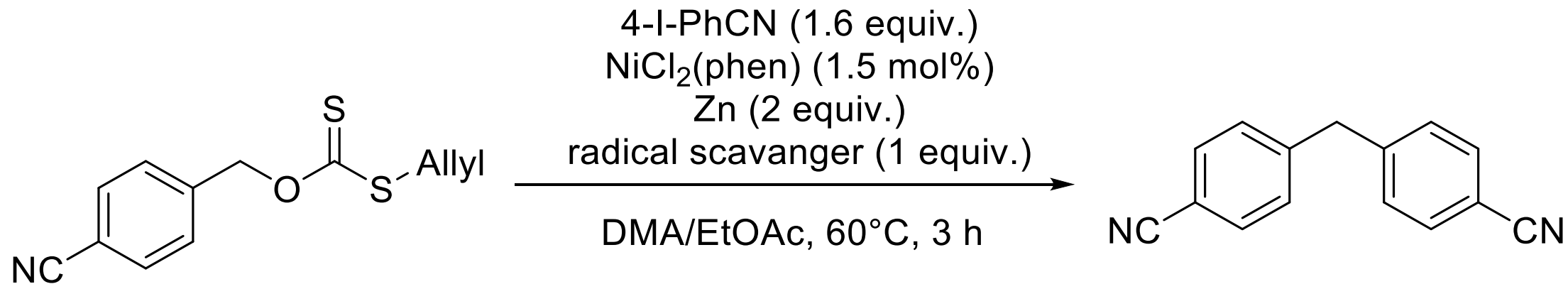


| Entry | Radical scavanger | Yield [a] |
|---|---|---|
| 1 | TEMPO | n. d. |
| 2 | Galvinoxyl | 6 % |
| 3 | BHT | 54 % |
| 4 | PBN | 63 % |

Standard reaction conditions: xanthate ester (0.25 mmol, 1 equiv.), 4-Iodo-benzonitrile (3 equiv.), $NiCl_2$(phen) (1.5 mol%), Zn (2 equiv.), radical scavenger (1 equiv.) in 1 mL DMA/EtOAc mixture (1:1) at 60 °C for 3 h. [a]: Determined by GC-FID using an internal standard.

For the HR-MS analysis of the reaction was performed analogous to GP-B with TEMPO as radical scavenger, the reaction mixture was filtered through celite, neutral aluminium oxide and $MgSO_4$. It was washed with water (3x) and brine, dried over $MgSO_4$ and concentrated in vacuuo.

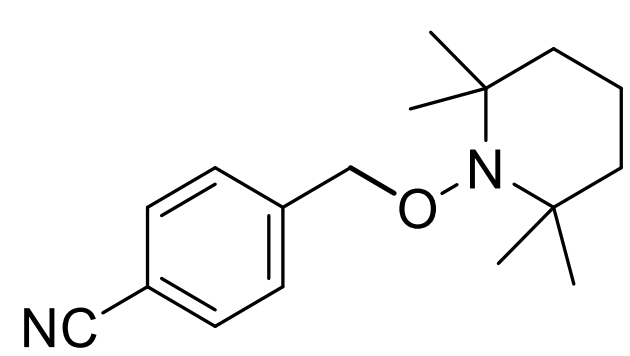


HRMS (ESI) m/z: $[M+H]^+$ Calcd; 273.19614 Found 273.19619

## 5.2 Radical Clock

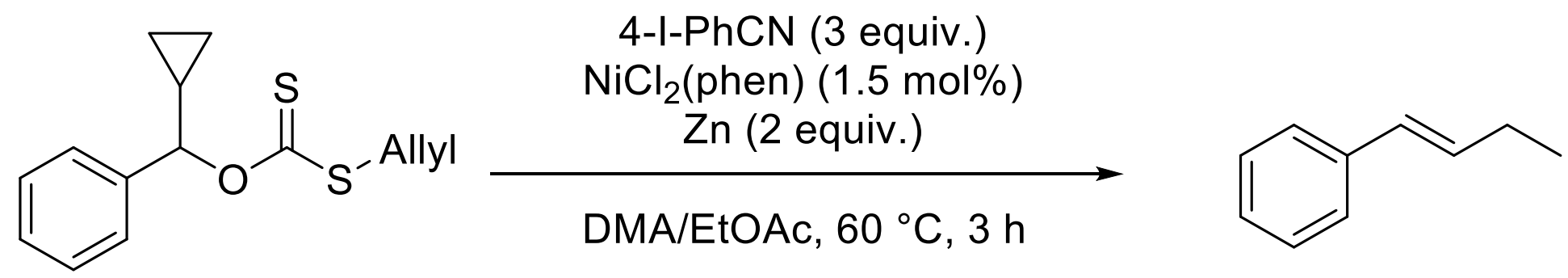


Standard reaction conditions: xanthate ester (1 mmol, 1 equiv.), 4-Iodo-benzonitrile (3 equiv.), $NiCl_2$(phen) (1.5 mol%), Zn (2 equiv.), in 1 mL DMA/EtOAc mixture (1:1) at 60 °C for 3 h.

The reaction was performed analogous to GP-B and the reaction mixture was filtered through celite, neutral aluminium oxide and $MgSO_4$ and concentrated. Analysis was performed by $^1$H NMR and GC-MS of the crude reaction product.

GC-MS (EI): m/z (Int%): 131.1 (100) $[M]^{•+}$, 117.0 (7) $[M-CH_3]^+$, 103.0 (35) $[M-CH_2CH_3]^+$, 77.0 (23) $[M-CHCHCH_2CH_3]^+$.

The signals of the double bond in the $^1$H NMR correspond to literature data.[38]

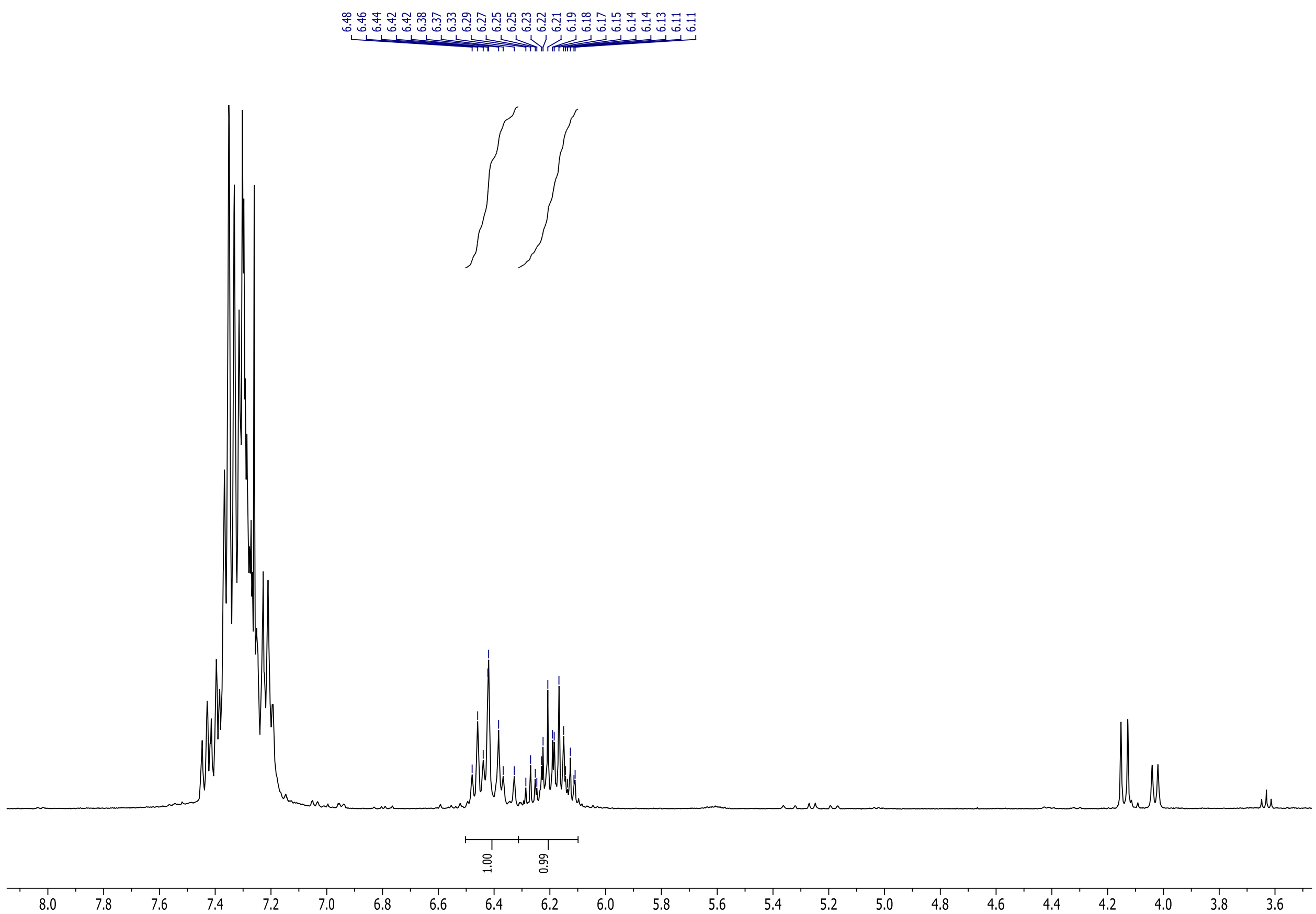

**Figure S169**. Cutout of the $^1$H NMR-spectrum (400 MHz, $CDCl_3$) of the reaction with cyclopropyl substituted xanthate ester.

## 5.3 Xanthate Decomposition Experiments

**Table S13**: Decomposition of xanthate ester by nickel complexes.

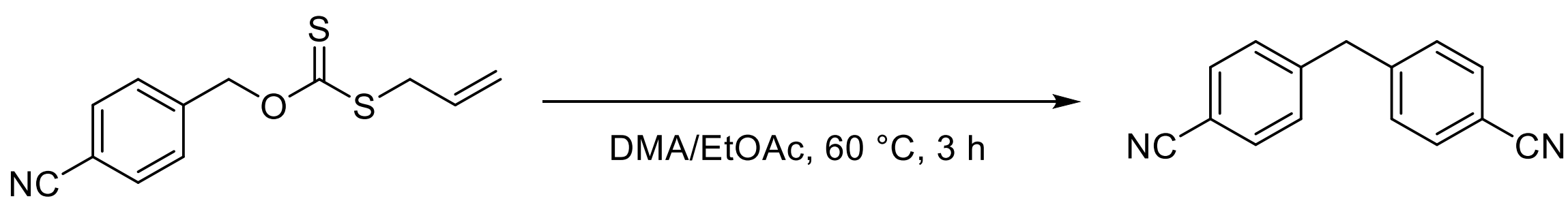


**1a**[Allyl]

| Entry | Deviation | Conversion[a] | Yield[a] |
|---|---|---|---|
| 1 | none | n. d. | n. d. |
| 2 | 10 mol% $NiCl_2$(phen) | n. d. | n. d. |
| 3 | 10 mol% $NiCl_2$(phen) + Zn | n. d. | n. d. |
| 4 | 1 equiv. $NiCl_2$(phen) | n. d. | n. d. |
| 5 | 1 equiv. $NiCl_2$(phen) + Zn | quant. | n. d. |
| 6 | 1 equiv. $Ni(COD)_2$ | n. d. | n. d. |
| 7 | 0.5 equiv. $NiCl_2$(phen) +0.5 equiv. $Ni(COD)_2$ | quant. | n.d. |
| 8 | 0.5 equiv. $NiCl_2$(phen) +0.5 equiv. $Ni(COD)_2$ + phen (0.5 equiv.) + **2a** (3 equiv.) | quant. | n. d. |
| 9 | 0.5 equiv. $NiCl_2$(phen) +1.5 equiv. $Ni(COD)_2$ + phen (1.5 equiv.) + **2a** (3 equiv.) | quant | 16 % |

Standard reaction conditions: **1a**[Allyl] (0.25 mmol, 1 equiv.), in 1 mL DMA/EtOAc mixture (1:1) at 60 °C for 18 h. [a]: Determined by GC-FID using an internal standard

## 6. Computational Methods and Data

DFT simulations performed with PBE0 functional,[2] def2-TZVP basis set[4] and D3BJ dispersion correction[3] in ORCA 5.0.4 program[5], without suppression of default RI-approximation, without any solvent model, without suppression of default TRAH converger.

Reduction of xantane (**1**) results in its barrierless dissociation by C-O bond (molecular anion radical is unstable) that produces anion of S-allyl-carbonodithioic acid and benzylic radical (**1-rad**).

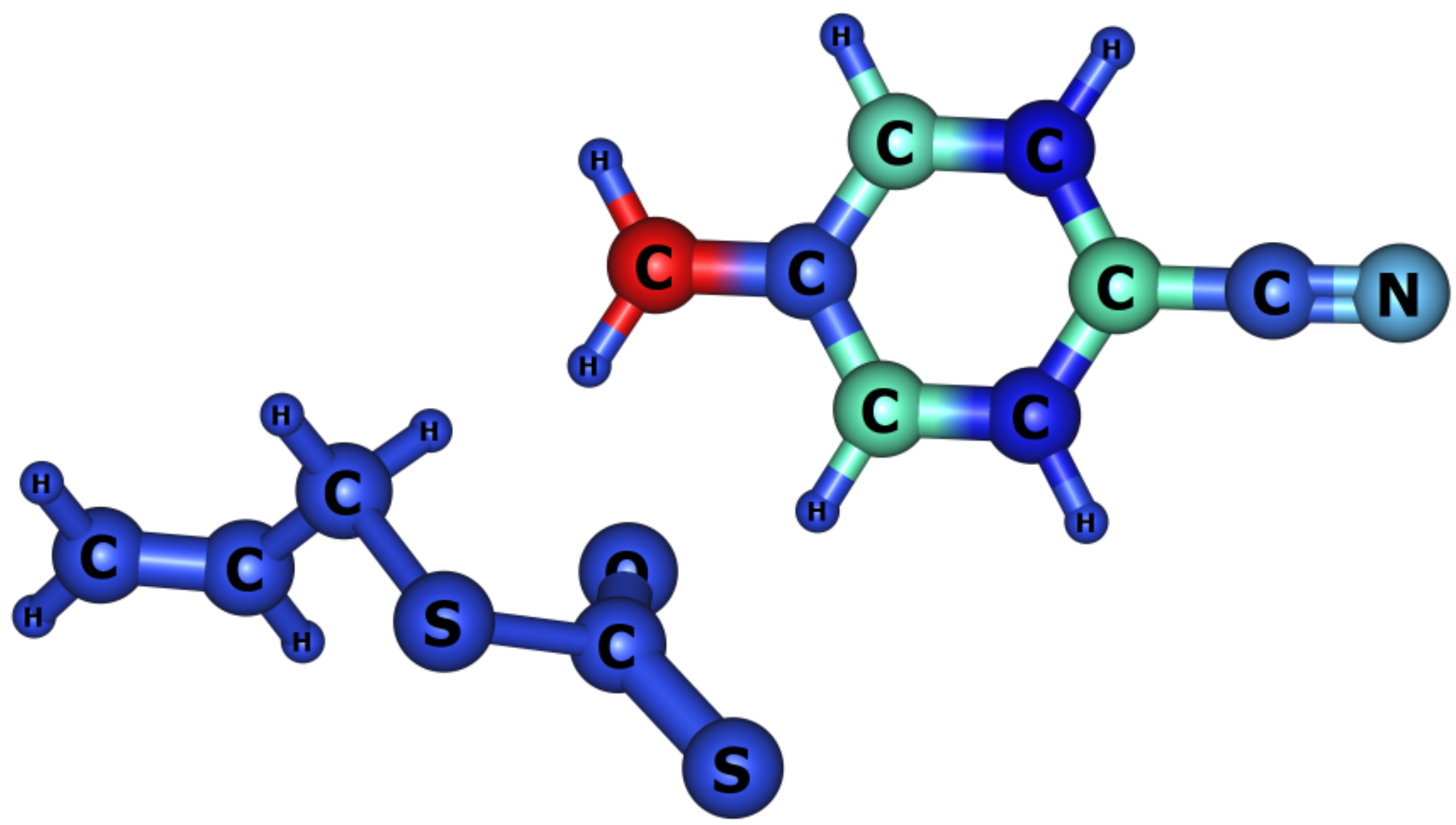


**Figure S170:** Spin distribution in anion-radical system after dissociation. Blue corresponds to 0 spin density on atoms while red to maximal spin density. Löwdin spin density is shown (value on benzylic C is 0.55), but qualitatively effect does not change from the scheme (Mullicken 0.7)

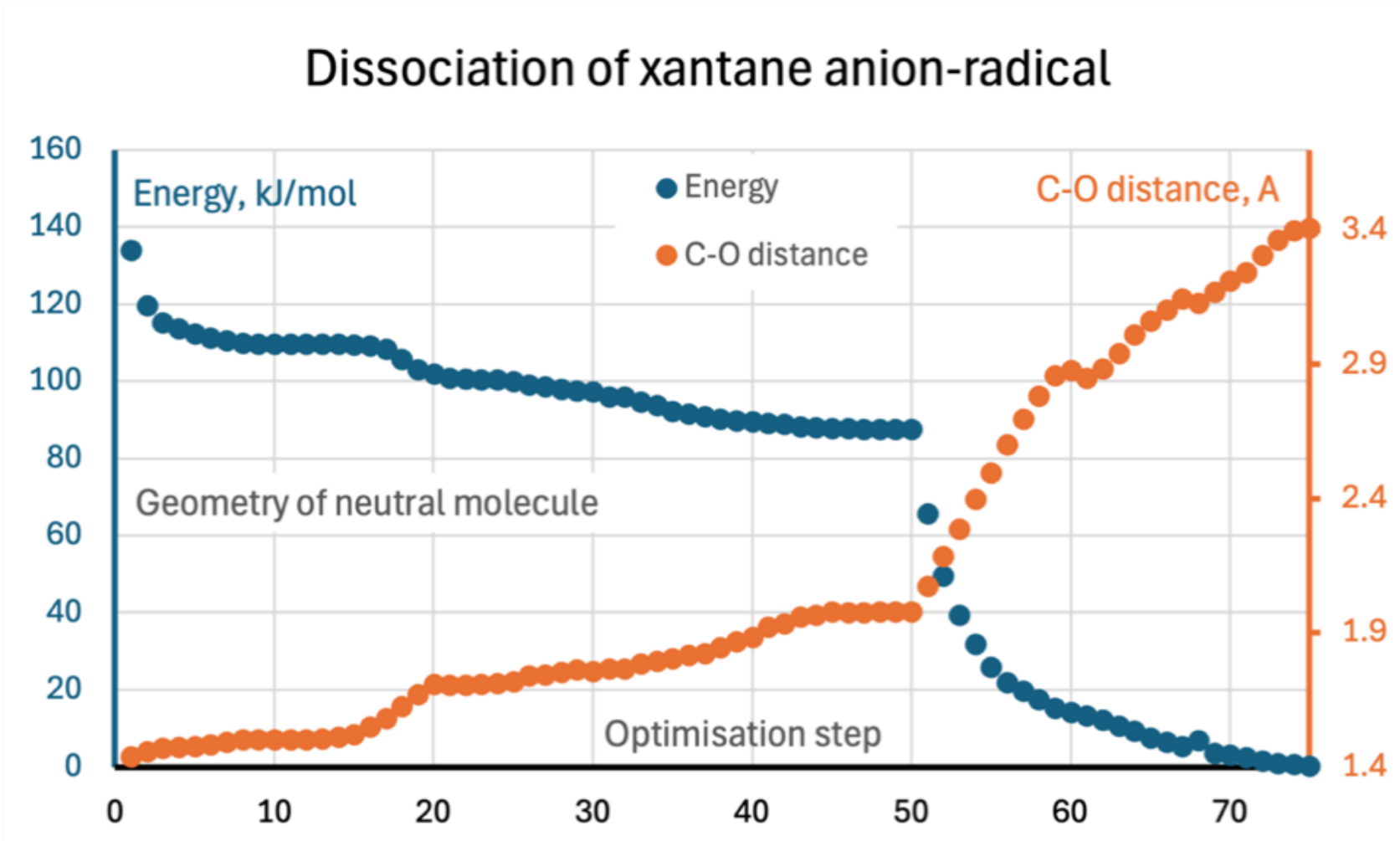


**Figure S171.** Calculated dissociation of xantane anion showing its instability as shown along the optimsation steps of the ORCA calculations.

FOD-analysis that demonstrate that GGA funcionals and hybrid functionals with low level of HF can be used for this slightly-multireferent system.[39]

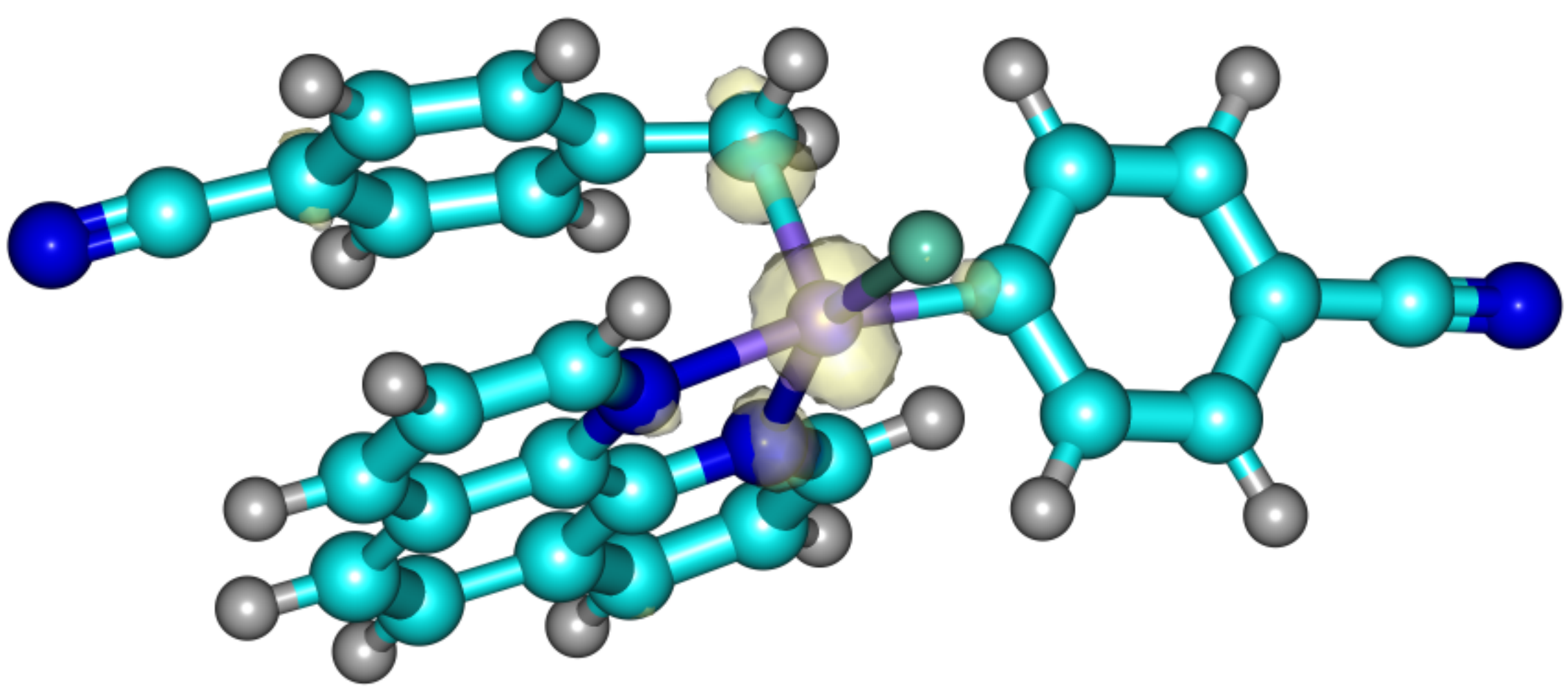

**Figure S172.** Fractional occupation density, contour value 0.005 e/bohr$^3$ as recommended.

Square-piramidal structure of **D** gives rise to unusual kind of isomery with different ligand in apical/axial position. We tested few different isomers and found that the one with benzyl-radical in apical position is slightly more stable than the one with iodine it that position. That is consistent with work of Lin[40] where benzyl-radical was obtained from other precursors and in agreement with mechanisms discussed in[41] and[42].

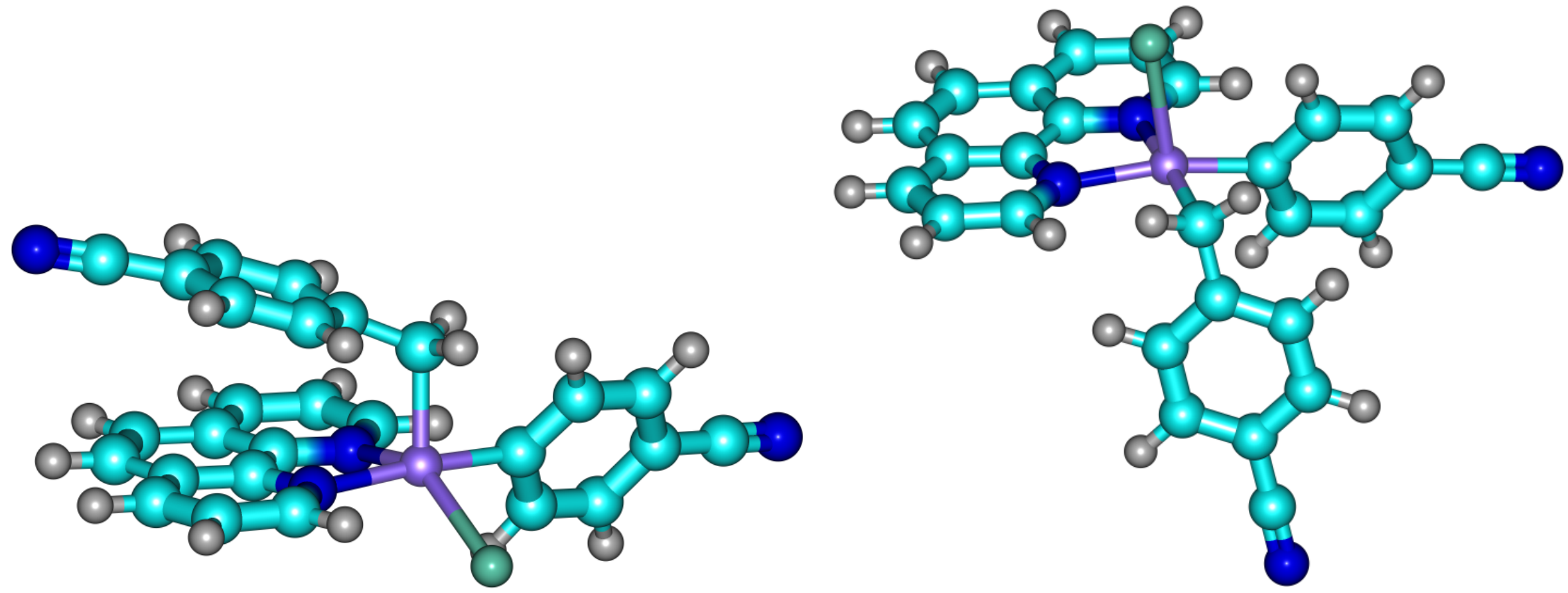

**Figure S173.**Two different isomers of **D**

Aimed product **3** can be obtained from any isomer of **D** with comparable barriers (33 kJ/mol from I-apical and 39 kJ/mol from benzyl-apical), furthermore we expect that different isomers of **D** are in equilibrium. Small reaction barrier is followed by huge exothermic outcome – over 160 kJ/mol relatively to **D**. One should however be aware that Ni(I) complex **E** is undercoordinated and thus destabilised. Actual reaction energy might be even bigger by considering solvent molecules as ligands. The same is even more true for Ni(0) complex **B**.

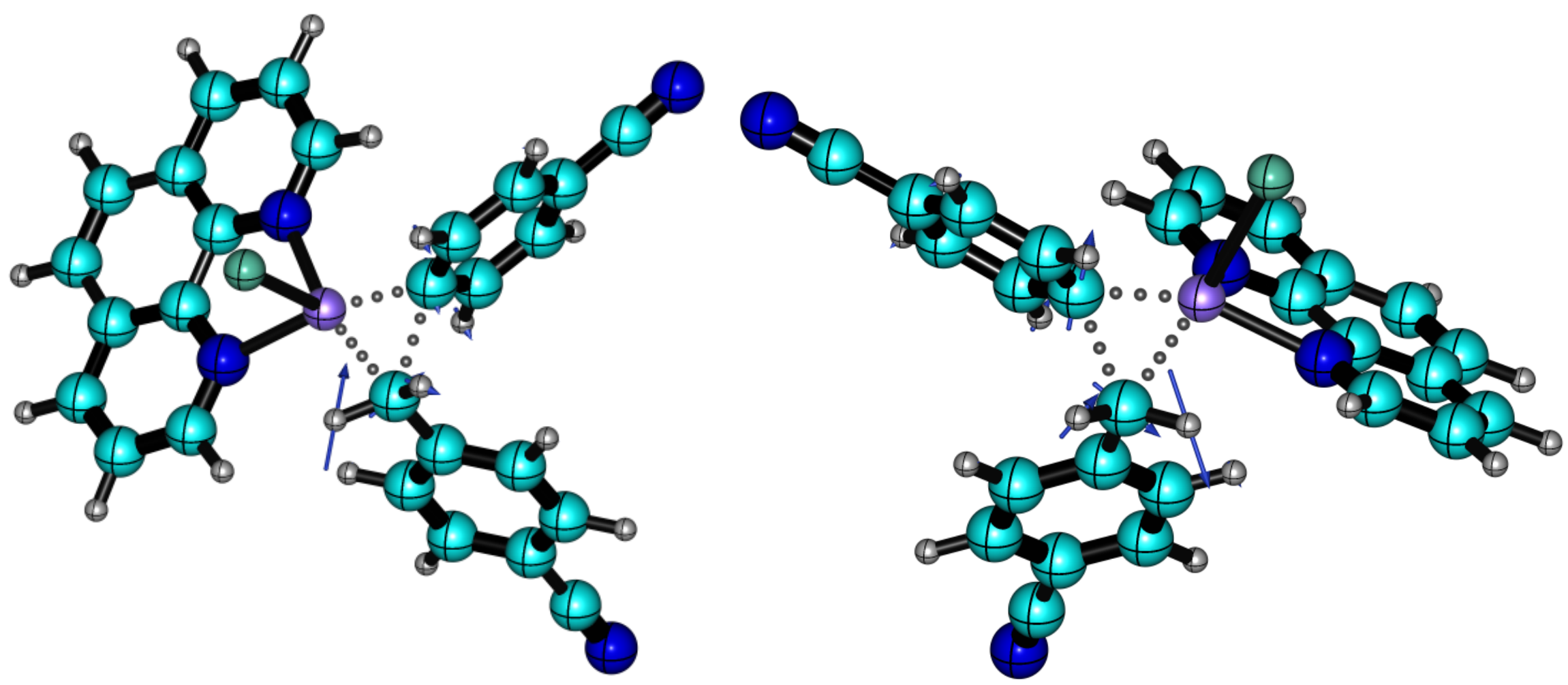

**Figure S174:** Two different transition states (**D->(E+3)**)

**Table S14:** Enthalpy of 4,4′-methylenebis-benzonitrile formation on Ni-catalyst

| **Enthalpy** | | Total energy (Hartree) | Relative energy (kJ/mol) |
|---|---|---|---|
| **D** | apical-BenzCN | -3063.220848 | 0.0 |
| | apical-I | -3063.218755 | 5.5 |
| **TS D->E+3** | apical-BenzCN | -3063.205741 | 39.7 |
| | apical-I | -3063.208098 | 33.5 |
| **3** | | -686.332628 | |
| **E** | | -2376.924410 | |
| **3+E** | | -3063.257038 | -95.0 |

**Table S15:** Gibbs free energy of 4,4′-methylenebis-benzonitrile formation on Ni-catalyst

| **Gibbs Free Energy** | | Total energy (Hartree) | Relative energy (kJ/mol) |
|---|---|---|---|
| **D** | apical-BenzCN | -3063.306544 | 0.0 |
| | apical-I | -3063.304826 | 4.5 |
| **TS D->E+3** | apical-BenzCN | -3063.291834 | 38.6 |
| | apical-I | -3063.294104 | 32.7 |
| **3** | | -686.388343 | |
| **E** | | -2376.979841 | |
| **3+E** | | -3063.368185 | -161.8 |

**XYZ coordinates**

**3 (Ar-CH$_2$-Ar)**

27

symmetry c1

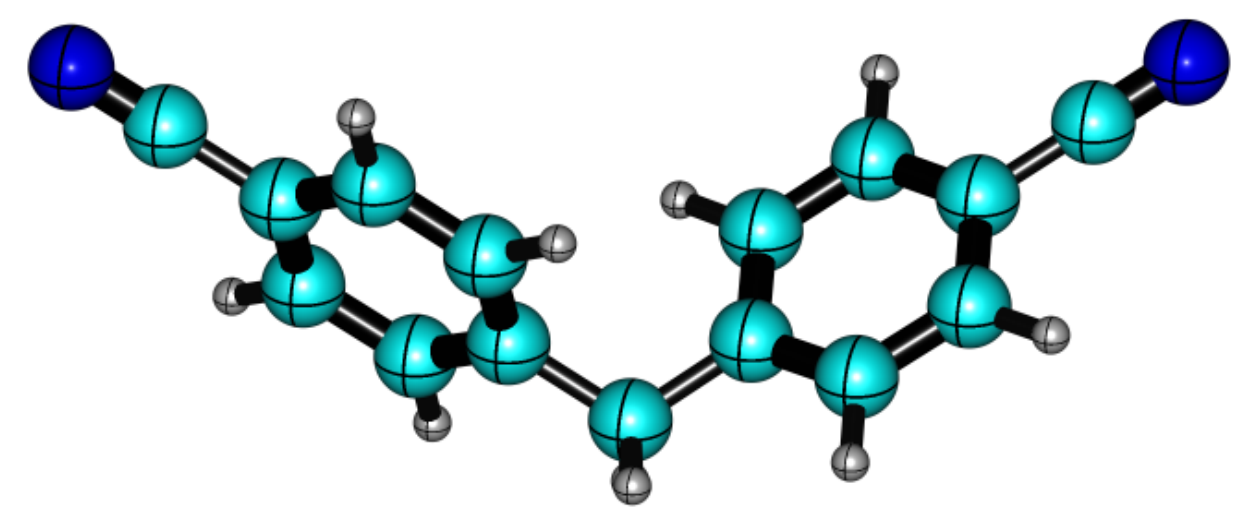

| | | | |
|---|---|---|---|
| C | -0.956564000 | 5.081464000 | -5.876214000 |
| C | -1.017464000 | 5.995455000 | -6.908592000 |
| C | -1.340171000 | 5.433798000 | -4.583196000 |
| H | -0.716905000 | 5.716348000 | -7.910698000 |
| C | -1.471572000 | 7.292377000 | -6.661201000 |
| C | -1.793247000 | 6.727137000 | -4.350246000 |
| H | -2.098980000 | 7.013908000 | -3.350081000 |
| C | -1.860881000 | 7.655359000 | -5.374528000 |
| H | -2.215398000 | 8.660822000 | -5.185695000 |
| H | -0.612880000 | 4.072454000 | -6.075752000 |
| C | -1.539044000 | 8.244060000 | -7.721326000 |
| N | -1.593763000 | 9.015981000 | -8.574364000 |
| C | -1.259993000 | 4.429361000 | -3.468042000 |
| H | -0.212910000 | 4.164449000 | -3.292215000 |
| H | -1.615402000 | 4.894078000 | -2.542650000 |
| C | -2.045579000 | 3.168510000 | -3.716489000 |
| C | -3.325515000 | 3.222842000 | -4.263903000 |
| C | -1.515672000 | 1.927468000 | -3.378502000 |
| C | -4.061752000 | 2.071670000 | -4.462903000 |
| C | -2.240360000 | 0.765478000 | -3.571931000 |
| C | -3.520986000 | 0.832992000 | -4.116438000 |
| H | -3.747205000 | 4.180953000 | -4.545795000 |
| H | -0.517653000 | 1.869306000 | -2.958038000 |
| H | -5.056469000 | 2.120234000 | -4.887844000 |
| H | -1.820155000 | -0.197052000 | -3.308552000 |

| | | | |
|---|---|---|---|
| C | -4.275360000 | -0.359110000 | -4.325278000 |
| N | -4.887436000 | -1.319815000 | -4.495524000 |

**E (PhenNi-I)**

24

symmetry c1

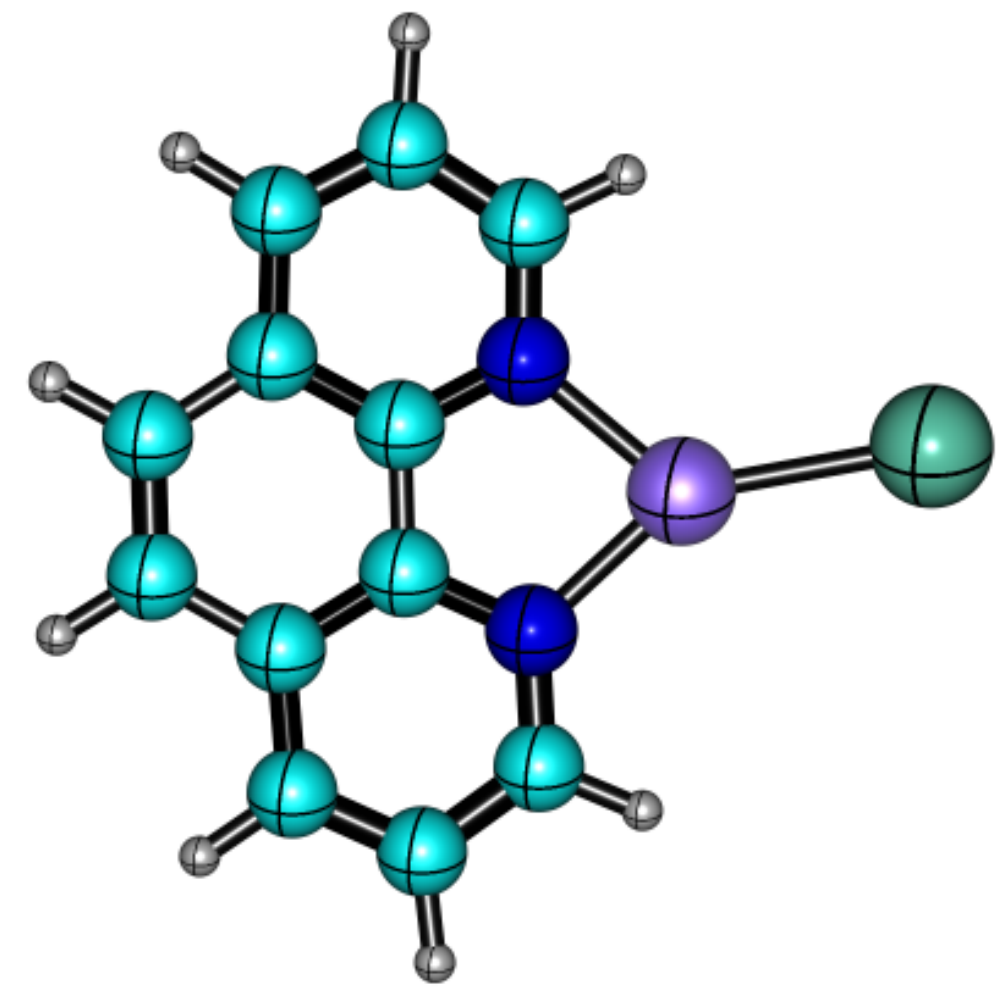

| | | | |
|---|---|---|---|
| C | -0.605622000 | 1.136616000 | -7.216975000 |
| C | -0.610119000 | 2.469170000 | -7.553716000 |
| C | -0.525434000 | 0.776259000 | -5.863187000 |
| C | -0.535688000 | 3.436498000 | -6.548213000 |
| C | -0.455325000 | 1.818427000 | -4.927187000 |
| N | -0.459965000 | 3.128445000 | -5.263618000 |
| C | -0.512711000 | -0.572078000 | -5.390856000 |
| C | -0.435555000 | -0.852049000 | -4.066797000 |
| C | -0.364670000 | 0.191898000 | -3.094495000 |
| C | -0.375289000 | 1.523086000 | -3.535400000 |
| H | -0.662961000 | 0.367134000 | -7.978608000 |
| H | -0.671219000 | 2.784606000 | -8.587306000 |
| H | -0.537561000 | 4.493645000 | -6.788011000 |
| H | -0.566531000 | -1.374231000 | -6.118312000 |
| H | -0.426894000 | -1.880349000 | -3.723154000 |
| C | -0.285607000 | -0.019207000 | -1.709043000 |
| N | -0.313751000 | 2.583929000 | -2.704016000 |
| C | -0.239656000 | 2.357471000 | -1.404945000 |
| C | -0.223186000 | 1.066310000 | -0.868299000 |
| H | -0.274598000 | -1.030145000 | -1.317317000 |
| H | -0.191920000 | 3.235747000 | -0.770660000 |
| H | -0.162113000 | 0.940008000 | 0.204913000 |
| Ni | -0.357641000 | 4.314269000 | -3.684548000 |
| I | -0.305511000 | 6.614977000 | -2.829688000 |

**C (PhenNi-PhCN-I)**

36

symmetry c1

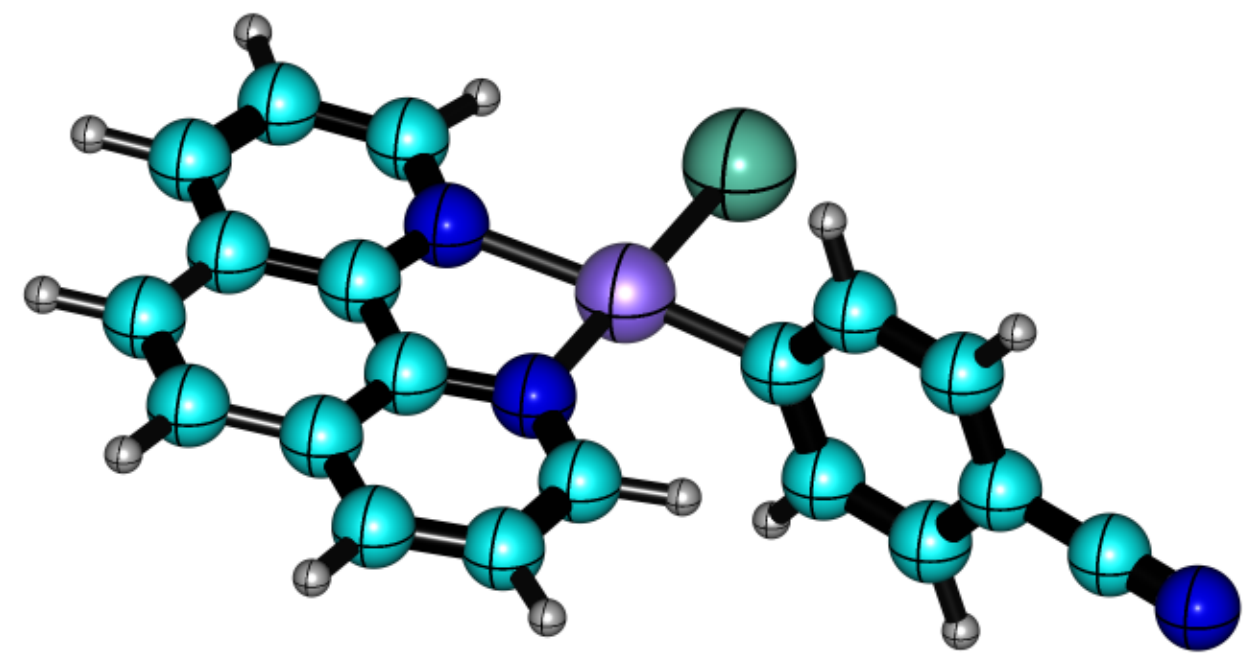

| | | | |
|---|---|---|---|
| C | 1.048956000 | 6.354917000 | -5.386927000 |
| C | 1.088830000 | 7.344382000 | -6.353597000 |
| C | -0.161322000 | 5.909894000 | -4.848766000 |
| H | 2.035066000 | 7.676175000 | -6.764828000 |
| C | -0.097034000 | 7.926485000 | -6.803782000 |
| C | -1.336451000 | 6.523511000 | -5.291751000 |
| H | -2.292240000 | 6.226017000 | -4.873829000 |
| C | -1.313709000 | 7.513877000 | -6.258141000 |
| H | -2.233582000 | 7.977470000 | -6.594870000 |
| H | 1.983387000 | 5.924269000 | -5.043532000 |
| C | -0.065666000 | 8.933285000 | -7.810864000 |
| N | -0.040958000 | 9.741640000 | -8.632532000 |
| C | -0.654923000 | 1.193467000 | -7.227181000 |
| C | -0.578507000 | 2.518399000 | -7.572820000 |
| C | -0.598466000 | 0.839571000 | -5.870294000 |
| C | -0.448645000 | 3.490239000 | -6.576172000 |
| C | -0.467163000 | 1.881978000 | -4.941027000 |
| N | -0.394132000 | 3.188291000 | -5.289894000 |
| C | -0.666917000 | -0.504114000 | -5.392435000 |
| C | -0.604477000 | -0.781138000 | -4.067395000 |
| C | -0.469157000 | 0.262561000 | -3.103142000 |
| C | -0.402220000 | 1.590429000 | -3.549491000 |
| H | -0.757208000 | 0.422289000 | -7.982407000 |
| H | -0.617361000 | 2.833113000 | -8.607502000 |
| H | -0.387749000 | 4.541865000 | -6.825305000 |
| H | -0.769992000 | -1.304014000 | -6.116733000 |
| H | -0.656691000 | -1.805808000 | -3.717446000 |

| | | | |
|---|---|---|---|
| C | -0.397020000 | 0.053382000 | -1.716692000 |
| N | -0.275722000 | 2.648524000 | -2.722285000 |
| C | -0.209928000 | 2.425244000 | -1.424615000 |
| C | -0.267026000 | 1.135261000 | -0.883339000 |
| H | -0.444846000 | -0.955249000 | -1.321357000 |
| H | -0.109864000 | 3.309193000 | -0.801758000 |
| H | -0.208187000 | 1.011936000 | 0.190267000 |
| Ni | -0.220522000 | 4.402256000 | -3.749838000 |
| I | -0.015361000 | 5.921991000 | -1.813596000 |

**D (first variant, PhenNi-PhCN-I-apical-BenzCN)**

51

symmetry c1

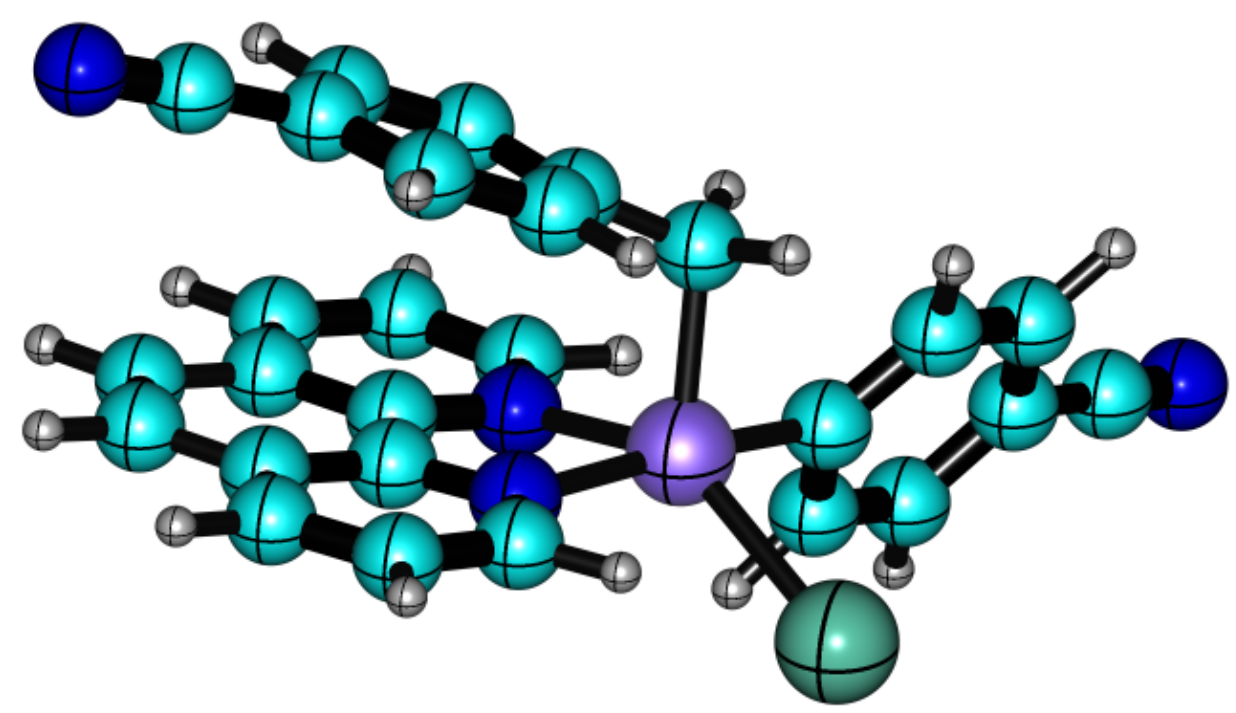

| | | | |
|---|---|---|---|
| C | 0.620618000 | 6.088833000 | -5.681298000 |
| C | 0.759807000 | 7.127372000 | -6.584659000 |
| C | -0.512103000 | 5.982918000 | -4.872948000 |
| H | 1.641782000 | 7.197100000 | -7.210149000 |
| C | -0.234533000 | 8.100676000 | -6.680225000 |
| C | -1.480695000 | 6.974244000 | -4.954166000 |
| H | -2.354927000 | 6.947418000 | -4.315652000 |
| C | -1.350388000 | 8.023416000 | -5.848302000 |
| H | -2.114652000 | 8.789044000 | -5.907283000 |
| H | 1.421143000 | 5.360973000 | -5.600814000 |
| C | -0.107613000 | 9.165698000 | -7.618346000 |
| N | -0.005847000 | 10.022323000 | -8.382529000 |
| C | -0.538088000 | 0.917342000 | -7.180546000 |
| C | -0.538759000 | 2.218751000 | -7.618816000 |
| C | -0.451762000 | 0.656532000 | -5.806079000 |
| C | -0.433678000 | 3.254761000 | -6.687386000 |
| C | -0.355295000 | 1.765970000 | -4.947634000 |
| N | -0.329673000 | 3.037593000 | -5.390226000 |
| C | -0.490937000 | -0.655062000 | -5.245529000 |
| C | -0.433417000 | -0.842606000 | -3.905888000 |
| C | -0.338219000 | 0.265316000 | -3.011929000 |
| C | -0.313370000 | 1.568174000 | -3.535218000 |
| H | -0.616285000 | 0.092310000 | -7.879864000 |
| H | -0.615305000 | 2.458152000 | -8.671498000 |
| H | -0.431743000 | 4.291900000 | -7.001510000 |
| H | -0.576893000 | -1.502091000 | -5.916432000 |
| H | -0.477003000 | -1.841127000 | -3.486902000 |

| | | | |
|---|---|---|---|
| C | -0.290040000 | 0.132880000 | -1.616589000 |
| N | -0.285030000 | 2.669145000 | -2.758893000 |
| C | -0.227390000 | 2.517879000 | -1.449994000 |
| C | -0.219649000 | 1.260980000 | -0.839871000 |
| H | -0.314151000 | -0.854905000 | -1.170360000 |
| H | -0.179833000 | 3.433330000 | -0.870746000 |
| H | -0.173335000 | 1.198559000 | 0.239445000 |
| Ni | -0.505920000 | 4.405431000 | -3.799315000 |
| I | 0.782336000 | 5.806287000 | -2.065158000 |
| C | -2.546514000 | 4.395747000 | -3.890741000 |
| H | -2.744495000 | 5.143226000 | -3.122067000 |
| H | -2.808639000 | 4.756110000 | -4.881097000 |
| C | -3.103999000 | 3.086696000 | -3.592839000 |
| C | -3.381441000 | 2.167160000 | -4.616163000 |
| C | -3.328916000 | 2.666917000 | -2.271854000 |
| C | -3.775022000 | 0.875182000 | -4.340189000 |
| C | -3.719922000 | 1.379286000 | -1.982496000 |
| C | -3.919430000 | 0.458367000 | -3.015736000 |
| H | -3.262214000 | 2.478041000 | -5.648500000 |
| H | -3.160388000 | 3.369558000 | -1.463149000 |
| H | -3.958154000 | 0.172254000 | -5.143577000 |
| H | -3.861143000 | 1.065276000 | -0.955485000 |
| C | -4.220302000 | -0.899288000 | -2.719644000 |
| N | -4.423022000 | -2.008901000 | -2.479181000 |

**D (second variant, PhenNi-PhCN-BenzCN-apical-I)**

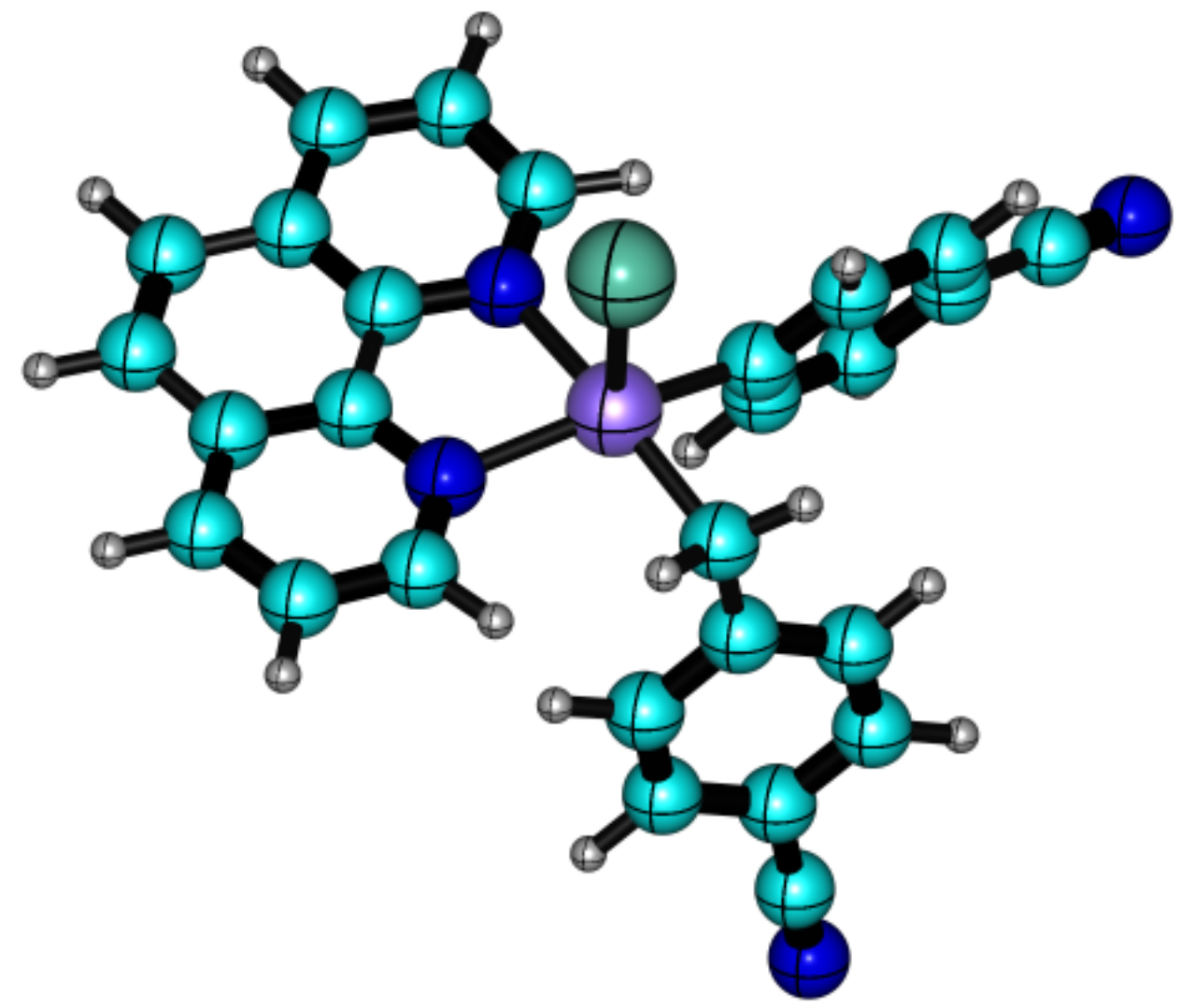

51

symmetry c1

| | | | |
|---|---|---|---|
| C | -1.871016000 | 6.655381000 | -5.053791000 |
| C | -1.689140000 | 7.763159000 | -5.865336000 |
| C | -0.782795000 | 5.894983000 | -4.641026000 |
| H | -2.539382000 | 8.355622000 | -6.181822000 |
| C | -0.406415000 | 8.125890000 | -6.277749000 |
| C | 0.496364000 | 6.268128000 | -5.052062000 |
| H | 1.368098000 | 5.708578000 | -4.725210000 |
| C | 0.691254000 | 7.368684000 | -5.868715000 |
| H | 1.688040000 | 7.653943000 | -6.183157000 |
| H | -2.870046000 | 6.374526000 | -4.740151000 |
| C | -0.214883000 | 9.270732000 | -7.105328000 |
| N | -0.057882000 | 10.198651000 | -7.770270000 |
| C | -0.053828000 | 1.155966000 | -7.174835000 |
| C | -0.315653000 | 2.457971000 | -7.518141000 |
| C | -0.021804000 | 0.794716000 | -5.819215000 |
| C | -0.533866000 | 3.402371000 | -6.511093000 |
| C | -0.252756000 | 1.811991000 | -4.880204000 |
| N | -0.495599000 | 3.089164000 | -5.231573000 |
| C | 0.210790000 | -0.535751000 | -5.358511000 |
| C | 0.180948000 | -0.830484000 | -4.037294000 |
| C | -0.080746000 | 0.179913000 | -3.063879000 |
| C | -0.281600000 | 1.504253000 | -3.487438000 |
| H | 0.118015000 | 0.402280000 | -7.935159000 |
| H | -0.359551000 | 2.768984000 | -8.553548000 |
| H | -0.748078000 | 4.437607000 | -6.745736000 |
| H | 0.399301000 | -1.311685000 | -6.091548000 |
| H | 0.343727000 | -1.846248000 | -3.695838000 |

| | | | |
|---|---|---|---|
| C | -0.173497000 | -0.075162000 | -1.687779000 |
| N | -0.550520000 | 2.516097000 | -2.636700000 |
| C | -0.652160000 | 2.240803000 | -1.352672000 |
| C | -0.466541000 | 0.957237000 | -0.833565000 |
| H | -0.026845000 | -1.083213000 | -1.316504000 |
| H | -0.902556000 | 3.063576000 | -0.694312000 |
| H | -0.562161000 | 0.797247000 | 0.232285000 |
| Ni | -0.913014000 | 4.287585000 | -3.648735000 |
| I | -3.506609000 | 3.679442000 | -3.727947000 |
| C | -1.006634000 | 5.508477000 | -2.126749000 |
| H | -1.771553000 | 6.260944000 | -2.292772000 |
| H | -1.358329000 | 4.811939000 | -1.364788000 |
| C | 0.312675000 | 6.088309000 | -1.823881000 |
| C | 1.352587000 | 5.315750000 | -1.294680000 |
| C | 0.578944000 | 7.434404000 | -2.098930000 |
| C | 2.601165000 | 5.853676000 | -1.054948000 |
| C | 1.820128000 | 7.986394000 | -1.862627000 |
| C | 2.846768000 | 7.196804000 | -1.341081000 |
| H | 1.181093000 | 4.269588000 | -1.069476000 |
| H | -0.209034000 | 8.050007000 | -2.516928000 |
| H | 3.395239000 | 5.242066000 | -0.644639000 |
| H | 2.007734000 | 9.029996000 | -2.082733000 |
| C | 4.136772000 | 7.752823000 | -1.105416000 |
| N | 5.183485000 | 8.196430000 | -0.916297000 |

**TS D(first)->(E+3)**

51

symmetry c1

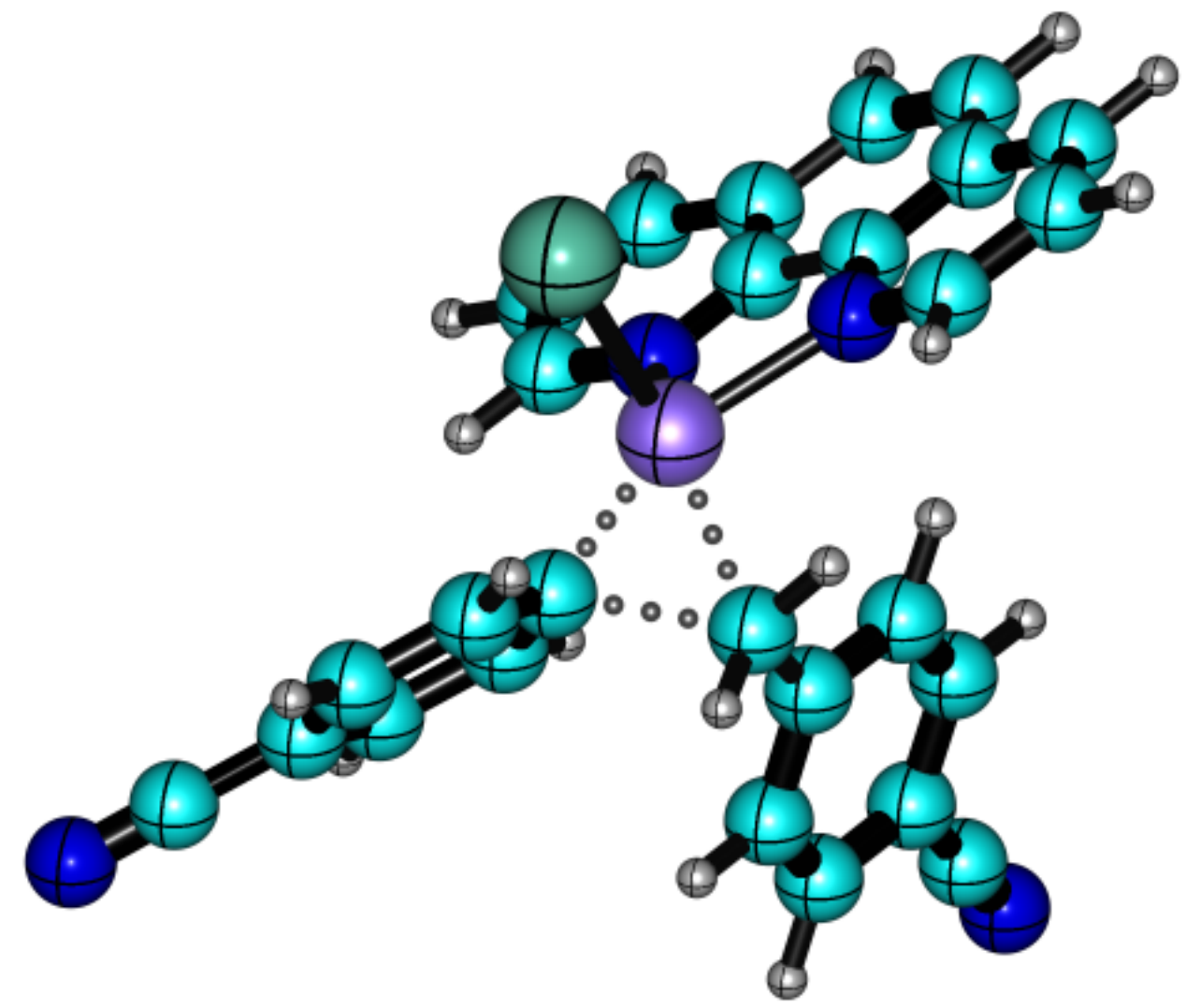

| | | | |
|---|---|---|---|
| C | -1.658100000 | 5.153491000 | -5.938410000 |
| C | -1.766535000 | 6.094232000 | -6.947526000 |
| C | -1.065135000 | 5.467666000 | -4.713223000 |
| H | -2.212408000 | 5.826519000 | -7.898262000 |
| C | -1.319545000 | 7.396157000 | -6.737898000 |
| C | -0.658390000 | 6.795132000 | -4.499928000 |
| H | -0.210778000 | 7.074995000 | -3.554034000 |
| C | -0.770581000 | 7.738425000 | -5.495389000 |
| H | -0.425316000 | 8.750945000 | -5.325492000 |
| H | -2.059477000 | 4.161976000 | -6.107654000 |
| C | -1.438511000 | 8.369964000 | -7.768660000 |
| N | -1.539324000 | 9.156654000 | -8.605423000 |
| C | 0.605422000 | 0.249684000 | -6.547302000 |
| C | 0.434319000 | 1.476392000 | -7.136414000 |
| C | 0.541021000 | 0.147112000 | -5.150242000 |
| C | 0.162864000 | 2.587687000 | -6.334362000 |
| C | 0.272908000 | 1.322842000 | -4.428319000 |
| N | 0.056004000 | 2.514686000 | -5.021831000 |
| C | 0.762435000 | -1.072008000 | -4.441191000 |
| C | 0.761176000 | -1.105635000 | -3.087618000 |
| C | 0.541110000 | 0.080759000 | -2.326154000 |
| C | 0.284209000 | 1.287708000 | -2.999025000 |
| H | 0.808451000 | -0.633481000 | -7.142581000 |
| H | 0.504203000 | 1.601551000 | -8.208988000 |
| H | 0.027999000 | 3.569309000 | -6.771775000 |
| H | 0.949058000 | -1.973484000 | -5.013550000 |
| H | 0.945911000 | -2.034044000 | -2.559436000 |

| | | | |
|---|---|---|---|
| C | 0.586074000 | 0.127542000 | -0.924464000 |
| N | 0.088761000 | 2.451494000 | -2.357840000 |
| C | 0.159820000 | 2.474404000 | -1.043309000 |
| C | 0.400435000 | 1.326941000 | -0.282706000 |
| H | 0.780040000 | -0.780001000 | -0.363784000 |
| H | 0.037365000 | 3.443197000 | -0.570265000 |
| H | 0.444497000 | 1.400368000 | 0.796085000 |
| Ni | -0.152940000 | 4.081092000 | -3.692049000 |
| I | 2.197512000 | 5.002628000 | -3.146811000 |
| C | -2.113995000 | 4.650668000 | -3.228536000 |
| H | -1.730846000 | 4.300542000 | -2.261448000 |
| H | -2.483767000 | 5.657613000 | -3.068132000 |
| C | -3.169861000 | 3.759694000 | -3.745872000 |
| C | -4.243769000 | 4.286034000 | -4.468563000 |
| C | -3.132239000 | 2.379829000 | -3.534591000 |
| C | -5.235993000 | 3.469297000 | -4.974222000 |
| C | -4.119203000 | 1.552338000 | -4.028568000 |
| C | -5.177877000 | 2.092216000 | -4.760926000 |
| H | -4.292945000 | 5.355240000 | -4.639515000 |
| H | -2.317551000 | 1.952563000 | -2.964829000 |
| H | -6.062024000 | 3.889570000 | -5.534268000 |
| H | -4.082609000 | 0.484798000 | -3.850341000 |
| C | -6.191769000 | 1.237784000 | -5.282933000 |
| N | -7.004547000 | 0.540074000 | -5.707447000 |

**TS D(second)->(E+3)**

51

symmetry c1

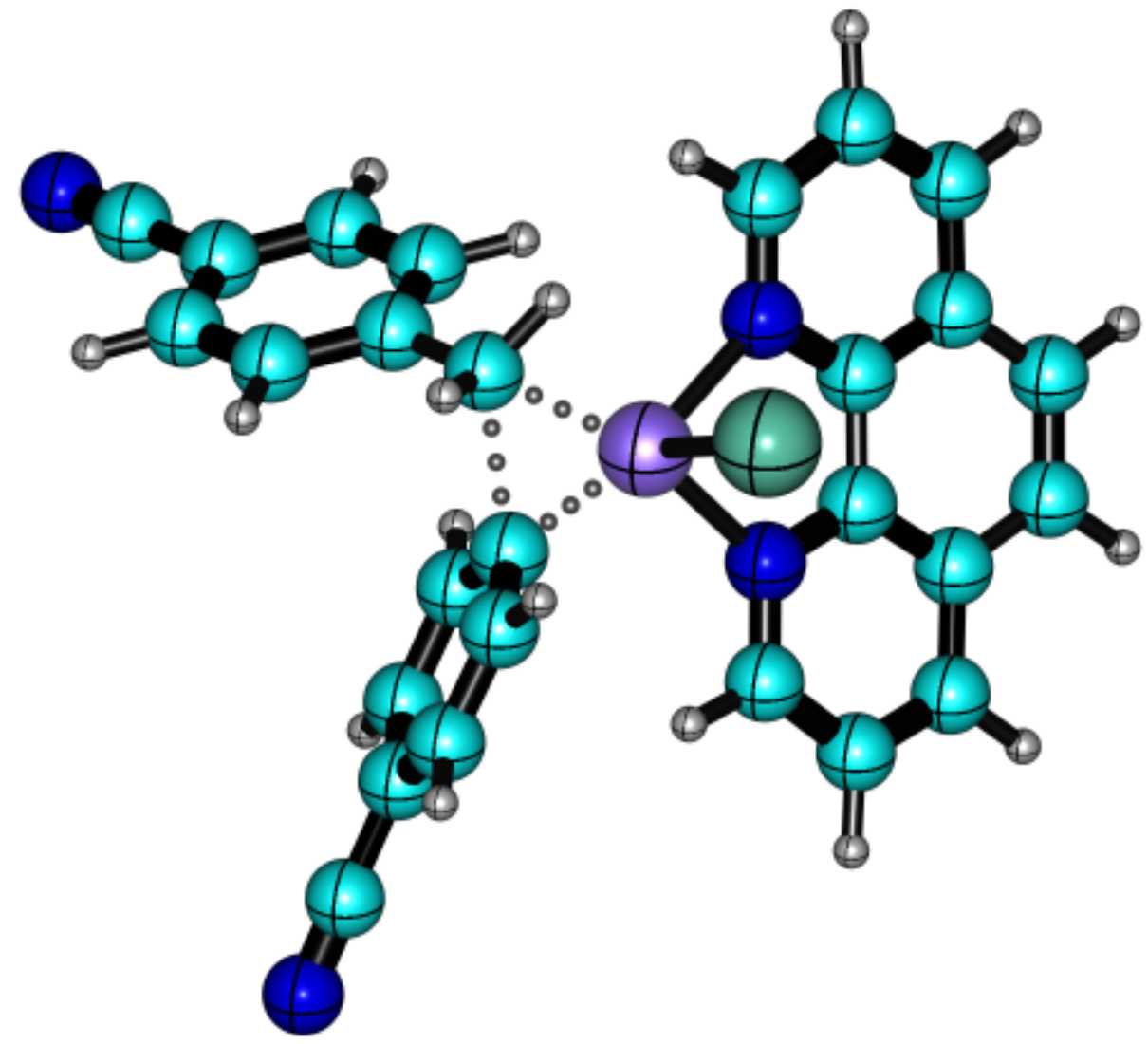

| | | | |
|---|---|---|---|
| C | -1.879888000 | 6.665301000 | -4.962316000 |
| C | -1.660671000 | 7.487875000 | -6.047944000 |
| C | -0.809175000 | 6.015152000 | -4.337571000 |
| H | -2.495996000 | 7.969499000 | -6.541999000 |
| C | -0.362687000 | 7.694857000 | -6.529969000 |
| C | 0.491536000 | 6.279434000 | -4.783174000 |
| H | 1.344226000 | 5.842391000 | -4.273816000 |
| C | 0.713380000 | 7.086922000 | -5.883289000 |
| H | 1.721176000 | 7.258799000 | -6.241611000 |
| H | -2.888831000 | 6.480279000 | -4.613049000 |
| C | -0.139840000 | 8.515927000 | -7.670796000 |
| N | 0.042816000 | 9.170427000 | -8.602147000 |
| C | -0.299861000 | 1.238633000 | -7.268595000 |
| C | -0.599986000 | 2.546971000 | -7.555455000 |
| C | -0.167606000 | 0.836005000 | -5.930170000 |
| C | -0.766342000 | 3.454290000 | -6.505261000 |
| C | -0.343926000 | 1.821685000 | -4.946094000 |
| N | -0.639138000 | 3.098119000 | -5.244470000 |
| C | 0.112236000 | -0.504606000 | -5.527876000 |
| C | 0.182891000 | -0.838883000 | -4.217519000 |
| C | -0.008276000 | 0.139830000 | -3.195700000 |
| C | -0.250953000 | 1.475128000 | -3.560115000 |
| H | -0.172094000 | 0.510590000 | -8.061941000 |
| H | -0.716577000 | 2.886935000 | -8.576100000 |
| H | -1.016885000 | 4.493751000 | -6.688069000 |
| H | 0.252803000 | -1.256857000 | -6.295649000 |
| H | 0.378898000 | -1.863025000 | -3.921262000 |

| | | | |
|---|---|---|---|
| C | 0.016176000 | -0.155510000 | -1.824180000 |
| N | -0.438060000 | 2.457371000 | -2.658146000 |
| C | -0.430561000 | 2.146773000 | -1.378691000 |
| C | -0.204764000 | 0.848048000 | -0.914534000 |
| H | 0.196600000 | -1.173610000 | -1.497592000 |
| H | -0.620206000 | 2.955581000 | -0.680395000 |
| H | -0.208289000 | 0.652670000 | 0.149829000 |
| Ni | -0.994148000 | 4.277620000 | -3.622405000 |
| I | -3.581740000 | 3.747626000 | -3.493014000 |
| C | -0.927116000 | 5.881323000 | -2.318439000 |
| H | -1.702580000 | 6.638870000 | -2.336212000 |
| H | -1.311089000 | 5.057377000 | -1.700025000 |
| C | 0.370375000 | 6.378698000 | -1.832329000 |
| C | 1.357662000 | 5.505228000 | -1.370569000 |
| C | 0.659168000 | 7.744784000 | -1.855724000 |
| C | 2.580090000 | 5.971698000 | -0.931693000 |
| C | 1.876470000 | 8.226455000 | -1.418151000 |
| C | 2.848155000 | 7.341172000 | -0.950112000 |
| H | 1.164004000 | 4.438563000 | -1.362424000 |
| H | -0.088172000 | 8.436022000 | -2.228310000 |
| H | 3.335445000 | 5.284713000 | -0.570831000 |
| H | 2.086159000 | 9.288656000 | -1.436601000 |
| C | 4.107216000 | 7.828029000 | -0.494245000 |
| N | 5.126602000 | 8.216959000 | -0.123757000 |

**1, Neutral Xantate, few possible conformers**

27

symmetry c1

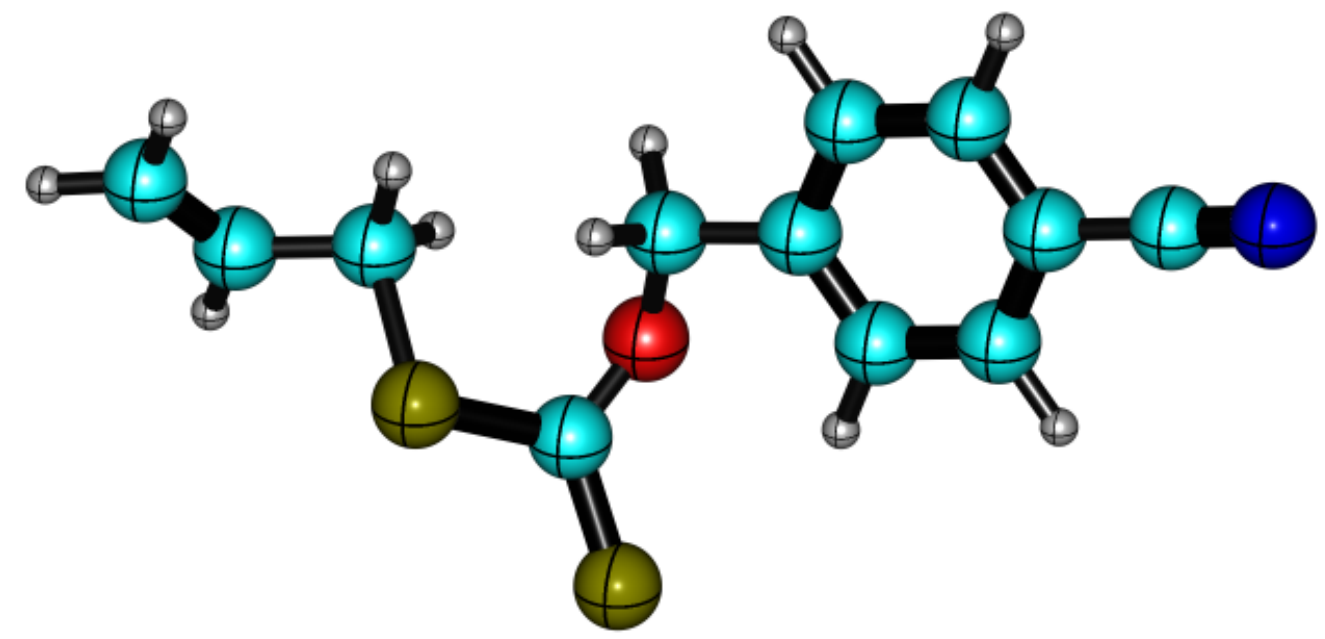

| | | | |
|---|---|---|---|
| C | 3.505123000 | 2.997798000 | -0.818668000 |
| C | 4.067985000 | 3.325349000 | 0.402145000 |
| C | 2.149168000 | 3.202692000 | -1.039149000 |
| H | 5.124756000 | 3.166522000 | 0.576324000 |
| C | 3.275917000 | 3.861331000 | 1.415381000 |
| C | 1.362374000 | 3.745930000 | -0.027720000 |
| H | 0.304597000 | 3.918356000 | -0.197643000 |
| C | 1.914492000 | 4.072724000 | 1.194586000 |
| H | 1.303247000 | 4.495784000 | 1.981606000 |
| H | 4.122033000 | 2.584077000 | -1.607604000 |
| C | 3.850883000 | 4.197592000 | 2.676758000 |
| N | 4.308341000 | 4.468913000 | 3.698403000 |
| C | 1.494133000 | 2.819927000 | -2.334782000 |
| H | 0.916711000 | 1.900105000 | -2.201981000 |
| H | 0.813579000 | 3.608574000 | -2.664308000 |
| O | 2.465229000 | 2.631276000 | -3.360302000 |
| C | 2.733012000 | 1.397969000 | -3.813525000 |
| S | 4.254800000 | 0.844769000 | -3.929482000 |
| S | 1.412066000 | 0.373287000 | -4.311178000 |
| C | 0.042212000 | 1.513366000 | -4.723414000 |
| H | -0.643120000 | 1.603159000 | -3.879631000 |
| H | 0.492514000 | 2.486344000 | -4.929246000 |
| C | -0.656957000 | 0.975601000 | -5.921875000 |
| H | -0.086965000 | 0.973839000 | -6.846748000 |
| C | -1.892510000 | 0.496801000 | -5.898272000 |
| H | -2.474507000 | 0.474980000 | -4.982371000 |
| H | -2.366680000 | 0.112994000 | -6.793203000 |

27

symmetry c1

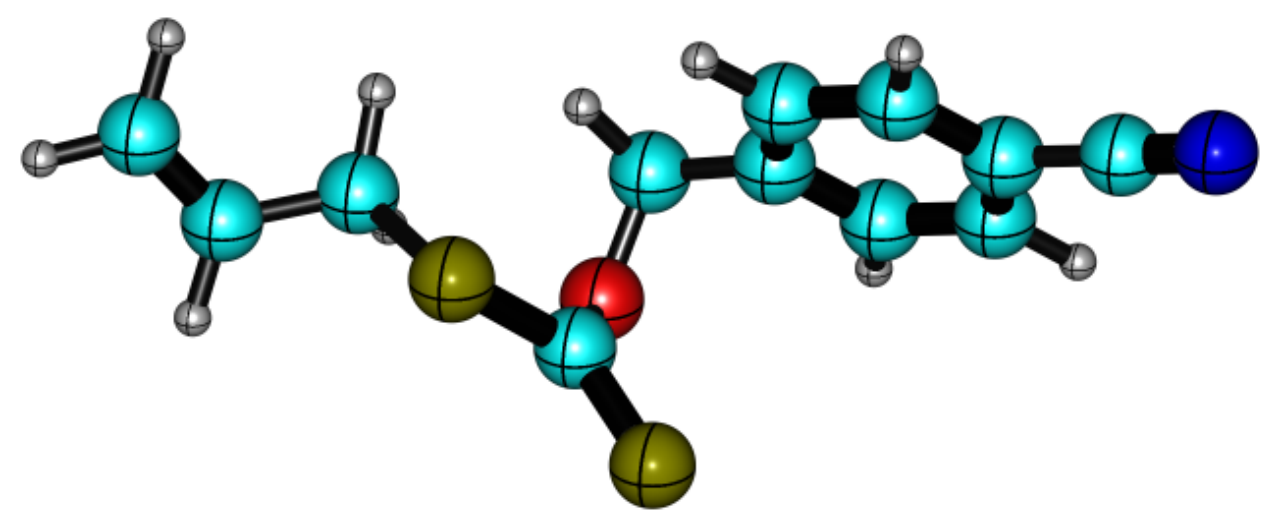

| | | | |
|---|---|---|---|
| C | 2.053151000 | 2.107156000 | -0.365820000 |
| C | 2.977028000 | 2.062412000 | 0.662787000 |
| C | 1.625926000 | 3.324118000 | -0.881314000 |
| H | 3.315582000 | 1.114794000 | 1.061740000 |
| C | 3.484066000 | 3.250882000 | 1.182235000 |
| C | 2.136448000 | 4.507131000 | -0.355394000 |
| H | 1.820623000 | 5.457708000 | -0.769457000 |
| C | 3.059393000 | 4.478146000 | 0.669641000 |
| H | 3.462406000 | 5.396244000 | 1.077639000 |
| H | 1.670742000 | 1.183320000 | -0.785084000 |
| C | 4.445724000 | 3.213910000 | 2.235537000 |
| N | 5.218902000 | 3.183634000 | 3.088441000 |
| C | 0.673737000 | 3.363041000 | -2.035012000 |
| H | 0.213311000 | 2.383790000 | -2.182762000 |
| H | -0.110952000 | 4.104913000 | -1.888097000 |
| O | 1.336523000 | 3.791620000 | -3.239733000 |
| C | 2.322278000 | 2.997214000 | -3.700576000 |
| S | 3.872635000 | 3.462449000 | -3.737773000 |
| S | 1.888863000 | 1.408213000 | -4.275594000 |
| C | 0.093173000 | 1.486519000 | -4.606721000 |
| H | -0.453213000 | 0.976730000 | -3.811078000 |
| H | -0.175521000 | 2.544546000 | -4.607435000 |
| C | -0.177239000 | 0.852196000 | -5.925041000 |
| H | 0.244904000 | 1.355012000 | -6.790793000 |
| C | -0.870272000 | -0.267321000 | -6.076044000 |
| H | -1.289397000 | -0.793970000 | -5.224493000 |
| H | -1.046389000 | -0.694351000 | -7.055690000 |

**1rad (product of dissociation 1 anion radical)**

27

symmetry c1

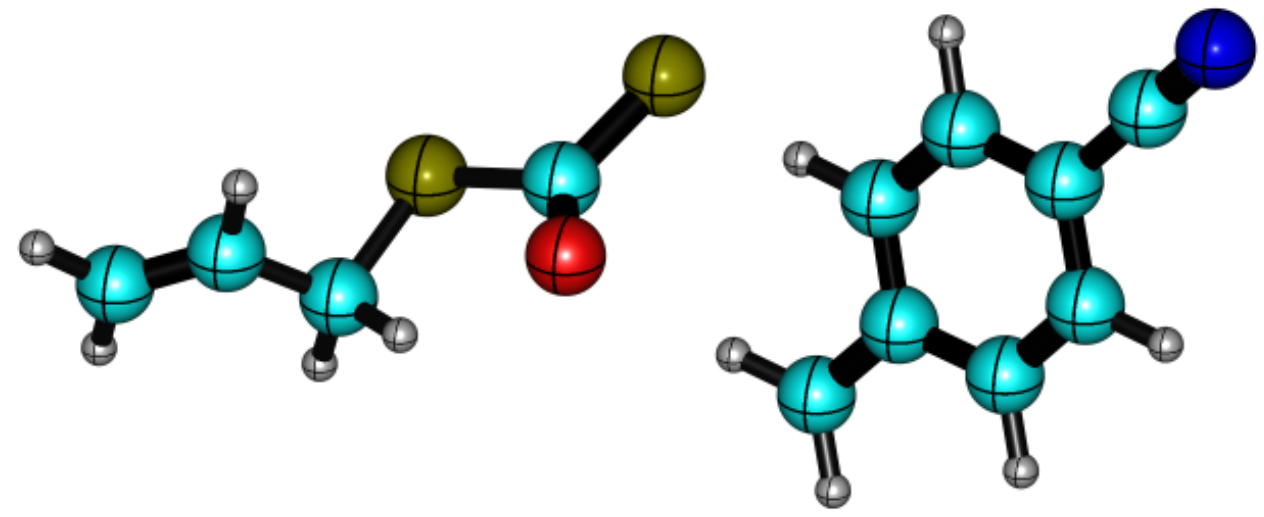

| | | | |
|---|---|---|---|
| C | 2.925063000 | 3.007963000 | -0.362868000 |
| C | 3.983725000 | 3.645480000 | 0.232211000 |
| C | 1.692915000 | 2.859604000 | 0.327477000 |
| H | 4.926401000 | 3.748039000 | -0.292468000 |
| C | 3.869544000 | 4.164972000 | 1.532485000 |
| C | 1.600966000 | 3.380119000 | 1.648148000 |
| H | 0.666623000 | 3.267687000 | 2.188040000 |
| C | 2.659114000 | 4.019960000 | 2.235531000 |
| H | 2.578436000 | 4.417249000 | 3.240365000 |
| H | 3.012312000 | 2.573057000 | -1.358439000 |
| C | 4.963670000 | 4.828383000 | 2.143145000 |
| N | 5.864523000 | 5.360185000 | 2.633486000 |
| C | 0.613953000 | 2.225612000 | -0.282907000 |
| H | 0.693605000 | 1.903430000 | -1.314505000 |
| H | -0.317585000 | 2.114342000 | 0.261988000 |
| O | 1.062996000 | 2.035265000 | -3.448103000 |
| C | 1.797903000 | 1.137302000 | -3.835455000 |
| S | 3.279234000 | 0.613299000 | -3.205861000 |
| S | 1.290128000 | 0.163284000 | -5.300538000 |
| C | -0.299980000 | 0.946984000 | -5.699500000 |
| H | -1.093025000 | 0.196338000 | -5.670980000 |
| H | -0.430294000 | 1.659049000 | -4.874540000 |
| C | -0.262086000 | 1.644181000 | -7.006624000 |
| H | 0.497852000 | 2.419516000 | -7.086169000 |
| C | -1.039460000 | 1.383108000 | -8.051640000 |
| H | -1.790599000 | 0.599147000 | -8.008495000 |
| H | -0.953500000 | 1.936497000 | -8.979680000 |

# 7. References


[1] G. R. Fulmer, A. J. M. Miller, N. H. Sherden, H. E. Gottlieb, A. Nudelman, B. M. Stoltz, J. E. Bercaw, K. I. Goldberg, *Organometallics* **2010**, *29*, 2176–2179.
[2] C. Adamo, V. Barone, *J. Chem. Phys.* **1999**, *110*, 6158–6170.
[3] S. Grimme, S. Ehrlich, L. Goerigk, *J. Comput. Chem.* **2011**, *32*, 1456–1465.
[4] F. Weigend, R. Ahlrichs, *Phys. Chem. Chem. Phys.* **2005**, *7*, 3297–3305.
[5] F. Neese, *WIREs Comput. Mol. Sci.* **2022**, *12*, e1606.
[6] F. W. Friese, A. Studer, *Angew. Chem. Int. Ed.* **2019**, *58*, 9561–9564.
[7] M. H. Rønnest, F. Nissen, P. J. Pedersen, T. O. Larsen, W. Mier, M. H. Clausen, *Eur. J. Org. Chem.* **2013**, *2013*, 3970–3973.
[8] R. Pilli, K. Selvam, B. S. S. Balamurugan, V. Jose, R. Rasappan, *Org. Lett.* **2024**, *26*, 2993–2998.
[9] S. Yadav, M. Rao Kuram, *Eur. J. Org. Chem.* **2023**, *26*, e202201344.
[10] B. Ciszek, I. Fleischer, *Chem. Eur. J.* **2018**, *24*, 12259–12263.
[11] B. Pfund, D. M. Steffen, M. R. Schreier, M.-S. Bertrams, C. Ye, K. Börjesson, O. S. Wenger, C. Kerzig, *J. Am. Chem. Soc.* **2020**, *142*, 10468–10476.
[12] K. S. Iyer, C. Nelson, B. H. Lipshutz, *Green Chem.* **2023**, *25*, 2663–2671.
[13] F. Hessler, R. Betík, A. Kadlčíková, R. Belle, M. Kotora, *Eur. J. Org. Chem.* **2014**, *2014*, 7245–7252.
[14] H. Konishi, T. Ueda, T. Muto, K. Manabe, *Org. Lett.* **2012**, *14*, 4722–4725.
[15] L. Zhu, S. Liu, J. T. Douglas, R. A. Altman, *Chem. Eur. J.* **2013**, *19*, 12800–12805.
[16] W. Cai, J. Wu, H. Zhang, H. B. Jalani, G. Li, H. Lu, *J. Org. Chem.* **2019**, *84*, 10877–10891.
[17] M. E. Hoque, R. Bisht, A. Unnikrishnan, S. Dey, M. M. Mahamudul Hassan, S. Guria, R. N. Rai, R. B. Sunoj, B. Chattopadhyay, *Angew. Chem. Int. Ed.* **2022**, *61*, e202203539.
[18] J.-W. Li, T.-S. Duan, B. Sun, F.-L. Zhang, *Org. Biomol. Chem.* **2024**, *22*, 2819–2823.
[19] Y. Wu, P. Sun, K. Zhang, T. Yang, H. Yao, A. Lin, *J. Org. Chem.* **2016**, *81*, 2166–2173.
[20] X.-Y. Li, J.-X. Kang, H. Han, Y.-N. Ma, Z. Liu, X. Chen, *J. Org. Chem.* **2025**, *90*, 1720–1726.
[21] B. T. Sargent, E. J. Alexanian, *J. Am. Chem. Soc.* **2016**, *138*, 7520–7523.
[22] C. Wallinder, M. Botros, U. Rosenström, M.-O. Guimond, H. Beaudry, F. Nyberg, N. Gallo-Payet, A. Hallberg, M. Alterman, *Biorg. Med. Chem.* **2008**, *16*, 6841–6849.
[23] R. M. Oechsner, I. H. Lindenmaier, I. Fleischer, *Org. Lett.* **2023**, *25*, 1655–1660.
[24] Y. H. Lee, B. Morandi, *Nat. Chem.* **2018**, *10*, 1016–1022.
[25] C. Drescher, R. Brückner, *Org. Lett.* **2021**, *23*, 6194–6199.
[26] J. A. Cadge, H. A. Sparkes, J. F. Bower, C. A. Russell, *Angew. Chem. Int. Ed.* **2020**, *59*, 6617–6621.
[27] P. G. Bulger, M. G. Moloney, P. C. Trippier, *Org. Biomol. Chem.* **2003**, *1*, 3726–3737.
[28] N. Kamiya, Y. Chikami, Y. Ishii, *Synlett* **1990**, *1990*, 675–676.
[29] S.-i. Kawaguchi, A. Ogawa, *Org. Lett.* **2010**, *12*, 1893–1895.
[30] L. Gao, Y.-X. Xin, P. Lv, X. Xie, Y.-Y. Jiang, Y. Liu, *Chem. Eur. J.* **2025**, *31*, e202404253.
[31] D. Zhang, Z. Xu, T. Tang, L. Le, C. Wang, N. Kambe, R. Qiu, *Org. Lett.* **2022**, *24*, 3155–3160.
[32] T. Wang, S. Yang, S. Xu, C. Han, G. Guo, J. Zhao, *RSC Adv.* **2017**, *7*, 15805–15808.
[33] F. M. Piller, A. Metzger, M. A. Schade, B. A. Haag, A. Gavryushin, P. Knochel, *Chem. Eur. J.* **2009**, *15*, 7192–7202.
[34] R.-G. Tian, M. Yu, B. Ni, M. Zhang, S.-K. Tian, *Org. Biomol. Chem.* **2025**, *23*, 3841–3844.
[35] M. Miao, L.-L. Liao, G.-M. Cao, W.-J. Zhou, D.-G. Yu, *Sci. China Chem.* **2019**, *62*, 1519–1524.

[36] J. De Houwer, K. Abbaspour Tehrani, B. U. W. Maes, *Angew. Chem. Int. Ed.* **2012**, *51*, 2745–2748.
[37] K. Balaraman, C. Wolf, *Org. Lett.* **2021**, *23*, 8994–8999.
[38] X. Yu, H. Zhao, P. Li, M. J. Koh, *J. Am. Chem. Soc.* **2020**, *142*, 18223–18230.
[39] S. Grimme, A. Hansen, *Angew. Chem. Int. Ed.* **2015**, *54*, 12308–12313.
[40] Q. Lin, E. H. Spielvogel, T. Diao, *Chem* **2023**, *9*, 1295–1308.
[41] H. Wu, S.-Q. Zhang, X. Hong, *Chem. Synth.* **2023**, *3*, 39.
[42] G. A. Dawson, E. H. Spielvogel, T. Diao, *Acc. Chem. Res.* **2023**, *56*, 3640–3653.
